# Effects of Aerobic Exercise Training Combined with Abdominal Massage in Functional Constipation: A Randomized Controlled Study

**DOI:** 10.5152/tjg.2026.101126

**Published:** 2026-07-01

**Authors:** İrem Gül Doğan, Ege Nur Atabey Gerlegiz, Türkan Akbayrak, Hatice Yasemin Balaban, Cem Şimşek, Beyza Atay, Serap Özgül

**Affiliations:** 1 Institute of Health Sciences, Hacettepe University Faculty of Physical Therapy and Rehabilitation, Ankara, Türkiye; 2 Department of Fundamental Physiotherapy and Rehabilitation, Hacettepe University Faculty of Physical Therapy and Rehabilitation, Ankara, Türkiye; 3 Division of Gastroenterology, Department of Internal Medicine, Hacettepe University Faculty of Medicine, Ankara, Türkiye

## Abstract

**Background/Aims:**

Various studies have investigated the effects of abdominal massage (AM) and aerobic exercise training (AET) in managing functional constipation (FC). These studies have shown that both AM and AET alone have beneficial effects on FC. However, to the author’s knowledge, no study has combined both AM and AET to examine their joint effects. Therefore, the aim of this study was to investigate the additional effects of AET on AM in individuals diagnosed with FC using a randomized controlled design.

**Materials and Methods:**

Participants aged 18-65 years who were diagnosed with FC according to the Rome IV criteria were randomly assigned to either the intervention group (patient education + AM + AET) or the control group (patient education + AM). AM and AET were performed in the clinic 3 days per week, with a recommended home program for 2 days per week (self-massage). AET consisted of walking on a treadmill or at home, and exercise intensity was determined using the Heart Rate Reserve method or the BORG scale. The primary outcome was defecation frequency, whereas secondary outcomes included defecation duration, frequency of laxative use, average stool consistency, constipation severity, gastrointestinal symptom severity, constipation-related quality of life, abdominal muscle strength, and functional exercise capacity. Data were analyzed using Wilcoxon and Mann–Whitney *U*-tests, with statistical significance set at *P* < .05.

**Results:**

Both groups showed statistically significant improvements in primary and secondary outcomes over time (*P* < .05). However, the intervention group (AM + AET) exhibited significantly greater improvements compared to the control group.

**Conclusion:**

Adding aerobic exercise to abdominal massage in individuals with FC results in superior outcomes in physical fitness, defecation frequency, duration, stool characteristics, symptom severity, and quality of life. Multimodal and holistic approaches should be prioritized in the conservative management of functional constipation.

Table 1.Characteristics of the Intervention and Control Groups

functional constipation (FC), n; number of patients

Table 2.Comparison of Primary Outcome Measurement Within and Between Groups

Data are presented as mean (SD).

n, number of patients. SD standart deviation.

^a^Wilcoxon test (within-group comparison).

^b^Mann–Whitney *U*-test (between-group comparison).

Table 3.Comparison of Secondary Outcome Measurements Within and Between Groups

Data are presented as mean (SD).

n, number of patients. SD standart deviation.

^a^Wilcoxon test (within-group comparison).

^b^Mann–Whitney *U*-test (between-group comparison).

^c^Fisher test.

## SS-04 Acute Hepatitis Agent Seen for the First Time in Eastern Anatolia; Sandfly Fever: Could the Vector Have Emerged in the Region After the Earthquake?

**Muhammed Furkan Keser ^1^ , Yahya Atayan ^1^ , Zeynep Büşra Keser ^2^ , Mehmet Ali Erdoğan ^1^ , Yüksel Seçkin ^1^ , Yasir Furkan Çağın ^1^ , Murat Harputluoğlu ^1^ , Oğuzhan Yıldırım ^1^**

^1^Department of Gastroenterology, İnönü University Faculty of Medicine, Malatya, Türkiye

^2^Department of Infectious Diseases and Clinical Microbiology, İnönü University Faculty of Medicine, Malatya, Türkiye

**Background/Aims: **Sandfly fever, caused by Phlebovirus species of the Bunyaviridae family, is typically a self-limiting viral infection; however, hepatic involvement may occur. This study aimed to present, for the first time in Eastern Anatolia, cases of sandfly fever associated with acute hepatitis, which were followed in the gastroenterology clinic, and to evaluate their clinical, laboratory, radiological, and inflammatory parameters from a gastroenterology perspective.

**Materials and Methods:** Patients admitted to a university hospital with constitutional symptoms and hospitalized in the gastroenterology clinic with a preliminary diagnosis of acute hepatitis were retrospectively analyzed. Other viral hepatitis and herpesviruses were excluded. Serum samples were sent to the National Arbovirus and Viral Zoonoses Laboratory; IgM positivity for sandfly fever was accepted as a definitive diagnosis. Demographic data, clinical findings, laboratory parameters, radiological results, and inflammatory markers were evaluated.

**Results:** Fifteen patients were included (7 females, 8 males; median age 27 years). The most common symptoms were fatigue (100%), myalgia (86.7%), fever (80%), and abdominal pain (60%). The median length of hospitalization was 6 days. Thrombocytopenia was present in 87% and neutropenia in 66% of cases. AST (median 794 U/L) and ALT (median 878 U/L) levels exceeded 10 times the upper normal limit in 93% of patients. Two cases exhibited ultrasonographic findings consistent with acute hepatitis. Inflammatory markers were frequently increased: CRP was increased in 73% of patients, and ferritin levels (median, 1057 ng/mL) were increased in 60% of cases, suggesting a potential biomarker for hepatic involvement.

**Conclusion:** For the first time, sandfly fever presenting with acute hepatitis was identified in Eastern Anatolia and systematically evaluated in a gastroenterology clinic. The disease may present with an acute hepatitis clinic and should be considered among rare etiologies. Post-earthquake environmental changes may have contributed to vector emergence. Further studies are warranted to clarify hepatic involvement and the role of inflammatory biomarkers.

## SS-06 Effects of Earthquake Fear and Posttraumatic Stress Disorder on Patients with Irritable Bowel Syndrome After a Major Earthquake in Türkiye: A Multicenter Study

**Rasim Eren Cankurtaran ^1^ , Hulusi Can Karpuzcu ^1^ , Engin Ataman ^2^ , Gökhan Aydın ^3^ , Kenan Koşar ^4^ , Sedat Çiçek ^4^ , Fatih Kıvrakoğlu ^5^ , Batuhan Başpınar ^6^ , Emre Dirican ^7^**

^1^Department of Gastroenterology, Ankara Etlik City Hospital, Ankara, Türkiye

^2^Department of Gastroenterology, Kahramanmaraş Necip Fazıl City Hospital, Kahramanmaraş, Türkiye

^3^Department of Gastroenterology, İskenderun State Hospital, Hatay, Türkiye

^4^Department of Gastroenterology, Adıyaman Training and Research Hospital, Adıyaman, Türkiye

^5^Department of Gastroenterology, Osmaniye State Hospital, Osmaniye, Türkiye

^6^Department of Gastroenterology, Kilis Prof. Dr. Alaeddin Yavaşca State Hospital, Kilis, Türkiye

^7^Department of Medical Informatics and Biostatistics, Mustafa Kemal University Faculty of Medicine, Hatay, Türkiye

**Background/Aims:** On February 6, 2023, 2 devastating earthquakes struck southeastern Türkiye, causing over 100 000 injuries and more than 50 000 deaths. This study aimed to investigate the impact of posttraumatic stress disorder (PTSD) and fear of earthquakes on the severity of irritable bowel syndrome (IBS) symptoms and IBS-related quality of life (IBS-QoL).

**Materials and Methods: **Participants diagnosed with IBS were categorized into 2 groups: those residing in earthquake zones and those in non-earthquake zones. Data regarding demographic characteristics, the Irritable Bowel Syndrome Symptom Severity Scale (IBS-SSS), the Irritable Bowel Syndrome Quality of Life (IBS-QoL), the Posttraumatic Stress Disorder Checklist for DSM-5 (PCL-5), and the Fear of Earthquake Scale (FES) were collected through validated questionnaires. Multivariate analyses, multiple linear regression, and elastic net models were performed to identify predictors of IBS-SSS and IBS-QoL scores.

**Results:** A total of 225 patients with IBS were included, with 117 (52%) from earthquake zones and 108 (48%) from non-earthquake zones. Mean IBS-SSS, IBS-QoL, FES, and PCL-5 scores were significantly higher in the earthquake group compared to the non-earthquake group (249 vs. 141; 44.1 vs. 22.8; 20 vs. 9; 47 vs. 28, respectively; *P* < .001 for all). PCL-5 and FES scores were independent predictors of IBS-SSS (OR = 1.057, *P* < .001 and OR = 1.082, *P* = .019). In regression and elastic net models, PCL-5 (*P* < .001) was the strongest predictor (100%) and FES (*P* = .006) the second (38%) for IBS-QoL.

**Conclusion:** PTSD and earthquake-related fear significantly impact IBS symptom severity and quality of life. A holistic treatment approach addressing psychosomatic and mental health factors may improve outcomes in patients with IBS.

## SS-07 Is It Possible to Predict Hemorrhage After Endoscopic Papillectomy?

**Hakan Şentürk ^1^ , Koray Koçhan ^2^ , Sercan Kiremitçi ^1^ , İbrahim Hakkı Köker ^3^ , Şerife Değirmencioğlu Tosun ^4^ , Ali Tüzün İnce ^1^**

^1^Department of Gastroenterology, Bezmialem Foundation University Faculty of Medicine, İstanbul, Türkiye

^2^Department of Gastroenterology, Biruni University Faculty of Medicine, İstanbul, Türkiye

^3^Department of Gastroenterology, Başkent University Faculty of Medicine, İstanbul, Türkiye

^4^Department of Gastroenterology, University of Health Sciences Bağcılar Training and Research Hospital, İstanbul, Türkiye

**Background/Aims:** Endoscopic papillectomy (EP) is an effective intervention for the management of papillary adenomas. It may also be considered for carcinoma without S2 invasion and neuroendocrine tumors. However, postpapillectomy bleeding (PPB) requiring reintervention is not uncommon. This study aimed to study several parameters to predict postpapillectomy bleeding.

**Materials and Methods:** Over a 15-year period, EP was performed on 132 patients. There were 68 males and 64 females. The median age was 46 (range: 32-76). For resection, Endocut Q current (ERBE, VIO 300 S electrosurgical unit with APC; Germany) was used.

**Results:** Bleeding requiring reintervention or blood transfusion occurred in 14 patients (10%). In another 7 patients, bleeding manifested as melena and a drop in hemoglobin; expectant management was sufficient (total 15.9%). Bleeding occurred with a median time of 12 hours (range: 1-42). Management by reintervention included clips, argon plasma coagulation (APC), Ankaferd spray, and metallic stent insertion into the common bile duct. None required surgery, and there were no deaths. These 21 patients were compared to 111 patients who did not bleed in reference to age, gender, coagulation parameters, use of antiplatelet or anticoagulant agents (which were stopped before a certain time of the procedure), size and histology of the resected papilla, and administration of APC after papillectomy; no significant differences were found. In 5 of the 21 patients (23%), bleeding occurred after 24 hours, and all had been discharged before bleeding manifested.

**Conclusion: **Bleeding after EP appears to be an unpredictable event. However, keeping patients in the hospital and withholding antiplatelet and anticoagulant agents for 48 hours after the procedure, if possible, may be safer.

## SS-08 Prevalence, Risk Factors, and Clinical Correlates of Malnutrition and Sarcopenia in Gastroenterology Inpatients: A Multicenter Cross-Sectional Study in Türkiye

**Göksel Bengi ^1^ , Süleyman Dolu ^1^ , Yavuz Özden ^2^ , Nevin Oruç ^3^ , Mukaddes Tozlu ^4^ , Gözde Derviş Hakim ^5^ , Genco Gençdal ^6^ , Ali Rıza Çalışkan ^7^ , Müge Ustaoğlu ^8^ , Ufuk Kutluana ^9^ , Engin Altıntaş ^10^ , Galip Egemen Atar ^11^ , Ahmet Uyanıkoğlu ^12^ , Sezgin Barutçu ^13^ , Kader Irak ^14^ , Deniz Koç ^15^ , Berat Ebik ^16^ , Züleyha Akkan Çetinkaya ^17^ , Tarık Kani ^18^ , Dilek Oğuz ^19^ , Filiz Araz ^20^ , Altay Kandemir ^21^ , Nermin Mutlu Bilgiç ^22^ , Özdal Ersoy ^23^ , Özlem Gül ^24^ , Banu Kara ^25^ , Burak Özşeker ^26^ , Hüseyin Alkım ^27^ , Sedat Boyacıoğlu ^28^**

^1^Department of Gastroenterology, Dokuz Eylül University Faculty of Medicine, İzmir, Türkiye

^2^Department of Gastroenterology, Kayseri City Hospital, Kayseri, Türkiye

^3^Department of Gastroenterology, Ege University Faculty of Medicine, İzmir, Türkiye

^4^Department of Gastroenterology, Sakarya Training and Research Hospital, Sakarya, Türkiye

^5^Department of Gastroenterology, University of Health Sciences İzmir Faculty of Medicine, İzmir City Hospital, İzmir, Türkiye

^6^Department of Gastroenterology, Koç University Faculty of Medicine, Ankara, Türkiye

^7^Department of Gastroenterology, Adıyaman Training and Research Hospital, Adıyaman, Türkiye

^8^Department of Gastroenterology, Ondokuz Mayıs University Faculty of Medicine, Samsun, Türkiye

^9^Department of Gastroenterology, Pamukkale University Faculty of Medicine, Denizli, Türkiye

^10^Department of Gastroenterology, Mersin University Faculty of Medicine, Mersin, Türkiye

^11^Department of Gastroenterology, Antalya Training and Research Hospital, Antalya, Türkiye

^12^Department of Gastroenterology, Şanlıurfa University Faculty of Medicine, Şanlıurfa, Türkiye

^13^Department of Gastroenterology, SANKO University Hospital, Gaziantep, Türkiye

^14^Department of Gastroenterology, Başakşehir Çam and Sakura City Hospital, İstanbul, Türkiye

^15^Department of Gastroenterology, İstanbul Gaziosmanpaşa Taksim Training and Research Hospital, İstanbul, Türkiye

^16^Department of Gastroenterology, Diyarbakır Gazi Yaşargil Training and Research Hospital, Diyarbakır, Türkiye

^17^Department of Gastroenterology, İstanbul Acıbadem Ataşehir Hospital, İstanbul, Türkiye

^18^Department of Gastroenterology, Marmara University Pendik Training and Research Hospital, İstanbul, Türkiye

^19^Department of Gastroenterology, Kırıkkale University Faculty of Medicine, Kırıkkale, Türkiye

^20^Department of Gastroenterology, Başkent University Adana Dr. Turgut Noyan Training and Research Center, Adana, Türkiye

^21^Department of Gastroenterology, Aydın Adnan Menderes University Faculty of Medicine, Aydın, Türkiye

^22^Department of Gastroenterology, Ümraniye Training and Research Hospital, İstanbul, Türkiye

^23^Department of Gastroenterology, İstanbul Acıbadem University Atakent Hospital, İstanbul, Türkiye

^24^Department of Gastroenterology, Lokman Hekim University Ankara Hospital, Ankara, Türkiye

^25^Gastroenterology Clinic, Adana City Training and Research Hospital, University of Health Sciences, Adana, Türkiye

^26^Division of Gastroenterology, Muğla Sıtkı Koçman University Faculty of Medicine, Muğla, Türkiye

^27^Gastroenterology Clinic, Şişli Hamidiye Etfal Training and Research Hospital, University of Health Sciences, İstanbul, Türkiye

^28^Department of Gastroenterology, Başkent University Faculty of Medicine, Ankara, Türkiye

**Background/Aims:** The aim of this study was to determine the prevalence of malnutrition and sarcopenia among patients hospitalized in gastroenterology clinics across different geographical regions of Türkiye, identify associated risk factors, and evaluate their relationship with clinical outcomes.

**Materials and Methods:** A total of 1051 patients admitted during the week of November 14, 2024, to 36 gastroenterology clinics in 6 geographical regions of Türkiye were evaluated in a cross-sectional design. Nutritional status was assessed using the Nutritional Risk Screening-2002 (NRS-2002), whereas sarcopenia risk was evaluated with the SARC-F questionnaire. Demographic data, clinical diagnoses, disease severity scores, and comorbidities were also recorded and analyzed.

**Results:** Of the included patients, 54.7% were female, with a mean age of 61.7 ± 17.2 years. The prevalence of malnutrition was 27.8%, whereas the risk of sarcopenia was 32.7%. Patients with malnutrition had significantly lower BMI (24.7 ± 5.3 vs. 27.1 ± 5.4, *P* < .001) and were older (67.6 ± 16.0 vs. 56.5 ± 17.1, *P* < .001). The risks of malnutrition and sarcopenia were particularly increased in patients with liver cirrhosis (40.7% malnutrition; 54.5% sarcopenia), gastrointestinal malignancies (50.5%; 44.2%), and diabetes mellitus. Logistic regression analysis identified advanced age, male sex, and malignancy as independent risk factors for malnutrition, whereas advanced age, female sex, presence of malnutrition, liver cirrhosis, and heart failure were independent risk factors for sarcopenia. A strong correlation was also observed between malnutrition and sarcopenia (*r* = 0.544, *P* < .001).

**Conclusion:** Approximately one-third of patients hospitalized in gastroenterology clinics across Türkiye are at risk of malnutrition and sarcopenia. These conditions are particularly associated with malignancy, cirrhosis, and metabolic comorbidities. These findings highlight the necessity of systematic screening for malnutrition and sarcopenia during hospital admissions.

## SS-09 Changes in the Gut Microbiota in Cholelithiasis: Identification of Discriminatory Bacterial Genera Based on Machine Learning

**Tayyip Karaman ^1^ , Suna Yapalı ^2^ , Cem Aygün ^3^ , Melih Kara ^4^ , Hakan Yardımcı ^4^ , Ali Arslan ^5^ , Uğur Sezerman ^1^ , Nurdan Tözün ^2^**

^1^Department of Biostatistics and Medical Informatics, Acıbadem Mehmet Ali Aydınlar University, İstanbul, Türkiye

^2^Department of Gastroenterology, Acıbadem Mehmet Ali Aydınlar University, İstanbul, Türkiye

^3^Department of Gastroenterology, Acıbadem Dr. Şinasi Can Hospital, İstanbul, Türkiye

^4^Department of General Surgery, Acıbadem Dr. Şinasi Can Hospital, İstanbul, Türkiye

^5^Department of Molecular Biology and Genetic, Yıldız Technical University, İstanbul, Türkiye

**Background/Aims**: Gallstone disease (cholelithiasis) results from the aggregation of bile components, primarily cholesterol, bile pigments, and calcium salts, within the gallbladder or biliary tract. Its development is influenced by multiple risk factors, including obesity, high-fat diets, rapid weight loss, pregnancy, and aging. Emerging evidence suggests that gut and biliary microbiota may contribute to gallstone formation by promoting crystallization through bacterial lipopolysaccharides, facilitating biofilm-mediated cholesterol and calcium entrapment, or inducing mucin overproduction and gallbladder hypomotility.

**Materials and Methods:** Current research on the relationship between cholelithiasis and the microbiome is limited and often lacks functional pathway interpretation. Therefore, this study compared the fecal microbiota of cholelithiasis patients and healthy individuals to identify discriminatory taxa and explore their potential roles in gallstone pathogenesis.

A total of 47 fecal samples were collected, including 27 from patients with gallstones and 20 from healthy controls. Long-read 16S rRNA sequencing was performed to characterize the gut microbiota composition. LEfSe and Random Forest classification models were used to detect discriminatory taxa associated with either the healthy or patient groups.

**Results and Conclusion:** Through machine learning analysis, discriminatory taxa were identified by excluding nonrelevant features. Six genera—*Waltera, Vescimonas, Eubacterium, Phocaeicola, Mediterraneibacter, and Anaerobutyricum*—were found to differentiate patients from controls. Differential abundance analyses further revealed increased levels of *Roseburia, Eubacterium, Phocaeicola, Dorea, Coprococcus*, and particularly *Enterococcus faecium*, indicating a dysbiotic environment that favors opportunistic species rather than direct causation. Although causality remains to be established, the consistent enrichment of these taxa, alongside increased microbial richness and the proliferation of opportunistic pathogens such as *Enterococcus faecium*, highlights the presence of a dysbiotic gut environment in cholelithiasis. Although these genera cannot yet be declared definitive biomarkers, they represent promising microbial candidates for future mechanistic investigations and potential diagnostic development following experimental validation.


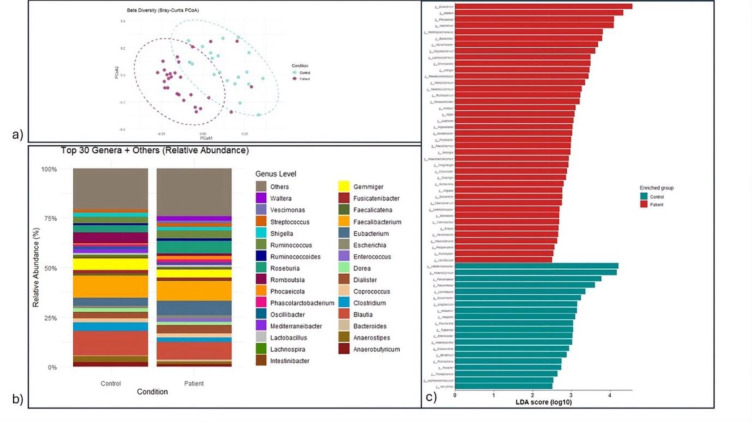


Figure 1.Microbiome beta diversity analyses, abundance variations, and LEfSe results between healthy and patient groups. a) Beta diversity analysis based on 16S rRNA microbiome sequencing revealed statistically significant clustering between patients with gallstone and the healthy control group, indicating distinct taxonomic separation between the 2 groups. b) Microbial abundances between the groups at the genus level are illustrated using bar charts. c) LEfSe analysis identified the key genera that most strongly differentiate the patient group from the healthy group.


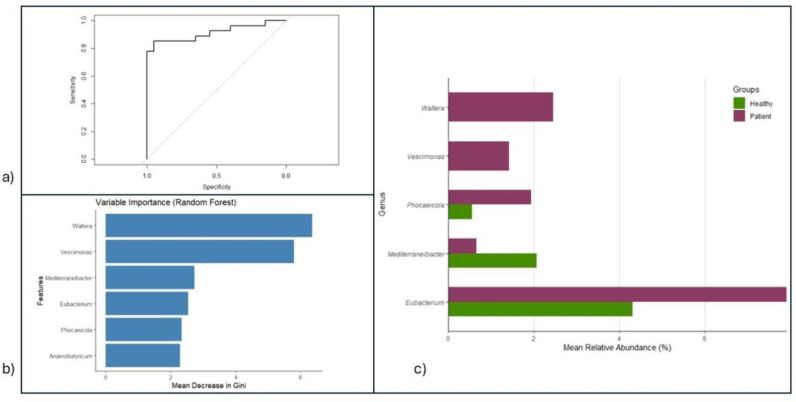


Figure 2.Random forest machine learning results. a) The ROC curve obtained from the Random Forest machine learning method demonstrates the sensitivity and specificity of the model. b) During model construction, statistically significant genera that contributed to group differentiation were identified. c) The abundances of the genera involved in the model were visualized for both groups using bar plots.

## SS-11 Relationship of Dietary Habits with Functional Dyspepsia and Irritable Bowel Syndrome Subtypes: A Cross-sectional Medical Student-based Preliminary Study

**Halit KANDEMIR ^1^ , Dilek ÇEKİM KANDEMİR ^2^ , Kenan MORAL ^1^ , Beril DEMİR ^1^ , Enes CÖMERT ^1^ , Derya KİRMAN ^1^ , Ali KARATAŞ ^1^ , Mehmet CİNDORUK ^1^ , Tarkan KARAKAN ^1^**

^1^Division of Gastroenterology, Department of Internal Medicine, Gazi University Faculty of Medicine, Ankara, Türkiye

^2^Department of Nutrition and Dietetics, Gaziler Physical Therapy and Rehabilitation Research and Training Hospital, Ankara, Türkiye

**Background/Aims: **Functional gastrointestinal disorders (FGIDs), including functional dyspepsia (FD) and irritable bowel syndrome (IBS), are common among young adults and are influenced by both dietary and psychosocial factors. Although their prevalence is well documented, the dietary associations remain unclear, especially among medical students who experience significant academic stress. This study aimed to examine the relationship between dietary habits and the presence of FD and IBS subtypes in Turkish medical students.

**Materials and Methods: **In this cross-sectional study, FD and IBS subtypes were identified using a structured questionnaire based on Rome IV criteria. Participants also answered detailed questions about meal patterns and dietary habits to explore their association with FGIDs.

**Results: **Among 636 students, the prevalence of FD was 34%, and IBS was 13%. Both disorders were significantly more common in females. IBS was notably more frequent in students in clinical training years. FD was associated with frequent consumption of processed foods, whereas IBS was linked to the intake of carbonated beverages. Diarrhea-predominant IBS was more common in males and those consuming high amounts of leafy greens. No significant differences were found between groups regarding fast food, dairy, protein-rich, or grain-based foods.

**Conclusion: **FGIDs in medical students may be associated with specific dietary patterns, particularly processed foods and carbonated beverages. Given the high academic stress in this population, evaluating and modifying dietary habits may help identify personal triggers, potentially improving gastrointestinal symptoms and overall well-being.


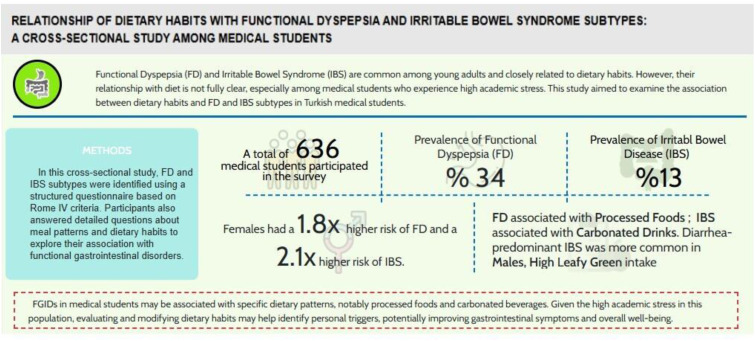


Figure 1.Summary of the study.


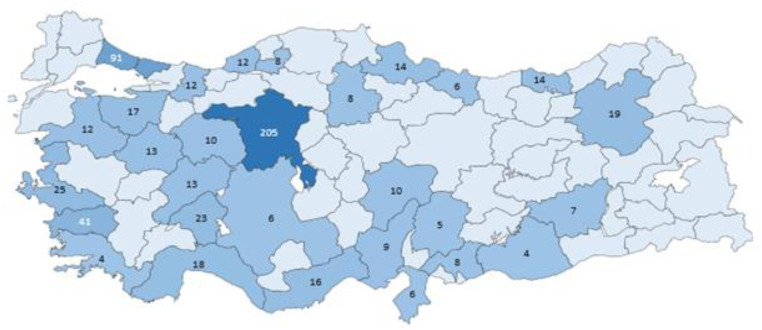


Figure 2.Number of survey participants by city in Türkiye.

## SS-13 Proteomic Analysis of OipA-Associated Responses in H. pylori-Infected Human Gastric Organoids

**Sümeyye Akçelik Deveci ^1^^,^^2^ , Ali Durmuş ^3^ , Sinem Öktem Okullu ^4^**

^1^Department of Medical Biotechnology, Acıbadem Mehmet Ali Aydınlar University Institute of Health and Science, İstanbul, Türkiye

^2^Department of Molecular Biology and Genetics, Boğaziçi University Faculty of Sciences, İstanbul, Türkiye

^3^Department of General Surgery, Private Avrasya Hospital, İstanbul, Türkiye

^4^Department of Medical Microbiology, Acıbadem Mehmet Ali Aydinlar University School of Medicine, İstanbul, Türkiye

**Background/Aims: ***Helicobacter pylori* (*H. pylori*) is a key pathogen in the development of gastric diseases. One of its outer membrane proteins, OipA, facilitates bacterial adhesion to the gastric mucosa and enhances infection severity. However, the molecular effects of OipA on host cells remain poorly understood. This study aimed to investigate the proteomic alterations driven by OipA during *H. pylori* infection in a human gastric organoid model.

**Materials and Methods: **Human gastric organoids were infected with *H. pylori* G27 (WT) and G27-ΔoipA strains. Proteins isolated from infected and control cells were analyzed by LC-MS/MS. Data were normalized using Thermo Proteome Discoverer, and differential expression was calculated via label-free quantification (LFQ). Proteins were considered significant with ≥2 unique peptides and a fold change ≥1.3. OipA-specific effects were determined by comparing uninfected controls and ΔoipA-infected groups. Protein-protein interaction (PPI) networks were generated using the STRING database and visualized in Cytoscape (3.8.2), with pathway enrichment performed via MCODE.

**Results:** Out of 3650 identified proteins, 1920 with ≥2 unique peptides were quantified. LFQ analysis revealed significant expression changes in 227 proteins during OipA-positive infection (152 upregulated, 75 downregulated). In contrast, the ΔoipA group exhibited 226 altered proteins (138 upregulated, 88 downregulated). PPI mapping highlighted OipA-dependent alterations in key pathways, including rRNA processing, apoptosis regulation, and amino acid metabolism. These findings indicate that OipA modulates over 10% of the host proteome during infection, reprogramming cell death and metabolic pathways.

**Conclusion:** This study demonstrates that OipA directly shapes host proteomic responses in gastric organoids, impacting essential pathways beyond adhesion. The results support OipA as not only a colonization factor but also a critical regulator of infection severity, apoptosis, and metabolic reprogramming. Collectively, these findings position OipA as a potential molecular target in the pathogenesis of *H. pylori*-associated gastric diseases.

## SS-14 Older Patients with Metabolic Dysfunction-Associated Steatotic Liver Disease (MASLD) Have Higher Rates of Lean Metabolic Dysfunction-Associated Steatotic Liver Disease and More Advanced Fibrosis but Better Health-Related Quality of Life (HRQL)

**Yusuf Yılmaz ^1^ , Ming Lung Yu ^2^ , Mohamed El Kassas ^3^ , Vasily Isakov ^4^ , Marlen Castellanos Fernandez ^5^ , Vincent Wai Sun Wong ^6^ , Yuichiro Eguchi ^7^ , Manuel Romero Gomez ^8^ , Ajay Duseja ^9^ , Elisabetta Bugianesi ^10^ , Wah Kheong Chan ^11^ , Khalid Alswat ^12^ , Saeed Hamid ^13^ , Ashwani K. Singal ^14^ , Jian Gao Fan ^15^ , Georgios Papatheodoridis ^16^ , Stuart C. Gordon ^17^ , Ziad H. Younes ^18^ , Stuart K. Roberts ^19^ , Nahum Méndez Sánchez ^20^ , Çağlayan Keklikkıran ^1^ , Brian Lam ^21^ , Fatema Nader ^21^ , Linda Henry ^21^ , Maria Stepanova ^21^ , Saleh Alqahtani ^22^ , Zobair M. Younossi ^21^**

^1^Department of Gastroenterology, Recep Tayyip Erdoğan University School of Medicine, Rize, Türkiye

^2^Department of Internal Medicine, Kaohsiung Medical University Faculty of Medicine, Kaohsiung, Taiwan

^3^Department of Endemic Medicine, Helwan University Faculty of Medicine, Cairo, Egypt

^4^Department of Gastroenterology and Hepatology, Federal Research Center for Nutrition, Biotechnology and Food Safety, Moscow, Russia

^5^University of Medical Sciences of Havana, Institute of Gastroenterology, Havana, Cuba

^6^Department of Medicine and Therapeutics, The Chinese University of Hong Kong, Medical Data Analytics Center, Hong Kong, China

^7^Loco Medical General Institute, Saga, Japan

^8^Digestive Diseases Unit, Virgen Del Rocío University Hospital, University of Seville, Seville, Spain

^9^Department of Hepatology, Post Graduate Institute of Medical Education and Research, Chandigarh, India

^10^Division of Gastroenterology, Department of Medical Sciences, A.O.U. Città della Salute e della Scienza di Torino, University of Turin, Turin, Italy

^11^Department of Medicine, University of Malaya Faculty of Medicine, Gastroenterology and Hepatology Unit, Kuala Lumpur, Malaysia

^12^Department of Medicine, King Saud University College of Medicine, Liver Disease Research Center, Riyadh, Saudi Arabia

^13^Department of Medicine, Aga Khan University Hospital, Karachi, Pakistan

^14^Department of Gastroenterology, Hepatology and Nutrition, University of Louisville School of Medicine and University of Louisville Alcohol Research Center, Kentucky, USA

^15^Department of Gastroenterology, Xinhua Hospital, Shanghai Jiaotong University School of Medicine, Shanghai, China

^16^Department of Gastroenterology, National and Kapodistrian University of Athens, Athens, Greece

^17^Henry Ford Health and Wayne State University School of Medicine, Detroit, USA

^18^Gastro One, Tennessee, USA

^19^Alfred Hospital and Monash University, Melbourne, Australia

^20^Liver Research Unit, Medica Sur Clinic and Foundation, National Autonomous University of Mexico Faculty of Medicine, Mexico City, Mexico

^21^Beatty Liver and Obesity Research Program, Inova Health System, Virginia, USA

^22^Department of Medicine, King Faisal Specialist Hospital and Research Center Gastroenterology Section, Riyadh, Saudi Arabia

**Background/Aims: **The impact of age on metabolic dysfunction-associated steatotic liver disease (MASLD) outcomes has not been fully assessed. The aim was to evaluate the clinical and patient-reported outcomes (PROs) of patients with MASLD based on age groups.

**Materials and Methods: **Patients with MASLD were prospectively enrolled in the Global NAFLD/MASLD Registry (GNR). Clinical and PRO data (FACIT-F, CLDQ-MASH, and WPAI) were analyzed in patients with MASLD aged <65 years, 65-74 years, and ≥75 years.

**Results: **A total of 8441 patients with MASLD from 18 countries were included, with a mean (SD) age of 52 (13) years, 46% male, 65% obese, 38% with type 2 diabetes (T2D), 49% with hypertension, 47% with hyperlipidemia, 14% with advanced fibrosis, 19% with depression, 39% experiencing clinically overt fatigue, and 24% with sleep apnea. Of these, 83% were <65 years, 14.5% were 65-74 years, and 2.3% were ≥75 years old. The proportion of lean MASLD increased from 5% in the <65 group to 9% in the 65-74 group and 17% in the ≥75 group, whereas the prevalence of T2D was 35% in <65 compared to 50% in 65-74 and 49% in ≥75; the rates of hypertension and hyperlipidemia also rose with age (all *P* < .0001). In patients aged ≥65 years, only T2D was a predictor of advanced fibrosis (OR = 2.56 (1.94-3.38), *P* < .0001). The health-related quality-of-life scores from CLDQ-MASH and FACIT-F were higher in older patients with MASLD. In patients aged ≥65 years, independent predictors of lower PRO scores included female sex, the presence of obesity and T2D, a history of psychiatric comorbidities, clinically overt fatigue, abdominal pain, sleep apnea, and lack of regular exercise (*P* < .05).

**Conclusion:** Older patients with MASLD have a higher rate of lean MASLD and more advanced fibrosis but report higher PRO scores.


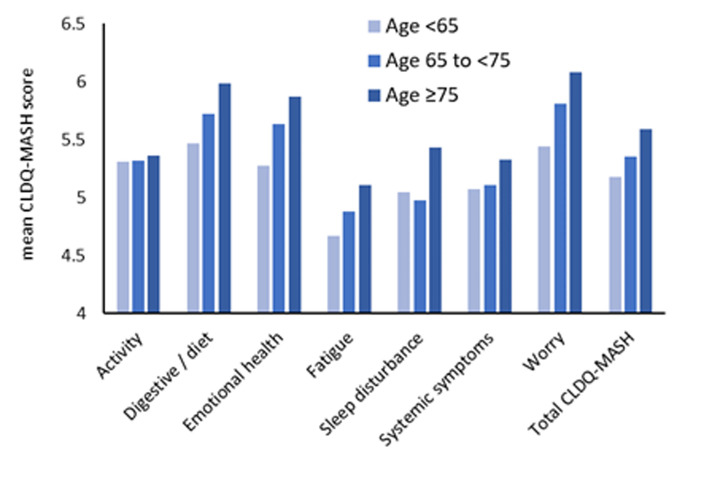


Figure 1. Evaluation of CLDQ-MASH and FACIT-F scores by age groups in older patients with MASLD.

## SS-15 EUS-Guided Treatment in Patients with Gallstone Disease Who Are Unsuitable for ERCP, Have Failed ERCP, or Cannot Undergo Cholecystectomy: A Retrospective Cohort with Long-term Follow-up

**Hakan Şentürk ^1^ , İbrahim Hakkı Köker ^2^ , Erkan Çağlar ^3^ , Sercan Kiremitçi ^1^ , Şerife Değirmencioğlu Tosun ^4^ , Ali Tüzün İnce ^1^**

^1^Department of Gastroenterology, Bezmialem Foundation University Faculty of Medicine, İstanbul, Türkiye

^2^Department of Gastroenterology, Başkent University Faculty of Medicine, İstanbul, Türkiye

^3^Department of Gastroenterology, Sarıkız Gastroenterology Center, Balıkesir, Türkiye

^4^Department of Gastroenterology, University of Health Sciences Bağcılar Training and Research Hospital, İstanbul, Türkiye

**Background/Aims:** Treatment of gallstone disease involves cholecystectomy for cholelithiasis and ERCP for choledocholithiasis. However, cholecystectomy may be risky for elderly patients and those with significant comorbidities. In surgically altered patients, ERCP may not be possible for choledocholithiasis, and in patients with preserved gastrointestinal anatomy, ERCP may also fail. The long-term outcomes of EUS-guided treatment performed between 2011 and 2022 in a group of such patients were evaluated.

**Materials and Methods:** The study included 23 patients. In the bile duct group, there were 16 patients consisting of 13 males and 3 females. In the gallbladder group,6 were female and 1 was male. The mean age was 62 ± 18.6 (range: 28-86) in the bile duct group and 73 ± 11.7 (51-83) in the gallbladder group. In the bile duct group, 1 patient had a hepaticojejunostomy without tract surgery, 12 had gastroenterostomy (including 1 with Billroth II and 9 with Roux-en-Y anastomosis), and 1 had a total gastrectomy with esophagojejunostomy. In 2 patients, the anatomy was physiological, but ERCP had failed. All patients in the gallbladder group had recurrent cholecystitis and were at high surgical risk. Under EUS guidance, 13 patients underwent hepaticogastrostomy, 1 underwent hepatoesophagostomy, 2 underwent choledochoduodenostomy, 5 underwent cholecystogastrostomy, and 2 underwent cholecystoduodenostomy. In the bile duct patients with normal anatomy, choledochoduodenostomy was preferred. After fistula formation, the stones were primarily treated with antegrade laser lithotripsy under cholangioscopic guidance.

**Results:** Technical success was achieved in all patients. However, 7 of those with bile duct stones continued to experience episodes of cholangitis. In all of these patients, the diameter of the common bile duct or main hepatic duct was greater than 20 mm. In the other 6 patients, the duct diameter was 15 mm or less, and no further episodes of cholangitis occurred. In patients who underwent the procedure for gallbladder stones, clinical success was 100%.

**Conclusion:** EUS-guided treatment is a safe and highly effective option for managing bile duct stones when ERCP is not feasible and for gallbladder stones in patients at high risk for cholecystectomy.

## SS-17 Wire-Guided Over-the-Scope Clip Method: Improves Success in Challenging Cases

**Kübra Köken, Bülent Ödemiş, Alper Macif, Nazmi Gökhan Ünver, Kerem Kenarlı, Göktürk Karataş, Ahmet Burak Fedai, Erdoğan Deniz, Muhammet Emin Oktay**

Department of Gastroenterology, Ankara Bilkent City Hospital, Ankara, Türkiye

**Background/Aims: **The over-the-scope clip (OTSC) system is an endoscopic clip used for managing gastrointestinal perforations, fistulas, anastomotic leaks, and massive bleeding. This study aimed to evaluate the success of wire-guided OTSC placement in challenging cases.

**Materials and Methods:** Patients who underwent the wire-guided OTSC technique in the clinic from 2014 to 2025 were evaluated. The lesion was identified using endoscopy, and a balloon catheter was guided to the area for assessment. In appropriate cases, the wire was retained in the lesion cavity or withdrawn from the opposite end of the cavity. The endoscope was then removed and re-inserted into the lesion area over the guidewire after the OTSC system was loaded. The defect was closed by deploying the clip with the wire centered. Technical success was defined as accurate clip placement, and clinical success as the resolution of the underlying condition according to the indication.

**Results:** Thirty-seven patients participated in the study, including 21 females (56.8%), with a mean age of 53.3 ± 19.9 years. Indications for OTSC placement included fistula (n = 15, 40.5%), perforation (n = 8, 21.6%), sleeve gastrectomy leak (n = 8, 21.6%), anastomotic leak (n = 2, 5.4%), and tracheoesophageal fistula (TEF) (n = 4, 10.8%). Among the 15 fistula cases, excluding sleeve gastrectomy leak and TEF, lesion sites were in the duodenum (n = 4), stomach (n = 4), esophagus (n = 2), rectum (n = 3), and surgically altered ileum accessible endoscopically (n = 2). Technical success was achieved in 35 patients (94.6%) with appropriate clip placement. OTSC was applied as a standalone technique in 24 patients (64.9%), whereas additional endoscopic interventions were combined in 13 patients (35.1%). One patient required surgery due to technical failure, and 2 were referred to surgery despite technical success due to a lack of clinical improvement. The long-term durable clinical success rate was 75.7%. No major complications were observed during the procedures.

**Conclusion:** The wire-guided OTSC method facilitates access to challenging sites, allows precise clip positioning near the luminal wall, reduces device mobility, and enables safe aspiration without damaging fibrotic tissue.

## SS-18 The Significance of Calprotectin Levels in the Differential Diagnosis of Ascitic Fluids

**İbrahim Bayhan ^1^ , Osman Yüksekyayla ^2^ , Ahmet Uyanıkoğlu ^2^**

^1^Department of Internal Medicine, Batman Training and Research Hospital, Batman, Türkiye

^2^Department of Gastroenterology, Harran University Faculty of Medicine, Şanlıurfa, Türkiye

**Background/Aims: **Determining the etiology of ascites is a common diagnostic challenge in clinical practice. One of the most widely used methods for this purpose is the serum-ascites albumin gradient (SAAG). In diagnostic testing, methods with high sensitivity and specificity, ease of application, and low cost are preferred. The aim of this study was to evaluate the diagnostic value of calprotectin levels in distinguishing between malignant and nonmalignant ascitic fluids.

**Materials and Methods:** A total of 88 patients with ascites were included in the study. Based on clinical, laboratory, and imaging findings, patients were divided into 2 groups: malignant ascites and nonmalignant ascites. Ascitic fluid samples were obtained from all cases, and calprotectin levels were measured. In addition, the SAAG was calculated, abdominal ultrasonography was performed, and oxidative stress parameters in ascitic fluid were analyzed for each patient.

**Results:** Ascitic fluid calprotectin levels were found to be significantly higher in the malignant group. Using a cutoff value of 20.68 ng/mL for calprotectin, a sensitivity of 92.8% and a specificity of 93% were achieved in distinguishing malignant ascitic fluid. In contrast, oxidative stress parameters assessed in ascitic fluid demonstrated lower diagnostic performance.

**Conclusion: **Ascitic fluid calprotectin levels show high diagnostic accuracy in differentiating malignant from nonmalignant ascites. Therefore, calprotectin may serve as an effective biomarker in clinical practice.


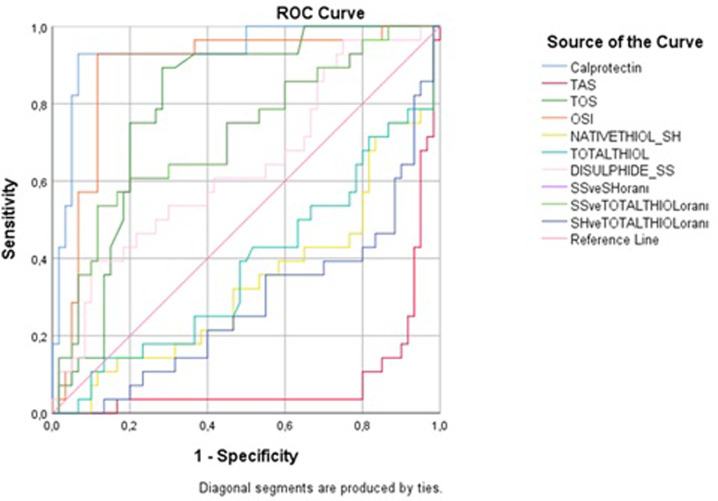


Figure 1. ROC analysis in patients with malignant ascites.

## SS-19 Monitoring the Effectiveness of Prophylactic Treatment in Portal Hypertension-Associated Esophageal Variceal Bleeding Using Transient Elastography-Mediated Liver and Spleen Stiffness Measurements

**İbrahim Bayhan ^1^ , Osman Yüksekyayla ^2^ , Ahmet Uyanıkoğlu ^2^**

^1^Department of Internal Medicine, Batman Training and Research Hospital, Batman, Türkiye

^2^Department of Gastroenterology, Marmara University, İstanbul, Türkiye

**Background/Aims:** Portal hypertension (PHT), defined as an increased portal-caval pressure gradient, is the main cause of esophageal variceal bleeding. Although the hepatic venous pressure gradient (HVPG) is the gold standard for diagnosis, its invasiveness limits its use. Noninvasive methods such as vibration-controlled transient elastography (VCTE), including liver stiffness (LSM) and spleen stiffness (SSM), have gained importance. This study aimed to evaluate the impact of beta-blockers (BB) and endoscopic band ligation (EBL) on LSM and SSM as indicators of portal pressure.

**Materials and Methods:** Patients undergoing endoscopy for variceal screening between October 2023 and November 2024 were prospectively enrolled. Simultaneous VCTE measurements were performed on the day of endoscopy. Patients were divided into 2 groups: BB and BB + EBL. LSM, SSM, and platelet counts (PLT) were recorded. According to Baveno criteria, LSM >20 kPa with PLT <150 000/mm^3^ and SSM >40 kPa were considered determinants of high-risk varices (grades 2 and 3). Follow-up VCTE was performed within 6-12 weeks.

**Results:** Thirty patients were included (18 BB, 12 BB + EBL). The Baveno criteria predicted high-risk varices with 100% accuracy. In detecting high-risk varices, LSM showed 72.7% accuracy, SSM 86.4%, and PLT 95.5%. LSM and SSM values were similar between the groups. At follow-up, SSM decreased by 5% in the BB group, whereas it increased by 34% in the BB+EBL group (*P* = .034). LSM increased similarly in both groups.

**Conclusion:** The Baveno criteria demonstrate high accuracy in predicting high-risk esophageal varices. In this study, a significant increase in SSM levels was observed after prophylaxis in the EBL group, whereas a decrease was noted in the BB-only group. These findings suggest that EBL may increase portal pressure and that SSM can serve as a valuable parameter for dynamic monitoring. Confirmation of these results requires multicenter, randomized studies with HVPG control.


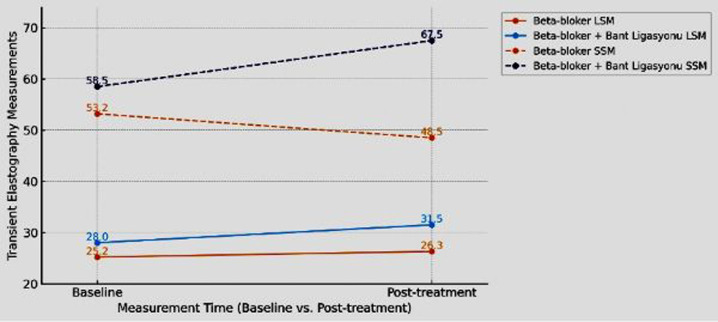


Figure 1. Change in SSM and LSM after prophylaxis by group. The Y-axis shows SSM and LSM measurements, and the X-axis shows measurement time.

## SS-20 Comparative Analysis of Endoscopic Ultrasound and Positron Emission Tomography-Computed Tomography Findings in the Diagnosis and Characterization of Pancreatic Masses

**Pelin Telli, Bilger Çavuş, Aslı Çifcibaşı Örmeci, Sezen Genç Uluçeçen, Gizem Dağcı, Mehmet Akif Yağlı, Asım Gurbanov, Besim Fazıl Ağargün, Sabuhi Mammadov, Ersel Bilgin, Kadir Demir, Fatih Beşışık, Sabahattin Kaymakoğlu, Filiz Akyüz**

Department of Gastroenterology and Hepatology, İstanbul University Faculty of Medicine, İstanbul, Türkiye

**Background/Aims: **The diagnosis of pancreatic masses presents a significant clinical challenge due to their malignant potential. Endoscopic ultrasound (EUS) and positron emission tomography-computed tomography (PET-CT) are critical evaluation modalities. This study aimed to evaluate and compare EUS and PET-CT findings in a cohort of patients with pancreatic masses.

**Materials and Methods:** Data from 94 consecutive patients evaluated with both EUS and PET-CT between 2020 and 2025 were retrospectively analyzed.

**Results:** Histopathological examination revealed 84 malignant (89.4%) and 10 benign (10.6%) masses. EUS demonstrated a smaller median mass size in benign lesions (20 mm vs. 30 mm; *P* = .005) and showed vascular invasion in 60.7% of malignant cases. PET-CT detected a lower median SUV-max in benign lesions (6 vs. 8; *P* = .023). Median CA 19-9 levels were higher in the malignant group (216 U/mL vs. 45 U/mL; *P* = .039). The highest diagnostic accuracy was associated with the EUS endosonographic impression, based on ROC analysis. The AUCs (95% CI, *P*) were: EUS impression, 0.744 (95% CI: 0.583-0.905, *P* = .002); EUS size, 0.701 (*P* = .011); EUS vascular invasion, 0.647 (*P* = .063); PET-CT qualitative assessment, 0.601 (*P* = .201); and PET-CT SUV-max, 0.568 (*P* = .399). Kaplan–Meier analysis showed that the median overall survival for patients with a benign EUS impression (49 months) was significantly longer than for those with a malignant impression (16 months) (log-rank *P* = .001). EUS-detected vascular invasion was associated with shorter median overall survival (6 months vs. 24 months in its absence) (log-rank *P* = .001).

**Conclusion:** These findings highlight the complementary roles of EUS and PET-CT. Integrating clinical information, imaging findings, and biomarker data is essential for optimal diagnosis and management.


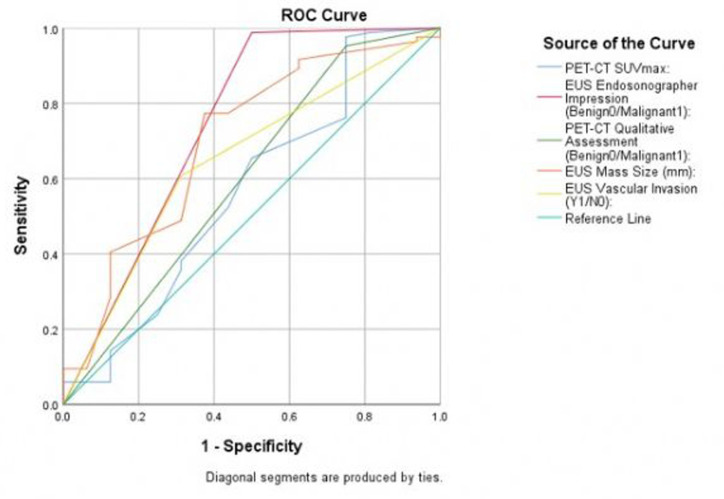


Figure 1. The highest diagnostic accuracy was associated with the EUS endosonographic impression, based on ROC analysis.

## SS-23 Endoscopic Ultrasound-Guided Lumen-Apposing Metal Stent Insertion: A Single-Center Experience

**Orhan Cem Deniz, Gürhan Şişman**

Department of Gastroenterology, Acıbadem Mehmet Ali Aydınlar Faculty of Medicine, İstanbul, Türkiye

**Background/Aims:** The study aimed to evaluate the outcomes of patients treated with lumen-apposing metal stent (LAMS) for various indications at the center.

**Materials and Methods:** The medical records of 36 patients who were treated with LAMS insertion at the center between May 2023 and September 2025 were retrospectively analyzed. Demographic data of the patients (age and gender), procedural indications and techniques, technical and clinical success rates, and follow-up durations were recorded and evaluated.

**Results:** Of the cases, 63.9% were male (n = 23) and 36.1% were female (n = 13). The mean age was 65.9 ± 16.5 years (range: 30-94 years). Biliary drainage for malignancy-related strictures was performed in 22.2% of cases (n = 8), gallbladder drainage in 47.2% (n = 17), and pancreatic fluid collection drainage in 30.6% (n = 11). Among patients with malignant obstruction, 50% (n = 4) underwent choledochoduodenostomy and 50% (n = 4) underwent gastroenterostomy. Among patients who underwent gallbladder drainage, 70.6% (n = 12) had cholecystoduodenostomy, whereas 29.4% (n = 5) had cholecystogastrostomy. In patients with pancreatic fluid collections, necrosectomy was performed in 36.4% (n = 4) and pseudocyst drainage in 63.6% (n = 7). The overall technical success rate was 94.4%, and the clinical success rate was 97.2%. Complications included bleeding in 2.8% of cases (n = 1) and stent migration in 2.8% (n = 1). The mean follow-up duration was 250.6 ± 253.8 days (range: 5-865 days).

**Conclusion:** Endoscopic ultrasound-guided LAMS insertion is a safe and effective procedure with high technical and clinical success rates when performed for appropriate indications. These findings demonstrate that, when applied in experienced centers, LAMS is associated with low complication rates and high clinical efficacy.

## SS-25 Endoscopic Necrosectomy: Emerging Standard of Care for Infected Necrosis in Acute Pancreatitis

**Hakan Şentürk ^1^ , Sercan Kiremitçi ^1^ , Elmas Biberci Keskin ^2^ , Şerife Değirmencioğlu Tosun ^3^ , Temel Fatih Yılmaz ^4^ , Ali Tüzün İnce ^1^**

^1^Department of Gastroenterology, Bezmialem Foundation University Faculty of Medicine, İstanbul, Türkiye

^2^Department of Internal Medicine, University of Health Sciences, Şişli Hamidiye Etfal Training and Research Hospital, İstanbul, Türkiye

^3^Department of Gastroenterology, University of Health Sciences, Bağcılar Training and Research Hospital, İstanbul, Türkiye

^4^Department of Radiology, Bezmialem Foundation University Faculty of Medicine, İstanbul, Türkiye

**Background/Aims:** Infected necrosis as a complication of acute necrotizing pancreatitis (ANP) is a serious condition with high mortality. In the past, surgical necrosectomy was the standard of care; however, its morbidity and mortality rates were very high. Recently, endoscopic necrosectomy (EN) has emerged as the potential standard of care in specialized centers. The results were reviewed with EN in ANP.

**Materials and Methods:** The data of 32 patients who underwent endoscopic necrosectomy were retrospectively studied. There were 19 males and 13 females, with a mean age of 46 ± 16 (range: 24-86). The leading etiologies were primarily biliary, hypertriglyceridemia, alcohol, or idiopathic, in order of frequency.

**Results:** Initially, either LAMS (20 × 16 mm) or FC-SEMS (40/60-20 mm) was introduced. After 1 week, a repeat EN was performed. In urgent cases, necrosectomy was proceeded to directly and immediately following stent insertion. It was performed weekly until a satisfactory response was achieved or the patient expired. The median number of sessions was 3 (range, 2-5), and the median session duration was 42 minutes (range, 26-98). ERCP was performed with pancreatic stenting in patients with disconnected duct syndrome or jaundice, and 2 EUS-guided cholecystogastrostomy/duodenostomy procedures were conducted due to acute cholecystitis. Percutaneous drainage was also performed in 17 of the 32 patients. Three patients underwent surgery (2 of whom expired). Nine out of 32 patients (28%) expired due to multiorgan failure. When the patients were divided into 2 halves (16/16), the number of patients who expired was 6 in the first half, whereas it was 3 in the second half. This difference is likely due not only to the increased success of necrosectomy but also to the overall improvement in multidisciplinary care. All surgery cases were in the first half.

**Conclusion:** EN for infected necrosis of the pancreas appears to be a promising approach in tertiary care centers concerning the prognosis of acute pancreatitis.

## SS-26 Endoscopic Cyst Evacuation in the Management of Hepatic Hydatid Disease with Biliary Communication: A Paradigm Shift

**Nazmi Gökhan Ünver, Bülent Ödemiş, Alper Macif, Kerem Kenarlı, Göktürk Karataş, Kübra Köken, Ahmet Burak Fedai, Erdoğan Deniz, Muhammed Emin Oktay**

Department of Gastroenterology, Ankara Bilkent City Hospital, Ankara, Türkiye

**Background/Aims:** In hepatic hydatid disease with biliary communication, ERCP traditionally aims to clear hydatid material from the bile ducts and close the cystobiliary fistula with stenting. The efficacy and safety of complete endoscopic evacuation of cyst contents as a curative treatment was evaluated in selected patients, with tract dilation when necessary.

**Materials and Methods:** This retrospective, single-center study included consecutive patients who underwent endoscopic cyst evacuation by a single endoscopist. The procedure was performed on centrally located cysts completely surrounded by liver parenchyma, without exophytic extension, and whose cavities can be endoscopically cannulated. When the fistulous tract was unsuitable for evacuation, it was dilated using a 6- to 8-mm balloon. The cyst cavity was irrigated with saline and debrided in sessions using stone extraction balloons and Dormia baskets. At the end of each session, a plastic or fully covered metal stent, or a nasocystic drain, was placed into the cavity. Clinical success was defined as endoscopic clearance and reduction in cyst size.

**Results:** Of 102 patients who underwent ERCP for biliary hydatid disease, 37 consecutive patients received endoscopic cyst evacuation. The mean age was 46.8 ± 19.1 years, and the mean cyst diameter was 82.9 ± 36.1 mm. A total of 184 ERCPs (median: 5) were performed. Sphincterotomy was performed in all cases. Balloon dilation of the cystobiliary opening was carried out in 19 patients (51.4%), nasocystic drainage in 12 (32.4%), and fully covered metal stents in 4 (10.8%) due to dense contents. Clinical success was achieved in 36 patients (97.3%). One patient required surgery for multiple hepatic abscesses. No recurrence or mortality occurred. Nine procedures (4.8%) had complications, all of which were successfully managed endoscopically or conservatively.

**Conclusion:** Endoscopic evacuation offers a safe and effective curative option for hepatic hydatid disease with biliary communication, with high success and low complication rates.


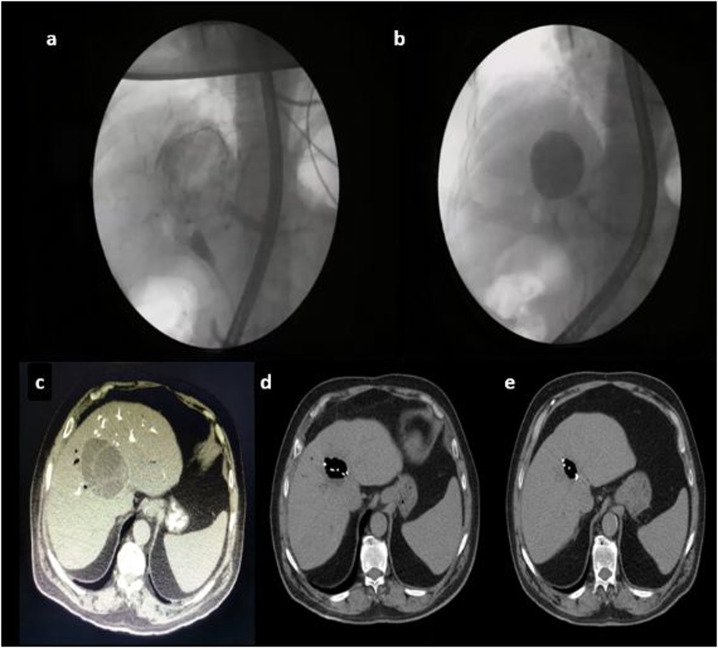


Figure 1. Fluoroscopic and CT images before and after endoscopic evacuation of a hepatic hydatid cyst with biliary communication. (a) Cyst cavity filled with hydatid material. (b) Cyst cavity with reduced volume and cleared contents after evacuation. (c) Pre-evacuation CT image. (d and e) Follow-up CT images after evacuation. A hyperdense appearance corresponding to the stent is visible within the cyst cavity.

## SS-28 An Experimental Model for the Evaluation of Non-alcoholic Fatty Pancreatic Disease: Combined Examination of Endocrine and Exocrine Functions

**Sevtap Kılınç ^1^ , Pelin Şahin ^2^ , Müşerref Şeyma Ceyhan ^3^ , Zeynep Yığman ^4^ , Ayşe Meltem Sevgili ^2^**

^1^Department of Physiology, Başkent University Faculty of Medicine, Ankara, Türkiye

^2^Department of Physiology, Gazi University Faculty of Medicine, Ankara, Türkiye

^3^Department of Histology and Embryology, Namık Kemal University Faculty of Medicine, Tekirdağ, Türkiye

^4^Department of Histology and Embryology, Gazi University Faculty of Medicine, Ankara, Türkiye

**Background/Aims:** Non-alcoholic fatty pancreas disease (NAFPD) disrupts pancreatic structure, impairing both exocrine and endocrine functions, with a pathogenesis that is not fully understood. Limited studies exist on the effects of fat accumulation, and a reliable experimental model is lacking. This study aimed to develop a model to assess NAFPD’s dual impact on pancreatic functions.

**Materials and Methods:** A total of 144 male C57BL/6 mice were divided into 2 main groups for endocrine and exocrine assessments. Each group was further subdivided into standard or high-fat high-sucrose diet (HFHSD) groups and evaluated at 2, 4, 6, 8, 10, and 12 weeks, creating 24 experimental groups. Exocrine function was assessed by fecal fat; serum lipid profile, insulin, and amylase levels were measured. Oxidative stress was evaluated using TAS, TOS, and OSI. Histology included fat score, inflammation, and fibrosis. Analyses were performed using Jamovi (*P* < .05).

**Results:** HFHSD-fed groups gained weight, whereas glucose levels remained unchanged. LDL and periepididymal fat increased in all diet groups. Amylase, total cholesterol, and HDL increased from week 6. Insulin levels increased from week 2 (*P* = .030). Fecal fat score increased at week 6 (*P* = .008). Pancreatic fat increased from week 6 (*P* = .029), with no change in inflammation or fibrosis. Endocrine parameters (TAS, TOS, OSI) increased at week 8.

**Conclusion:** This is the first experimental NAFPD model allowing for the temporal assessment of exocrine and endocrine pancreatic functions in HFHSD-fed mice. Exocrine dysfunction began at week 6, whereas endocrine impairments appeared at week 8. This model provides a reliable foundation for understanding NAFPD pathophysiology and developing future preventive or therapeutic strategies.

## SS-29 Acute Pancreatitis: Has the Epidemiological Profile Changed Over The Past Decade?

**Yavuz Özden, Serkan Koz**

Department of Gastroenterology, Kayseri City Hospital, Kayseri, Türkiye

**Background/Aims:** Acute pancreatitis (AP) is one of the most frequent gastrointestinal emergencies requiring hospitalization. Its etiology covers a broad spectrum and may vary over time and across regions. Gallstones and alcohol are traditionally the major causes; however, lifestyle modifications, the increasing prevalence of obesity and metabolic syndrome, and advances in imaging have reshaped its epidemiological profile. This study aimed to evaluate the current etiological, clinical, and prognostic characteristics of AP cases in a tertiary center and compare these findings with regional data from the previous decade to assess epidemiological shifts.

**Materials and Methods:** Between November 2023 and July 2025, records of 1000 consecutive patients hospitalized with AP were retrospectively analyzed. Diagnosis and severity were defined by the 2012 Revised Atlanta criteria. Biochemical parameters (amylase, lipase, CRP, hematocrit, creatinine) at admission and 48 hours later were recorded. Ultrasonography, CT, and/or MR imaging reports were retrospectively reviewed from archived records. Etiologies were categorized as biliary, alcohol-related, hypertriglyceridemic, post-ERCP, or idiopathic. The analysis included severe pancreatitis, organ failure, local complications, and in-hospital mortality. Findings were compared with the November 2013–July 2015 cohort from the same center to identify time-based changes.

**Results:** The most common etiology was biliary (62.3%), followed by idiopathic (16.1%), alcohol (11.3%), hypertriglyceridemia (8.2%), and post-ERCP (2.1%). Severe AP occurred in 9.0%, persistent organ failure in 11%, and mortality was 2.5%. Biliary cases had higher rates of cholangitis and ERCP (44.5%, *P* < .01). Compared with 2013-2015, alcohol-related AP increased by 30%, whereas idiopathic cases declined, possibly due to improved diagnostic accuracy with EUS and advanced imaging modalities.

**Conclusion:** The etiological spectrum of AP has changed significantly over the past decade. The increase in alcohol-related cases and the decline in idiopathic cases reflect evolving lifestyle patterns and diagnostic improvements. Early recognition of etiology remains essential for guiding treatment and predicting outcomes. These findings highlight the dynamic nature of AP epidemiology and the importance of ongoing regional surveillance and updated prevention strategies.

## SS-31 Endoscopic Cystogastrostomy: 15-Year Single-Center Outcomes and Complications

**Levent Aktaş ^1^ , Sezgin Vatansever ^1^ , Hakan Çamyar ^1^ , Süleyman Günay ^2^**

^1^Department of Gastroenterology, İzmir Katip Celebi University Atatürk Training and Research Hospital, İzmir, Türkiye

^2^Memorial Göztepe Hospital, İstanbul, Türkiye

**Background/Aims:** Endoscopic cystogastrostomy is a minimally invasive method for treating pancreatic pseudocysts and walled-off necrosis (WON). The long-term outcomes, complications, and procedure-related mortality were evaluated in patients treated between 2010 and 2025.

**Materials and Methods:** A retrospective analysis was conducted on 196 procedures performed in 172 patients who underwent endoscopic cystogastrostomy. Data on demographics, lesion type, complications, and clinical outcomes were collected and analyzed.

**Results:** The mean age was 54.1 years, and males predominated (61.7%). Lesion distribution included pseudocysts (63.4%, n = 109) and WON (35.5%, n = 61). Among the 196 endoscopic cystogastrostomy procedures, drainage was technically insufficient in 28 cases (14.3%). The overall complication rate was 14.8%, with bleeding (6.1%) and stent-related issues (5.1%) being the most frequent. Severe complications such as fistulization, pneumothorax, and septicemia were rare (<1%). A total of 83.7% of patients were followed in good health for at least 6 months after the procedure. The overall mortality rate was 10.5% (n = 18), of which 2 were procedure-related. In the WON group, although demographic features were similar, mortality was significantly higher compared with the pseudocyst group (14.8% vs. 8.3%). Endoscopic cystogastrostomy is a safe method with a high success rate and low procedure-related mortality (~1%). Bleeding and stent-related problems remain the principal technical challenges in cystogastrostomy practice. The majority of deaths were attributable to progression of the underlying disease rather than procedural complications. Mortality in patients with WON was significantly higher, likely due to a greater systemic disease burden, indicating that outcomes are primarily determined by systemic frailty and inflammatory load rather than the procedure itself.

**Conclusion:** The results are consistent with trends reported in the literature. The increased mortality in WON is generally associated with systemic inflammatory response, infection, and multiorgan dysfunction rather than procedural failure, and enhanced multidisciplinary support is recommended for patients with WON.


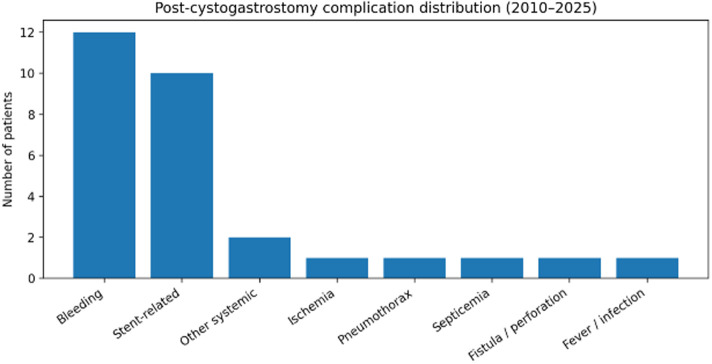


Figure 1. Distribution of complications after cystogastrostomy (2010-2025).

Table 1. Demographic Characteristics, Lesion Distribution, and Complications

Table 2. Procedure-Related Outcomes by Lesion Type

**Table:** 

Variable	Value
Age (years)	54.1
Sex (male), n (%)	106 (61.7)
Lesion distribution, n (%)	
Walled-off necrosis	61 (35.5)
Pseudocyst	109 (63.4)
Other	2 (1.2)
Patient-based mortality rate	
Walled-off necrosis	14.8% (9/61)
Pseudocyst	8.3% (9/109)
Complications	
Bleeding	12
Stent-related	9
Fistula	2
Infection/sepsis	1
Pneumothorax	1
Fever	1
Other (myocardial infarction, COVID-19, esophageal ischemia)	3
None	167

**Table:** 

Variable	Walled-off necrosis	Pseudocyst
Technical success rate (procedure-based)	81.3% (61/75)	88.2% (105/119)
Complication rate (procedure-based)	22.7% (17/75)	10.1% (12/119)
Mortality rate (procedure-based)	1 (1.4%)	1 (0.8%)

## SS-32 Deep Learning Model for Mayo Score Classification from Colonoscopy Images: Validation with CRP, Histological Activity, and Observer Agreement

**Gizem Dağcı, Mehmet Akif Yağlı, Besim Fazıl Ağargün, Pelin Telli, Asım Gurbanov, Sezen Genç Uluçeçen, Bilger Çavuş, Aslı Çiftçibaşı Örmeci, Kadir Demir, Selman Fatih Beşışık, Sabahattin Kaymakoğlu, Filiz Akyüz**

Division of Gastroenterology, Department of Internal Medicine, İstanbul University Faculty of Medicine, İstanbul, Türkiye

**Background/Aims:** In this study, an artificial intelligence (AI) model was developed that automatically assigns Mayo 0-3 scores to colonoscopy images performed at a single center. The model’s performance was compared with CRP, histological activity (Nancy score), and observer assessments.

**Materials and Methods:** In this single-center, retrospective study, 4000 colonoscopy images (approximately 1000 frames from each Mayo category) from approximately 150 patients with ulcerative colitis were analyzed. An average of 30-40 frames were selected from each patient to represent different colon segments.

Data were split into 80% training and 20% validation on an episode-by-episode basis.

EfficientNet-B0 (ImageNet pretrained) was used as the model.

Model performance was assessed using accuracy, macro-F1, macro-AUC, confusion matrix, and class boundary separations.

AI_prob_active_M2M3 = p̄(Mayo2) + p̄(Mayo3), calculated at the episode level, was compared with CRP and the Nancy score using Spearman correlation.

Human-AI agreement was measured by 2 independent endoscopists blindly evaluating a subset of samples.

**Results: **The model achieved 61.8% accuracy on the validation set, macro-F1 = 0.60, and macro-AUC = 0.94.

This agreement was similar to the interobserver agreement at the Mayo 1-2 boundary. The model performed excellently in distinguishing active from inactive (AUROC (0 vs. ≥1) = 0.96, AUROC (≤1 vs. ≥2) = 0.99).

A significant positive correlation was observed between AI-Mayo and CRP (ρ = 0.6, *P* < .01).

The AI_prob_active_M2M3 value demonstrated high discrimination power (AUROC = 0.85) in the presence of active histology (Nancy ≥1).

High agreement (κ = 0.8) was found between the 2 observers; model predictions largely aligned with the observers’ assessments.

**Conclusion:** The AI model distinguished Mayo 0 from 3 in colonoscopy images with near-human accuracy. The model’s high agreement with biochemical and histological activity demonstrates its potential for clinical decision support in the objective and reproducible assessment of mucosal healing.


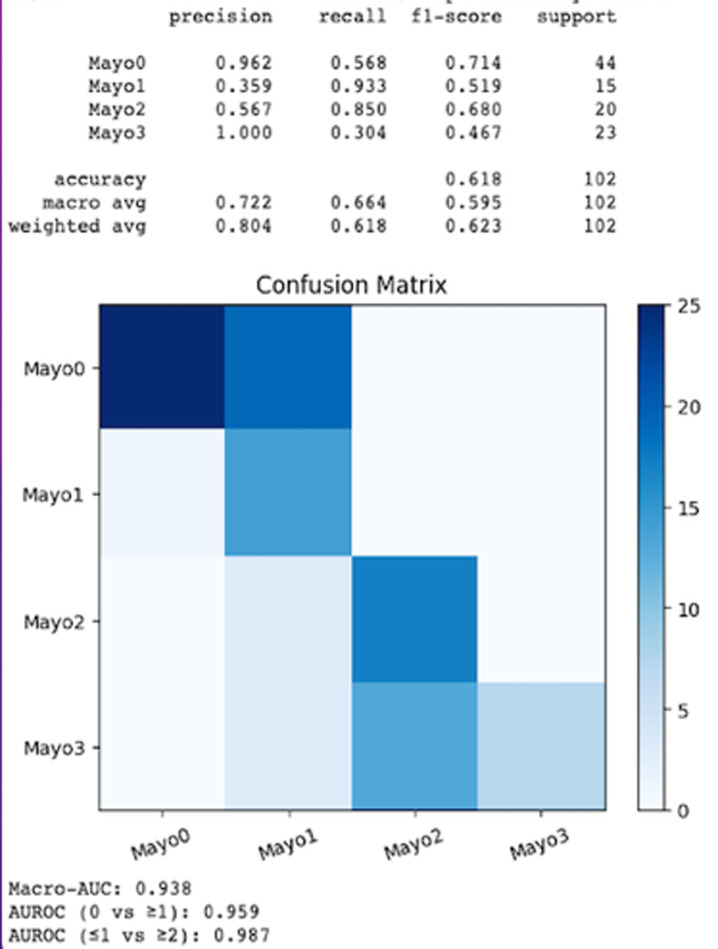


Figure 1. Discriminatory power of the model for Mayo 1-2 and Mayo 0 and 1.


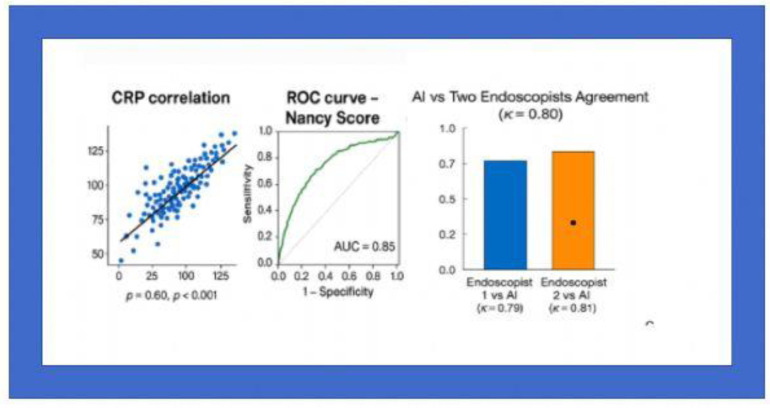


Figure 2. Correlation of AI-based Mayo classification scores with CRP and histologic (Nancy) activity, and agreement between 2 endoscopists and the AI model.

## SS-34 The Molecular Footprint of Time: How Disease Duration Shapes the Plasma Metabolome and Lipidome in Ulcerative Colitis?

### Gülden Bilican^1^, Engin Koçak^2^, Tarkan Karakan^1^

^1^Department of Gastroenterology, Gazi University Faculty of Medicine, Ankara, Türkiye

^2^Department of Pharmacology, University of Health Sciences Gülhane Faculty of Pharmacy, Ankara, Türkiye

**Background/Aims:** Ulcerative colitis (UC) is a chronic inflammatory bowel disease characterized by recurrent mucosal inflammation. Although numerous metabolomic and lipidomic studies have explored biomarkers for diagnosis or treatment response, the molecular impact of disease duration remains unclear. This study aimed to investigate how chronicity shapes the plasma metabolome and lipidome in UC.

**Materials and Methods:** Plasma samples from 58 patients with UC in clinical remission and 70 age- and sex-matched healthy controls were analyzed. Untargeted metabolomics and lipidomics analyses were performed using GC-MS and LC-MS platforms. Patients were subgrouped according to disease duration (≤2 years vs. >2 years). Data were analyzed using MetaboAnalyst 5.0, and pathway enrichment was based on the KEGG database.

**Results:** Distinct metabolic clustering differentiated UC from controls. Patients with UC showed reduced levels of β-alanine, lactic acid, pyruvic acid, and saturated fatty acids, whereas N2-(D-1-carboxyethyl)-L-arginine and 2-hydroxybutyric acid were increased. Altered pathways included fatty acid biosynthesis and amino acid metabolism. Lipidomics revealed significant changes in phosphatidylcholines (PC), lysophosphatidylcholines (LPC), phosphatidylethanolamines (PE), and oxidized phospholipids (OxPL). When stratified by duration, longer-standing disease was associated with altered amino acid and sphingomyelin pathways, reflecting metabolic remodeling over time.

**Conclusion:** Metabolic and lipidomic alterations persist in UC even during remission, and their intensity increases with disease duration. These findings highlight ongoing molecular adaptation and may aid in identifying metabolic markers for disease progression and personalized therapy.


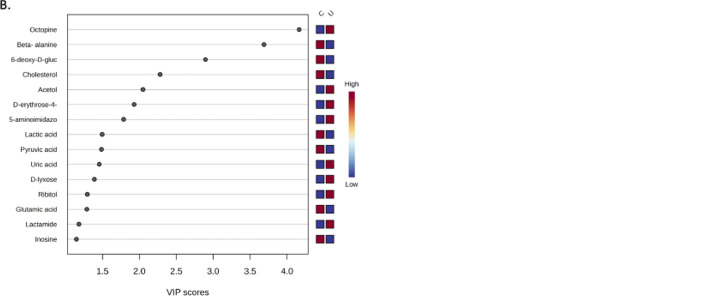


Figure 1. (A) PLS-DA score plot showing clear separation between patients with ulcerative colitis (UC) and healthy controls (HC). (B) VIP score plot displaying the top 15 discriminatory metabolites differentiating UC from HC.

## SS-35 Efficacy and Safety of First-Line Vedolizumab in Patients with Moderate-to-Severe Ulcerative Colitis: Real-World Data from the Southern Türkiye IBD Group (GünTürk-IBH)

**Orhan Sezgin ^1^ , Filiz Araz ^2^ , Sezgin Barutçu ^3^ , Nalan Gülşen Ünal ^4^ , Ümit Karaoğullarından ^5^ , Ayhan Balkan ^6^ , Kadri Atay ^7^ , Pırıl Akıncıoğlu ^8^ , Mehmet Hadi Yaşa ^9^ , Gözde Derviş Hakim ^10^ , Cumali Efe ^11^ , Burak Özşeker ^12^ , Ümit Karabulut ^13^ , Göksel Bengi ^14^ , Altuğ Şenol ^15^ , Zehra Betül Paköz ^16^ , Kadir Gişi ^17^ , Elif Tuğba Tuncel ^18^ , Halil Yılmaz ^19^ , Birol Özer ^2^ , Fehmi Ateş ^1^ , Hasan Mammadov ^1^ , Nevin Oruç ^4^ , Emre Odabaş ^5^ , Taylan Metin ^6^ , Harun Küçük ^7^ , Dinç Dinçer ^8^ , Eren Düzgün ^10^ , Ersin Batıbay ^11^**

^1^Department of Gastroenterology, Mersin University Faculty of Medicine, Mersin, Türkiye

^2^Department of Gastroenterology, Başkent University, Adana Training and Research Hospital, Adana, Türkiye

^3^Department of Gastroenterology, Sanko University Faculty of Medicine, Gaziantep, Türkiye

^4^Department of Gastroenterology, Ege University Faculty of Medicine, İzmir, Türkiye

^5^Department of Gastroenterology, Çukurova University Faculty of Medicine, Adana, Türkiye

^6^Department of Gastroenterology, Gaziantep University Faculty of Medicine, Gaziantep, Türkiye

^7^Department of Gastroenterology, Mardin Training and Research Hospital, Mardin, Türkiye

^8^Department of Gastroenterology, Akdeniz University Faculty of Medicine, Antalya, Türkiye

^9^Department of Gastroenterology, Adnan Menderes University Faculty of Medicine, Aydın, Türkiye

^10^Department of Gastroenterology, İzmir City Hospital, İzmir, Türkiye

^11^Department of Gastroenterology, Harran University Faculty of Medicine, Şanlıurfa, Türkiye

^12^Department of Gastroenterology, Muğla University Faculty of Medicine, Muğla, Türkiye

^13^Department of Gastroenterology, Diyarbakır Training and Research Hospital, Diyarbakır, Türkiye

^14^Department of Gastroenterology, Dokuz Eylül University Faculty of Medicine, İzmir, Türkiye

^15^Department of Gastroenterology, Süleyman Demirel University Faculty of Medicine, Isparta, Türkiye

^16^Department of Gastroenterology, Katip Çelebi Katip Çelebi, İzmir, Türkiye

^17^Department of Gastroenterology, Kahramanmaraş Sütçü İmam University Faculty of Medicine, Kahramanmaraş, Türkiye

^18^Department of Gastroenterology, Celal Bayar University Faculty of Medicine, Manisa, Türkiye

^19^Department of Gastroenterology, Pamukkale University Faculty of Medicine, Denizli, Türkiye

**Background/Aims:** To evaluate the efficacy and safety of vedolizumab (VDZ) as a first-line treatment in moderate-to-severe ulcerative colitis (UC).

**Materials and Methods:** This multicenter real-world study included patients from 18 centers across 14 cities (GünTürk-IBH network). Patients with moderate-to-severe UC who received vedolizumab between November 2024 and September 2025 were included. Clinical response was defined as a ≥3-point reduction in the partial Mayo Score (PMS), and clinical remission as PMS ≤ 1. At week 14, steroid-free clinical remission (SFCR), clinical response, clinical remission rates, and adverse events were evaluated.

**Results:** A total of 122 patients with UC were analyzed. The mean age was 47.4 ± 17.9 years, and 48% were female. The mean treatment duration was 25.9 ± 23.45 weeks. Extensive/pancolitis was present in 61.5% and proctitis or left-sided colitis in 38.5% of patients. Steroids were initiated with VDZ in 50% of cases. The median baseline PMS was 7.00 (5.00-8.00), decreasing to 1.00 (0.00-3.00) at week 14 (*P* < .001). Median CRP declined from 9.00 (2.98-19.20) mg/L to 3.75 (1.50-7.65) mg/L (*P* < .001), and fecal calprotectin from 743.5 (324.0-840.0) µg/g to 126.0 (30.0-180.0) µg/g (*P* = .004). SFCR was achieved in 42%, clinical response in 78%, and clinical remission in 52% of all patients. The steroid withdrawal rate at week 14 was 72.1%. Clinical response was observed in 85.3% of extensive and 66.0% of patients with left-sided colitis (*P* = .012), whereas clinical remission and SFCR rates were similar. Clinical outcomes were comparable across age groups (*P* > .05). Compared to moderate disease, clinical response (63.0% vs. 89.7%, *P* = .001) and SFCR (31.5% vs. 50.0%, *P* = .039) rates were higher in severe disease, whereas clinical remission rates were similar (*P* = .93). The adverse event rate was 11.7%. The serious adverse event rate leading to treatment discontinuation was 0.8%.

**Conclusion:** In biologic-naïve patients with moderate-to-severe UC, vedolizumab was found to be effective and safe as a first-line therapy.

Table 1.Comparison of Clinical Response and Remission Rates According to Disease Extent, Severity, and Age Categories

**Table:** 

		Clinical Response		Clinical Remission		Steroid-Free Clinical Remission	
n (%)	*P*	n (%)	*P*	n (%)	*P*
Disease location	Extensive pancolitis	64 (85.3)	.012	43 (57.3)	.112	34 (45.3)	.318
	Left-sided distal colitis	31 (66.0)		20 (42.6)		17 (36.2)	
Age (years)	<40 years	35 (77.8)	.215	23 (51.1)	.622	19 (42.2)	.433
	40-65 years	45 (83.3)		30 (55.6)		25 (46.3)	
	>65 years	15 (65.2)		10 (43.5)		7 (30.4)	
Disease severity	Moderate	34 (63.0)	0.001	28 (51.9)	.967	17 (31.5)	.039
	Severe	61 (89.7)		35 (51.5)		34 (50.0)	

## SS-36 Investigation of the Frequency of Cardiometabolic Disease in Inflammatory Bowel Disease

**Erman Mercan ^1^ , Murat Yıldırım ^1^ , Doğancan Akyürek ^1^ , Şefikcan Biricik ^1^ , Semra Dağdelen ^1^ , Merve Ceren Ceylan Eroğlu ^1^ , İlker Şen ^1^ , Emrullah Düzgün Erdem ^1^ , Mehtap Uçar ^1^ , Canan Alkım ^2^ , Hüseyin Alkım ^2^**

^1^Department of Gastroenterology, Şişli Hamidiye Etfal Training and Research Hospital, İstanbul, Türkiye

^2^Department of Gastroenterology, University of Health Science, Şişli Hamidiye Etfal Training and Research Hospital, İstanbul, Türkiye

**Background/Aims:** A common inflammatory mechanism is thought to underlie both intestinal inflammation and metabolic disorders. This study aimed to investigate the prevalence of cardiometabolic disease (CMD) among individuals with inflammatory bowel disease (IBD).

**Materials and Methods:** A total of 1035 patients (534 with ulcerative colitis [UC] and 501 with Crohn’s disease [CD]) followed at the center between 2008 and 2025 were included. Demographic and clinical characteristics, including gender, age, disease duration, biological therapy exposure, and the presence of CMD (diabetes mellitus, hypertension, coronary artery disease, cerebrovascular disease, peripheral artery disease, or hyperlipidemia), were evaluated. Univariate analyses were performed using the chi-square and Mann–Whitney *U*-tests, and multivariate analysis was conducted using logistic regression.

**Results:** CMD was more prevalent in patients with UC than in those with CD (30.7% vs. 14.6%). Among patients with UC, those with CMD were older (median 60 vs. 41 years, *P* < .001) and had longer disease duration (median 144 vs. 96 months, *P* < .001). CMD was more common in men with UC (37.0% vs. 23.5%, *P* < .001). In multivariate analysis, age (OR = 1.09) and male sex (OR = 2.05) were independently associated with CMD, whereas disease duration, anti-TNF exposure, and experience with ≥2 biologics were not.

Among patients with CD, those with CMD were older (median 56 vs. 42 years, *P* < .001) and had longer disease duration (median 124 vs. 108 months, *P* = .005). CMD was more prevalent among women (19.6% vs. 11.0%, *P* = .007) and in those with anti-TNF experience (17.5% vs. 10.5%, *P* = .030). In multivariate analysis, age (OR = 1.07) and anti-TNF exposure (OR = 3.12) were independently associated with CMD, whereas sex, disease duration, and experience with ≥2 biologics were not.

**Conclusion:** The cardiometabolic risk profile differs between IBD subtypes. Special attention should be paid to older patients and those with prolonged disease duration or experience with biologic therapy, as these factors may indicate an increased risk for CMD.

## SS-37 A Functional Cure After Nucleos(t)ide Analog Discontinuation in Noncirrhotic Patients with HBeAg-Negative Chronic Hepatitis B

**Zeynep Melekoğlu Ellik, Özge Koç, Volkan Yılmaz, Mesut Gümüşsoy, Ramazan Erdem Er, Hale Gökcan, Ramazan İdilman**

Department of Gastroenterology, Ankara University School of Medicine, Ankara, Türkiye

**Background/Aims:** Discontinuation of long-term nucleos(t)ide analog(NA) treatment can induce a functional cure in patients with chronic hepatitis B (CHB);however, data are scarce. This study aimed to evaluate HBsAg loss after NA treatment discontinuation in noncirrhotic patients with HBeAg-negative CHB and to determine virological outcomes after NA discontinuation.

**Materials and Methods:** This single-center cohort study included 146 noncirrhotic, patients with HBeAg-negative CHB who achieved long-term viral suppression under NA treatment. Seventy-three patients discontinued NA therapy after more than 5 years of treatment and at least the last 3 years of virological suppression. The historical age- and sex-matched control group comprised 73 patients who continued NA therapy for a 60-month observational period. HBV reactivation was defined as HBVDNA > 2000 IU/mL with serum ALT levels exceeding 2 times the upper limit of normal. The primary endpoint was sustained HBsAg loss for at least 6 months, whereas the secondary outcome was virological and biochemical response at month 60 after NA cessation.

**Results:** Patients’ characteristics were similar in both groups. HBsAg loss occurred in 8 patients (11%) in the discontinuation group, whereas none were observed in the treatment continuation group (*P* = .004). The cumulative probability of HBsAg loss was 13% at month 120. Multivariate Cox regression analysis revealed that a younger age at NA cessation (<50 years) (*P* = .033)and abnormal baseline serum AST level (≥40U/L) (*P* = .027) were independent predictors of HBsAg loss. Anti-HBs seroconversion was observed in 6 patients with HBsAg loss. Forty-four patients (60%) experienced sustained remission. HBV reactivation occurred in 29 patients, primarily (72%) within the first 3 months after treatment discontinuation. Patients’ characteristics were not associated with HBV reactivation. HBV reactivation was slightly higher in patients with an HBsAg titer of >1000 IU/mL at NA discontinuation than in those with <1000 IU/mL (*P* = .097). NA retreatment was initiated in all relapsed patients, resulting in a virological and biochemical response upon re-initiation of therapy.

**Conclusion:** NA discontinuation in noncirrhotic patients with HBeAg-negative CHB was associated with a functional cure. NA discontinuation is safe and tolerable in selected patients with CHB.

## SS-40 ERCP Results in Pediatric Liver Transplant Patients

**Hakan Yıldız ^1^ , Fatih Oğuz Önder ^2^ , Vildan Ertekin ^3^ , Hamdi Karakayalı ^4^**

^1^Department of Gastroenterology, New Century University, İstanbul, Türkiye

^2^Department of Gastroenterology, Acıbadem Mehmet Ali Aydınlar University, İstanbul, Türkiye

^3^Department of Pediatric Gastroenterology, Acıbadem Mehmet Ali Aydınlar University, İstanbul, Türkiye

^4^Department of General Surgery, Acıbadem Mehmet Ali Aydınlar University, İstanbul, Türkiye

**Background/Aims:** The study aimed to investigate the side effects and safety of ERCP in pediatric liver transplant patients.

**Materials and Methods:** Patients under the age of 18 who underwent living-donor liver transplantation and developed biliary complications were retrospectively analyzed.

**Results:** Between 2020 and 2025, 571 patients underwent liver transplantation, 116 (20.3%) of whom were pediatric patients. The median age at transplantation was 4.3 (1.08-15.59) years. Fourteen (12%) patients underwent ERCP, and the median age at ERCP was 7.47 (3.18-15.8) years. One (6%) of the patients underwent ERCP due to a biliary leak, whereas all other patients underwent ERCP due to biliary anastomotic stenosis. Each patient received prophylactic antibiotics before the procedure. The median follow-up period for the patients was 9.08 (1.48-23.91) months. Selective cannulation was used in 10 (66.7%) patients, and fistulotomy with a needle was performed in 4 (26.7%). The total number of ERCP sessions was 40, with a median of 3 (1-6) per patient. Balloon dilatation was performed using a 6-mm balloon in 3 (21.4%) patients and with a Sohendra in 1 (6%) patient. The success rate of cannulation and the procedure was 100%. In the postprocedure follow-up, increased pancreatic enzyme levels were observed in 3 (21.4%) patients, and none of them experienced pain. Cholangitis developed in 1 (6%) patient after stent removal.

**Conclusion:** ERCP can be safely performed in pediatric patients over 1 year of age or weighing more than 10 kg. Biliary complications are more common in adults due to the size of the common bile duct or the difference in diameter between the donor and recipient. The success rate of LDLT ERCP in pediatric patients has been reported to be between 58% and 76%. In this study, cannulation and procedural success were found to be high. The specialized materials used in pediatric patients may contribute to this success. ERCP is a safe and highly successful procedure and can be used as the first-line treatment for patients with biliary strictures.


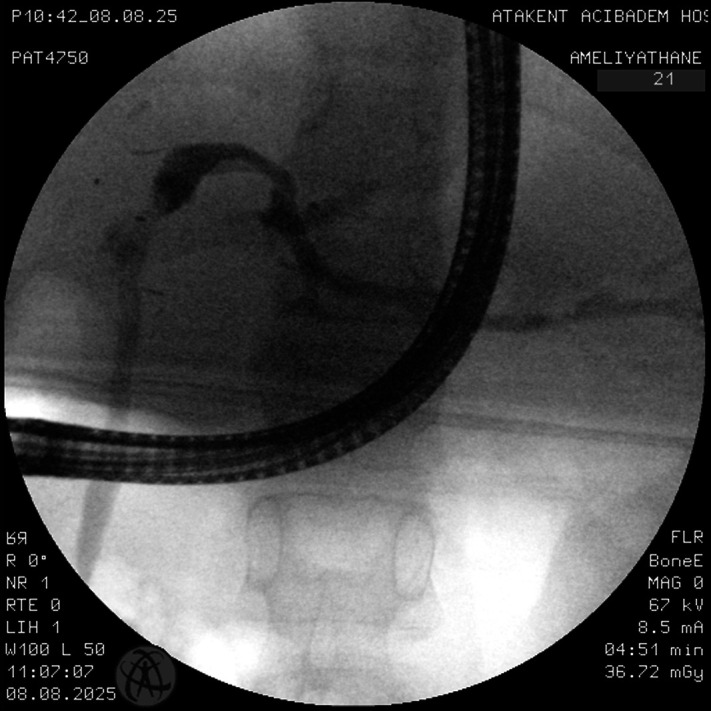


Figure 1. Biliary stricture under fluoroscopy in a LDLT patient.


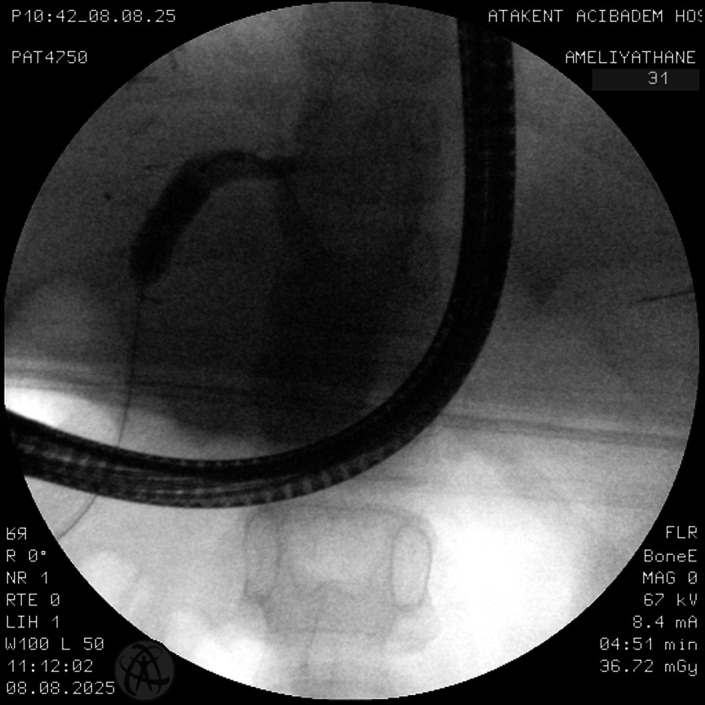


Figure 2. Balloon dilatation procedure in the biliary stricture area with a 6-mm balloon.

## SS-44 Prevalence and Risk Factors of Portal Hypertensive Gastropathy: A Meta-Analysis

**Gizem Dağcı, Mehmet Akif Yağlı, Bilger Çavuş, Aslı Çiftçibaşı Örmeci, Filiz Akyüz, Kadir Demir, Selman Fatih Beşışık, Sabahattin Kaymakoğlu**

Division of Gastroenterology, Department of Internal Medicine, İstanbul University Faculty of Medicine, İstanbul, Türkiye

**Background/Aims:** Portal hypertensive gastropathy (PHG) is a common complication in patients with portal hypertension; however, its prevalence and associated risk factors have been variably reported. This meta-analysis aimed to provide a pooled prevalence estimate and identify significant risk factors for PHG.

**Materials and Methods:** A systematic review identified studies reporting PHG prevalence and risk factors in adults with portal hypertension. Four eligible studies comprising 1035 patients were included. Pooled prevalence rates were calculated using inverse variance weighting. Odds ratios (OR) for risk factors were derived using a fixed-effects model. Between-study heterogeneity was assessed using the I^2^ statistic.

**Results:** The pooled prevalence of PHG was 79.2% (95% CI: 76.9-81.6). Significant risk factors included hypoalbuminemia, thrombocytopenia, the presence of esophageal and gastric varices, and an increased hepatic venous pressure gradient (HVPG). The combined OR for these risk factors was 1.78 (95% CI: 1.68-1.89). Minimal heterogeneity was observed across studies (I^2^ = 12%), supporting the consistency of the findings.

**Conclusion:** PHG is highly prevalent in patients with portal hypertension, with hypoalbuminemia, varices, and increased HVPG emerging as key risk factors. Early identification of high-risk patients is critical to optimizing management, particularly endoscopic surveillance. Integrating clinical risk factors with endoscopic findings may refine prognostic models and guide therapeutic strategies for PHG.

## SS-45 The Efficacy of Multiple Stenting from the Minor Papilla in Achieving Sustained Remission of Recurrent Acute Pancreatitis

**Hakan Şentürk ^1^ , İbrahim Hakkı Köker ^2^ , Erkan Çağlar ^3^ , Sercan Kiremitçi ^1^ , Şerife Değirmencioğlu Tosun ^4^ , Ali Tüzün İnce ^1^**

^1^Department of Gastroenterology, Bezmialem Foundation University Faculty of Medicine, İstanbul, Türkiye

^2^Department of Gastroenterology, Başkent University Faculty of Medicine, İstanbul, Türkiye

^3^Department of Gastroenterology, Sarıkız Gastroenterology Center, Balıkesir, Türkiye

^4^Department of Gastroenterology, University of Health Sciences Bağcılar Training and Research Hospital, İstanbul, Türkiye

**Background/Aims:** Pancreas divisum (PD) is one of the causes of recurrent acute pancreatitis (RAP). Its diagnosis may be missed, which can result in chronic pancreatitis and, in rare cases, ductal adenocarcinoma. Treatment aims to provide sustained dilation of the minor papillary orifice. This study aimed to assess the efficacy of multiple plastic stenting of the main pancreatic duct through the minor papilla in PD cases.

**Materials and Methods:** The data of 24 PD patients were evaluated with RAP treated between 2011 and 2023, who underwent multiple plastic stenting with at least 24 months of follow-up.

**Results:** There were 11 females and 13 males, with a median age of 44 (range, 17-65). The patients underwent pancreatic sphincterotomy with plastic stents sized 7-10 F. After 6 months, either the size of the stent was increased or the number of stents were increased according to the size of the main pancreatic duct. This was continued at 6-month intervals until satisfactory calibration was obtained. The median number of plastic stents was 2 (1-3). The median number of sessions was 4 (3-6). Balloon dilation was also performed with 4- to 8-mm balloons for some patients. The maximum size of stents was 2 10 F plus 1 7 F. After retrieval of the stents, whereas no RAP occurred in 19 patients, RAP occurred with stricture of the minor papillary orifice in 5 patients who required further endoscopic treatment. All recurrences happened in the second year of follow-up: median 17 months (range, 15-23). No serious side effects occurred.

**Conclusion:** Management of pancreas divisum with multiple stenting is safe and has long-term benefits in 79.1% (19/24) of patients. However, in longer-term follow-up, more recurrences may be expected. Recently, some patients were treated with SEMS, and data arising from small numbers are encouraging, showing fewer sessions (unpublished observation).

## SS-47 EUS-guided Pancreatic Duct Drainage: Which Patients, How to Perform It? Is It as Complicated as Thought?

**Sercan Kiremitçi ^1^ , Gülseren Seven ^2^ , İbrahim Hakkı Köker ^3^ , Şerife Değirmencioğlu Tosun ^4^ , Ali Tüzün İnce ^1^ , Hakan Şentürk ^1^**

^1^Department of Gastroenterology, Bezmialem Foundation University Faculty of Medicine, İstanbul, Türkiye

^2^Department of Gastroenterology, Zincirlikuyu Medicana Hospital, İstanbul, Türkiye

^3^Department of Gastroenterology, Başkent University Faculty of Medicine, İstanbul, Türkiye

^4^Department of Gastroenterology, University of Health Sciences Bağcılar Training and Research Hospital, İstanbul, Türkiye

**Background/Aims:** EUS-guided pancreatic duct drainage (EUS-PDD) is one of the most technically challenging procedures. The most common indications include pancreatic duct strictures, stones, and altered anatomy due to surgery. This study aims to evaluate patients requiring EUS-PDD, the indications and types of procedures performed, and the success and complication rates.

**Materials and Methods:** The data of 8 patients who underwent EUS-PDD between 2020 and 2024 due to various reasons that made retrograde pancreatography impossible were reviewed.

**Results:** A total of 22 procedures were performed on 8 male patients. The median age was 58.5 years (range 21-75). Seven patients had pancreatic duct stones that prevented retrograde cannulation, 6 patients had findings of simultaneous chronic pancreatitis, and 4 had pancreatic duct strictures. The remaining patient had a malignant lesion. In this series, 1 patient received only a plastic stent, 1 received a metal stent, and the remaining 6 patients underwent drainage with a combination of plastic and metal stents in subsequent procedures. Three patients underwent dual procedures (EUS-PDD + ERCP) in follow-up sessions, 2 patients received spyglass-assisted laser lithotripsy following EUS-PDD with an appropriate fistula created, 2 patients were referred to extracorporeal shock wave lithotripsy for pancreatic duct stones after EUS-PDD, and 1 patient underwent PDD only. The technical success rate for EUS-PDD was 100%, and the clinical success rate was 82%. There were no serious complications such as perforation or mortality; however, 5 of the 8 patients experienced symptoms such as pain and transient fever after the procedures.

**Conclusion:** EUS-PDD is crucial for accessing the pancreatic duct in cases of strictures or altered anatomies that cannot be managed via retrograde approaches. Although it is technically challenging compared to other interventional endoscopic procedures, it does not have a higher complication rate than might be expected. When performed by experienced endoscopists, it has a high safety profile.

## SS-48 Long-Term Outcomes and Predictors of Relapse After Biologic Discontinuation in Inflammatory Bowel Disease

**Yasemin Armutcuoglu ^1^ , Tuğba Tolu Bülte ^1^ , Uğur Çiftci ^1^ , Betül Piyade ^2^ , Rabia Ertürk ^2^ , Haluk Tarık Kani ^1^ , Özlen Atuğ ^1^ , Yeşim Özen Alahdab ^1^**

^1^Department of Gastroenterology, Marmara University School of Medicine, İstanbul, Türkiye

^2^Department of Internal Medicine, Marmara University School of Medicine, İstanbul, Türkiye

**Background/Aims:** Biological therapies are crucial for inducing and maintaining remission in inflammatory bowel disease (IBD). Whether they can be safely discontinued in patients with sustained remission remains uncertain. This study aimed to evaluate the long-term outcomes and predictors of relapse in patients with who discontinued biologic therapy during remission.

**Materials and Methods:** This single-center retrospective cohort included patients with IBD treated with biological therapy for ≥6 months who discontinued treatment between 1995 and 2024. Relapse was defined by clinical symptoms, biomarkers, and endoscopic disease activity scores. Predictors of relapse were assessed using univariate analysis and multivariate logistic regression. Model performance was assessed with receiver operating characteristic (ROC) curve analysis.

**Results:** A total of 62 patients were included (mean age: 50.7 ± 14.3 years; 53.2% female);74.2% had Crohn’s disease and 25.8% had ulcerative colitis. Biologic agents used included infliximab (61.3%) and adalimumab (38.7%). The mean duration of biologic therapy prior to discontinuation was 56.4 ± 28.7 months. Reasons for withdrawal included sustained remission (64.5%), patient preference (11.3%), adverse events (8.1%), and other causes (16.1%). During a mean follow-up of 43.7 months, the overall relapse rate was 37.1%. Relapse occurred in 9.8% of patients within the first year, 7.5% in the second year, 12.2% in the third year, and 9.3% by the fifth year. Multivariate logistic regression analysis identified enteric fistula as an independent predictor of relapse (OR: 10.1; 95% CI: 1.56-65.5; *P* = .015). Clinical remission at the time of biologic discontinuation showed a protective trend against relapse (OR: 0.09; *P* = .057). The discriminatory ability of the final model was acceptable, with an area under the curve (AUC) of 0.744 (*P* = .009).

**Conclusion:** Approximately one-third of patients with IBD relapsed following biologic discontinuation. Enteric fistula was significantly associated with an increased risk of relapse, whereas clinical remission at the time of discontinuation was protective. Findings highlight the importance of individualized discontinuation strategies, particularly in fistulizing disease.

## SS-49 The Role of Circadian Genes in Ulcerative Colitis

**Suleyman Yıldırım ^1^ , Ceren Tunçalp ^2^ , Memduh Şahin ^1^ , Celalettin Herek ^1^ , Sacide Pehlivan ^2^**

^1^Department of Gastroenterology, Başakşehir Çam and Sakura City Hospital, İstanbul, Türkiye

^2^Department of Medical Biology and Genetics, İstanbul University, İstanbul, Türkiye

**Background /Aims:** Although circadian rhythm genes are primarily known for regulating the body’s rhythmic timing, accumulating evidence suggests that these genes also play fundamental regulatory roles in the development of inflammation. This study aimed to evaluate the possible associations between functional variants of the BMAL1, CLOCK, and CRY1 genes—which regulate circadian rhythm—and disease susceptibility, clinical activity, biochemical parameters, and treatment requirements in patients with ulcerative colitis (UC).

**Materials and Methods:** A total of 107 patients with UC and 80 healthy controls were included in the study. Peripheral blood samples were collected for DNA isolation, and the BMAL1-rs7950226, CLOCK-rs1801260, and CRY1-rs2287161 gene variants were analyzed using polymerase chain reaction (PCR). Genotype and allele frequencies, as well as their associations with clinical and biochemical parameters, were statistically evaluated.

**Results:** The BMAL1-rs7950226 AA genotype was more frequent in the control group, although the difference was not statistically significant (*P* = .68).

For the CRY1-rs2287161 variant, the GC genotype was significantly more common in patients with UC (*P* = .004), suggesting a potential association with disease susceptibility, whereas the GG genotype, which was more frequent among controls, may have a protective role.

No significant difference was observed for the CLOCK-rs1801260 variant between groups; however, a higher frequency of azathioprine use in patients carrying the TT genotype (*P* = .020) suggests a possible link between CLOCK polymorphism and treatment requirements or a positive therapeutic response.

**Conclusion:** This study demonstrates that among circadian rhythm-related genes, CRY1 variants may play a role in the pathogenesis of ulcerative colitis. The CLOCK gene variant appears to be noteworthy due to its potential association with treatment response.

Overall, the findings emphasize the potential importance of circadian genetic variations in inflammatory bowel diseases and highlight the need for further studies to elucidate their mechanistic role in disease development.

## SS-50 Prevalence and Risk Factors of Thromboembolism in Inflammatory Bowel Disease

**Okan Katı ^1^ , Oğuz Kağan Bakkaloğlu ^1^ , Ali İbrahim Hatemi ^1^ , Aykut Ferhat Çelik ^1^ , Emire Seyahi ^2^ , Yusuf Ziya Erzin ^1^**

^1^Department of Gastroenterology, İstanbul University-Cerrahpaşa Cerrahpaşa Medical Faculty, İstanbul, Türkiye

^2^Department of Rheumatology, İstanbul University-Cerrahpaşa Cerrahpaşa Medical Faculty, İstanbul, Türkiye

**Background/Aims: **Inflammatory bowel disease (IBD), a chronic inflammatory disorder, may promote atherosclerosis, arterial events, and venous thromboembolism (VTE). This study aimed to determine the frequency and risk factors of acute thromboembolism in the IBD cohort.

**Materials and Methods: **A total of 3133 patients (1414 with Crohn’s disease [CD], 1667 with ulcerative colitis [UC], and 52 with IBD-unclassified [IBD-U]) were retrospectively analyzed. Patients who developed acute arterial or venous thromboembolism (n = 39) during follow-up were compared with 78 controls matched for sex and diagnosis. Those with Behçet’s syndrome (n = 126), systemic vasculitis (n = 16), or hereditary thrombophilia (n = 5) were excluded.

**Results:** Among 3133 patients with IBD, 124 (3.95%) had arterial or venous thromboembolic events, totaling 132 events (86 arterial, 46 venous). Coronary artery disease was significantly more frequent in UC than in CD (2.3% vs. 1.2%; *P* = .028). During follow-up, 40 acute events (25 arterial, 15 venous) occurred in 39 patients (1.24%), with no significant difference between CD and UC (*P* = .871). Exacerbation, hospitalization, and disease activity were associated with both arterial and venous events. Smoking, age at diagnosis, body mass index, nonmucosal CD (Montreal B2–B3), and higher baseline CRP were linked only to arterial events, whereas higher CRP at the last visit, annual exacerbation frequency, additional inflammatory disease, complications (surgery, abscess), longer cumulative steroid exposure, and recent steroid use were associated only with venous events. In regression analysis, recent steroid therapy and additional inflammatory disease were independent predictors of venous thromboembolism, whereas older age at IBD diagnosis was independently associated with arterial events.

**Conclusion: **In addition to traditional risk factors such as age, smoking, and obesity, parameters indicating disease activity, inflammatory comorbidities, and recent corticosteroid exposure appear to increase the risk of acute thromboembolism in patients with IBD.

Table 1.IBD-Related Risk Factors

CRP, C-reactive protein; IBD, inflammatory bowel disease; IQR, interquartile range; VE, vascular event.

^a^Pearson chi-square test.

Table 2.Demographics and Non-IBD Risk Factors

BMI, body mass index; CD, Crohn’s disease; IQR, interquartile range; UC, ulcerative colitis; VE, vascular event.

^a^Mann–Whitney *U*-test.

^b^Independent samples *t*-test.

^c^Pearson chi-square test.

**Table:** 

	VE (n = 40)	Control (n = 78)	*P*	Arterial	Venous	*P*Arterial. vs. Control	*P*Venous vs. Control
Hospital admission, n (%)	20 (50)	13 (16.6)	**<.0011**	12 (48)	8 (53.3)	**.001**	**.002**
Number, [med (IQR)]	0.5 (1)	0 (0)	**<.0012**	1 (1)	1 (1)	**.0042**	**.0062**
Exacerbation, n (%)	33 (82.5)	40 (51.2)	**.0011**	20 (80)	13 (87)	**.0113**	**.0111**
* *Per year, med (IQR)	0.45 (1.02)	0.052 (0.23)	**<.0012**	0.9 (1.9)	1 (0.7)	.0582	**.0042**
Steroid use, n (%)	25 (62.5)	40 (51.2)	.2761	14 (56)	11 (73)	.7243	.1271
Steroid exposure (% follow-up period) [med (IQR)]	0.20 (0.46)	0.04 (0.07)	**.0082**	0.14(0.4)	0.2 (0.8)	.1212	**.0332**
Endoscopic remission, n (%)	11 (27.5)	38 (48.7)	**.0201**	7 (28)	4 (27)	.0553	.1551
IBD related							
Complications, n (%)	10 (25)	5 (6.4)	**.0501**	4 (16)	4 (27)	.2511	**.03 ^a^**
Surgery, n (%)	9 (22.5)	11 (14.1)	.2501	4 (16)	5 (33)	.7551	.0713
Last visit							
Clinically active, n (%)	18 (45)	12 (15.3)	**<.0011**	9 (36)	9 (60)	**.0183**	**<.0013**
Steroid therapy, n (%)	9 (22.5)	3 (3.8)	**.0031**	2 (8)	7 (47)	.5931	**<.0011**
Lowest CRP (mg/L) [med (IQR)]	1.47 (2.33)	0.9 (1.85)	**.0212**	2.2 (2.5)	1.3(1.3)	**.0252**	.5422

**Table:** 

	VE (n = 39)	Control (n = 78)	*P*VE vs. C.	aVE (n = 25)	vVE (n = 14)	*P*Art. Vs. V	*P*Art. vs. C	*P*Ven. vs. C
Diagnosis								
UC, n (%)	61.5 (24)	61.5 (48)	–	68 (17)	50 (7)	.273	.723	.423
CD, n (%)	38.5 (15)	38.5 (30)		32 (8)	50 (7)			
Gender								
Male, n (%)	53.8 (21)	53.8 (42)	–	64 (16)	35.7 (5)	.093	.33	.213
BMI, kg/m^2^ mean ± SD	26.3 ± 4.6	24.5 ± 4.8	.0671	28.5 (11)	26 (8)	.2361	**.0331**	.3431
Age (years), median(IQR)								
At diagnosis	45.5 (14)	31 (15)	**.0012**	48 (13)	37 (14)	**.0092**	**<.001 ^a^**	.5422
At VE	51.5 (16)	–	–	55 (13)	47 (16)	**.009**	–	–
Smoking								
Current, n (%)	79 (31)	58 (45)	**.0333**	84 (21)	66 (10)	.2043	**.0173**	.5173
Pack-year, median (IQR)	20 (20)	11 (16)	.1512	20 (20)	17.5 (16)	.662	.12	.652
Extraintestinal, n (%)	45 (18)	47 (37)	.8	40 (10)	50 (14)	.54	.65	.86
Comorbidities, n (%)								
Inflammatory	23 (9)	9 (7)	**.043**	16 (4)	35.7 (5)	.2343	.3223	**.033**
Malignancy	10.2 (4)	7.6 (6)	.73	8 (2)	14.2 (2)	.6083	13	.3513
DM/HT	23 (9)	12.8 (10)	.1763	36 (9)	7.1 (1)	**.043**	**.0093**	.543
Follow-up months, median (IQR)								
Total	101.5 (113)	105.5 (134)	.8462	98 (91)	112 (131)	.832	.252	.772
Before VE	49.5 (106)	–		52 (87)	45 (111)	.832	–	–

## SS-51 Upadacitinib in Biologic-experienced Inflammatory Bowel Disease: Safety and Laboratory Results

**Osman Özdoğan, Serkan Yaraş, Mehmet Kasım Aydın, Oktay Bayraktar, Hasan Mammadov, Ahmet Emre Ergan, Ümit Yeşilova, Fehmi Ateş, Engin Altıntaş, Orhan Sezgin**

Department of Internal Medicine and Gastroenterology, Mersin University Faculty of Medicine, Mersin, Türkiye

**Background/Aims:** The safety profile of JAK inhibitors, particularly concerning cardiovascular events and infections, is a critical consideration in the long-term management of inflammatory bowel disease (IBD). This study aimed to provide real-world data on the safety and effects on laboratory parameters of Upadacitinib in a cohort of biologic-experienced patients with moderate-to-severe Crohn’s disease (CD) and ulcerative colitis (UC).

**Materials and Methods:** This retrospective cohort study included 41 patients with IBD (22 UC, 19 CD). Safety assessments, including the monitoring of adverse events (AEs), were conducted at each visit. The study also prospectively evaluated changes in various laboratory parameters at baseline and at 3 and 6 months, including lipid profiles (total cholesterol, LDL-C, HDL-C, triglycerides), liver and kidney function tests (AST, ALT, creatinine), glucose, and hemogram parameters.

**Results:** No serious adverse events, venous thromboembolism (VTE), major adverse cardiac events (MACEs), or deaths were observed throughout the study period. A total of 20 patients (48.8%) experienced adverse events, with the most common being infections (17.1%) and dermatological issues (12.2%). Two patients discontinued the medication due to severe nausea and vomiting during the induction phase. Upadacitinib therapy led to a significant increase in TC, LDL-C, and HDL-C levels. TC levels increased from a baseline mean of 173.3 ± 41.8 mg/dL to a peak of 214.1 ± 50.1 mg/dL at month 3. However, the LDL-C/HDL-C ratio remained unchanged (*P* = .233). Although albumin levels significantly increased, vitamin B12, folic acid, and ferritin levels showed a significant decrease. No clinically significant changes were observed in glucose, HbA1c, liver and kidney function tests, or hemogram parameters.

**Conclusion:** In this real-world cohort, Upadacitinib demonstrated a favorable safety profile with no serious cardiovascular or thromboembolic events. Although the drug led to an increase in cholesterol levels, the stability of the LDL-C/HDL-C ratio suggests that this change may not translate to an increased cardiovascular risk.

## SS-52 Persistence of Biologic Therapy in Inflammatory Bowel Disease: Single-Center Experience

**Beyza Atay, Bengi Öztürk, Muhammed Furkan Çakmak, Taylan Kav**

Department of Gastroenterology, Hacettepe University Medical Faculty, Ankara, Türkiye

**Background/Aims: **Ulcerative colitis (UC) and Crohn’s disease (CD) are chronic inflammatory bowel diseases characterized by relapsing and remitting courses. Biologic agents constitute a cornerstone of treatment, and treatment persistence—defined as the continuation of therapeutic response—is crucial for clinical outcomes. This study aimed to evaluate the persistence and usage patterns of biologic therapies in inflammatory bowel disease using real-world, single-center data.

**Materials and Methods:** Demographic data, type of biologic agent, treatment sequence, duration of therapy, and reasons for discontinuation were retrospectively collected from the hospital records of patients with inflammatory bowel disease receiving biologic therapy at the Department of Gastroenterology, Hacettepe University.

**Results:** A total of 242 patients receiving biologic agents were included in the study; 122 were male (50.4%) and 120 were female (49.6%). Among them, 154 patients (63.6%) were diagnosed with Crohn’s disease (CD), and 88 patients (36.4%) with ulcerative colitis (UC). In the CD group, disease behavior was classified as inflammatory in 81 patients (52.6%), structuring in 44 patients (28.6%), penetrating in 16 patients (10.4%), and both structuring and penetrating in 8 patients (5.2%).

Median treatment durations varied between agents and ranged from 14 to 47 months in both CD and UC patients. The median persistence durations of adalimumab, infliximab, ustekinumab, and vedolizumab were calculated as 47/14 months, 31/21 months, 15.5/10 months, and 36/8.5 months in CD and UC, respectively. The most common reason for treatment discontinuation was identified as secondary loss of response.

**Conclusion:** Real-world data indicate that biologic agents can be used sequentially in inflammatory bowel disease, and loss of response to 1 agent can often be addressed with another. Persistence with biologic therapy varies according to disease type and treatment line, but median durations typically span several years, demonstrating the long-term efficacy and sustainability of biologic treatments.

## SS-54 SBP-RISK-C1000: A New, Simple, and Powerful Bedside Score for Predicting the Risk of Spontaneous Bacterial Peritonitis in Cirrhotic Patients

**Yavuz Özden**

Department of Gastroenterology, Kayseri City Hospital, Kayseri, Türkiye

**Background/Aims:** Spontaneous bacterial peritonitis (SBP) is a severe infection in cirrhotic patients with ascites, associated with high mortality and the need for early recognition. Because symptoms are often subtle, diagnostic delays negatively affect outcomes. Existing risk scores are based on limited variables, lack modern inflammatory biomarkers, and show suboptimal accuracy in clinical practice. This study aimed to develop a simple, high-performing bedside score (SBP-RISK-C1000) using routine clinical and laboratory parameters to predict the risk of SBP at presentation.

**Materials and Methods:** From June 2020 to June 2025, 1000 hospitalized cirrhotic patients with ascites who underwent diagnostic paracentesis within 24 hours were retrospectively analyzed (derivation: n = 700, validation: n = 300). Candidate variables were tested using multivariable logistic regression, and significant predictors were assigned integer points to create the SBP-RISK-C1000 score. Model discrimination was assessed by the area under the ROC curve (AUROC), calibration by the Hosmer–Lemeshow test, and performance was compared with the Mansoura and Wehmeyer models. Clinical benefit was evaluated using decision curve analysis and the net reclassification improvement (NRI) index.

**Results:** SBP prevalence was 18.5%. Four independent predictors were identified: C-reactive protein >50 mg/L (2 points), neutrophil-to-lymphocyte ratio >4 (2 points), serum sodium <130 mmol/L (1 point), and age >60 years (1 point). The total score (0-6) correlated strongly with SBP probability. AUROC values were 0.92 in the derivation cohort and 0.91 in the validation cohort. A cutoff of ≥3 predicted SBP with 88.1% sensitivity and 91.7% specificity. SBP-RISK-C1000 outperformed existing models (*P* < .01) and showed the highest net benefit.

**Conclusion:** SBP-RISK-C1000 is a practical, transparent, and accurate bedside score based on 4 routine parameters. It facilitates early SBP recognition, guiding the prioritization of paracentesis, timely empirical antibiotic use, and selective prophylaxis. With its simplicity, broad usability, and strong internal validation, SBP-RISK-C1000 may set a new standard in SBP risk prediction. Prospective multicenter validation is warranted.

Table 1.SBP-RISK-C1000 Scoring System (Components, Thresholds, and Points)

A simple bedside score derived from 4 clinical and laboratory parameters. The total score ranges from 0 to 6, with higher scores indicating a higher probability of SBP.

CRP, C-reactive protein; NLR, neutrophil-to-lymphocyte ratio; SBP, spontaneous bacterial peritonitis.

Table 2.Key Performance Metrics of SBP-RISK-C1000 and Comparative Results

AUROC, area under the receiver operating characteristic curve; SBP, spontaneous bacterial peritonitis.

**Table:** 

Component	Threshold	Points
C-reactive protein	>50 mg/L	2
Neutrophil-to-lymphocyte ratio	>4	2
Serum sodium	<130 mmol/L	1
Age (years)	>60 years	1

**Table:** 

Parameter	Result
SBP prevalence	18.5%
Derivation cohort AUROC	0.92
Validation cohort AUROC	0.91
Cutoff for predicting SBP	≥3
Sensitivity at cutoff ≥3	88.1%
Specificity at cutoff ≥3	91.7%
Comparison with existing models	SBP-RISK-C1000 outperformed the Mansoura and Wehmeyer models
Comparative significance	*P* < .01
Net benefit	Highest among the evaluated models

## SS-56 Endocan and Endoglin as Fibrosis Biomarkers in Metabolic Dysfunction-Associated Steatotic Liver Disease: A Cross-Sectional Study

**Doğan Can Gavcar ^1^ , Mehmet Akca ^1^ , Hüseyin Döngelli ^1^ , Mine Arayıcı ^4^ , Nevin Deniz Kırca ^4^ , Servet Kızıldağ ^3^ , Nilay Danış ^2^ , Mesut Akarsu ^2^**

^1^Department of Internal Medicine, Dokuz Eylül University Hospital, İzmir, Türkiye

^2^Department of Gastroenterology, Dokuz Eylül University Hospital, İzmir, Türkiye

^3^Department of Vocational School of Health Sciences, Dokuz Eylül University Hospital, İzmir, Türkiye

^4^Department of Neuroscience, Institute of Health Sciences, Dokuz Eylül University, İzmir, Türkiye

**Background/Aims:** Metabolic dysfunction-associated steatotic liver disease (MASLD) is the most prevalent chronic liver disorder worldwide. Thus, this study aimed to determine the diagnostic utility of serum endocan and endoglin in identifying MASLD and distinguishing the severity of fibrosis.

**Materials and Methods:** In this study, conducted between December 2023 and November 2024, 58 patients with MASLD and 30 healthy controls were enrolled; the sample size was based on a power analysis. Vibration-controlled transient elastography, which reports controlled attenuation parameters and liver stiffness in kilopascals, was used to quantify hepatic steatosis and fibrosis, and serum levels of endocan and endoglin were measured. Comparisons were made between the MASLD and control groups, as well as within the MASLD group between patients with advanced-stage fibrosis and those with lower-stage fibrosis. The diagnostic power of the biomarkers was evaluated using receiver operating characteristic (ROC) analysis. Independent associations between serum biomarkers and kilopascals values in the patient group were assessed through regression analysis.

**Results:** Serum endocan and endoglin levels were significantly increased in the MASLD group compared with controls. In the MASLD group, patients with advanced fibrosis exhibited higher levels of endocan and endoglin than those with lower-stage fibrosis. In ROC analysis for discriminating advanced fibrosis, endoglin demonstrated strong diagnostic performance, whereas endocan exhibited good diagnostic accuracy. Multivariate regression analysis revealed that log-transformed kilopascals values were independently associated with serum endocan and endoglin levels.

**Conclusion:** Endocan and endoglin are promising biomarkers for diagnosing MASLD and for noninvasively predicting the stage of fibrosis in these patients.

## SS-57 The Association of Novel Defined Lipid Indices with MASLD and Liver Fibrosis Among Patients in Türkiye; A National, Multicenter, Cross-Sectional Study

**İhsan Solmaz ^1^ , Ali Kırık ^2^ , İsmail Demir ^3^ , Oğuzhan Sıtkı Dizdar ^4^ , Nevzat Gözel ^5^ , Gökhan Tazegül ^6^ , Bilgin Bahadır Başgöz ^7^ , Cem Şahin ^8^ , Kubilay İşsever ^9^ , Gülali Aktaş ^10^ , Pınar Yıldız ^11^ , Emin Gemcioğlu ^12^ , Enes Şahiner ^13^ , Hilal Bektaş Uysal ^14^ , Yasin Şahintürk ^15^ , DAHUDER MASLD Study Group ^16^**

^1^Department of Internal Medicine, Diyarbakır Gazi Yaşargil Training and Research Hospital, Diyarbakır, Türkiye

^2^Department of Internal Medicine, Balıkesir University Medical School, Balıkesir, Türkiye

^3^Department of Internal Medicine, Bozyaka Training and Research Hospital, İzmir, Türkiye

^4^Department of Internal Medicine, Kayseri City Hospital, Kayseri, Türkiye

^5^Department of Internal Medicine, Firat University Faculty of Medicine, Elazığ, Türkiye

^6^Department of Internal Medicine, Marmara University Pendik Training and Research Hospital, İstanbul, Türkiye

^7^Department of Internal Medicine, University of Health Sciences, Antalya City Training and Research Hospital, Antalya, Türkiye

^8^Department of Internal Medicine, Muğla Sıtkı Koçman University Faculty of Medicine, Muğla, Türkiye

^9^Department of Internal Medicine, Giresun University Faculty of Medicine, Giresun, Türkiye

^10^Department of Internal Medicine, Bolu Abant İzzet Baysal University Hospital, Bolu, Türkiye

^11^Department of Internal Medicine, Eskisehir Osmangazi University Faculty of Medicine, Eskişehir, Türkiye

^12^Department of Internal Medicine, University of Health Sciences, Ankara Etlik City Training and Research Hospital, Ankara, Türkiye

^13^Department of Internal Medicine, Ankara Bilkent City Hospital, Ankara, Türkiye

^14^Department of Internal Medicine, Aydın Adnan Menderes University Faculty of Medicine, Aydın, Türkiye

^15^Department of Internal Medicine, Antalya Training and Research Hospital, Antalya, Türkiye

^16^DAHUDER MASLD Study Group, Türkiye

**Background/Aims:** Monocyte-to-HDL cholesterol ratio (MHR), Visceral Adiposity Index (VAI), Plasma Atherogenic Index (PAI), and Cardiometabolic Index (CMI) are considered biomarkers for the diagnosis and risk assessment of inflammatory and metabolic diseases. This study aimed to investigate the roles of MHR, VAI, AIP, and CMI indices in determining hepatic inflammation and fibrosis in patients with MASLD across Türkiye.

**Materials and Methods:** This retrospective, cross-sectional national study was conducted with individuals who had at least 1 cardiometabolic risk factor from 44 internal medicine clinics in 31 provinces representing all statistical regions of Türkiye. Demographic characteristics, comorbidities, and laboratory parameters of participants were recorded. MHR, VAI, AIP, CMI indices, and FIB-4 scores were calculated.

**Results:** A total of 14 322 patients were included, and baseline characteristics, comorbid conditions, and index scores are given in Table 1. Of these patients, 10 836 (75.66%) were diagnosed with MASLD, and VAI, PAI, and CMI were significantly higher in individuals with MASLD, whereas MHR did not show a significant difference. Among patients with MASLD, 1214 (11.2%) were in the high FIB-4 score group. All indices were found to be similar between high and low FIB-4 score groups (*P* > .05, Table 2).

**Conclusion:** The results of this study briefly revealed that although VAI, PAI, and CMI were significantly higher in patients with MASLD, no such association was observed between FIB-4 score and lipid indices. The results indicate that although novel lipid indices can be used in MASLD screening and risk assessment, these indices have no role in predicting liver fibrosis.

## SS-59 What is the Importance of Young Cases in Colorectal Cancer Screening in the Country?

**Levent Erdem ^1^ , Colorectal Cancer and Polyp Study Group ^2^**

^1^Department of Gastroenterology, Demiroğlu Bilim University, Florence Nightingale Hospital, İstanbul, Türkiye

^2^Colorectal Cancer and Polyp Study Group, Türkiye

**Background/Aims:** The importance of colorectal cancer precursor polyps is well recognized. As a working group, this is among the first centers in the world to report that the age for colorectal screening should be reduced to 45 years. In recent years, publications have shown an increase in colorectal cancer diagnoses at younger ages. In some countries, colorectal cancer is reported as the most common cancer in young men. Screening reduces incidence and mortality. The first aim of this study was to evaluate the colorectal cancer screening data for young patients in terms of polyp and cancer risk, and the second aim was to emphasize the importance of the subject.

**Materials and Methods:** The study data were obtained from the colorectal screening studies conducted between 2015 and 2025. The first study was conducted during the 2015-2017 period with 16 centers. The second study was conducted during the 2018-2022 period with 12 centers. The third study is ongoing from 2023 to 2025. Demographic characteristics, family history, smoking habits, alcohol consumption, BMI (kg/m^2^), polyp detection rates, location, polyp histological types, colon cancer incidence, location, and types were recorded in all studies. The risk score that was developed was calculated for all young cases (age, gender, smoking, BMI, family history).

**Results:** A total of 9105 screening cases meeting the study criteria were evaluated. The first study included 6508 cases (all adult ages), the second study had 2217 cases (under 50, ages 18-49), and the third study included 380 cases (also under 50, ages 18-49). In all young cases, the adenoma detection rate was 23%, and the colorectal cancer rate was 1.3%. In young patients with high-risk scores, the rates of adenoma (45%) and cancer (2.6%) were significantly higher.

**Conclusion:** The risk of adenoma and colorectal cancer is higher in patients under 50 years of age, especially those with high-risk scores. Therefore, this age group should be carefully evaluated for colorectal cancer screening.

## SS-60 Functional Gastrointestinal Disorders and Screen Time: Effects of Modern Life

**Beril Demir, Halit Kandemir, Enes Cömert, Derya Kirman, Fatih Acehan, Yusufcan Yılmaz, Kenan Moral, Güner Kılıç, Ali Karataş, Gülden Bilican, Yunus Emre Börü, Murat Kekilli, Tarkan Karakan, Mehmet Cindoruk**

Division of Gastroenterology, Department of Internal Medicine, Gazi University Medical Faculty, Ankara, Türkiye

Screen exposure is increasing in modern life, and its relationship with functional gastrointestinal disorders remains uncertain. In this cross-sectional, survey-based study conducted at the Gastroenterology Outpatient Clinic of Gazi University Faculty of Medicine Hospital between June 27, 2025 and October 1, 2025, the association between daily screen time and functional bowel disorders among adults aged 18-80 years was evaluated. Diagnoses of IBS, functional constipation, functional diarrhea, and functional abdominal bloating/distension were determined using Rome IV criteria, and the primary outcome was the presence of IBS. Individuals who declined participation or had inflammatory bowel disease, malignancy, or celiac disease were excluded. Of 813 participants, 812 were analyzed. Mean daily screen time was 6.03 ± 2.75 hours/day (median 6). No significant association was found between screen time in hours and IBS (*P* = .099). In sensitivity analysis, log-transformed screen time showed a small, borderline-significant association with IBS (OR = 1.35, *P* = .045). The difference in group means was −0.35 hours, with a two-sided *P* = .098. Time spent on the toilet and phone use while on the toilet were strongly associated with IBS (both *P* < .001; linear trend *P* < .001). Female sex was more frequent among IBS cases (*P* = .007). TV and computer viewing time were not associated with IBS (*P* ≥ .087 and *P* = .575). In conclusion, the primary analysis did not demonstrate a clinically meaningful association between daily screen time and IBS. The modest and borderline statistical signal after log transformation suggests a weak effect and sensitivity to measurement scale. The findings indicate that any potential effect of screen exposure on IBS may arise indirectly through toileting-related behaviors.

## SS-61 Anorectal Manometry in Anorectal and Pelvic Floor Disorders: A Single-Center, Single-Operator Experience

**Özdal Ersoy**

Department of Internal Medicine, Arel University Medical Faculty, Memorial Bahçelievler Hospital, İstanbul, Türkiye

**Background/Aims:** Anorectal manometry (ARM) is an important diagnostic tool for evaluating anorectal and pelvic floor disorders. However, real-world data and large series comparing the contributions of high-resolution anorectal manometry (HRAM) with conventional ARM remain limited in Türkiye. This study aimed to present the diagnostic spectrum, technical yield, and clinical impact of ARM procedures performed by a single operator in a tertiary pelvic floor center.

**Materials and Methods:** A retrospective review was conducted of consecutive patients who underwent ARM between January 2013 and May 2025 in the gastroenterology/anorectal disorders departments of Acıbadem Healthcare Group hospitals. Techniques used included conventional ARM from 2013 until September 2022, followed by HRAM thereafter. Main indications included chronic constipation, anal/urinary incontinence, low anterior resection syndrome (LARS), chronic pelvic pain, stoma follow-up, and pelvic organ prolapse. Analyzed parameters included resting and squeeze sphincter pressures, the recto-anal inhibitory reflex (RAIR), rectal sensory thresholds, defecation patterns, the cough reflex, and the balloon expulsion test. Demographic and clinical data were obtained from electronic medical records.

**Results:** A total of 690 procedures were performed: 480 (69%) were conventional ARM, and 210 (31%) were HRAM. The mean age was 48.5 ± 32.5 years, with a female predominance of 68%. The main indications were chronic constipation (42%), LARS (21%), anal incontinence (16%), urinary incontinence (3%), stoma assessment (10%), and pelvic organ prolapse (8%).Absent RAIR was observed in 12% of patients (including cases of Hirschsprung’s disease). HRAM significantly improved the detection of defecatory disorders in patients with LARS and constipation (41% vs. 28%; *P* < .01) and provided topographic pressure mapping that influenced specific therapeutic decisions.

**Conclusion:** This large experience highlights the indispensable role of ARM in diagnosing pelvic floor disorders. Moreover, ARM proved valuable not only in classical dyssynergic defecation but also in detecting U-wave patterns, hypertonic sphincters, and pressure abnormalities associated with solitary rectal ulcer, thereby enhancing the diagnostic yield in LARS and guiding individualized treatment strategies. These findings highlight the need to expand ARM and strengthen standardized reporting.

## SS-62 Comparative Evaluation of the Prognostic Performance of ABC, MAP(ASH), H3B2, Glasgow–Blatchford, and AIMS65 Scores in Acute Upper Gastrointestinal Bleeding

**Yavuz Özden, Ömer Yüzügülen, Nuh Mehmet Büyükberber**

Department of Gastroenterology, Kayseri City Hospital, Kayseri, Türkiye

**Background/Aims:** Acute upper gastrointestinal bleeding (AUGIB) remains a critical emergency with a mortality rate of 7%-10%. Early risk stratification is essential for predicting mortality, rebleeding, and the need for therapeutic intervention. This study aimed to directly compare the prognostic performance and clinical applicability of 5 scoring systems—ABC, MAP(ASH), H3B2, Glasgow–Blatchford (GBS), and AIMS65—in a large patient cohort.

**Materials and Methods:** A total of 2000 adult patients were retrospectively analyzed with endoscopically confirmed AUGIB admitted between November 2023 and November 2024. All scores were calculated using clinical and laboratory data at presentation. The primary outcome was 30-day all-cause mortality; secondary outcomes included therapeutic intervention and 30-day rebleeding. Discriminative performance was assessed by receiver operating characteristic (ROC) curve analysis, and predefined cutoff values from the literature were applied to identify low-risk groups.

**Results:** The ABC score showed the highest discriminatory power for mortality (AUROC 0.83), followed by MAP(ASH) (0.81) and H3B2 (0.78). GBS (0.70) and AIMS65 (0.75) demonstrated lower accuracy. For predicting the need for intervention, GBS (0.78) and MAP(ASH) (0.77) performed best. All scores exhibited limited predictive ability for rebleeding (AUROC 0.60-0.66). At low-risk thresholds, ABC ≤ 3 and MAP(ASH) ≤2 identified approximately 40% of patients with a mortality rate below 1%. GBS ≤1 and AIMS65 = 0 classified 15% and 10% of patients, respectively, as low risk. These findings highlight the superior discriminative ability of ABC and MAP(ASH) for both mortality and intervention outcomes.

**Conclusion:** ABC and MAP(ASH) demonstrated the strongest prognostic performance in AUGIB. H3B2 was useful for hemodynamic assessment but limited in mortality prediction. GBS remained reliable for identifying low-risk patients. This large single-center analysis—the most extensive national series to date—suggests that combining ABC and MAP(ASH) may offer an effective strategy for early discharge, shorter hospital stay, and optimal resource utilization.

Table 1.Area Under the Receiver Operating Characteristic Curve Values Reported in the Abstract for Predicting 30-Day Mortality, Need for Intervention, and 30-Day Rebleeding in Acute Upper Gastrointestinal Bleeding

Score names: ABC, age, blood tests, and comorbidities; MAP(ASH), altered mental status, ASA score, pulse, albumin, systolic blood pressure, and hemoglobin; H3B2, hematemesis, heart rate, systolic blood pressure, hemoglobin, and blood urea nitrogen; AIMS65, albumin <3.0 g/dL, INR >1.5, altered mental status, systolic blood pressure ≤90 mmHg, and age ≥65 years.

AUGIB, acute upper gastrointestinal bleeding; AUROC, area under the receiver operating characteristic curve; GBS, Glasgow–Blatchford score.


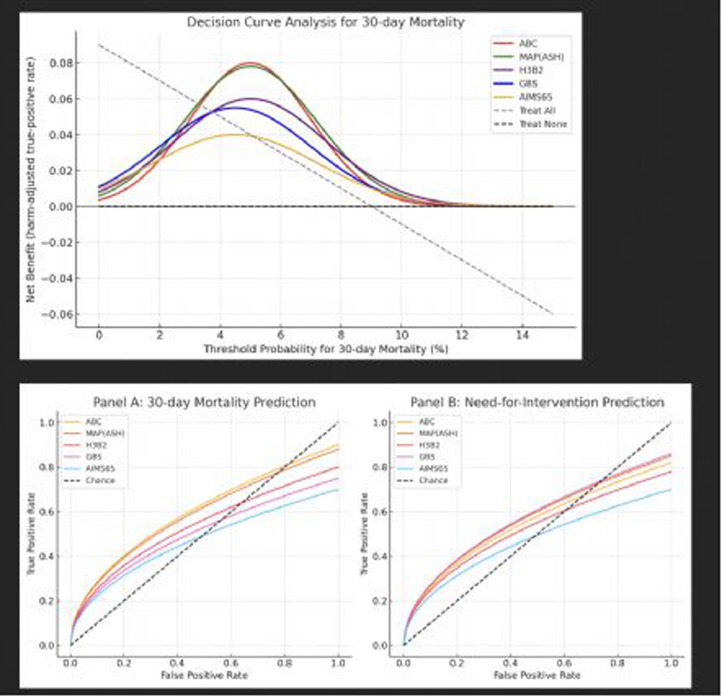


Figure 1. Receiver operating characteristic (ROC) curves and decision curve analysis comparing the prognostic performance of ABC, MAP(ASH), H3B2, Glasgow–Blatchford, and AIMS65 scores for predicting 30-day mortality and the need for intervention in acute.

**Table:** 

Outcome	Score	AUROC
30-day mortality	ABC	0.83
30-day mortality	MAP(ASH)	0.81
30-day mortality	H3B2	0.78
30-day mortality	AIMS65	0.75
30-day mortality	Glasgow–Blatchford	0.70
Need for intervention	Glasgow–Blatchford	0.78
Need for intervention	MAP(ASH)	0.77
30-day rebleeding	All scores	0.60-0.66

## SS-64 Determination of Points for Diagnosis and Treatment in Gastric Cancers by in Silico Methods

**Rauf Mehtiyev ^1^ , Ender Berat Ellidokuz ^2^ , Asim Leblebici ^3^ , Zerrin Isik ^4^**

^1^Department of Gastroenterology, Dokuz Eylül University Faculty of Medicine, İzmir, Türkiye

^2^Large Biotechnology İnformatics Health Joint Stock Company, İzmir, Türkiye

^3^Department of Information Technologies, İzmir İnstitute of Technology, İzmir, Türkiye

^4^Department of Computer Science, Vrije Universiteit, Amsterdam, Holland

**Background/Aims: **Many patients with gastric cancer (GC) are diagnosed at advanced stages, missing the best treatment opportunity. This study aimed to elucidate the molecular mechanisms of GC and identify specific biomarkers for its prognosis, early diagnosis, and targeted therapy.

**Materials and Methods:** Ten mRNA datasets (GSE79973, GSE34942, GSE38749, GSE35809, GSE22377, GSE66222, GSE54129, GSE42252, GSE19826, and GSE13911) were selected from the Gene Expression Omnibus (GEO)database. The dataset comprises 177 normal gastric tissue samples and 360 gastric adenocarcinoma samples, totaling 537 samples. Differentially expressed genes (DEGs) were selected using R software. R software and volcano plots were utilized to identify differentially expressed genes. Subsequently, Gene Ontology (GO)analysis and Kyoto Encyclopedia of Genes and Genomes (KEGG) pathway analysis were performed. STRING and Cytoscape software were also used to analyze protein-protein interaction (PPI) networks of DEGs common among the 10 datasets.

**Results:** A total of 520 DEGs were identified, consisting of 260 upregulated and 260 downregulated genes. Using R software and volcano plots, DEGs with log FC > 2 were identified among the 520 DEGs. KEGG analysis showed that the upregulated DEGs were primarily enriched in protein digestion and absorption, ECM-receptor interaction, drug metabolism, IL-17 signaling pathway, and cytokine-cytokine receptor interaction. GO analysis indicated that the upregulated DEGs were mainly enriched in extracellular matrix organization, extracellular structure organization, external encapsulating structure organization, regulation of cell population proliferation, and collagen fibril organization. The STRING database and Cytoscape software were used to predict and analyze protein interactions among 83 DEGs, 16 of which were upregulated (log FC > 2) and 67 downregulated (log FC < 2). By combining the STRING and Cytoscape analysis results, 10 DEGs (INHBA, SULF1, COL1A2, CTHRC1, THBS2, COL1A1, COL11A1, SFRP4, FN1, and COL10A1) were selected. These DEGs are upregulated, have a high degree of association, log FC > 2, and the highest FcS.

**Conclusion:** This study used bioinformatics analysis methods to identify 10 DEGs, including INHBA, SULF1, COL1A2, CTHRC1, THBS2, COL1A1, COL11A1, SFRP4, FN1, and COL10A1, which were significantly overexpressed in patients with gastric cancer. These findings suggest that these 10 DEGs may serve as potential biomarkers and therapeutic targets for GC.


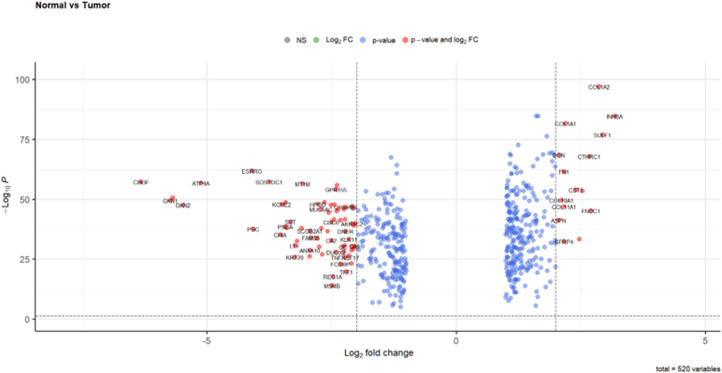


Figure 1. Using the volcano plot, upregulated (log FC > 2) and downregulated (log FC < 2) DEGs were identified out of 520 DEGs. Thirteen DEGs with log FC > 2 were detected: INHBA, SULF1, COL1A2, FNDC1, CTHRC1, CST1, COL1A1, COL11A1, SFRP4, FN1, COL10A1, BGN, and ASPN.


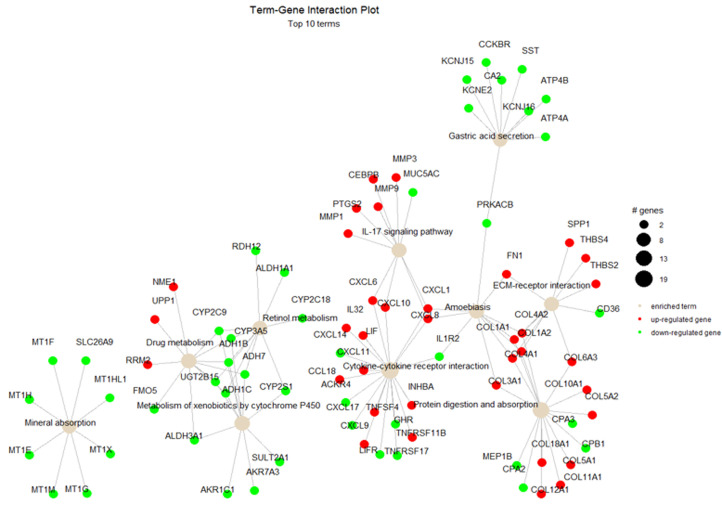


Figure 2. Term-gene interaction plot in KEGG pathways. Genes were enriched in KEGG pathways including protein digestion and absorption, ECM-receptor interaction, drug metabolism, IL-17 signaling pathway, cytokine-cytokine receptor interaction, and amoebiasis pathways. These pathways frequently contained upregulated DEGs.


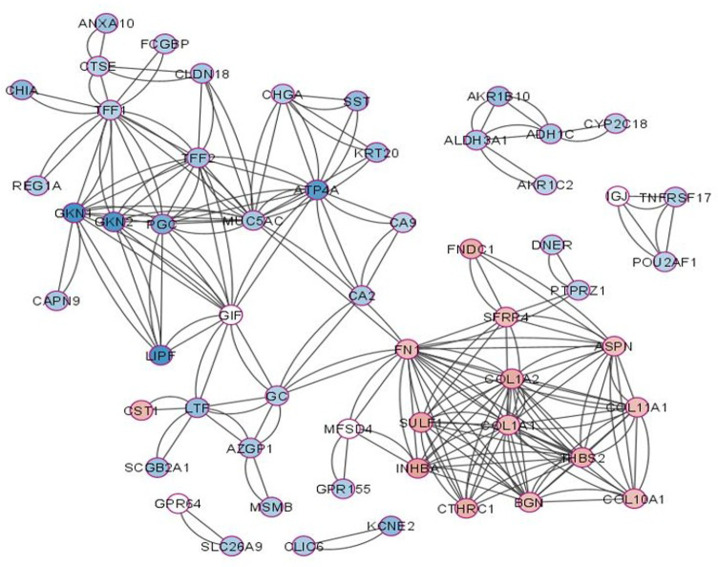


Figure 3. The PPI network of DEGs was constructed using Cytoscape. Red nodes represent upregulated genes, whereas blue nodes represent downregulated genes. Fourteen upregulated genes were obtained. Among these, 10 DEGs with high interaction (log FC > 2) were identified: INHBA, SULF1, COL1A2, CTHRC1, THBS2, COL1A1, COL11A1, SFRP4, FN1, and COL10A1.

## SS-65 Best Treatment Options for Severe Helicobacter pylori Infections

**Gülhan Kanat Ünler ^1^ , Hilal Erinanç ^1^ , Aydın Karakoca ^2^ , Hüseyin Savaş Göktürk ^1^**

^1^Başkent University Faculty of Medicine, Ankara, Türkiye

^2^Necmettin Erbakan University Faculty of Science Statistics, Konya, Türkiye

**Background/Aims:** *Helicobacter pylori* affects half of the world’s population. Increasing antibiotic resistance appears to be causing significant clinical problems. This study investigated the efficacy of bismuth-containing sequential therapy was with clarithromycin (BSTC), bismuth-containing sequential therapy with levofloxacin (BSTL), and bismuth-containing quadruple therapy (BQT) regimens on *H. pylori* eradication. The study also examined whether high gastric *H. pylori* colonization density impacted treatment success across different regimens.

**Materials and Methods:** A total of 751 *H. pylori*-positive patients were included retrospectively in the following treatment groups: sequential therapy with clarithromycin, sequential therapy with levofloxacin, and bismuth-containing quadruple therapy.

**Results:** There was a significant difference between the 3 treatment protocols regarding treatment success rates. When the success rates of the treatments were analyzed, the highest success rate was observed for BSTL (85.3%), which was statistically significantly higher compared to both BQT (74.8%) and BSTC (74.8%). A significant difference was found between the success rates of the protocols in the group with high bacterial density (*P* = .003). The success rates in this group were calculated as BSTL (88.6%), BQT (71.4%), and BSTC (79.4%).

**Conclusion:** It was concluded that BSTL may be the better option for treating *H. pylori* infections as a first-line treatment. This regimen is particularly effective in cases of severe *H. pylori* colonization.

## SS-66 Preliminary Insights into Escherichia coli and Helicobacter pylori Pathogenicity Among Turkish Patients with Gastrointestinal Cancer

**Tayyip Karaman ^1^ , Nesteren Mansur ^1^ , Arzu Tiftikçi ^2^ , Suna Yapalı ^2^ , Cem Aygün ^2^ , Osman Uğur Sezerman ^3^ , Tanıl Kocagöz ^1^ , Nurdan Tözün ^2^ , Sinem Oktem Okullu ^4^**

^1^Department of Medical Biotechnology, Acıbadem Mehmet Ali Aydınlar University Institute of Health Science, İstanbul, Türkiye

^2^Department of Gastroenterology, Acıbadem Mehmet Ali Aydınlar University School of Medicine, İstanbul, Türkiye

^3^Department of Biostatistics and Medical Informatics, Acıbadem Mehmet Ali Aydınlar School of Medicine, İstanbul, Türkiye

^4^Department of Medical Microbiology, Acıbadem Mehmet Ali Aydınlar University School of Medicine, İstanbul, Türkiye

**Background/Aims:** Gastrointestinal cancers are among the most common malignancies in Türkiye, with gastric and colorectal cancers showing particularly high prevalence. Microbial factors such as *Helicobacter pylori* and colibactin-producing *Escherichia coli* strains have increasingly been recognized as important contributors to gastric and colon carcinogenesis. In this study, the prevalence and virulence gene profiles of *H. pylori* in patients with gastric cancer was investigated, and the occurrence of colibactin-associated (clb) genes and pks islands in patients with colorectal cancer was evaluated.

**Materials and Methods:** Tissue samples from 4 patients with gastric cancer and 9 healthy individuals were analyzed using a multiplex PCR approach. For colorectal cancer, 19 tumor samples and healthy controls were screened for clb genes, and whole genome sequencing (WGS) was performed on cultured isolates to further characterize colibactin-producing strains.

**Results:** Results showed that 1 patient with gastric cancer was negative for all *H. pylori* virulence genes, whereas 1 healthy control carried the complete virulent gene set, consistent with asymptomatic carriage of pathogenic strains. This highlights the multifactorial etiology of gastric cancer, where alternative drivers such as host genetics, viral oncogenesis, or other microbial taxa may contribute. In colorectal cancer, clb gene positivity was detected in 4 of 19 patients, with 3 harboring the complete clbA, clbB, clbN, and clbQ gene set. WGS identified *E. coli Nissle*, a widely used probiotic, carrying the pks island, suggesting a potentially underestimated pathogenic capacity. In contrast, *Citrobacter braakii* was isolated but lacked clb genes, showing only fimbrial and biofilm-associated clusters.

**Conclusion:** Overall, these findings suggest that *H. pylori* remains an important but nonexclusive factor in gastric cancer, whereas pks-positive *E. coli* strains, including *E. coli Nissle*, may play underappreciated roles in colorectal cancer progression. The unexpected detection of *C. braakii* further underscores the complexity of the tumor-associated microbiome, emphasizing the need for broader multiomics approaches and reevaluation of microbial biomarkers in gastrointestinal cancer pathogenesis.


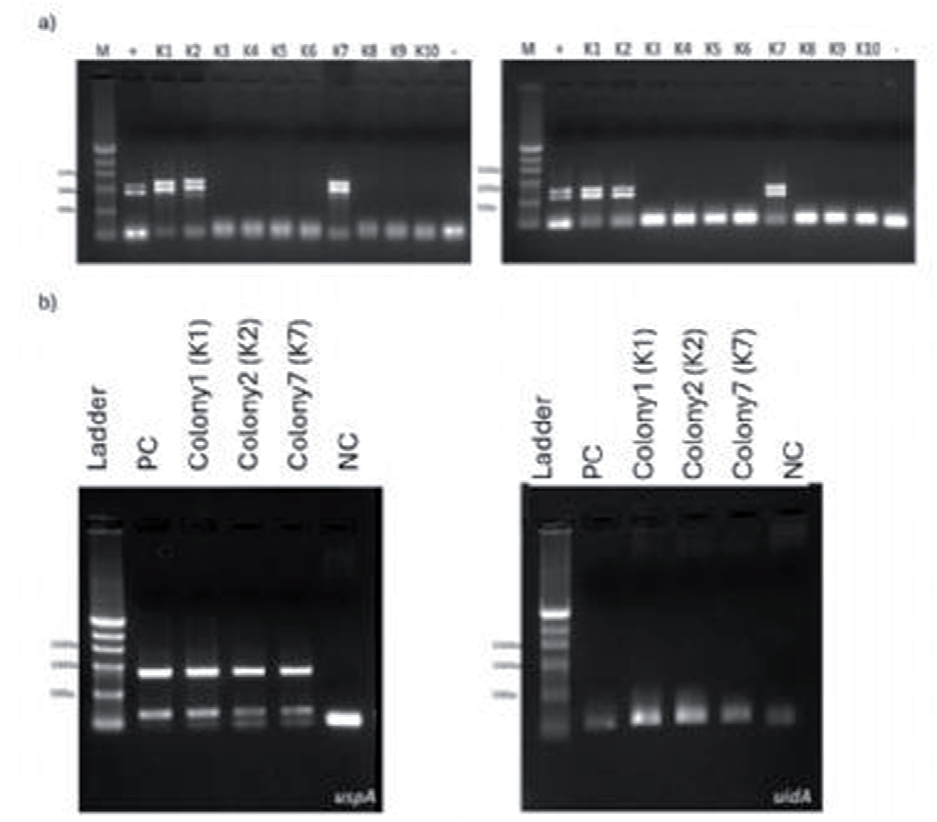


Figure 1. Gel electrophoresis images of colibactin PCR products from bacterial colonies.

Tumor tissue samples positive for clb genes (CC1, CC10, and CC19) were cultured, and single colonies were isolated. The purpose of isolating single colonies was to verify the presence of clb-positive *E. coli* and to prepare them for whole genome sequencing to determine strain identity. DNA was extracted from 10 cultured bacterial colonies, and PCR was performed to detect the uspA, uidA, clbA, clbB, clbN, and clbQ genes. Among these, 3 colonies (K1, K2, and K7) were positive for the complete set of clb genes. A subsequent PCR analysis targeting uspA and uidA confirmed that colonies 1, 2, and 7 were also positive for these 2 *E. coli* marker genes. These results demonstrate that the colonies obtained from tumor tissues of patients with colorectal cancer represent colibactin-producing *E. coli* strains.


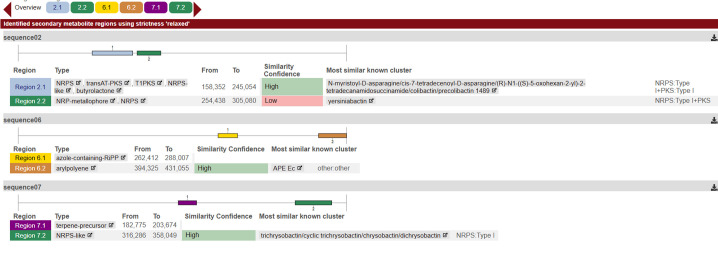


Figure 2. Secondary metabolite analysis.

To further characterize the clb genes detected by PCR, the genome sequence was analyzed. Hypothetical proteins were extracted from the GenBank file and compiled into a separate FASTA dataset. Secondary metabolite prediction using antiSMASH revealed a PKS island with high-confidence similarity. BLAST analysis further confirmed the presence of multiple clb genes within the colibactin (pks) genomic island of *E. coli* Nissle. Strong matches were obtained for clbA, clbB, clbC, clbF, clbG, clbK, clbM, clbP, clbQ, clbS, clnO, and clbN, all aligning to the same large genomic contig (contig_12). Sequence identities ranged from 96% to 100%, with alignments spanning nearly the full length of each gene, indicating highly conserved homologs across the cluster.

## SS-70 De Ritis Ratio as a Predictor of Lymph Node Involvement in Gastric Cancer

**Yunus Halil Polat, Ahmet Yozgat**

Department of Gastroenterology, Health Sciences University Ankara Training and Research Hospital, Ankara, Türkiye

**Background/Aims:** Early-stage gastric cancer (EGC) is defined as a carcinoma limited to the gastric mucosa and/or submucosa, regardless of lymph node status. Endoscopic submucosal dissection is recommended as the first-line treatment option for superficial gastric lesions without submucosal invasion. Different levels of aspartate aminotransferase (AST) and alanine aminotransferase (ALT) have been reported as prognostic markers in several cancer types. However, there are not enough studies on the use of the De Ritis ratio (DRR) (AST/ALT ratio) in gastric cancer. The aim of this study was to evaluate the association of DRR with lymph node involvement, an important marker of survival in EGC and advanced gastric cancer.

**Materials and** **Methods:** Two hundred and thirty patients with gastric adenocarcinoma treated with gastrectomy and lymph node dissection were retrospectively analyzed in the study.

**Results:** In the lymph node positive group, the mean tumor diameter was larger than in the lymph node negative group (6.3 ± 2.8 vs. 4.12 ± 2 cm, *P* < .001). According to the T-stage distribution, in the lymph node positive group, 88 (50.3%) patients were in T3-T4 stage, whereas in the lymph node negative group, only 10 (18.2%) patients were in T3-T4 stage (*P* < .001). The median AST/ALT ratio was 1.38 (0.73-12) in the lymph node positive group, whereas it was 1.2 (0.47-1.89) in the lymph node negative group (*P* < .001). Patients with a higher AST/ALT ratio showed worse pathological profiles and correlated with T-stage, N-stage, tumor diameter, and lymphovascular invasion (rs = 0.200, *P* = .002; rs = 0.173, *P* = .008; rs = 0.218, *P* = .001; rs = 0.144, *P =* .030, respectively).

**Conclusion:** The optimum AST/ALT ratio cutoff point for determining lymph node involvement was 1.29. A high AST/ALT ratio indicates worse pathological profiles and can determine lymph node involvement in patients with gastric cancer. Since lymph node involvement is a key prognostic indicator in EGC and AGC, these findings may have a potential prognostic impact on selecting which patients undergo ESD and gastrectomy.

## SS-71 Bismuth-Based Quintuple Therapy: A Step Beyond Quadruple Regimens in H. Pylori Treatment and the Impact of Patient Compliance

**Ramazan Dertli ^1^ , Mehmet Asıl ^1^ , Yahya Atayan ^2^ , Ramazan Yolaçan ^3^ , Uğurcan Coşar ^1^ , Murat Bıyık ^1^ , Muharrem Keskin ^1^ , Gürkan Şahinoğlu ^1^ , Ali Demir ^1^**

^1^Department of Internal Medicine, Necmettin Erbakan University Faculty of Medicine, Department of Gastroenterology, Konya

^2^Division of Gastroenterology, Department of Internal Medicine, İnönü University School of Medicine, Malatya, Türkiye

^3^Department of Gastroenterology, Gaziantep City Hospital, Gaziantep, Türkiye

**Background/Aims: ***Helicobacter pylori* (*H. pylori*) infection is one of the most common infections affecting humanity. As the level of development and socioeconomic status of countries decreases, the prevalence of *H. pylori* infection increases. Although numerous *H. pylori* eradication regimens have been tested in recent years, the desired level of eradication success has not been achieved. This study aimed to evaluate the effectiveness of alternative treatment protocols.

**Materials and Methods:** Patients who presented to the clinic between 2023 and 2024 were included in the study. The patient groups were treated with rabeprazole (R), metronidazole (M), amoxicillin (A), and bismuth (B) (RMAB), or with esomeprazole (E), metronidazole (M), tetracycline (T), and bismuth (B) (EMTB) regimens. Patients who were resistant to both RMAB and EMTB treatment protocols received quintuple therapy with RTAMB and ETAMB. All patients were thoroughly informed about their treatment process, management of side effects, and adherence to the therapy.

**Results:** A total of 611 patients were included in the study. Of the participants, 60.7% were women, and the mean age was 53.7 ± 15.2 years. For posttreatment response evaluation, 73.5% (n = 449) of the patients returned to the clinic. The overall *H. pylori* eradication success rate for the entire patient group was 49.6%. The success rates for patients receiving RMAB and EMTB treatments were 48.2% and 51.3%, respectively. Among the group of patients who were under controlled supervision and had optimal treatment compliance (n = 449), the success rates for the RMAB and EMTB treatments were found to be 66.9% and 68.5%, respectively. RTAMB and ETAMB treatment was applied to 105 patients who were resistant to RMAB and EMTB treatments. Of these patients, 96.2% complied with the treatment and underwent a response evaluation. The eradication success rates for patients treated with the RTAMB and ETAMB protocols were 65.6% and 67.6%, respectively.

**Conclusion:** This study demonstrated that bismuth-based quintuple therapy can be used as an alternative to bismuth-based quadruple therapies and other high-risk treatments. However, the critical importance of treatment adherence was also highlighted.

Table 1.Clinical and Demographic Data of the Groups

BS, bismuth; CAD, coronary artery disease; DM, diabetes mellitus; EMTB, esomeprazole, metronidazole, tetracycline, bismuth; ETAMB, esomeprazole, tetracycline, amoxicillin, metronidazole, bismuth; HT, hypertension; RMAB, rabeprazole, metronidazole, amoxicillin, bismuth; RTAMB, rabeprazole, tetracycline, amoxicillin, metronidazole, bismuth.


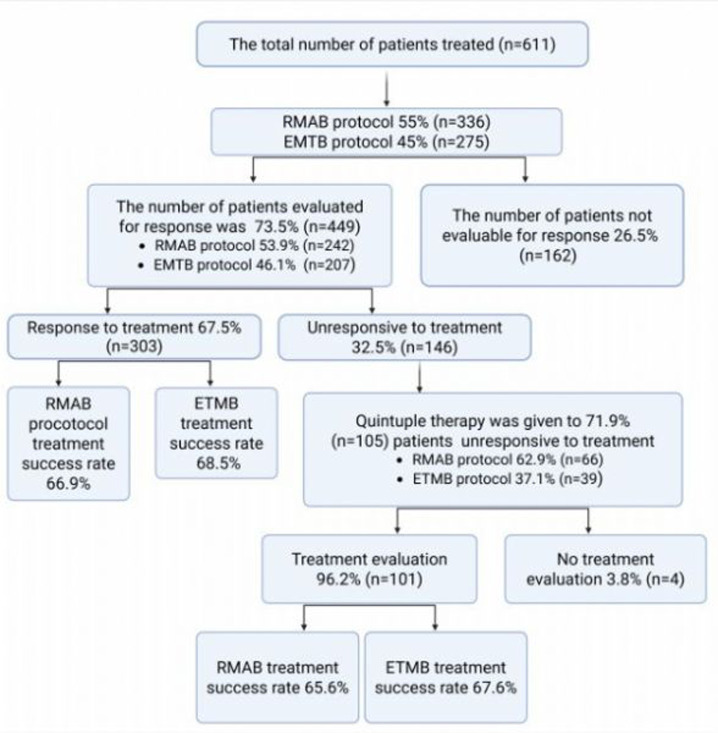


Figure 1. Treatment flow diagram. RMAB, rabeprazole, metronidazole, amoxicillin, bismuth; ETMB, esomeprazole, metronidazole, tetracycline, bismuth; RTAMB, rabeprazole, tetracycline, amoxicillin, metronidazole, bismuth; ETAMB, esomeprazole, tetracycline, amoxicillin, metronidazole, bismuth.

**Table:** 

	All Patients	Patients Compliant with Treatment
Age (years)	53.7 ± 15.2	54.3 ± 15.7
Female, n (%)	370 (60.6)	275 (61.2)
BS-based quadruple treatment		
RMAB, n (%)	336 (55)	242 (53.9)
EMTB, n (%)	275 (45)	207 (46.1)
BS-based quintuple therapy		
RTAMB, n (%)	66 (62.9)	64 (63.4)
ETAMB, n (%)	39 (37.1)	37 (36.6)
Presence of comorbidity		
Presence of DM, n (%)	57 (9.3)	50 (11.2)
Presence of HT, n (%)	121 (19.8)	95 (21.2)
Presence of asthma, n (%)	63 (10.3)	49 (10.2)
Presence of CAD, n (%)	45 (7.4)	37 (8.2)
No comorbidities	398 (65.1)	280 (62.4)

## SS-73 Is It Possible to Stop Treatment? Clinical Experience in HBeAg-Negative Chronic Hepatitis B

**Merve Eren Durmuş ^1^ , Gökhan Köker ^2^ , Galip Egemen Atar ^1^ , Ferda Akbay Harmandar ^1^ , Serdar Akça ^1^ , Serkan Öcal ^1^ , Ayhan Hilmi Çekin ^1^**

^1^Department of Gastroenterology, University of Health Sciences Antalya Training and Research Hospital, Antalya, Türkiye

^2^Department of Internal Medicine, University of Health Sciences, Antalya Training and Research Hospital, Antalya, Türkiye

**Background/Aims: **Chronic hepatitis B (CHB) is a major health problem worldwide. Long-term nucleos(t)ide analog (NA) therapy suppresses viral replication and reduces the risk of cirrhosis and hepatocellular carcinoma. However, prolonged therapy is challenging, and HBsAg loss with anti-HBs seroconversion is rare. These outcomes are the best markers of functional cure. Thus, evaluating serological outcomes after treatment discontinuation is important, especially in HBeAg-negative patients.

**Materials and Methods:** Twenty-four HBeAg-negative patients followed in the clinic were included. During follow-up, therapy was restarted in 1 patient due to cirrhosis. The mean treatment duration was 564.5 weeks (~10.8 years), and mean follow-up after cessation was 57 weeks (~1.1 years).

**Results:** HBsAg loss occurred in 4 patients (16%), with a mean time of 69.8 weeks. Of these, 1 received TAF/TDF, 2 ETV, and 1 TDF. In 1 case with HBsAg loss, anti-HBs positivity developed at week 81.9, whereas in another patient still HBsAg-positive, anti-HBs positivity developed at week 65. Seven patients (29%) became inactive carriers (5 ETV, 2 TAF/TDF). Six (25%) required retreatment: 1 received TAF/TDF, 1 ETV, 3 TAF/TDF, and 1 ETV. All had virological relapse, and 2 also experienced biochemical relapse. One additional patient resumed therapy by choice.

**Conclusion:** These findings suggest that functional cure and immune response are possible in selected HBeAg-negative patients who discontinue NA therapy. HBsAg loss and anti-HBs seroconversion occurred at rates consistent with prior reports, supporting seroconversion as the main goal. Still, the 25% retreatment rate highlights the need for careful patient selection. The presence of cirrhosis during follow-up indicates that the risk of complications persists. Larger prospective studies are needed to define safe discontinuation and strategies to enhance seroconversion.


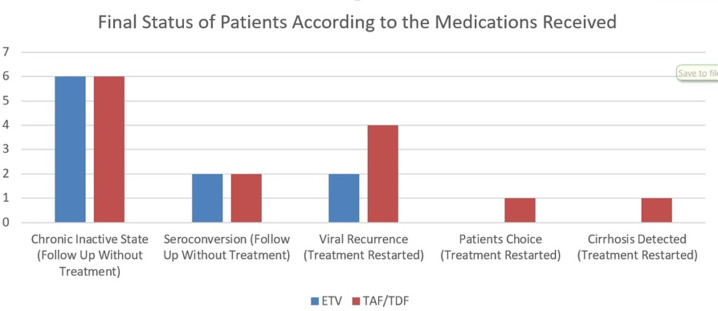


Figure 1. Final status of patients according to the medications received.

## SS-75 The Prevalence of Hepatitis E Virus Infection in the Adult Turkish Population: A Systematic Review of the Literature and Prevalence Study in Blood Donors in Mersin Province

**Orhan Sezgin ^1^ , Serkan Yaraş ^1^ , Gönül Aslan ^2^ , Seda Tezcan Ülger ^2^ , Eyyüp Naci Tiftik ^3^**

^1^Department of Gastroenterology, Mersin University School of Medicine, Mersin, Türkiye

^2^Department of Medical Microbiology, Mersin University School of Medicine, Mersin, Türkiye

^3^Department of Hematology, Mersin University School of Medicine, Mersin, Türkiye

**Background/Aims:** The hepatitis E virus (HEV) is an RNA virus that causes acute hepatitis and can become chronic in immunocompromised patients, although this is rare. The frequency of HEV infection varies depending on factors such as geographical region, socioeconomic status, and age. Despite limited studies on the adult population in Türkiye, there is currently no information about HEV frequency in the country. Therefore, this study aimed to analyze the data from such studies in comparison to the results of the present study.

**Materials and Methods:** A total of 900 healthy blood donors at the University Hospital Blood Center who consented to the use of their data were enrolled in the study. Serum anti-HEV-IgG antibody was examined using the enzyme-linked immunosorbent assay method. The donors’ location, occupation, and animal contact status were determined. Additionally, the study evaluated the full texts and conference papers (in Turkish or English) of Türkiye-based HEV seroprevalence studies from 1990 to 2020, focusing on the adult population.

**Results:** The average age was 35.22 ± 9.60 years, with 889 (98.7%) being men. Anti-HEV-IgG was positive in 12.8% of the serum samples. The average age of the seropositive volunteers was 40.40 ± 9.72 years, and 98.2% were men. No association was found between anti-HEV IgG positivity and occupation, place of residence, or contact with animals. An evaluation of the studies conducted in Türkiye reveals that the average HEV infection seroprevalence is 9.52% in the healthy population, with higher prevalence in the Southeastern Anatolia region. Patients with acute hepatitis and those on hemodialysis also had increased rates.

**Conclusion:** The anti-HEV IgG seropositivity rate in healthy blood donors in Mersin province was 12.8%, which is similar to the rates reported earlier in the country. However, this rate, found in a sample of individuals from a healthy population, raises concern about what the frequency may be in sick individuals. Wide-ranging community screening is needed.

## SS-78 Clinical Characteristics of Metabolic Dysfunction-Associated Steatotic Liver Disease Patients Over 60 Years of Age: A Biopsy-Proven Study from Türkiye and Comparison with Other Age Groups

**Zeynep Kaşıkcı ^1^ , Eda Kaya ^2^ , Yusuf Yılmaz ^3^**

^1^Recep Tayyip Erdoğan University School of Medicine, Rize, Türkiye

^2^Department of Medicine, Ruhr University Bochum, University Hospital Knappschaft Kliniken Bochum, Bochum, Germany

^3^Department of Gastroenterology, Recep Tayyip Erdoğan University School of Medicine, Rize, Türkiye

**Background/Aims:** Metabolic dysfunction-associated steatotic liver disease (MASLD) is increasingly prevalent with age. Although older adults have more metabolic risk factors, aging is paradoxically linked to weight loss. This study aimed to examine the clinical features—particularly metabolic risk factors—of elderly Turkish patients with biopsy-confirmed MASLD.

**Materials and Methods:** Patients from the Turkish NAFLD Biobank were included in the analysis and stratified into age groups: 18-30, 31-40, 41-50, 51-60, and over 60 years. Histological classification of the biopsies was performed according to the Steatosis, Activity and Fibrosis (SAF) Fatty Liver Inhibition of Progression (FLIP) algorithm and the NAFLD Activity Score (NAS) scoring system.

**Results:** A total of 639 biopsy-confirmed patients with MASLD were analyzed (54.1% male (N = 346); median age 47 years). Patients over 60 showed a high prevalence of metabolic dysfunction-associated steatohepatitis (N = 52, 92.9%) and significant fibrosis (N = 39, 69.6%). The prevalence of advanced fibrosis increased progressively with age: 12.2% (N = 6), 7.5% (N = 10), 17.2% (N = 33), 28% (N = 59), and 46.4% (N = 26) across successive age groups (*P* < .001). A similar age-related trend was observed for diabetes mellitus: 10.2% (N = 5), 31.8% (N = 42), 42.4% (N = 81), 55.4% (N = 117), and 71.4% (N = 40) (*P* < .001). Age was weakly but significantly correlated with fibrosis stage (rho = 0.294, *P* < .001). Body mass index (BMI) was also weakly correlated with fibrosis (rho = 0.202, *P* < .001) and age (rho = 0.123, *P* = .002). Notably, no patients over 60 had a normal BMI. There were only 19 patients older than 65 years. The age cutoff of 55 years demonstrated the best performance in predicting advanced-stage fibrosis (AUROC: 0.684).

**Conclusion:** MASLD severity, particularly liver fibrosis, increases with age alongside comorbidities such as diabetes and obesity. These findings highlight the need for age-specific approaches in MASLD management to reduce disease progression in older adults.

## SS-79 The limitations and exclusion factors in the evaluation of living liver donor candidates: a single-center experience

**Kübra Köken ^1^ , Derya Arı ^1^ , Osman Aydın ^2^ , Dilara Turan Gökçe ^4^ , Cenkgazi Karabıyıkoğlu ^3^ , Rıza Sarper Ökten ^3^ , Erdal Birol Bostancı ^2^ , Meral Akdoğan Kayhan ^1^**

^1^Department of Gastroenterology, Ankara Bilkent City Hospital, Ankara, Türkiye

^2^Department of Gastroenterology Surgery, Ankara Bilkent City Hospital, Ankara, Türkiye

^3^Department of Radiology, Ankara Bilkent City Hospital, Ankara, Türkiye

^4^Department of Gastroenterology, Ankara University, Ankara, Türkiye

**Background/Aims:** Liver transplantation is a crucial procedure for end-stage liver disease, with living-donor liver transplantation serving as a vital alternative due to low deceased organ donation rates in the country. This study aimed to identify factors that limit the eligibility of living liver donor candidates.

**Materials and Methods:** This retrospectively analyzed data from 542 living-donor candidates who approached the Liver Transplant Coordination Center at Ankara Bilkent City Hospital from February 26, 2019, to July 22, 2025. Demographic data, volumetric measurements, parenchymal characteristics, and surgical anatomy criteria were evaluated. Key eligibility criteria included a macrovesicular steatosis rate of less than 10%, a graft-to-recipient weight ratio (GRWR) of at least 0.8, and a residual liver volume of at least 30% for donor safety.

**Results:** Out of 542 candidates, 496 were deemed suitable for evaluation by the transplant board. However, 233 candidates (43%) were found unsuitable for organ donation. Among these, 201 (86.3%) had intrahepatic contraindications, 26 (11.2%) had extrahepatic contraindications, and 6 (2.6%) had combined contraindications. The most common extrahepatic issues included newly diagnosed breast cancer (1.3%), suspicious pancreatic lesions (1.3%), and hydatid cysts (1.3%). Intrahepatic exclusions were primarily due to insufficient graft volume (31.9%), inadequate remnant liver volume (26.1%), vascular anomalies (21.3%), and hepatic steatosis (16.9%). Evaluations were halted for 38 candidates (7%) due to poor recipient condition, 32 (5.9%) due to recipient recovery, and 41 (7.6%) due to donor withdrawal. Ultimately, 152 candidates (28%) were suitable for donation, and 104 liver transplantations were successfully performed.

**Conclusion:** Many living liver donor candidates were deemed unsuitable due to volumetric insufficiency, parenchymal steatosis, or vascular and biliary variations. This highlights the importance of thorough radiological evaluation and multidisciplinary assessment to ensure donor safety in living-donor liver transplantation.

## SS-80 Frequency of Chronic Atrophic Gastritis in Patients with Cirrhosis

**Aizirek Abdikaiyrova ^1^ , Haldun Selçuk ^2^ , Ahmet Sedat Boyacıoğlu ^2^**

^1^Department of Internal Medicine, Başkent University Ankara Hospital, Ankara

^2^Department of Gastroenterology, Başkent University Ankara Hospital, Ankara

**Background/Aims:** Chronic atrophic gastritis (CAG) represents a crucial step in the Correa cascade leading to gastric cancer. In patients with cirrhosis, premalignant gastric lesions are reported in nearly one-third of cases, regardless of the severity of portal hypertension. Cirrhosis is thought to increase the risk of CAG by promoting gastric mucosal injury and inflammation. This study aimed to compare the frequency of CAG in patients with cirrhosis with a dyspeptic control group.

**Materials and Methods:** A total of 115 patients with cirrhosis (mean age: 54.6 ± 13.4 years) and 510 dyspeptic controls (mean age: 55.8 ± 14.6 years) were included. Demographics, comorbidities, endoscopic findings, and histopathological biopsy results were assessed. The presence and severity of atrophy, as well as *H. pylori* status, were determined. Gender distribution was comparable between the groups.

**Results:** The prevalence of atrophy was significantly higher in patients with cirrhosis (51.3%) compared to controls (31.7%, *P* < .001). Moreover, the severity of atrophy was significantly greater in patients with cirrhosis. *H. pylori* positivity was lower in patients with cirrhosis (17.3%) compared to controls (26.7%, *P* < .05).

**Conclusion:** Patients with cirrhosis exhibit a significantly higher frequency and severity of CAG compared to controls. These findings strengthen the association between cirrhosis, premalignant mucosal changes, and gastric cancer development. The presence of more severe and frequent CAG highlights its role as a critical risk factor for gastric carcinogenesis in cirrhosis. Routine endoscopic surveillance with targeted biopsies should therefore be considered an essential preventive strategy in this patient population.

## SS-83 Male predominance in inflammatory bowel disease despite similar disease activity: A large retrospective cohort study

**Ali Atay ^1^ , Mücahit Ergül ^1^ , Oğuz Öztürk ^1^ , Kadir Can Acun ^1^ , Yavuz Çağır ^2^ , Muhammed Bahaddin Durak ^3^ , İlhami Yüksel ^4^**

^1^Department of Gastroenterology, Ankara Bilkent City Hospital, Ankara, Türkiye

^2^Department of Gastroenterology, Ankara Yıldırım Beyazıt University Yenimahalle Training and Research Hospital, Ankara, Türkiye

^3^Department of Gastroenterology, Hacettepe University School of Medicine, Ankara, Türkiye

^4^Department of Gastroenterology, Ankara Yıldırım Beyazıt University School of Medicine, Ankara, Türkiye

**Background/Aims:** Inflammatory bowel diseases (IBD), including ulcerative colitis (UC) and Crohn’s disease (CD), exhibit clinical heterogeneity, with sex-related differences potentially influencing disease course and prognosis. This study investigates the impact of sex on IBD prognosis in a large cohort.

**Materials and Methods:** Data were collected from patients diagnosed with IBD at the clinics between July 1993 and April 2025. Demographic, clinical, and treatment variables were analyzed, focusing on sex-based differences in disease onset, disease activity, extraintestinal manifestations (EIMs), treatment escalation, hospitalizations, and surgical interventions.

**Results:** A total of 1774 patients diagnosed with IBD were included in the study. A male predominance (61%) was observed in IBD, with higher male-to-female (M/F) ratios in both UC (1.6 : 1) and CD (1.47 : 1). Female patients presented earlier with IBD (mean age 34.7 vs. 37.0 years; *P* = .001) and had a higher frequency of EIMs, particularly peripheral arthritis and erythema nodosum (*P* < .001). No significant sex differences were observed in disease severity, hospitalizations, or need for treatment escalation. However, female patients with CD demonstrated higher disease activity at baseline, as measured by the Crohn’s Disease Activity Index (CDAI). Both sexes exhibited similar rates of biologic therapy use, number of biologic therapies, hospitalizations, and major surgeries.

**Conclusion:** Despite the male predominance in IBD prevalence, sex-related differences in clinical outcomes were minimal, with both sexes experiencing comparable disease activity, treatment escalation, and need for surgery. However, female patients exhibited a higher frequency of EIMs, highlighting the importance of a sex-based approach in clinical management.

## SS-84 A Real-World, Comparative Analysis of Ustekinumab and Vedolizumab in Biologic-Exposed Patients with Moderate-to-Severe Inflammatory Bowel Disease

**Rasim Eren Cankurtaran ^1^ , Hulusi Can Karpuzcu ^3^ , Halit Kandemir ^4^ , Mücahit Ergül ^1^ , Muhammed Mustafa İnce ^1^ , Sanlı Arabacı ^5^ , Güner Kılıç ^4^ , Ali Karataş ^4^ , Seçkin Özgül ^5^ , Evrim Kahramanoğlu Aksoy ^5^ , Naciye Şemnur Büyükaşık ^2^ , Öykü Tayfur Yürekli ^2^ , Yasemin Özin ^1^ , Tarkan Karakan ^4^**

^1^Department of Gastroenterology, Ankara Bilkent City Hospital, Ankara, Türkiye

^2^Department of Gastroenterology, Ankara Yıldırım Beyazıt University Faculty of Medicine, Ankara, Türkiye

^3^Department of Gastroenterology, Ankara Etlik City Hospital, Ankara, Türkiye

^4^Department of Gastroenterology, Gazi University Faculty of Medicine, Ankara, Türkiye

^5^Department of Gastroenterology, Atatürk Sanatory Training and Research Hospital, Ankara, Türkiye

**Background/Aims: **The use of non-anti-TNF biologics has been increasing among patients with moderate-to-severe inflammatory bowel disease (IBD) who have prior biologic exposure. Ustekinumab and vedolizumab are biologic agents with distinct mechanisms of action; however, comparative real-world data are limited. This study aimed to compare the real-world effectiveness and safety of ustekinumab and vedolizumab in biologic-exposed patients with IBD.

**Materials and Methods:** This multicenter retrospective study included adult patients with moderate-to-severe IBD who had previously received biologic therapy and were subsequently treated with ustekinumab or vedolizumab. Clinical, demographic, and endoscopic data were obtained from electronic medical records. The primary endpoints were clinical response and remission at weeks 14, 52, and 104, assessed by the partial Mayo Score for ulcerative colitis (UC) and the Harvey-Bradshaw Index for Crohn’s disease (CD). Secondary endpoints included serological remission, endoscopic response, adverse events, and treatment persistence.

**Results:** A total of 384 patients (UC: 44%, CD: 54.7%) were included; 150 received vedolizumab and 234 received ustekinumab. IBD-related intestinal surgery and extraintestinal manifestations were more common in the ustekinumab group (*P* = .006 and *P* = .002). In UC, clinical and serological response rates at weeks 14, 52, and 104 were comparable between the 2 groups (*P* > .05). In CD, the clinical remission rate at week 52 was significantly higher in the ustekinumab group compared to the vedolizumab group (70.1% vs. 56.1%, *P* = .046).

**Conclusion:** Both ustekinumab and vedolizumab demonstrated comparable real-world effectiveness and safety in biologic-exposed patients with moderate-to-severe IBD. Ustekinumab showed higher midterm clinical remission rates in Crohn’s disease, and serious adverse event rates were low for both treatments.

## SS-85 Effect of Ustekinumab Treatment on Patient Quality of Life and Work Productivity in Patients with Inflammatory Bowel Disease

**Zekiye Nur Harput ^1^ , Mehmet Kasım Aydın ^2^ , Oktay Bayraktar ^2^ , Orhan Sezgin ^2^**

^1^Department of Gastroenterology, Kütahya City Hospital, Kütahya, Türkiye

^2^Department of Gastroenterology, Mersin University Faculty of Medicine, Mersin, Türkiye

**Background/Aims:** IBD is a chronic condition that can negatively impact quality of life and functional ability, leading to anxiety and depression. Although the treatments used lead to clinical and laboratory changes, improving patients’ quality of life is also an important goal of treatment. This study aimed to evaluate the effects of ustekinumab treatment on the quality of life of patients with IBD.

**Materials and Methods:** Patients over 18 years of age who were initiated on ustekinumab for IBD at the Mersin University Faculty of Medicine Gastroenterology Clinic and who agreed to participate in the study were assessed at baseline and at the 52nd week using the “Short Inflammatory Bowel Disease Questionnaire” (SIBDQ), “Patient Health Questionnaire” (PHQ-9), “Work Productivity and Activity Index” (WPAI), and “Rating IBD Patient Concerns” (RFIPC). Disease activity was assessed using the “Simple Clinical Colitis Activity Index” for UC and the “Harvey-Bradshaw Index” for CD. Patient age, gender, comorbidities, extraintestinal involvement, number of biological agents, and history of IBD-related surgery were recorded.

**Results:** Fifty-four patients who completed the 52nd week of treatment were evaluated (25 UC, 29 CD). The mean age was 39.5 ± 14.4 years, and 50% were male. The median disease duration was 5 years (min: 1 year, max:24 years). In the comparison, decreases in PHQ9, RFIPC, and WPAI scores (*P* = .022, *P* = .002,* P* < .001), and increases in the SIBDQ total score (*P* < .001) were detected. The results for UC and CD were similar. No significant differences were observed with respect to age, disease duration, education level, marital status, presence of extraintestinal involvement, or chronic diseases. Although the total score of females was lower compared to that of males only in SIBDQ (*P* = .03), the scores of other questionnaires were similar (*P* > .05). Except for a limited positive correlation between the severity of CD and WPAI (*P* = .05), no significant correlation was observed between the total scores of all questionnaires and the severity of both diseases.

**Conclusion:** It was determined that the 52nd week of ustekinumab treatment had an effect on the quality of life and work productivity of the patients.

Table 1.Comparison of Ustekinumab Treatment Initiation and Posttreatment at Week 52

Values are presented as mean ± SD; groups were compared using the paired sample *t*-test.

PHQ-9, Patient Health Questionnaire; RFIPC, Rating Inflammatory Bowel Disease Patient Concerns; SIBDQ, Short Inflammatory Bowel Disease Questionnaire; WPAI, Work Productivity and Activity Index.

**Table:** 

**Questionnaire**	**n**	**Mean**	**SD**	***P***
PHQ-9 baseline PHQ-9 week 52	54 54	10.9630 9.1667	5.69330 6.23063	**.0022**
SIBDQ baseline SIBDQ week 52	54 54	35.5185 45.5370	14.68762 13.85874	**<.001**
RFIPC baseline RFIPC week 52	54 54	3.9496 3.1393	2.03703 2.07649	**.002**
WPAI (activity impairment) (%) Baseline WPAI (activity impairment) (%) week 52	54 54	52.4074 34.4444	31.91588 30.32321	**<.001**

## SS-86 CATCH Score—Evaluating Abdominal CT Necessity in Crohn’s Disease with Sudden-Onset Complaints

**Ömer Faruk Özkan ^1^ , Ali Karataş ^2^ , Mehmet Cindoruk ^2^ , Tarkan Karakan ^2^ , Murat Kekilli ^2^ , Çağdaş Kalkan ^2^ , Güner Kılıç ^2^**

^1^Department of Internal Medicine, Gazi University Faculty of Medicine, Ankara, Türkiye

^2^Department of Gastroenterology, Gazi University Faculty of Medicine, Ankara, Türkiye

**Background/Aims:** To evaluate the necessity of abdominal computed tomography (CT) and the need for invasive interventions in patients with Crohn’s disease presenting with acute symptoms; to validate the performance of the CATCH score; and to propose an improved predictive model.

**Materials and Methods:** This single-center, retrospective, cross-sectional study included 202 patients with Crohn’s disease who underwent abdominal CT for acute symptoms at Gazi University Gastroenterology Clinic between January 1, 2020 and January 10, 2023. Demographics, CATCH components (ileocolonic involvement, C-reactive protein [CRP], platelet count, neutrophil percentage, smoking, fever, diarrhea), and additional clinical and disease history variables were recorded. CT outcomes were categorized as obstruction, abscess, perforation, fistula, or non-Crohn-related findings. Primary outcomes were (i) a clinically significant CT finding and (ii) the need for an invasive intervention.

**Results:** Clinically significant CT findings were detected in 62/202 patients (30.7%). The CATCH score, increased neutrophils and platelets, higher CRP, longer disease duration, and a history of intra-abdominal abscess were significantly associated with positive CT findings. Receiver operating characteristic analysis identified an optimal CATCH cutoff of 1.5 (sensitivity 67.7%, specificity 92.1%). Adding a history of intra-abdominal abscess and disease duration to the CATCH framework increased sensitivity while preserving specificity for predicting positive CT results. Among patients who required invasive interventions, the CATCH score and prior intra-abdominal abscess were significant predictors.

**Conclusion:** The CATCH score is a practical tool with high specificity (threshold ≥1.5) for guiding abdominal CT indications and anticipating the need for invasive interventions in acute Crohn’s presentations. Enhancing the score with abscess history and disease duration improves sensitivity. These findings may help reduce unnecessary CT use and better identify patients who require early intervention. Prospective, multicenter validation is warranted.

## SS-88 Seroprevalence of Borrelia burgdorferi and Impact of Its Antibody Seropositivity in Patients with Ulcerative Colitis

**Gökhan Aydın, Ahmet Cumhur Dülger, Eray Beşirli**

Division of Gastroenterology, Giresun University School of Medicine, Giresun, Türkiye

**Background/Aims:** Lyme disease is a significant zoonotic tick-borne disease with increasing prevalence in the US and northern hemisphere. The infected tick of the genus Ixodes is responsible for spreading the disease. Many studies have described a wide range of genetic, immunologic, and environmental risk factors in patients with ulcerative colitis (UC). However, there is still no report on whether the prevalence and impact of Borrelia burgdorferi antibodies exist in patients with UC.

**Materials and Methods:** This was a single-center retrospective observational study of patients with UC. The medical records of gastroenterology clinic patients with UC between October 2024 and October 2025 were reviewed. A total of 100 (41 female; mean age 48.5 ± 17 years) patients with UC were tested for BB antibodies. Patients were divided into seropositive and seronegative groups.

**Results:** A total of 22 patients tested positive for BB IgG antibodies(prevalence was 22%). There were no gender or age differences between groups (*P* > .005). Although the mean levels of CRP and serum uric acid were lower in the seropositive group (4829 ± 7967 U/L vs. 10.5 ±16.76 U/l and 4.38 ±1.27/dL vs. 5606 ± 1353 mg/dL, *P* = .020 and *P* = .001), the mean levels of MCV and MCH were higher in patients who tested positive for BB IgG antibodies (88 427 ± 4065 vs. 85 241 ± 6847 fL. *P* = .032 and 29 173 ± 2195 versus 27 637 ± 3076 g/dL, *P* = .025). Other laboratory parameters did not differ significantly between groups.

**Conclusion:** In conclusion, these data indicate, for the first time, that seropositivity of BB antibodies in patients with UC has been associated with low disease activity in those with UC, and this unique association can lead to the design of new diagnostic and prognostic tools in the treatment of UC.

## SS-90 Evaluation of the Effectiveness of the Questionnaire in Determining Fecal Incontinence and Urgency in Patients with Inflammatory Bowel Disease: Who Should Administer It? Self-Report? Clinician?

**Engin Altintas ^1^ , Melis Yilmaz Gür ^2^**

^1^Department of Gastroenterology, Mersin University Faculty of Medicine, Mersin, Türkiye

^2^Department of Internal Medicine, Mersin University Faculty of Medicine, Mersin, Türkiye

**Background/Aims:** Fecal incontinence (FI) and defecation urgency are common problems in patients with inflammatory bowel disease (IBD) (Crohn’s disease and ulcerative colitis) and negatively impact their quality of life due to the unpredictable nature of the disease. This study aimed to compare whether a self-report questionnaire or face-to-face clinician interviews were more effective in determining FI and defecation urgency in patients with IBD.

**Materials and Methods:** This prospective study included 64 patients with CD and 64 patients with UC. Patients’ fecal incontinence severity was assessed using the Fecal Incontinence Severity Index (FISI), and quality of life was assessed using the Fecal Incontinence Quality of Life Scale (FIQOL). These assessments were conducted through self-report in 64 of the 128 patients and through face-to-face interviews in 64 patients.

**Results:** A moderately positive and statistically significant correlation was found between the FISI score and both UC and CD activity (*r* = 0.605, *P* < .001 and *r* = 0.444, *P* < .001). The mean FISI score of the self-reported patients (15.8 ± 10.1) was higher compared to the mean FISI score of the face-to-face survey patients (12.8 ± 10.2), but no statistically significant difference was found between them (*P* = .093). A moderately significant negative correlation was found between the disease activity index and the FIQOL score (*r* = −0.533, *P* < .001 for UC and *r* = −0.457, *P* < .001 for CD). The mean FIQOL score of patients who self-reported was 73.5 ± 25.9, whereas the mean FIQOL score of patients who received a face-to-face survey was 86.9 ± 27.8, and a statistically significant difference was found between them (*P* = .006).

**Conclusion:** FISI and FIQOL scores were not different in CD and UC. There was also no difference in gender, but there was an inverse relationship between the number of births and pregnancies among women and the FIQOL score.

## SS-91 The Role of Oral Calcium Butyrate in Mitigating Age-Related Colonic and Cerebral Inflammation and Supporting Intestinal Barrier Integrity

**Sevim Kandiş Sever ^1^ , Rabia Ilgın Alan ^3^ , Mehmet İbrahim Tuğlu ^2^ , Nazan Uysal Harzadın ^3^**

^1^Department of Physiology, Dokuz Eylül University Faculty of Medicine, İzmir, Türkiye

^2^Department of Histology and Embryology, Manisa Celal Bayar University Faculty of Medicine, Manisa, Türkiye

^3^Independent Researcher, İzmir, Türkiye

**Background/Aims:** To test whether oral calcium butyrate (CaB), a microbiota-derived short-chain fatty acid, mitigates age-related inflammation and histopathology across the colon-brain axis.

**Materials and Methods:** Young adult (12-14 weeks) and aged (12-14 months) Wistar rats received CaB at a dose of 300 mg/kg/day by mouth for 21 days or vehicle. The hippocampus and colon were collected to assess tight-junction proteins (occludin/OCLN, ZO-1), neuroglial markers (NeuN, GFAP, Iba-1), and cytokines (TNF-α, IL-10, IL-1β, IL-6) using standard histology/immunohistochemistry and immunoassays. Group comparisons evaluated age- and treatment-dependent effects.

**Results:** CaB shifted the inflammatory milieu toward an anti-inflammatory profile, increasing IL-10 and lowering TNF-α in both hippocampal and colonic tissues. This effect was most pronounced in the hippocampus of young rats and in the colon of aged rats. NeuN expression was increased in the CaB groups compared to controls, whereas GFAP and Iba-1 were reduced, indicating attenuated gliosis and preservation of neuronal integrity. In the colon, ZO-1 and OCLN were significantly upregulated, with larger gains observed in aged animals, consistent with improved epithelial barrier status.

**Conclusion:** Twenty-one days of oral CaB enhances intestinal tight-junction protein expression and attenuates neuroinflammatory and systemic inflammatory markers in both young adult and aged rats, with region- and age-specific maxima (young hippocampus; aged colon). These findings support CaB as a candidate adjunct to bolster colon barrier integrity and brain homeostasis during aging.

## SS-92 Dietary Management and Follow-up in Pediatric Patients with Eosinophilic Esophagitis

**Büşra Akyol Yılmaz ^1^ , Merve Usta ^2^ , Sevgi Sipahi Çimen ^3^ , Ayşegül Gümüşlü ^2^ , Nafiye Urgancı ^2^**

^1^Department of Pediatrics, Şişli Hamidiye Etfal Training and Research Hospital, University of Health Sciences, İstanbul, Türkiye

^2^Department of Pediatrics, Şişli Hamidiye Etfal Training and Research Hospital, University of Health Sciences, İstanbul, Türkiye

^3^Department of Pediatric Allergy, Şişli Hamidiye Etfal Training and Research Hospital, University of Health Sciences, İstanbul, Türkiye

**Background/Aims:** Eosinophilic esophagitis (EoE) is a chronic immune/antigen-mediated esophageal disease that presents with abdominal pain, nausea, vomiting, or refractory reflux in children, and dysphagia or food impaction in older patients. Histological confirmation requires at least 15 eosinophils per high-power field. Nutrition plays a key role in treatment, complementing pharmacological therapy. The main dietary strategies include elemental diet, empiric six-food elimination, and allergy test-directed elimination. Dietary therapies and outcomes of pediatric patients with EoE under multidisciplinary follow-up were evaluated.

**Materials and Methods:** Medical records of patients diagnosed between 2017 and 2024 were retrospectively analyzed. Diagnosis was based on clinical, endoscopic, and histopathological findings. Patients were followed by pediatric gastroenterologists, allergists, and dietitians. Demographics, anthropometrics, and therapeutic approaches were recorded.

**Results:** Twenty-one patients (20 males) were included, with a mean age of 12.2 ± 4.25 years; the mean age at diagnosis was 9.9 ± 4.62 years. At diagnosis, 2 patients had a BMI below −2 SDS, and 2 above +2 SDS. The mean height SDS was −0.14 ± 1.38; the mean BMI SDS was −0.171 ± 1.08. Fifteen patients received combined pharmacological and dietary therapy, whereas 6 had pharmacological therapy alone. Among dietary therapies, 13 followed allergy test-based elimination, 1 began with an elemental diet (later switched to test-based elimination), and 1 received empiric elimination. Milk and egg were the most frequently excluded foods. During follow-up, 7 continued combined therapy, 1 used pharmacological therapy alone, 1 discontinued the diet but maintained pharmacological treatment, 2 achieved remission, and 4 were lost to follow-up for over 1 year.

**Conclusion:** Dietary therapy in pediatric EoE should be individualized and closely monitored to support growth. Long-term management requires coordination among gastroenterologists, allergists, and dietitians, integrating dietary and pharmacological strategies to optimize outcomes.

## SS-93 Parameters Predicting Disease Severity in Hypertriglyceridemia-Associated Acute Pancreatitis

**Yunus Günegül, İlyas Ethem Şenocak, Talha Ercan, Yunus Emre Demiral, Arda Bilgili, Ahmet Tarık Eminler**

Division of Gastroenterology, Sakarya University Faculty of Medicine, Sakarya, Türkiye

**Background/Aims: **This study aimed to identify parameters predicting severity, prognosis, and recurrence risk in hypertriglyceridemia-associated acute pancreatitis (HTG-AP).

**Materials and Methods: **Between January 1, 2015, and May 1, 2025, 2473 patients hospitalized with acute pancreatitis at Sakarya University Training and Research Hospital were retrospectively evaluated. Seventy-five patients (21 females, 54 males) with HTG-AP (serum triglyceride ≥500 mg/dL) were included. Disease severity was classified by the Revised Atlanta criteria into mild (class 1, n = 42) and moderately severe–to-severe (class 2-3, n = 33) groups. Admission laboratory parameters were analyzed, including C-reactive protein (CRP), CRP-to-albumin ratio (CAR), Prognostic Nutritional Index (PNI), prothrombin time-to-albumin ratio (PTAR), LDH-to-albumin ratio, HDL-to-monocyte ratio, and triglyceride-to-HDL ratio.

**Results:** CRP and CAR were significantly higher in moderately severe–to-severe patients, whereas PNI was inversely related and lower (*P* < .05). PTAR and HDL/monocyte ratio showed limited discriminatory value and were not statistically significant. LDH/Alb, TG/HDL, and admission TG levels did not correlate with severity. Recurrence occurred in 45.5% of the moderately severe–to-severe group compared to 11.9% in the mild group, representing a 3.8-fold higher risk (RR = 3.82; 95% CI: 1.55-9.43; *P* = .0015). No parameter significantly predicted recurrence.

**Conclusion:** In HTG-AP, CRP, CAR, and PNI emerged as significant predictors of severity. Compared with the guideline-recommended 48-72 hours CRP (≥150 mg/L), baseline CRP, CAR, and PNI at admission may offer a more practical tool for early risk stratification. CRP and CAR showed superior sensitivity, whereas PNI demonstrated greater specificity.


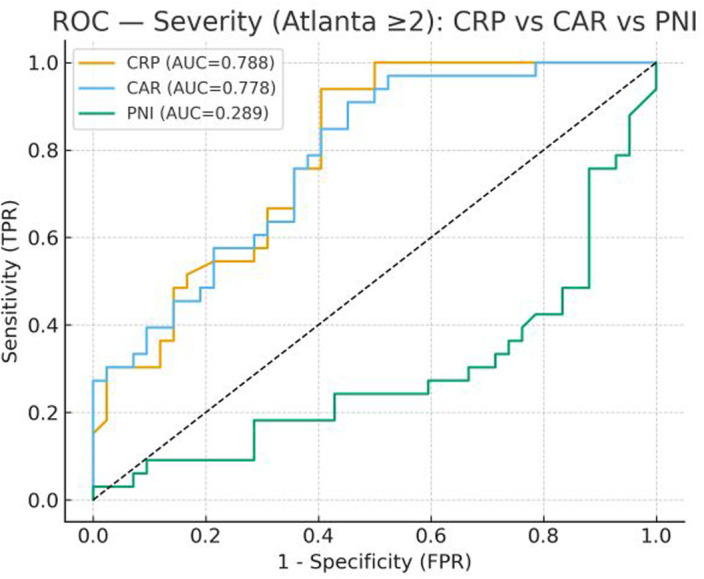


Figure 1. ROC analysis of parameters. CRP AUC = 0.788, cutoff ≈ 83.6 mg/L, sensitivity = 0.94, specificity = 0.60, *P* < .05; CAR AUC = 0.778, cutoff ≈ 23.2, sensitivity = 0.91, specificity = 0.55, *P* < .05; Low PNI associated with increased severity: AUC ≈ 0.711, cutoff ≈ 44.3, sensitivity = 0.70, specificity = 0.71, *P* < .05.

## SS-94 Predictive Value of Noninvasive Fibrosis Scores for the Presence and Grade of Esophageal Varices in Liver Cirrhosis

**Bayram Yeşil ^1^ , Zekiye Nur Öztürk ^2^ , Artuner Varlıbaş ^2^ , Ramis Çatalbaş ^1^ , Aykut Hacıömeroğlu ^2^ , Dilek Oğuz ^2^**

^1^Department of Gastroenterology, Kırıkkale University Faculty of Medicine Hospital, Kırıkkale, Türkiye

^2^Department of Internal Medicine, Kırıkkale University Faculty of Medicine Hospital, Kırıkkale, Türkiye

**Background/Aims:** Esophageal varices are a major cause of mortality in patients with cirrhosis. Although endoscopy remains the gold standard, its limited accessibility highlights the need for reliable noninvasive predictors.

**Materials and Methods:** Four hundred patients with cirrhosis who underwent endoscopy for variceal evaluation were retrospectively analyzed. Laboratory parameters and noninvasive fibrosis scores (APRI, FIB-4, KING’s, FibroQ) were assessed in relation to the presence and grade of varices.

**Results:** The mean age was 63.0 ± 12.3 years; 168 patients (42.0%) were female and 232 (58.0%) male. Varices were absent in 61 patients (15.3%) and present in 339 patients (84.8%). Variceal grades were distributed as follows: 126 patients (31.5%) were grade 1, 125 patients (31.3%) were grade 2, and 88 patients (22.0%) were grade 3. Patients with varices showed significant differences in PLT, PCT, spleen size, APRI, FIB-4, PLT/spleen, RBC/PLT, KING’s, and FibroQ. Increasing variceal grade was negatively correlated with PLT and PLT/spleen and positively correlated with APRI, FIB-4, KING’s, FibroQ, and spleen size. Ordinal logistic regression identified APRI (OR = 1.259, *P* = .009), FIB-4 (OR = 1.111, *P* < .001), FibroQ (OR = 1.022, *P* = .002), and KING’s (OR = 1.006, *P* = .018) as independent predictors, with APRI being the strongest.

**Conclusion: **This study demonstrates that hematological parameters and noninvasive fibrosis scores possess significant predictive value for both the presence and severity of esophageal varices in patients with cirrhosis. Although endoscopy remains indispensable for diagnosis and grading, the use of simple, low-cost, and easily accessible tests may help prioritize high-risk patients, especially in settings with limited endoscopic availability. The strong predictive performance of APRI highlights the potential of noninvasive indices to complement, and possibly guide, endoscopic assessment in future clinical practice.

## SS-95 Prognostic Value of the MPV/Albumin Ratio in Acute Cholangitis

**Bayram Yeşil ^1^ , Zekiye Nur Öztürk ^2^ , Artuner Varlıbaş ^2^ , Ramis Çatalbaş ^1^ , Aykut Hacıömeroğlu ^2^ , Dilek Oğuz ^1^**

^1^Department of Gastroenterology Kırıkkale University Faculty of Medicine, Kırıkkale, Türkiye

^2^Department of Internal Medicine, Kırıkkale University Faculty of Medicine, Kırıkkale, Türkiye

**Background/Aims:** Acute cholangitis is associated with systemic inflammation and variable clinical severity. Mean platelet volume (MPV), serum albumin, and their ratio (MPV/albumin) may reflect inflammatory burden and nutritional status. This study aimed to evaluate whether the MPV/albumin ratio differs across cholangitis severity grades and to assess its prognostic performance for intensive care unit (ICU) requirements and in-hospital mortality.

**Materials and Methods:** This retrospective study included patients diagnosed with acute cholangitis in a tertiary gastroenterology clinic. Admission laboratory parameters were extracted, and cholangitis severity was graded. MPV, albumin, and MPV/albumin were compared across grades. Prognostic performance for ICU admission and mortality was analyzed using ROC curves, and optimal cutoffs were determined.

**Results:** A total of 619 cases were evaluated: 263 were grade 1 (42.5%); 155 were grade 2 (25.0%); and 201 (32.5%) were grade 3. Forty-six (7.4%) patients required ICU admission, and 38 (6.1%) died during observation. MPV levels increased significantly with higher cholangitis grade (8.6 ± 1.25; 8.78 ± 1.44; 9.78 ± 1.7; *P* ≤ .001). Albumin levels decreased significantly as grade increased (3.96 ± 0.46; 3.74 ± 0.55; 3.32 ± 0.63; *P* ≤ .001). MPV/ALB differed across grades (2.21 ± 0.47; 2.43 ± 0.71; 3.09 ± 0.95; *P* ≤ .001; post-hoc: all pairwise *P* ≤ .05). For mortality, ROC analysis showed MPV (AUC = 0.628; *P* = .008), albumin (AUC = 0.185; *P* ≤ .001), and MPV/albumin (AUC = 0.800; *P* ≤ .001) discriminated nonsurvivors; MPV/albumin at 2.42 identified deaths with 89.5% sensitivity and 57.7% specificity. For ICU requirement, ROC analysis showed MPV (AUC = 0.609; *P* = .014), albumin (AUC = 0.191; *P* ≤ 0.001), and MPV/albumin (AUC = 0.789; *P* ≤ .001) were discriminatory; MPV/albumin at 2.81 detected ICU cases with 69.6% sensitivity and 77.7% specificity.

**Conclusion:** The MPV/albumin ratio varies across cholangitis severity grades and demonstrates good prognostic performance for both ICU requirements and in-hospital mortality. These findings suggest that MPV/albumin, obtainable at admission, may aid in early risk stratification in acute cholangitis.


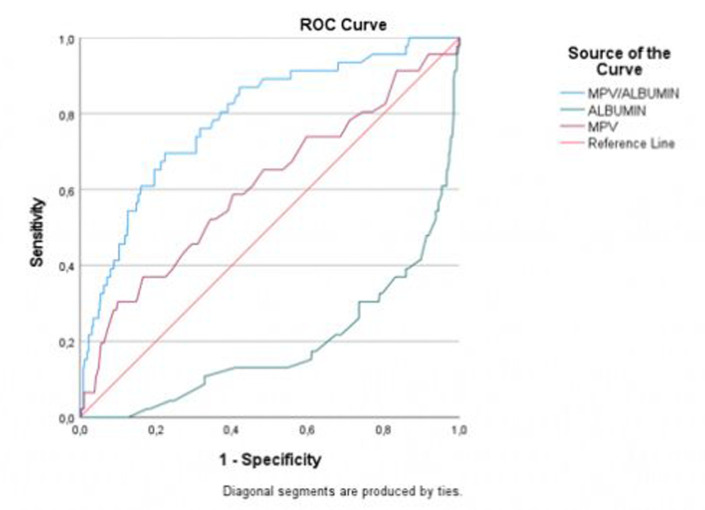


Figure 1. MPV/albumin ratio and ICU, ROC analysis.


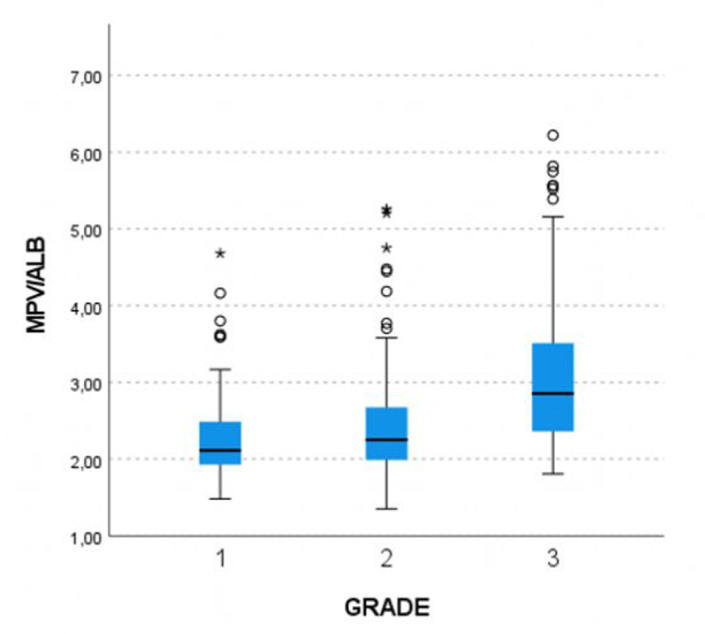


Figure 2. MPV/albumin ratio and cholangitis stages.


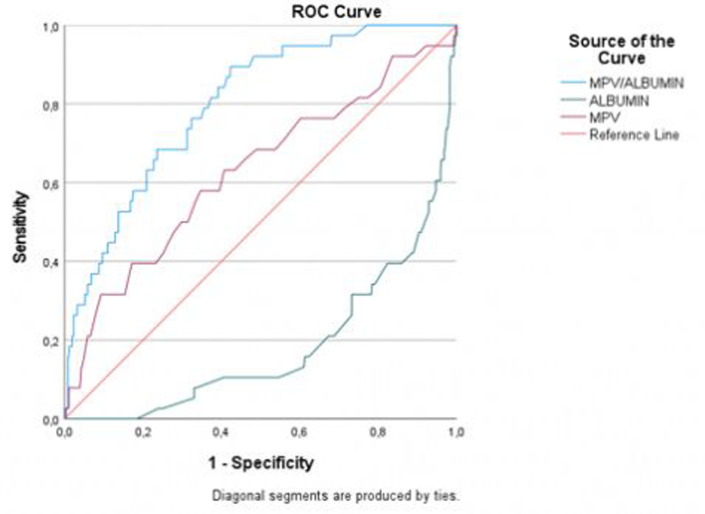


Figure 3. MPV/albumin ratio and mortality, ROC analysis.

## SS-98 Effect of Aspirin Resistance on Aspirin-Associated Upper Gastrointestinal Bleeding

**Serap Baysal Onkun ^1^ , Serkan Öcal ^2^ , Serdar Akça ^2^ , Osman Çağın Buldukoğlu ^2^ , Galip Egemen Atar ^2^ , Ferda Akbay Harmandar ^2^ , Ayhan Hilmi Çekin ^2^**

^1^Department of Internal Medicine, Antalya Resource and Education Hospital, Antalya, Türkiye

^2^Department of Gastroenterology, Antalya Resource and Education Hospital, Antalya, Türkiye

**Background/Aims:** Aspirin is widely prescribed for the prevention of cardiovascular and cerebrovascular diseases; however, upper gastrointestinal (GI) bleeding remains its most significant adverse effect. In recent years, “aspirin resistance” has emerged as a clinical challenge that may compromise treatment efficacy. This study aimed to investigate the impact of aspirin resistance on aspirin-associated upper GI bleeding.

**Materials and Methods: **This prospective, single-center, cross-sectional study was conducted between May 25, 2024, and May 20, 2025, at the S.B.U. Antalya Training and Research Hospital. A total of 130 patients aged over 18 years who had been using aspirin regularly for at least 2 weeks were enrolled. Patients presenting with upper GI bleeding were included, excluding those with a prior history of GI bleeding. Demographic data, comorbidities, vital signs, smoking status, body mass index, aspirin dosage and duration, clinical scores, endoscopic findings, and laboratory parameters were recorded. Aspirin resistance was assessed using the light transmission aggregometry (LTA) method.

**Results: **The mean age of participants was 68.5 years, with a predominance of male patients (~69%). Hypertension (68.5%) and congestive heart failure (51.5%) were the most frequent comorbidities. Endoscopy revealed peptic ulcers in 35.3% of cases (18.4% gastric, 16.9% duodenal). Aspirin resistance was detected in 27.6% of the study population. Patients with aspirin resistance demonstrated a higher likelihood of bleeding, raising concerns about the safety of dose escalation strategies.

**Conclusion: **Although current literature suggests increasing aspirin dosage in cases of resistance, these findings indicate that this approach may increase the risk of upper GI bleeding. Therefore, dose adjustment in aspirin-resistant patients should be considered cautiously, and further large-scale studies are warranted to clarify this relationship.

## SS-99 The Role of Serum Gasdermin D Level Kinetics in Predicting the Severity and Prognosis of Acute Pancreatitis

**Tayfun Ustabaş ^1^ , Serkan Öcal ^2^ , Serdar Akça ^2^ , Ferda Akbay Harmandar ^2^ , Osman Çağın Buldukoğlu ^2^ , Galip Egemen Atar ^2^ , Ayhan Hilmi Çekin ^2^**

^1^Department of Internal Medicine, University of Health Sciences, Antalya Training and Research Hospital, Antalya, Türkiye

^2^Department of Gastroenterology, University of Health Sciences, Antalya Training and Research Hospital, Antalya, Türkiye

**Background/Aims: **Gasdermin D (GSDMD) is the key effector protein of pyroptosis, an inflammatory form of programmed cell death. It forms pores in the cell membrane, leading to cell lysis and the release of proinflammatory cytokines. This study aimed to investigate the relationship between serum GSDMD level kinetics and the severity of acute pancreatitis (AP), as well as its potential as a prognostic biomarker.

**Materials and Methods: **In this prospective case-control study, 65 patients diagnosed with AP and 65 age- and sex-matched healthy controls were included. Serum GSDMD levels were measured by ELISA at admission (baseline) and at the 48th hour in the AP group. The severity of pancreatitis was assessed using the Revised Atlanta Classification, BISAP, Ranson, and CTSI scoring systems. GSDMD levels were compared with these scores.

**Results:** Serum GSDMD levels in the AP group were significantly lower compared to those in the control group (*P* < .001). There was no significant difference between baseline and 48-hour serum GSDMD levels within the AP group (*P* = .572). GSDMD levels did not show a significant correlation with disease severity based on the Revised Atlanta, BISAP, Ranson, or CTSI scoring systems.

**Conclusion: **These results suggest that serum GSDMD levels may not adequately reflect systemic inflammation or tissue injury in acute pancreatitis. Since pyroptosis is only one of several regulated cell death pathways, including apoptosis and necroptosis, it may not represent the predominant mechanism in all cases. Further studies incorporating tissue-level evaluations and exploring alternative cell death pathways are needed to clarify the role of GSDMD in the pathogenesis and prognosis of acute pancreatitis.

## SS-100 Endoscopic Sleeve Gastroplasty in Türkiye: Early Experience and Clinical Outcomes

**Mehmet Çelikbilek, Hande Aldemir Toptaş**

Private Clinic, Ankara, Türkiye

**Background/Aims: **Endoscopic sleeve gastroplasty (ESG) uses full-thickness suturing to reconfigure the gastric body and reduce its volume. This study aimed to present the initial experience and outcomes in a real-life clinical setting in Türkiye.

**Materials and Methods: **Patients who underwent ESG between October 2022 and August 2025 were retrospectively analyzed. Demographic and anthropometric data, comorbidities, suture counts, and techniques were recorded. Follow-up was scheduled weekly for 2 months and biweekly thereafter up to 1 year, either face-to-face or online. Nutrition counseling emphasized behavioral change with individualized diet plans rather than strict restriction.

**Results: **A total of 29 patients (82.8% female; mean age 39.1 ± 8 years; baseline weight 101 ± 16.8 kg; BMI 36.6 ± 5.4 kg/m^2^) were included. Minor adverse events such as abdominal discomfort, nausea, or vomiting occurred in most patients; 1 required overnight hospitalization. No serious complications or nutritional deficiencies occurred. Follow-up data were available for 29, 26, 22, and 9 patients at 1, 3, 6, and 12 months, respectively. Mean BMI decreased from 36.6 ± 5.4 kg/m^2^ preoperatively to 32.9 at 1 month, 30.6 at 3 months, 29.4 at 6 months, and 28.6 at 12 months. Mean %TBWL was 9.8 ± 2.5%, 16.0 ± 3.8%, 16.6 ± 6.4%, and 16.8 ± 6.6% at 1, 3, 6, and 12 months, respectively. %EWL was 29.9 ± 9% at 1 month, 48.9 ± 14.9% at 3 months, 53.3 ± 23.7% at 6 months, and 60.3 ± 31.7% at 12 months. At 1 month, all patients achieved ≥5% TBWL, with 45% achieving ≥10%. By 3 months, 96% reached ≥10% TBWL, and at 12 months, 78% achieved ≥10% and 67% ≥15%.

**Conclusion: **In this initial Turkish single-center series, ESG demonstrated significant weight reduction with a favorable safety profile, supporting its role as an effective endoscopic bariatric option in real-world practice.

Table 1.Demographic and Metabolic Parameters

Values are expressed as *n* (%), mean ± SD, or median (min-max).

BMI, body mass index; ESG, endoscopic sleeve gastroplasty; MASH, metabolic dysfunction-associated steatohepatitis.

**Table:** 

**Demographics**	**Patients (N = 29)**
Mean age (years) (SD)	39.1 ± 8
Gender, female [n (%)]	24 (82.8)
Basic weight parameters and comorbidities	
Weight, kg (SD)	101 ± 16.8
BMI, kg/m^2^ (SD)	36.6 ± 5.4
Hypertension, n	4
Diabetes mellitus, n	2
MASH, n	10
Psychiatric illness, n	1 (under control)
Hypothyroidism, n	4 (under control)
Transaction Information	
Number of sutures, median	5 (3-6) (5 sutures for 22 patients)
Suture pattern	U pattern (17)/interlocking ESG (12)
Number of patients at each time point	29 (1 month), 27 (3 months), 23 (6 months), 9 (12 months)

## SS-101 A Rare Case of Post-Pancreatitis Walled-off Necrosis

**Ümran Bahar, Ercan Tut, Aleyna Erdem, Fatma Nur Teke**

Department of Gastroenterology, Başakşehir Çam and Sakura City Hospital, İstanbul, Türkiye

Walled-off necrosis (WON) is a well-encapsulated necrotic collection that develops 4-6 weeks after acute necrotizing pancreatitis. In symptomatic cases, drainage is indicated, and endoscopic methods are less invasive compared to surgery. A 50-year-old male patient with a history of hypertension and diabetes mellitus presented to the emergency department with abdominal pain, nausea, and vomiting. Laboratory findings revealed a CRP of 158.1 mg/L, amylase of 860 U/L, and lipase of 178 U/L. Imaging showed a cystic collection in the perigastric region measuring up to 11 cm. Endoscopic ultrasound (EUS)-guided cystogastrostomy was performed, during which a double-pigtail stent was placed but subsequently migrated. In a second procedure, a lumen-apposing metal stent (LAMS) was implanted, the migrated stent was removed, and the necrotic material was cleared endoscopically. The patient underwent scheduled endoscopic debridement sessions and was discharged after clinical improvement. EUS-guided cystogastrostomy using LAMS is a safe and effective method for the treatment of WON. It allows for the drainage of both fluid and solid components of the collection and can be combined with endoscopic debridement when necessary.


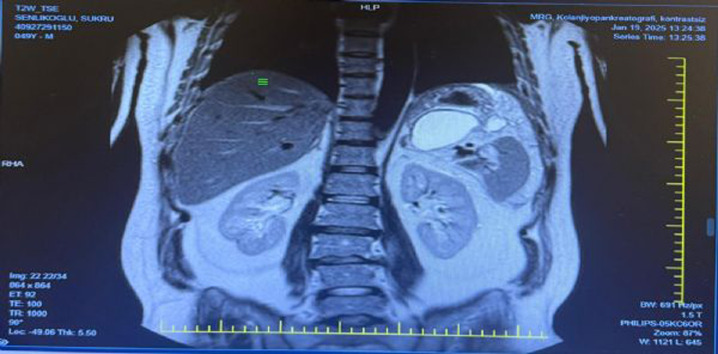


Figure 1. BT image.


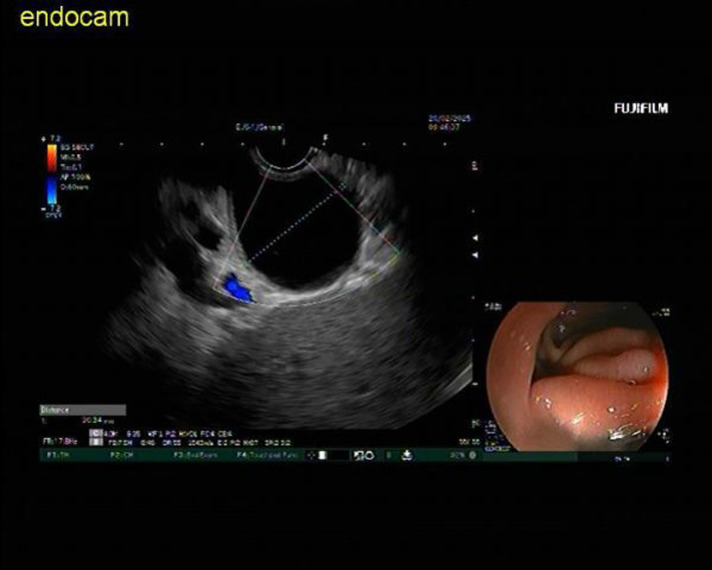


Figure 2. EUS image.

## SS-102 Minor Complication of Percutaneous Endoscopic Gastrostomy Tube: Tube Obstruction

**Güldan Kahveci ^1^ , Çiseli Altuntaş ^2^ , Nermin Mutlu Bilgiç ^3^ , Zerrin Dandin ^4^**

^1^Department of Nutritional Nursing, Ümraniye Training and Research Hospital, İstanbul, Türkiye

^2^Department of Endoscopy, Ümraniye Training and Research Hospital, İstanbul, Türkiye

^3^Department of Gastroenterology, Ümraniye Training and Research Hospital, İstanbul, Türkiye

^4^Department of Health Care Services, Ümraniye Training and Research Hospital, İstanbul, Türkiye

Percutaneous endoscopic gastrostomy (PEG) is a procedure to place a tube into the stomach to provide enteral nutrition for patients who cannot meet their nutritional needs. Although PEG placement is considered safe, it can cause minor complications such as hypergranulation tissue, pneumoperitoneum, tube obstruction, and peristomal infection. This case presents a patient whose blocked tube was cleared by administering sodium bicarbonate through it. A 59-year-old patient named MT underwent PEG placement in London 5 months ago due to difficulty swallowing. He was being fed via PEG tube. In July 2025, nutritional products were started for the patient in the neurology ward and this was reported upon discharge. During training, the patient was informed that nothing other than nutritional products, medication, and water should be administered through the PEG tube. The patient continued to administer other substances through the tube at home and presented to the endoscopy unit due to tube obstruction. To clear the blockage, the tube was massaged back-and-forth between the thumb and index finger. A measure of 10 mL of warm water and 1 ampule of sodium bicarbonate were drawn into the gavage syringe. This mixture was slowly administered through the tube with slight back-and-forth movement. After waiting 20-30 minutes for dissolution, the tube obstruction was resolved. There are various risk factors for tube blockage: tube length, small tube diameter, insufficient flushing during medication administration and/or dissolution, slow feeding flow rate, and high-calorie enteral nutrition products. To prevent tube blockage, at least 30 mL of water should be administered through the tube every 4 hours during continuous tube feeding, as well as before and after intermittent feedings. The tube should be flushed with 15 mL of water after each medication administration. After PEG placement, the healthcare personnel’s knowledge and practices regarding tube care and complications are critical for patients.

**SS-103 Endoscopic Intervention Due to Swallowing of Packaged Medication in the Upper Gastrointestinal System: A Case Report**

**Özge Bengü Şeniz ^1^ , Rafet Mete ^2^ , Nurten Türkel Küçükmetin ^2^**

^1^Tekirdağ University Hospital Endoscopy Unit, Tekirdağ, Türkiye

^2^Department of Gastroenterology, Tekirdağ University Faculty of Medicine, Tekirdağ, Türkiye

Swallowing foreign bodies is rare in adults and usually occurs due to psychiatric disorders, alcohol consumption, or accidental reasons. The ingestion of medication along with its aluminum packaging is a rare condition reported in the literature, with a high risk of complications. This case is noteworthy due to the ingestion of medication with its aluminum packaging and its subsequent endoscopic management. The patient is a 49-year-old woman with a graduate education. She regularly uses antihypertensive medication for hypertension and accidentally swallowed the medication along with its aluminum packaging. Her presenting complaint was mild retrosternal discomfort and difficulty swallowing. During the first endoscopy, the packaged medication was observed to be lodged in the upper and middle esophagus, and the foreign body was pushed into the stomach. However, since the stomach was full, the procedure was postponed to the second session. In the second endoscopy, hemorrhages, erosions, and superficial ulcers were detected in the antrum and prepyloric area. The packaged medication was found in the body region, and the sharp edges were smoothed with lithotripsy before being removed from the esophagus. Delayed endoscopic intervention may lead to serious complications, particularly hemorrhage, ulceration, and perforation. In the endoscopic removal of foreign bodies, techniques such as lithotripsy that reduce the sharpness of the object’s edges can help prevent mucosal trauma. Regardless of the individual’s education level, swallowing medication with its packaging can occur due to carelessness. Therefore, attention should be paid when using medications.

## SS-104 Management of a Patient with Skin Ulceration Due to Peristomal Leakage: A Case Report

**Güldan Kahveci ^1^ , Ekmel Burak Özşenel ^2^ , Nermin Mutlu Bilgiç ^3^ , Zerrin Dandin ^4^ , Sema Basat ^2^**

^1^Department of Nutritional Nursing, HÜmraniye Training and Research Hospital, İstanbul, Türkiye

^2^Department Internal Medicine, Ümraniye Training and Research Hospital, İstanbul, Türkiye

^3^Department of Gastroenterology, Ümraniye Training and Research Hospital, İstanbul, Türkiye

^4^Department of Health Care Services, Ümraniye Training and Research Hospital, İstanbul, Türkiye

Percutaneous endoscopic gastrostomy (PEG) involves the placement of a gastric tube to provide long-term enteral nutritional support. Complications following PEG are generally minor. Peristomal leakage can lead to infection and potentially result in tube loss. This case report discusses the evaluation and management of skin ulceration resulting from peristomal leakage. In this case, a PEG exchange tube was placed in an 84-year-old immobile female patient with a diagnosis of dementia in a nursing home 2 months ago. The patient was admitted to the gastroenterology ward due to issues such as leakage, redness, and frequent wetting of the dressing around the PEG. The medical history revealed that feeding was maintained through bolus administration, with no constipation reported, and the patient exhibited a cough. The patient was started on a proton pump inhibitor as prescribed by the physician. Dressings were applied twice daily by the nutrition nurse using zinc oxide cream and barrier cream. Risk factors for peristomal leakage include skin infection, increased gastric acid secretion, gastroparesis, distension, constipation, lateral tube rotation, increased tension between internal and external supports, buried bumper syndrome, and granulation tissue. Additionally, diabetes, immunosuppression, and malnutrition can delay wound healing. The causes of peristomal leakage should be identified, and the situation should be managed by taking the necessary precautions. In this case, skin ulceration resulting from peristomal leakage after PEG was successfully managed with accurate diagnosis and regular skin care. Training healthcare professionals working in the nursing home on PEG care, complications, and management will reduce complications and positively contribute to the long-term use of the PEG tube.


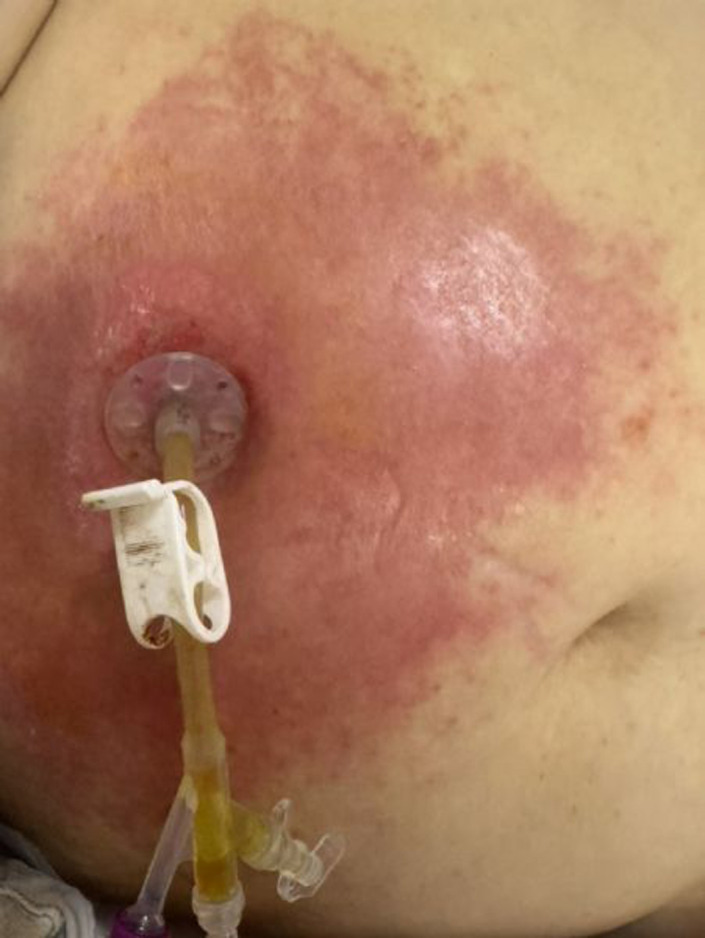


Figure 1. Peristomal leakage.


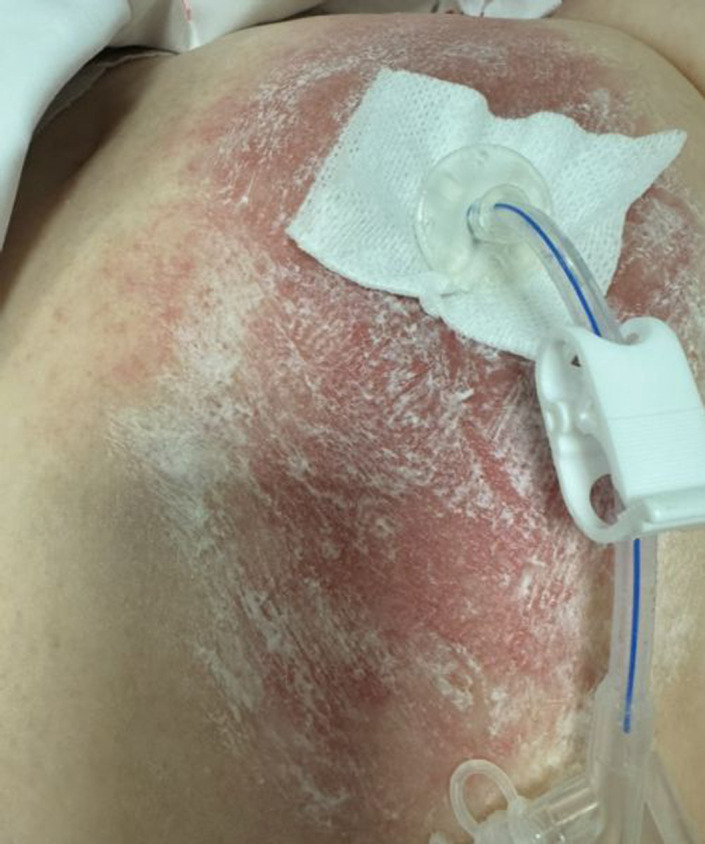


Figure 2. Care with barrier cream and cream containing zinc oxide.


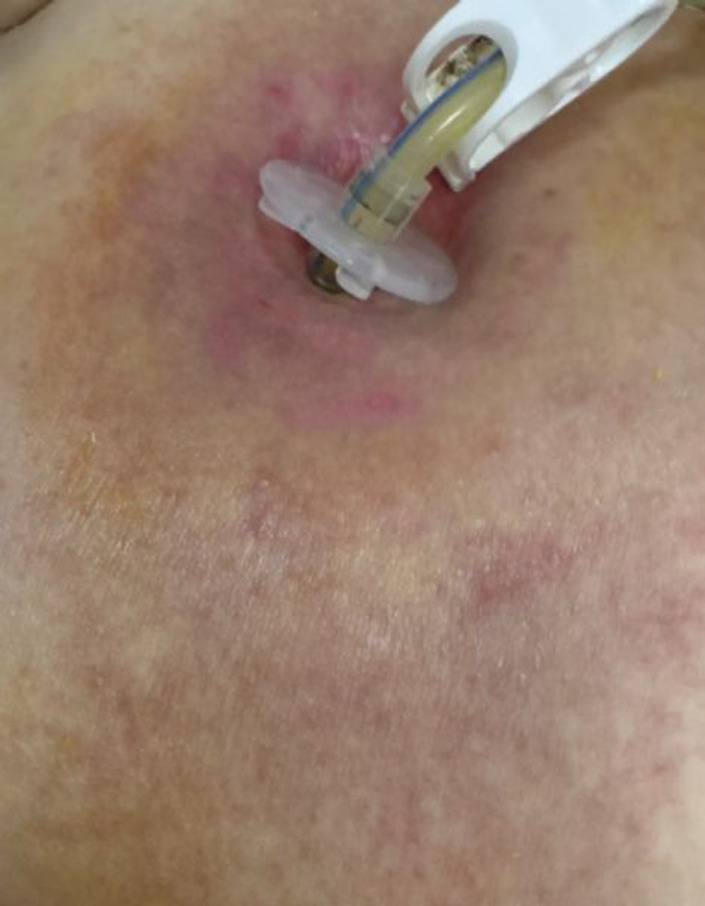


Figure 3. Skin appearance after 1 week.

## SS-105 Anxiety Levels in Patients Undergoing Endoscopic Retrograde Cholangiopancreatography at the Gastroenterology Clinic of İzmir City Hospital

**Vildan Keten, Filiz Sevin, Emre Büyükçapar, Ayten Parlak**

Department of Gastroenterology, İzmir City Hospital Endoscopy Unit, İzmir, Türkiye

**Background/Aims: **Endoscopic retrograde cholangiopancreatography (ERCP) is an invasive procedure frequently used in the diagnosis and treatment of biliary and pancreatic diseases. Its invasive nature, potential complications, and patient uncertainty may increase preprocedural anxiety. This study aimed to evaluate the effect of preprocedural information on anxiety levels in patients undergoing ERCP.

**Materials and Methods: **A total of 100 patients undergoing ERCP at the Endoscopy Unit of İzmir City Hospital were included. Fifty patients received preprocedural information, whereas the other 50 underwent ERCP without information. Anxiety levels were assessed using the State-Trait Anxiety Inventory (STAI) before the procedure.

Results: In the informed group, total anxiety scores ranged from 59 to 160 (mean = 93.18, SD = 18.12). In the uninformed group, scores ranged from 74 to 140 (mean = 102.06, SD = 16.31). Statistical analysis showed significantly lower anxiety scores in the informed group compared to the uninformed group.

**Conclusion: **Providing information prior to ERCP significantly reduces preprocedural anxiety. These findings highlight the importance of structured patient education not only for patient rights but also for improving patient comfort and treatment outcomes.

## SS-106 The Impact of Physical Environment Arrangements on Patients Perception of Embarrassment and Privacy in Colonoscopy Procedures at a Private Hospital

**Emel Kurşunbekiroğlu, Dilan Gökdemir, Gökhan Kabaçam**

Department of Gastroenterology, Güven Private Hospital, Ankara, Türkiye

**Background/Aims:** This study aimed to determine the effect of physical environment arrangements on feelings of embarrassment and perceptions of privacy during colonoscopy in a private hospital.

**Materials and Methods: **A descriptive design was used. The population included patients visiting Güven Hospital Gastroenterology Clinic between August 1 and September 15, 2025, who had previously undergone colonoscopy. The sample comprised patients who agreed to participate and met the criteria (n = 100). Data were collected via a Personal Information Form, Physical Environment Evaluation Form, and Colonoscopy Embarrassment Scale prepared by the researcher. Data were analyzed using SPSS 25.

**Results: **Among the 100 participants, 59% were female, and 62% were aged 40-60. Fifty-nine percent were married, and 60% were university graduates. Sixty-six percent had a chronic disease. All participants had prior colonoscopy experience and knowledge about the procedure. The mean score on the Colonoscopy Embarrassment Scale was 23.67 ± 6.402. No significant differences were found between embarrassment scores and age, marital status, education, chronic disease, previous colonoscopy, or prior information. However, gender was significantly associated with embarrassment levels (*P* = .045). According to the scale, 67% of patients were concerned about being awake during the procedure. In the physical environment evaluation, 74% reported that an appropriate environment influenced their feelings of embarrassment.

**Conclusion: **Patients often feel embarrassed about being awake and worry about insufficient bowel preparation. Female patients had higher embarrassment scores. Specialized healthcare staff should provide education, counseling, and nurse consultations. Scales can assess patients’ perceptions and embarrassment before the procedure. An adequate physical environment directly affects comfort and privacy, and staff should receive training in privacy protection. Improving the environment reduces embarrassment during colonoscopy.


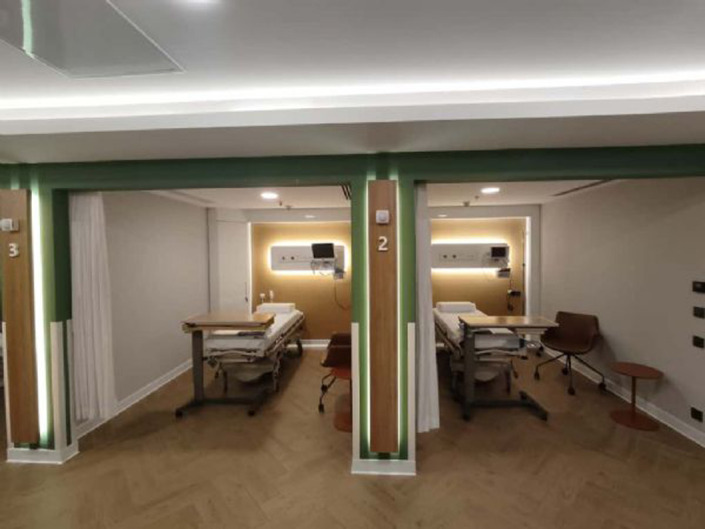



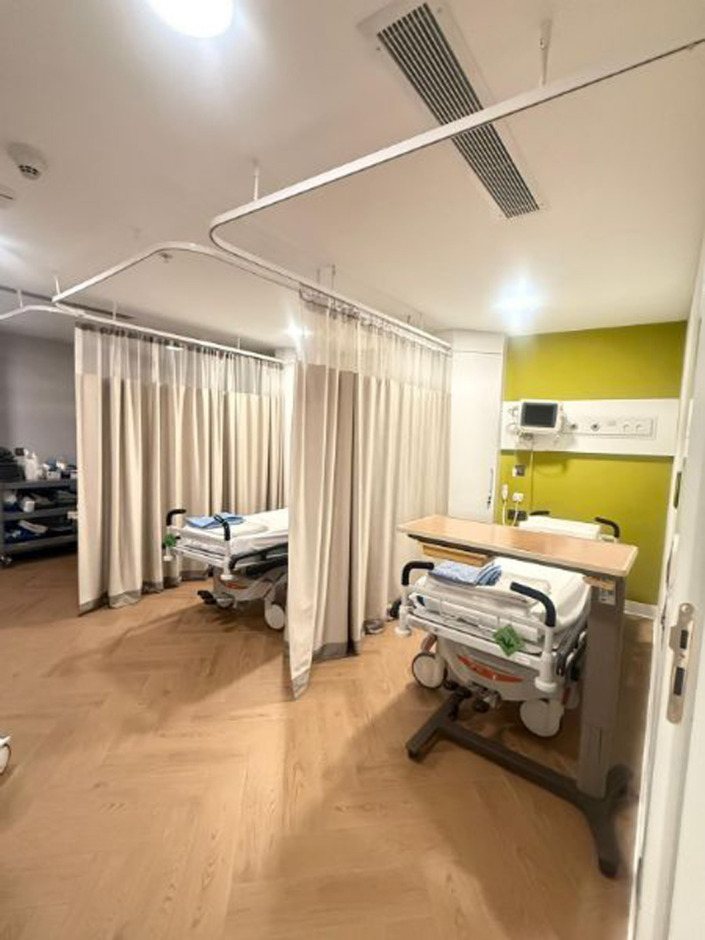


Figure 1. Physical environment.


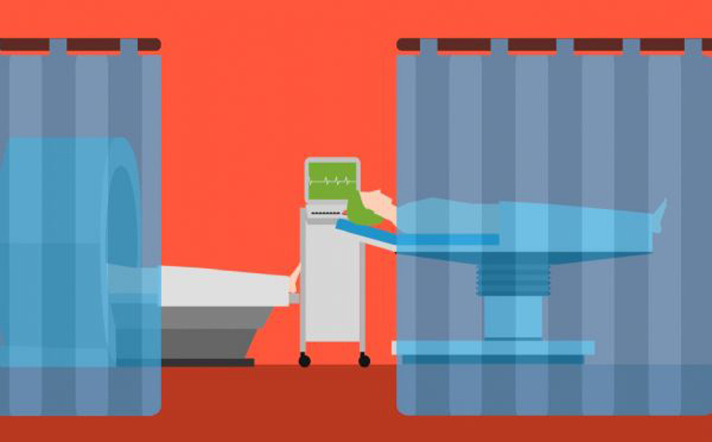


Figure 2. Patient privacy.

## SS-107 Determining the Burnout Levels of Endoscopy Nurses and Technicians in Relation to Encountered Risk Factors

**Tuğba Şenel, Yasemin Çakır, Dilek Öz, Saime Aydın Şahin, Asya Karaşin, Ramazan Mustafa Ekmekci**

Department of Endoscopy, Acıbadem Altunizade Hospital, İstanbul, Türkiye

**Background/Aims: **The steadily increasing occupational fatigue among healthcare professionals—arising from excessive demands and stressors in the workplace—leads to disruptions in bodily functions, cognitive performance, and overall work capacity. Consequently, healthcare workers are increasingly at risk of burnout syndrome. With the rapid advancement of endoscopic diagnostic and therapeutic technologies, the roles and competencies of endoscopy nurses have continuously expanded. Due to the complexity and specificity of the role, the detected rate of occupational fatigue among endoscopy nurses has reached 57.32%, resulting in reduced work efficiency, increased risk of errors and accidents, and emotional exhaustion—thereby affecting the quality of endoscopic diagnosis and treatment as well as patient safety. This study aimed to determine the level of burnout syndrome in relation to clinical risk factors among endoscopy nurses and technicians working in private and public hospitals and, based on the findings, to offer recommendations.

**Materials and Methods: **The study was designed as cross-sectional descriptive research and was conducted between April and May 2025 with 146 members of the Association of Gastrointestinal Endoscopy Nurses and Technicians. Data were collected online via SurveyMonkey by the principal investigator using an “Informed Consent Form,” “Demographic Data Form,” “Work Environment and Risk Assessment Form,” and the “Maslach Burnout Inventory (MBI).” Data analyses were performed in SPSS v25.0 using chi-square tests and descriptive percentages.

**Results: **Among participants, 91.1% were women; 40.4% were aged 41-50 years; and 76.0% were married. A total of 59.6% worked in public hospitals; 27.4% had ≥5 years of experience in endoscopy units. Based on participants’ responses, emotional exhaustion and depersonalization were at low levels, whereas personal and professional accomplishment were high.

**Conclusion: **This study reports that endoscopy nurses are prone to burnout due to workload and emotional pressures. Burnout can be mitigated through organizational arrangements, social support, and professional incentives.

## SS-108 Musculoskeletal Disorders and Ergonomic Solutions among Nurses Working in Endoscopy Units

**Burcu Türkoğlu**

Endoscopy Unit, Bezmialem Vakif University Hospital, İstanbul, Türkiye

**Background/Aims: **Nurses working in endoscopy units are at risk of musculoskeletal disorders (MSDs) due to prolonged standing, patient positioning, lifting heavy equipment, and repetitive movements. This study aimed to determine the prevalence of MSDs among nurses in endoscopy units and propose ergonomic solutions.

**Materials and Methods: **This descriptive and cross-sectional study was conducted with nurses working in endoscopy units (n = 100). Data were collected using a demographic information form, the Nordic Musculoskeletal Questionnaire (NMQ), and additional questions evaluating the perception of ergonomic risks. Pain severity was assessed using the Visual Analog Scale (VAS). Descriptive statistics (percentage, mean, SD) were used for the analysis.

Results: Seventy percent of the participants reported low back pain, 55% reported neck pain, 40% reported shoulder pain, and 35% reported wrist/hand pain. MSDs were more prevalent among nurses who stood for more than 6 hours a day. Patient handling and the weight of endoscopy equipment were the most frequently reported ergonomic risk factors. Nurses who had received ergonomics training reported fewer complaints. These findings are consistent with the literature. Previous studies reported that low back, neck, and shoulder pain were common among nurses. Similarly, another study emphasized that endoscopy nurses are exposed to significant ergonomic risks and that regular training can reduce such complaints.

**Conclusion: **MSDs are a significant health problem among endoscopy nurses. Ergonomic interventions (height-adjustable tables, transfer devices, job rotation) and regular training programs can help reduce these issues. Institutional policies should be developed to protect employee health.

## SS-109 Artificial Intelligence-Assisted Polyp Detection in Colonoscopy: Potential to Reduce Adenoma Miss Rates and Clinical Perspectives

**Mutlu Erdi Bilecen**

Department of Medical Services and Techniques, Hasan Kalyoncu University, Gaziantep, Türkiye

Colorectal cancer is among the most common malignancies worldwide, and the early detection and removal of polyps play a pivotal role in reducing mortality. However, operator dependency during colonoscopy may lead to missed polyps. Recently developed artificial intelligence (AI)-based computer-aided detection (CAD) systems have emerged as supportive tools for real-time polyp detection. This study aims to evaluate the impact of AI-assisted colonoscopy on adenoma detection rates (ADR) and clinical outcomes based on current literature.

SS-110 Endoscopic Vacuum-Assisted Treatment of Rectal Anastomotic Leaks and the Role of the Endoscopy Nurse in Treatment

**Hülya Avcı, Fikriye Arslan, Üsküdar Berkay Çaralan, Akın Bostanoğlu, Erdal Birol Bostancı**

Gastroenterology Surgery Clinic, Ankara Bilkent City Hospital, Ankara, Türkiye

Anastomotic dehiscence and leakage are observed in rectal cancer patients after surgery. Endoscopic methods are also effectively utilized in treatment management. This study aimed to investigate the application of endoscopic vacuum therapy and the role of the endoscopy nurse in this phase. Five patients who underwent surgery for rectal cancer at the Gastroenterology Surgery Department of Ankara Bilkent City Hospital between January 2024 and October 2025 and received endoscopic vacuum therapy for anastomotic dehiscence in the postoperative period were included in this study. Patient demographics and clinical findings were recorded. All patients had received neoadjuvant chemoradiotherapy. Diagnosis was made via rectoscopy on an average of the fourth postoperative day. In patients with anastomotic dehiscence, a vacuum sponge system was applied to the leak site during rectoscopy performed under sedation in the endoscopy unit. The vacuum catheter was changed every 2 days. After an average of 5 applications, the dehiscence was observed to shrink, and the abscess pouch healed. The vacuum system was prepared in the unit using sponge. During the postprocedure follow-up, patients were instructed on their sleeping position and precautions. Device usage instructions and potential problems were shared with the patients. Daily drainage rates from the vacuum system were monitored. Rectal anastomotic leaks can have life-threatening consequences. Endoscopic vacuum therapy can eliminate the need for reoperation. This method is easily implemented in endoscopy units. Nursing care is an essential part of this treatment.


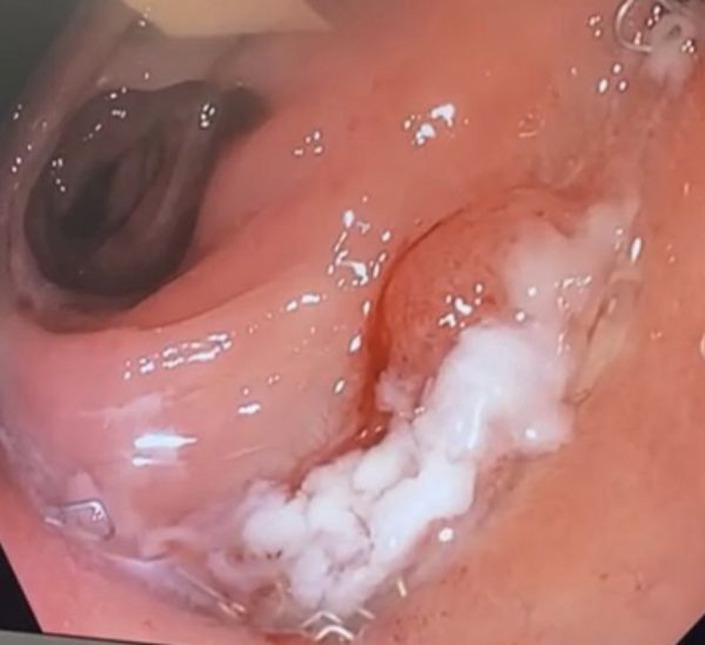


Figure 1. Appearance of healed anastomosis after endoscopic vacuum-assisted treatment.


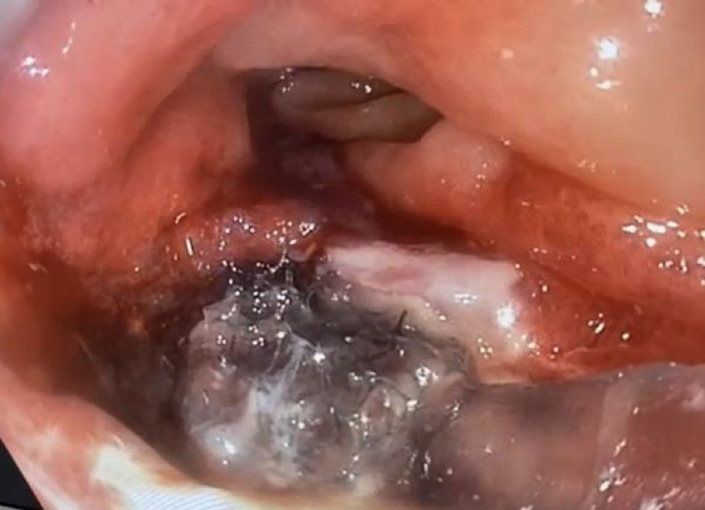


Figure 2. Endoscopic vacuum-assisted treatment of rectal anastomosis dehiscence.

## SS-111 Prevalence and Associated Characteristics of Steatohepatitis Linked to High-Risk Metabolic Dysfunction Identified by the FAST Score in Patients with Type 2 Diabetes

**Çağlayan Keklikkıran ^1^ , Melisa Melek Otrakçıer ^2^**

^1^Department of Gastroenterology, Recep Tayyip Erdogan University Faculty of Medicine, Rize, Türkiye

^2^Clinical Research Unit, Recep Tayyip Erdogan University, Rize Training and Research Hospital, Rize, Türkiye

**Background/Aims:** This study aimed to determine the prevalence of high-risk metabolic dysfunction-associated steatohepatitis (MASH) as identified by the noninvasive FAST score and to comparatively outline the clinical profile of this patient group in an adult cohort with type 2 diabetes mellitus (T2DM).

**Materials and Methods: **This retrospective, cross-sectional cohort study included 180 adult patients diagnosed with T2DM. Individuals with viral hepatitis, autoimmune liver diseases, or significant alcohol consumption were excluded. The cohort was divided into 2 groups based on the FAST score—a noninvasive index developed to detect MASH with significant fibrosis: the “high-risk MASH (+)” group (FAST ≥0.67, n = 54) and the “high-risk MASH (-)” group (FAST <0.67, n = 126).

**Results: **The prevalence of high-risk MASH in the entire patient population was 30%. No significant differences were observed between the groups regarding demographic data, including age, sex, body mass index, and waist circumference. However, HbA1c and non-HDL cholesterol levels were significantly higher in the high-risk MASH (+) group. This group also exhibited significantly lower platelet counts, increased ferritin levels, and higher liver enzymes. FibroScan measurements demonstrated that both liver steatosis, as measured by CAP, and liver stiffness, as measured by LSM, were significantly higher in the high-risk MASH (+) group. The prevalence of cirrhosis (F4) was also significantly higher in this group (85.1% vs. 45.2%).

**Conclusion: **This study revealed that 30% of adult patients with T2DM had high-risk MASH, identifiable by the noninvasive FAST score. This patient subgroup is characterized by more severe hepatocellular injury, higher HbA1c and non-HDL cholesterol levels, more pronounced fibrosis, and a substantially higher rate of cirrhosis. These findings suggest that the FAST score can serve as a valuable screening tool within the T2DM population for identifying patients at risk of progressive liver disease who may benefit most from pharmacological intervention.

Table 1.Demographic, Anthropometric, Laboratory, and FibroScan Measurement Characteristics of All Patients and High-Risk Metabolic Dysfunction-Associated Steatohepatitis (MASH) Groups

Results are presented as mean ± SD.

FAST score ≥0.67 was considered as high-risk MASH. CAP >257 dB/m indicated steatosis, LSM >5.8 kPa indicated fibrosis. F1 (mild fibrosis): 5.8-8.5 kPa, F2 (significant fibrosis): 8.5-9.5 kPa, F3 (advanced fibrosis): 9.5-12.5 kPa, F4 (cirrhosis): ≥12.5 kPa.

CAP, controlled attenuation parameter; HT, hypertension; LSM, liver stiffness measurement. BMI, body mass index; LDL → Low-Density Lipoprotein; HDL → High-Density Lipoprotein; ALT → Alanin Aminotransferaz; AST → Aspartat; Aminotransferaz; ALP → Alkalen Fosfataz; GGT → Gama Glutamil Transferaz; PT → Prothrombin Time; INR → International Normalized Ratio.

**Table:** 

**Parameter**	**All Patients (n = 180)**	**High-Risk MASH (−) (n = 126)**	**High-Risk MASH (+) (n = 54)**	***P***
Demographic				
Age (years)	59.5 ± 13.8	60.3 ± 14.7	57.5 ± 11.2	.220
Sex (male/female)	66/114	45/81	21/33	.685
HT, n (%)	135 (75)	94 (74.6)	41 (75.9)	.851
Anthropometric				
Obesity, n (%)	152 (84)	104 (82.5)	48 (88.8)	.281
BMI (kg/m^2^)	36.6 ± 6.5	36.2 ± 6.7	37.6 ± 5.8	.215
Waist circumference (cm)	115.3 ± 12.4	114.8 ± 13.3	116.4 ± 10.2	.433
Laboratory parameters				
Plasma glucose (mg/dL)	160.5 ± 67.6	156.7 ± 67.5	169.3 ± 67.7	.254
HbA1c (%)	8.6 ± 5.5	8.2 ± 4.6	10.7 ± 9	***.026***
Creatinine (mg/dL)	0.90 ± 0.30	0.91 ± 0.26	0.87 ± 0.39	.472
Hemoglobin (g/dL)	13.5 ± 1.7	13.9 ± 1.7	14 ± 1.6	.079
Platelet (10^3^/µL)	217.8 ± 75.7	230.6 ± 74.2	187.5 ± 71.1	***<.001***
Albumin (g/L)	4.25 ± 0.68	4.25 ± 0.71	4.24 ± 0.63	.885
Ferritin (ng/mL)	103.3 ± 164.8	73.3 ± 86.6	170.3 ± 256.1	***.001***
Total cholesterol (mmol/L)	212.3 ± 50.4	209.5 ± 49.7	219.4 ± 52.2	.273
LDL (mmol/L)	127.5 ± 41.5	123.1 ± 41.4	137.6 ± 40.3	.055
HDL (mmol/L)	49.1 ± 14.1	49.9 ± 14.4	46.9 ± 13.1	.239
Non-HDL (mmol/L)	174.3 ± 126.8	159.6 ± 47.9	211.1 ± 222.8	***.034***
Triglycerides (mmol/L)	219.4 ± 277.1	230.7 ± 321.9	191.5 ± 100.6	.430
Liver function tests				
ALT (U/L)	48.1 ± 36.1	34.5 ± 21.3	79.8 ± 42.9	***<.001***
AST (U/L)	43.4 ± 28.8	30.9 ± 13.9	72.6 ± 33.4	***<.001***
ALP (U/L)	97.8 ± 62.9	95.7 ± 71.9	102.6 ± 34.7	.542
GGT (U/L)	95.8 ± 105.4	76.8 ± 86.2	140 ± 131	***<.001***
Total bilirubin (mg/dL)	1.31 ± 5.97	1.50 ± 7.21	0.88 ± 0.45	.559
Direct bilirubin (mg/dL)	0.17 ± 0.14	0.16 ± 0.14	0.20 ± 0.14	.175
PT	13.5 ± 4.26	13.8 ± 4.9	13.0 ± 2.2	.353
INR	1.12 ± 0.38	1,140,44	1.08 ± 0.18	.399
FIB-4 score	2.15 ± 2.19	1.69 ± 1.15	3.22 ± 3.40	***<.001***
FAST score	0.50 ± 0.25	0.38 ± 0.19	0.80 ± 0.06	***<.001***
Fibroscan measurements				
CAP (dB/m)	292.2 ± 50.4	284.4 ± 48.2	310.3 ± 51.3	***.001***
Steatosis, n (%)	137 (76.1)	88 (69.8)	49 (90.7)	***.002***
LSM (kPa)	17.7 ± 11.9	14.1 ± 6.5	25.9 ± 16.6	***<.001***
F1/F2/F3/F4	9/26/42/103	9/25/35/57	0/1/7/46	***<.001***
Cirrhosis (F4), n (%)	103 (57.2)	57 (45.2)	46 (85.1)	***<.001***

## SS-112 Comparative Analysis of Recommendations Provided by Large Language Models in the Management of Esophageal Variceal Bleeding: A Descriptive Study

**Ferya Çelik ^1^ , Pırıl Akıncıoğlu ^2^ , Serkan Torun ^3^ , Haydar Adanır ^4^ , Hicran Bektaş ^1^ , Dinç Dinçer ^4^**

^1^Department of Internal Medicine Nursing, Akdeniz University Faculty of Nursing, Antalya, Türkiye

^2^Department of Gastroenterology, Karaman Training and Research Hospital, Karaman, Türkiye

^3^Department of Gastroenterology, Düzce University Faculty of Medicine, Düzce, Türkiye

^4^Department of Gastroenterology, Akdeniz University Faculty of Medicine, Antalya, Türkiye

**Background/Aims:** The aim of this study is to compare the recommendations provided by large language models (LLMs) in the management of patients with esophageal variceal bleeding.

**Materials and Methods: **This study was conducted in October 2025 using a comparative and descriptive research design. Based on a case scenario of esophageal variceal bleeding, the recommendations provided by 4 LLMs—ChatGPT, Gemini, DeepSeek, and Grok—were comparatively analyzed. The recommendations generated by the LLMs were evaluated based on the European Society of Gastrointestinal Endoscopy (ESGE) (2022) guidelines. The assessment focused on key domains: hemodynamic stabilization, timing of endoscopy, vasoactive therapy, antibiotic treatment, use of nonselective beta-blockers, blood transfusion, endoscopic band ligation, transjugular intrahepatic portosystemic shunt (TIPS) procedure, and use of proton pump inhibitors. The evaluations were conducted independently by 4 gastroenterologists using a 0-5 point scale. Statistical analyses were performed using Python software (version 3.13.3). Differences among the LLMs were analyzed using the Friedman test, whereas interrater agreement among experts was assessed with Kendall’s W coefficient. A significance level of *P* < .05 was considered statistically significant.

Results: When compared with the recommendations of the European Society of Gastrointestinal Endoscopy, ChatGPT achieved the highest mean score (4.31 ± 0.2) in the management of esophageal variceal bleeding. The performances of Gemini and Grok were found to be similar, whereas DeepSeek demonstrated the lowest performance among the evaluated models. It was determined that DeepSeek provided incomplete or inaccurate recommendations in domains such as TIPS indication and antibiotic use. The differences in mean scores among the LLMs were found to be statistically significant (*P* < .05). Post-hoc analyses revealed that ChatGPT outperformed the other models.

**Conclusion: **It may be suggested that although artificial intelligence-based applications can assist in the clinical implementation of evidence-based guidelines, they should not be used solely as tools for clinical decision-making.


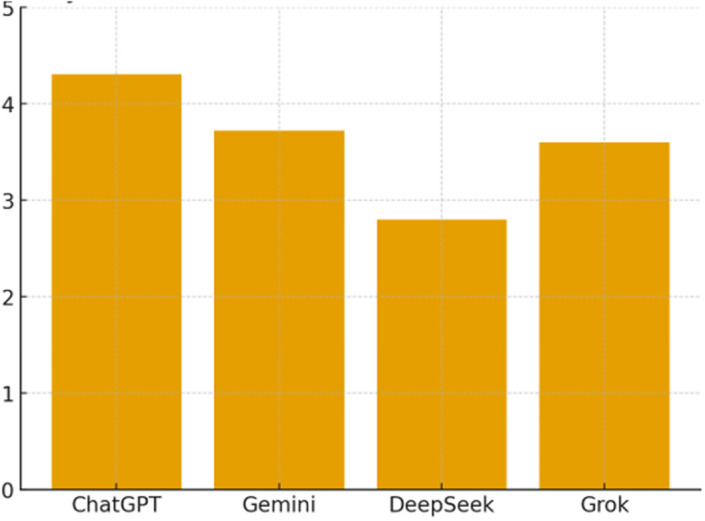


Figure 1. Comparison of the mean scores of large language models.

## TP-002 Serum Fibrosis Scores as Predictors of Liver Fibrosis and Long-Term Outcomes in Metabolic Dysfunction-Associated Steatotic Liver Disease, Including Cirrhosis

**Toprak Koçak ^1^ , Nilay Danış ^2^ , Hüseyin Döngelli ^1^ , Anıl Aysal Ağalar ^3^ , Göksel Bengi ^2^ , Mesut Akarsu ^2^**

^1^Department of Internal Medicine, Dokuz Eylül University Hospital, İzmir, Türkiye

^2^Department of Gastroenterology, Dokuz Eylül University Hospital, İzmir, Türkiye

^3^Department of Medical Pathology, Dokuz Eylül University Hospital, İzmir, Türkiye

**Background/Aims: **Metabolic dysfunction-associated steatohepatitis (MASH) is a common clinical condition characterized by fibrosis, which can progress to cirrhosis and liver failure. This study aimed to evaluate the predictive power of FIB4, APRI, and NFS scores—calculated using pre-biopsy laboratory data—for liver fibrosis in patients with MASH. Additionally, the development of de novo cirrhosis and survival after biopsy were analyzed.

**Materials and Methods: **This single-center, retrospective study included 175 patients with MASH. FIB4, APRI, and NFS scores were calculated in the pre-biopsy period. Fibrosis staging was performed based on liver biopsy. The predictive performance of these scores for advanced fibrosis (stages 3-4) was assessed using ROC analysis. Cox regression models were used to evaluate the development of de novo cirrhosis and survival.

**Results: **The mean age was 49.9 ± 14.1 years, and 54.9% of the patients were female. The median follow-up duration was 78 months. For predicting advanced fibrosis, FIB4 had an AUC of 0.77 with 85% sensitivity and 58% specificity; APRI had an AUC of 0.74 with 75% sensitivity; and NFS had an AUC of 0.74 with 68% sensitivity. Among 169 patients without cirrhosis at baseline, 25 developed de novo cirrhosis. In multivariate analysis, fibrosis stage at the time of biopsy (HR: 3.045; *P* = .001) and hypertension (HR: 4.096; *P* = .047) were identified as independent risk factors. In the survival analysis, age, albumin, and HbA1c levels were significantly associated with outcomes.

**Conclusion: **FIB4 emerged as the best predictor of advanced fibrosis in patients with MASH. Fibrosis stage and hypertension were significant factors in the development of de novo cirrhosis. HbA1c levels were significantly associated with survival. These findings highlight the importance of individualized follow-up and management of comorbidities in patients with MASH.

## TP-004 Evaluation of Sarcopenia Severity in Cirrhotic Patients According to Complications and Received Treatments

**Fatih Emin Öztürk, Oğuz Atar, Selçuk Eren Ekici, Memduh Şahin**

Başakşehir Çam and Sakura City Hospital, İstanbul, Türkiye

**Background/Aims:** This study aims to determine the severity of sarcopenia in patients with cirrhosis based on the treatment modalities applied and the presence of cirrhosis-related complications. The findings are expected to provide new insights into the diversity of treatment approaches for patients with cirrhosis.

**Materials and Methods: **In this study, 53 patients who presented to the gastroenterology clinic of the hospital between December 1, 2023, and December 1, 2024, were prospectively evaluated. The effect of diuretic use on sarcopenia in patients with cirrhosis was assessed. Muscle strength and mass were evaluated using DEXA, Jamar dynamometer, and the SARC-F score.

**Results: **There were no statistically significant differences in muscle strength, muscle mass, or functional sarcopenia scores among cirrhotic patients with different underlying etiologies. In patients using diuretics, the Jamar score was significantly lower, whereas the SARC-F score was significantly higher. In patients with ascites, Jamar and DEXA scores were significantly lower, whereas SARC-F scores were significantly higher. In the group with hepatic encephalopathy, DEXA scores were also significantly lower.

**Conclusion: **The findings of this study indicate that patients with cirrhosis who exhibit worse sarcopenia scores, reflecting reduced muscle mass and strength, tend to have more frequent and advanced cirrhotic complications. Therefore, the assessment of sarcopenia in patients with liver cirrhosis is of critical importance. Incorporating sarcopenia evaluation into the management of cirrhotic patients may enhance the understanding of disease prognosis and facilitate more accurate and effective treatment planning.

## TP-009 Assessment of Liver Stiffness by Transient Elastography in Heart Failure: A Cross-Sectional Study Linking Hepatic Congestion with Echocardiographic Findings

**Besim Fazıl Ağargün ^1^ , Mustafa Lütfi Yavuz ^2^ , Elif Ayduk Gövdeli ^2^ , İbrahim Volkan Şenkal ^1^ , Aynura Rustamzade ^1^ , Kanan Nuriyev ^1^ , Zülal İstemihan ^1^ , Ziya İmanov ^1^ , Sezen Genç Uluçeçen ^1^ , Gizem Dağcı ^1^ , Mehmet Akif Yağlı ^1^ , Asım Gurbanov ^1^ , Pelin Telli ^1^ , Sabuhi Mammadov ^1^ , Ersel Bilgin ^1^ , Bilger Çavuş ^1^ , Filiz Akyüz ^1^ , Kadir Demir ^1^ , Fatih Beşışık ^1^ , Sabahattin Kaymakoğlu ^1^ , Ekrem Bilal Karaayvaz ^2^ , Aslı Çifcibaşı Örmeci ^1^**

^1^Division of Gastroenterohepatology, Department of Internal Medicine, İstanbul University İstanbul Faculty of Medicine, İstanbul, Türkiye

^2^Department of Cardiology, İstanbul University İstanbul Faculty of Medicine, İstanbul, Türkiye

**Background/Aims: **Noninvasive assessment of hepatic congestion in heart failure remains challenging. Transient elastography (TE) quantifies liver stiffness (LSM), which may reflect right-sided filling pressures and structural remodeling. This study aimed to evaluate the relationship between LSM and echocardiographic parameters, particularly the severity of tricuspid regurgitation (TR) and right ventricular function, in patients with heart failure.

**Materials and Methods: **This cross-sectional study included consecutive outpatients with clinically diagnosed heart failure who underwent both transthoracic echocardiography and TE. The primary measurement was LSM (kPa). Echocardiographic parameters included tricuspid annular plane systolic excursion (TAPSE), systolic pulmonary artery pressure (SPAP), and TR grade. Correlations between LSM and echocardiographic variables were analyzed using Spearman’s rho, and differences in LSM across TR categories were tested with the Kruskal–Wallis method.

**Results: **Eighty-six patients were analyzed (mean age 60.6 ± 13.9 years, 56% male). Median LSM was 8.0 kPa (IQR, 5.5-20.5). LSM showed a positive correlation with TR grade (rho = 0.516, n = 76, *P* < .001) and SPAP (rho = 0.429, n = 72, *P* < .001), and a negative correlation with TAPSE (rho = −0.564, n = 51, *P* < .001). LSM increased progressively with higher TR grades (Kruskal–Wallis H = 29.46, *P* < .001).

**Conclusion: **In patients with heart failure, higher liver stiffness measured by transient elastography was associated with greater TR severity, higher SPAP, and lower TAPSE. These findings suggest that TE may serve as a simple, noninvasive tool to evaluate hepatic congestion and right-sided dysfunction in clinical practice.

## TP-010 Impact of Timing of Biological Therapy Initiation on Drug Persistence: A Retrospective Analysis in Inflammatory Bowel Disease

**Ayşe Yörük Öğüt ^1^ , Osman Çağın Buldukoğlu ^2^ , Tahir Saygın Öğüt ^3^ , Galip Egemen Atar ^2^ , Serkan Öcal ^2^ , Serdar Akça ^2^ , Ferda Akbay Harmandar ^2^ , Ayhan Hilmi Çekin ^2^**

^1^Department of Internal Medicine, Antalya Training and Research Hospital, Antalya, Türkiye

^2^Division of Gastroenterology, Antalya Training and Research Hospital, Antalya, Türkiye

^3^Division of Rheumatology, Antalya City Hospital, Antalya, Türkiye

**Background/Aims: **Biological therapies, particularly TNF-α inhibitors, have become central to treatment strategies in recent years. However, the effect of the timing of biological therapy initiation on disease activity, drug persistence, and clinical response remains unclear. This study aimed to evaluate the impact of the timing of biological therapy initiation on clinical outcomes.

**Materials and Methods:** Patients aged>18 years with IBD who received biological therapy and were followed at the gastroenterology clinic between January 2000 and December 2024 were retrospectively evaluated. Demographic, clinical, and laboratory data were collected.

**Results: **A total of 234 patients were included (163 CD, 71 UC). The mean age at diagnosis was 35.0 ± 13.0 years in CD and 35.5 ± 12.4 years in UC. Patients with UC had a longer follow-up duration and a longer median time from diagnosis to initiation of biological therapy. Among patients with CD, female sex and the presence of extraintestinal manifestations were significantly more common in those who received more than 1 biological agent (*P* = .010, *P* = .028).

Baseline disease activity was similar between early and late initiators: in CD, the median CDAI was 285 (190-380) in early initiators and 290 (200-370) in late initiators (*P* = .64). In UC, the median Mayo score was 9 (6-12) in early initiators and 9 (7-12) in late initiators (*P* = .71). CRP and ESR values also did not differ significantly between groups. The timing of biological therapy initiation (early vs. late) did not affect drug survival in either CD or UC. In CD, survival was 22.5 months when therapy was initiated within<12 months and 25.5 months when initiated >36 months after diagnosis (*P* = .867). In UC, survival was 7 months for ≤12 months, 52 months for 13-24 months, and 16.5 months for>36 months, with no statistically significant difference (*P* > .05). Prior use of conventional therapies did not significantly influence drug survival.

**Conclusion: **The interval between diagnosis and initiation of biological therapy does not affect disease activity or drug survival in either CD or UC. These results emphasize that treatment decisions should prioritize individualized risk profiles and the presence of extraintestinal manifestations rather than focusing solely on the timing of biological therapy initiation.


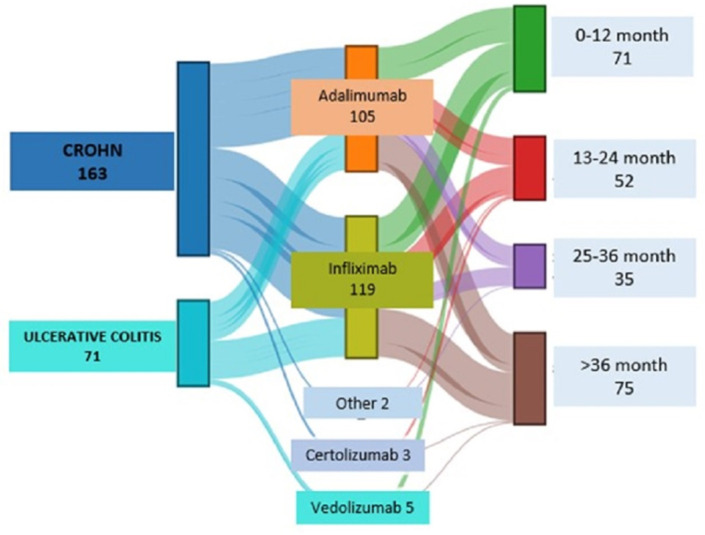


Figure 1. Sankey diagram showing initial biologic agent preferences and persistence of the first biologic agent in patients with IBD.

## TP-013 The Role of Helicobacter pylori in Patients with Gastroesophageal Reflux Disease and Its Effect on Esophageal Manometry and 24-Hour pH Monitoring Test Results

**Aslı Çiftçi ^1^ , Taylan Metin ^2^ , Mehmet Ali Şahan ^2^ , Ayhan Balkan ^2^**

^1^Department of Internal Medicine, Gaziantep University School of Medicine, Gaziantep, Türkiye

^2^Department of Gastroenterology, Gaziantep University School of Medicine, Gaziantep, Türkiye

**Background/Aims: **Gastroesophageal reflux disease (GERD) is characterized by symptoms resulting from the reflux of stomach contents into the esophagus. This study determined the role of *H. pylori* in GERD and evaluated its relationship with esophageal manometry and 24-hour impedance pH meter results.

**Materials and Methods: **This study assessed the role of *H. pylori* in patients with gastroesophageal reflux and evaluated its relationship with esophageal manometry and 24-hour impedance pH meter results. This study included 253 patients with clinical reflux who were admitted to the hospital between November 2020 and November 2023. The patients were then divided into 2 groups: *H. pylori* positive and negative.

**Results: **The number of patients diagnosed with endoscopic esophagitis was significantly higher in the *H. pylori*-negative group (*P* = .04). The incidence of hiatal hernia and lower esophageal sphincter relaxation was also significantly higher in the *H. pylori*-negative group (*P* = .03, *P* = .01, respectively). Grade A esophagitis was more common in the *H. pylori*-negative group, whereas the fewest cases were Grade D esophagitis. No significant difference was observed between the 24-hour impedance pH meter findings in the *H. pylori*-positive and -negative groups. When these patient groups were analyzed by mean age, the mean age in the hypersensitive esophagus group was significantly younger. Higher distal delay (DL) and fewer acid reflux episodes were found in the *H. pylori*-negative patient group with hypersensitive esophagus.

**Conclusion: ***H. pylori* infection is not associated with endoscopic esophagitis, hiatal hernia, or LES flaccidity in patients with GERD. Furthermore, *H. pylori* infection has no effect on 24-hour impedance pH and esophageal manometry results in patients with GERD.

## TP-014 Relationship of Disease Activation and Treatment with Pulmonary Function Tests and Obstructive Sleep Apnea Syndrome in Ulcerative Colitis Patients

**Halil Emek ^1^ , Taylan Metin ^2^ , Mehmet Ali Şahan ^2^ , Ayhan Balkan ^2^**

^1^Department of Internal Medicine, Gaziantep University School of Medicine, Gaziantep, Türkiye

^2^Department of Gastroenterology, Gaziantep University School of Medicine, Gaziantep, Türkiye

**Background/Aims: **Ulcerative colitis (UC) is a chronic disease characterized by relapses and remissions and may also be associated with extraintestinal pathologies. Patients may develop anemia, which can cause shortness of breath and limitations in exercise capacity. The aim of this study was to compare patients with UC with healthy subjects and to determine the relationship between disease presence, activation status, treatment, pulmonary function, and obstructive sleep apnea syndrome (OSAS).

**Materials and Methods: **The study included 55 patients with UC admitted to the inflammatory bowel diseases (IBD) outpatient clinic of Gaziantep University School of Medicine within the last year and 55 healthy controls. Disease activation and endoscopic evaluation were based on the Mayo score. Pulmonary function tests, lung diffusion tests (DLCO), MMRC scores, and 6-minute walk tests were performed in all participants. In the Berlin Questionnaire, patients were questioned about the frequency and severity of snoring, the frequency of diagnosed apnea, and the presence of hypertension or obesity.

**Results: **MMRC scores were significantly higher in patients with UC compared to the control group (*P* = .001). According to the Berlin questionnaire, 56.4% of patients with UC were at high risk for OSAS, whereas this rate was 30.9% in the control group (*P* = .007). FEV1 values were higher in patients with UC who received biological agents compared to those who did not (*P* = .041). As the endoscopic Mayo score increased, patients were found to be at higher risk for OSAS according to the Berlin questionnaire (*P* = .002).

**Conclusion: **Patients with UC have poorer respiratory function outcomes and a higher risk of OSAS compared to healthy individuals. Additionally, severe disease and high Mayo scores in subgroups of patients with UC adversely affect these parameters. Therefore, the assessment of pulmonary function and OSAS risk in patients with UC should be a routine part of patient management.

## TP-017 Investigation of the Effect of Humic Acid on Experimental Copper Accumulation in the Liver, Kidney, and Brain in Rats

**Yasir Furkan Çağın ^1^ , Yahya Atayan ^1^ , Onural Ozhan ^2^ , Ilhami Berber ^3^ , Azibe Yildiz ^4^ , Feyzi Doğru ^5^ , Yusuf Kirec ^2^**

^1^Department of Gastroenterology, İnönü University Medical Faculty, Malatya, Türkiye

^2^Department of Medical Pharmacology, İnönü University Medical Faculty, Malatya, Türkiye

^3^Department of Hematology, İnönü University Medical Faculty, Malatya, Türkiye

^4^Department Histology and Embryology, İnönü University Medical Faculty, Malatya, Türkiye

^5^Department of Physiology, İnönü University Medical Faculty, Malatya, Türkiye

**Background/Aims: **An affordable oral chelator is needed to reduce treatment costs in chronic copper intoxication, such as Wilson’s disease. Humic acid (HA), a naturally occurring molecule in water and soil with electron transfer ability, can eliminate toxic compounds. This study aimed to demonstrate the chelator-antioxidant effect of HA against copper-induced hepatotoxicity, nephrotoxicity, and neurotoxicity.

**Materials and Methods:**

Group I (control, n = 10): normal diet for 14 days

Group II (HA, n = 10): normal diet + HA 536 mg/kg/day po for 14 days

Group III (Cu, n = 10): normal diet + copper sulfate 75 mg/kg/day po for 14 days

Group IV (Cu+HA, n = 10): normal diet + HA (536 mg/kg/day, po, evening) + copper sulfate 75 mg/kg/day po for 14 days.

Blood, liver, kidney, and brain tissues were collected for biochemical and histopathological analyses.

**Results: **In the CuSO_4_ group, serum copper levels increased and ceruloplasmin levels decreased (*P* < .05). Copper accumulated in the liver, brain, and kidneys (*P* < .01). In the Cu+HA group, these values significantly decreased (*P* < .05). MDA and TOS levels were increased in CuSO_4_ rats (*P* < .01) but decreased with HA treatment (*P* < .05). Antioxidant parameters declined in CuSO_4_ rats (*P* < .01) and partially recovered with HA. Histopathology revealed hepatocyte injury, tubular degeneration, epithelial necrosis, and neuronal degeneration in CuSO_4_ rats, with milder effects observed in the Cu + HA group. Caspase-3 and caspase-9 levels increased in CuSO_4_ rats (*P* < .01) and decreased with HA treatment (*P* < .05).

**Conclusion: **In this rat model, copper-induced oxidative stress in the liver, kidney, and brain was demonstrated biochemically and histopathologically. HA, as a chelator and antioxidant, exhibited protective effects. Further studies are required to confirm its protective and therapeutic potential.

## TP-019 Assessment of Fibrosis in Chronic Hepatitis B Using APRI, FIB-4, FIB-5, FIB-6, and S-Index: Comparison with Histology

**Evrim Kahramanoğlu Aksoy ^1^ , Mehmet Raşit Ayte ^1^ , Harun Çobanoğlu ^2^ , Fırat Korlaelçi ^3^ , Metin Uzman ^1^ , Seçkin Özgül ^1^ , Haluk Cihad Albayrak ^1^ , Müzeyyen Burcu Kaplan Yılmaz ^4^ , Sanlı Arabacı ^1^ , Kerem İzmirlioğlu ^1^ , Bora Aktaş ^1^**

^1^Department of Gastroenterology, University of Health Sciences, Ankara Atatürk Sanatory Training and Research Hospital, Ankara, Türkiye

^2^Department of Internal Medicine, University of Health Sciences, Ankara Atatürk Sanatory Training and Research Hospital, Ankara, Türkiye

^3^Department of Gastroenterology, University of Health Sciences, Mehmet Akif İnan Training and Research Hospital, Şanlıurfa, Türkiye

^4^Department of Pathology, University of Health Sciences, Ankara Atatürk Sanatory Training and Research Hospital, Ankara, Türkiye

**Background/Aims: **Chronic hepatitis B (CHB) remains a leading global cause of cirrhosis and hepatocellular carcinoma. Although liver biopsy is the reference standard for fibrosis staging, its invasiveness has led to the development of noninvasive serum-based indices. This study aimed to compare APRI, FIB-4, FIB-5, FIB-6, and S-index with histology in patients with CHB.

**Materials and Methods:** One hundred ninety-one patients with CHB who underwent liver biopsy between 2012 and 2024 were retrospectively analyzed. Fibrosis was staged using the METAVIR system and classified as nonsignificant (F0–F2) or significant (F3–F4). Serum indices were calculated, and their diagnostic performance was evaluated using receiver operating characteristic (ROC) analysis.

**Results: **Of the 191 patients, 75 (39.3%) had significant fibrosis. APRI, FIB-4, FIB-6, and S-index were significantly higher in patients with significant fibrosis, whereas FIB-5 lacked discriminatory value. ROC analysis demonstrated modest overall accuracy. APRI (AUC 0.661) achieved the highest sensitivity (88.0%) but low specificity (38.8%), whereas FIB-4 (AUC 0.601) and FIB-6 (AUC 0.608) were more specific (86.2% and 87.1%, respectively) but less sensitive. S-Index (AUC 0.601) exhibited moderate performance with relatively balanced sensitivity and specificity.

**Conclusion: **Serum-based indices can aid in differentiating significant from nonsignificant fibrosis in CHB, though their diagnostic accuracy is limited. APRI appears most suitable for screening due to its high sensitivity, whereas FIB-4 and FIB-6 may serve better as confirmatory tools. S-Index demonstrated modest but proportionate diagnostic performance. Further validation in larger, multicenter prospective studies is warranted.


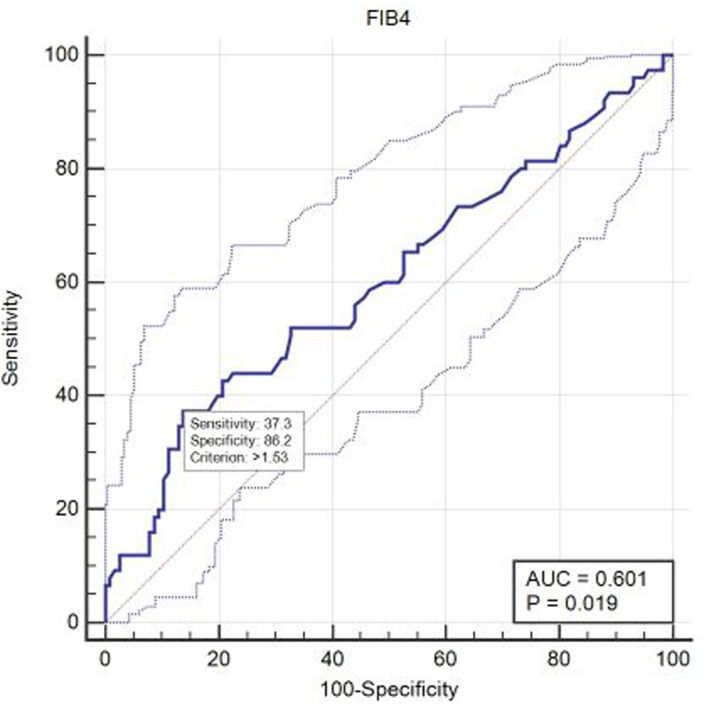


Figure 1. Receiver operating characteristic (ROC) analysis curves. ROC curve images of FIB-4.


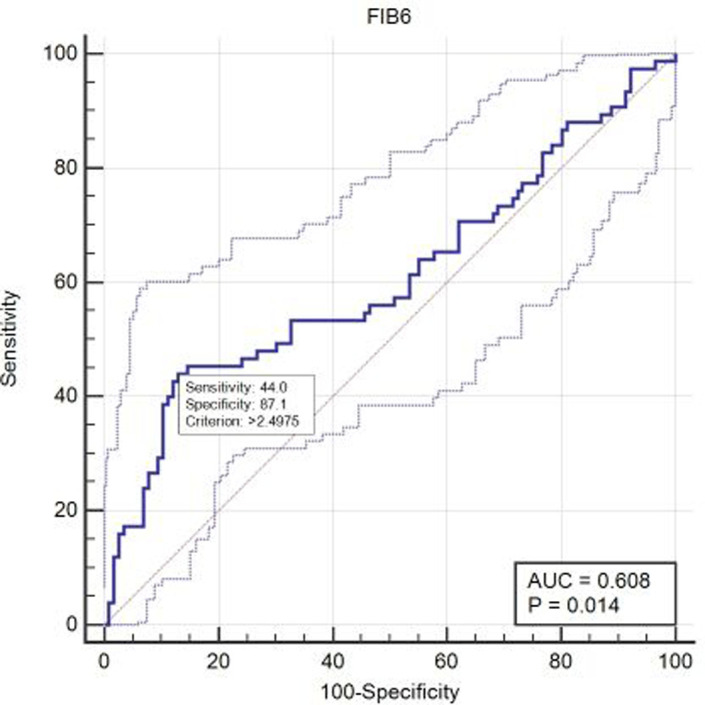


Figure 2. Receiver operating characteristic (ROC) analysis curves. ROC curve images of FIB-6.


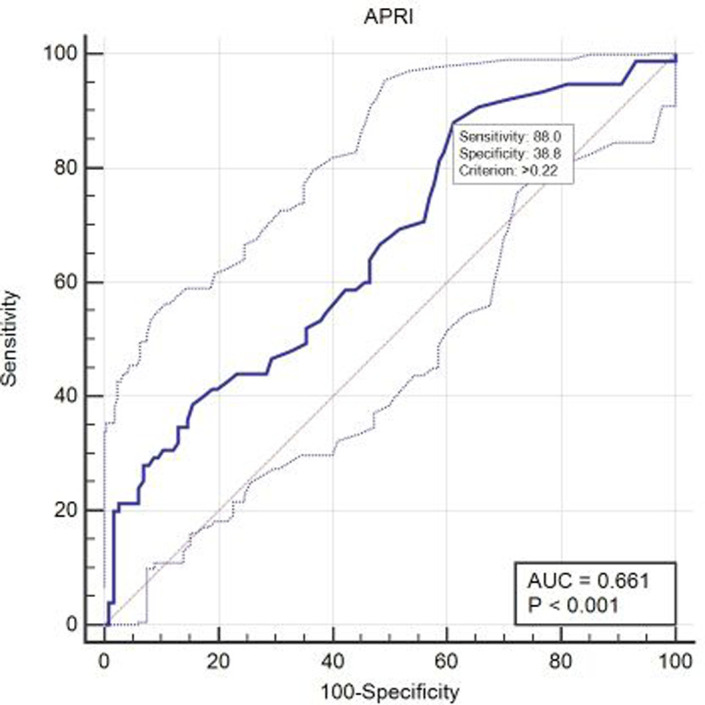


Figure 3. Receiver operating characteristic (ROC) analysis curves. ROC curve images of APRI.

## TP-021 Inflammatory Bowel Disease in Transgender Individuals: Clinical Course After Gender-Affirming Hormone Therapy—A Three-Case Series

**Bengi Öztürk ^1^ , Seda Hanife Oğuz Baykal ^2^ , Taylan Kav ^1^**

^1^Department of Gastroenterology, Hacettepe University Medical Faculty, Ankara, Türkiye

^2^Department of Endocrinology, Hacettepe University Medical Faculty, Ankara, Türkiye

Management of inflammatory bowel disease in transgender individuals is influenced not only by immunological mechanisms but also by gender-affirming hormone therapies, psychosocial factors, and treatment adherence. The clinical courses of 3 transgender individuals with IBD after hormone therapy are presented. Two had Crohn’s disease (a 25-year-old trans man and a 28-year-old trans woman), and 1 had ulcerative colitis (a 26-year-old trans man). Ages at diagnosis were 20, 22, and 15. The main presenting complaints were weight loss and diarrhea. The trans man with CD had diffuse ileocolonic and esophageal involvement; the trans woman had involvement of the terminal ileum and ascending colon; the UC case had pancolitis. All had a history of corticosteroid use; in the UC case, 1 year of steroid treatment caused iatrogenic Cushing’s syndrome. After withdrawal, azathioprine was initiated, followed by biologic therapy (infliximab, ustekinumab). All were active smokers. Family history revealed IBD in the trans man with CD and colorectal cancer in the others. Gender-affirming hormone therapy began after diagnosis, during remission, at ages 20, 27, and 20. The trans man with CD discontinued conventional therapy and remained in remission for 1 year but later developed sigmoid colon involvement and anovaginal fistula after appendectomy; infliximab and azathioprine were then started. During the treatment-free period with testosterone, no disease activation occurred; stopping testosterone at the initiation of biologic therapy did not reduce disease activity but increased dysphoria. Even after hormone cessation, flares and hospitalizations persisted. The trans woman with CD remained in remission for 3 years under infliximab but later switched to ustekinumab due to psoriasis; despite the use of estradiol and cyproterone acetate, no disease activation occurred. The trans man with UC discontinued ustekinumab on his own; testosterone use was not linked to increased disease activity. Difficulties in gender affirmation, psychiatric comorbidities, limited physician knowledge, and nonadherence complicate IBD management. A multidisciplinary approach is critical. Hormonal and immunomodulatory/biologic therapies, along with psychosocial factors, may shape long-term outcomes.

## TP-022 Evaluation of Alexithymia Levels in Patients with Ulcerative Colitis: A Single-Center Experience

**Beyza Atay, Bengi Öztürk, Muhammed Furkan Çakmak, Taylan Kav**

Division of Gastroenterology, Department of Internal Medicine, Hacettepe University Faculty of Medicine, Ankara, Türkiye

**Background/Aims: **The aim of this study was to evaluate alexithymic traits—defined as difficulties in recognizing, expressing, and communicating emotions—in patients diagnosed with ulcerative colitis during periods of active disease.

Materials and Methods: Patients with ulcerative colitis who were followed at the Department of Gastroenterology, Hacettepe University, and were experiencing active disease were administered the 20-item Toronto Alexithymia Scale adapted into Turkish. Alexithymia levels were determined using the scale and evaluated alongside demographic and clinical data.

**Results: **The study included 13 patients with ulcerative colitis, with a median age of 38 years (range, 23-76). Eight patients had extensive colitis, 4 had left-sided colitis, and 1 had proctitis. Seven patients were receiving biologic therapy, whereas 6 were on conventional agents. Mayo scores were 2 in 4 patients and 3 in 9 patients. None had a history of psychiatric illness. The median alexithymia score was 67 (range, 46-76). Three patients had no alexithymia, 2 had possible alexithymia, and 8 showed definite alexithymia.

**Conclusion: **Alexithymia levels were evaluated in patients diagnosed with ulcerative colitis and found high levels of alexithymic traits in a substantial proportion. The findings suggest that difficulties in recognizing and expressing emotions may be common during active phases of chronic inflammatory diseases such as ulcerative colitis. These results highlight the need to integrate psychosocial evaluations and, when necessary, psychological support interventions into the treatment process for this patient group.

## TP-023 Efficacy and Safety of Vedolizumab and Ustekinumab Treatment in Anti-Tumor Necrosis Factor–Exposed Inflammatory Bowel Disease Patients

**Narmin Naghizada ^1^ , Tuğba Tolu Bülte ^2^ , Yeşim Özen Alahdab ^2^ , Özlen Atuğ ^2^ , H . Tarık Kani ^2^**

^1^Department of Internal Medicine, Marmara University Faculty of Medicine, İstanbul, Türkiye

^2^Division of Gastroenterology, Marmara University Faculty of Medicine, İstanbul, Türkiye

**Background/Aims: **Patients with inflammatory bowel disease (IBD) who fail anti-tumor necrosis factor (anti-TNF) therapy require alternative biologics with different mechanisms of action. Vedolizumab (VDZ) and ustekinumab (UST) are established effective options; however, real-world comparative data remain limited.

**Materials and Methods: **A retrospective, single-center study of 114 anti-TNF-experienced patients with IBD (40 ulcerative colitis (UC), 74 Crohn’s disease (CD)) treated with VDZ or UST was conducted between 2017 and 2024. Clinical and laboratory parameters were collected at baseline, 3 months, 12 months, and last follow-up. Disease activity was assessed using the partial Mayo Score (UC) and Harvey-Bradshaw Index (CD). Treatment response, persistence, adverse events, and need for surgery were analyzed.

**Results: **Of 40 patients with UC, 34 (85%) were treated with VDZ and 6 (15%) with UST. Among 74 patients with CD, 34 (46%) were treated with VDZ and 40 (54%) with UST. In UC, VDZ led to significant reductions in pMayo scores at 3 and 12 months (*P* < .0001), whereas UST showed numerical improvement without statistical significance. In CD, both VDZ and UST significantly reduced HBI scores at 3 and 12 months (*P* < .001). Treatment persistence did not differ significantly between VDZ and UST in the overall, UC, or CD cohorts. Adverse events occurred in 9 patients (7.9%), mostly mild, with no serious complications. Surgical interventions were required in 9 patients, most of whom were treated with VDZ.

**Conclusion: **Both agents were effective and safe in anti-TNF-experienced IBD patients. The real-world data indicate distinct response patterns between UC and CD, highlighting the clinical utility of both agents as therapeutic options after anti-TNF failure.


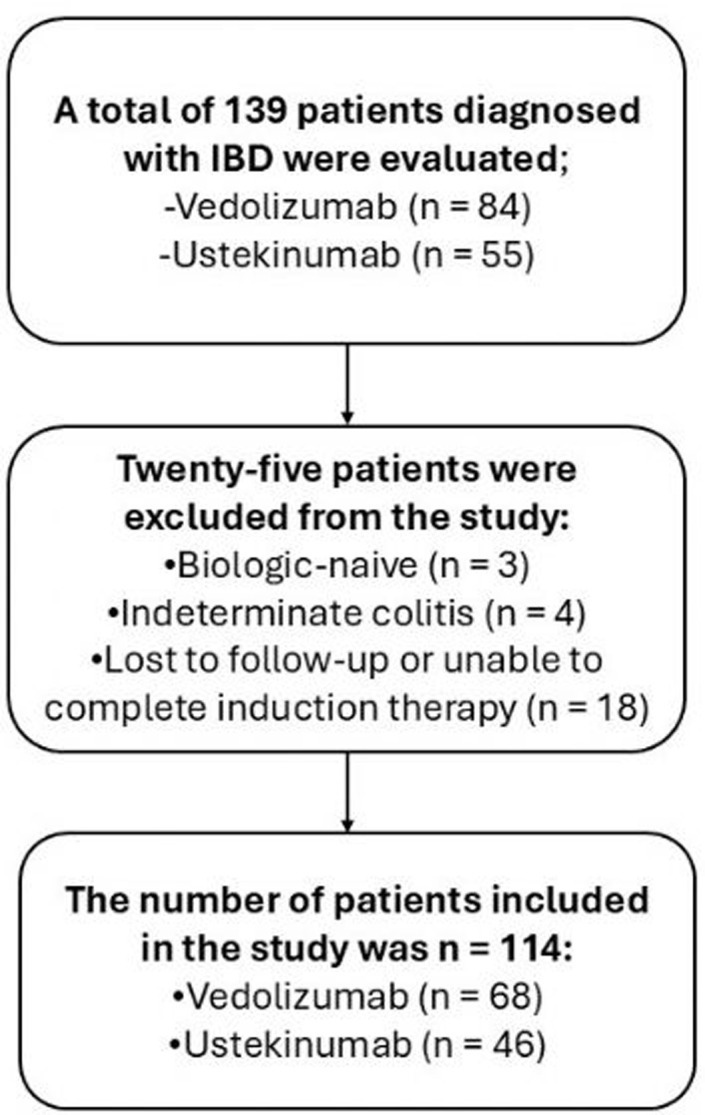


Figure 1. Flow diagram of patient selection.


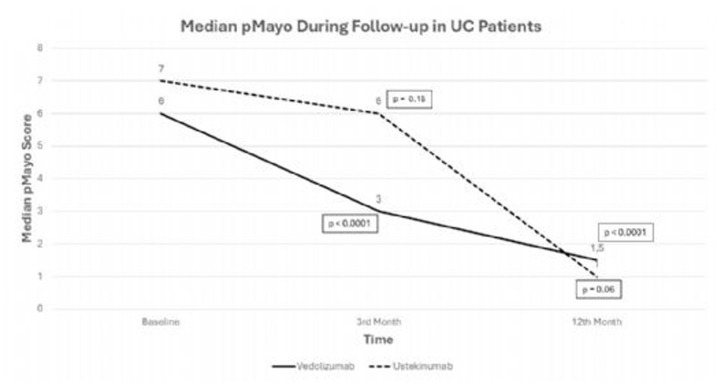


Figure 2. Median partial Mayo (pMayo) scores over time in patients with ulcerative colitis (UC) treated with vedolizumab or ustekinumab.


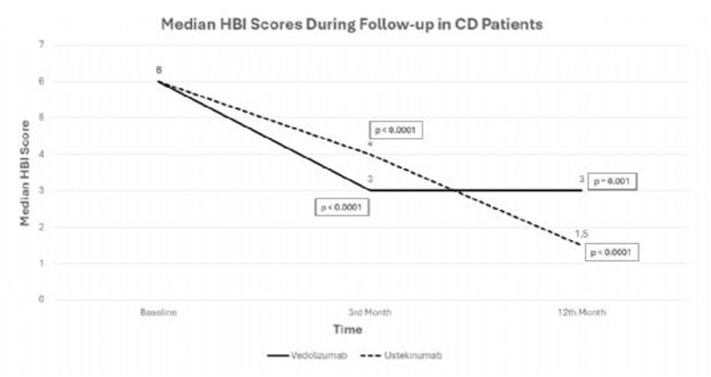


Figure 3. Median Harvey-Bradshaw Index (HBI) scores over time in patients with Crohn’s disease (CD) treated with vedolizumab or ustekinumab.

## TP-025 Investigation of the Frequency of Hepatocellular Carcinoma in Patients Followed Up with a Diagnosis of Hepatitis B

**Fatma Zehra Agan ^1^ , Feride Ebru Kayan ^1^ , Ahmet Uyanıkoğlu ^2^**

^1^Department of Internal Medicine, Harran University Faculty of Medicine, Şanlıurfa, Türkiye

^2^Division of Gastroenterology, Department of Internal Medicine, Harran University Faculty of Medicine, Şanlıurfa, Türkiye

**Background/Aims: **This study aimed to determine the risk of developing hepatocellular carcinoma (HCC) in patients diagnosed with hepatitis B, investigate the characteristics of patients with HCC, and compare the findings with literature data.

Materials and Methods: Patients diagnosed with hepatitis B and followed at the Division of Gastroenterology, Harran University Hospital between January 2014 and December 2022 were screened for HCC development. A total of 1275 patients meeting the inclusion criteria were enrolled, and 60 patients diagnosed with HCC were evaluated in detail. Cancer staging, treatment modalities, and surveillance were assessed, and data were analyzed using SPSS 24.0.

**Results: **Among the 1275 patients, 525 (41.2%) were female and 750 (58.8%) male, with a mean age of 40.92 ± 14.78 years. Disease duration averaged 79.01 ± 63.05 months. Of these, 674 (52.9%) were inactive hepatitis B carriers, 338 (26.5%) had HBeAg-negative chronic hepatitis B, 84 (6.6%) had HBeAg-positive chronic hepatitis B, and 179 (14%) had HBV-related cirrhosis. HCC was detected in 60 patients (4.7%). The HCC group included 11 females (18.3%) and 49 males (81.7%) with a mean age of 61.87 ± 11.82 years. Comorbidities were present in 43.3%, with diabetes mellitus being the most common. Delta antibody was positive in 10%, and anti-HCV in 3.3%. Cirrhosis was present in 90% of HCC cases. Hepatic steatosis and gallstones were detected in 8.3% and 11.7%, respectively. Tumor characteristics showed that 66.7% had a single lesion, with an average lesion size of 5.63 ± 3.44 cm. Treatments included transarterial chemoembolization (18.3%), radiofrequency ablation (3.3%), sorafenib (6.7%), liver transplantation (16.7%), and 55% were followed conservatively.

**Conclusion: **Despite advances in treatment, chronic hepatitis B remains a major cause of HCC in the region. The high prevalence in patients with cirrhosis and late-stage diagnosis underline the importance of enhanced monitoring to enable earlier detection and improve patient outcomes.

## TP-028 Nonampullary Duodenal Polyps: Risk Factors, Incidence, and Histopathological Features

**Murat Saruç ^1^ , Gürhan Şişman ^1^ , Hakan Ümit Ünal ^2^ , Şafak Kızıltaş ^2^ , Oya Yönal ^2^ , Can Gönen ^1^ , Hakan Yıldız ^2^ , Fatih Oğuz Önder ^1^ , Suna Yapalı ^1^ , Cem Aygün ^2^ , Arzu Tiftikçi ^1^ , Nesliar Eser Kutsal ^2^ , Hülya Hamzaoğlu ^2^ , Aysun Bozbaş ^2^ , Erkin Öztaş ^2^ , Bahattin Çiçek ^2^ , Süha Göksel ^3^ , Ayşe Sibel Erdamar Çetin ^3^ , Melisa Ulufi ^2^ , Cavit Kerem Kayhan ^3^ , Can Boynukara ^4^ , Orhan Cem Deniz ^1^ , Ariorad Moniri ^1^ , Parsa Mohri ^1^ , Elif Doğa Karaburun ^1^ , Ayşe Nurdan Tözün ^1^**

^1^Department of Gastroenterology, Acıbadem Mehmet Ali Aydınlar University School of Medicine, İstanbul, Türkiye

^2^Acıbadem Health Group, İstanbul, Türkiye

^3^Department of Pathology, Acıbadem Mehmet Ali Aydınlar University, İstanbul, Türkiye

^4^Department of Internal Medicine, Acıbadem Mehmet Ali Aydınlar University School of Medicine, İstanbul, Türkiye

**Background/Aims:** Nonampullary duodenal polyps are incidentally found in approximately 5% of upper GI endoscopies and carry malignant potential, necessitating careful endoscopic management. There are limited studies on the prevalence and risk factors of duodenal polyps. This study aimed to establish the prevalence, clinicopathological features, and associated risk factors for duodenal polyps diagnosed between 2020 and 2024.

**Materials and Methods:** This retrospective, multicenter cohort study analyzed electronic data from gastroscopies performed between October 2019 and October 2024. Assessment of patient demographics, polyp features (size, number, histology, location), comorbidities, lifestyle habits, BMI, *Helicobacter pylori* status, and medication use was conducted.

**Results: **Among 3011 upper GI endoscopies, 761 duodenal polyps were detected. Of these, 544 (71.5%) were histopathologically confirmed as duodenal polyps, yielding a prevalence of 17% in the total series. In 217 cases (28.5%), the polyp diagnosis was not histopathologically verified. Among the 544 confirmed cases, 89% were nonneoplastic and 11% were neoplastic. The majority of polyps (n = 389, 71.5%) were located in the first part of the duodenum. Patients were 55% male and 45% female, with a mean age of 57 years (range 27-86) and a mean BMI of 27 kg/m^2^. Risk factor analysis revealed a strong correlation with smoking (*P* < .001, OR = 6.2, 95% CI) and a significant correlation with PPI use (*P* = .0038, OR = 4.871, 95% CI). For endoscopic treatment, 70% were removed with biopsy forceps, 20% underwent polypectomy via underwater technique or standard hot/cold snare, and 10% received EMR/ESD. Complications occurred in 2% of patients, including postpolypectomy bleeding (n = 2) and perforation (n = 1), all managed with hemoclips.

**Conclusion: **Duodenal polyps are challenging due to their location, malignant potential, and high risk of postresection perforation, particularly for novices. Histological confirmation is mandatory for guiding treatment. A strategy based on risk stratification is crucial to prevent unnecessary interventions and ensure appropriate management of high-risk lesions.

## TP-030 The Clinical Value of Quality-of-Life Assessment in the Follow-up of Patients with Celiac Disease: A Single-Center Experience

**Yavuz Özden**

Department of Gastroenterology, Kayseri City Hospital, Kayseri, Türkiye

**Background/Aims: **Celiac disease is a chronic autoimmune enteropathy triggered by gluten ingestion in genetically predisposed individuals, necessitating lifelong adherence to a strict gluten-free diet. Such dietary restrictions impact not only physical health but also social, psychological, and economic well-being. Regular evaluation of quality of life (QoL) during follow-up may enhance treatment adherence and patient satisfaction. This study aimed to assess the QoL of patients with celiac disease, identify factors influencing it, and examine the role of patient support systems.

**Materials and Methods: **This prospective, single-center, observational study included 100 adults with biopsy-confirmed celiac disease followed at the tertiary center. QoL was assessed using the validated Turkish version of the Celiac Disease Quality of Life Survey (CD-QoL). Demographic data, disease duration, dietary adherence, membership in celiac associations, and education level were recorded. Descriptive analyses were performed, and relationships between QoL and clinical or sociodemographic factors were evaluated.

**Results:** The mean age was 32 years, and 65% were female. Thirty-two percent had been diagnosed within the previous year, and 8% were members of a patient association. Thirty-five percent reported that the disease significantly restricted their daily lives, 27% felt stigmatized, 35% experienced social difficulties, and 30% struggled to follow the diet while traveling. Although 78% reported full adherence to a gluten-free diet, 75% still experienced reduced QoL. The most frequent challenges were the high cost of gluten-free foods and limited access to safe dietary options in restaurants or during travel.

**Conclusion:** Celiac disease significantly reduces QoL even in asymptomatic individuals. This impact extends beyond physical symptoms to psychosocial and economic domains. Routine QoL assessment, along with clinical follow-up, should be incorporated into patient management. Educational programs, support from dietitians or nurses, and collaboration with patient associations may enhance both dietary adherence and overall QoL, contributing to more comprehensive long-term care.

## PS-001 Evaluation of The Relationship Between PNI and CONUT Scores with Steroid Treatment Response and Length of Hospital Stay in Patients with Ulcerative Colitis

**Eda Nur Duran ^1^ , Selçuk Candan ^2^ , Alper Uysal ^2^ , Murat Akarsu ^1^ , Şengül Aydın Yoldemir ^1^ , Ömür Tabak ^1^**

^1^Department of Internal Medicine, Kanuni Sultan Süleyman Education and Research Hospital, University of Health Sciences, İstanbul, Türkiye

^2^Department of Gastroenterology, Kanuni Sultan Süleyman Education and Research Hospital, University of Health Sciences, İstanbul, Türkiye

**Background/Aims:** Ulcerative colitis (UC) is a chronic inflammatory disease of the gastrointestinal tract that can significantly affect patients’ quality of life. Nutritional status plays an important role in disease prognosis and treatment response. In recent years, the prognostic nutritional index (PNI) and the nutritional status control score (CONUT) have been associated with disease activity, complication risk, and treatment decisions in patients with UC. It has also been reported that evaluating these scores alongside inflammatory markers such as C-reactive protein may more accurately reflect disease activity. This study aimed to investigate the association of PNI and CONUT scores with steroid treatment response and length of hospital stay in hospitalized patients with UC.

**Materials and Methods:** Data from 53 patients hospitalized with UC between 2020 and 2025 were retrospectively analyzed. Demographic and laboratory data at admission, steroid response, and length of hospital stay were recorded. Steroid response was categorized as complete, partial, or none based on clinical findings. Statistical analysis was performed using SPSS 18.0; *P*-values <.05 were considered significant.

**Results:** The mean age was 39.7 ± 14.8 years; 66% showed a complete response, 32.1% a partial response, and only 1 patient had no response to steroids. The mean PNI score was 42.63 ± 7.47, and the mean CONUT score was 4.09 ± 2.77. No significant difference was found between response groups in terms of PNI or CONUT scores (*P* = .331 and *P* = .462). There was no correlation between either score and length of hospital stay (*P* = .909 and *P* = .847). However, the CONUT score was significantly higher in patients treated with mesalazine (*P* = .022).

**Conclusion: **PNI and CONUT scores were not significantly associated with steroid response or length of hospital stay. However, the significant association between CONUT and mesalazine treatment suggests that nutritional status may play a role in clinical decision-making. Larger multicenter studies are needed to confirm these findings.

## PS-002 New Biomarker in The Diagnosis of MAFLD: S-Index

**Muhammet Ali Diken ^1^ , Mustafa Cengiz ^2^**

^1^Department of Internal Medicine, Konya Meram State Hospital, Konya, Türkiye

^2^Department of Gastroenterology and Hepatology, Gülhane Training and Research Hospital, University Of Health Sciences Faculty of Medicine, İstanbul, Türkiye

**Background/Aims:** This study aimed to evaluate the diagnostic performance of the S-index in identifying metabolic dysfunction-associated steatohepatitis (MASH) and predicting the stage of fibrosis, in comparison with other noninvasive tests, among patients with biopsy-proven diagnoses.

**Materials and Methods:** In this single-center, cross-sectional, retrospective study conducted at the Gastroenterology Department between November 2016 and December 2024, a total of 177 participants were included: 11 with biopsy-proven hepatic steatosis, 83 with biopsy-proven MASH, and 83 healthy controls. Liver biopsy specimens were assessed using the Non-Alcoholic Fatty Liver Disease Activity Score (NAS) system. The diagnostic performance of the S-index was compared with the aspartate aminotransferase to platelet ratio index (APRI), fibrosis-4 index (FIB-4), aspartate aminotransferase to alanine aminotransferase ratio (AAR), AAR to platelet ratio index (AAPRI), gamma-glutamyl transferase to platelet ratio (GPR), King’s score, Fibro-Q, and platelet-to-lymphocyte ratio (PLR).

**Results:** Separate analyses were conducted with and without the inclusion of the steatosis group. Among the patients with MASH, 74.7% were classified as having mild fibrosis, whereas 25.3% had advanced fibrosis. Patients with a fibrosis score ≥2 were categorized as having advanced fibrosis, and those with a score of 1 or no fibrosis were classified as having mild fibrosis. The S-index demonstrated high diagnostic accuracy in distinguishing patients with MASH from healthy controls, showing the highest sensitivity (89.2%) among all indices tested (AUC: 0.923, *P* < .001). Furthermore, in the three-group analysis, the S-index effectively discriminated patients with MASH from those with simple steatosis (*P* < .001). However, the S-index did not show a significant ability to differentiate between fibrosis stages (*P* = .077).

**Conclusion:** The S-index is a biomarker with high diagnostic accuracy for predicting MASH. It exhibited superior discriminative capacity and the highest sensitivity in differentiating patients with MASH from healthy controls. Additionally, in three-group comparisons, the S-index was able to significantly distinguish patients with MASH from those with simple steatosis.

## PS-003 A Rare Coexistence: Celiac Disease and Ulcerative Colitis

**Ahmet Uyanıkoğlu ^1^ , Serpil Uyanıkoğlu ^2^**

^1^Division of Gastroenterology, Harran University Faculty of Medicine, Şanlıurfa, Türkiye

^2^Department of Family Practice, Harran University Medical Faculty, Şanlıurfa, Türkiye

According to the literature, the coexistence of CD and UC is infrequent. This study presents a young female patient with CD who developed UC. A 26-year-old female patient presented at Harran University Gastroenterology Polyclinic with complaints of bloody diarrhea with mucus occurring 15-20 times a day, along with nausea and vomiting. It was noted that the patient had previously presented to the polyclinic with complaints of short stature during childhood and was diagnosed with CD 12 years ago but did not adhere to the celiac diet. On physical examination, the patient appeared in moderate condition, standing 153 cm tall and weighing 42 kg, with a BMI of 17.1. Laboratory results showed leukocyte count: 14.74 10e3/dL, hemoglobin: 6.7 g/dL, and CRP: 16.1 mg/dL. The patient received 2 units of erythrocyte suspension. In gastroscopy, the second part of the bulbus and duodenum appeared hyperemic, edematous, and velvety. Biopsy results indicated Marsh 3C. Colonoscopy revealed hyperemic, edematous, granular mucosa with localized ulceration in all segments of the colon. The patient was diagnosed with UC (left colon involvement, moderate-to-severe activity). The biopsy showed cryptitis and crypt distortion, which were consistent with UC. The patient was started on oral and rectal mesalazine, parenteral steroids, and a gluten-free diet. The patient’s symptoms decreased, leading to a switch to oral steroids, and she was discharged with outpatient follow-up recommendations. The only current treatment for celiac disease is adherence to a gluten-free diet. It is rare for CD to develop UC after the initial diagnosis, which is known to be associated with many conditions. If a patient with CD presents with bloody diarrhea, it should be considered that it may be UC, as in the patient in the present study.


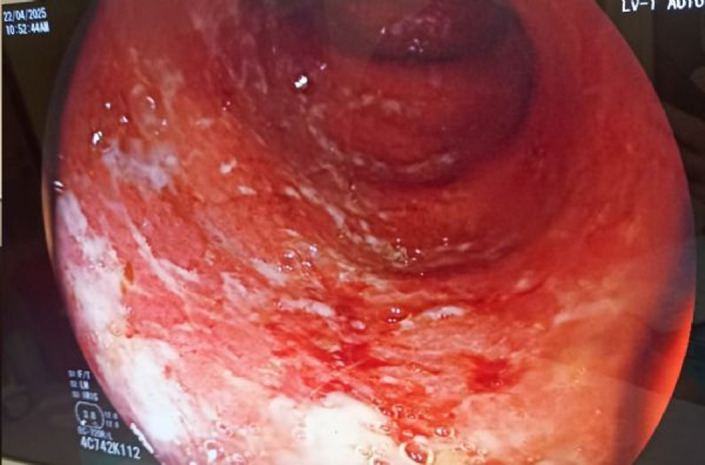


Figure 1. Ulcerative colitis in a celiac patient.

## PS-004 Evaluation of Quality of Life in Reproductive-Stage Women with Irritable Bowel Syndrome

**Ceylan Keskin ^1^ , Esat Sabuncu ^2^ , Arjin Baytar ^3^ , Malik Enes Yokuş ^3^ , Zeynep Münire Uyanıkoğlu ^4^ , Ahmet Uyanıkoğlu ^5^**

^1^Department of Internal Medicine, Harran University Faculty of Medicine, Şanlıurfa, Türkiye

^2^Department of Psychiatry, Harran University Faculty of Medicine, Şanlıurfa, Türkiye

^3^Harran University Faculty of Medicine, Şanlıurfa, Türkiye

^4^İstanbul Aydın University Faculty of Medicine, İstanbul, Türkiye

^5^Division of Gastroenterology, Harran University Faculty of Medicine, Şanlıurfa, Türkiye

**Background/Aims: **Irritable bowel syndrome (IBS) is a common condition in society. This study aimed to compare the quality of life of female patients with IBS in the reproductive period with healthy controls without IBS.

**Materials and Methods: **The study included 50 consecutive female patients aged 18-55 years diagnosed with IBS according to the Rome IV criteria and 50 healthy controls with similar demographic characteristics, without malignant or chronic diseases and without IBS. Demographic data, health anxiety scale, BECK anxiety scale, and quality of life scale questionnaires were completed for both groups, and the groups were compared.

**Results: **The mean age of the 50 patients diagnosed with IBS was 34.84 ± 11.44 (range, 18-54), and the mean age of the control group was 28.18 ± 9.39 (range, 18-53). The demographic characteristics of both groups were similar. Nine (18%) of the patients with IBS had psychiatric illnesses, compared to 4 (8%) in the control group (*P* < .05). Eleven (22%) of patients with IBS were smokers, whereas 6 (12%) were in the control group (*P* < .05). Although the mean health anxiety was similar in patients with IBS compared to the control group, the BECK anxiety scale and IBS quality of life scale were found to be significantly higher in the control group (35.44 ± 8.8 vs. 33.36 ± 8.8, *P* > .05; 25.06 ± 9.4 vs. 13.26 ± 10.8, *P* < .05; 98.3 ± 22.3 vs. 37.8 ± 6.6, *P* < .05).

**Conclusion: **In patients with IBS in the reproductive period, the health anxiety scale was similar to that of the healthy control group with comparable age and demographics, whereas the BECK score was approximately 2 times higher, and the IBS quality of life scale score was approximately 3 times higher.

## PS-005 A Case of Miliary Tuberculosis Presenting with Inappropriate ADH Syndrome, Mimicking Gastric Malignancy

**Ahmet Uyanıkoğlu ^1^ , Osman Yüksekyayla ^1^ , Mustafa Fırat Güler ^2^**

^1^Division of Gastroenterology, Harran University Faculty of Medicine, Şanlıurfa, Türkiye

^2^Department of Internal Medicine, Harran University Faculty of Medicine, Şanlıurfa, Türkiye

Syndrome of inappropriate ADH secretion is a condition characterized by excessive or uncontrolled ADH secretion from the hypothalamus and/or pituitary gland, leading to decreased serum osmolality and dilute hyponatremia. Miliary tuberculosis is a disseminated form that occurs in the body due to the hematogenic spread of mycobacterium tuberculosis, particularly affecting the lungs and other organs. In these cases, it has been reported that central nervous system involvement, although rare, increases ADH secretion by indirectly affecting the hypothalamic-pituitary axis. A case of miliary tuberculosis manifesting as the syndrome of inappropriate ADH in a patient suspected of having gastric malignancy due to weight loss and dyspeptic complaints is presented. A 75-year-old male patient presented with an involuntary weight loss of approximately 20 kg over the past 3 months, along with longstanding fatigue and weakness. He had no other complaints. Endoscopy for malignancy screening revealed antral gastritis, and colonoscopy showed internal hemorrhoids. The patient had a history of chronic hyponatremia, and laboratory results were consistent with the syndrome of inappropriate antidiuretic hormone release (SIADH). Since the endoscopy and colonoscopy results were normal, other potential causes of SIADH were investigated. Chest CT was compatible with miliary tuberculosis. The sputum ARB test was positive for 3 consecutive days, and the patient was diagnosed with miliary tuberculosis. The patient was transferred to the chest disease service, and despite receiving a four-drug antituberculosis regimen and supportive care for 1 week, his general condition deteriorated, and he died due to respiratory and cardiac arrest. This case suggests that the underlying cause of inappropriate ADH may be miliary tuberculosis and may present with dyspeptic complaints.


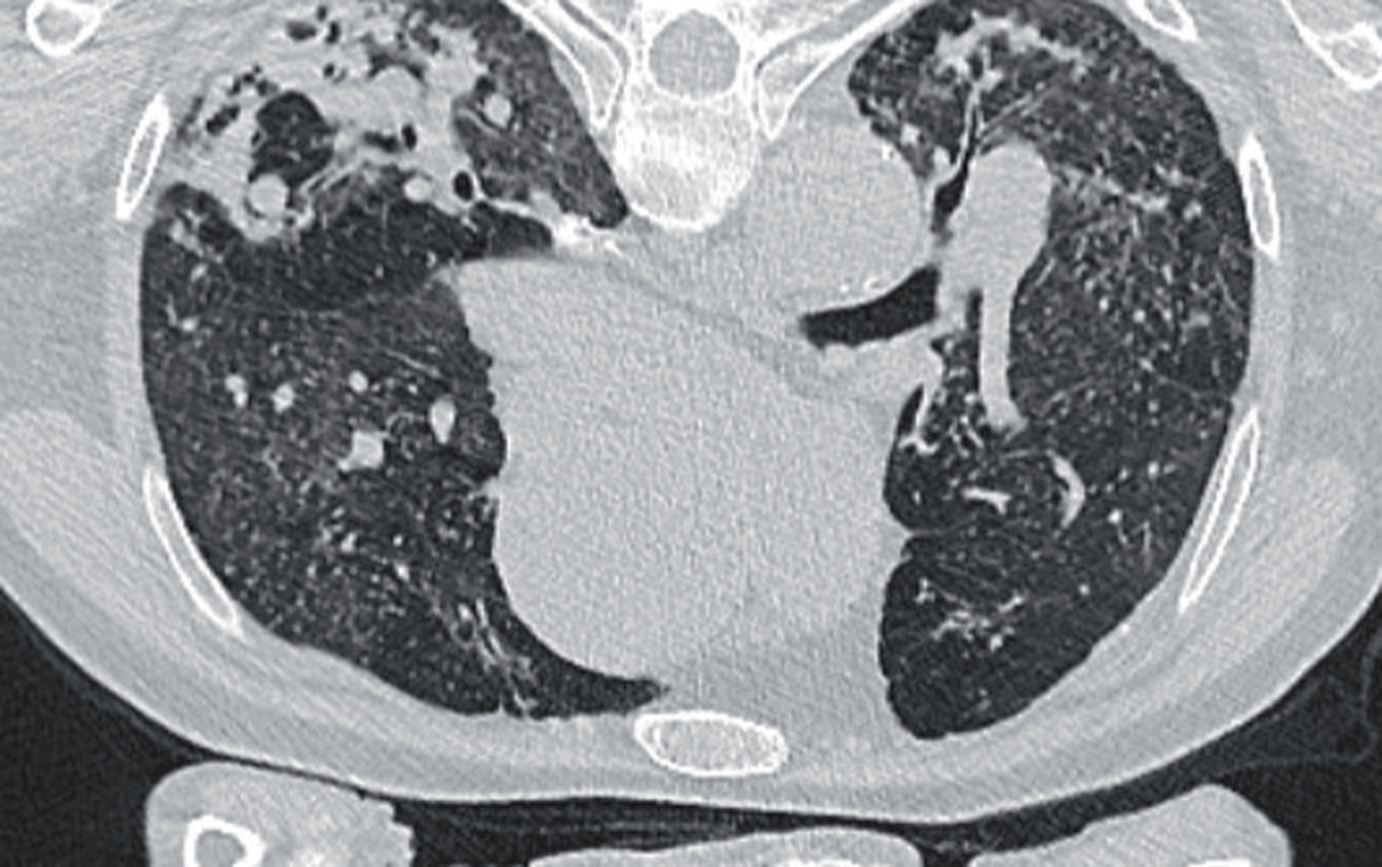


Figure 1. Infiltrative lesions with miliary distribution in all lung fields.

## PS-008 Intragastric Knotting of a Percutaneous Endoscopic Gastrostomy with a Jejunal Extension Tube in a Patient with Dementia: A Rare Mechanical Complication

Etibar Mammadov, Mehmet Kapan, Nejla Küçük, Utku Hasan Kocal, Murat Başaran, Mehmet Suat Yalçın, Burak Özşeker

Muğla Training and Research Hospital, Muğla, Türkiye

Percutaneous endoscopic gastrostomy with a jejunal extension (PEG-J) is a commonly preferred method for long-term enteral nutrition in patients unable to take oral intake. Although generally considered safe, mechanical complications may occur rarely. One of the least reported of these is the knotting of the jejunal extension within the stomach. A case of intragastric knotting of a PEG-J tube in a 70-year-old female patient diagnosed with dementia is presented. During endoscopy, the knotted tube was visualized and carefully removed. A new tube was successfully placed, and the procedure was completed without complications. Intragastric knotting of PEG-J tubes is a rare but potentially serious complication. In patients presenting with feeding intolerance, prompt endoscopic evaluation and timely intervention are essential.


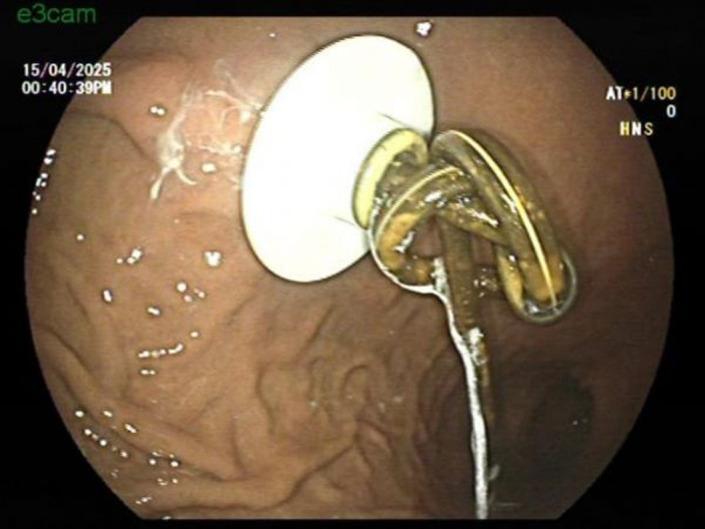


Figure 1. Endoscopic view of the PEG-J tube knotted within the stomach.


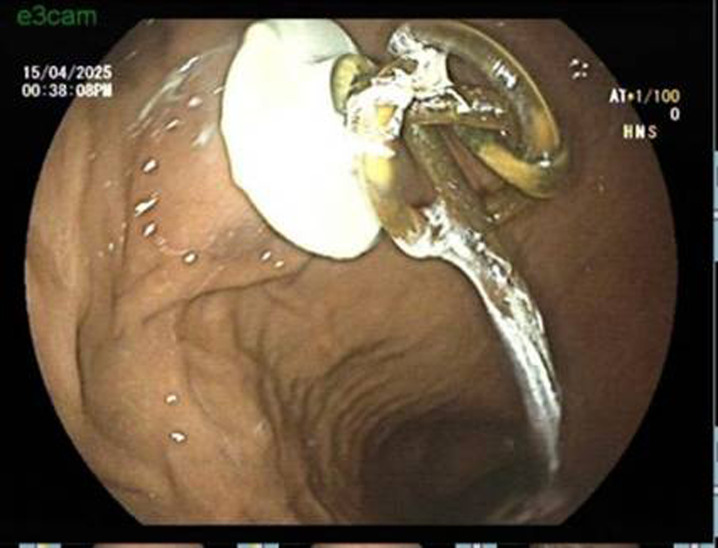


Figure 2. Close-up view of the knotted PEG-J tube covered in mucus.

## PS-009 Celiac Disease Presenting with Growth and Developmental Delay

**Ceylan Keskin ^1^ , Ahmet Uyanıkoğlu ^2^ , Zeynep Münire Uyanıkoğlu ^3^**

^1^Department of Internal Medicine, Harran University Faculty of Medicine, Şanlıurfa, Türkiye

^2^Division of Gastroenterology, Harran University Faculty of Medicine, Şanlıurfa, Türkiye

^3^İstanbul Aydın University Faculty of Medicine, İstanbul, Türkiye

Celiac disease is an autoimmune condition caused by sensitivity to the protein gluten, found in grains such as wheat, barley, and rye, in genetically susceptible individuals. This sensitivity results in intestinal mucosal damage and malabsorption. A gluten-free diet is the cornerstone of treatment. In adults, the initial symptom is often diarrhea, but it can also present with extraintestinal findings such as anemia, osteoporosis, increased nonspecific transaminases, and developmental delay. The most common findings in children are diarrhea, along with growth and developmental delay. Vitamin D, zinc, iron, and vitamin A deficiencies are the most common micronutrient deficiencies in patients with celiac disease. A pediatric patient presenting with growth and developmental delay is described. A 14-year-old child presented to the outpatient clinic with complaints of abdominal pain, diarrhea, and growth and developmental delay. The patient was noted to be short in stature and developmentally delayed compared to peers. The patient’s physical examination was normal, with both lungs participating equally in respiration. There was no significant S1-S2 murmur. The abdomen was soft, with no rebound tenderness or defense. Temperature was 36.5°C. Routine blood tests were requested, and tissue transglutaminase and anti-endomysium antibodies were tested for celiac disease. Laboratory findings included vitamin D at 15.85 ng/mL(normal range, 30-100), tissue transglutaminase IgA at 24.2 (positive), tissue transglutaminase IgG at 111.8 (positive), anti-endomysium IgG at +3 (positive), and anti-endomysium IgA at +3 (positive). Gastroscopy findings were consistent with celiac disease, and Marsh type 3c was detected in the biopsy. The patient’s symptoms, serological tests, gastroscopy, and biopsy results were consistent with celiac disease, leading to a definitive diagnosis. Nutritional deficiencies were corrected, and a gluten-free diet was initiated. The patient was placed under outpatient follow-up. Celiac disease can present with growth and developmental delays, as seen in this patient. Early diagnosis and appropriate treatment can prevent complications such as anemia, osteoporosis, and developmental delay, particularly those caused by malabsorption.

## PS-010 Effects of Coronavirus Disease-2019 Pandemic on PEG

**Duran Deha Çetin, Şehmus Ölmez, Bünyamin Sarıtaş, Mustafa Harı, Banu Kara**

Department of Gastroenterology, Adana City Training and Research Hospital, University of Health Sciences, Adana, Türkiye

**Background/Aims:**Coronavirus disease 2019 (COVID-19) is a viral disease transmitted via the respiratory route that has caused a global pandemic. Percutaneous endoscopic gastrostomy (PEG), a frequently preferred method for patients requiring long-term enteral nutrition for various reasons, is often performed on fragile patients with multiple comorbidities who are at high risk of complications. This study aimed to evaluate the impact of the COVID-19 pandemic on PEG procedures.

**Materials and Methods: **Data from patients who underwent PEG in the endoscopy unit of Adana City Training and Research Hospital during the 2 years before and after the COVID-19 pandemic were retrospectively analyzed. Patient records were reviewed to collect demographic characteristics, PEG indications, changes in requesting clinical departments, laboratory parameters, complication rates, and mortality data. The pre-pandemic period (group 1) and post-pandemic period (group 2) were compared.

**Results: **In the pre-pandemic period (group 1), 270 patients (141 males, 129 females) were included, whereas in the post-pandemic period (group 2), 170 patients (90 males, 80 females) were included. The mean age was 65.2 ± 18.1 years in group 1 and 63.8 ± 17.8 years in group 2. In both groups, the most common indication for PEG was cerebrovascular accident (CVA). The mean survival time was 146 days in group 1 and 69 days in group 2 (*P* = .003). Although no significant increase in complication rates was observed in group 2, the mortality rate was found to be 1.46 times higher compared to group 1.

**Conclusion: **Although no significant change in complication rates was observed in patients undergoing PEG during the COVID-19 pandemic, a significant decrease in survival time was detected. This may be attributed to changes in the patient profile in intensive care units, stricter indications for PEG placement, and disruptions in follow-up processes during the pandemic. These findings may provide guidance for maintaining continuity of enteral nutrition in potential future infectious disease pandemics.

## PS-011 Clinical Faces of Gastrointestinal System Lymphomas: A Case Series

**Mehmet Kapan ^1^ , Nejla Küçük ^1^ , Etibar Mammadov ^1^ , Hasan Utku Kocal ^1^ , Murat Başaran ^2^ , Mehmet Suat Yalçın ^2^ , Burak Özşeker ^1^**

^1^Department of Gastroenterology, Muğla Sıtkı Koçman University Faculty of Medicine, Muğla, Türkiye

^2^Muğla Training and Research Hospital, Muğla, Türkiye

Gastrointestinal lymphomas account for 1%-4% of gastrointestinal malignancies and 10%-15% of non-Hodgkin lymphomas. They are the most common extranodal site, representing 35%-40% of all extranodal lymphomas. GI lymphomas are most frequently found in the stomach, followed by the small intestine, colorectal region, and esophagus. Diagnostic imaging methods such as magnetic resonance imaging and computed tomography, along with esophagogastroduodenoscopy and endoscopic ultrasound, can be used. Diagnosis is confirmed through histopathological examination and immunohistochemical staining. The clinical presentation of lymphomas can range from gastric ulcers to mass lesions. This case series was presented with the aim to emphasize that, although rare, lymphoproliferative diseases should not be overlooked during endoscopic imaging.


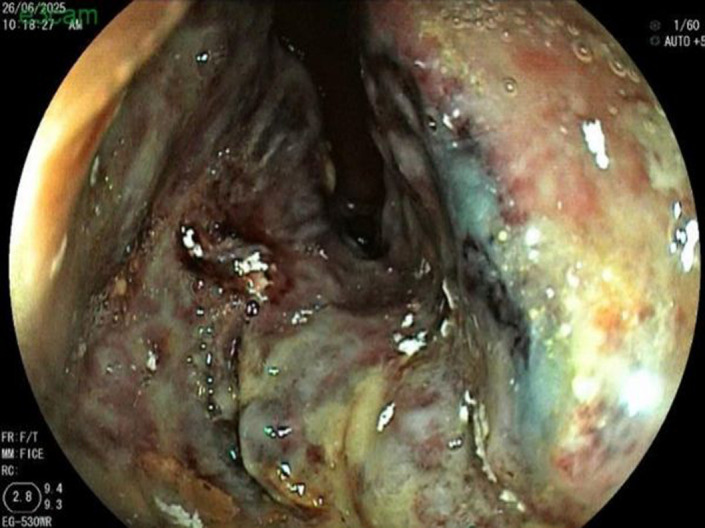


Figure 1. Fragile necrotic mass observed in a 7-cm segment starting from the second part of the duodenum and including the papilla.


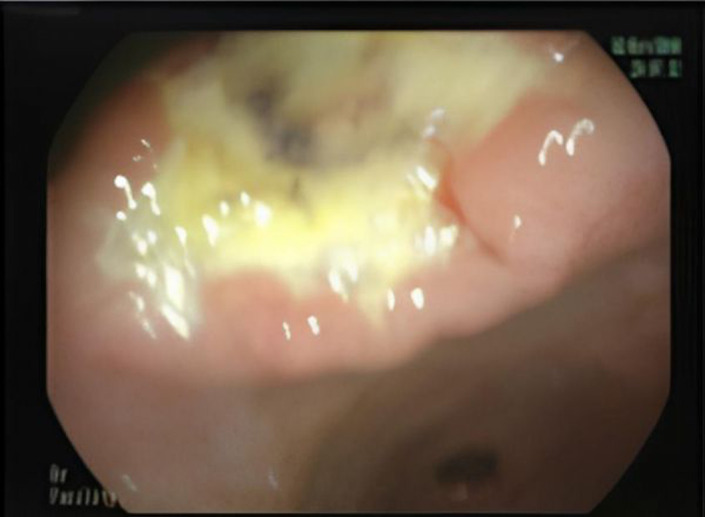


Figure 2. An ulcer in the incisura angularis with a swollen mucosa and a depressed center, approximately 2 cm in diameter, covered with white exudate.


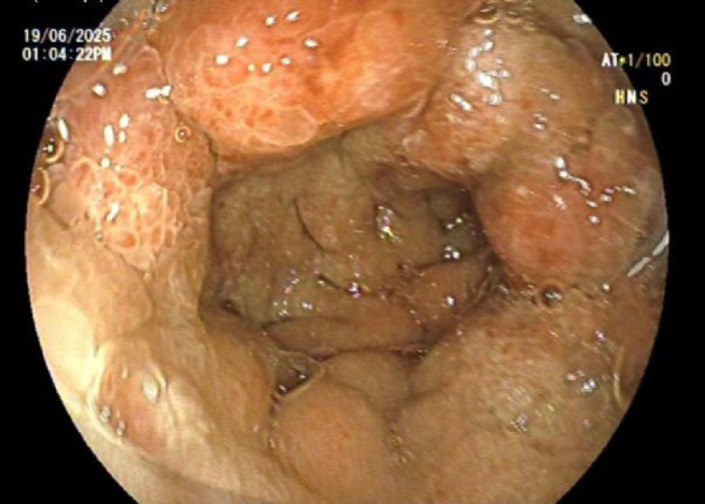


Figure 3 Irregularity, edema, and hyperemic areas in the prepyloric antrum mucosa.

## PS-014 Management Challenges of Acute Severe Ulcerative Colitis Complicated by Epstein–Barr Virus and Tuberculosis-Triggered Hemophagocytic Lymphohistiocytosis

**Tugba Tolu Bulte, Yasemin Armutcuoğlu, H. Tarık Kani, Özlen Atuğ, Yeşim Özen Alahdab**

Department of Gastroenterology, Marmara University School of Medicine, İstanbul, Türkiye

Hemophagocytic lymphohistiocytosis (HLH) is a rare hyperinflammatory syndrome with high mortality, occasionally observed in immunosuppressed patients such as those with inflammatory bowel disease (IBD). A middle-aged male patient, diagnosed with extensive ulcerative colitis in 2016 and known to be steroid-refractory, presented with an acute severe attack (pMayo: 8), bicytopenia, and persistent fever while under treatment with azathioprine and newly initiated infliximab. No infectious etiology was identified during the early course. His clinical status deteriorated with persistent neutropenia, increased inflammatory markers, and hypofibrinogenemia. Lymph node aspiration revealed *Mycobacterium tuberculosis* PCR positivity, and biopsy demonstrated histiocytic proliferation with hemophagocytosis, as well as Epstein–Barr virus (EBV)-encoded RNA (EBER) positivity. The findings were consistent with the HLH-2004 diagnostic criteria. Following a hematology consultation, the initiation of antituberculosis therapy was recommended for the suspected underlying etiology, along with close clinical monitoring.

Antituberculosis therapy was initiated, and the patient was transferred to the infectious diseases department. In the third month of hospitalization, he developed massive gastrointestinal bleeding secondary to a Forrest 1a ulcer and, despite interventional radiology and surgical procedures, died from hemorrhagic shock due to disseminated intravascular coagulation triggered by HLH. This case highlights the diagnostic challenges and high mortality risk of HLH in patients with IBD receiving immunosuppressive therapy. HLH should be considered early in the management of acute severe ulcerative colitis, and prompt multidisciplinary intervention is essential.


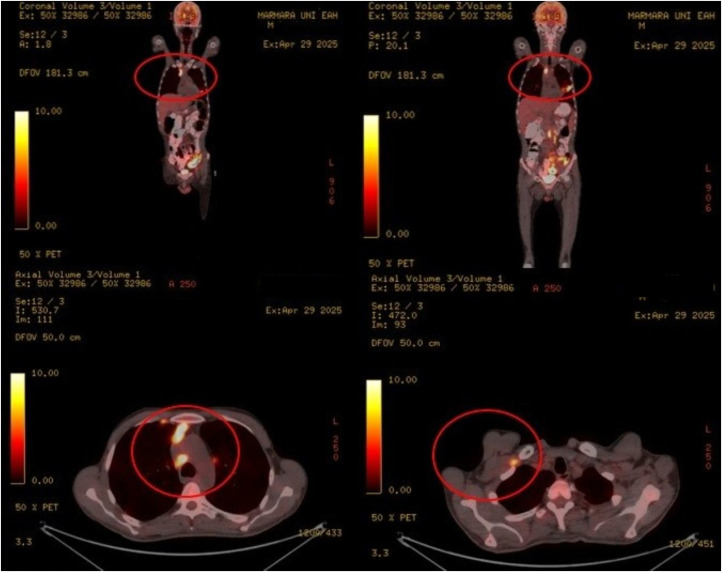


Figure 1. FDG-PET/CT demonstrating multiple hypermetabolic lymph nodes in the mediastinal, abdominal, and pelvic regions. Findings are consistent with widespread hyperinflammatory activity associated with hemophagocytic lymphohistiocytosis.

## PS-015 Gastroduodenitis Associated with Ulcerative Colitis: A Case Report

**Ali Bilgen**

Division of Gastroenterology, Private Gaziantep Anka Hospital, Gaziantep, Türkiye

A 30-year-old female patient presented to the outpatient clinic with complaints of diarrhea with bloody mucus occurring 15 times a day, nausea, and colic-type epigastric pain for the last 3 weeks. On physical examination, her vital signs were normal, but there was abdominal tenderness with no defense or rebound. Laboratory tests revealed CRP: 58 mg/L, creatinine: 0.58, AST: 29, ALT: 16, Sodium: 138, Potassium: 4, Calcium: 8.9, WBC: 10 800, Hgb: 12.1, and PLT: 449 000. Abdominal tomography revealed thickening of the gastric mucosa and wall thickening of all colonic loops. Stool microscopy revealed no parasites or cysts, but showed dense leukocytes and erythrocytes. Upper GI endoscopy revealed severe erosive gastritis and erosive bulbit; colonoscopy revealed a 10 cm ileum and a normal ileocecal valve. The mucosa of the cecal base, ascending colon, transverse colon, descending colon, sigmoid colon, and rectum was hyperemic, erythematous, edematous, and bled upon touch, with widespread continuous mucosal phalanges. Biopsies were taken from the antrum, corpus, bulb, and colorectal areas. Gastric biopsies were *H. pylori* negative, and findings of active gastritis with crypt abscesses within the gland lumen were reported, whereas duodenal and colon biopsies revealed findings of active duodenitis and colitis with crypt abscesses, respectively. The patient was suspected of having gastroduodenitis associated with ulcerative colitis. Treatment was initiated with 40 mg/day intravenous methylprednisolone, 3 g/day oral 5-ASA, and 40 mg pantoprazole. The patient’s symptoms significantly decreased within 3 days. One week later, stool count and consistency returned to normal, and epigastric pain and dyspeptic complaints resolved. Maintenance therapy was initiated with a plan to reduce steroids by 4 mg per week. Follow-up endoscopy and colonoscopy were scheduled, and medical treatment was arranged. The patient was discharged.

## PS-017 Metastatic Malignant Melanoma of the Stomach

**Vedat Göral ^1^ , Özcan Yıldız ^2^ , Ferhat Özden ^3^**

^1^Department of Gastroenterology, Medipol Mega Hospital, İstanbul Medipol University, İstanbul, Türkiye

^2^Department of Medical Oncology, Medipol Mega Hospital, İstanbul Medipol University, İstanbul, Türkiye

^3^Department of Pathology, Medipol Mega Hospital, İstanbul Medipol University, İstanbul, Türkiye

Melanoma is the most invasive skin cancer with the highest risk of death. Although it is a serious skin cancer, it is highly treatable when diagnosed early. This case report presents a patient with metastatic malignant melanoma that developed 5 years after an initial diagnosis of malignant melanoma in an outpatient setting. A 48-year-old male patient presented to the clinic with widespread body and bone pain and had experienced abdominal pain in the epigastric region. Radiological examinations revealed metastatic lesions in the liver, bones, and lungs. In 2020, a biopsy performed on a lesion on the foot revealed acral lentiginous malignant melanoma. CT and PET/CT scans were performed, and no metastasis was detected. The mass was removed from the sole of the foot by a plastic surgeon at a private hospital, and a hip flap was performed. The examination revealed no metastasis and clean surgical margins, but no oncological follow-up was conducted. When he presented to us in April 2025, he underwent endoscopy and colonoscopy. The endoscopy revealed a hard, hollow tumor larger than 2 cm in diameter in the fundus of the stomach. Eight biopsies were taken from the lesion. The pathological examination noted the presence of metastatic malignant melanoma (S100: Positive, MelanA: Positive, panCK: Positive in surface and gland epithelium, negative in tumor). Brunner’s gland hyperplasia was detected in the duodenum, and a tubular adenoma was detected in a polyp in the colon. The patient was referred for follow-up and treatment in medical oncology. The majority of skin cancer-related deaths (75%) are due to melanoma. In this case, no follow-up was performed after the initial treatment, and the patient consulted a physician only when metastases appeared. Patients with malignant melanoma must be monitored regularly.

## PS-018 A Rare Cause of Exudative Ascites and Peritoneal Involvement

**Kamil Enli, Halil Yılmaz, Yekta Duygu Çimen Beşirli, Mustafa Çelik**

Department of Gastroenterology, Pamukkale University Faculty of Medicine, Denizli, Türkiye

Ascites is the abnormal accumulation of fluid in the peritoneal cavity, observed in many clinical conditions. Distinguishing exudative from transudative ascites is essential for identifying the underlying cause. Extramedullary hematologic malignancies account for only a small fraction of hematologic neoplasms, and gastrointestinal or peritoneal involvement is extremely rare. Reported cases primarily affect the stomach or duodenum, whereas peritoneal disease is far less common. Because symptoms are nonspecific, diagnosis is often delayed. A rare case of unexplained peritonitis and colonic involvement with rapid deterioration and a fatal outcome is presented. A 53-year-old man with diabetes, hypertension, and chronic kidney disease on thrice-weekly hemodialysis presented with abdominal pain, distension, and diarrhea. Examination revealed ascites, and he was admitted for evaluation. Abdominal CT showed diffuse ascites, peritoneal thickening, and suspicious lymph nodes. Ascitic fluid was exudative with lymphocyte predominance and low glucose. Tuberculous peritonitis was suspected, but cultures were negative for acid-fast bacilli. Colonoscopy, limited to the splenic flexure due to poor preparation, showed hyperemic, edematous, exudate-covered mucosa in the rectum and sigmoid colon. A laparoscopic peritoneal biopsy was performed, followed by ward monitoring. During follow-up, spontaneous colonic perforation occurred, requiring emergency subtotal colectomy and omentectomy. The patient died postoperatively. Histopathology of the biopsy and surgical specimens confirmed plasma cell neoplasm. This case highlights that gastrointestinal and peritoneal involvement may represent a rare manifestation of hematologic malignancies. Exudative ascites and colitis-like endoscopic findings can mimic tuberculosis or inflammatory bowel disease. Histopathology remains crucial for definitive diagnosis. In patients with unexplained peritonitis and colonic lesions, rare hematologic malignancies should be considered in the differential diagnosis, and early multidisciplinary management is vital.


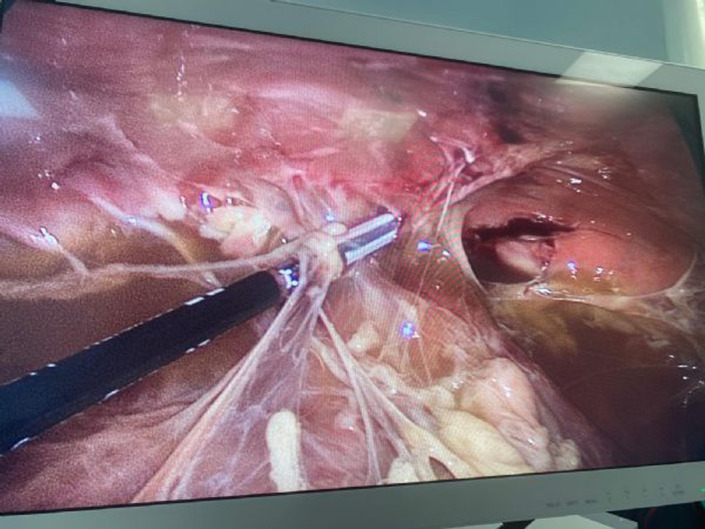


Figure 1. Plasma cell neoplasm peritoneal involvement. This is the image of the patient’s laparoscopic peritoneal biopsy.

## PS-019 Unexpected Persistence of Hemoclips in The Management of Gastrointestinal Bleeding: 17 Months of Experience

**Nejla Küçük, Etibar Mammadov, Mehmet Kapan, Murat Başaran, Mehmet Suat Yalçın, Burak Özşeker**

Division of Gastroenterology, Muğla Training and Research Hospital, Muğla, Türkiye

The primary objectives in the management of gastrointestinal bleeding are to stop active hemorrhage and prevent rebleeding. Achieving these goals necessitates a multimodal therapeutic approach, including endoscopic, pharmacological, angiographic, and surgical interventions. Hemoclips are commonly utilized in endoscopic hemostasis and are generally expected to detach spontaneously within days to weeks after application. However, in rare cases—such as the 1 presented here—hemoclips may persist in situ for several months or even years without detachment.


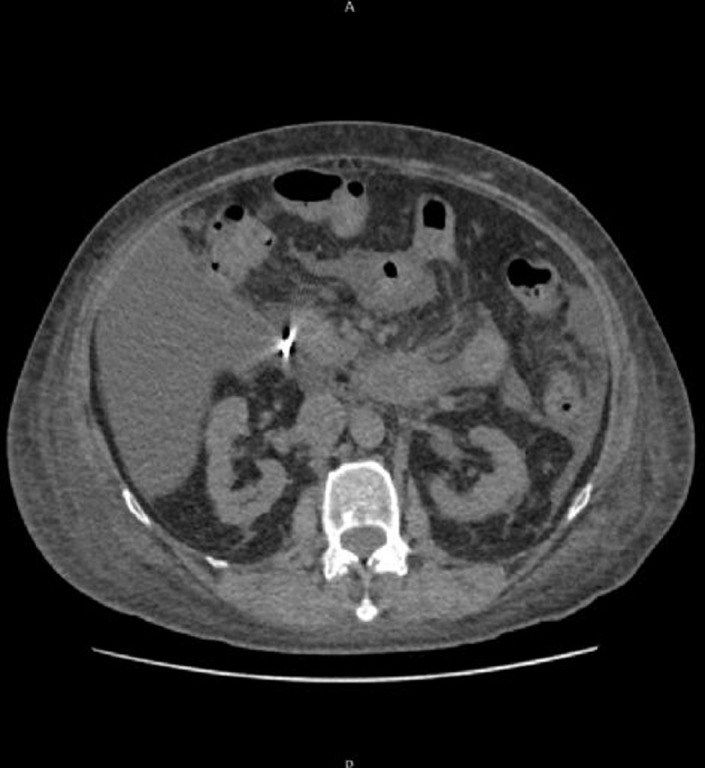


Figure 1. CT image of hemoclips after 17 months.


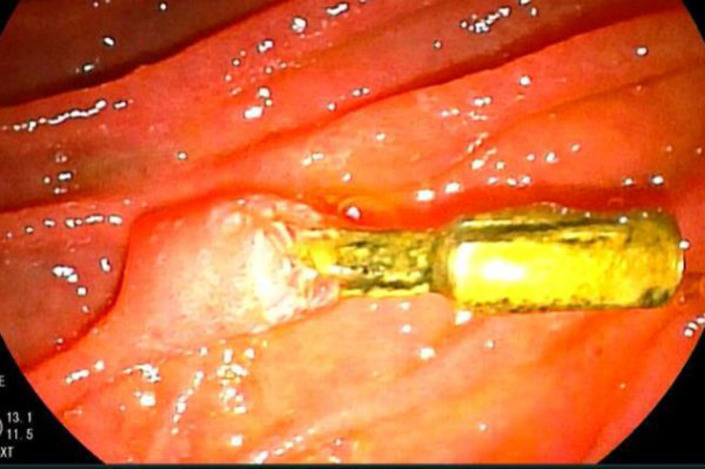


Figure 2. Endoscopy image of hemoclips after 17 months.

## PS-020 Paraduodenal (Groove) Pancreatitis Complicated by Intramural Duodenal Hematoma and Gastrointestinal Hemorrhage: A Case Report

**Tugba Tolu Bulte ^1^ , Yasemin Armutcuoğlu ^1^ , Muhsinem Yılmaz Düz ^2^ , Hale Nur Tıraş ^2^ , Coşkun Özer Demirtaş ^1^**

^1^Division of Gastroenterology, Marmara University Faculty of Medicine, İstanbul, Türkiye

^2^Department of Internal Medicine, Marmara University Faculty of Medicine, İstanbul, Türkiye

Paraduodenal pancreatitis (PP), also known as groove pancreatitis, is a rare form of segmental chronic pancreatitis localized between the pancreatic head and the duodenum. Obstructive symptoms are common, but hemorrhagic complications are exceedingly rare. A 47-year-old man with recurrent pancreatitis was found to have developed a large duodenal hematoma contiguous with the pancreatic head, initially mimicking active gastrointestinal bleeding. Computed tomography angiography revealed no evidence of arterial communication or active contrast extravasation; therefore, the patient was managed conservatively. Clinical improvement was associated with spontaneous decompression of the hematoma into the duodenal lumen. This case highlights the diagnostic challenge of PP and the importance of recognizing its rare hemorrhagic manifestations.


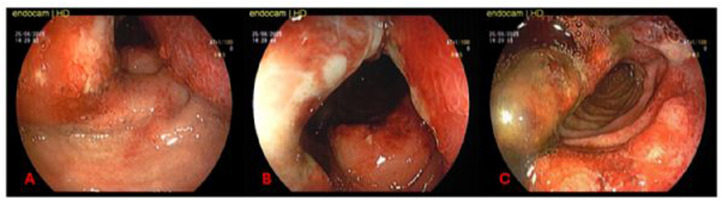


Figure 1. Endoscopic findings: (A) Edematous and hyperemic mucosa at the pyloric inlet, (B) Marked inflammatory changes causing narrowing at the pyloric inlet, (C) The second portion of the duodenum showing luminal narrowing due to an intramural hematoma with adjacent mucosal edema.


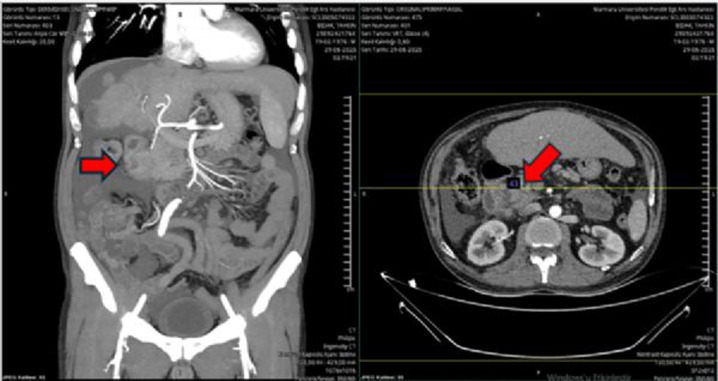


Figure 2. Coronal (left) and axial (right) contrast-enhanced CT images demonstrate a large intramural hematoma within the duodenal wall, in close proximity to the pancreatic head (arrows). The hematoma is associated with significant luminal narrowing of the duodenum.

## PS-023 A Rare Cause of Upper Gastrointestinal Bleeding: Gastric Glomus Tumor

**Mehmet Şerif Aktaş ^1^ , Serdar Akça ^1^ , Serkan Öcal ^1^ , Ayhan Hilmi Çekin ^1^ , Osman Çağın Buldukoğlu ^1^ , Tebessüm Çakır ^2^ , Ömer Kürklü ^2^ , Özlem Etli ^3^**

^1^Department of Gastroenterology, Antalya Training and Research Hospital, Antalya, Türkiye

^2^Department of Gastroenterology, Antalya Training and Research Hospital, Antalya, Türkiye

^3^Department of Pathology, Antalya Training and Research Hospital, Antalya, Türkiye

Gastric glomus tumors are rare mesenchymal neoplasms arising from the modified smooth muscle cells of the glomus body. Their occurrence in the stomach is infrequent, accounting for approximately 1%-2% of all benign gastric tumors and about 1% of gastric stromal tumors. The clinical presentation is often nonspecific, with symptoms varying based on the tumor’s size and location, as well as the presence of mucosal ulceration. This article will present a rare case of a gastric glomus tumor that presented with upper gastrointestinal bleeding. An otherwise healthy 76-year-old man presented to the emergency department with dizziness and melena. An upper gastrointestinal endoscopy revealed a 15-mm-diameter ulcer in the greater curvature of the antrum. A 40 × 34-mm lesion extending exophytically at the level of the greater curvature of the stomach was observed in computed tomography. With imaging and endoscopy findings suggestive of an intramucosal neoplasm of the stomach greater than 3 centimeters in diameter, surgical intervention was decided upon. A tumor with a diameter of 5 cm was excised, and microscopic examination revealed a relatively well-circumscribed, multinodular tumorous lesion located in the muscularis propria, with focal infiltration into the submucosa, separated by muscle fibers. The tumor was composed of monotonous cells with regular chromatin distribution, small round nuclei, and focal clear cytoplasm, forming solid islands around vessels within a hyalinized stroma. Immunohistochemical studies showed the tumor was positive for smooth muscle actin, calponin, caldesmon, and Type 4 collagen, consistent with a diagnosis of glomus tumor. In this case report, a rare cause of gastrointestinal bleeding is presented: a glomus tumor. Preoperative radiological assessment of the tumor suggested a gastrointestinal stromal tumor. A wedge resection with clean surgical margins was performed, and the definitive diagnosis was subsequently confirmed through immunohistochemical evaluation.


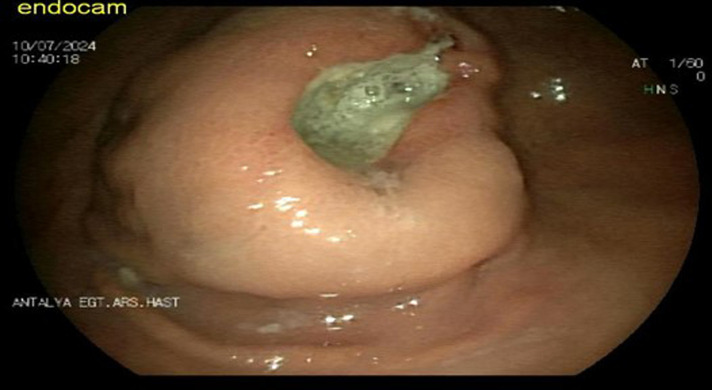


Figure 1. Esophagogastroduodenoscopy (EGD) showed a 1- to 1.5-cm-diameter ulcer in the greater curvature of the antrum, which was raised from the mucosa with a depressed, crater-like center and an exudative surface.


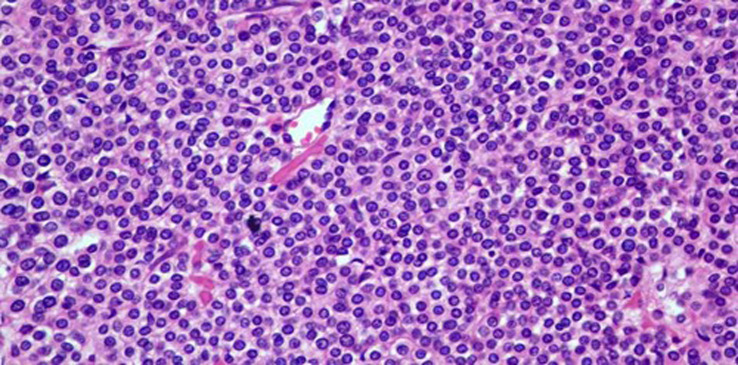


Figure 2. Photomicrograph of gastric glomus tumor. Monotonous tumor cells with round nuclei forming solid islands around thin-walled, narrow-lumened vascular structures. (Hematoxylin and eosin 40× magnification).

## PS-024 A Nationwide Cross-sectional Study on Endoscopy-Related Musculoskeletal Injuries: The Hidden Occupational Burden of Gastroenterologists in Türkiye

**Halit Kandemir, Ali Karataş, Gülden Bilican, Kenan Moral, Beril Demir, Enes Cömert, Derya Kirman, Yusuf Can Yılmaz, Güner Kılıç, Çağdaş Kalkan, Murat Kekilli, Tarkan Karakan, Mehmet Cindoruk**

Department of Gastroenterology, Gazi University Faculty of Medicine, Ankara, Türkiye

**Background/Aims: **Endoscopic procedure-related musculoskeletal injuries (ERMI) are a major problem for gastroenterologists. ERMI arise from increased workload, extended times of advanced endoscopic procedures, and repetitive overuse. There has been no study on ERMI among gastroenterologists in Türkiye. This study aimed to assess the frequency, causes, and risk factors associated with ERMI among gastroenterologists in Türkiye.

**Materials and Methods: **A 26-item electronic survey was sent to 1100 Turkish Gastroenterology Association physician members and to 253 gastroenterology fellows still in training. Demographic characteristics, prevalence of ERMI, workload parameters, and possible risk factors were evaluated using univariate and multivariate analyses.

**Results: **The survey was completed by 132 gastroenterologists, 78% of respondents were male, with a mean professional experience of 11 years. Overall, 72% reported experiencing at least 1 ERMI during their career, and 63.2% reported injuries involving multiple joints. The most commonly affected areas were the neck, left thumb, and right wrist. Significant differences in ERMI were observed based on gender, affiliated institution, and total weekly number of procedures. Regarding injury severity, sex and glove size were significant factors. In logistic regression analysis, female sex increased the risk of ERMI by 5.6 times, and performing ERCP procedures increased the risk by 2.6 times.

**Conclusion: **ERMI is highly prevalent among gastroenterologists in Türkiye and represents a significant occupational health problem with implications for work loss. Preventive strategies such as reducing the volume of procedures, increasing the frequency of breaks, and making ergonomic adjustments in procedure rooms are necessary to reduce the risk of ERMI.

## PS-025 Oxymetholone-associated Acute Liver Failure

**Hasan Utku Kocal, Mehmet Kapan, Etibar Mammadov, Nejla Küçük, Murat Başaran, Mehmet Suat Yalçın, Burak Özşeker**

Division of Gastroenterology, Muğla Training and Research Hospital, Muğla Sıtkı Koçman University, Muğla, Türkiye

Oxymetholone is a synthetic derivative classified as an anabolic-androgenic steroid, primarily indicated for the treatment of certain hematological disorders, particularly anemia. However, prolonged use or administration at high doses has been associated with significant hepatotoxicity. The hepatotoxic adverse effects of anabolic steroids include a range of hepatic pathologies, such as intrahepatic cholestasis, peliosis hepatis, hepatic adenoma, and hepatocellular carcinoma. Although rare, acute liver failure secondary to oxymetholone use is a life-threatening complication. This case report presents the clinical course and management of a patient who developed acute liver failure following oxymetholone use and required intensive care unit monitoring. By contributing to the limited number of reports in the existing literature, this case aims to raise awareness of this rare but severe complication and to explore potential underlying mechanisms, thereby enhancing the understanding of anabolic steroid-induced hepatotoxicity.

## PS-026 Patient-Reported Outcomes After Microcurrent Electrical Stimulation Therapy Compared to Conventional Medical Therapy for Chronic Anal Fissure: A Follow-Up Study

**Büşra İnal ^1^ , Neriman Şengül ^2^ , Özden Arısoy ^3^ , Nuriye Özengin ^1^**

^1^Department of Physiotherapy and Rehabilitation, Bolu Abant İzzet Baysal University Faculty of Health Sciences, Bolu, Türkiye

^2^Department of General Surgery, Bolu Abant İzzet Baysal University Faculty of Medicine, Bolu, Türkiye

^3^Department of Psychiatry, Bolu Abant İzzet Baysal University Faculty of Medicine, Bolu, Türkiye

**Background/Aims: **Chronic anal fissure (CAF) is a painful disorder that is often resistant to medical therapy. Microcurrent electrical stimulation (MES) has recently emerged as a noninvasive alternative aimed at promoting wound healing and symptom relief. This study aimed to compare patient-reported outcomes in patients with CAF treated with combined MES and standard medical treatment compared to standard medical treatment.

**Materials and Methods:** Patients who had completed combined MES therapy (3 days/week for 4 weeks; MES, diaphragmatic breathing, defecation training, and topical diltiazem) or conventional medical treatment (topical diltiazem) approximately 10 months prior were evaluated using a telephone survey. Perceived improvements in pain, bleeding, constipation, quality of life, and disease improvement were assessed using a 4-point Likert scale (1 = worse, 2 = no change, 3 = improved, 4 = much improved). Additionally, current pain intensity was measured using a 10-point Likert scale.

**Results:** Twenty-nine patients (15 MES, 14 conventional; 26 F/3 M, mean age 42 years) participated in the follow-up. Baseline demographics and Wexner constipation scores were similar between the groups (*P* > .05). The MES group reported significantly greater improvement in pain (*P* = .039), bleeding (*P* = .007), and constipation symptoms (*P* = .005). However, current pain severity scores, quality of life, and perception of disease improvement were similar between the groups (*P* > .05).

**Conclusion:** From the patient-reported outcome perspective, adjuvant MES therapy offers limited benefit to overall quality of life and disease improvement status despite significant symptomatic relief.

## PS-027 Does Chronic Anal Fissure Affect Physical Activity?

**Büşra İnal ^1^ , Neriman Şengül ^2^ , Özden Arısoy ^3^ , Nuriye Özengin ^1^**

^1^Department of Physiotherapy and Rehabilitation, Bolu Abant İzzet Baysal University Faculty of Health Sciences, Bolu, Türkiye

^2^Department of General Surgery, Bolu Abant İzzet Baysal University Faculty of Medicine, Bolu, Türkiye

^3^Department of Psychiatry, Bolu Abant İzzet Baysal University Faculty of Medicine, Bolu, Türkiye

**Background/Aims:** Chronic anal fissure (CAF) is a painful and distressing anorectal condition that reduces quality of life (QOL). Patients with CAF are known to experience pain during and after defecation, as well as during physical activities. In this study, the aim was to compare the physical activity levels of patients with chronic constipation (CC) with and without CAF.

**Materials and Methods:** Twenty-four patients with CC-CAF (F/M = 20/4, mean age 41.25) and 14 patients with CC only (F/M = 13/1, mean age 41.92) were included in the study. Physical activity levels were assessed using the International Physical Activity Questionnaire (IPAQ). Constipation-related quality of life and symptom severity were evaluated using the Patient Assessment of Constipation Quality of Life (PAC-QOL), the Constipation Severity Instrument, and the Wexner Constipation Scoring System. In addition, the intensity of pain during defecation and sitting was assessed using a 10-point Likert scale.

**Results:** The physical and sociodemographic characteristics of the 2 groups were similar (*P* > .05). The majority of patients with CC-CAF were physically inactive, whereas most patients with CC only were minimally active; however, the groups were comparable in terms of physical activity (*P* > .05). Furthermore, constipation-related quality of life and symptom severity were similar between the groups (*P* > .05).

**Conclusion:** To the best of the author’s knowledge, this is the first study to evaluate physical activity levels in patients with chronic anal fissure. These results showed that physical activity levels were low in both groups, regardless of the presence of CAF. This finding indicates that patients with constipation have low physical activity levels, and the presence of an anal fissure does not affect physical activity.

## PS-031 A Rare Complication of Biliary Stenting: Proximal Migration of a Double Pigtail Stent

**Duran Deha Çetin, Şehmus Ölmez, Bünyamin Sarıtaş, Mustafa Harı, Yılmaz Çelik, Banu Kara**

Department of Gastroenterology, Adana City Training and Research Hospital, University of Health Sciences, Adana, Türkiye

Endoscopic biliary stenting is an effective method for relieving biliary obstruction; however, stent-related complications occur in 8%-10% of cases. Common adverse events include cholangitis, cholecystitis, perforation, pancreatitis, bleeding, and occlusion. Stent migration is less frequent, reported in 5%-10% of patients. Among available devices, the double pigtail stent is considered less prone to migration than straight plastic stents. A rare case of proximal migration of a double pigtail stent is reported. A 70-year-old man with diabetes mellitus presented with 15 days of nausea, vomiting, abdominal pain, and fever. He was hemodynamically stable, with tenderness in the right upper quadrant. One year earlier, he had undergone endoscopic retrograde cholangiopancreatography (ERCP) for choledocholithiasis, during which a stent was placed for an impacted stone; no revision had been performed since. Laboratory tests showed total/direct bilirubin levels of 7/5 mg/dL, ALT 150 U/L, AST 109 U/L, GGT 714 mg/dL, CRP 102 mg/L, and WBC 11 000/µL. Ultrasonography demonstrated a hydropic gallbladder with sludge, dilated intrahepatic ducts, and a 15-mm common bile duct containing sludge and a stent. With a presumptive diagnosis of cholangitis, the patient underwent ERCP. Endoscopy revealed a periampullary diverticulum and a prior sphincterotomy, but no stent was protruding from the papilla. Fluoroscopy identified a proximally migrated stent. Using a balloon catheter, the stent was retrieved into the duodenum with drainage of abundant sludge. Despite repeated balloon sweeps, biliary clearance remained incomplete due to impacted stones; therefore, a straight plastic stent was inserted. The patient’s cholestatic and infectious markers normalized, and he was discharged on day 5. Although double pigtail stents are less likely to migrate, this case demonstrates that proximal migration may still occur and lead to obstruction. Awareness of this complication and timely endoscopic intervention are essential.


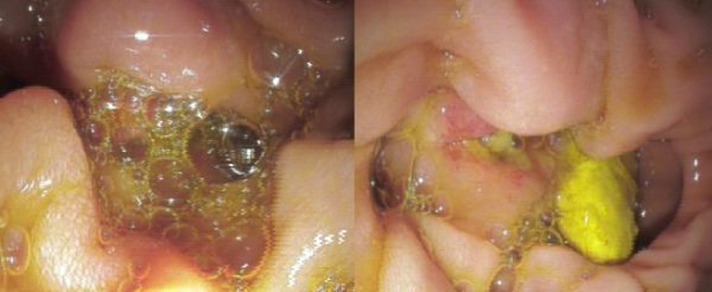


Figure 1. Papilla at the edge of a periampullary diverticulum with a migrated stent.


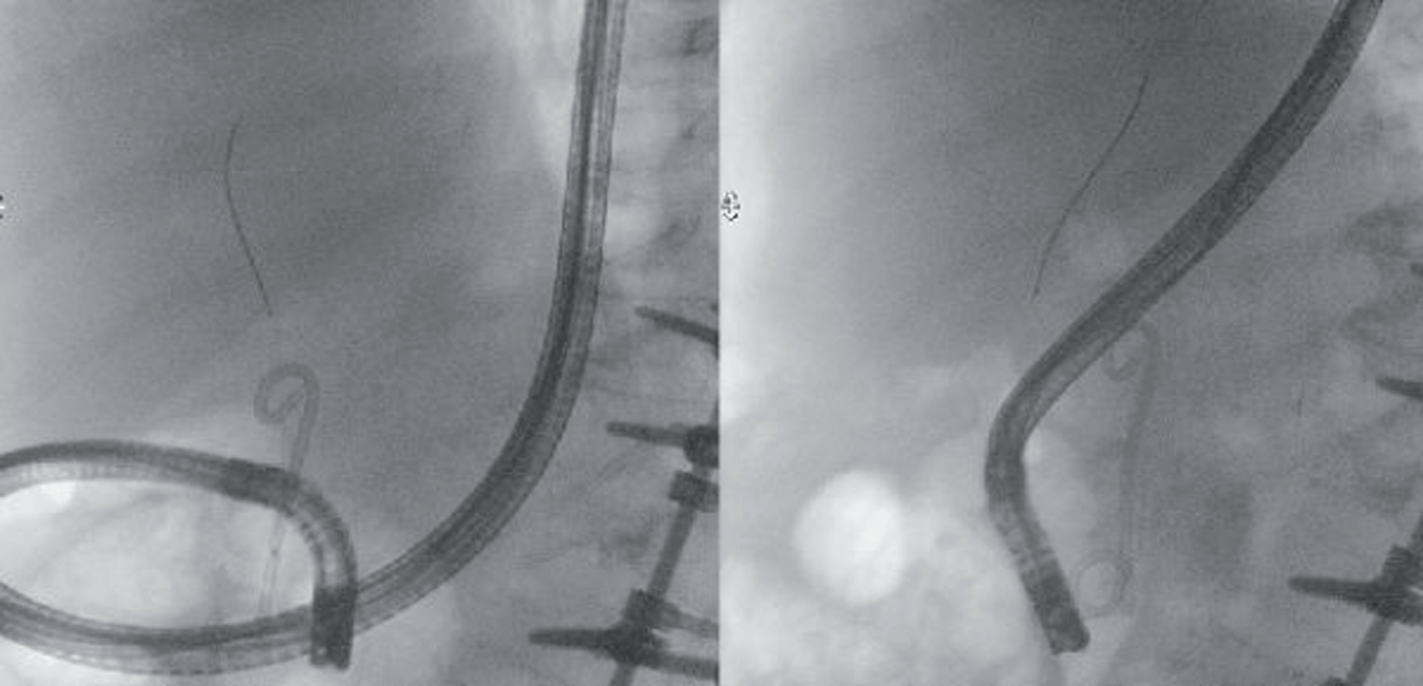


Figure 2. Fluoroscopic appearance of the proximally migrated stent.


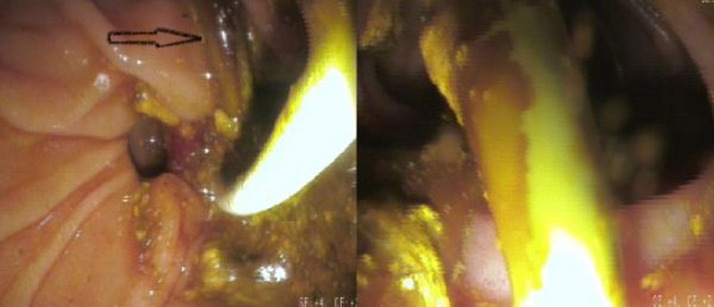


Figure 3. Stent visualized at the papilla after balloon-assisted extraction.

## PS-034 Surprise in a Patient Presenting with Rectal Bleeding and Pain; Gastric Mucosa in the Rectum: Case Report

**Hatice Rızaoğlu Balcı**

Department of Gastroenterology, VM Medical Park Mersin Hospital, Mersin, Türkiye

Gastric heterotrophy is frequently observed in the intestinal tract. It is most commonly seen in the esophagus and small intestine but rarely in the colon; the rectum is the most commonly affected area. Its appearance can be confused with a polyp. A 39-year-old woman presented to the gastroenterology clinic with complaints of pain and bleeding. The patient had previously been diagnosed with a tailgut cyst, and these symptoms suggested complications such as compression and malignant transformation from this cyst. A colonoscopy was scheduled for the patient. Rectal examination was normal, but colonoscopy revealed a sessile polyp-like mass approximately 3 cm in size, located 4 cm proximal to the dentate line, with folds resembling gastric folds, prominently raised rectal mucosa, and a soft consistency. There was no ulcer on this lesion that can explain the rectal bleeding. Because the normal mucosa ruled out the possibility of a polyp, multiple biopsies were taken from the lesion, and the procedure was completed. Bleeding was thought to be due to hemorrhoids. Pathological examination revealed ectopic gastric mucosa. Ectopic gastric mucosa is the presence of gastric tissue in a region outside the stomach. CDX2 mutations can lead to the development of ectopic lesions with a gastric phenotype in the midgut endoderm. Although most commonly seen in the gastrointestinal tract, they have also been described in the spinal cord, mediastinum, and scrotum. Within the gastrointestinal tract, they are frequently found in the esophagus and small intestines, but rarely in the colon. The rectum is the most common site in the colon. It can cause complaints such as rectal bleeding, tenesmus, and rectal pain in patients. In this case, the patient complained of rectal pain and bleeding. Most cases are located more than 5 cm proximal to the anal verge. They are often mimicked by structures such as diverticula and polyps. In this case, the lesion was observed at the 4 cm mark of the rectum and appeared to be a sessile polyp. Because the knowledge of this lesion is based entirely on case reports, there are no current guidelines on optimal treatment and follow-up.


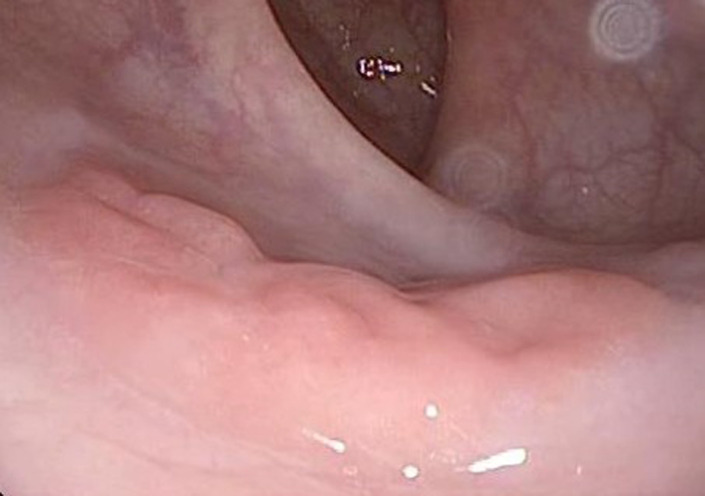
 
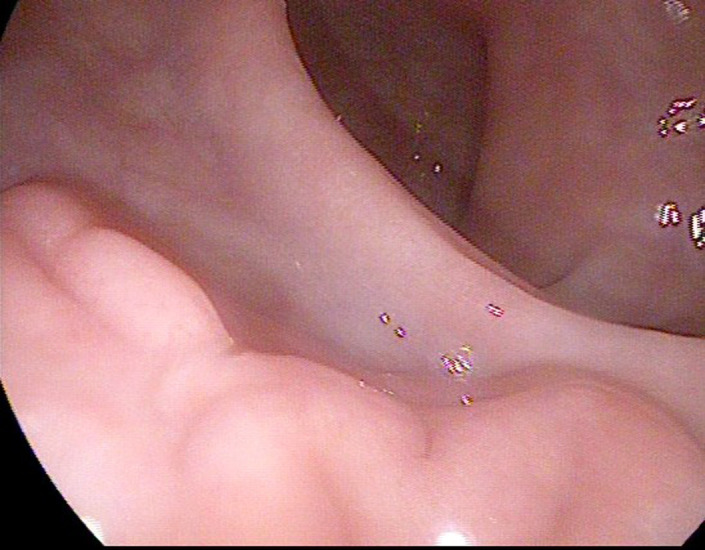


Figure 1. Ectopic gastric mucosa in the rectum (endoscopic image).

## PS-035 Liver Transplant Recipient with Refractory Pruritus and Diarrhea: Posttransplant Lymphoproliferative Disorder

**Khumara Rzayeva ^1^ , Tuğba Tolu Bülte ^2^ , Coşkun Özer Demirtaş ^2^**

^1^Department of Internal Medicine, Marmara University Faculty of Medicine, İstanbul, Türkiye

^2^Department of Gastroenterology, Marmara University Faculty of Medicine, İstanbul, Türkiye

Posttransplant lymphoproliferative disorder (PTLD) is a serious malignancy associated with solid organ transplantation, Epstein–Barr virus (EBV) infection, and immunosuppression. Clinical presentations are heterogeneous and often mimic gastrointestinal symptoms. The incidence of PTLD after liver transplantation is 1%-3%, ranging from asymptomatic lymphoid proliferation to aggressive lymphoma. Gastrointestinal involvement is common and usually presents with nonspecific symptoms. A rare case of PTLD presenting with pruritus and diarrhea is reported. A 49-year-old man with autoimmune hepatitis underwent cadaveric liver transplantation in 2017. His initial immunosuppressive regimen included tacrolimus, but he developed recurrent pruritus and diarrhea. Colonoscopy revealed mucosal abnormalities, and biopsies were consistent with kappa-restricted polymorphic PTLD. EBER-CISH demonstrated focal EBV positivity. PET-CT showed segmental FDG uptake in the terminal ileum and ascending colon, along with nonmetabolically active splenomegaly. Tacrolimus was discontinued, everolimus was started, and 4 cycles of rituximab were administered. Six- and 12-month follow-ups showed lesion regression and decreased FDG uptake. PTLD is a major cause of morbidity and mortality after liver transplantation. It is usually EBV-driven under immunosuppression, although late-onset EBV-negative PTLD is being increasingly reported. Gastrointestinal PTLD often presents with nonspecific symptoms; endoscopy and biopsy are essential for diagnosis. PET-CT is useful for staging and monitoring response, but its sensitivity is limited in mucosa-limited disease. Reduction of immunosuppression is the first-line therapy, balanced against the risk of graft rejection. Rituximab, with approximately 60% remission rates, is the first-line therapy for polymorphic and monomorphic PTLD; early initiation improves survival. Switching from tacrolimus to everolimus reduces the risk of malignancy while preserving graft function. This case highlights pruritus as an early atypical symptom, emphasizing the need for careful evaluation of unusual presentations. Nonspecific gastrointestinal symptoms and pruritus in liver transplant recipients should raise suspicion for PTLD. Early biopsy, adjustment of immunosuppression, and rituximab therapy are essential. Transitioning to everolimus may reduce the risk of malignancy.

## PS-036 Study of the Performance of MELD, MELD-Na, and MELD 3.0 Scores in Predicting in-Hospital Mortality in Patients with Cirrhosis

**Omaima Ghallab, Aicha Darif**

Department of Hepatology and Gastroenterology, Ibn Rochd University Hospital, Casablanca, Morocco

**Background/Aims:** The Model for End-Stage Liver Disease (MELD) has been updated to MELD-Na and, more recently to MELD 3.0, which includes albumin and a correction for female gender, thereby improving both mortality prediction and fairness in liver transplant allocation. The aim was to calculate MELD 3.0 prognostic scores and compare them with MELD-Na and MELD in hospitalized patients with cirrhosis.

**Materials and Methods:** A single-center, retrospective, descriptive, and analytical study that included all patients admitted for cirrhosis decompensation between January 2022 and December 2024 was conducted. Demographic and clinical-biological data were collected. MELD, MELD-Na, and MELD 3.0 scores were calculated. Receiver operating characteristic (ROC) curve analysis was performed to compare the performance of these scores in predicting in-hospital mortality.

**Results:** A total of 176 patients were included (median age 54 years [IQR: 18-79]; 59.1% men, 40.9% women; sex ratio 1.45). The etiologies of cirrhosis were HCV (27%), HBV (12%), AIH (7.5%), NASH (9.7%), alcohol (2.8%), and other causes (41%). Child-Turcotte-Pugh classification was A: 4.5%, B: 22%, C: 73.5%. Median MELD 3.0, MELD-Na, and MELD scores were 22.3 (IQR: 10.8-38.8), 25.8 (IQR: 7.4-47.9), and 19 (IQR: 9-32), respectively, with an overall in-hospital mortality rate of 18.8%. AUROC values for MELD 3.0, MELD-Na, and MELD in predicting mortality were 0.786, 0.705, and 0.754 (*P* < .05), respectively. The optimal MELD 3.0 threshold for predicting in-hospital mortality was >29.9 (sensitivity = 69.7%, specificity = 90.6%).

**Conclusion:** MELD 3.0 outperforms MELD-Na and MELD in predicting in-hospital mortality among patients with cirrhosis.

## PS-038 Concurrent Pleural and Peritoneal Mesothelioma Associated with Asbestos Exposure: A Rare Case Report

**Selcan Cesur, Ali Türeyen, Berrin Yalınbaş Kaya**

Department of Gastroenterology, Eskişehir City Hospital, Eskişehir, Türkiye

Malignant mesothelioma is a rare but aggressive tumor strongly associated with asbestos exposure. Although most cases arise from the pleura, only 6%-10% originate from the peritoneum. Due to its nonspecific symptoms, early diagnosis remains challenging. A 60-year-old male patient was evaluated for increased acute-phase reactants in the infectious diseases clinic and was referred to the unit for investigation of iron deficiency anemia and constipation. Upper endoscopy revealed a 2 × 2 cm ulcerated polypoid lesion in the gastric fundus, whereas colonoscopy showed 2 2 × 2 cm masses in the ascending colon and a 4 × 3 cm ulcerated mass in the proximal transverse colon. The patient reported living in an asbestos-painted house until the age of 11 and spending summers there thereafter. Family history was significant for pleural mesothelioma, lung cancer, and colon cancer. In 2022, a lung nodule was detected and subsequently diagnosed as pleural mesothelioma; he received radiotherapy and chemotherapy, followed by immunotherapy, which was discontinued due to adverse effects. Ten days prior, he presented to the emergency department with subileus. Clinical and histopathological evaluation confirmed malignant peritoneal mesothelioma concurrent with pleural mesothelioma. Asbestos exposure is the most significant risk factor for both pleural and peritoneal mesotheliomas, although their molecular pathogeneses differ. This case is extremely rare due to both the familial cancer history and concurrent involvement. Considering the diagnostic difficulties, malignant mesothelioma should always be considered in patients with relevant risk factors.

## PS-039 Treatment of Patients with Plummer-Vinson Syndrome

**Natavan Hıdırova, Tarverdi Rzayev, Amrah Ahmedov**

Department of Surgical Diseases, Azerbaijan Medical University Faculty of Medicine, Baku, Azerbaijan

**Background/Aims: **Plummer-Vinson syndrome is a rare disease characterized by the classic triad of dysphagia, membranous esophageal stricture, and iron deficiency anemia. It is mainly observed (in 90% of cases) in white women aged 40-70 years. In patients, the symptoms of anemia are more pronounced, and dysphagia is painless and either transient or gradually progresses over the years. The diagnosis is made based on laboratory findings and endoscopic examination. Patients with Plummer-Vinson syndrome have an increased risk of developing esophageal carcinoma. The aim of this study is to investigate the treatment of patients diagnosed with Plummer-Vinson syndrome in the clinic.

**Materials and Methods:** During 2018-2025, 5 female patients with Plummer–Vinson syndrome were admitted to the clinic. The average age of the patients was 51 years. The duration of the disease ranged from 5 to 9 years. Grade 1 dysphagia was observed in 3 patients, and grade 2 dysphagia in 2 patients. At admission, hemoglobin levels ranged from 5.6 to 8.9 g/dL. During endoscopic examination, a membranous stricture was detected in the upper part of the esophagus. Hemoglobin levels in all patients were corrected under the supervision of a hematologist, and balloon dilatation of the esophageal stricture was performed in 4 patients.

**Results:** The hemoglobin level of all 5five patients increased after treatment by a hematologist (prescription of iron preparations) to levels between 11.8 and 12.5 g/dL. In 1 patient, symptoms (dysphagia, pain) resolved after medical treatment, and balloon dilatation was not required. In 2 patients, a single session of balloon dilatation was sufficient to relieve dysphagia. In the remaining 2 patients, balloon dilatation was performed twice with an interval of 5 days. Improvement in swallowing was noted after the first session.

**Conclusion:** Patients with Plummer-Vinson syndrome respond well to treatment. Administration of iron preparations and balloon dilatation of the esophagus yield positive results. Continuous follow-up by hematologists and endoscopists is recommended for these patients.

## PS-040 Application of Endoscopic Hemoclips in Cases of Perforation of the Esophagus by Foreign Bodies

**Tarverdi Rzayev, Natavan Hıdırova, Eldar Aliyev, Amrah Ahmadov**

Department of Surgical Diseases, Azerbaijan Medical University Faculty of Medicine, Baku, Azerbaijan

**Background/Aims: **Esophageal perforations (EP) are rare pathologies that require urgent treatment. If not treated promptly, they result in death in 5%-30% of cases. Typical symptoms include pain, odynophagia, dyspnea, subcutaneous crepitus, and fatigue. In late cases, symptoms such as high fever, tachycardia, and hypotension can lead to complications with high mortality, including respiratory failure and sepsis. This study aims to investigate the results of endoscopic hemoclip application in cases of esophageal perforation caused by foreign bodies.

**Materials and Methods:** The results of 10 patients treated for esophageal perforation due to foreign bodies in the clinic over the past 10 years were analyzed. Six of the patients were male, and 4 were female, with a mean age of 42.8 years (53 ± 8.32). During endoscopic treatment, primary suture with hemoclip was performed in patients with perforations smaller than 2.0 cm. All patients were prescribed conservative treatment, including discontinuation of oral food intake, total parenteral nutrition, antibiotics, and proton pump inhibitors.

**Results:** Foreign body perforation of the esophagus was detected in 10 patients. In 7 of them, the perforation was noted in the thoracic region, and in 3 in the cervical region. The wounds of 6 patients with perforation in the thoracic region and 1 patient with perforation in the cervical region were closed primarily with endoscopic hemoclips. Thus, in patients whose perforations in the esophagus were smaller than 2.0 cm and had occurred within the previous 24 hours, the wounds were closed with endoscopic hemoclips while conservative treatment was performed concurrently. The wounds of all patients healed primarily and they were sent for outpatient treatment after 1 week.

**Conclusion:** In cases of esophageal perforation by a foreign body, primary closure with an endoscopic clip is recommended if less than 24 hours have passed and the perforation hole is smaller than 2.0 cm.


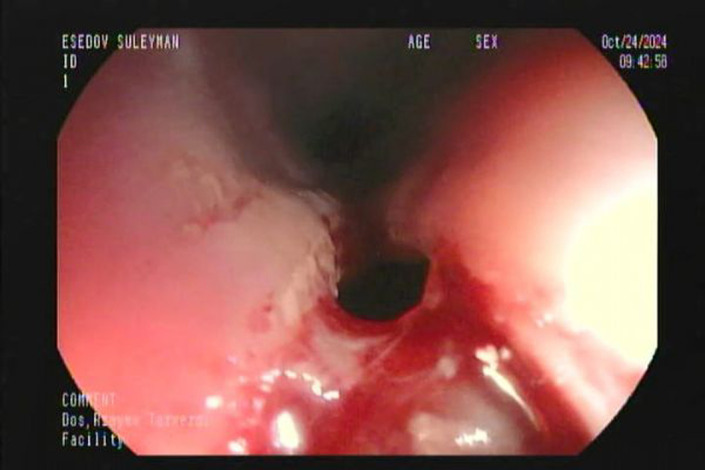


Figure 1. Esophageal perforation.


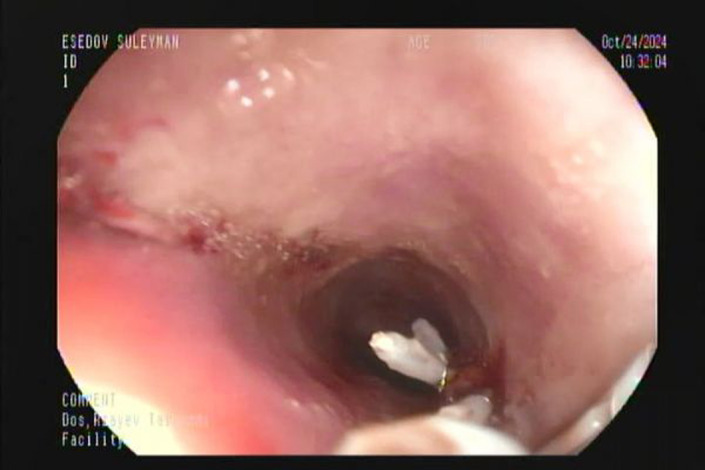


Figure 2. Endoscopic closure of the esophageal perforation with hemoclips.

## PS-041 Use of Biological Agents in Pediatric Inflammatory Bowel Disease: A Single-Center Experience

**Samet Demir ^1^ , Günsel Kutluk ^2^**

^1^Department of Pediatrics, Başakşehir Çam ve Sakura City Hospital, University of Health Sciences, İstanbul, Türkiye

^2^Department of Pediatric Gastroenterology, Başakşehir Çam ve Sakura City Hospital, University of Health Sciences, İstanbul, Türkiye

**Background/Aims:** This study aimed to evaluate the effectiveness of biological agents in pediatric inflammatory bowel diseases (IBD). The response to biological therapy, growth and development parameters, laboratory findings, and changes in disease activity indices were analyzed in patients diagnosed with Crohn’s disease (CD) and ulcerative colitis (UC).

**Materials and Methods:** A retrospective analysis was conducted on 44 pediatric IBD patients who were followed up at the Başakşehir Çam and Sakura City Hospital Pediatric Gastroenterology Clinic between 2020 and 2024 and had started biological therapy due to inadequate response to conventional treatment. The clinical and laboratory characteristics at diagnosis, as well as growth and development parameters, inflammatory markers, and disease activity scores before and after biological therapy (at 0, 3, and 6 months), were assessed.

**Results:** Of the 44 patients included in the study, 25 were diagnosed with Crohn’s disease and 19 with ulcerative colitis. The patients received infliximab or adalimumab, and significant improvements in disease activity scores were observed during treatment. The use of biological agents positively affected growth and development parameters, and a decrease in inflammatory markers was detected. Patients who started treatment earlier demonstrated better clinical responses.

**Conclusion:** Biological agent therapy suppresses inflammation and positively affects growth and development in pediatric patients with IBD. It is an effective and reliable option for patients who do not respond to conventional treatments, positively influencing the disease course, laboratory values, and clinical symptoms.

## PS-044 Unexpected Polypoid Colon Metastasis of Hepatocellular Carcinoma After Long-Term Control with Locoregional Therapy

**Yasemin Armutcuoglu ^1^ , Tuğba Tolu Bülte ^1^ , Emine Bozkurtlar ^2^ , Osman Cavit Özdoğan ^1^ , Coşkun Özer Demirtaş ^1^**

^1^Department of Gastroenterology, Marmara University School of Medicine, İstanbul, Türkiye

^2^Department of Pathology, Marmara University School of Medicine, İstanbul, Türkiye

Hepatocellular carcinoma (HCC) is the most common primary liver malignancy and the third leading cause of cancer-related mortality. Extrahepatic dissemination occurs in 13%-42% of patients, but gastrointestinal (GI) involvement is rare (<2%), with colonic polypoid metastasis reported in only a few cases. Long-term survival after locoregional therapies such as radiofrequency ablation (RFA) and transarterial chemoembolization (TACE) may contribute to these unusual metastatic patterns. A 78-year-old man with hepatitis B virus-related cirrhosis and comorbidities including coronary artery disease, diabetes mellitus, and hypertension was reported. He was diagnosed with HCC in 2012 and underwent the resection of hepatic segment 5. Over the following years, intrahepatic recurrences were managed with 3 sessions of RFA (2014, 2015, 2017) and 1 TACE (2021), achieving long-term intrahepatic disease control. Surveillance magnetic resonance imaging revealed a 38 × 45 mm soft tissue lesion in the left iliac chain, indistinguishable from the sigmoid colon. Colonoscopy identified a 5-mm sigmoid polyp, which was resected. Histopathology confirmed metastatic HCC, with tumor cells showing eosinophilic cytoplasm, atypical nuclei, and strong HepPar-1 positivity, excluding primary colorectal adenocarcinoma. Positron emission tomography-computed tomography (PET-CT) later demonstrated a hypermetabolic pelvic lesion adjacent to the sigmoid colon (SUVmax: 9.48). At diagnosis, liver function was preserved (MELD-Na: 8, Child-Pugh class A5; ECOG: 0). The patient was considered to have metastatic HCC and started on sorafenib, which was discontinued due to neurological adverse effects. Under best supportive care, the patient died 5 months later. Colonic metastasis of HCC is rare, with polypoid presentation estimated at <1%. Spread may occur via hematogenous and lymphatic dissemination or through vascular alterations following locoregional therapies such as RFA or TACE. This case highlights the need for vigilance for atypical metastases in long-term HCC survivors after locoregional therapy and emphasizes the importance of histopathological confirmation and individualized management.


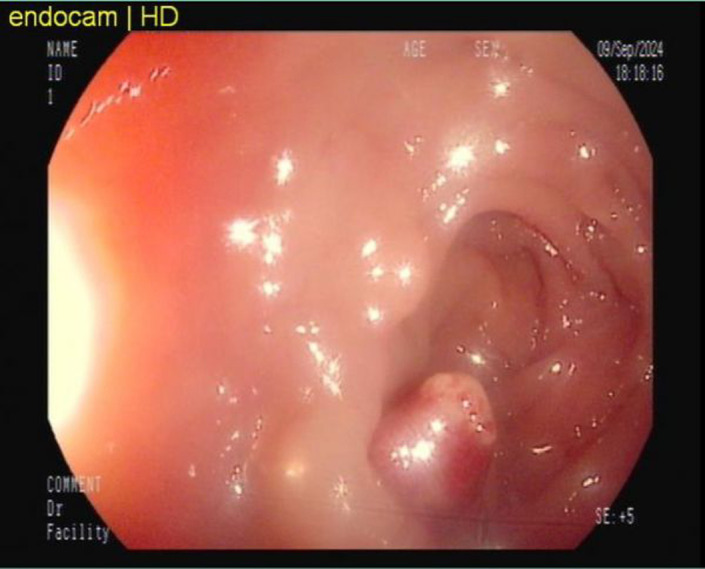


Figure 1. Colonoscopic view of a polypoid lesion in the sigmoid colon representing metastatic hepatocellular carcinoma.


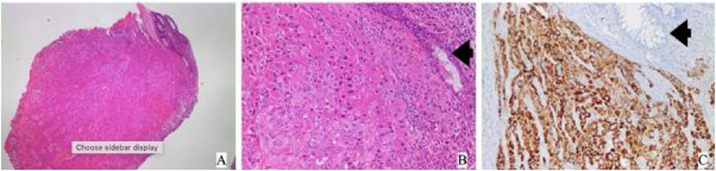


Figure 2. A: Hepatocellular carcinoma metastasis to colonic mucosa, H&E, 4x. B: Neoplastic cells with abundant eosinophilic cytoplasm and centered atypical nucleus of hepatocellular carcinoma, with a colonic crypt on the right upper side of the image (arrow), H&E, 20x. C: Positive immunoreactivity with HepPar1 in neoplastic cells of metastatic hepatocellular carcinoma, with no immunoreactivity in the colonic crypt on the right upper side of the image (arrow), HepPar1, 20x.

## PS-045 Mumps Infection in an Elderly Patient: A Rare Case of Hepatitis and Acalculous Cholecystitis

**Muhammed Furkan Keser ^1^ , Yahya Atayan ^1^ , Zeynep Büşra Keser ^2^ , Yüksel Seçkin ^1^ , Murat Harputluoğlu ^1^ , Mehmet Ali Erdoğan ^1^**

^1^Department of Gastroenterology, İnönü University Faculty of Medicine, Malatya, Türkiye

^2^Department of Infectious Diseases and Clinical Microbiology, İnönü University Faculty of Medicine, Malatya, Türkiye

Mumps is a contagious RNA virus belonging to the Paramyxoviridae family, typically presenting with parotitis. Although generally self-limiting, it may rarely cause complications such as pancreatitis, hepatitis, or nephritis. The association with acalculous cholecystitis is extremely rare. A 79-year-old man presented with jaundice, abdominal pain, fever, and a deteriorating general condition. Laboratory results showed AST 398 U/L, ALT 268 U/L, total bilirubin 48 mg/dL, lipase 3114 U/L, and thrombocytopenia (19 000/µL). Abdominal ultrasound revealed a hydropic gallbladder with wall thickening but no stones. Viral serology demonstrated positive mumps IgM and IgG, whereas other viral markers and autoimmune parameters were negative. The patient was diagnosed with severe pancreatitis, hepatitis, and acalculous cholecystitis. He was treated with supportive care, plasmapheresis, renal replacement therapy, and percutaneous cholecystostomy. Clinical and biochemical recovery was achieved, and the patient was discharged. Mumps infection in the elderly may rarely present with severe complications. Hepatobiliary involvement and acalculous cholecystitis should be considered in the differential diagnosis. This case highlights a rare example of mumps-associated acalculous cholecystitis reported in the literature.

## PS-046 Hepatic Gastrostomy Application in Benign Disease

**Hakan Yıldız ^1^ , Fatih Oğuz Önder ^2^**

^1^Department of Gastroenterology, Yeni Yüzyıl University, İstanbul, Türkiye

^2^Department of Gastroenterology, Acıbadem Mehmet Ali Aydınlar University, İstanbul, Türkiye

ERCP is the primary treatment modality for obstructive bile duct diseases, and in cases of failure (patients with altered anatomy and obstructive tumors), percutaneous transhepatic catheterization (PTC) is an alternative treatment option. However, complications have been reported in 40% of cases with PTC, and it is known that PTC catheters disrupt patients’ daily lives. With the increasing therapeutic use of EUS, its application in malignant bile duct diseases has become widespread as an alternative. EUS drainage of the duodenum to the bile duct is called choledochoduodenostomy, and hepaticogastrostomy refers to the drainage of the bile duct from the stomach. The use of EUS in benign bile duct diseases is increasing. Here, 2 cases of EUS-hepaticogastrostomy in benign bile duct diseases are reported. Case 1: A 55-year-old woman underwent a hepaticojejunostomy after a cholecystectomy 8 years ago, suffering a common bile duct injury. She presented with itching and increased bilirubin levels. MRCP revealed a 20-mm stone. Single-balloon enteroscopy failed to reach the hepaticojejunostomy anastomosis. A fully covered 8-mm metallic stent (SEMS) was placed via hepaticogastrostomy. The SEMS was removed, 12-15 mm balloon dilatation was performed, and the biliary stones were cleared with therapeutic endoscopy. Case 2: A 48-year-old female patient underwent hepaticojejunostomy in 2022 due to a common bile duct injury following a cholecystectomy in 2021. She underwent PTC for HJ stenosis and developed a liver abscess. The patient presented to us with weight loss, jaundice, and a bronchobiliary fistula. An MRI revealed a biliobronchial fistula, HJ anastomotic stenosis, and a dilated IHSY. Because the major papilla cannot be reached with double-balloon enteroscopy, a hepaticogastrostomy was performed, and the HJ stenosis was dilated with a pediatric gastroscope in the following procedure. Conclusion: EUS biliary drainage can be used successfully in biliary tract diseases in cases where ERCP cannot be performed. Its complication rate is lower compared to that of other modalities, and its ability to expand biliary access endoscopically increases procedural success. EUS-HGS can be used as a safe procedure when ERCP is unsuccessful.


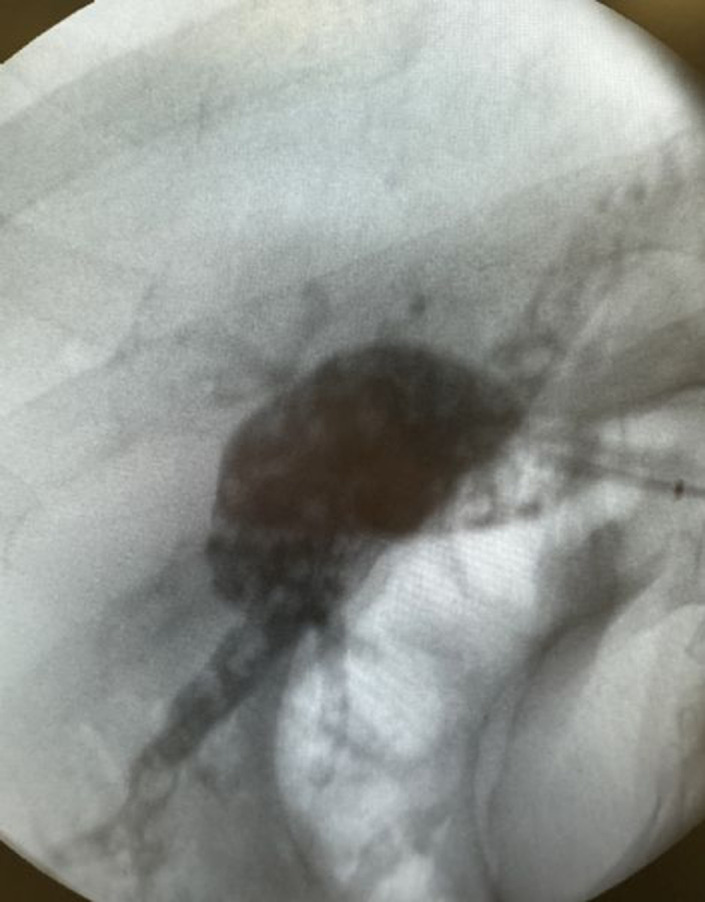


Figure 1. IHSY dilatation and multiple stones.


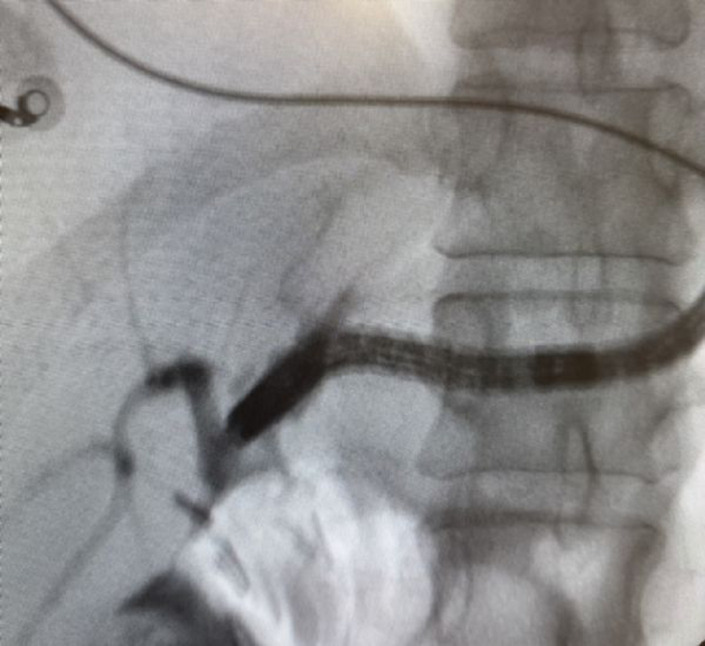


Figure 2. Pediatric gastroscope fluoroscopic view into the bile ducts.


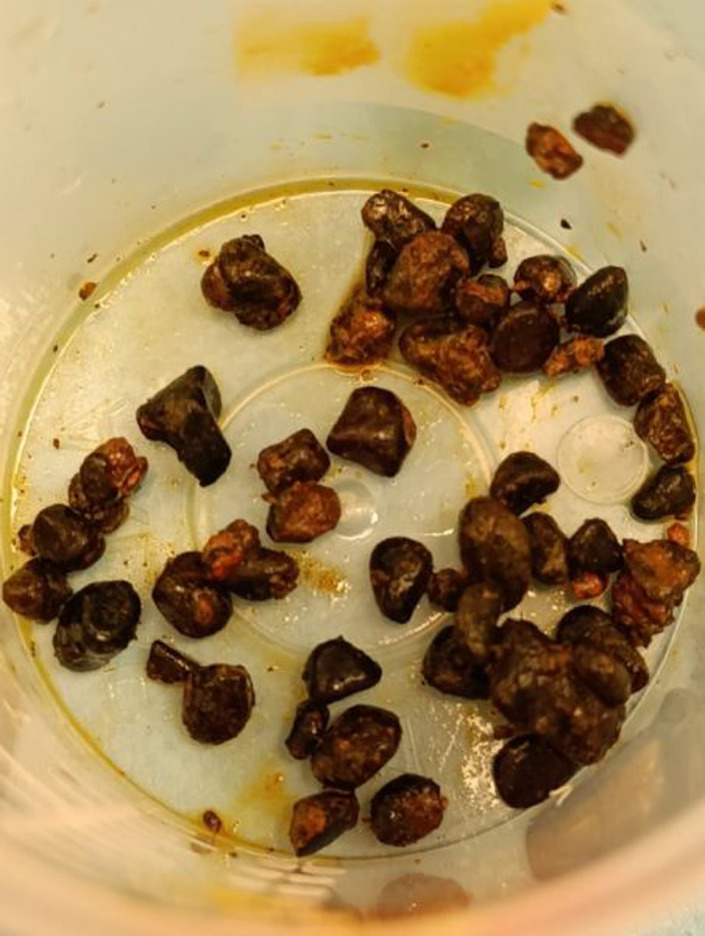


Figure 3. IHSY extracted stone image.

## PS-047 Tubuloside A, A Phenylethanoid Glycoside, Alleviates Diclofenac-Induced Hepato-Nephro Oxidative Injury via Nrf2/HO-1

Ali Türeyen^1^, Hasan Hüseyin Demirel^2^, Ezgi Nur Demirkapı^3^, Azra Mila Eryavuz^4^, Sinan İnce^5^

^1^Department of Gastroenterology, Ministry of Health Eskişehir City Hospital, Eskişehir, Türkiye

^2^Afyon Kocatepe University Bayat Vocational School, Afyonkarahisar, Türkiye

^3^Department of Physiology, Afyon Kocatepe University Faculty of Veterinary Medicine, Afyonkarahisar, Türkiye

^4^Department of Biochemistry, Afyon Kocatepe University Faculty of Veterinary Medicine, Afyonkarahisar, Türkiye

^5^Department of Pharmacology and Toxicology, Afyon Kocatepe University Faculty of Veterinary Medicine, Afyonkarahisar, Türkiye

**Background/Aims:** The major adverse effects associated with the use of nonsteroidal anti-inflammatory drugs (NSAIDs) such as diclofenac (DF) are hepatorenal injuries. Natural antioxidants may be preferred as alternative and/or adjunctive therapeutic approaches to mitigate these effects. Therefore, the present study was conducted to evaluate the protective effect of Tubuloside A (TA) against diclofenac (DF)-induced hepatorenal injury.

**Materials and Methods:** A total of 30 Sprague Dawley rats were used in the study. TA (1 mg/kg, i.p.) and silymarin (25 mg/kg, p.o.) were administered for 5 consecutive days, whereas DF (50 mg/kg, i.p.) was given only on days 4 and 5. No treatment was applied to the control group. The effect of TA on hepatorenal toxicity was evaluated through macroscopic examinations of liver and kidney tissues, biochemical analyses, and mRNA expression analysis of genes involved in the Nrf2/HO-1 signaling pathway.

**Results:** DF administration increased plasma levels of AST, ALT, ALP, BUN, and creatinine. Furthermore, DF treatment increased 8-OHdG and malondialdehyde levels while reducing glutathione, superoxide dismutase, and catalase levels. In addition, DF-induced alterations in the mRNA expression of genes involved in the Nrf2/HO-1 signaling pathway (Nrf2, HO-1, NQO-1, IL-6, iNOS, Cox-2, TNF-α, IL-1β, and NF-κB) and in the apoptotic process (Bcl-2, Cas-3, and Bax), along with histopathological changes in tissues. In contrast, biochemical, histopathological, and molecular findings demonstrated that TA administration protected the liver and kidney against DF-induced damage.

**Conclusion:** In conclusion, DF induced oxidative stress in the liver and kidney by enhancing Nrf2/HO-1 signaling and releasing inflammatory mediators, whereas TA effectively prevented these alterations. These findings suggest that TA may have potential clinical applications in reducing DF-induced hepatorenal toxicity.

## PS-048 Liver Hydatid Cyst as a Rare Cause of Cough: A Case Report

**Yekta Duygu Çimen Beşirli, Halil Yılmaz, Kamil Enli, Mustafa Çelik**

Division of Gastroenterology, Department of Internal Medicine, Pamukkale University Faculty of Medicine, Denizli, Türkiye

Hydatid disease, caused by Echinococcus granulosus, is a zoonotic parasitic infection. Although the liver is the most commonly affected organ, other sites, such as the lungs, may also be involved. Most cases remain asymptomatic; however, clinical manifestations may occur depending on cyst size, location, or the development of complications. One rare complication of hepatic hydatid cysts is bronchobiliary fistula, which may present with atypical symptoms such as cough and bilious sputum. This report aims to highlight an uncommon presentation. A 59-year-old woman presented with a 6-month history of persistent cough and a 3-month history of bilious sputum. Her medical history revealed a previous percutaneous puncture, aspiration, injection, and re-aspiration (PAIR) for a hepatic hydatid cyst. Magnetic resonance imaging demonstrated a cyst in the right hepatic lobe extending to the diaphragm and lung, complicated by bronchobiliary fistula. Albendazole therapy was initiated, and endoscopic retrograde cholangiopancreatography (ERCP) with endoscopic drainage was performed. By day 3 postprocedure, her cough and bilious sputum had improved, and by day 7, the symptoms had completely resolved. Hydatid disease remains a significant health problem in endemic regions such as this country. Although most cases are asymptomatic, the clinical spectrum in complicated cases may vary widely. In patients with unexplained chronic cough and bilious sputum, hepatic hydatid disease should be considered in the differential diagnosis. Bronchobiliary fistula is a rare but serious complication, and imaging studies, along with ERCP, play a pivotal role in diagnosis. ERCP not only allows direct visualization of the fistula but also enables therapeutic interventions. Endoscopic treatment represents a valuable alternative for high-risk surgical patients. With early diagnosis and appropriate management, the prognosis of hydatid disease is generally favorable.


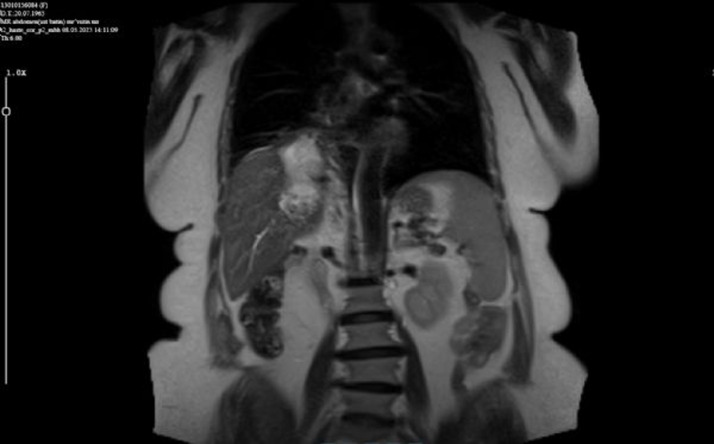


Figure 1. MRI image.


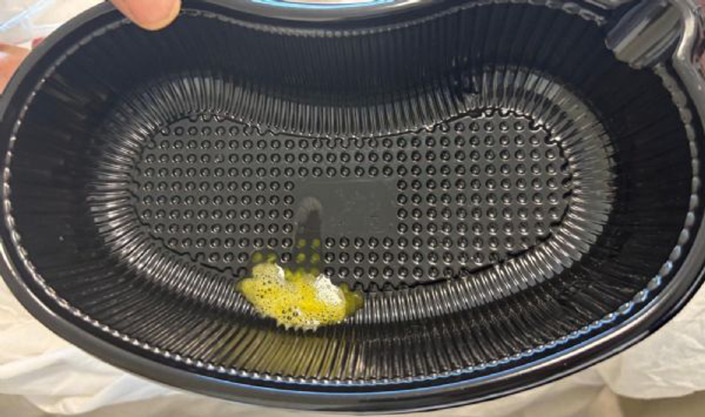


Figure 2. Bilious sputum.

## PS-050 A Rare Cause of Abdominal Pain: Jejunal Diverticulitis

**Müge Erdem Çağı, Berk Baş**

Department of Gastroenterology, Aydın Adnan Menderes University Faculty of Medicine, Aydın, Türkiye

Diverticula may occur throughout the gastrointestinal tract, most commonly in the colon. Clinical presentation is usually asymptomatic, though symptoms vary based on location and complications. Jejunal diverticula are rare, more common in men over 50, and often detected incidentally during imaging. Complications are uncommon, with diverticulitis and perforation being the most frequent. Their rarity leads to limited clinical experience, delayed diagnosis, and presentation after serious complications. A case of jejunal diverticulitis presenting as abdominal pain is reported. A 56-year-old man presented with sudden, severe, midline abdominal pain that disturbed his sleep, accompanied by nausea and fatigue. Five days earlier, he visited another hospital with similar complaints, was diagnosed with intestinal obstruction, improved with symptomatic therapy, and was discharged 2 days prior. His history was unremarkable except for prior gastric hernia surgery. On physical examination, his general condition was good, with a Glasgow Coma Scale score of 15, and his vital signs were normal. There was a laparoscopic operation scar in the abdomen, bowel sounds were increased, and there was tenderness in the left lower quadrant of the abdomen. Laboratory studies revealed increased C-reactive protein (4.55 mg/L; normal 0-0.5), with other values normal. CT enterography demonstrated a 70 mm segment of thickening in the proximal jejunal wall, surrounding fat infiltration, and increased density. Two thick-walled diverticula (21 mm and 13 mm) extended to the mesentery, and abscesses measuring 24×11 mm and 21×20 mm with enhancing walls were identified. Antibiotics were initiated, and a surgical consultation was obtained. After a reduction in abscess size, elective surgery was recommended. The patient’s pain subsided with medical therapy, and he was discharged with plans for elective surgical follow-up. Jejunal diverticulitis and its complications should be considered in patients with intestinal obstruction or findings of acute abdomen.


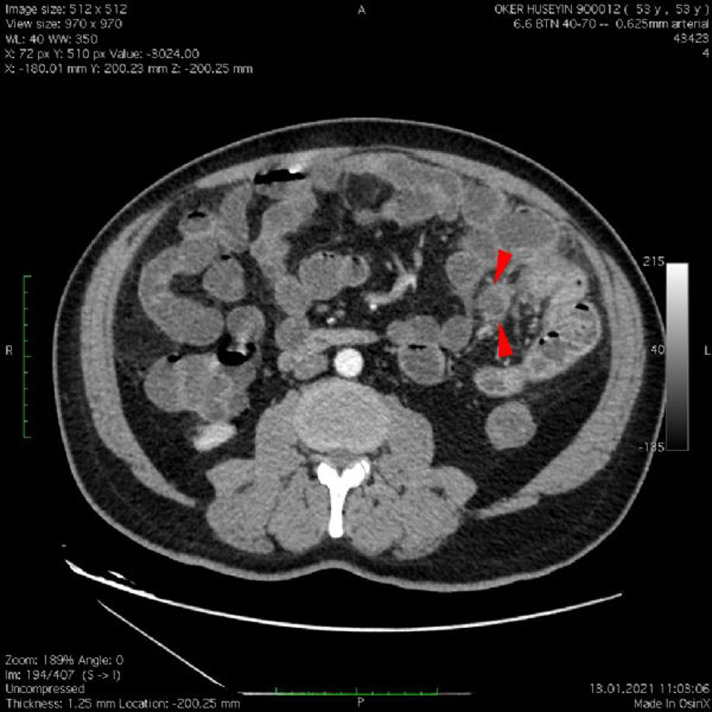


Figure 1. Abscess formation.


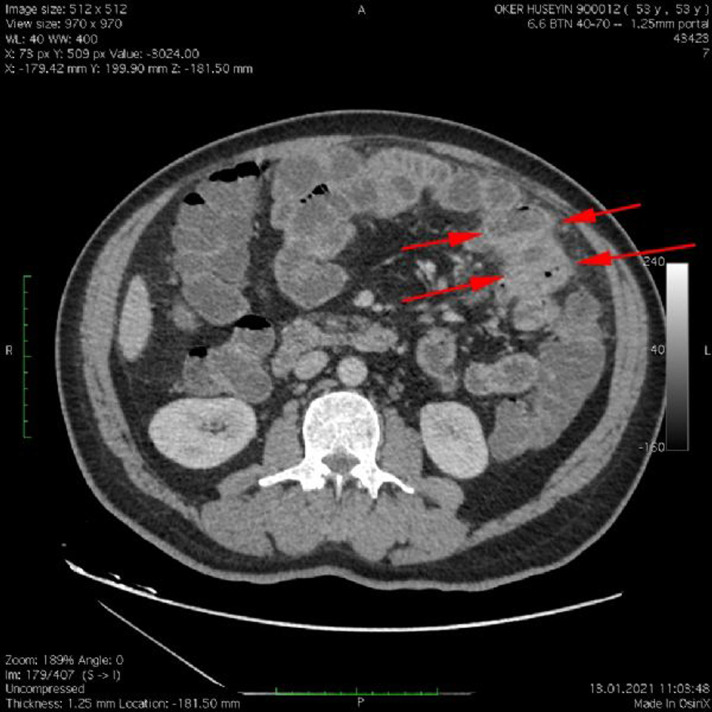


Figure 2. Jejunal loops with thickened walls.

## PS-051 A Rare Cause of Gastrointestinal Bleeding: Colonic Angiosarcoma Metastasis

**Fatih Polat ^1^ , Mehmet Kapan ^2^ , Etibar Mammadov ^2^ , Ferhat Yılmaz ^1^ , Mehmet Suat Yalçın ^3^ , Murat Başaran ^3^ , Havva Özşeker ^4^ , Burak Özşeker ^2^**

^1^Department of Internal Medicine, Muğla Training and Research Hospital, Muğla, Türkiye

^2^Division of Gastroenterology, Muğla Sıtkı Koçman University Faculty of Medicine, Muğla, Türkiye

^3^Department of Gastroenterology, Muğla Training and Research Hospital, Muğla, Türkiye

^4^Department of Pathology, Muğla Training and Research Hospital, Muğla, Türkiye

Angiosarcoma is a rare and aggressive tumor that accounts for 1%-2% of soft tissue tumors arising from lymphoid and vascular endothelial cells. It is typically of cutaneous origin, with metastases most commonly occurring in the lungs, liver, and regional lymph nodes. Colonic metastasis is extremely rare, with only 6 cases reported in the literature. Among these, 3 patients presented with gastrointestinal bleeding. A patient with gastrointestinal bleeding who was diagnosed with metastatic colonic angiosarcoma, marking the seventh case reported in the literature was presented.


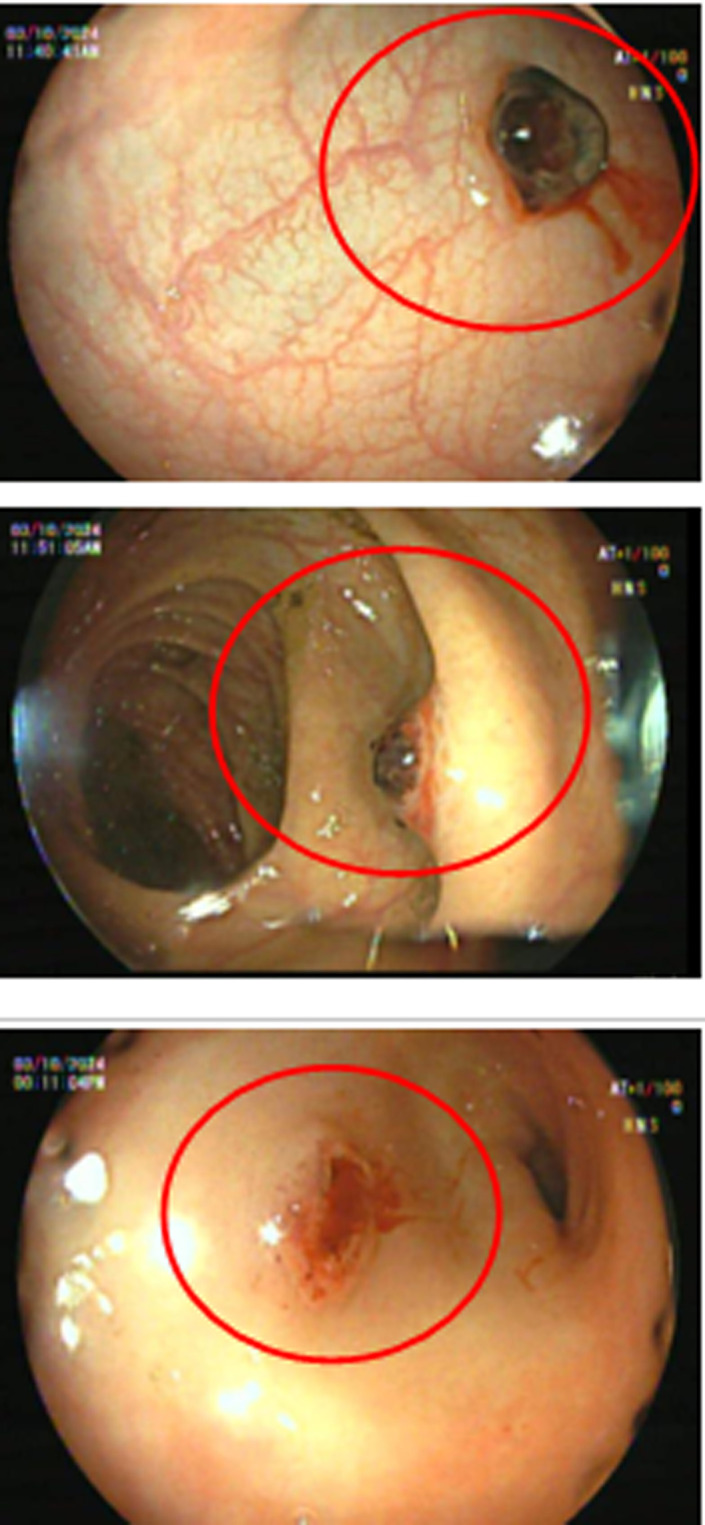


Figure 1. Colonoscopy findings of detected angiosarcomas.

## PS-052 Efficacy of Upadacitinib Treatment in Patients with Inflammatory Bowel Disease: 6-Month Results

**Osman Özdoğan, Serkan Yaraş, Mehmet Kasım Aydın, Oktay Bayraktar, Hasan** **Mammadov, Ahmet Emre Ergan, Ümit Yeşilova, Fehmi Ateş, Engin Altıntaş, Orhan Sezgin**

Division of Gastroenterology, Department of Internal Medicine, Mersin University Faculty of Medicine, Mersin, Türkiye

**Background/Aims:** This study aimed to evaluate the clinical and laboratory efficacy of upadacitinib (UPA) in biologic-experienced patients with moderate-to-severe Crohn’s disease (CD) and ulcerative colitis (UC)

**Materials and Methods:** In this retrospective cohort study, the induction (CD at 3 months, UC at 2 months) and maintenance (6 months) periods were examined. The Harvey-Bradshaw Index (HBI) was used for CD and the Partial Mayo Score (PMS) was used for UC to assess disease severity. Clinical response (CR) was defined as an improvement of ≥ 3 points on HBI and >= 2 points on PMS. Clinical remission (CRM) was defined by scores of HBI < 5 and PMS < 2. Fecal calprotectin (FCAL), CRP, and disease activity scores were also analyzed for patients who discontinued treatment.

**Results:** A total of 20 CD and 23 patients with UC were included in the study, after excluding 7 patients (5 due to lack of follow-up and 2 due to side effects). Two patients (CD = 1, UC = 1) were primary nonresponders, and 3 patients (CD = 2, UC = 1) experienced loss of response. At induction, CR and CRM rates were 80% and 45% for CD, and 90.1% and 52.2% for UC, respectively. At 6 months, the UPA persistence rate was 78.3% for CD and 88% for UC. CR rates were 72.7% for CD and 83.3% for UC, whereas CRM rates were 50% for CD and 58.3% for UC. The proportion of patients with increased CRP and FCAL at baseline significantly decreased by the sixth month in both patient groups.

**Conclusion:** Upadacitinib has demonstrated strong efficacy in a challenging group of biologic-experienced patients with inflammatory bowel disease. High CR and CRM rates, along with significant improvements in objective inflammatory markers, highlight its value as a robust treatment option in this population.


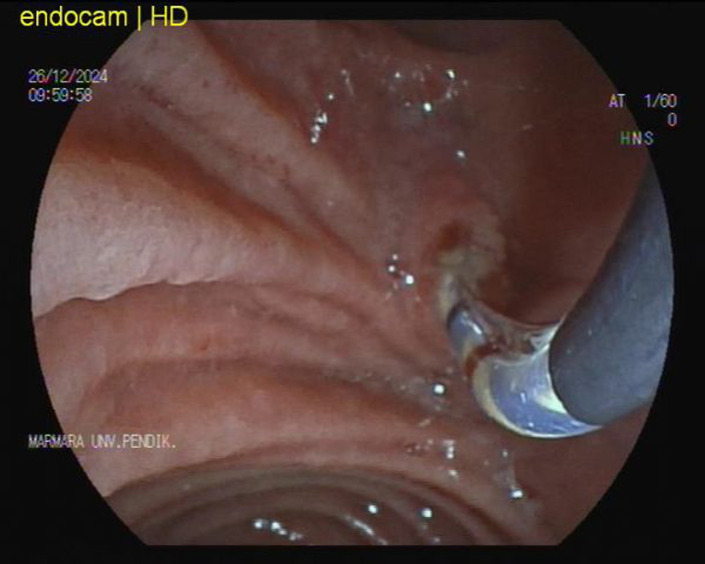


Figure 1. Biliary sphincterotomy in a patient with SIT.


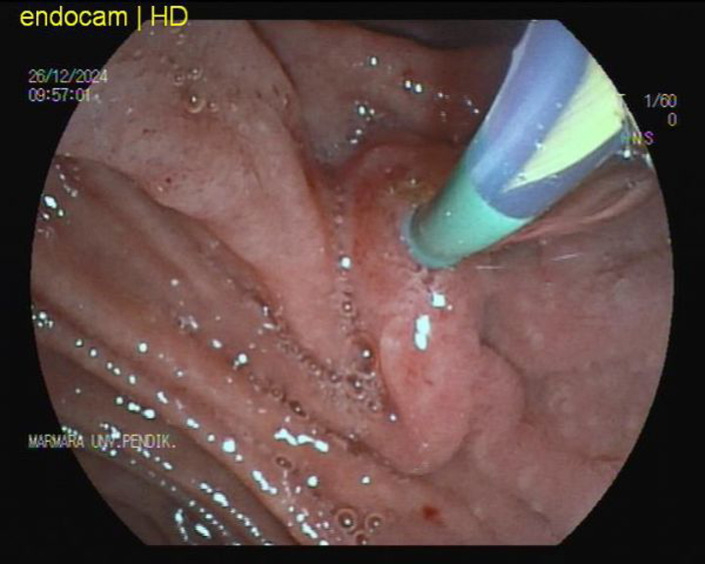


Figure 2. Papilla and biliary cannulation in a patient with SIT.

## PS-053 Inappropriate ADH Syndrome (SIADH): Paraneoplastic Syndrome Developing in Pancreatic Adenocarcinoma

**Kübra Ağca ^1^ , Yakub Patat ^1^ , Satı Karataş ^1^ , Volkan Sayğılı ^1^ , Gülten Can Sezgin ^1^ , Mevlüt Başkol ^1^ , Kadri Güven ^1^ , Ömer Özbakır ^1^ , Kemal Deniz ^2^ , Şebnem Gürsoy ^1^**

^1^Department of Gastroenterology, Erciyes University Faculty of Medicine, Kayseri, Türkiye

^2^Department of Pathology, Erciyes University Faculty of Medicine, Kayseri, Türkiye

SIADH is commonly linked to pulmonary diseases (10%), CNS disorders, medications, and infections. It presents with hyponatremia, low plasma osmolality, high urine osmolality, and increased urine sodium. Symptoms can range from asymptomatic to severe neurological signs. Its association with gastrointestinal malignancies is rare. This case reveals SIADH leading to a diagnosis of pancreatic adenocarcinoma. An 81-year-old man presented with pruritus and jaundice. His history included bladder carcinoma treated 8 years ago and interstitial lung disease (UIP pattern) for 1 year. The physical exam showed scleral and skin jaundice. Labs: AST 204 U/L, ALT 188 U/L, ALP 1286 U/L, GGT 829 U/L, bilirubin 19/16 mg/dL. On day 9 of hospitalization, resistant hyponatremia developed. High urine sodium and osmolality with euvolemia suggested SIADH. Imaging (USG, CT, MRCP) showed bile duct dilation, distal common bile duct narrowing, and a 30 × 30 mm pancreatic head mass. ERCP was not feasible due to pyloric deformity; external drainage was performed via PTC. A biopsy confirmed adenocarcinoma. Other SIADH causes were excluded, indicating a paraneoplastic syndrome. Tolvaptan normalized sodium levels.

SIADH is rare in pancreatic adenocarcinoma and may result from ectopic ADH secretion. Although uncommon in GI malignancies, especially outside P-NETs, SIADH should be considered in cancer patients with hyponatremia. In this case, SIADH was a key clue that guided the diagnostic process.

## PS-054 Pancreatic Head Mass Resolved with Steroid Therapy: A Case of IgG4-Related Pancreatitis

**Kübra Ağca ^1^ , Yakub Patat ^1^ , Satı Karataş ^1^ , Volkan Sayğılı ^1^ , Gülten Can Sezgin ^1^ , Mevlüt Başkol ^1^ , Kadri Güven ^1^ , Ömer Özbakır ^1^ , Kemal Deniz ^2^ , Şebnem Gürsoy ^1^**

^1^Department of Gastroenterology, Erciyes University Faculty of Medicine, Kayseri, Türkiye

^2^Department of Pathology, Erciyes University Faculty of Medicine, Kayseri, Türkiye

IgG4-related diseases are immune-mediated conditions that cause inflammatory mass lesions in various organs. With growing awareness, their incidence has increased, and diagnostic criteria continue to evolve. IgG4-related pancreatitis can clinically and radiologically mimic pancreatic tumors. Accurate diagnosis and timely treatment often lead to resolution, making differential diagnosis essential. This case involves a patient initially suspected of having pancreatic cancer due to a pancreatic head mass, later diagnosed with IgG4-related pancreatitis, and successfully treated with steroids. A 51-year-old woman presented with left flank pain. She had a history of hypothyroidism treated with levothyroxine. Physical examination revealed jaundice and epigastric tenderness. Lab results: total/direct bilirubin: 8.76/8.37 mg/dL, GGT/ALP: 399/742 U/L, AST/ALT: 115/231 U/L, amylase/lipase: 227/973 IU/mL. Imaging (USG, CT, MRCP) showed a 3×3 cm mass in the pancreatic head encasing the SMV (~180°). The common bile duct was dilated (9 mm) with a blunt end, suggesting malignancy. Biopsy confirmed IgG4-related pancreatitis. Methylprednisolone 60 mg/day was started. After 1 month, liver function normalized and symptoms improved. Steroids were tapered. CT at 2 months showed complete resolution. Steroids were discontinued at 6 months, and the patient remained under follow-up. IgG4-related pancreatitis is a rare inflammatory disease that can resemble pancreatic cancer. It typically responds rapidly to steroids. Rituximab may be considered if steroids are ineffective. Even after remission, regular follow-up with labs and imaging is necessary. In case of recurrence, alternative therapies should be considered. This case highlights the importance of accurate diagnosis and demonstrates that complete remission is achievable with proper treatment.

## PS-055 The Relationship Between BMPR1A Mutation and Hereditary Polyposis Syndromes

**Kübra Ağca ^1^ , Yakub Patat ^1^ , Satı Karataş ^1^ , Volkan Sayğılı ^1^ , Gülten Can Sezgin ^1^ , Mevlüt Başkol ^1^ , Kadri Güven ^1^ , Ömer Özbakır ^1^ , Kemal Deniz ^2^ , Şebnem Gürsoy ^1^**

^1^Department of Gastroenterology, Erciyes University Faculty of Medicine, Kayseri, Türkiye

^2^Department of Pathology, Erciyes University Faculty of Medicine, Kayseri, Türkiye

Colonic polyps may develop either sporadically or as part of hereditary syndromes. juvenile polyposis syndrome (JPS) and hereditary mixed polyposis syndrome (HMPS) are rare conditions associated with a risk of malignant transformation. Several studies have shown that mutations in the BMPR1A gene are responsible for JPS and HMPS. Data suggest that inactivation of BMPR1A may initiate colorectal tumorigenesis. A case is presented in which multiple types of polyps were observed concurrently, and a BMPR1A mutation was identified through genetic analysis. A 28-year-old male patient with no known comorbidities presented with constipation, episodes of diarrhea, bloating, and rectal bleeding. Colonoscopy revealed numerous polyps of varying sizes in the rectum, sigmoid colon, and transverse colon. Polyps smaller than 4 mm were removed using forceps, whereas those between 4 and 10 mm were excised via hot snare polypectomy. No polyps larger than 10 mm were detected. Biopsies were taken from sessile polyps. Histopathological evaluation revealed juvenile, hyperplastic, and inflammatory polyps. Immunohistochemical analysis showed positive expression of MLH1, MSH2, MSH6, and PMS2, indicating microsatellite stability (MSS). Genetic testing confirmed a BMPR1A mutation, suggesting a diagnosis of JPS and/or HMPS. Patients with BMPR1A mutations are known to have increased risks of colorectal, gastric, pancreatic, thyroid, and prostate cancers. Therefore, screening for associated malignancies was performed. Given the autosomal dominant inheritance and the presence of precancerous lesions, genetic evaluation of family members was recommended. The coexistence of different types of polyps is rare and clinically significant due to their precancerous potential. In patients with multiple polyps, complete removal and combined pathological and genetic evaluation are essential. Upon detection of a BMPR1A mutation, close surveillance and family screening are required to reduce cancer risk, as recommended by international scientific societies. This case highlights the importance of a multidisciplinary approach and genetic assessment in complex polyp presentations.

## PS-056 Distribution of Achalasia Subtypes and Clinical Features by High-Resolution Manometry: A Single-Center Experience

**Halit Uğur, Taner Kara, Eren Düzgün, Emre Çöloğlu, Tolga Gözmen, Barış Cengiz, Tarık Turan, Mustafa Mustafayev, Güleycan Parça, Sevil Özer Sarı, Gözde Derviş Hakim**

Department of Gastroenterology, İzmir City Hospital, İzmir, Türkiye

**Background/Aims:** Achalasia is a primary motility disorder characterized by impaired deglutitive relaxation of the lower esophageal sphincter (LES) and loss of peristalsis. High-resolution manometry (HRM) is the diagnostic gold standard. The Chicago Classification v4.0 (CCv4.0) defines subtypes (I–III) based on abnormal median integrated relaxation pressure (IRP) in the supine/upright position along with associated peristaltic patterns. The study aimed to evaluate the demographic characteristics, referral indications, subtype distribution, and IRP profiles of patients diagnosed with achalasia by HRM.

**Materials and Methods:** A retrospective review of 227 patients referred to the Motility Laboratory of İzmir City Hospital, Department of Gastroenterology, between November 2024 and May 2025 was performed. Patients diagnosed with achalasia were classified according to CCv4.0. Gender, age, referral indications, IRP (mmHg), and subtype distribution were analyzed.

**Results:** Sixty-five patients were diagnosed with achalasia. Referral indications included dysphagia in 53 (81.5%), food sticking sensation in 7 (10.8%), and dyspepsia in 3 (4.6%). Thirty-six patients (55.4%) were male, and 29 (44.6%) were female. Subtype distribution was as follows: Type 2 in 56 (86.2%), Type 3 in 7 (10.8%), and Type 1 in 2 (3.1%). The mean IRP was 29.15 ± 13.74 mmHg (range, 3-62).

**Conclusion:** In this cohort, the mean IRP was ≈ 29 mmHg, with some patients exhibiting low IRP values. Previous studies also report “normal IRP achalasia,” suggesting that endoFLIP assessment may be helpful in such cases. The predominance of Type 2 (86%) is consistent with international cohorts. Achalasia should always be considered in patients presenting with dysphagia, and referral to motility laboratories facilitates timely diagnosis.

## PS-058 Challenging Cases in Crohn’s Disease: Abscess Management and Clinical Analysis

**Halit Uğur, Taner Kara, Eren Düzgün, Emre Çöloğlu, Tolga Gözmen, Barış Cengiz, Tarık Turan, Mustafa Mustafayev, Güleycan Parça, Sevil Özer Sarı, Gözde Derviş Hakim**

Department of Gastroenterology, İzmir City Hospital, İzmir, Türkiye

Crohn’s disease is a chronic inflammatory disorder that can affect any part of the gastrointestinal tract. Abscess formation is one of its major complications. This study presents the clinical management of 3 different patients with Crohn’s disease and abscesses, highlighting the importance of a multidisciplinary approach. Case 1 involved a 19-year-old man with a large abdominal abscess. Drainage under interventional radiology guidance and broad-spectrum antibiotics led to a successful outcome. Case 2 was a 49-year-old woman with Crohn’s disease who had undergone a prior right hemicolectomy. A large abscess extending from the left abdomen to the pleura was identified. During drainage, a rare complication—a bronchopleural fistula—occurred but was successfully managed by a multidisciplinary team. Case 3 involved an 18-year-old man with fistulizing Crohn’s disease who developed a mandibular abscess. Drainage was performed with ENT support, antibiotic therapy was initiated, and cultures grew Gram-positive cocci. Complete regression was observed on follow-up. Abscess management in Crohn’s disease is complex and requires a multidisciplinary approach. Radiological drainage combined with appropriate antibiotic therapy is often effective, whereas rare complications (e.g., bronchopleural fistula) should be managed with individualized strategies. Early diagnosis and multidisciplinary collaboration significantly improve patient outcomes.

## PS-059 Comparison of Gastroenterology and Interventional Radiology Approaches in Liver Biopsies: A Single-Center Retrospective Study

**Fatih Kıvrakoğlu ^1^ , Celal Yazıcı ^2^ , Mustafa Ergin ^3^**

^1^Department of Gastroenterology, Osmaniye State Hospital, Osmaniye, Türkiye

^2^Department of Radiology, Osmaniye State Hospital, Osmaniye, Türkiye

^3^Department of Gastroenterology, Aksaray Training and Research Hospital, Aksaray, Türkiye

**Background/Aims:** Liver biopsy remains a cornerstone in the diagnosis and staging of liver diseases, particularly chronic hepatitis B (CHB). This study aimed to compare the technical, histopathological, and clinical aspects of liver biopsies performed by gastroenterology and interventional radiology teams at the same center.

**Materials and Methods:** This retrospective, single-center study included 81 patients with CHB who underwent biopsy between February 1, 2022, and November 30, 2023, at Osmaniye State Hospital. The gastroenterology team performed biopsies using a 16G Hepafix needle via an intercostal approach after ultrasound marking, whereas the radiology team used an 18G automatic Geotek tru-cut needle via a subcostal approach under ultrasound guidance. Biopsy specimens were evaluated by pathologists using the Ishak scoring system.

**Results:** Thirty-three patients underwent biopsy by the gastroenterology team and 48 by the radiology team. The mean age was significantly higher in the radiology group (47.7 ± 13.2 vs. 42 ± 11.4 years; *P* = .046). ALT, INR, and HBV DNA levels were significantly higher in the gastroenterology group. No significant differences were found in fragmented biopsy rates, sample length, or diagnostic adequacy. However, the median number of portal tracts was greater in the gastroenterology group (13 vs. 10; *P* = .006). Fibrosis stage showed a borderline difference (*P* = .050). All patients in the gastroenterology group were hospitalized for 1 day of observation, whereas no hospitalizations were required in the radiology group (*P* < .001). No complications or mortality occurred in either group.

**Conclusion:** Both approaches provided diagnostically sufficient and safe biopsies. Gastroenterologists obtained samples with more portal tracts, whereas radiologists performed procedures on an outpatient basis with lower costs. These findings suggest that the choice of specialist may influence sample quality and healthcare costs, although overall diagnostic reliability and safety remain comparable.

## PS-060 Cholangiosepsis as the First Presentation of Crohn’s Disease: An Unusual Case Report

**Muhammed Furkan Keser ^1^ , Yahya Atayan ^1^ , Ayşe Hafsa Çağın ^2^ , Zeynep Büşra Keser ^3^ , Yüksel Seçkin ^1^ , Yasir Furkan Çağın ^1^**

^1^Department of Gastroenterology, İnönü University Faculty of Medicine, Malatya, Türkiye

^2^Department of Internal Medicine, Necmettin Erbakan University Faculty of Medicine, Konya, Türkiye

^3^Department of Infectious Diseases and Clinical Microbiology, İnönü University Faculty of Medicine, Malatya, Türkiye

Crohn’s disease is a chronic inflammatory disorder that can affect any segment of the gastrointestinal tract. The terminal ileum and colon are most commonly involved, whereas duodenal disease occurs in about 5% of patients. Hepatobiliary manifestations are uncommon, and cholangiosepsis as the first presentation of Crohn’s disease is exceptionally rare. A 29-year-old man with no prior medical history presented with fever, right upper quadrant pain, jaundice, fatigue, and hypotension. Laboratory tests showed marked leukocytosis, hyperbilirubinemia, increased transaminases, impaired renal function, hypoalbuminemia, and anemia. Imaging revealed intrahepatic bile duct dilatation, distal common bile duct narrowing, and a segment 6-7 cholangitic abscess. ERCP demonstrated an edematous, ulcerated, and hypertrophic ampulla, but the procedure cannot be completed. Therefore, percutaneous transhepatic cholangiography with external biliary drainage was performed. Ampullary biopsy showed features consistent with Crohn’s disease. Colonoscopy revealed terminal ileal ulcerations with crypt abscesses and crypt distortion. Drainage of the liver abscess yielded Klebsiella pneumoniae. All tuberculosis investigations were negative. The patient was hospitalized for 20 days. Although sepsis and the liver abscess were managed successfully, renal function did not recover, and he required chronic hemodialysis. A final diagnosis of Crohn’s disease was established, and infliximab therapy was planned. Duodenal and periampullary involvement in Crohn’s disease is rare but can lead to biliary obstruction and cholangiosepsis. Only a few cases have been reported: Stefanovic et al reported ampullary involvement in a patient with established Crohn’s disease, whereas Foutch reported a surgically treated case. To the author’s knowledge, this is the first report of cholangiosepsis as the initial presentation of Crohn’s disease. Cholangiosepsis may represent a rare but severe initial manifestation of Crohn’s disease. This unusual case expands the recognized clinical spectrum of Crohn’s disease.


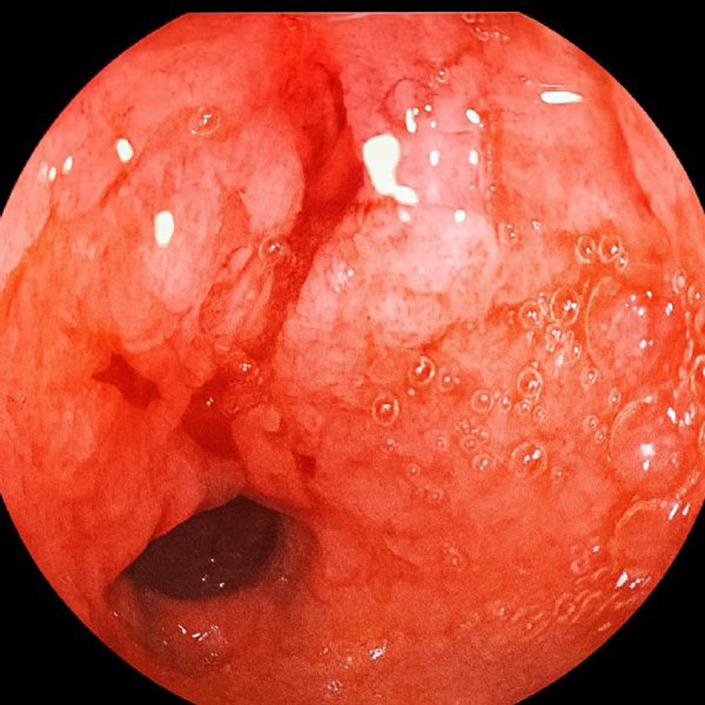


Figure 1. Endoscopic view showing marked edema, ulceration, and mucosal irregularity around the ampulla of Vater. Findings are consistent with rare duodenal and periampullary involvement of Crohn’s disease.


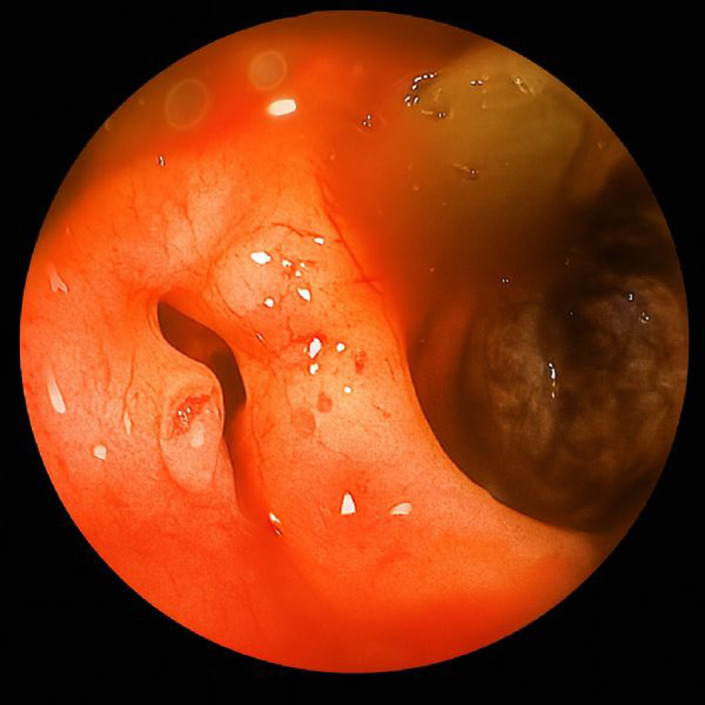


Figure 2. Endoscopic view of the terminal ileum showing marked ulceration, mucosal edema, and irregularity. Findings are consistent with the typical ileal manifestation of Crohn’s disease.


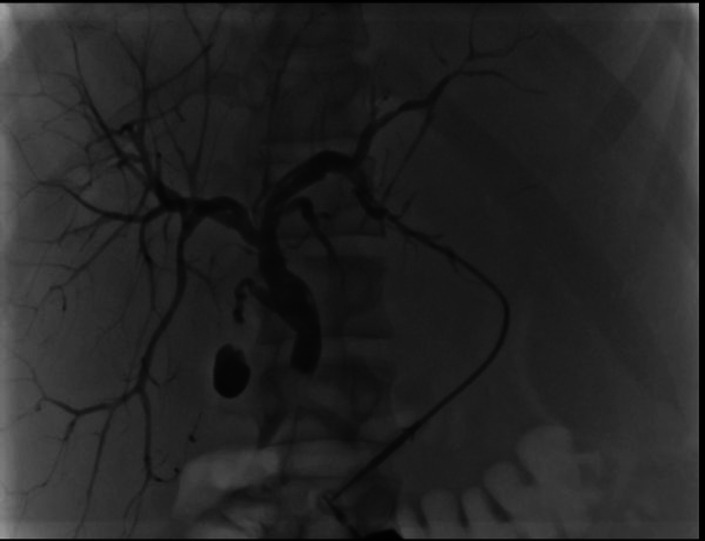


Figure 3. Cholangiographic view showing marked dilatation of the intrahepatic bile ducts with loss of passage at the distal common bile duct level. The percutaneous transhepatic drainage catheter is also visible.

## PS-063 Rare Cause of Mechanical Bowel Obstruction: Abdominal Cocoon Syndrome

**Ahmet Emre Ergan ^1^ , Osman Özdoğan ^1^ , Cumhur Özcan ^2^ , Feramuz Demir Apaydın ^3^ , Ümit Yeşilova ^1^ , Mammadhasan Mammadov ^1^ , Oktay Bayraktar ^1^ , Mehmet Kasım Aydın ^1^ , Serkan Yaraş ^1^ , Fehmi Ateş ^1^ , Engin Altıntaş ^1^ , Orhan Sezgin ^1^**

^1^Department of Gastroenterology, Mersin University Faculty of Medicine, Mersin, Türkiye

^2^Department of General Surgery, Mersin University Faculty of Medicine, Mersin, Türkiye

^3^Department of Radiology, Mersin University Faculty of Medicine, Mersin, Türkiye

Abdominal cocoon syndrome (ACS), also known as sclerosing encapsulated peritonitis, is a rare cause of bowel obstruction characterized by a fibrous membrane forming around the small intestines. Most cases are reported in women, and the exact cause remains unknown. Diagnosis is typically confirmed via laparoscopy. This case report details the diagnostic process of ACS in a young male patient with acute abdominal pain. A 23-year-old man presented to the emergency department with abdominal pain and vomiting. He had a surgical history of anal atresia at age 1. A physical exam revealed diffuse abdominal tenderness. He was initially admitted with a preliminary diagnosis of volvulus; however, imaging studies were crucial to the correct diagnosis. An abdominal ultrasound (USG) performed by gastroenterologists noted “a peritoneal enveloping structure around the small intestines, which were gathered in the stomach and left upper quadrant,” suggesting ACS. A subsequent abdominal CT scan showed a “whirlpool sign and gathering of jejunal loops with a thick, wall-like structure,” which supported the diagnosis of ACS. A multidisciplinary board confirmed the diagnosis, and surgical intervention was planned. The patient underwent a laparotomy, during which membrane resection and bridectomy were performed. His symptoms rapidly resolved, and he was discharged. This case highlights the importance of considering rare causes of acute abdominal pain in young patients. Despite the initial misdiagnosis, the detailed USG and CT findings guided the team to the correct diagnosis. It is plausible that the patient’s previous surgery for high-type anal atresia may have contributed to this condition, a link not yet reported in the literature. This case offers clinicians a unique perspective on the causes and diagnostic approach for this rare syndrome, suggesting a potential association between a congenital anomaly and ACS.


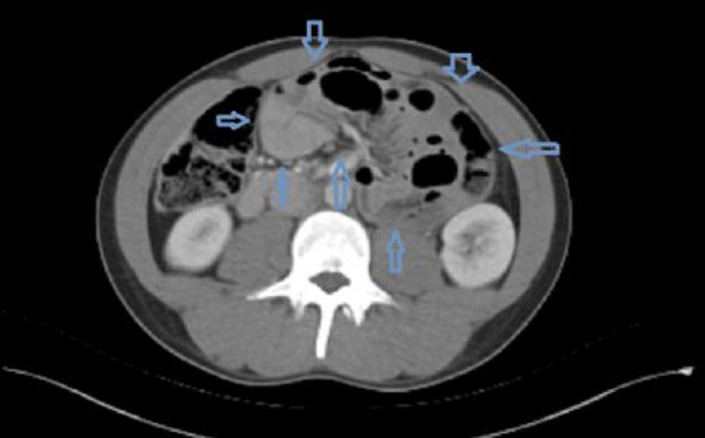


Figure 1. The patient’s abdominal CT scan taken before the surgical procedure.

## PS-064 Clinical, Serological, and Noninvasive Parameters Predicting Relapse in Autoimmune Hepatitis

**İlyas Ethem Şenocak, Yunus Emre Demiral, Yunus Günegül, Talha Ercan, Arda Bilgili, Ahmet Tarık Eminler**

Department of Gastroenterology, Sakarya University Training and Research Hospital, Sakarya, Türkiye

**Background/Aims:** Autoimmune hepatitis (AIH) is a chronic, immune-mediated liver disease characterized by fluctuating disease activity. Despite immunosuppressive therapy, relapse remains a frequent challenge and can lead to progressive liver damage if not identified early. Identifying reliable predictors of relapse is essential for optimizing long-term management. This study aimed to evaluate clinical, serological, and noninvasive scoring systems to determine their effectiveness in predicting relapse in patients with AIH.

**Materials and Methods:** A retrospective cohort study was conducted involving 68 patients diagnosed with AIH at a tertiary care center. The mean age was 53.9 ± 15.9 years, and 76.5% of the patients were female. Patients were followed for a median period of 36.5 months. Relapse was defined as an increase in alanine aminotransferase (ALT) or aspartate aminotransferase (AST) levels greater than twice the upper limit of normal (ULN) and/or an increase in serum immunoglobulin G (IgG) levels. Patients were categorized into 2 groups based on relapse status: those who experienced a relapse (n = 29) and those who did not (n = 39). Baseline laboratory parameters including ALT, AST, platelet count, IgG, FIB-4, and APRI scores were analyzed for predictive value.

**Results:** Significant differences were observed between the relapse and nonrelapse groups across all measured parameters (*P* < .05). Among these, APRI (*Z* = −4.371) and FIB-4 (*Z* = −4.030) demonstrated the strongest statistical association with relapse (*P* < .001). In terms of predictive accuracy, APRI had an area under the curve (AUC) of 0.812, whereas FIB-4 had an AUC of 0.787, indicating high diagnostic performance.

**Conclusion:** APRI and FIB-4 are effective, noninvasive tools for predicting relapse in patients with autoimmune hepatitis. Their routine use in follow-up assessments may support earlier intervention and improved disease monitoring.

Table 1.Baseline Demographic Findings According to Relapse Status

Values are expressed as mean ± SD or median (interquartile range), as appropriate.

APRI, AST-to-platelet Ratio Index; ALT, alanine aminotransferase; AST, aspartate aminotransferase; FIB-4, Fibrosis-4 index; IgG, immunoglobulin G.

**Table:** 

Variable	Total	Relapse (+) (n = 29)	Relapse (−) (n = 39)	*P*
Age (years)	53.9 ± 15.9	55.4 ± 16.9	52.8 ± 15.3	.362
ALT (U/L)	245.0 ± 332.3	314.6 ± 325.5	193.3 ± 331.9	.0038
AST (U/L)	225.7 ± 284.4	303.9 ± 311.1	167.6 ± 251.3	<.001
Platelet count (10^9^/L)	205.7 ± 81.5	172.8 ± 86.2	230.2 ± 69.2	.002
IgG (g/L)	23.18 ± 7.61	26.54 ± 8.76	20.68 ± 5.54	.0133
APRI	1.3 (0.8-5.0)	4.5 (1.5-9.1)	0.9 (0.6-1.5)	<.001
FIB-4	3.2 (1.7-5.1)	5.0 (3.0-9.2)	2.2 (1.2-3.4)	<.001

## PS-067 First Report of Fatal Candida Auris Liver Abscess in an Immunocompetent Patient

**Muhammed Furkan Keser ^1^ , Yahya Atayan ^1^ , Mustafa Duru ^1^ , Yüksel Seçkin ^1^ , Zeynep Büşra Keser ^2^ , Ayşe Hafsa Çağın ^3^**

^1^Department of Gastroenterology, İnönü University Faculty of Medicine, Malatya, Türkiye

^2^Department of Infectious Diseases and Clinical Microbiology, İnonu University Faculty of Medicine, Malatya, Türkiye

^3^Department of Internal Medicine, Necmettin Erbakan University Faculty of Medicine, Konya, Türkiye

Candida auris is an emerging fungal pathogen associated with multidrug resistance and high mortality. It is primarily reported in intensive care and immunosuppressed patients, whereas hepatobiliary involvement is rare. This case presents the first fatal Candida auris liver abscess in an immunocompetent patient. A 63-year-old immunocompetent man with a history of mitral valve surgery and diabetes mellitus presented with fever and right upper quadrant pain. Laboratory tests showed leukocytosis (28 310/mm^3^), procalcitonin 9.5 ng/mL, total bilirubin 5.46 mg/dL, and direct bilirubin 3.4 mg/dL. CT and MRI revealed multiple loculated collections with air densities in both liver lobes, along with intrahepatic bile duct dilatation and wall enhancement, consistent with cholangitis and cholangitic abscesses. ERCP demonstrated distal common bile duct obstruction with a fistula; a stent was placed, followed by abscess drainage. Initial cultures yielded Candida spp., whereas further testing confirmed multidrug-resistant Candida auris from urine and abscess specimens. Follow-up cultures later grew Pseudomonas aeruginosa. The patient received anidulafungin, fluconazole, and meropenem for about 2 months. Predischarge control cultures were negative, and he was discharged with the drainage catheter. Five days later, he was readmitted with increased acute-phase reactants. Imaging identified a new psoas abscess. Despite plans for drainage, the patient died from multiple organ failure before the procedure can be completed. Candida auris is most often isolated from blood, urine, and respiratory tract samples. Hepatobiliary involvement is exceedingly rare; previous reports include a liver abscess in a transplant recipient, another in chronic liver disease, and a case of acute gangrenous cholecystitis, all in immunosuppressed hosts. This case is the first report of a fatal Candida auris liver abscess in an immunocompetent patient. Recurrence despite antifungal therapy highlights the pathogen’s resistance and management challenges. Accurate species identification, early diagnosis, and timely antifungal treatment remain essential.


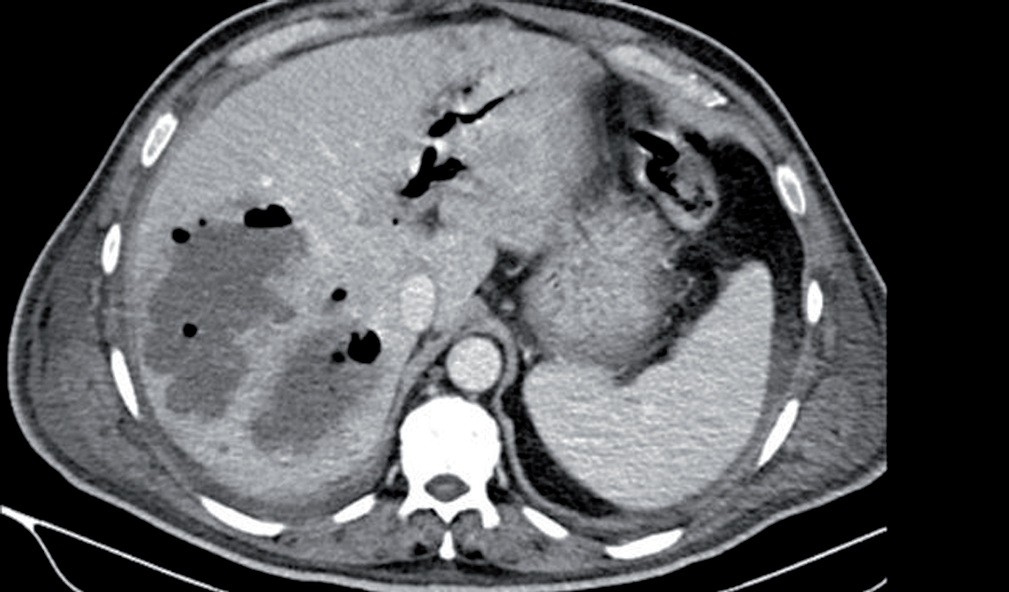


Figure 1. Contrast-enhanced abdominal CT demonstrates multiple lobulated hypodense collections containing air densities in both liver lobes, consistent with cholangitic abscesses.

## PS-068 Retrospective Examination of the Etiological Distribution of Cirrhosis Disease

**Hürü Kerse Yeşil ^1^ , Ali Can Erdem ^2^ , Murat Bıyık ^2^ , Mehmet Asıl ^2^ , Muharrem Keskin ^2^ , Ramazan Dertli ^2^ , Ali Demir ^2^**

^1^Ministry of Health Meram State Hospital, Konya, Türkiye

^2^Necmettin Erbakan University Faculty of Medicine, Konya, Türkiye

**Background/Aims:** This study aimed to retrospectively investigate the etiological distribution of patients diagnosed with liver cirrhosis who were admitted to the gastroenterology outpatient clinic of Necmettin Erbakan University Meram Faculty of Medicine Hospital between 2016 and 2021.

**Materials and Methods:** A total of 792 patients with liver cirrhosis, identified by ICD diagnostic codes in the hospital electronic system between January 1, 2016, and January 1, 2021, were included. Etiological factors, endoscopic findings, laboratory parameters (biochemistry and hemostasis), abdominal ultrasonography, and clinical evaluations were retrospectively analyzed.

**Results:** The mean age of the patients was 63.9 ± 13.1 years, and 54.2% were male. The most common etiologies were cryptogenic cirrhosis (36.8%), HBV (29.2%), autoimmune (10.9%), HCV (8.1%), alcohol (4.1%), NASH (3.9%), and cardiac causes (1.9%). According to the Child-Pugh classification, 40.1% were Child A, 35.5% Child B, and 15.3% Child C. Among complications, esophageal varices were detected in 79% (23.4% stage 1, 24.7% stage 2, 17% stage 3), congestive gastropathy in 39.2%, and splenomegaly in 60.3%.

**Conclusion:** These findings indicate that HBV and cryptogenic cirrhosis (often associated with obesity and diabetes) remain the leading etiologies in the region. Alcohol-related cirrhosis was found to be significantly lower compared to national data and reports from Western countries.


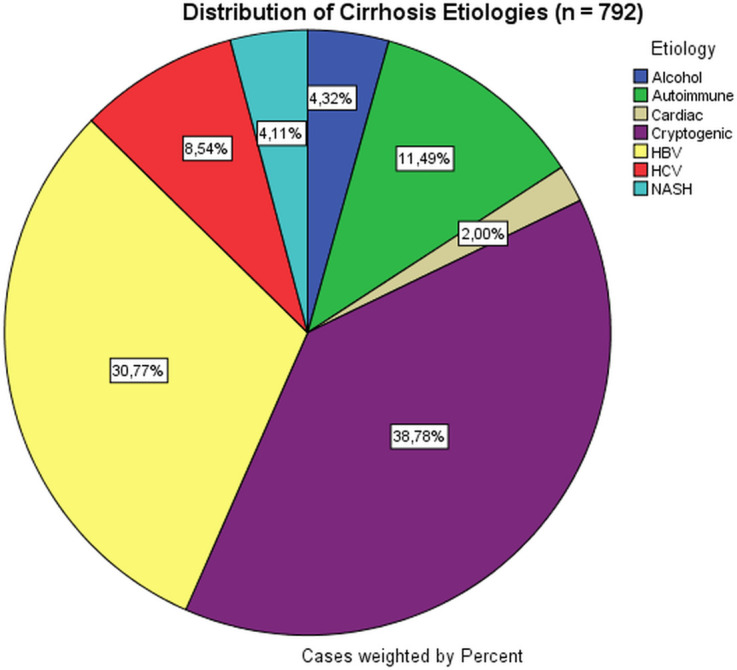


Figure 1. Distribution of cirrhosis etiologies.

## PS-069 Effect of Entecavir Used in Chronic Hepatitis B Treatment on Platelets

**Aysun Yakut**

Department of Gastroenterology, İstanbul Medipol University Sefaköy Health Practice Research Center, İstanbul, Türkiye

**Background/Aims:** P2X7 receptor (P2X7R) activation is thought to contribute to the formation of platelet (PLT) thrombosis. Various publications have shown that the P2X7R antagonist entecavir (ENT), used in the treatment of chronic hepatitis B (CHB), interacts strongly with human P2X7R (hP2X7R) and inhibits platelet thrombosis. This study aimed to show the changes in the number of PLTs in patients diagnosed with CHB who received medical treatment compared to those who did not.

**Materials and Methods:** This is a retrospective study in which the PLT values of 241 patients diagnosed with noncirrhotic, delta agent-free CHB between June 2022 and January 2024 were evaluated based on whether they received antiviral treatment. During the follow-up visits of the patients, their anamnesis, medical treatment status (entecavir 0.5 mg/day (ENT), tenofovir disoproxil fumarate 245 mg/day (TDF), or no medical treatment), laboratory results, and ultrasound information were recorded from the hospital automation system. Additionally, the FIB-4 scores of these patients were calculated and recorded cross-sectionally at the time the blood samples were taken.

**Results: **The research included a total of 241 patients, 57.3% (n = 138) of whom were male and 42.7% (n = 103) female, who presented to the gastroenterology outpatient clinic between June 2022 and January 2024. The ages of the patients ranged from 18 to 80, with an average of 46.1±14.2. A statistically significant difference was found between the PLT measurement values of the cases according to the drug groups (*P* = .001). PLT measurement values in the ENT group were found to be significantly lower compared to those of patients who did not use medication and those who used TDF (*P* = .001; *P* = .001; *P* = .01).

**Conclusion:** Although studies have shown that ENT does not have a significant effect on prothrombin time, activated partial thrombin time, thrombin time, fibrinogen, mean PLT volume, and PLT numbers, this study demonstrated a significant partial decrease in PLT.

## PS-070 Fulminant Liver Failure Following Biliary Leakage After Laparoscopic Cholecystectomy

**Şafak Meriç Özgenel**

Department of Gastroenterology, Osmangazi University Faculty of Medicine, Eskişehir, Türkiye

A 54-year-old woman underwent laparoscopic cholecystectomy in a state hospital in June 2025 for cholelithiasis. Postoperatively, after the intra-abdominal drain was removed, the patient began complaining of abdominal distension and discharge from the drain site due to intra-abdominal fluid. The drain was removed by the operating surgeon, and the patient was referred to a gastroenterologist, who diagnosed her with a cirrhotic liver during intraoperative liver evaluation. A postoperative MR cholangiopancreatography dated August 7, 2025, revealed a 39 × 22-mm heterogeneous hyperintense signal area in liver segment 6. No dilatation or filling defect was detected in the common bile duct, intrahepatic bile ducts, or extrahepatic bile ducts. No dilatation was detected in the pancreatic duct. Extensive free fluid was observed in the abdomen. An abdominal USG dated August 13, 2025, revealed diffuse fluid around the liver and spleen. The liver contours and size were normal. A diffuse increase in parenchymal echogenicity consistent with moderate steatosis was observed. No dilatation was detected in the intrahepatic bile ducts or common bile duct. The patient was referred to a tertiary healthcare unit for percutaneous drainage. Fluid examination revealed total bilirubin: 8.97 mg/dL, direct bilirubin: 5.77 mg/dL, albumin: 0.91 g/dL (serum albumin: 2.02 g/dL), leukocytes: 385/uL. Culture revealed no pathogen growth. A pelvic drainage catheter was placed via the right paracolic area, and approximately 2000 cm^3^ of dark yellowish bile-compatible content was drained. In addition to drainage, the patient received intravenous broad-spectrum antibiotics. No clinical or laboratory response was observed. The patient developed coagulopathy, hepatic encephalopathy, and septic shock. Inotropic therapy was initiated, and the patient was diagnosed with fulminant liver failure, leading to a decision for a liver transplant. The patient underwent a right lobe living donor liver transplant. She was transferred to the intensive care unit under high-dose inotropic support and intubated. The patient died on the first postoperative day. Due to the patient’s clinical evaluation being inconsistent, a biliary leak was overlooked, and she ultimately died. Close postoperative follow-up is crucial. The possibility of a biliary leak should have been considered, and appropriate action should have been taken.


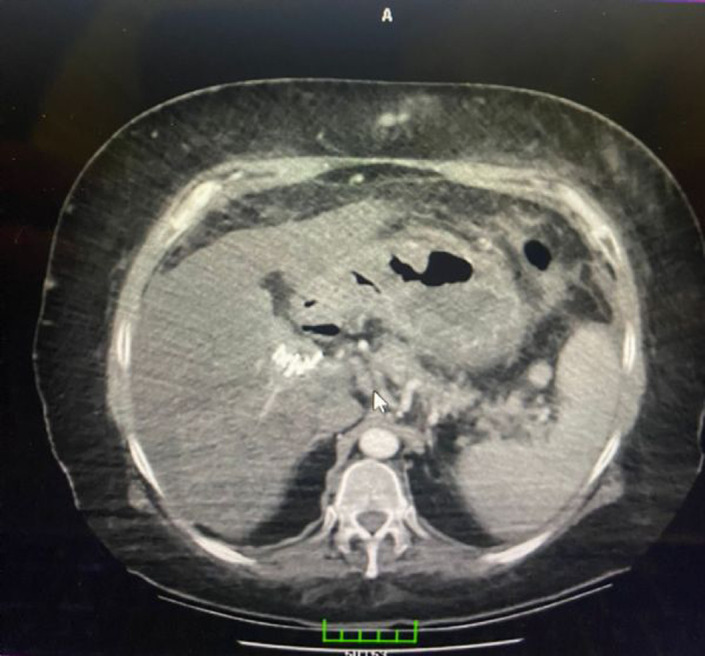


Figure 1. Computed tomography axial plane.


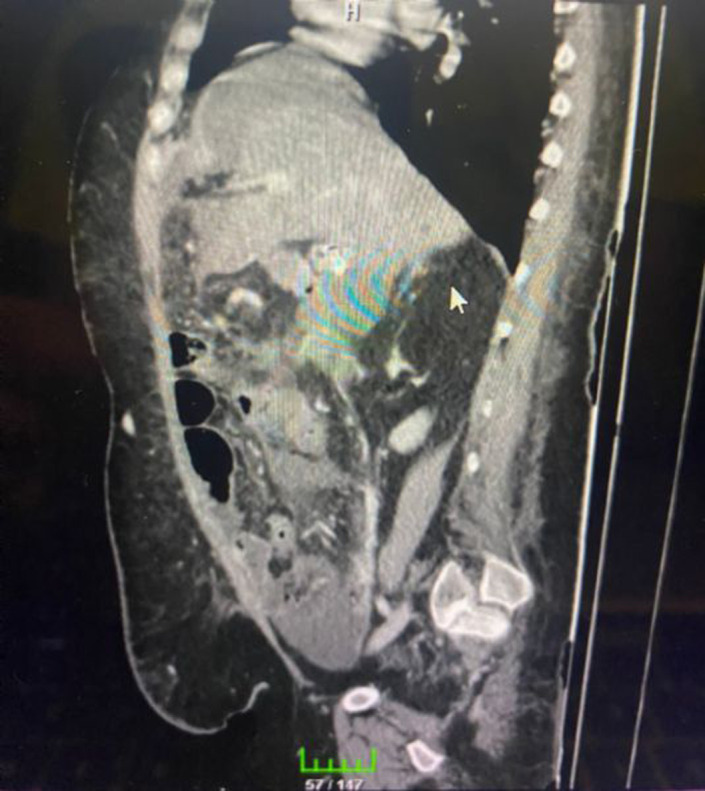


Figure 2. Computed tomography coronal plane


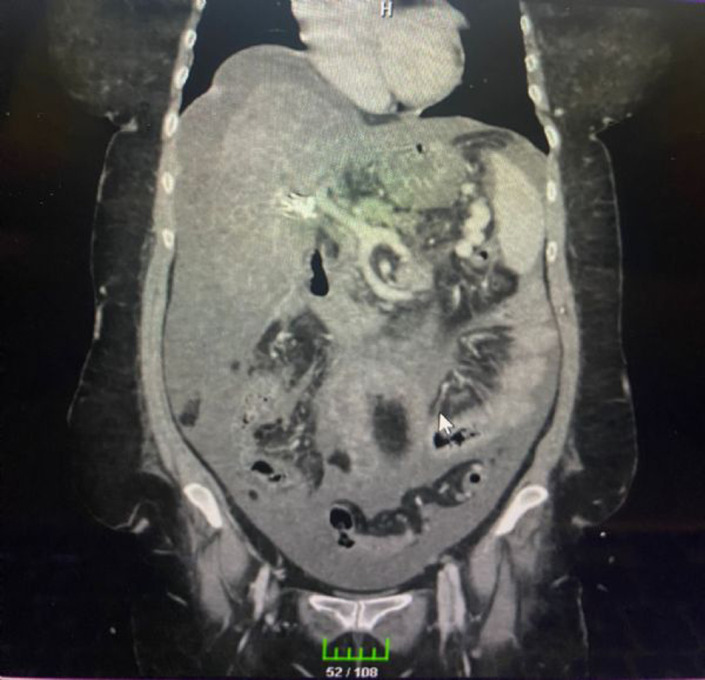


Figure 3. Computed tomography sagittal plane.

## PS-071 Obscure Gastric Outlet Obstruction Revealed by Endoscopy: Migration of a Metallic Biliary Stent

**Gökhan Köker ^1^ , Merve Eren Durmuş ^2^ , Serkan Öcal ^2^**

^1^Department of Internal Medicine, University of Health Sciences, Antalya Training and Research Hospital, Antalya, Türkiye

^2^Department of Gastroenterology, University of Health Sciences, Antalya Training and Research Hospital, Antalya, Türkiye

Gastric outlet obstruction (GOO) is a syndrome caused by impaired gastric emptying due to blockage of the distal stomach, pylorus, or proximal duodenum. It presents with nausea, vomiting, and distention. In biliary malignancies, palliative stent placement is common, but complications such as migration, occlusion, and infection may occur, often leading to diagnostic difficulties. A 58-year-old man presented with persistent vomiting. He had a history of cholangiocarcinoma, but medical records were unavailable. Initial endoscopy was inconclusive due to retained contents. A repeat endoscopy after prolonged fasting again revealed gastric retention. Following aspiration, a metallic biliary stent was visualized obstructing the pylorus. This case highlights the need to consider delayed stent-related complications in patients with biliary malignancies who exhibit GOO symptoms. Persistent gastric contents despite fasting should suggest mechanical obstruction. Stent migration, though rare, is a clinically significant event reported in the literature. Endoscopy remains essential, providing both diagnosis and therapeutic intervention. With the increasing use of stents, awareness of rare but important complications is vital. In patients with GOO, stent-related events must be suspected, as early recognition and management can improve outcomes.


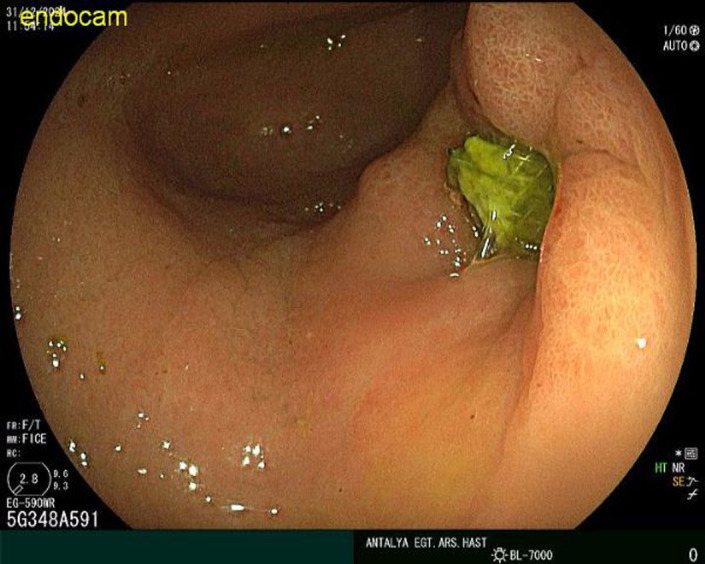


Figure 1. Endoscopic image of the migrated metallic stent obstructing the pylorus; its lumen was filled with gastric content.

## PS-072 Evidence of a Biliary Intruder: Endoscopic Removal of Fasciola Hepatica

**Gökhan Köker ^1^ , Merve Eren Durmuş ^2^ , Serkan Öcal ^2^**

^1^Department of Internal Medicine, University of Health Sciences, Antalya Training and Research Hospital, Antalya, Türkiye

^2^Department of Gastroenterology, University of Health Sciences, Antalya Training and Research Hospital, Antalya, Türkiye

Fasciola hepatica is a hepatobiliary parasite endemic in various regions worldwide, transmitted through the ingestion of contaminated aquatic vegetation or water. In its chronic phase, it can mimic common biliary pathologies such as choledocholithiasis or cholangiocarcinoma, leading to diagnostic challenges. The case of a 28-year-old woman is reported, who presented with abdominal pain, vomiting, and progressive jaundice. Laboratory tests revealed marked eosinophilia and increased cholestatic liver enzymes. Ultrasonography showed gallstones, whereas magnetic resonance cholangiopancreatography (MRCP) demonstrated a filling defect in the distal common bile duct (CBD). Endoscopic retrograde cholangiopancreatography (ERCP) revealed a motile, irregularly shaped intraluminal filling defect adherent to the CBD wall. During balloon extraction, a live adult Fasciola hepatica worm was removed. Antiparasitic therapy was initiated, and the patient was discharged in good condition. This case highlights the importance of considering parasitic infections in the differential diagnosis of biliary obstruction, especially in patients from endemic areas or with eosinophilia. ERCP plays a critical role in both diagnosis and treatment. The accompanying video captures the endoscopic extraction of the live fluke and may serve as an educational visual reference for clinicians.


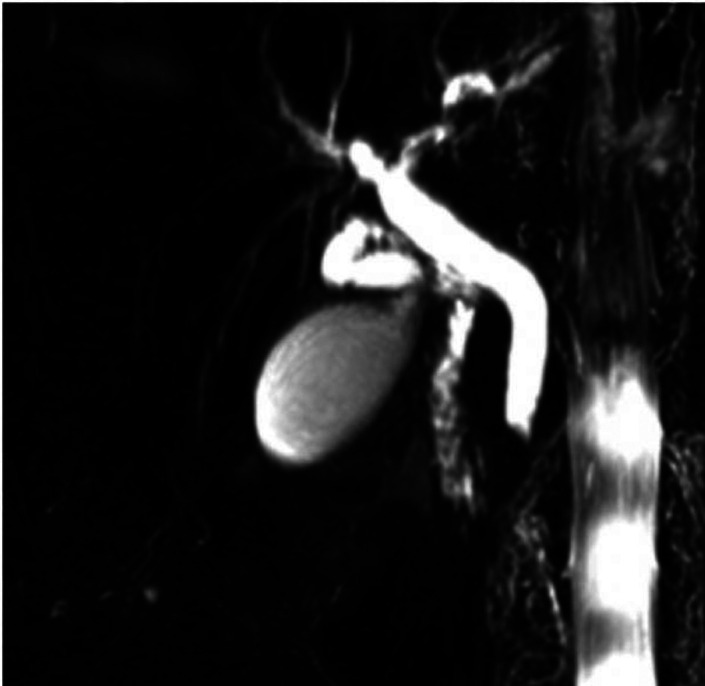


Figure 1. MRCP showing a distal common bile duct filling defect caused by *Fasciola hepatica*.


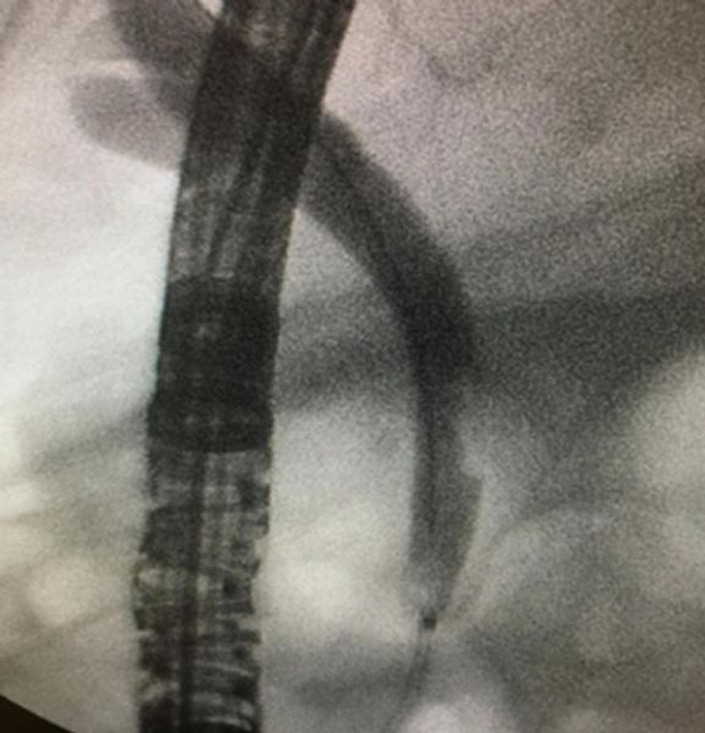


Figure 2. ERCP showing a distal common bile duct filling defect caused by *Fasciola hepatica*.


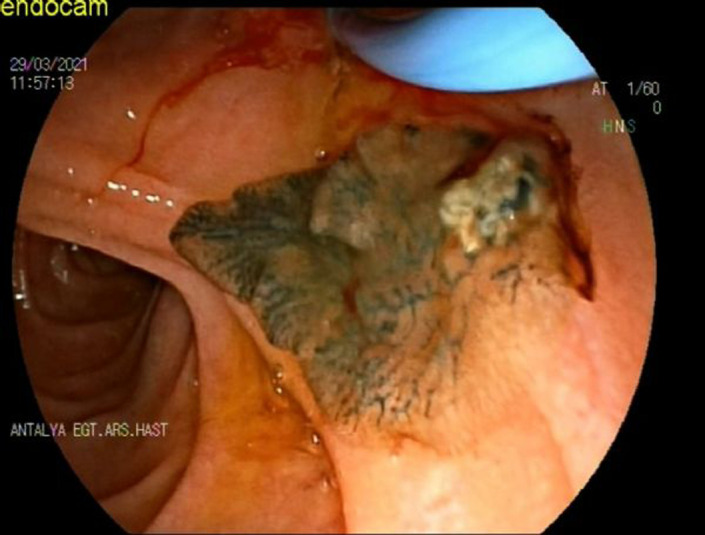


Figure 3. Endoscopic view of a live *Fasciola hepatica* removed from the common bile duct.

## PS-073 A Curious Case of Gastrointestinal Bleeding: Gastric Impostor

**Gökhan Köker ^1^ , Merve Eren Durmuş ^2^ , Kemal Eyvaz ^3^ , Arif Aslaner ^3^ , Serkan Öcal ^2^**

^1^Department of Internal Medicine, University of Health Sciences, Antalya Training and Research Hospital, Antalya, Türkiye

^2^Department of Gastroenterology, University of Health Sciences, Antalya Training and Research Hospital, Antalya, Türkiye

^3^Department of General Surgery, University of Health Sciences Antalya Training and Research Hospital, Antalya, Türkiye

Upper gastrointestinal bleeding and obstruction are commonly attributed to peptic ulcer disease and malignancies. Gastric lesions are less frequent but present unique diagnostic and therapeutic challenges, particularly when their location and morphology lead to misleading presentations. A rare case of a 77-year-old woman who presented with upper GI bleeding and obstructive symptoms is reported. Initial endoscopy revealed a bleeding mass in the second part of the duodenum, suggestive of a primary duodenal lesion. However, further evaluation with repeat endoscopy and abdominal CT revealed a large pedunculated tumor originating from the lesser curvature of the stomach that had prolapsed through the pylorus into the duodenum. The lesion was temporarily repositioned endoscopically to prevent gastric outlet obstruction. Due to its size and mobility, endoscopic resection was not feasible. The patient underwent laparoscopic surgery, which was converted to open resection. Pathology confirmed a low-grade gastrointestinal stromal tumor (GIST) with negative margins. Pedunculated gastric GISTs can mimic lesions in adjacent segments, leading to diagnostic confusion. This case emphasizes the importance of thorough imaging and endoscopic re-evaluation in atypical GI presentations and highlights the critical role of multidisciplinary management in complex upper GI tumors.


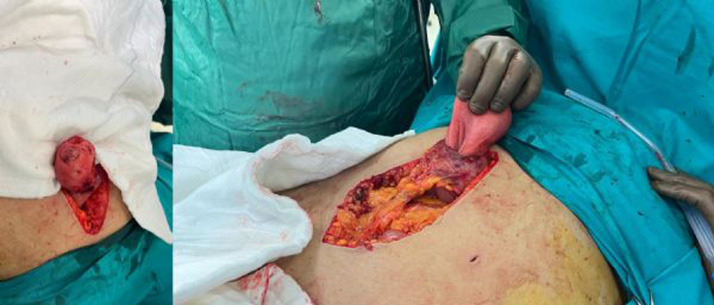


Figure 1. Surgical resection material.


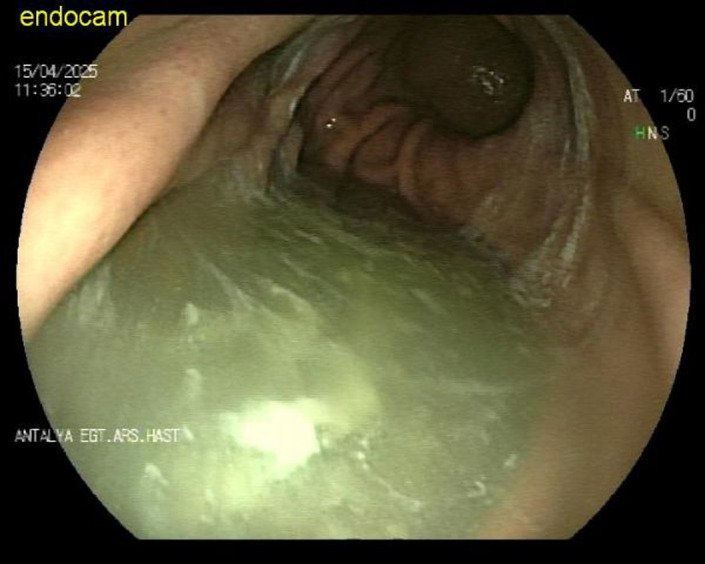


Figure 2. Endoscopic image from the first endoscopy.


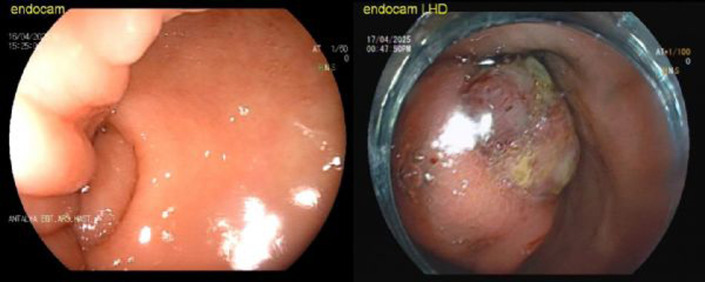


Figure 3. Images from the second session of endoscopy, showing the lesion’s stalk and where it was pulled back to the antrum.

## PS-074 Coil Migration: A Rare Cause of Cholangitis

**Mehmet Şerif Aktaş ^1^ , Merve Eren Durmuş ^1^ , Galip Egemen Atar ^1^ , Gökhan Köker ^2^ , Serkan Öcal ^1^ , Serdar Akça ^1^**

^1^Department of Gastroenterology, University of Health and Science, Antalya Training and Research Hospital, Antalya, Türkiye

^2^Department of Internal Medicine, University of Health and Science, Antalya Training and Research Hospital, Antalya, Türkiye

Cholangitis is an infection of the bile ducts, usually resulting from obstruction of bile flow. Common causes include choledocholithiasis, malignancies, and biliary strictures. A rare cause is coil migration following embolization procedures. Migrated coils can obstruct the bile ducts and lead to secondary infection. Only a few such cases have been reported in the literature. An 85-year-old woman presented with fever, right upper quadrant pain, and nausea. Her medical history included a cholecystectomy 20 years ago, during which coil embolization was performed due to a bile leak, and biliary stent placement 6 months ago for a benign stricture. Laboratory tests revealed signs of cholestasis and infection. MRCP showed common bile duct dilation and T2 hypointense filling defects. During ERCP, the biliary stents were removed. A distal stricture and foreign material (coil and sludge) were observed in the bile duct. The coil and debris were successfully removed with a balloon catheter, and the stricture was dilated using a CRE balloon. The procedure was completed without complications. Coil migration is a rare but potentially serious complication that may occur even decades after the initial embolization. When coils migrate into the biliary system, they can cause obstruction and cholangitis. In such cases, ERCP is a highly effective and minimally invasive method for both diagnosis and treatment. Awareness of this condition is crucial for early recognition and management, especially in patients with a history of prior coil embolization who present with signs of cholangitis. This case contributes to the limited number of reports on this rare clinical entity.


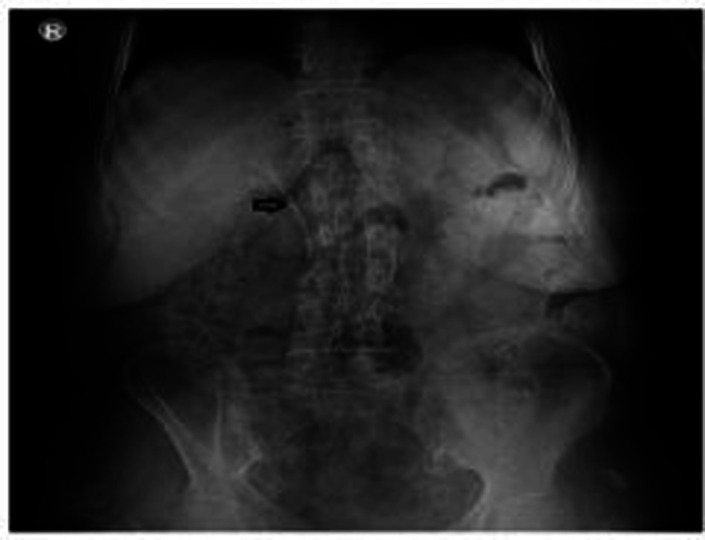


Figure 1. The biliary stents indicated by the black arrow.


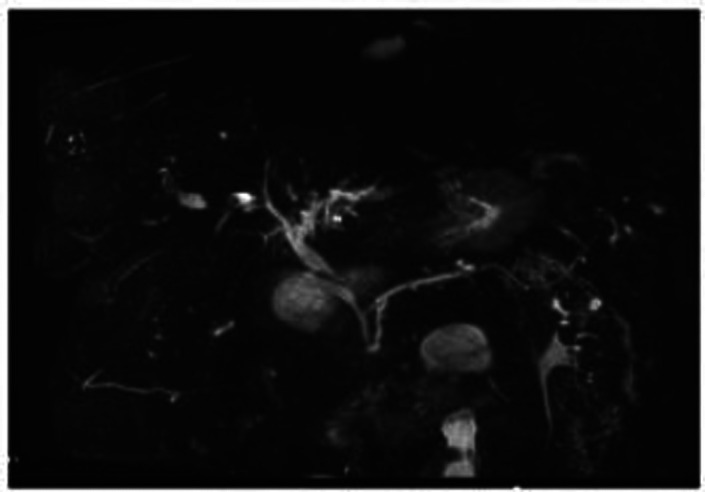


Figure 2. Hypointense filling defects, which completely fill the lumen in places, are noted within the common bile duct lumen.


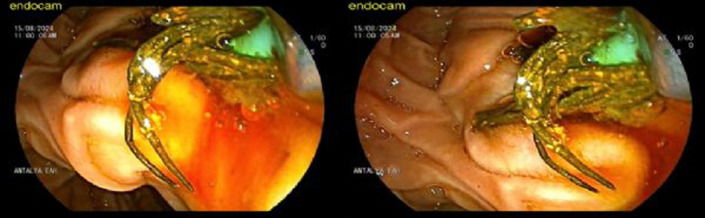


Figure 3. Endoscopic view of coils and sludge removed with a balloon.

## PS-075 Collagenous Sprue: A Rare Malabsorption Syndrome

**Satı Karataş ^1^ , Yakub Patat ^2^ , Şebnem Gürsoy ^2^ , Kemal Deniz ^3^**

^1^Kayseri City Hospital, Kayseri, Türkiye

^2^Department of Gastroenterology, Erciyes University Faculty of Medicine, Kayseri, Türkiye

^3^Department of Pathology, Erciyes University Faculty of Medicine, Kayseri, Türkiye

Collagenous sprue (CS) is a malabsorption syndrome that causes chronic diarrhea, weight loss, electrolyte imbalance, and nutritional deficiency. EGD shows combing and nodularity of the villi; duodenal biopsies show villous atrophy, and subepithelial collagen bands are present. A subepithelial collagen band thicker than 10 µm supports the diagnosis according to histopathological assessment, the gold standard. Autoimmune, drug-induced, and celiac disease are differential diagnoses. There is no conventional treatment, but gluten-free diets, immunosuppressive drugs, and corticosteroids are utilized. This case presentation reports the presentation, diagnosis, and treatment of CS. A 63-year-old male patient with no chronic disease or medication history presented to the Erciyes University Gastroenterology Outpatient Clinic with 2 years of abdominal pain, watery, bloodless diarrhea 6-7 times a day, nausea, vomiting, loss of appetite, and a 30 kg weight loss over the past year. Laboratory testing showed low magnesium, sodium, potassium, albumin, and folic acid levels. The stool tests were normal, and anti-TG and anti-EMA IgG were negative. Colonoscopy was normal. Upper endoscopy showed duodenal villi combing and nodularity. CS was diagnosed from duodenal samples with villous atrophy and subepithelial collagen bands. Initial treatment included a gluten-free diet and 32 mg/day methylprednisolone. Eight weeks were spent lowering the dose to 8 mg/day. The methylprednisolone dose was reduced to 4 mg/day when diarrhea subsided and oral intake and electrolyte imbalance improved. After 6 months, the patient gained weight and stopped having diarrhea. Follow-up upper endoscopy demonstrated decreased villous combing and collagen layer thinning in duodenal biopsies. CS might be misdiagnosed as celiac disease due to its similar clinical appearance. Malnutrition worsens the condition, which can be deadly; thus, early detection through microscopic and macroscopic evaluation is crucial. Early consideration of CS is warranted in gluten-free-resistant patients with chronic diarrhea, malabsorption, and electrolyte imbalance. A definitive diagnosis requires endoscopy, and histological evaluation should guide treatment.


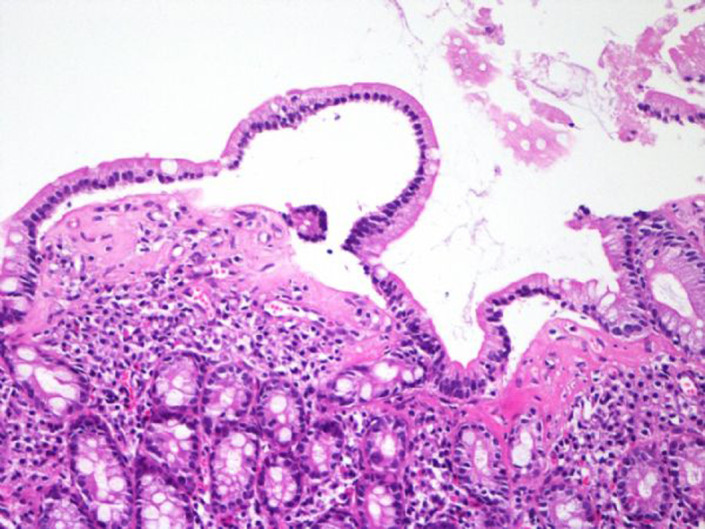


Figure 1. Pathological appearance of a patient with collagenous sprue before treatment.

## PS-077 A Case of Hepatitis B Reactivation Following Ribociclib Therapy in a Patient with Metastatic Breast Cancer

**Yunus Emre Demiral, İlyas Ethem Şenocak, Yunus Günegün, Talha Ercan, Arda Bilgili, Ahmet Tarık Eminler**

Department of Gastroenterology, Sakarya University Faculty of Medicine, Sakarya, Türkiye

Hepatitis B virus (HBV) reactivation is a recognized risk during immunosuppressive therapy and may lead to severe liver injury, therapy interruption, and increased mortality. Although CDK4/6 inhibitors, including ribociclib, are widely used in the treatment of hormone receptor-positive metastatic breast cancer, they are not traditionally considered high-risk for HBV reactivation. The first documented case of HBV reactivation is reported in a 61-year-old woman with metastatic breast cancer receiving ribociclib. The patient was anti-HBc IgG positive, with borderline HBsAg positivity and low HBV DNA prior to therapy. She received prophylactic tenofovir disoproxil fumarate (TDF) in accordance with international guidelines. Ribociclib was initiated, resulting in a favorable oncologic response. However, the patient discontinued TDF without medical consultation. Three months later, she developed biochemical hepatitis with increased ALT, AST, and bilirubin levels. HBV DNA increased to 10^7^ copies/mL, confirming HBV reactivation. TDF was re-initiated, leading to rapid biochemical improvement. This case highlights the potential for HBV reactivation during CDK4/6 inhibitor therapy in patients who prematurely discontinue antiviral prophylaxis. It highlights the importance of guideline-based management and strict adherence to prophylactic antiviral therapy, even with agents not classified as high-risk for HBV reactivation.


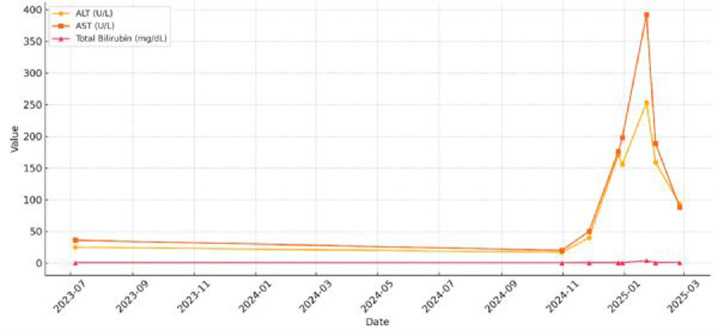


Figure 1. Alanine aminotransferase (ALT), aspartate aminotransferase (AST), and total bilirubin levels during the course of treatment with ribociclib and tenofovir disoproxil fumarate.

## PS-078 Clinical Characteristics of IBD Patients Transferred from Pediatric to Adult Gastroenterology: A Single-Center Experience

**Emre Çölüoğlu ^1^ , Eren Düzgün ^1^ , Taner Kara ^1^ , Tolga Gözmen ^1^ , Halit Uğur ^1^ , Mehmet Barış Cenğiz ^1^ , Tarık Turan ^1^ , Güleycan Parça ^1^ , Mustafa Mustafayev ^1^ , Sevil Özer Sarı ^1^ , Gözde Derviş Hakim ^1^ , Betül Aksoy ^2^ , Yeliz Çağan Appak ^2^ , Maşallah Baran ^2^**

^1^Department of Gastroenterology, İzmir City Hospital, İzmir, Türkiye

^2^Department of Pediatric Gastroenterology, İzmir City Hospital, İzmir, Türkiye

**Background/Aims:** Ulcerative colitis (UC) and Crohn’s disease (CD) affect approximately 1% of the population. In pediatric patients diagnosed with IBD, a transition process to adult gastroenterology care is necessary. In this study, the demographic and clinical characteristics of patients with IBD transferred from the pediatric gastroenterology clinic to the adult gastroenterology outpatient clinic were evaluated. The aim of this study was to investigate the demographic and clinical characteristics of patients with IBD transferring from pediatric gastroenterology to adult gastroenterology care.

**Materials and Methods:** Between October 2023 and August 2025, 22 patients who were transferred from the pediatric gastroenterology clinic to the adult gastroenterology clinic at İzmir City Hospital were included in the study. Data were retrieved from the hospital information system. Recorded parameters included sex, age, age at transfer, diagnosis (CD/UC), disease activity at transfer (remission/active), family history of IBD, and medications used at the time of transfer.

**Results:** A total of 22 patients were included. Eleven patients were male and 11 were female; 13 patients had UC and 9 had CD. The mean age at transfer was 18.4 years. At the time of transfer, 9 patients were in remission, whereas 13 patients had active disease. Among the 13 patients with active disease, 5 had CD and 8 had UC. Of the 5 patients with active CD, 2 were male and 3 were female; of the 8 patients with active UC, 5 were male and 3 were female. No family history of IBD was reported in 17 patients, whereas 5 patients had a positive family history. At transfer, 2 patients were on 5-ASA monotherapy, 10 on 5-ASA plus azathioprine, 3 on 5-ASA plus azathioprine and adalimumab, 4 on 5-ASA plus azathioprine and infliximab, 1 on adalimumab monotherapy, and 2 on azathioprine plus adalimumab.

**Conclusion:** In this study, pediatric-onset IBD patients were nearly equally distributed between UC and CD. More than half of the patients were transferred with active disease. In conclusion, a multidisciplinary and well-planned transition process between pediatric and adult gastroenterology clinics is critical for the optimal management of patients with IBD.


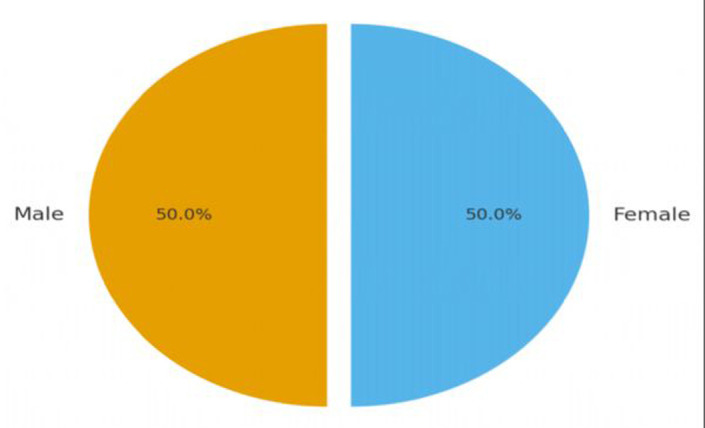


Figure 1. Gender distribution.


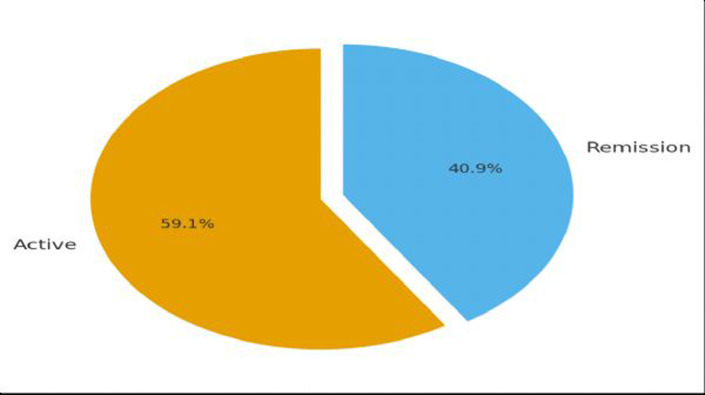


Figure 2. Clinical status at transfer.

## PS-079 Demographic Characteristics of Patients Diagnosed with Primary Biliary Cholangitis and Their Relationship with Other Autoimmune Diseases

**Berfin Seçil Gül, Fatma Yılmaz Öncül, Muhsin Kaya**

Department of Gastroenterology, Dicle University Faculty of Medicine, Diyarbakır, Türkiye

**Background/Aims:** Primary biliary cholangitis (PBC) is a chronic autoimmune liver disease that causes damage to the small bile ducts and is more common in women. This study aimed to analyze the demographic, clinical, laboratory, and treatment response characteristics of patients diagnosed with PBC and its relationship with other autoimmune diseases.

**Materials and Methods:** A total of 60 patients with PBC who were followed up in the Gastroenterology Department of Dicle University Medical Faculty Hospital between 2012 and 2023 were included in the study. Age, gender, date of diagnosis, laboratory results at initial presentation, endoscopy findings, liver biopsy, concomitant autoimmune diseases, treatments, and clinical course were analyzed. In addition, the results of complete blood and biochemistry tests performed before and at regular intervals after the initiation of treatment were recorded.

**Results:** A total of 58 (96.7%) of the patients were female and 2 (3.3%) were male. The mean age at initial diagnosis was 54.9 ± 14.2 years. At the time of initial diagnosis, 32 (53.3%) of the patients had pruritus, 44 (73.3%) had fatigue, 13 (21.7%) had insomnia, 5 (8.3%) had hyperpigmentation, 5 (8.3%) had jaundice, 11 (18.3%) had dry mouth, 13 (21.7%) had dry eyes, 13 (21.7%) had hepatomegaly, and 19 (31.7%) had splenomegaly. Overall, 43 (71.7%) of the patients had another concomitant disease. Concomitant autoimmune diseases included Sjögren syndrome in 13 (30.2%), autoimmune hepatitis in 10 (23.2%), Hashimoto thyroiditis in 7 (16.2%), rheumatoid arthritis in 5 (11.6%), scleroderma in 2 (4.6%), Raynaud phenomenon in 1 (2.3%), idiopathic thrombocytopenia in 1 (2.3%), ulcerative colitis in 1 (2.3%), celiac disease in 1 (2.3%), dermatomyositis in 1 (2.3%), and adrenal insufficiency in 1 (2.3%). Antimitochondrial antibody (AMA) was positive in 39 (65%) and negative in 21 (35%) cases. The mean follow-up period was 48.0 ± 34.41 months and the mean survival was 112.3 ± 3.62 months. There was no significant correlation between endoscopy and biopsy results and mortality. Ursodeoxycholic acid (UDCA) treatment was given to all patients, and there was a significant decrease in the laboratory values of the patients after treatment.

**Conclusion:** Most of the patients with PBC were middle-aged women, often accompanied by other autoimmune diseases. AMA-negative PBC was more frequent than expected, and a significant biochemical response to UDCA treatment was observed.

## PS-080 Chemical Gastritis: A Single-Center Case Series

**Mevlut Kıyak**

Department of Gastroenterology, İstanbul University of Health and Technology Faculty of Medicine, Kolan Hospital, İstanbul, Türkiye

**Background/Aims:** Chemical gastritis (reactive/chemical gastropathy) refers to gastric mucosal injury caused by chemical or pharmacological agents. Clinical and endoscopic findings may vary considerably. This condition, frequently encountered in clinical practice and often associated with medication use, presents diverse features in terms of diagnosis and management. In this study, a single-center case series is presented, reporting patient characteristics, exposure history, endoscopic findings, and short-term outcomes.

**Materials and Methods:** Patients diagnosed with chemical/reactive gastritis based on pathology reports within the last 12 months were retrospectively analyzed. Demographic data, presenting symptoms, medication exposure, and endoscopic findings were collected. Descriptive statistical methods were used for analysis.

**Results:** A total of 31 patients were included in the study. The mean age was 47.4 ± 14.5 years, and 61.3% of the patients were female. The most common presenting symptoms were dyspepsia (61%) and reflux complaints (27%). Medication exposure included NSAIDs (32.3%), antiplatelet agents (25.8%), and bisphosphonates (6.5%). Endoscopic findings included pangastritis (65%), antral gastritis (16%), reflux esophagitis (15%), and peptic ulcer (6%). After discontinuation of the offending medication and 8 weeks of proton pump inhibitor (PPI) therapy, clinical improvement was observed in 84% of patients.

**Conclusion:** Chemical gastritis is a common but heterogeneous condition with variable clinical and endoscopic presentations. In this series, NSAID and antiplatelet use were identified as the most significant risk factors. The majority of patients responded favorably to conservative management with PPI therapy. Discontinuation of the causative drug combined with PPI treatment appears to result in a high rate of clinical improvement; however, larger prospective studies are warranted.

## PS-082 Risk Factors for the Development of Portal and Mesenteric Vein Thrombosis

**Yelda Velioğlu Kaya ^1^ , Fatma Yılmaz Öncül ^1^ , İbrahim Akbudak ^2^ , Muhsin Kaya ^1^**

^1^Department of Gastroenterology, Dicle University Faculty of Medicine, Diyarbakır, Türkiye

^2^Department of Radiology, Dicle University Faculty of Medicine, Diyarbakır, Türkiye

**Abstract**

**Background/Aims:** Portal vein thrombosis (PVT) and mesenteric vein thrombosis (MVT) are vascular diseases associated with high morbidity, clinically presenting in various forms ranging from acute intestinal ischemia to chronic portal hypertension. The aim of this study was to reveal the characteristics of patients diagnosed with PVT and MVT.

**Materials and Methods:** The records of patients diagnosed with PVT and/or MVT in the hospital between January 1, 2014 and December 31, 2024 were retrospectively reviewed. Demographic characteristics, accompanying diseases, clinical features, laboratory findings, and radiological and endoscopic findings at the time of the initial diagnosis were recorded.

**Results:** A total of 74 patients with a mean age of 57.2 ± 18 years, including 47 (63.5%) males and 27 (36.5%) females, were diagnosed with PVT and/or MVT. In the development of portal vein thrombosis, cancer was the cause in 25 (34%) cases, cirrhosis in 19 (25.7%), myeloproliferative disease in 2 (3%), Factor V Leiden mutation in 4 (5%), prothrombin gene mutation in 1 (1%), and pregnancy in 2 (3%), whereas the remaining 21 (28%) cases were considered idiopathic. In patients with myeloproliferative disease, unlike those in whom thrombosis developed due to other causes, more extensive thrombosis was present simultaneously. It was determined that the presence of portal hypertensive gastropathy was associated with more frequent thrombosis in the left branch of the portal vein (*P* < .01) and the main portal vein (*P* = .03); the presence of collaterals in the liver hilum was associated with thrombosis in the right branch of the portal vein (*P* = .03) and the splenic vein (*P* = .02); the absence of an extrahepatic mass was associated with right branch portal vein thrombosis (*P* = .02); and the absence of gallstones was associated with superior mesenteric vein thrombosis (*P* < .01).

**Conclusion:** In the majority of the cases, malignancies and cirrhosis were responsible for the development of thrombosis, whereas the idiopathic group was significant. Thrombophilic conditions such as myeloproliferative diseases, Factor V Leiden mutation, and prothrombin gene mutation were found to be less common than expected. The simultaneous development of thrombosis in the portal and mesenteric veins should suggest myeloproliferative diseases.

## PS-084 A Rare Case: Hydroxychloroquine-Associated Pancreatitis

**Vuslat Zorlu Genç ^1^ , Ganime Özge Ekız ^1^ , Merve Eren Durmuş ^2^ , Galıp Egemen Atar ^2^ , Serkan Öcal ^2^**

^1^Department of Internal Medicine, University of Health Sciences Antalya Training and Research Hospital, Antalya, Türkiye

^2^Department of Gastroenterology, University of Health Sciences Antalya Training and Research Hospital, Antalya, Türkiye

Acute pancreatitis is characterized by inflammation of the pancreas, presenting with abdominal pain and increased serum pancreatic enzyme levels. The most common etiologies are gallstones and alcohol, accounting for approximately two-thirds of cases. Drug-induced pancreatitis, on the other hand, constitutes less than 5% of cases. Prognosis is generally favorable, and mortality is low. Here, a case of drug-induced pancreatitis is presented which, to the best of the authors’ knowledge, has not been previously reported. A 69-year-old female patient was admitted to the clinic with acute pancreatitis, presenting with belt-like pain radiating to her back and increased serum amylase-lipase levels. The patient had no history of alcohol use or hypertriglyceridemia. Imaging studies excluded biliary pancreatitis. The patient had a history of hydroxychloroquine (HCQ), methotrexate, and thiazide use due to rheumatoid arthritis and hypertension. She was also hospitalized for acute pancreatitis at the clinic a week prior. Drug-induced pancreatitis was suspected as the etiology. Initially, as HCQ was considered the least likely pancreatitis-inducing agent among her medications, it was restarted first. On the second day of HCQ, the patient’s abdominal pain recurred, and amylase-lipase levels increased approximately 30-fold. Acute pancreatitis was diagnosed, and HCQ was discontinued. Following cessation of the drug, her pain subsided, and enzyme levels decreased. Over 100 drugs are reported as potential causes of drug-induced pancreatitis. HCQ, an antimalarial agent widely used in the treatment of rheumatologic conditions, has not been previously reported to directly induce acute pancreatitis. However, a recent study by Fareed et al (2024) demonstrated that long-term HCQ administration in rats resulted in significant histopathological and biochemical pancreatic injury, suggesting a potential toxic effect of the drug on the exocrine pancreas.

Although HCQ is considered a low-probability agent for inducing pancreatitis, it should be considered in the differential diagnosis of acute pancreatitis, especially when other common causes are excluded.

## PS-085 Leukocytoclastic Vasculitis Associated with Upadacitinib Therapy in Ulcerative Colitis

**Yunus Günegül, İlyas Ethem Şenocak, Talha Ercan, Yunus Emre Demiral, Arda Bilgili, Mukaddes Tozlu, Ahmet Tarık Eminler, Mustafa İhsan Uslan**

Department of Gastroenterology, Sakarya University Faculty of Medicine, Sakarya, Türkiye

Upadacitinib, a selective JAK1 inhibitor, is increasingly used in the management of moderate-to-severe ulcerative colitis. Although various cutaneous adverse reactions have been reported, leukocytoclastic vasculitis (LCV) has not been reported in the literature. A 54-year-old man with a 7-year history of ulcerative colitis, previously treated with multiple biologic agents, was started on upadacitinib during a severe flare. The patient achieved clinical and endoscopic remission shortly after treatment and remained in remission during follow-up. In the fourth month of therapy, he presented with purpuric skin lesions, whereas clinical and endoscopic remission was still maintained (Mayo endoscopic subscore: 1; Mayo Disease Activity Index: 1). A skin biopsy was consistent with leukocytoclastic vasculitis. Laboratory evaluation revealed a CRP level of 3.45 mg/dL, with normal hemoglobin and albumin levels; systemic screening showed no additional pathology. Upadacitinib was discontinued and corticosteroid therapy was initiated, resulting in complete resolution of the skin lesions. During follow-up, remission was maintained under ustekinumab therapy, and leukocytoclastic vasculitis or other dermatologic manifestations did not reappear at subsequent visits. This case reports leukocytoclastic vasculitis as a rare cutaneous adverse event that may be associated with upadacitinib therapy in ulcerative colitis. The remission status at presentation, the absence of additional pathology on systemic evaluation, and the lack of recurrence after drug withdrawal suggest a potential causal relationship. Clinicians should remain aware of this possibility when purpuric skin lesions occur in patients receiving JAK inhibitors.


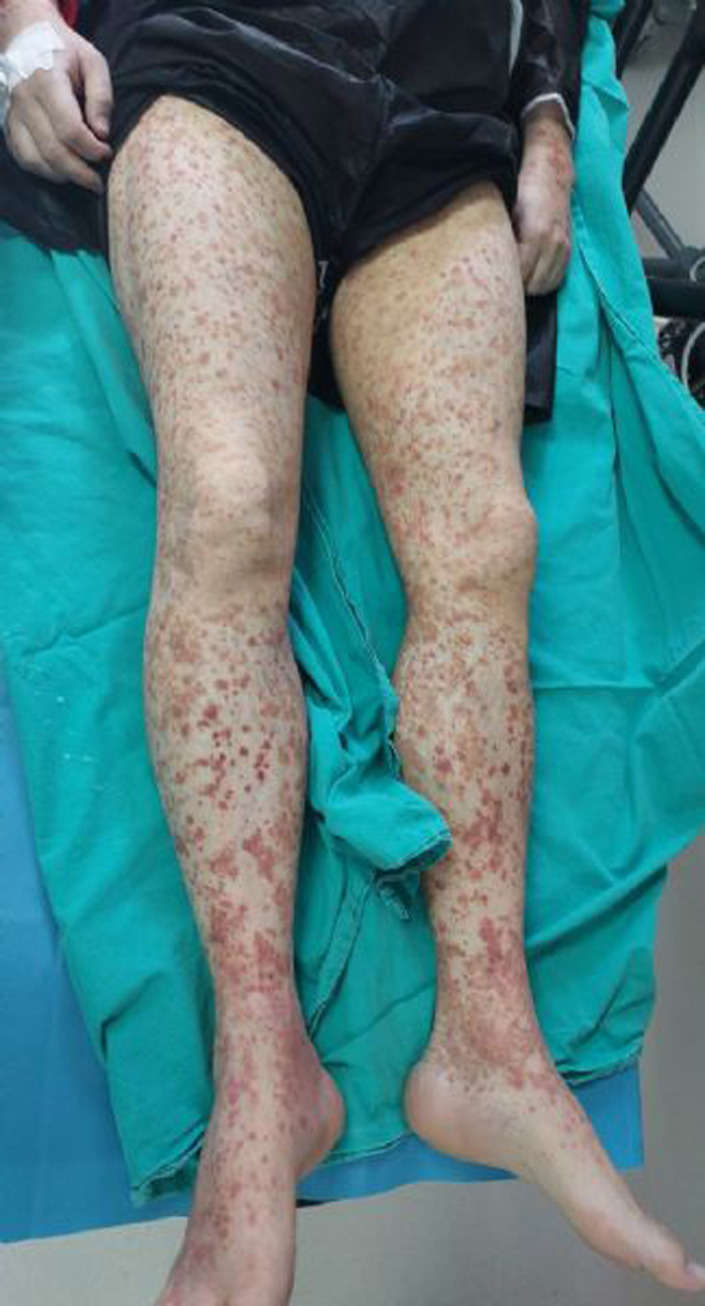


Figure 1. Purpuric cutaneous lesions noted at admission.

## PS-087 Artemisia absinthium (Wormwood)-Induced Toxic Hepatitis: 2 Cases with Contrasting Clinical Outcomes

**Muhammed Furkan Keser, Yahya Atayan, Murat Harputluoğlu, Yüksel Seçkin, Mehmet Ali Erdoğan**

Department of Gastroenterology, İnönü University Faculty of Medicine, Malatya, Türkiye

Artemisia absinthium is traditionally used in Türkiye for gastrointestinal complaints, loss of appetite, bile stimulation, parasitic infections, lactation, and as a diuretic. However, due to its content of thujone, it may cause hepatotoxicity. Reports of A. absinthium-related toxic hepatitis are extremely rare. Two such cases with contrasting outcomes are presented. A 52-year-old man consumed ~10 g/day of wormwood infusion (200 mL) for 1 week for urinary complaints. He was admitted with acute liver failure. Laboratory results showed AST 1279 U/L, ALT 1174 U/L (>30× normal), INR 1.8, total bilirubin 32.3 mg/dL, direct bilirubin 15.3 mg/dL, and GGT 113 U/L. Viral and autoimmune causes were excluded. Supportive therapy over 69 days included intermittent plasmapheresis (hepatic replacement therapy), branched-chain amino acids, L-ornithine L-aspartate, lactulose, dextrose, and antibiotics. He remained stable until day 69, when encephalopathy developed. Cadaveric liver transplantation was performed on day 71, but he died 25 days later from multiple organ failure. Histology showed hepatocyte loss, portal inflammation, focal cholestasis, ductular proliferation, and pericellular fibrosis, consistent with toxic hepatitis. A 24-year-old woman ingested ~5 g/day of wormwood infusion (200 mL) for 3 days to promote lactation. She presented with fatigue and jaundice. Labs showed AST 1618 U/L, ALT 1655 U/L, total bilirubin 15.7 mg/dL, direct bilirubin 8.0 mg/dL, ALP 266 U/L, GGT 144 U/L, and LDH 569 U/L, with normal renal function. Viral and autoimmune causes were excluded. Supportive treatment (lactulose, fluids, symptomatic care) was provided. She improved progressively and recovered completely after 32 days of follow-up. Artemisia absinthium is widely perceived as harmless but may cause severe hepatotoxicity. These cases demonstrate its broad clinical spectrum, from full recovery to transplantation and death. A detailed history of herbal use should be obtained in acute hepatitis, and awareness of this risk must be raised.

## PS-089 Chronic Diarrhea Developing After Celiac Artery Aneurysm Surgery

**Civanmert Bayrak**

Department of Gastroenterology, Dokuz Eylül University Faculty of Medicine, İzmir, Türkiye

A 63-year-old woman was admitted to the gastroenterology clinic for evaluation of chronic diarrhea. She has a history of hypertension and restless legs

syndrome, as well as a previous aneurysm surgery. The patient has a 40-pack-year smoking history. She presented with 7-8 episodes of watery, yellow, bloodless, mucusless diarrhea per day. Tests revealed negative stool analyses, including direct stool examination, stool culture, IgA, TSH, T4, viral hepatitis, and HIV serology. She underwent endoscopy and colonoscopy, which showed endoscopic erythematous pangastritis with biopsy.

Colonoscopy results were normal, and a biopsy is currently being monitored for possible microscopic colitis. The patient was started on loperamide, taking 2 grams 4 times a day, which decreased the frequency of diarrhea to 3-4 episodes per day. Approximately 2.5 months before her admission to the Gastroenterology Service, interventional radiology deemed endovascular treatment inappropriate for a celiac artery aneurysm. A celiac artery aneurysm operation was performed by a cardiovascular surgeon. During this operation, the celiac artery was found to be aneurysmal before the bifurcation, and an arteriotomy was performed in the affected area. The patient’s preoperative angiography report performed by interventional radiology is presented in the images. Based on all these examinations, it is believed that the aneurysm operation was performed after the celiac ganglion was affected.

Table 1.Biochemistry Laboratory Results

Table 2.Complete Blood Count (Hemogram)

**Table:** 

Parameter	Result	Unit	Reference Range	Previous Result
Blood urea nitrogen (BUN)	9.4	mg/dL	8-23	13.9/41.4
Glucose	104	mg/dL	70-100	84/74
Creatinine	0.65	mg/dL	0.6-1.1	0.77/0.87
eGFR (CKD-EPI)	95			82/71
Uric acid	5.58	mg/dL	2.6-6	6.30/6.96
Aspartate aminotransferase (AST)	16	U/L	0-35	16/13
Alanine aminotransferase (ALT)	15	U/L	0-35	17/19
Gamma-glutamyl transferase (GGT)	9	U/L	1-39	9/10
Alkaline phosphatase (ALP)	64	U/L	30-120	63/64
Indirect bilirubin	0.31	mg/dL		0.55/0.32
Total bilirubin	0.35	mg/dL	0.3-1.2	0.68/0.42
Direct bilirubin	0.04	mg/dL	0-0.2	0.13/0.10
Lactate dehydrogenase (LDH)	139	U/L	125-220	145/94
Total protein	4.90	g/dL	6-8.3	4.76/4.89
Albumin	3.02	g/dL	3.5-5.2	3.24/3.42
Total protein (g/L)	49	g/L	60-83	47.6/48.9
Albumin (g/L)	30.2	g/L	35-52	32.4/34.2
Sodium	142	mmol/L	136-145	142/138
Potassium	3.09	mmol/L	3.5-5.1	4.39/4.05
Chloride	113	mmol/L	98-113	113/109
Calcium	8.01	mg/dL	8.8-10.6	8.87/8.64
Phosphorus (inorganic)	2.64	mg/dL	2.8-4.1	2.99/2.78
Magnesium	0.71	mmol/L	0.77-1.03	0.83/0.88
C-reactive protein (CRP)	0.8	mg/L	0.2-5	0.8/2.0

**Table:** 

Parameter	Result	Unit	Reference Range	Previous Result
WBC	4.5	10^3^/µL	4-10.3	4.9/5.8
NEU %	49.8	%	41-73	45.4/52.1
LYM %	40.0	%	19.4-44.9	41.4/38.0
MONO %	6.9	%	5.1-10.9	9.5/7.3
BASO %	0.5	%	0.3-1.5	0.5/0.4
EOS %	2.8	%	0.9-6	3.2/2.2
NEU #	2.2	10^3^/µL	2.1-6.1	2.2/3.0
LYM #	1.8	10^3^/µL	1.3-3.5	2.0/2.2
MONO #	0.3	10^3^/µL	0.3-0.9	0.5/0.4
EOS #	0.1	10^3^/µL	0-0.5	0.2/0.1
BASO #	0.0	10^3^/µL	0-0.2	0.0/0.0
RBC	2.90	10^6^/µL	4-5.77	2.85/2.73
Hemoglobin (HGB)	9.5	g/dL	12-16	9.4/8.7
Hematocrit (HCT)	27.4	%	36-46	26.7/25.5
MCV	94.5	fL	80.7-95.5	93.9/93.4
MCH	32.6	pg	27.2-33.5	33.1/32.0
MCHC	34.5	g/dL	32.7-35.6	35.3/34.3
RDW	15.0	%	11.8-14.3	14.6/14.9
Platelet (PLT)	159	10^3^/µL	156-373	145/147
MPV	9.0	fL	6.9-10.8	8.7/9.0
PCT	0.144	%	0.20-0.36	0.125/0.132

## PS-090 Management of Gastric Leakage Following Fundoplication with Stent Placement

**Tuba Erürker Öztürk**

Gastroenterology Clinic, Denizli Denipol Hospital, Denizli, Türkiye

Fundoplication is a surgical procedure commonly used to treat gastroesophageal reflux disease (GERD) and hiatal hernia. Although the overall incidence of complications is approximately 4.1%, gastric or esophageal perforation occurs in about 0.9% of cases (2). Early diagnosis and management of such complications are crucial for improving survival outcomes. In this case report, it is proposed that a fully covered mega self-expandable metallic stent (SEMS) can be an effective therapeutic option for managing gastric perforation following laparoscopic fundoplication. A 65-year-old female patient with known hepatitis B carrier status underwent laparoscopic Nissen fundoplication in February 2025 for a 6 cm hiatal hernia. Preoperative laboratory results were within normal limits. Postoperatively, the patient developed bilateral pleural effusions, necessitating bilateral chest tube placement. On postoperative day 2, approximately 100 mL of purulent fluid was observed from the abdominal drain, with a C-reactive protein (CRP) level of 379 mg/L. Consequently, an exploratory laparotomy was performed, during which a primary leak was identified and sutured. Despite this intervention, CRP levels remained increased and drain output persisted. An upper gastrointestinal endoscopy was performed. The esophagus appeared normal, but upon entering the stomach, purulent drainage was visualized at the 10 o’clock position of the proximal stomach. Due to persistent leakage, a fully covered mega SEMS was placed, extending from the esophagus through the pylorus, and a nasojejunal feeding tube was inserted through the stent. The nasojejunal tube was removed after 2 weeks, and the patient was transitioned to oral feeding. As no further output was observed from the thoracic or abdominal drains, both were removed. At week 6, the SEMS was also extracted. No residual pus or leakage was observed in the stomach. However, unlike sleeve gastrectomy, fundoplication does not reduce gastric volume, which may pose a risk of stent migration or displacement. Nevertheless, this case demonstrates that SEMS may serve as an effective and feasible treatment option for gastric leaks occurring after fundoplication procedures.


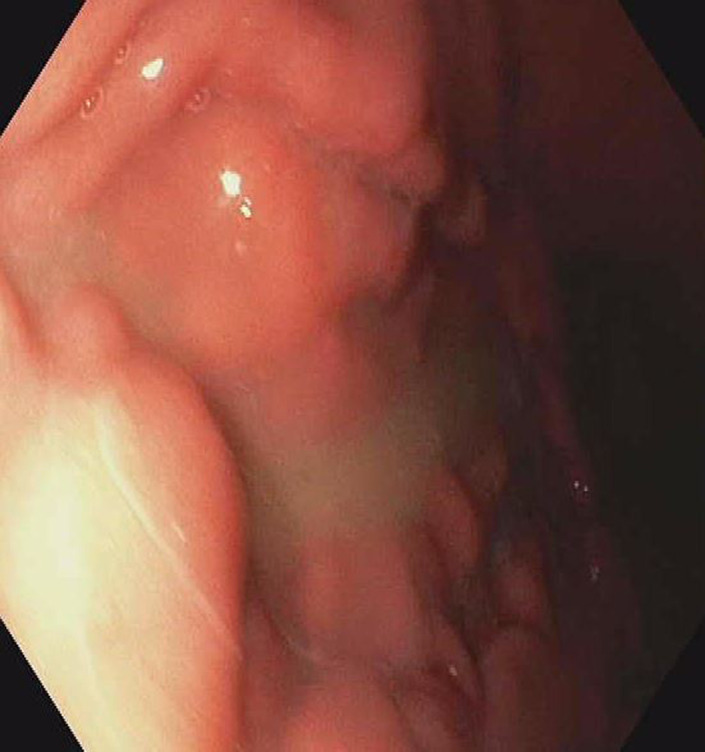


Figure 1. Postoperative endoscopy.


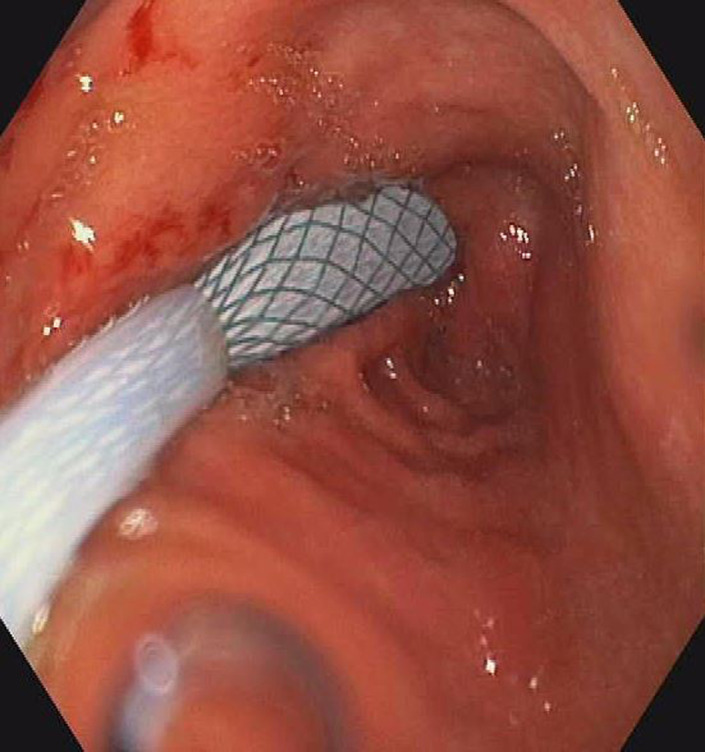


Figure 2. Stent placement.


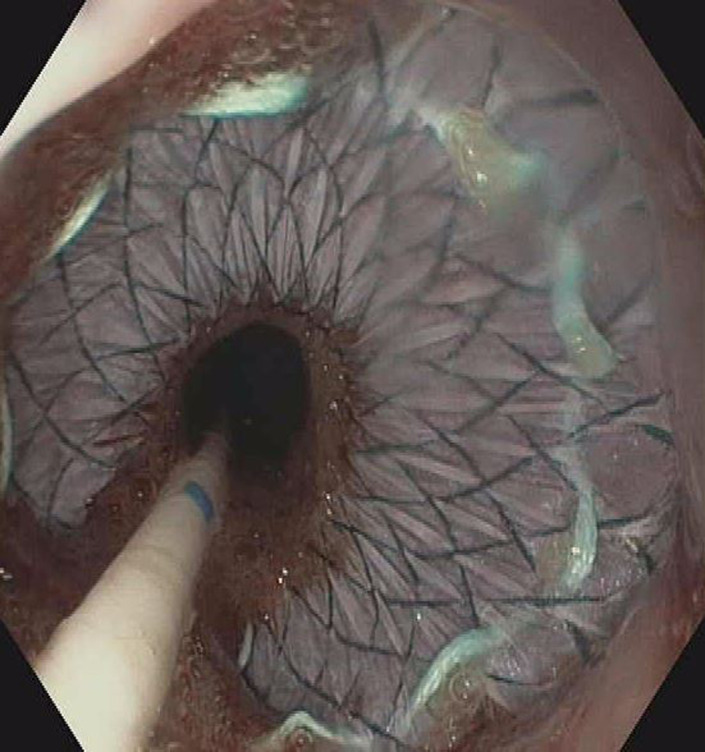


Figure 3. Nasojejunal placement.

## PS-091 ERCP in a Patient with Situs Totalis Inversus: A Case Report

**Gülce Çelik Günsay, Uğur Çiftçi, Coşkun Özer Demirtaş**

Department of Gastroenterology, Marmara University Faculty of Medicine, İstanbul, Türkiye

Situs inversus totalis (SIT) is a rare autosomal recessive congenital anomaly resulting in the transposition of the left and right anatomy in a mirror image. Because of these anatomical changes, endoscopic retrograde cholangiopancreatography (ERCP) in this population is more challenging and complicated. Herein, the case of a 19-year-old Turkish man with total SITis reported, who presented with obstructive jaundice, left upper quadrant pain, and nausea. The patient underwent a therapeutic ERCP for choledocholithiasis. The procedure was initiated while the patient was in a prone position, with the endoscopist standing on the right side of the patient. Once the pylorus was passed, the patient was placed in a left lateral decubitus position, and endoscopic sphincterotomy and endoscopic balloon dilatation were successfully performed.


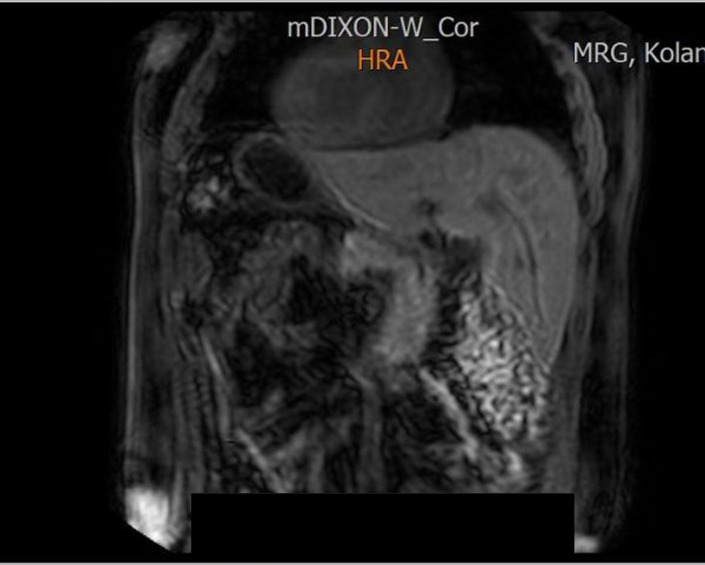


Figure 1. MRCP image.


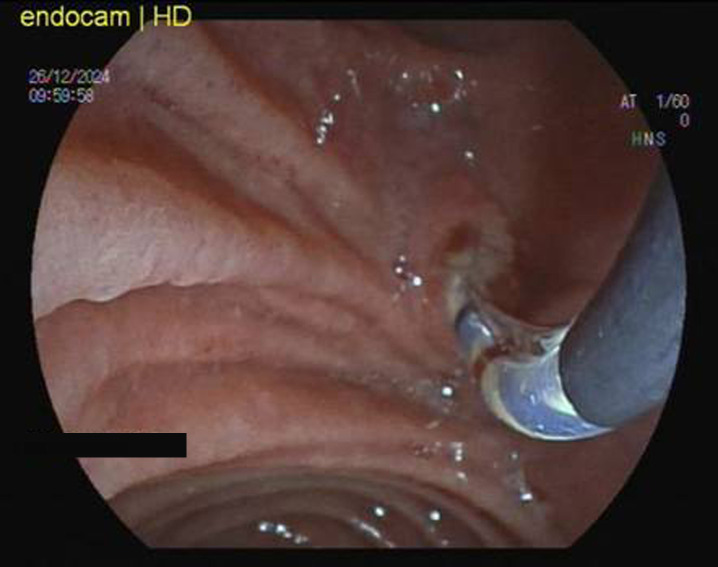


Figure 2. Biliary sphincterotomy in a patient with SIT.


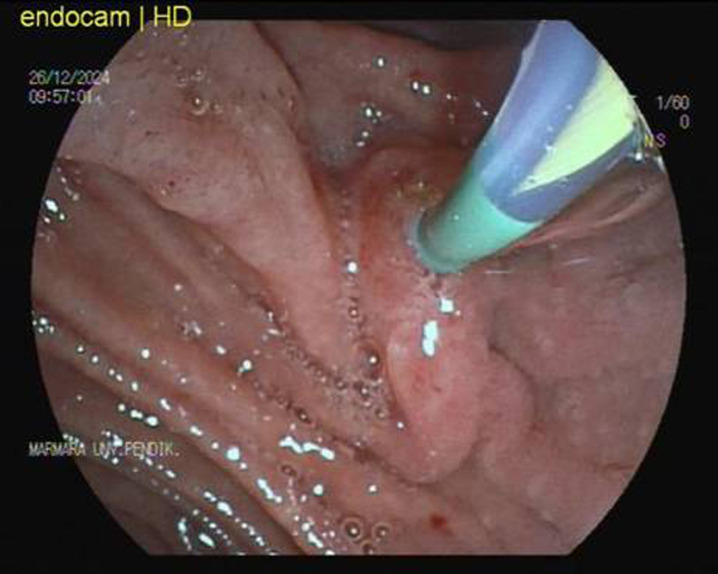


Figure 3. Papilla and biliary cannulation in a patient with SIT.

## PS-092 An Incidentally Detected Antral Hemangioma with Unusual Morphology and Localization

**Rohat Sakar ^1^ , Ismail Hakki Kalkan ^2^ , Mehmet Arhan ^3^ , Omer Gunhan ^4^ , Fatma Dedeoglu ^4^**

^1^TOBB University of Economics and Technology Faculty of Medicine, Ankara, Türkiye

^2^Department of Gastroenterology, TOBB University of Economics and Technology Faculty of Medicine, Ankara, Türkiye

^3^Department of Gastroenterology, Gazi University Faculty of Medicine, Ankara, Türkiye

^4^Department of Pathology, TOBB University of Economics and Technology Faculty of Medicine, Ankara, Türkiye

Gastric hemangiomas are rare vascular malformations that pose significant diagnostic challenges, particularly when they present with atypical morphology and localization. This case report reports an incidentally detected antral hemangioma with unique features that deviate from typical presentations documented in the literature. A 35-year-old female presented with an 8-week history of heartburn and dyspepsia. Her medical history was significant for Hashimoto’s thyroiditis, which was treated with levothyroxine. Upper gastrointestinal endoscopy revealed a 1×2 cm hyperemic lesion with distinct borders in the gastric antrum, demonstrating intense vascularity on narrow band imaging. Computed tomography confirmed the diagnosis of antral hemangioma, measuring 9×5 mm on the anterior wall of the gastric antrum. Given the absence of symptoms directly attributable to the hemangioma and the lack of complications, conservative management with surveillance was adopted. The patient remained asymptomatic during 6 months of routine monitoring without any notable incidents or complications related to the finding. This case highlights the diagnostic complexity of gastric hemangiomas presenting with atypical flat mucosal morphology rather than the typical submucosal protrusion and unusual antral localization. The successful identification through endoscopy and confirmation with CT imaging emphasizes the importance of advanced imaging techniques in diagnosing vascular lesions with atypical presentations.


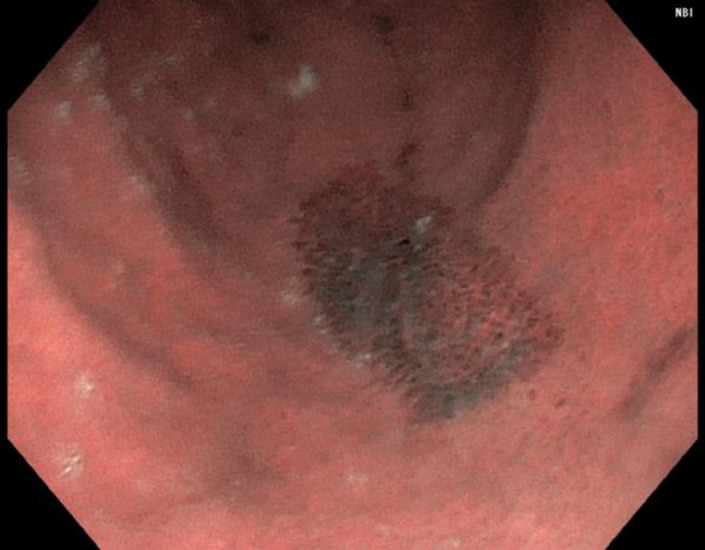


Figure 1. Endoscopic visualization of antral hemangioma. 1 × 2-cm hyperemic lesion with distinct borders clearly demarcated from normal mucosa in the gastric antrum.


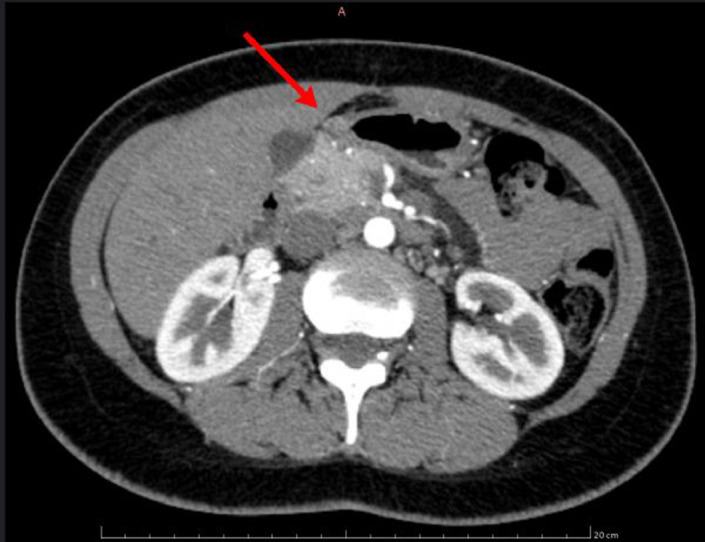


Figure 2. Computed tomography (CT) of antral hemangioma. 9 × 5-mm area on the anterior wall of the gastric antrum that appeared relatively more opaque during the arterial phase compared to surrounding tissue (red arrow).

## PS-094 De Novo Crohn’s Disease after Mini-Gastric Bypass: A Case Report

**Önder Buğra Kaynarca ^1^ , Mehmet Kürşad Keskin ^2^ , Nizameddin Koca ^1^**

^1^Department of Internal Medicine, University of Health Sciences Bursa City Hospital, Bursa, Türkiye

^2^Department of Gastroenterology, University of Health Sciences Bursa City Hospital, Bursa, Türkiye

Obesity and inflammatory bowel disease (IBD) are increasingly prevalent global health issues. Traditionally, these conditions have been viewed as distinct entities; however, they are now recognized to share overlapping pathophysiological mechanisms involving chronic low-grade inflammation, immune dysregulation, and alterations in gut microbiota. Bariatric surgery is an effective intervention for severe obesity and has documented metabolic benefits. However, emerging evidence suggests a potential association between bariatric procedures and de novo IBD in some patients. The case of a 64-year-old woman with a history of type 2 diabetes mellitus (T2DM) and diabetic complications is reported. The patient presented to the outpatient clinic with persistent abdominal pain, recurrent nausea, and unintentional weight loss. Her medical history revealed that she had undergone mini-gastric bypass surgery 6 years earlier. Despite a prior cholecystectomy and multiple inconclusive endoscopic investigations, her symptoms persisted. Magnetic resonance enterography revealed segmental ileal wall thickening and edema in the jejunum. Subsequent endoscopic biopsies of the duodenal bulb and terminal ileum confirmed chronic inflammatory changes consistent with Crohn’s disease. The patient was managed with oral mesalamine, corticosteroids, and proton pump inhibitors, and clinical improvement was noted at follow-up. This case highlights the possibility of de novo Crohn’s disease following bariatric surgery. Although a causal relationship remains unproven, accumulating evidence indicates that altered gut homeostasis, dysregulated immune response, microbial shifts, and changes in bile acid metabolism postsurgery may contribute to IBD pathogenesis. Among patients who have undergone bariatric surgery, clinicians should maintain a high index of suspicion for IBD when unexplained gastrointestinal symptoms occur, even long after the surgery.


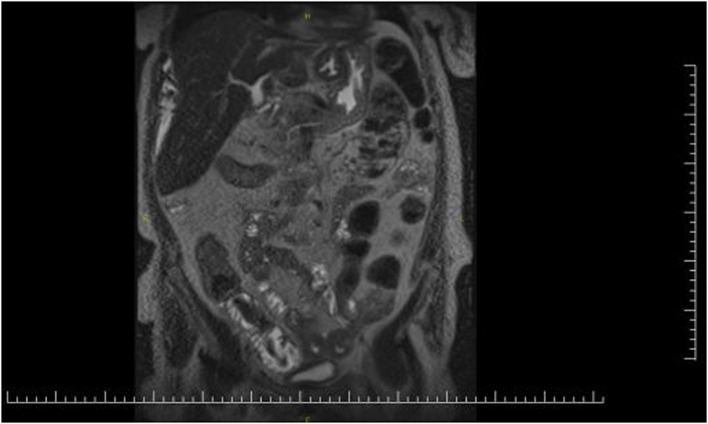


Figure 1. Coronal magnetic resonance enterography (MRE) showing mural thickening and submucosal edema in ileal loops, consistent with active inflammation suggestive of Crohn’s disease.


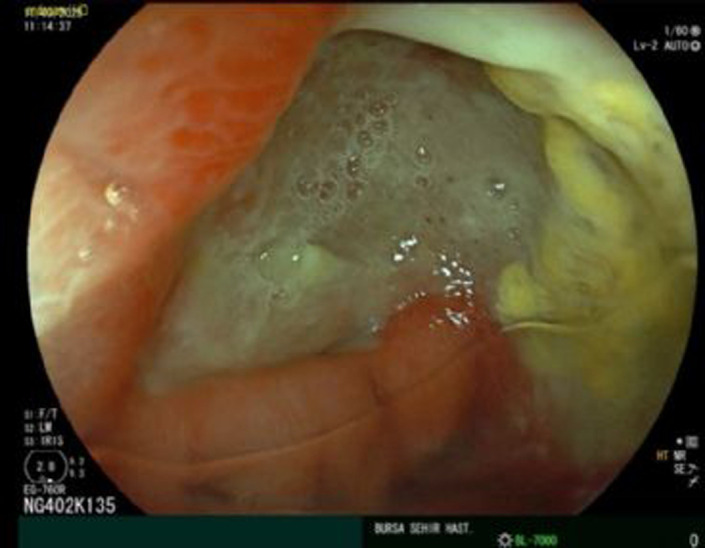


Figure 2. Endoscopic image of the bulb demonstrating mucosal ulceration with surrounding erythema and exudate.


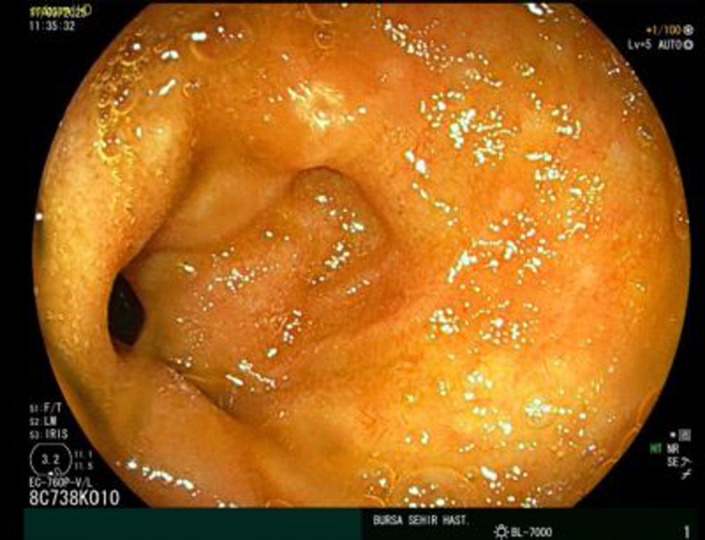


Figure 3. Colonoscopy image showing aphthous ulcerations and mucosal edema in the terminal ileum.

## PS-095 A Rare Case of Breast Cancer Presenting with Acute Budd-Chiari Syndrome and a Fatal Course

**Muhammed Furkan Keser ^1^ , Yahya Atayan ^1^ , Yüksel Seçkin ^1^ , Oğuzhan Yıldırım ^1^ , Ayşe Hafsa Çağın ^2^**

^1^Department of Gastroenterology, İnönü University Faculty of Medicine, Malatya, Türkiye

^2^Department of Internal Medicine, Necmettin Erbakan University Faculty of Medicine, Konya, Türkiye

Budd-Chiari syndrome is a rare, life-threatening condition caused by hepatic venous outflow obstruction from thrombosis or compression. It is most often linked to hematologic disorders and hepatocellular carcinoma, whereas its association with breast cancer is extremely rare. Acute Budd-Chiari syndrome as the first manifestation of breast cancer is exceptional. In this case report, a rare patient with breast cancer initially manifesting as acute Budd-Chiari syndrome with a fatal course is presented. A 34-year-old woman presented with fatigue, jaundice, and right upper quadrant fullness. Physical examination revealed hepatomegaly. Laboratory tests showed total bilirubin 31.8 mg/dL, AST 1138 U/L, ALT 335 U/L, ALP 1006 U/L, GGT 346 U/L, LDH 1673 U/L, and INR 1.52. Doppler ultrasonography and dynamic liver CT demonstrated loss of hepatic venous flow and heterogeneous perfusion, consistent with acute Budd-Chiari syndrome. Incidentally, an 8-cm right breast mass was detected. Breast ultrasonography classified the lesion as BIRADS 5, and biopsy confirmed invasive breast carcinoma. Anticoagulation was started, but interventional recanalization of the hepatic veins cannot be performed. Two days after diagnosis, the patient developed hepatic encephalopathy. Because of the newly diagnosed malignancy, liver transplantation and TIPS were not performed. Despite intensive supportive management, including hepatic replacement therapy, the patient deteriorated and died on day 4 of hospitalization. Budd-Chiari syndrome is usually associated with hematologic disorders or hepatic malignancies, whereas its development secondary to extrahepatic solid tumors is very rare. Breast cancer initially presenting with Budd-Chiari syndrome has been reported only rarely. In this case, acute Budd-Chiari syndrome was the first manifestation of breast cancer and progressed rapidly. This rare association emphasizes the need to consider unusual etiologies in the evaluation of Budd-Chiari syndrome.


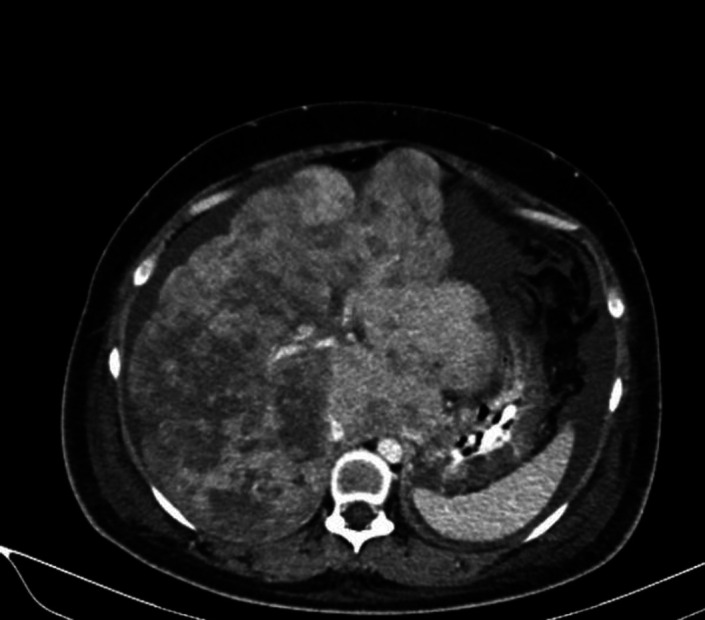


Figure 1. Dynamic liver CT demonstrating findings consistent with Budd-Chiari syndrome.


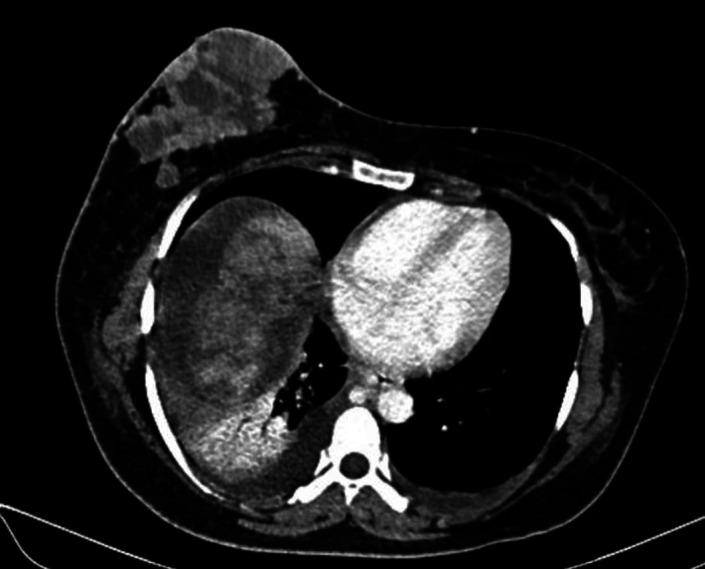


Figure 2. Incidental right breast mass detected on dynamic liver CT.

## PS-096 Colonic Leiomyoma Discovered on Screening Colonoscopy: Endoscopic Resection and Diagnosis

**Rohat Sakar ^1^ , Ismail Hakki Kalkan ^2^ , Mehmet Arhan ^3^ , Omer Gunhan ^4^ , Fatma Dedeoglu ^4^**

^1^TOBB University of Economics and Technology Faculty of Medicine, Ankara, Türkiye

^2^Department of Gastroenterology, TOBB University of Economics and Technology Faculty of Medicine, Ankara, Türkiye

^3^Department of Gastroenterology, Gazi University Faculty of Medicine, Ankara, Türkiye

^4^Department of Pathology, TOBB University of Economics and Technology Faculty of Medicine, Ankara, Türkiye

Colonic leiomyomas are exceptionally rare benign smooth muscle tumors, accounting for only 3% of all gastrointestinal leiomyomas. This case highlights the diagnostic challenges and successful management of an incidentally discovered colonic leiomyoma during routine screening colonoscopy. A 53-year-old asymptomatic man underwent routine colorectal cancer screening. Colonoscopy revealed a 12- to 13-mm sessile polypoid lesion at the rectosigmoid junction. Endoscopic polypectomy with submucosal adrenaline injection was successfully performed. Histopathological examination confirmed the colonic leiomyoma, showing well-circumscribed spindle-shaped eosinophilic cells without atypia, necrosis, or mitotic activity. Colonic leiomyomas are benign with a low risk of recurrence. Precise diagnosis is essential to avoid unnecessary interventions. Increased awareness and diagnostic advancements facilitate the accurate identification and effective management of these rare entities.


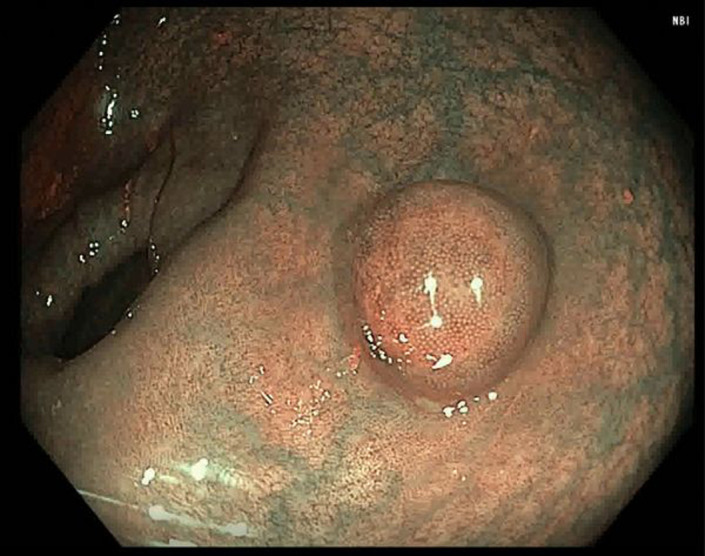


Figure 1. Lesion under NBI (narrow band imaging) at the rectosigmoid junction.


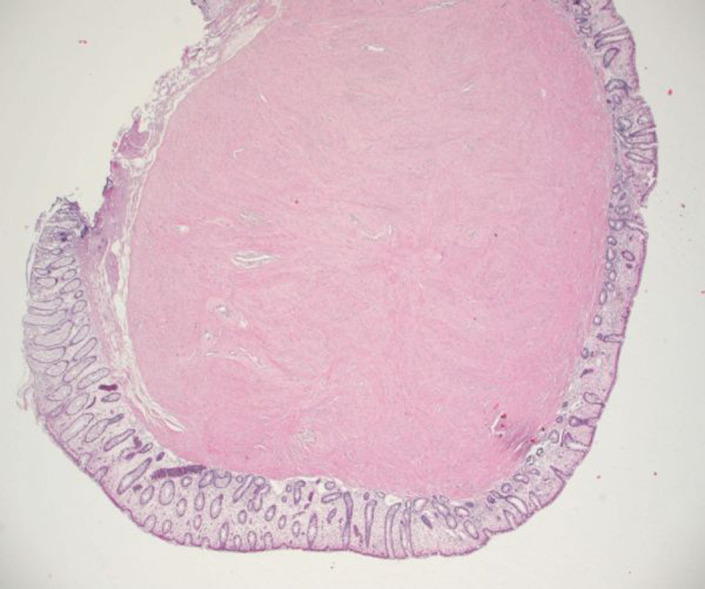


Figure 2. Histopathology of myomatous lesion composed of well-circumscribed spindle-shaped eosinophilic cells.

## PS-097 Esophageal Melanosis in a 38-Year-Old Woman

**Rohat Sakar ^1^ , Ismail Hakki Kalkan ^2^ , Mehmet Arhan ^3^ , Omer Gunhan ^4^ , Fatma Dedeoglu ^4^**

^1^TOBB University of Economics and Technology Faculty of Medicine, Ankara, Türkiye

^2^Department of Gastroenterology, TOBB University of Economics and Technology Faculty of Medicine, Ankara, Türkiye

^3^Department of Gastroenterology, Gazi University Faculty of Medicine, Ankara, Türkiye

^4^Department of Pathology, TOBB University of Economics and Technology Faculty of Medicine, Ankara, Türkiye

Esophageal melanosis (EM) is a rare benign condition characterized by abnormal melanin deposition in the esophageal mucosa. This case report presents an unusual occurrence of EM in a younger female patient, contributing to the limited literature on the presentation of this condition outside typical demographics. A 38-year-old woman presented with stomach pain and was subsequently diagnosed with esophageal melanosis during an endoscopic evaluation. Initial workup revealed gallbladder sludge, a hepatic cyst, and *Helicobacter pylori* infection. Endoscopy demonstrated characteristic black pigmented lesions in the distal esophagus, confirmed histopathologically as melanin deposition without significant melanocyte proliferation. The patient received *H. pylori* eradication therapy and endoscopic management with hemostatic clips for a mucosal defect. Postprocedural imaging showed no complications, and the patient had favorable outcomes with complete recovery. This case highlights the importance of recognizing EM in younger patients and females, populations typically underrepresented in the EM literature. Although generally benign, the potential association with chronic mucosal injury and malignancy warrants careful evaluation and follow-up.


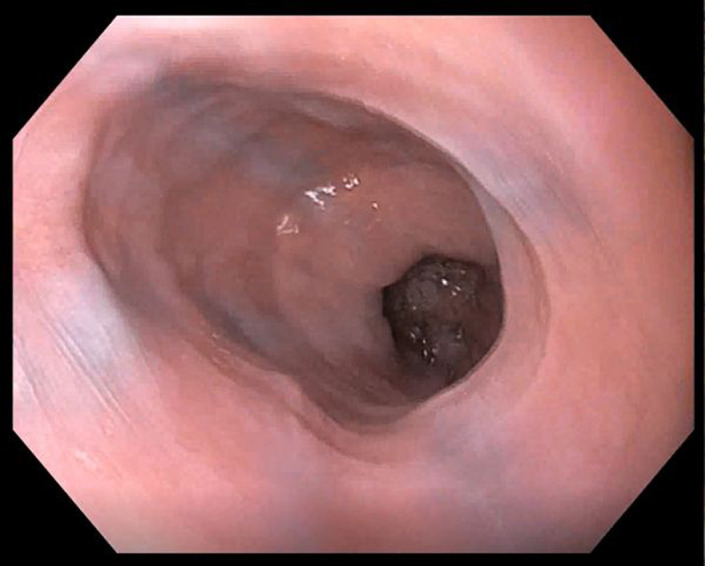


Figure 1. Endoscopic visualization of esophageal melanosis. Numerous darkly pigmented lesions were observed in a linear pattern in the distal esophagus.


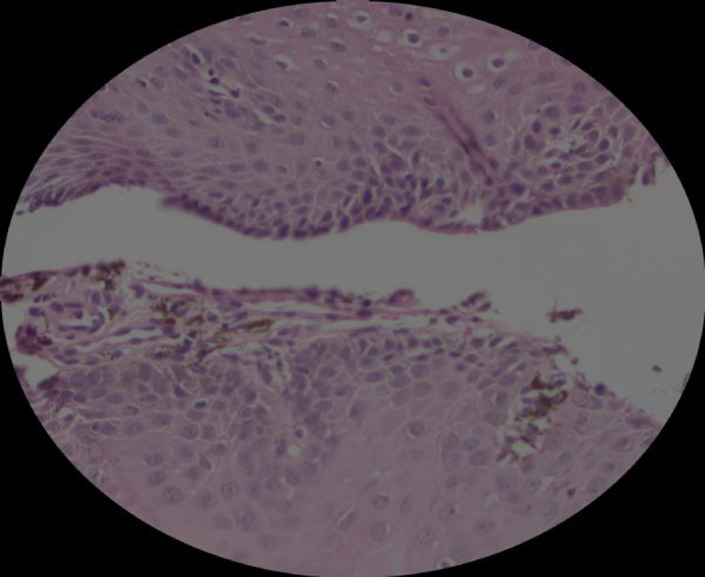


Figure 2. Histopathology of esophageal melanosis. An increase in melanin was observed in the basal layer of the esophageal epithelium, and dark brown melanin was seen within melanophages in the connective tissue beneath the epithelium.

## PS-098 Compliance with Hepatitis B Immunoglobulin Treatment in Patients Undergoing Liver Transplantation due to HBV+Delta Infection: Single-Center Experience

**İsmail Atasoy ^1^ , Nilay Danis ^1^ , Tarkan Ünek ^2^ , Mesut Akarsu ^1^**

^1^Division of Gastroenterology, Department of Internal Medicine, Dokuz Eylül University, İzmir, Türkiye

^2^Department of General Surgery, Dokuz Eylül University, İzmir, Türkiye

**Background/Aims:** This study aims to evaluate adherence to antiviral therapy and hepatitis B immunoglobulin (HBIG) administration among liver transplant recipients who underwent transplantation due to HBV+HDV infection between 1998 and 2024 at Dokuz Eylul University Faculty of Medicine Hospital. The study also includes the pandemic period to assess treatment continuity.

**Materials and Methods:** Designed as a descriptive and cross-sectional study, all liver transplants performed between 1998 and 2024 at the center were reviewed, and data from 97 surviving patients who underwent transplantation due to HBV+HDV infection were collected. The study is ongoing, and between August 15, 2025, and October 15, 2025, patients are being contacted using phone numbers obtained from the hospital’s patient data system.

**Results:** To date, 20 patients have been called, 15 patients have been reached, and 5 patients cannot be contacted. Of these, 3 had changed their phone numbers, and 2 cannot be reached despite repeated attempts. Among the 15 patients who were reached, 9 were followed up in the gastroenterology clinic and were receiving only antiviral therapy without HBIG. These patients were counseled on the importance of HBIG. The remaining 6 patients were receiving both antiviral therapy and HBIG regularly and were under routine gastroenterology follow-up.

**Conclusion:** Continuous adherence to antiviral and HBIG therapy is critical for graft survival in liver transplant recipients with HBV+HDV infection. This study represents a preliminary report, and as more patients are contacted, adherence levels will be clarified, and the impact of patient education strategies on treatment adherence will be further evaluated.

## PS-099 The Importance of Regular Endoscopic Follow-Up After Colectomy in a Case of Familial Adenomatous Polyposis

**Melike Derici ^1^ , Alper Uysal ^2^ , Selçuk Candan ^2^ , Murat Akarsu ^1^ , Şengül Aydın Yoldemir ^1^ , Ömür Tabak ^1^**

^1^Department of Internal Medicine, Kanuni Sultan Süleyman Training and Research Hospital, İstanbul, Türkiye

^2^Gastroenterology Clinic, Kanuni Sultan Süleyman Training and Research Hospital, İstanbul, Türkiye

Familial adenomatous polyposis (FAP) is an autosomal dominant inherited disorder caused by mutations in the APC gene, characterized by the presence of hundreds to thousands of colorectal adenomas. If left untreated, FAP inevitably progresses to colorectal cancer in nearly all patients. Even after prophylactic colectomy, the risk of neoplasia in the upper gastrointestinal tract, particularly in the duodenum and periampullary region, remains significant. Therefore, regular endoscopic surveillance is essential. Herein, a case of periampullary tumor development in a patient with FAP and a history of colectomy is presented, highlighting the importance of continued surveillance.

## PS-101 Ultrasonographic Diagnosis of a Delayed-Recognition Case of Peutz-Jeghers Syndrome

**Mammadhasan Mammadov ^1^ , Osman Özdoğan ^1^ , Ege Altan ^2^ , Umit Yeşilova ^1^ , Ahmet Emre Ergan ^1^ , Mehmet Kasım Aydın ^1^ , Serkan Yaraş ^1^ , Fehmi Ateş ^1^ , Engin Altıntaş ^1^ , Orhan Sezgin ^1^**

^1^Department of Gastroenterology, Mersin University Faculty of Medicine, Mersin, Türkiye

^2^Adana Dr. Turgut Noyan Training and Research Hospital, Adana, Türkiye

Peutz-Jeghers syndrome (PJS) is a rare autosomal dominant disorder characterized by multiple hamartomatous polyps in the gastrointestinal tract, mucocutaneous pigmentation, and an increased risk of malignancy. The most common finding is a mutation in the STK11 (LKB1) gene (50%-70%). Polyps are usually located in the small intestine but may also be present in the colon and stomach. Patients with PJS may present with abdominal pain, bleeding, chronic anemia, intussusception, and obstruction. Ultrasonography (USG) is a rapid, safe, and cost-effective method used in clinical diagnosis. A foreign patient with a 5-year history of constipation, abdominal pain, fatigue, and iron deficiency anemia had been evaluated at different centers. In October 2024, the patient was admitted to the clinic. A peripheral smear showed no atypical cells; hemoglobin was 11 g/dL, ferritin 4 ng/mL, MCV 70 fL, and transferrin saturation was 15. The patient’s mother had cervical cancer, but the PAP smear test was negative. Abdominal USG revealed a pseudokidney sign due to intussusception in the left upper quadrant near the diaphragm. Contrast-enhanced computed tomography, endoscopy, and colonoscopy were planned. CT demonstrated segmental thickening of the proximal jejunal wall. Endoscopy and colonoscopy revealed hyperemic gastropathy and grade 1 hemorrhoids. Double-balloon enteroscopy detected a 5 cm pedunculated polyp at the 50 cm mark of the jejunum, which was removed by polypectomy. Histopathological examination was consistent with a PJS polyp. The patient was diagnosed with PJS due to a positive STK11 mutation and was placed under routine follow-up. This case highlights the importance of USG as a safe and rapid first-line method in diagnosing gastrointestinal diseases. In patients presenting with abdominal pain, distension, mass, or bleeding, USG should be used for early diagnosis and follow-up.


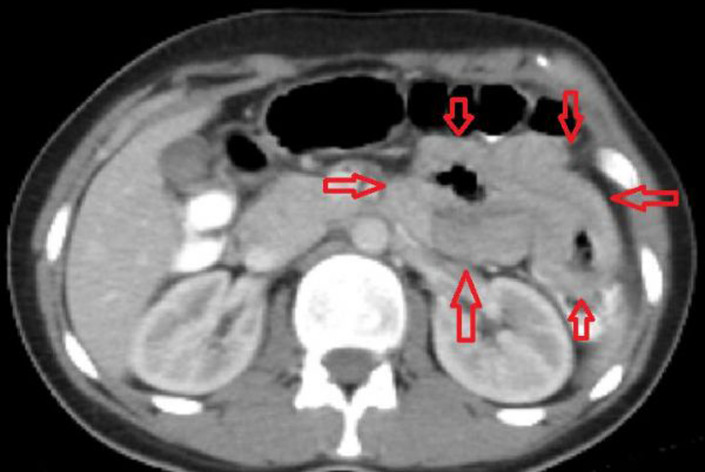


Figure 1. Thickening of the jejunal loop on computed tomography.


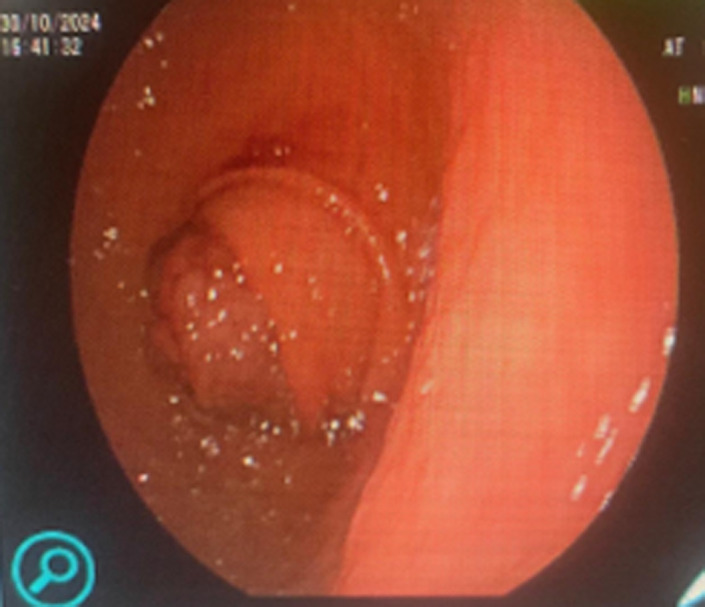


Figure 2. Appearance of a hamartomatous polyp on double-balloon enteroscopy.

## PS-102 First Turkish Trial: Diagnostic Effectiveness of Capsule Endoscopy and Double-Balloon Enteroscopy in Suspected Small Intestinal Disease

**Batuhan Oğuz ^1^ , Esra Bayar ^2^ , Meltem Ergün ^2^ , Ümit Akyüz ^3^ , Cengiz Pata ^2^**

^1^Department of Cardiology, The Essex Cardiothoracic Centre, Basildon University Hospital, London, United Kingdom

^2^Department of Gastroenterology, Yeditepe University Faculty of Medicine, İstanbul, Türkiye.

^3^Department of Gastroenterology, Fatih Sultan Mehmet Training and Research Hospital, İstanbul, Türkiye

**Background/Aims:** Much progress has been made in the diagnosis and treatment of small bowel disorders since the advent of capsule endoscopy (CE) and double-balloon endoscopy (DBE). The aim of this study is to compare the effectiveness of CE and DBE to determine the optimal diagnostic approach.

**Materials and Methods:** In 2022, a total of 24 patients (7 females, 17 males) who met the inclusion and exclusion criteria and presented with obscure gastrointestinal bleeding, unexplained abdominal pain, or suspected gastrointestinal stromal tumor were retrospectively included in the study. All patients had undergone capsule endoscopy (CE) followed by double-balloon enteroscopy (DBE) within a 3-month period. The endoscopic images were retrospectively evaluated by gastroenterologists, and the lesion detection rates of the 2 modalities were statistically compared.

**Results:** DBE has been used as the gold standard; overall, the sensitivity of CE for all lesions was 85.7%, and the specificity was 66.7%. CE was the most sensitive (100%) and most specific (91.3%) for polyps, but the number of polyps was very low (n = 3). It was sensitive (75% and 80%) and significantly specific (87.5% and 89.5%) for angioectasia and ulcers. However, diverticular lesions identified by DBE cannot be detected by CE.

**Conclusion:** CE offers significant advantages as a noninvasive modality for visualizing the small intestine, providing high safety and a comprehensive examination. However, certain limitations inherent to CE must be considered for accurate diagnosis. In this study, CE demonstrated high sensitivity and specificity in diagnosing various small bowel diseases; however, it was unreliable in excluding diverticula. Furthermore, these findings support the current clinical algorithm for managing indeterminate bleeding, which generally recommends observation after a negative CE.

## PS-103 Endoscopic Foreign Body Removal: A Single-Center Experience

**Güleycan Parça, Mustafa Mustafayev, Tarık Turan, Ali Şenkaya, Ozan Fatih Sarıkaya, Sevil Özer Sarı, Gözde Derviş Hakim**

Department of Gastroenterology, İzmir City Hospital, İzmir, Türkiye

**Background/Aims:** Foreign body (FB) impaction in the upper gastrointestinal system is one of the most common indications for emergency endoscopy, most frequently occurring in the esophagus and stomach. Endoscopic removal is the first-line treatment, with a high success rate and low risk of complications. This study aimed to evaluate the demographic features, localization, endoscopic techniques, and outcomes of patients who underwent endoscopic foreign body removal between 2024 and 2025 at this hospital.

**Materials and Methods:** A retrospective analysis was conducted on patients who presented with foreign body ingestion and underwent emergency endoscopy at the Gastroenterology Clinic, İzmir City Hospital, between January 2024 and September 2025. Patient demographics, localization, techniques used, and complications were recorded.

**Results:** A total of 39 patients were included. No foreign body was detected in 8 patients, whereas 31 had foreign bodies (24 males [77.4%], 7 females [22.6%]).- Localization: Esophagus 14 (45.2%), stomach 11 (35.5%), duodenum 2 (6.5%), rectum 4 (12.9%).- Techniques: Overtube + forceps in 13, snare in 15, pushing food into stomach in 2, polypectomy snare in 1.- Complications: 1 perforation (surgery required), 2 ulcerations, 1 case where FB cannot be removed due to ulceration (surgery required), 1 minor bleeding after removal.

**Conclusion:** Foreign bodies were most frequently localized in the esophagus and stomach. Some esophageal cases had underlying motility disorders or anatomical obstructions. Endoscopic techniques achieved high success rates. Although the complication rate was low, surgical intervention was required in cases with ulceration or perforation. Endoscopy is a safe and effective method for managing foreign bodies.

## PS-105 Recurrent Proximal Migration of a Biliary Stent in Caroli Disease Presenting with Cholangitis: A Case Report

**Şehmus Ölmez, Abdullah İlhan, Duran Deha Çetin, Mustafa Harı, Bünyamin Sarıtaş**

Department of Gastroenterology, Adana City Training and Research Hospital, Adana, Türkiye

Caroli’s disease (CD) is a rare congenital disorder characterized by segmental dilatation of the intrahepatic bile ducts, often leading to recurrent episodes of cholangitis. In patients with CD, endoscopic retrograde cholangiopancreatography (ERCP) may be required to remove biliary stones, dilate strictures, or place biliary stents. Here, a rare case of CD with recurrent proximally migrated biliary stents (PMBS) treated with stent removal and new stent insertions via ERCP is reported. A 43-year-old male patient, diagnosed with Caroli disease 7 years earlier, had previously undergone 3 ERCP procedures during which plastic biliary stents were placed in the common bile duct. He was admitted to the hospital with complaints of abdominal pain and chills over the past 4 days. Physical examination and laboratory values were consistent with cholangitis. Computed tomography and magnetic resonance imaging confirmed CD. Anti-biotherapy was initiated. ERCP revealed 2 PMBS, and both stents were removed using balloon extraction. During this procedure, purulent biliary drainage and millimetric stones were observed. A double pigtail stent was placed in the common bile duct, and a straight biliary stent was inserted into the right anterior hepatic duct. The patient recovered in 10 days. Written permission was obtained from the patient for this article. The definitive treatment for CD is liver transplantation. Since organ donation is very limited and the patient had no suitable living donors, he underwent recurrent ERCP sessions for cholangitis. Placement of biliary stents is one of the preferred treatment options for these patients. PMBS is a relatively rare complication of stent insertion but can lead to serious consequences, including cholangitis, liver abscess, and even death. Prompt removal of PMBS, or insertion of a new stent if removal is not possible, is essential to prevent complications. As far as the authors know, this is the first case of recurrent PMBS in a patient with CD treated with ERCP.


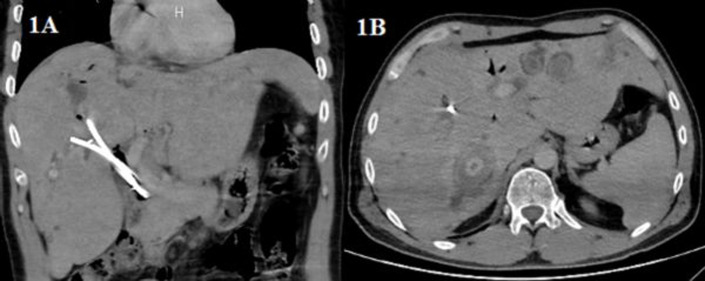


Figure 1. Computed tomographic images of a patient with Caroli disease: (1A) Coronal contrast-enhanced abdominal CT image demonstrating intrahepatic bile duct dilatations and the central dot sign consistent with Caroli disease and the presence of a proximally migrated biliary stent. (1B) Axial contrast-enhanced CT image showing multiple intrahepatic cystic dilatations and the central dot sign.


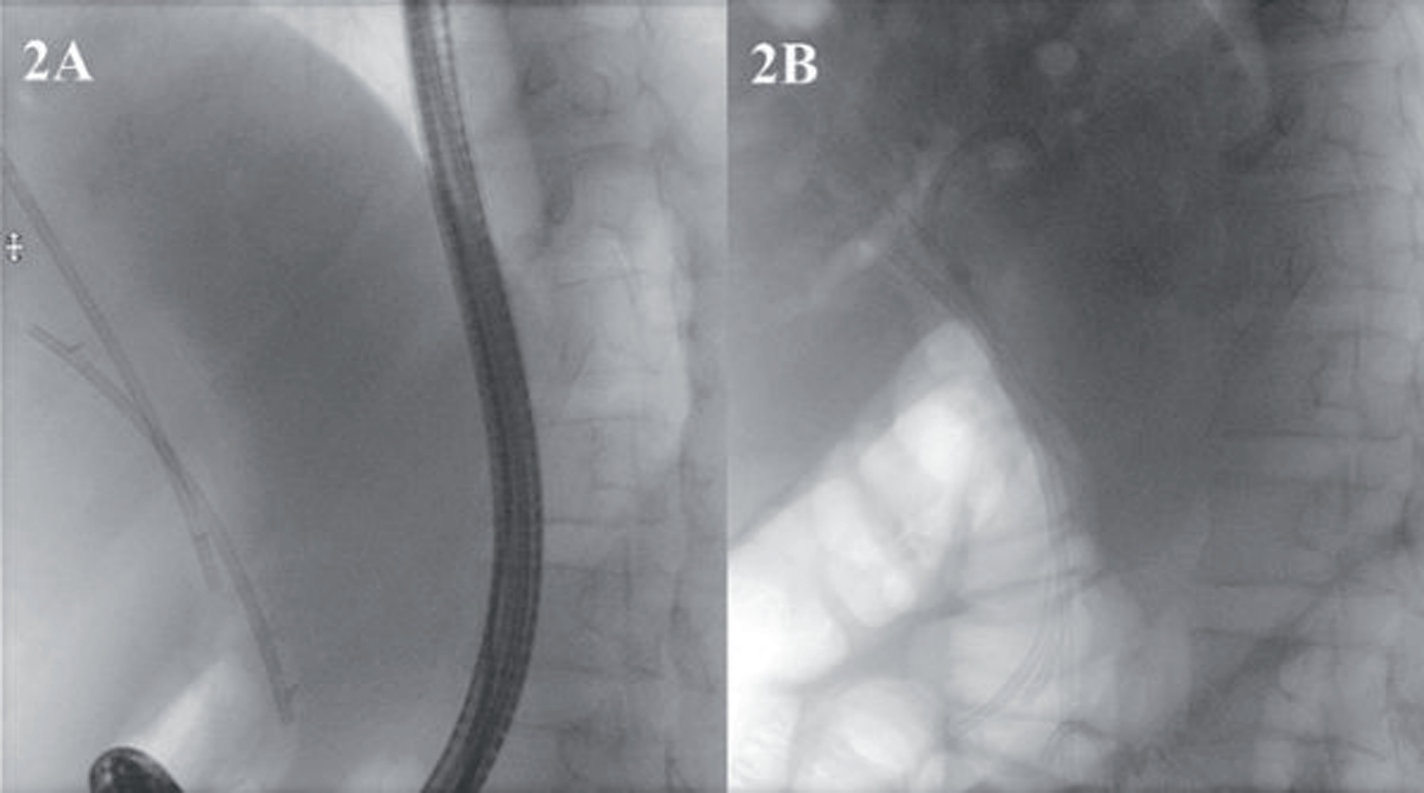


Figure 2. Fluoroscopic images showing 2 proximally migrated biliary stents within the common bile duct (2A) and the placement of new biliary stents (2 straight stents and 1 double pigtail stent) after retrieval of the migrated stents (2B).


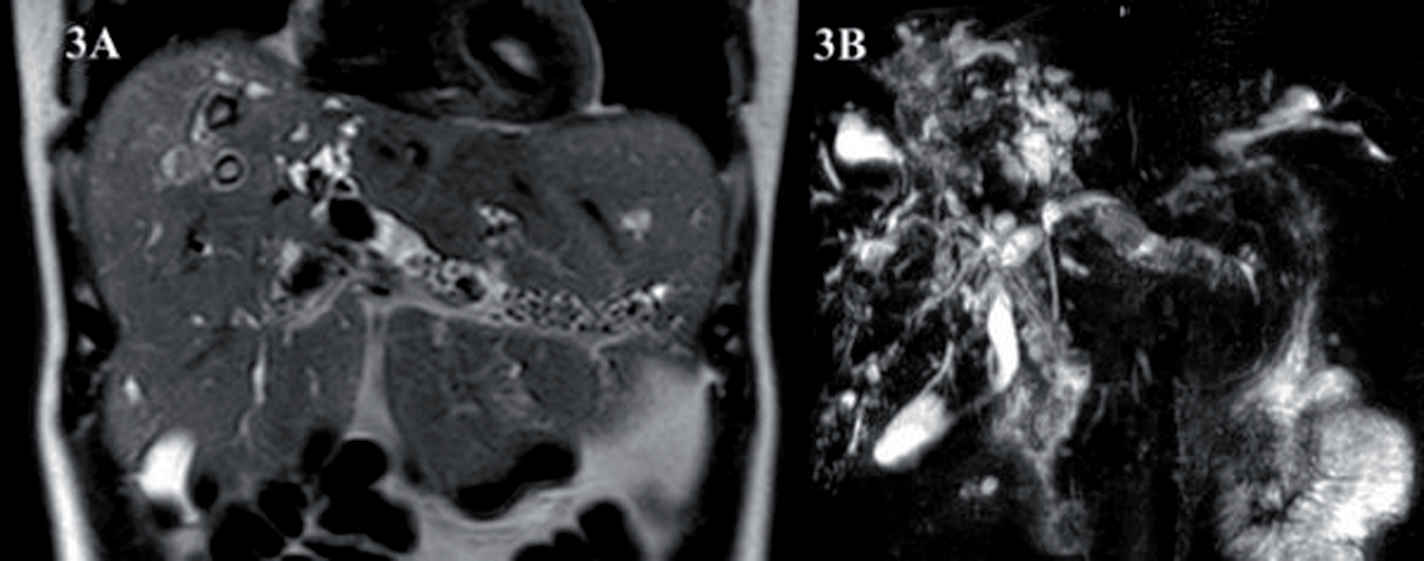


Figure 3. Coronal MR image showing multiple intrahepatic cystic dilatations and biliary stones with the characteristic “central dot sign,” consistent with Caroli disease (3A) and MR cholangiopancreatography (MRCP) image demonstrating segmental intrahepatic bile duct dilatations and communication with the biliary tree (3B).

## PS-106 Recurrent Unusual Foreign Body Ingestion in a Patient with Schizophrenia: Pen and Dessert Fork

**Fatime Demir, Abdullah İlhan, Şehmus Ölmez**

Department of Gastroenterology, University of Health Sciences, Adana Training and Research City Hospital, Adana, Türkiye

Foreign body ingestion (FBI) is rare in adults; however, it may occur recurrently, particularly in patients with psychiatric disorders, dementia, or among prisoners. A case of a patient with schizophrenia who ingested unusual foreign bodies (FB) twice within a short period is presented. A 58-year-old male patient was admitted to the emergency department after swallowing a pen. He had a prior history of schizophrenia. A radiopaque FB was detected in the upper abdomen on direct abdominal radiography and abdominal computed tomography. Emergency endoscopy was performed, revealing a pen approximately 130 mm in length within the stomach, which was successfully removed endoscopically using a snare. Two months later, the patient was admitted to the emergency department due to the ingestion of a fork. Direct radiography demonstrated the presence of a fork within the stomach. The patient underwent emergency endoscopy, which revealed a fork in the stomach. After gastric insufflation, the fork was grasped with a snare, rotated within the stomach, and subsequently removed with the snare.

People with schizophrenia are at increased risk of repeated FBI. Management of these patients must be done in collaboration with psychiatrists. Endoscopy is the first therapeutic approach in the management of FBI. The majority of foreign bodies in the upper gastrointestinal tract (>95%) can be successfully removed with endoscopic methods, and the complication rate is very low. The European Society of Gastrointestinal Endoscopy guidelines recommend urgent therapeutic endoscopy for sharp objects, magnets, batteries, and large/long objects in the stomach. In the present patient, endoscopy was performed within 24 hours because the first foreign body was long (about 13 cm) and the next foreign body was sharp-pointed and long. Both the pen and the fork were successfully removed endoscopically without complications. Endoscopy is the most effective and safest method for managing FBI in the upper gastrointestinal tract.


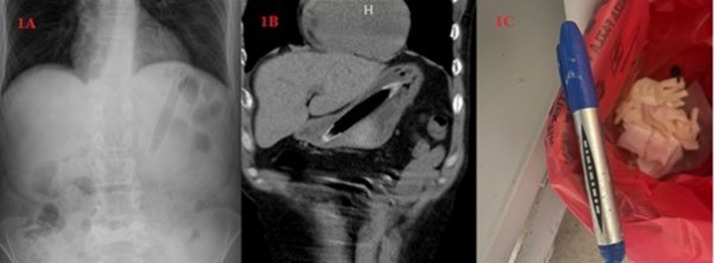


Figure 1. Images of the ingested pen: direct radiography (1A), coronal abdominal computed tomography (1B), and postendoscopic removal view (1C).


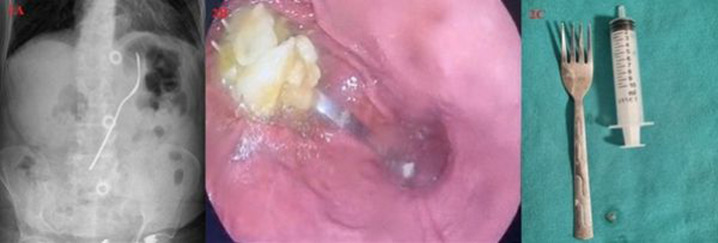


Figure 2. Images of the ingested fork: direct radiography (2A), endoscopic view (2B), and postendoscopic removal view (2C).

## PS-108 A Rare Etiology of Upper Gastrointestinal Bleeding: Cameron Lesion

**Aziz Aslan, Selçuk Turgut, Said Kocakaya, Ceren Cennet Alimoğlu, Hatice Çilem Solak, Mesut Aydın**

Department of Gastroenterology, University of Health Sciences İzmir Faculty of Medicine, Tepecik Training and Research Hospital, İzmir, Türkiye

Gastrointestinal (GI) bleeding is a major cause of morbidity and mortality in the elderly. A rare etiology is Cameron lesions, linear ulcerations within the hiatal hernia pouch, usually linked to chronic iron deficiency anemia but occasionally presenting with acute bleeding. An 82-year-old female patient was admitted to the emergency department with acute-onset hematemesis and melena. Her past medical history included hypertension, coronary artery disease, hyperthyroidism, and orthopedic platinum implantation. Medication history revealed the use of low-dose NSAIDs at least 3 times per week and administration of systemic steroids 2 days prior to admission for back pain. There was no history of alcohol consumption, viral hepatitis, or oral iron supplementation. On admission, the patient’s vital signs were stable. Laboratory findings revealed hemoglobin: 8.7 g/dL, urea: 111 mg/dL, and creatinine: 1.4 mg/dL. Intravenous proton pump inhibitor therapy and hydration were initiated, and 2 units of packed red blood cells were transfused. Upper gastrointestinal endoscopy demonstrated a 5-cm hiatal hernia pouch containing linear erosions with ulcerated surfaces and clean bases (Cameron lesions). The esophageal, gastric corpus, antral, and duodenal mucosa appeared normal. During follow-up, the patient’s hemoglobin level increased to 11.7 g/dL, and she remained clinically stable. She was discharged with recommendations for outpatient follow-up at gastroenterology and nephrology clinics. Cameron lesions usually cause chronic anemia but may rarely present with acute bleeding. NSAIDs and corticosteroids increase the risk of mucosal injury and bleeding. Endoscopic evaluation is essential, particularly in elderly, comorbid patients. Cameron lesions, though rare, are an important cause of upper GI bleeding and should be considered in patients with hiatal hernia presenting with unexplained anemia or acute bleeding.


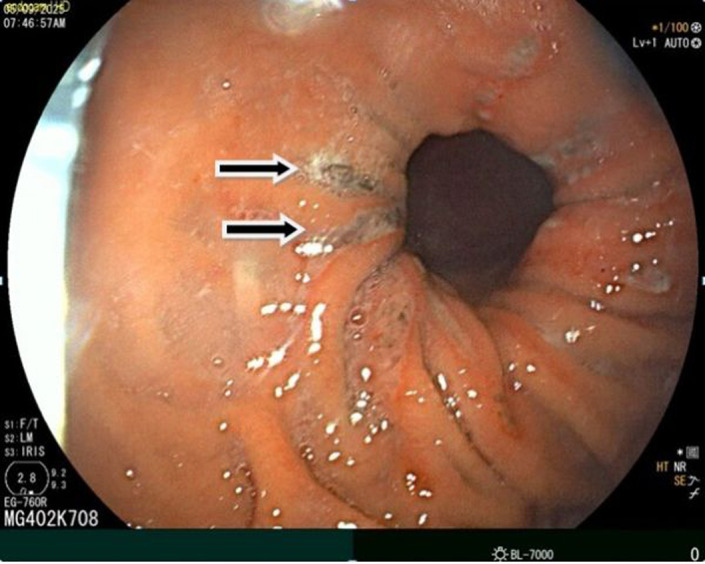


Figure 1. Linear erosion at the gastroesophageal junction on endoscopy, compatible with Cameron lesion.

## PS-111 Selection of Eligible Alzheimer Patients for Percutaneous Endoscopic Gastrostomy Using Ultrasonography-Proven Nonalcoholic Fatty Liver Disease: A New Tool for Making a Decision for PEG Procedure. Black Sea PEG Rule

**Ahmet Cumhur Dülger, Gökhan Aydın, Eray Beşirli**

Division of Gastroenterology, Giresun University School of Medicine, Giresun, Türkiye

**Background/Aim:** In Alzheimer (ALZD) patients who are also candidates for PEG procedures due to the prevention of malnutrition, neurologists often play a key role in determining whether the PEG procedure is needed. However, there is no data on whether gastroenterologists fully decide on that procedure. Thus, a comprehensive ultrasonographic examination was conducted in connection with other laboratory parameters involving fatty liver disease in patients with ALZD.

**Materials and Methods:** Between October 2021 and October 2025, a total of 159 ALZD patients (82 females; 51.6%, mean age 84 years) who underwent PEG procedures at Giresun University School of Medicine-affiliated Aksu State Hospital were enrolled and further evaluated regarding ultrasonographic findings of the liver in connection with other relevant laboratory data linked to hepatosteatosis. Data were statistically analyzed using SPSS version 25.0 statistical software. Categorical variables were compared using the chi-square test. *P* < .05 values were considered statistically significant.

**Results: **The mean levels of AST and ALT were 71 ± 212 U/L (normal: 0-32; CI: 38-105) and 43 ± 127 U/L (normal: 0-33, CI: 23-63). In addition, the mean levels of ALP and GGT were as follows: 137 ± 108 (normal: 40-135, CI: 105-169) and 57±80 (normal: 10-60, CI: 35-80). From a radiologic perspective, the absence of NAFLD was noted in 89 patients (56%), grade 1 NAFLD was present in 51 patients (32.1%) grade 2 NAFLD was present in 11 patients (6.9%), and grade 3 NAFLD was present in 6 patients (3.8%).

**Conclusion: **The lower rates of ultrasonographically detected moderate or advanced NAFLD in patients who underwent the PEG procedure were the key findings of this study. Furthermore, normal levels of cholestatic enzymes and near-normal ALT levels were significant laboratory findings that also indicated a lack of biochemical NAFLD. The absence of NAFLD in those with malnutrition can serve as a key marker for selecting patients with ALZD.

## PS-112 A Rare Esophageal Pigmentation: Esophageal Melanocytosis

**Tuba Durukan ^1^ , Nejla Küçük ^2^ , Etibar Mammadov ^2^ , Mehmet Kapan ^2^ , Hasan Utku Kocal ^2^ , Murat Başaran ^2^ , Mehmet Suat Yalçın ^2^ , Burak Özşeker ^2^ , Melek Ünçel ^3^**

^1^Department of Internal Medicine, Muğla Training and Research Hospital, Muğla, Türkiye

^2^Gastroenterology Clinic, Muğla Training and Research Hospital, Muğla, Türkiye

^3^Department of Medical Pathology, Muğla Training and Research Hospital, Muğla, Türkiye

Esophageal melanocytosis is a rare benign pathological condition characterized by melanocytic proliferation in the basal layer of the squamous epithelium of the esophagus and melanin accumulation in the mucosa. Although it is thought to be a precursor to primary esophageal melanoma, little is known about its etiology and course. A rare case of esophageal melanocytosis confirmed by histopathological examination is presented.


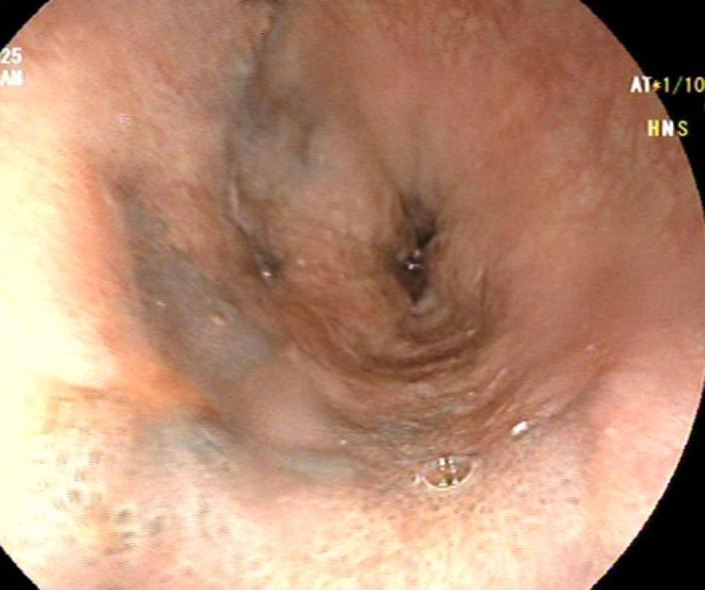


Figure 1. Increased pigmentation in the esophagus during upper GI endoscopy.


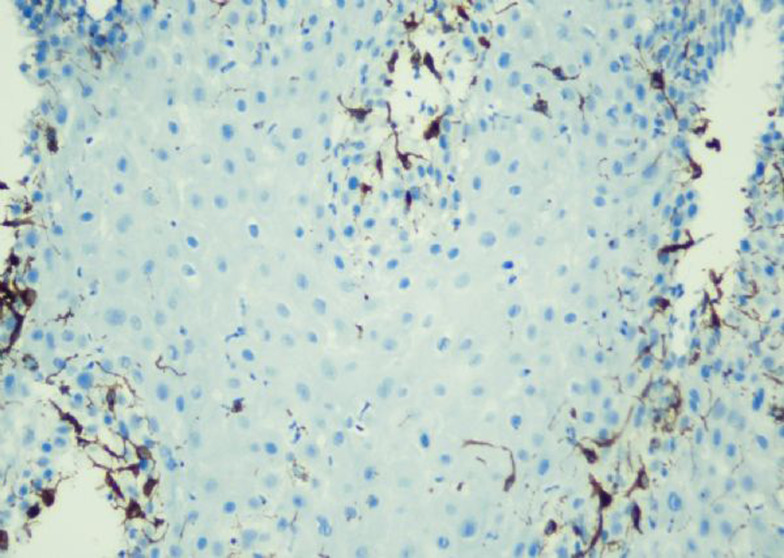


Figure 2. Histopathological image of melanocytes with large cytoplasm containing melanin in the cytoplasm.

## PS-113 Can Cephalexin Cause Acute Pancreatitis in a Patient with Pancreas Divisum?

**Ahmet Emre Ergan ^1^ , Osman Özdoğan ^1^ , Feramuz Demir Apaydın ^2^ , Mammadhasan Mammadov ^1^ , Ümit Yeşilova ^1^ , Oktay Bayraktar ^1^ , Mehmet Kasım Aydın ^1^ , Serkan Yaraş ^1^ , Fehmi Ateş ^1^ , Engin Altıntaş ^1^ , Orhan Sezgin ^1^**

^1^Department of Gastroenterology, Mersin University Faculty of Medicine, Mersin, Türkiye

^2^Department of Radiology, Mersin University Faculty of Medicine, Mersin, Türkiye

Acute pancreatitis (AP) is a common gastrointestinal admission to the emergency department, most often caused by gallstones or alcohol consumption. Drug-induced AP is rare (∼2%) and difficult to differentiate from idiopathic cases. This presentation discusses a case of AP in a young woman with underlying pancreas divisum (PD), where cephalexin is implicated as the causative agent. A 35-year-old woman presented to the emergency department with severe epigastric pain radiating in a band-like pattern. Laboratory tests showed significantly increased amylase (2754 U/L) and lipase (8363 U/L) levels. Liver enzymes, bilirubin, calcium, and triglycerides were normal. She had no history of alcohol abuse or prior pancreatitis attacks. Her medical history was unremarkable except for 3 days of cephalexin use for a urinary tract infection. CT and ultrasound confirmed AP with an edematous pancreas, peripancreatic fluid, and increased fat stranding; there were no findings in the gallbladder or common bile duct. IgG and IgG4 levels, tested to rule out autoimmune etiologies, were within normal limits. MRCP reported “Visualization of the dorsal pancreatic duct coursing anterior to the common bile duct and draining into the duodenum cranial to the ampulla. A separate ventral pancreatic duct system communicating with the dorsal duct was not identified. These findings are suggestive of pancreas divisum.” An ERCP procedure for minor papilla sphincteroplasty and stenting was planned. Pancreas divisum, the most common congenital pancreatic anomaly, is considered an AP risk factor due to relative obstruction at the minor papilla. Although cephalexin is a rare cause of AP, the exclusion of other major etiologies suggests a drug-induced cause in this patient. This case proposes that an anatomical predisposition like PD may have reduced the threshold for cephalexin-induced AP development. Rare drug etiologies should be considered in the differential diagnosis of idiopathic pancreatitis, especially when an underlying anatomical variant like PD is present.


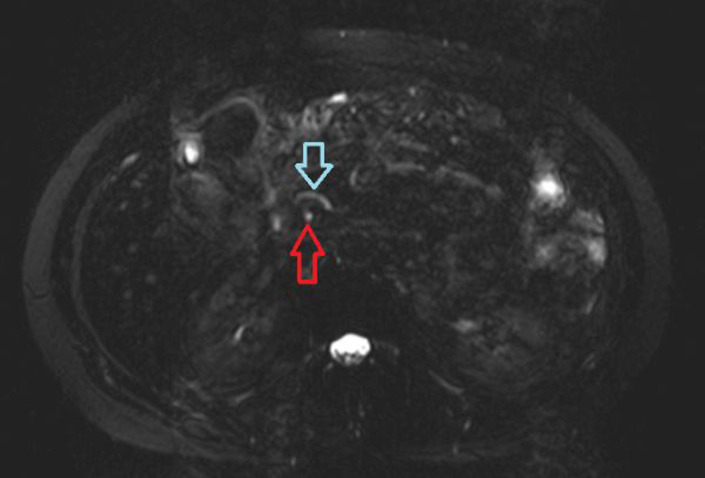


Figure 1. Visualization of the common bile duct (red) draining into the major papilla and the main pancreatic duct (blue) draining into the minor papilla, consistent with pancreas divisum on the cholangiographic phase of MRCP.

## PS-114 A Useful Endoscopic Description of Borderline Ischemia in Sigmoid Volvulus: Fallen Leaves in Autumn

**Ömer Küçükdemirci**

Department of Gastroenterology, Hakkari State Hospital, Hakkari, Türkiye

Sigmoid volvulus (SV) is an uncommon cause of intestinal obstruction resulting from axial twisting of the sigmoid colon around its mesenteric base. In the absence of complications, endoscopic detorsion is considered the first-line treatment, whereas urgent surgery is required in cases of perforation, peritonitis, or failed detorsion (1). The “coffee bean sign” (massively dilated sigmoid loop directed toward the right upper quadrant) and the “whirl sign” (twisting of mesentery and vessels) are well-established radiological findings of SV (2). By contrast, endoscopic features are less well characterized, and evaluating mucosal ischemia or necrosis endoscopically remains challenging. Completely black mucosa indicates full-thickness necrosis, whereas borderline ischemia may present with variably discolored mucosa, including yellowish and reddish spots. In 2021, Uysal et al described this finding as the “fallen leaves in autumn” sign, reflecting superficial necrosis with preserved viability of deeper layers. The case of an 83-year-old man admitted to the emergency department with abdominal pain and rectal bleeding is reported. Imaging revealed classic signs of SV. During endoscopic detorsion, findings consistent with borderline ischemia were noted: loss of submucosal vascularity and scattered reddish-yellowish dots, some confluent. Areas suspected of complete necrosis displayed black mucosa with a sandpaper-like appearance. The patient underwent surgery for irreversible ischemia but unfortunately died a few days later due to postoperative complications. This case highlights that the “fallen leaves in autumn” sign may serve as a useful endoscopic descriptor of borderline ischemia in SV, complementing established radiological features.


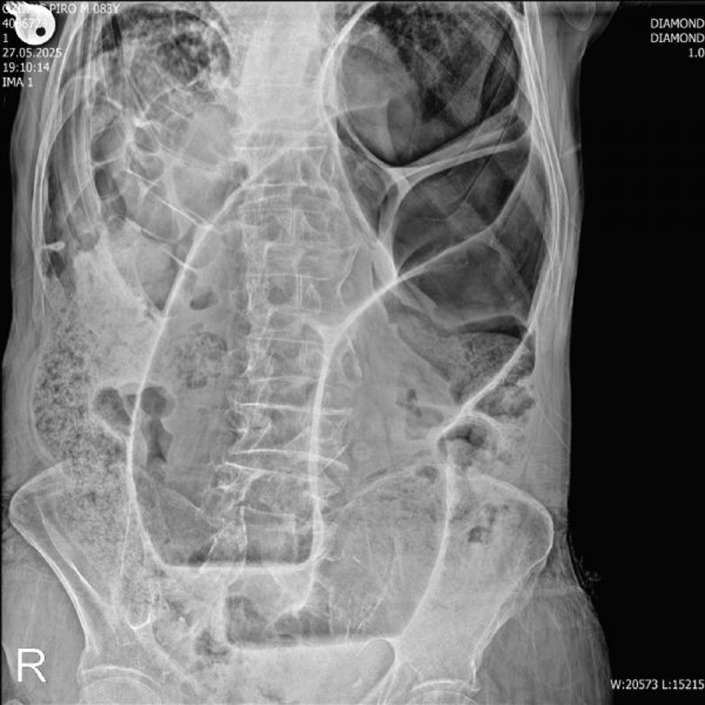


Figure 1. Coffee bean sign


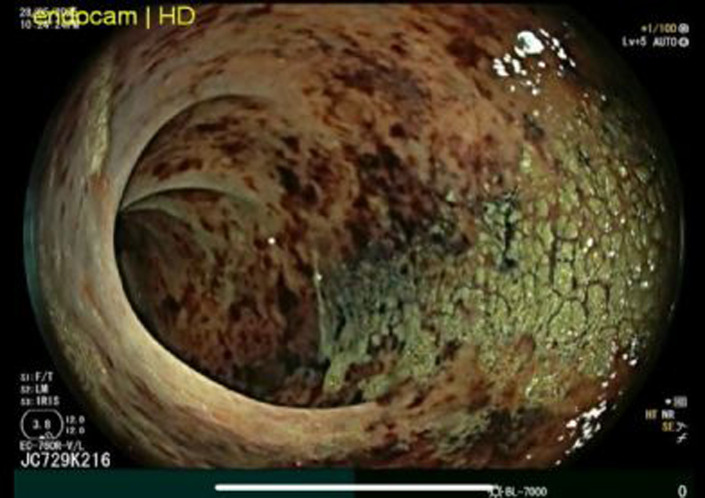


Figure 2. Fallen leaves in autumn sign.


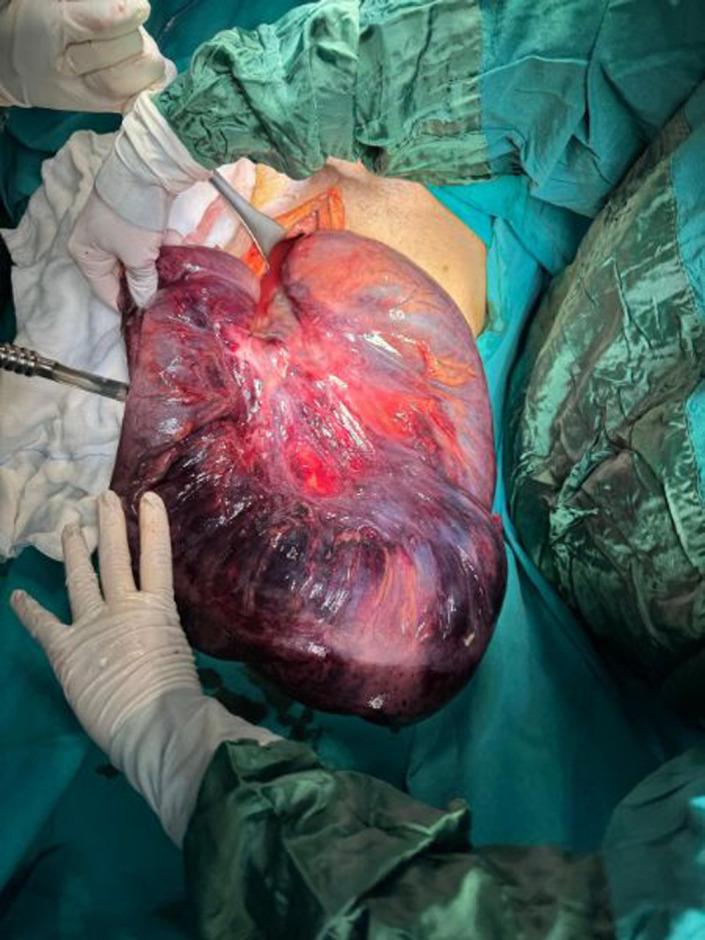


Figure 3. XXX.

## PS-115 Removal of a Large Common Bile Duct Stone by Endoscopic Retrograde Cholangiopancreatography in a Patient with Upside-Down Stomach

**Şehmus Ölmez, Fatime Demir, Hasan Selim Güler**

Department of Gastroenterology, Adana City Training and Research Hospital, Health Sciences University, Adana, Türkiye

Endoscopic retrograde cholangiopancreatography (ERCP) is a crucial therapeutic modality used in the management of patients with choledocholithiasis. The extraction of large common bile duct stones (LCBDS) (>15 mm) involves technical challenges. Upside-down stomach (UDS) is the rarest type of hiatal hernia, accounting for less than 5% of cases, and is characterized by the herniation of the entire stomach or most of the stomach into the posterior mediastinum. In these patients, access to the papilla is technically difficult because it is challenging to pass the duodenoscope through the pylorus. Here, a rare case of UDS is presented, in which an LCBDS (>2 cm) was successfully removed with ERCP. A 70-year-old female patient was admitted with complaints of abdominal pain. Her past medical history included diabetes mellitus and asthma. On physical examination, there was tenderness in the right upper quadrant. Laboratory examination revealed WBC 12.9 × 10^3^/μL, total bilirubin 6.1 mg/dL, direct bilirubin 3.4 mg/dL, AST 58 U/L, ALT 50 U/L, alkaline phosphatase 146 U/L, gamma-glutamyl transferase 587 U/L, and CRP 168 mg/L. Computed tomography showed bile duct dilatation, LCBDS, and UDS. ERCP was performed early, and the ampulla can hardly be reached with the duodenoscope. The papilla was successfully cannulated, and fluoroscopy followed by contrast agent administration showed LCBDS and bile duct dilatation. Following endoscopic sphincterotomy, the common bile duct stone was successfully removed using a balloon after dilatation with a standard stone removal balloon. Written informed consent was obtained from the patient’s relatives for this letter. Very few cases of UDS have been reported in the literature involving ERCP. Despite the significant technical challenges posed by the combination of LCBDS and UDS, ERCP was successfully performed in the current case, and the LCBDS was successfully removed through balloon dilatation and extraction. It is believed that successful ERCP in a patient with UDS and LCBDS is clinically significant.


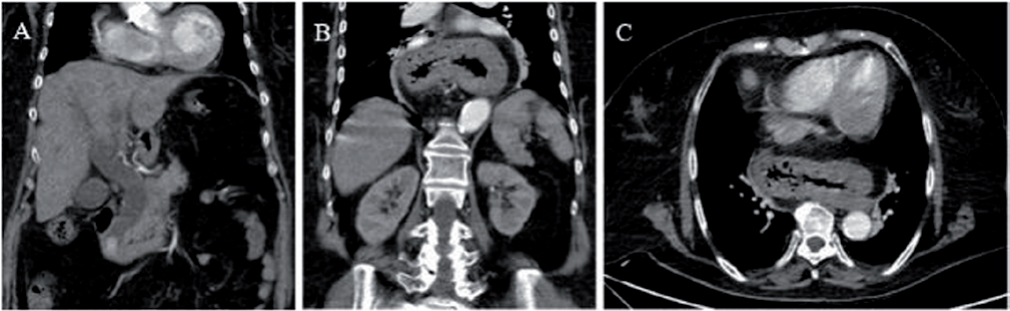


Figure 1. Computed tomography images showing dilatation of the bile duct with a distal common bile duct stone (A), and coronal (C) and transverse (B) views of the stomach herniated into the mediastinum.


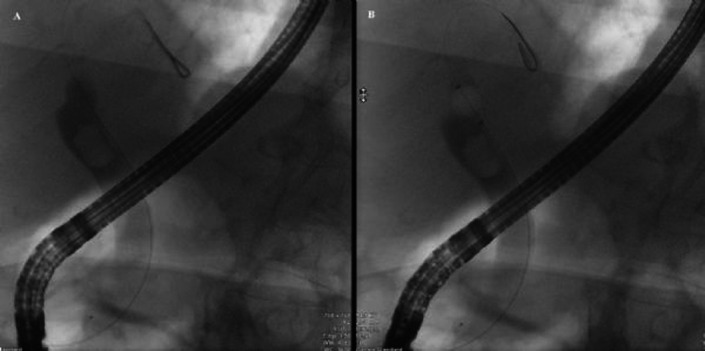


Figure 2. Fluoroscopy images showing a large common bile duct stone (20 × 10 mm) with biliary dilatation (A), and the application of endoscopic balloon extraction (B).


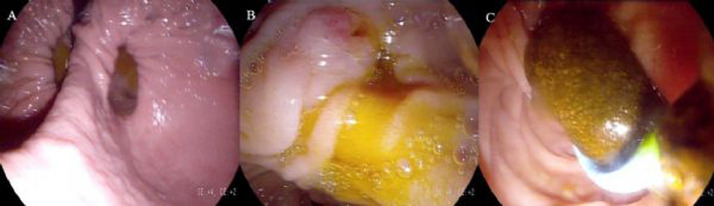


Figure 3. Endoscopic views in a patient with upside-down stomach: gastric views (A), visualization of the papilla (C), and extraction of a large stone using balloon sweeping (D).

## PS-117 Oral Squamous Cell Carcinoma Developing after Immunosuppressive Therapy in a Patient with Crohn’s Disease

**Kamil Enli, Halil Yılmaz, Yekta Duygu Çimen Beşirli, Mustafa Yılmaz**

Department of Gastroenterology, Pamukkale University Faculty of Medicine, Denizli, Türkiye

The risk of malignancy increases in Crohn’s disease, especially during long-term immunosuppressive therapy with thiopurines and anti-TNF agents. This increase is primarily associated with lymphoma and skin cancers. Oral squamous cell carcinoma (SCC) is extremely rare, and its diagnosis is often delayed. Here, the case of oral SCC developing in a patient with long-standing Crohn’s disease is presented. A 46-year-old male patient had been diagnosed with Crohn’s disease since 2006. He was hospitalized twice in June and July 2024 due to abdominal pain and episodes of diarrhea. Since there was no response to mesalazine and azathioprine therapies, infliximab was initiated, and a total of 9 doses were administered. In September 2024, after the third dose of infliximab, follow-up colonoscopy revealed Crohn’s disease in remission and normal oral mucosa. In February 2025, the patient presented with a 3-month history of a nonhealing aphthous-like lesion on the left buccal mucosa. He was referred to the ENT department for evaluation of possible malignancy. A punch biopsy revealed SCC, and infliximab therapy was discontinued. PET-CT showed metastatic lymph nodes in the left submandibular and submental regions. The oral lesion developed approximately 7 months after starting anti-TNF therapy, and the diagnosis was made in the tenth month. Oral lesions developing during long-term immunosuppression in Crohn’s disease should be carefully evaluated. Nonhealing aphthous ulcers must be investigated for malignancy at an early stage. This case highlights that, although rare, oral SCC may develop in such patients, and clinicians should remain vigilant.


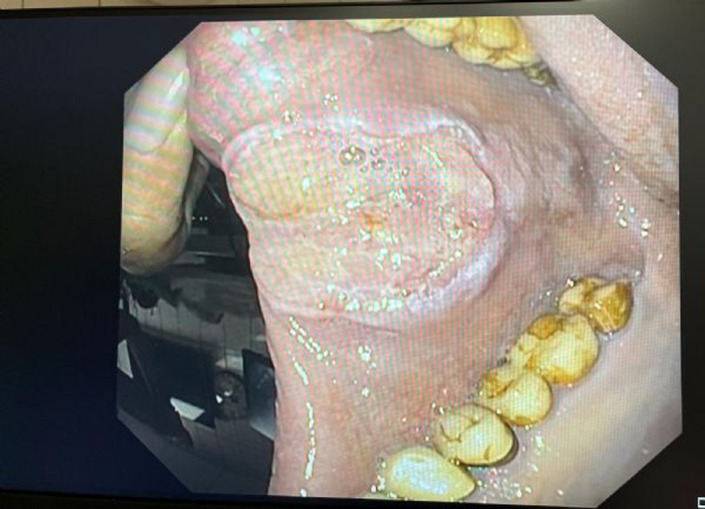


Figure 1. Ulcerated lesion on the left buccal mucosa.

## PS-118 A Rare Clinical Presentation of Hemochromatosis Associated with Heterozygous HFE H63D Mutation: Acute Pancreatitis

**Haluk Cihad Albayrak, Bora Aktaş, Sanlı Arabacı, Mehmet Raşit Ayte, Seçkin Özgül, Evrim Kahramanoğlu Aksoy**

Ankara Atatürk Sanatorium Training and Research Hospital, Ankara, Türkiye

Hereditary hemochromatosis (HH) is characterized by systemic iron overload due to HFE gene mutations, with pancreatic involvement being extremely rare. Acute pancreatitis (AP) is typically associated with C282Y homozygous or compound heterozygous mutations, whereas data regarding heterozygous H63D variants are limited. A 59-year-old man, with a history of cholecystectomy 10 years prior and no history of alcohol consumption, presented with sudden onset of epigastric pain. Serum lipase was 750 U/L, ferritin 1657 ng/mL, and transferrin saturation 60%. Magnetic resonance relaxometry confirmed mild hepatic iron overload (LIC 1.9 mg Fe/g; T2 × 17 milliseconds). HFE genotyping revealed a heterozygous H63D mutation. Biliary obstruction, hypertriglyceridemia, hypercalcemia, and drug-induced pancreatitis were excluded. The pancreatitis resolved with supportive care, and a regular phlebotomy regimen was initiated to reduce iron overload. H63D heterozygous carriers may also develop clinically significant iron overload, which can precipitate acute pancreatitis. In cases of idiopathic pancreatitis, iron parameters, HFE genotyping, and MR relaxometry should be considered in the diagnostic workup.

## PS-119 Concurrent Cholecystitis and Predisposing Factors in Patients with Acute Pancreatitis

**Yüsüf Uğur, Fatma Yılmaz Öncül, Berat Ebik, Nazım Ekin, Muhsin Kaya**

Department of Gastroenterology, Dicle University Faculty of Medicine, Diyarbakır, Türkiye

**Background/Aims:** Gallstones are the most common cause of acute pancreatitis (AP) and acute cholecystitis, and the symptoms and signs of these 2 diseases can be confused. This study aimed to determine the frequency of concurrent acute cholecystitis in patients with acute pancreatitis and identify the risk factors affecting its development.

**Materials and Methods:** One hundred fifty patients diagnosed with AP were prospectively studied between May 2024 and May 2025. Demographic data, clinical characteristics, and laboratory findings at the time of admission were recorded. All patients underwent abdominal ultrasonography performed by the same physician.

**Results:** The mean age of the patients was 56.3±18.4 years; 86 (57.3%) were female and 64 (42.7%) were male. The hospital stay averaged 5.9±4.2 days. Of the 150 patients diagnosed with AP, biliary causes were the most common etiological factor in 97 (64.7%). Other etiological factors included idiopathic/other causes in 29 (19.3%) patients, hyperlipidemia in 13 (8.7%), alcohol use in 7 (4.6%), and post-ERCP in 4 (2.6%). Among concurrent biliary diseases, acute cholecystitis was the most common, occurring in 41 (27.3%) patients. All patients with cholecystitis had gallstones. The presence of biliary pancreatitis was found to be the strongest independent risk factor for the development of acute cholecystitis in both single and multiple analyses (OR: 4.52, *P* = .001). Low calcium levels (*P* = .037) and hypertriglyceridemia (*P* = .049) were also significant risk factors for the development of acute cholecystitis in patients with AP; however, these parameters were not identified as independent risk factors in multiple analyses. The most common comorbidities were diabetes in 52 (34.7%) patients and hypertension in 38 (25.3%) patients.

**Conclusion:** The presence of biliary pancreatitis is an independent risk factor for the development of concurrent cholecystitis. Patients with pancreatitis due to biliary causes should be evaluated more carefully for the development of acute cholecystitis.

## PS-121 The Prevalence of Colonic Diseases in Very Elderly Patients Aged 90 Years and Older

**Gökhan Aydın ^1^ , Ahmet Cumhur Dülger ^1^ , İsmail Aydın ^2^**

^1^ Division of Gastroenterology, Giresun University School of Medicine, Giresun, Türkiye

^2^ Division of General Surgery, Giresun University School of Medicine, Giresun, Türkiye

**Background/Aims: **The rate of very elderly patients is slightly above normal ranges, particularly in the coastal areas of the eastern Black Sea region in Türkiye. Most of them have suffered from gastrointestinal diseases and required colonoscopy despite their low health status, which can be associated with mortality.

**Materials and Methods: **Between October 2021 and October 2025, a total of 215 very elderly patients (72% females, mean age 97 years) who underwent colonoscopy procedures at Giresun University School Hospital were enrolled and further evaluated regarding colonoscopic findings in connection with other relevant laboratory data. Data obtained in this study were statistically analyzed using SPSS version 25.0 statistical software. Categorical variables were compared using the chi-square test.

**Results: **Study patients mostly came from internal medicine wards (29%) and intensive care units (23%) compared to younger (<90 years) counterparts (*P* < .01). There were no reported deaths during colonoscopy. Two patients had perforations. From the colonoscopic perspective, 15% had solitary rectal ulcers (SRU), 19% had pan-colonic diverticula, 32% had segmentary diverticula, 17.2% had colonic adenoma, and 3.25% had colonic adenocarcinoma. From an anesthesiologic point of view, the length of stay was longer for patients over 90 years (*P* = .043). The mean propofol dose was lower for younger patients undergoing colonoscopy (*P* = .01), and lower propofol doses were associated with higher AST levels in the study group (*P* = .037). The mean laboratory values were as follows: WBC: 11.800/mm^3^, Hb: 9.2 g/dL, PT: 14.6, PLT: 247.000/mm^3^, lactic acid: 3.2 mmol/L, creatinine: 1.3 mg/dL, albumin: 2.2 mg/dL, CRP: 82 u/L.

**Conclusion: **The rate of colon cancer was very low, whereas the rates of pan-colonic diverticula and SRU were very high in the study group. Most of the study patients had hyperlactatemia and hypoalbuminemia. Lower rates of mortality during examination were also noted.

## PS-122 Blister-Coated Medication Ingestion as a Cause of Esophageal Foreign Body in Elderly Patients: Two Case Reports

**Tolga Yıldırım, Deniz Armağan Deniz**

Department of Gastroenterology, Etlik City Hospital, Ankara, Türkiye

Esophageal impaction of foreign bodies or food is a common cause of emergency visits. In adults, true foreign body ingestion is usually seen in psychiatric patients, those with alcohol intoxication, the elderly, and prisoners seeking secondary gain (1). Sharp blister edges may cause esophageal injury, perforation, and mediastinitis. Reported outcomes include dysphagia, ulceration, perforation, mediastinitis, and death. Here, 2 elderly cases managed in the unit within 1 week are presented. Case 1 was an 83-year-old man undergoing endoscopy for iron deficiency anemia who was incidentally found to have a blister pack lodged in the esophagus. It was detached, advanced to the stomach, and removed with an overtube. Superficial abrasions were seen in the mid and distal esophagus, with no deep laceration or perforation. The patient was unaware of the ingestion. Case 2 was a 71-year-old woman who accidentally swallowed a tablet with its blister and presented with retrosternal pain. Endoscopy revealed a free blister pack in the lumen, which was removed uneventfully with a net snare. Only minimal proximal esophageal abrasions were noted, and symptoms resolved after the procedure. Although rare, blister ingestion may cause severe complications. In elderly patients, neurological disease, cognitive decline, and polypharmacy increase the risk. Thus, this possibility should be considered in those presenting with dysphagia or retrosternal pain. Education of caregivers, dispensing tablets without blisters, and safer packaging may prevent such events. Otherwise, laceration, bleeding, perforation, mediastinitis, and even death may occur.


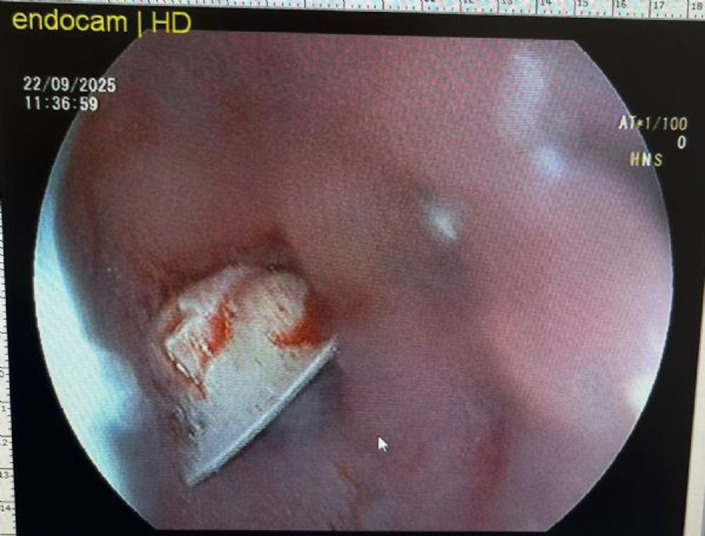


Figure 1. Endoscopic image of the foreign body stuck in the esophageal mucosa of Case 1.


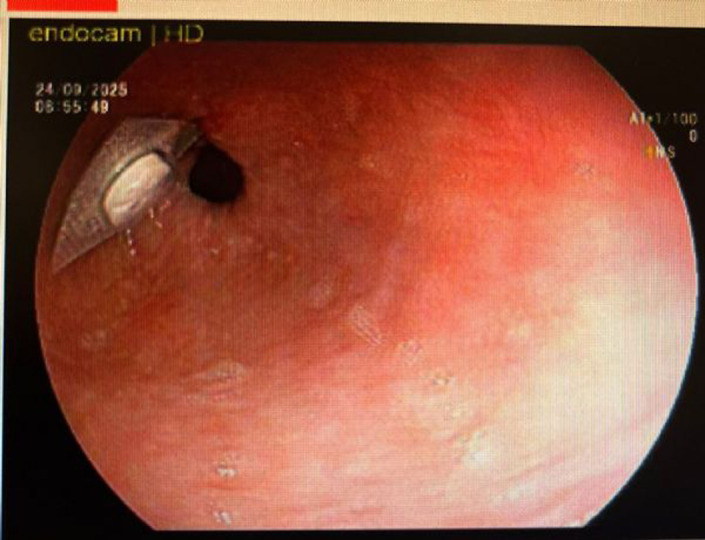


Figure 2. Endoscopic image of the esophageal foreign body in Case 2.

## PS-123 Fistula Healing with Antiviral Treatment in a Patient with HIV+ Ileal Fistula

**Civanmert Bayrak, Süleyman Dolu, Mesut Şan, Rauf Mehtiyev, Arzu Nazlı Zeka**

Department of Gastroenterology, Dokuz Eylül University Faculty of Medicine, İzmir, Türkiye

A 60-year-old man with no comorbidities presented to the gastroenterology clinic due to periumbilical abdominal pain and a 20 kg weight loss lasting 6 months.

The patient’s evaluations at an external center were as follows:

- Endoscopy: Hiatal hernia, antral gastritis
- Pathology: No chronic active gastritis or malignancy/dysplasia
- Colonoscopy: Calcified nodule in the colon
- Pathology: No nonspecific colitis, malignancy, atypia, or specific pathological findings.
- CT: Irregular thickening of the terminal ileum wall, increased contrast enhancement, luminal dilatation, increased heterogeneous density in the peri-ileal fat planes, and iliocecal mesenteric lymphadenopathy (LAPs).

Evaluations at the center were as follows:

- CT: Increased wall thickness at the cecum, ascending colon, and terminal ileum, heterogeneity in the surrounding fatty tissue, and prominent vascular structures; suggestive of inflammatory bowel disease
- Colonoscopy: The terminal ileum was advanced to the distal 20 cm. The ileal mucosa up to this area was hyperemic and edematous. Two bowel loops (fistulas) were observed in the distal ileum. There was stenosis in this area that allowed the scope to pass. The ileocecal valve was edematous. All colonic mucosa observed retrograde from the cecum was edematous. The anal canal was normal upon inversion.
- Endoscopic Diagnosis: Ileal fistula tract (Crohn’s disease?) + bx
- Pathology: Diagnosis 1: Active ileitis
- Erosion, ulceration, mucosal necrosis: absent
- Crypt Structure: Epithelial inflammation, crypt abscess, superficial crypt loss, necrosis, distortion, reactive mucosal atypia, dysplasia, and Paneth cell metaplasia, apoptotic activity: absent
- Lamina propria: Inflammation: present + Plasma cell: present, within normal limits. Edema: present ++ Fibrosis: absent

After tests ordered for chronic diarrhea revealed HIV Ag+, an HIV-RNA test was performed. Upon the positive result, bictegravir sodium, emtricitabine, and tenofovir alafenamide were started by the infectious diseases department.

A follow-up colonoscopy performed after approximately 1 year after treatment was reported as normal. The patient was recommended for annual follow-up.


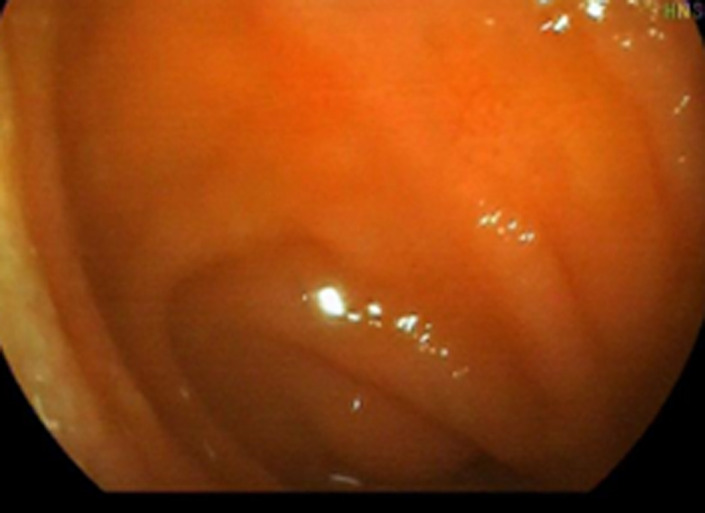


Figure 1. Posttreatment ileum.


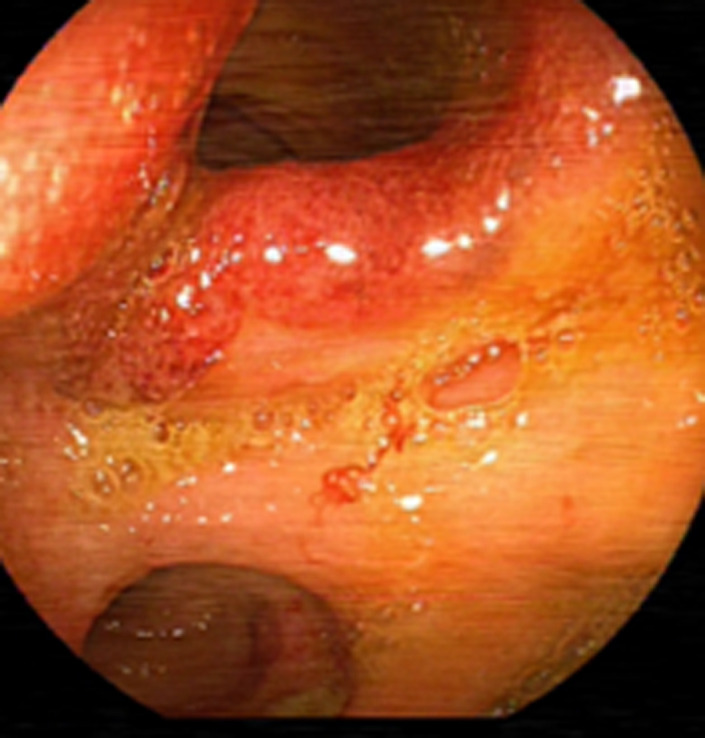


Figure 2. Fistula in the ileum - before treatment.


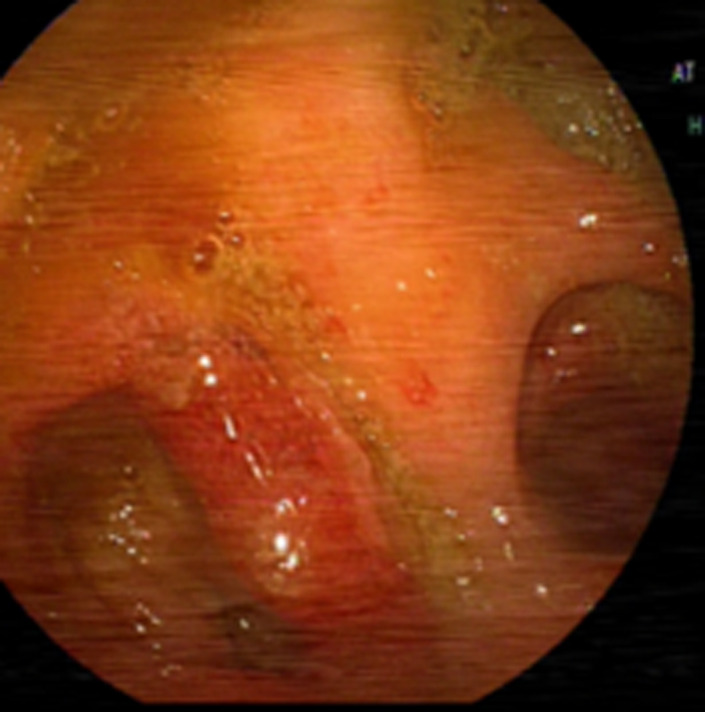


Figure 3. Fistula in the ileum - before treatment

## PS-124 A Rare Cause of Upper Gastrointestinal Hemorrhage: Gastric Invasion of Retroperitoneal Liposarcoma

**Şehmus Ölmez, Ali Abbas Gezgin, Bünyamin Sarıtaş**

Department of Gastroenterology, Adana City Training and Research Hospital, Health Sciences University, Adana, Türkiye

Sarcomas constitute a heterogeneous group of tumors arising from different genetic backgrounds. Despite their rarity, they often carry a high mortality rate, largely determined by histological subtype, tumor location, and volume. Liposarcoma accounts for 15%-20% of sarcomas and usually affects the retroperitoneum, trunk, and extremities. In the literature, gastric, duodenal, and colon invasion/metastasis of liposarcoma is rarely reported. These patients seldom present with upper gastrointestinal bleeding or gastric perforation. Here, a rare case of retroperitoneal liposarcoma that invaded the stomach and caused recurrent upper gastrointestinal bleeding is presented. A 74-year-old female patient presented to the emergency department with complaints of abdominal pain and melena. In her past medical history, she had been diagnosed with retroperitoneal liposarcoma 1 year earlier, for which she underwent 2 surgical resections followed by 14 cycles of chemotherapy. She had experienced 2 episodes of upper gastrointestinal bleeding previously. On physical examination, her vital signs were stable; however, her overall condition was poor. Abdominal computed tomography revealed a heterogeneous mass measuring 21 × 19 cm in the abdomen. Laboratory examination revealed WBC: 18.1 × 10^3^/µL, hemoglobin: 4.4 g/L, C-reactive protein: 305 mg/L, and albumin: 16 g/L. Other laboratory tests were normal. The patient received 2 units of erythrocyte suspension (control hemoglobin: 7 g/dL). Subsequently, endoscopy was performed, revealing a giant mass lesion occupying the gastric corpus and fundus. In light of her medical history, this finding was highly suggestive of retroperitoneal liposarcoma invasion of the stomach. She was treated conservatively. The patient died 20 days later. The patient’s legal representatives provided written consent for this article. Liposarcomas can rarely invade the stomach at an advanced stage. Recurrent upper gastrointestinal bleeding may occur, as seen in this case. Prognosis is poor at this stage.


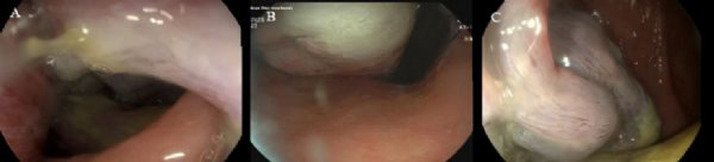


Figure 1. Endoscopic view of a giant fibrosarcoma invading a large area of the stomach.


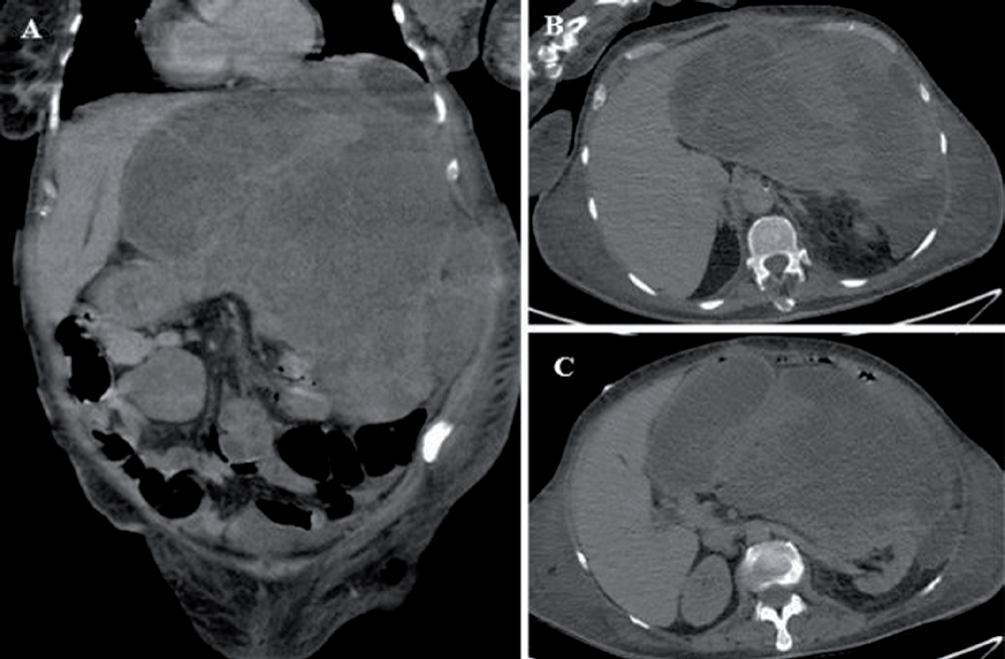


Figure 2. Abdominal computed tomography showing the giant mass in the coronal plane (A) and in the transverse sections (B and C)

## PS-125 Selective IgA Deficiency and Celiac Disease

**Mehmet Refik Göktuğ, Önder Bekar, Fatih Eren, Macit Gülten**

Department of Gastroenterology, Bursa Uludağ University Faculty of Medicine, Bursa, Türkiye

Celiac disease (CD) is typically diagnosed through serological detection of anti-tissue transglutaminase (tTG) IgA autoantibodies. However, selective immunoglobulin A (IgA) deficiency occurs 10-15 times more frequently in patients with CD than in the general population. As most patients are unaware of their IgA deficiency, diagnosis can be significantly delayed. In such cases, IgG-based serologic testing plays a critical role. Here, a case of CD diagnosed in a patient with known IgA deficiency who presented with atypical symptoms is presented. A 22-year-old man came to the gastroenterology outpatient clinic with a history of recurrent oral aphthous ulcers since childhood. He was under immunological follow-up for selective IgA deficiency and was not on regular medication. There was no history of genital ulcers, uveitis, papulopustular rash, abdominal pain, diarrhea, bloating, or weight loss. Laboratory findings were as follows: Hgb 14.2 g/dL, ferritin 6 ng/mL, folic acid <1.6 ng/mL, vitamin B12 312 pg/mL, ANA and RF negative, IgA <0.058 g/L. Serologic tests revealed anti-tTG IgG >200 U/mL, along with positive anti-endomysial IgG and anti-gliadin IgG. Esophago-gastro-duodenoscopy was performed due to the positive IgG-type celiac antibodies. Endoscopic findings included intestinal lymphangiectasia in the duodenal bulb and mucosal scalloping in the second part of the duodenum. Duodenal biopsies demonstrated histopathological features consistent with gluten-sensitive enteropathy, classified as Marsh type 3. The patient was diagnosed with CD, and a gluten-free diet was initiated. Clinical follow-up is ongoing. As standard serological tests primarily detect IgA antibodies, selective IgA deficiency may result in false-negative findings. Therefore, assessment of IgG-based antibodies is essential in suspected cases. In line with Cataldo et al, patients with IgA deficiency often present with atypical or silent CD, as demonstrated in this case. Early recognition ensures timely diagnosis and appropriate management.

## PS-127 Gastrointestinal Graft-Versus-Host Disease with Colonic Involvement: A Case Report

**Ufuk Yazar ^1^ , Özlem Gül ^2^ , Enes Çelikmakas ^1^ , Hatice Azra Begüm Sarıoğlu Salimoğlu ^1^ , Hasan Kurt ^1^ , Emre Akarsu ^3^ , Bilal Ergül ^2^**

^1^Department of Internal Medicine, Lokman Hekim University Faculty of Medicine, Ankara, Türkiye

^2^Department of Gastroenterology, Lokman Hekim University Faculty of Medicine, Ankara, Türkiye

^3^Department of Medical Pathology, Lokman Hekim University Faculty of Medicine, Ankara, Türkiye

Allogeneic hematopoietic stem cell transplantation is a potentially curative treatment for hematologic malignancies. However, graft-versus-host disease (GVHD) remains a major cause of morbidity and mortality. Gastrointestinal involvement, particularly of the colon, is common, with profuse watery diarrhea being the most frequent manifestation. Here, a case of gastrointestinal GVHD following allogeneic stem cell transplantation is presented. A 61-year-old man was admitted to the gastroenterology clinic with a 1-month history of watery diarrhea. His medical history revealed diffuse large B-cell lymphoma in 2018, autologous transplantation in 2019, and haploidentical transplantation in 2024 following relapse, as no suitable donor was available. At 7 months posttransplant, he reported 10-15 episodes of watery diarrhea daily, without mucus or blood. Stool tests for infectious colitis were negative. Colonoscopy showed diffuse ulcerations throughout the colon, the largest measuring 1-2 cm with central whitish exudates. In the distal rectum and sigmoid colon, whitish areas between ulcers were observed. Histopathological examination demonstrated crypt apoptosis, crypt loss, and ulceration, consistent with GVHD. GVHD after allogeneic transplantation is a severe immunologically mediated complication often affecting the gastrointestinal tract. Diarrhea is the leading symptom, with weight loss, abdominal pain, anorexia, and malabsorption as additional features. Endoscopic findings typically include mucosal erythema, edema, ulcerations, and exudates. The differential diagnosis includes infectious colitis (*Clostridioides difficile*, CMV, adenovirus), drug-induced enterocolitis, and inflammatory bowel disease. Histopathology is critical, with crypt apoptosis, mucosal injury, and ulceration as characteristic findings. In this patient, colonoscopic and biopsy findings confirmed GVHD after haploidentical transplantation. Early diagnosis and timely immunosuppressive therapy are essential to reduce morbidity and mortality. GVHD should always be considered in transplant recipients with persistent diarrhea.


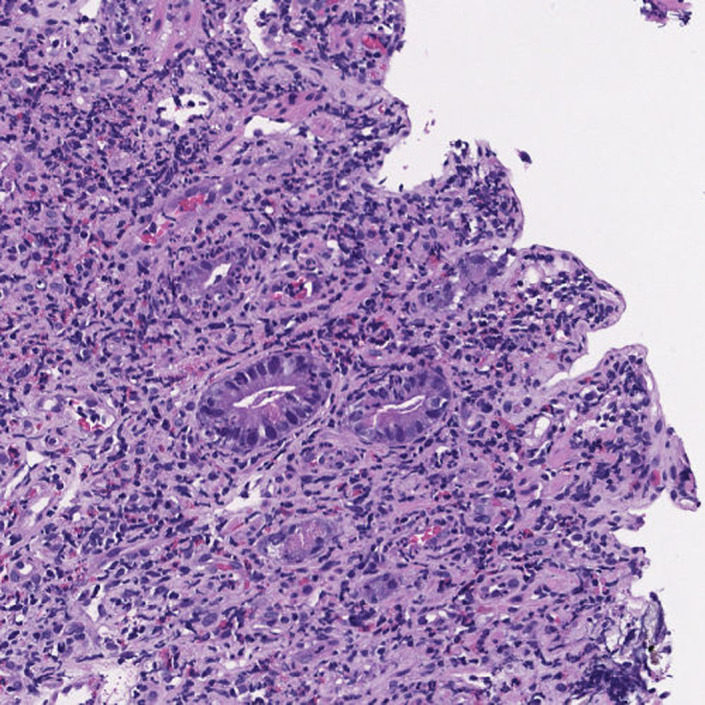


Figure 1. Pathology Image

## PS-128 Evaluation of Alzheimer Patients Who Unintentionally and Completely Pulled Their Percutaneous Endoscopic Gastrostomy Bumpers Off Into the Atmosphere

**Gökhan Aydın ^1^ , Ahmet Cumhur Dülger ^1^ , İsmail Aydın ^2^**

^1^Division of Gastroenterology, Giresun University School of Medicine, Giresun, Türkiye

^2^Division of General Surgery, Giresun University School of Medicine, Giresun, Türkiye

**Background/Aims: **Patients with ALZD undergo PEG procedures primarily to prevent malnutrition. Major complications of this procedure include aspiration pneumonia, hemorrhage, buried bumper syndrome, bowel perforation, necrotizing fasciitis, and metastatic seeding. Cases of patients unintentionally and completely pulling their PEG bumpers into the atmosphere are very rare, and there are no reported data in the literature on this unique issue.

**Materials and Methods: **Between October 2024 and October 2025, a total of 37 patients with ALZD (26 females; 70.3%, mean age 82 ± 21 years) who unintentionally pulled out their PEG bumpers into the atmosphere were enrolled and further compared to patients (21 patients, 17 females, 81%; mean age: 84.1 ± 12.3 years) who underwent standard PEG exchange procedures due to PEG dysfunction and deformation.

Statistical Analysis: Data obtained in this study were statistically analyzed using SPSS version 25.0 statistical software. Categorical variables were compared using the chi-square test. *P*<.05 values were considered statistically significant.

**Results: **The mean duration of PEG use (PEG takers) was 3 months, whereas in the PEG exchange group (PEG exchangers), it was 18.7 months (*P* < .05). With the exception of 2 patients, the PEG procedure was successfully performed for all study participants. (The former PEG orifice was also used for 15 patients.) No internal organ injuries were reported in the PEG takers group. The mean levels of serum direct bilirubin (0.57 ± 0.69 mg/dL versus 0.23 ± 0.17 mg/dL; *P* = .005), GGT (64 ± 50.6 U/l vs. 26.7 ± 22.7 U/L; *P* = .004), serum uric acid (6.26 ± 2.85 mg/dL vs. 4 ± 1.69 mg/dL; *P* = .008), and procalcitonin (16.6±34.3 μg /L vs. 1.56 ± 0.37 µg/L; *P* = .001) were significantly higher in the PEG takers group.

**Conclusion: **Higher levels of cholestatic enzymes, hyperuricemia, and hyperprocalcitoninemia can be predictive factors for estimating unintentional PEG pulling out in patients with ALZD. A shorter duration (less than 2 years) PEG exchanges due to PEG dysfunction was also a key indicator of inadequate PEG care in the coastal the Black Sea region.

## PS-129 A Rare Case of Gastric Outlet Obstruction: Bouveret Syndrome

**Yasemin Gündoğdu ^1^ , Orhan Cem Deniz ^2^ , Müjgan Orman ^3^ , Arzu Tiftikçi ^2^**

^1^Department of Internal Medicine, Acıbadem Mehmet Ali Aydınlar University, İstanbul, Türkiye

^2^Department of Gastroenterology, Acıbadem Mehmet Ali Aydınlar University, İstanbul, Türkiye

^3^Department of Radiology, Acıbadem Mehmet Ali Aydınlar University, İstanbul, Türkiye

Bouveret syndrome is a rare variant of gallstone ileus, characterized by gastric outlet obstruction due to the migration of a gallstone into the duodenum through a bilioenteric fistula. Because the presentation is atypical and nonspecific, and often occurs in the elderly, this syndrome has a documented high mortality rate of 12%-30%. A case of a patient presenting with persistent vomiting, upper abdominal pain, and constipation, where prompt intervention was performed is reported. A 71-year-old woman with no significant medical history presented with a 1-week history of intermittent abdominal pain, persistent nausea, and recurrent episodes of nonbilious vomiting. The pain was postprandial and located in the right upper quadrant, necessitating several emergency department visits during the past week. She reported decreased passage of flatus, along with an absence of bowel movements for 5 days. On physical examination, tenderness was noted in the epigastric region and RUQ, without signs of peritoneal irritation, and bowel sounds were hypoactive. Laboratory investigations revealed leukocytosis with a WBC count of 14.72 × 10^3^/μL, neutrophilia (11.8 × 10^3^/μL), and increased hemoglobin (16.6 g/dL) with a CRP of 23 mg/dL. Endoscopic evaluation revealed hyperemic and ulcerated mucosa from the distal esophagus to the gastric body. The antrum was hyperemic, and a voluminous biliary structure, likely a fistulized gallbladder remnant, was observed in the duodenal bulb. An abdominopelvic CT showed a 2 cm centrally radiolucent structure with a calcified rim in the distal ileum, suggesting a calculus with proximal small bowel dilatation up to 3.5 cm, consistent with ileus. On abdominal MRI, the gallbladder was found to be connected to the wall of the duodenal bulb. The preliminary diagnosis was Bouveret syndrome, and the patient was referred for surgical management. Bouveret syndrome is a rare but serious complication of gallstone disease and should be considered in patients with unexplained vomiting, epigastric pain, and recent constipation.


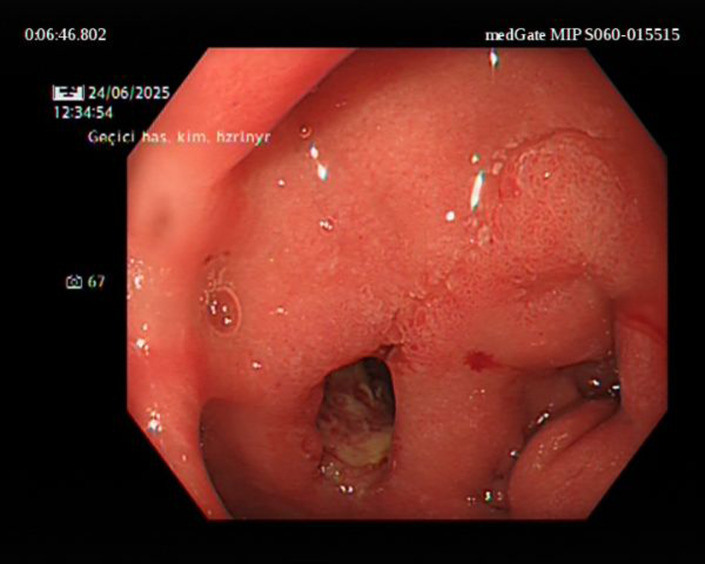


Figure 1. Diverticular appearance in the bulbus.


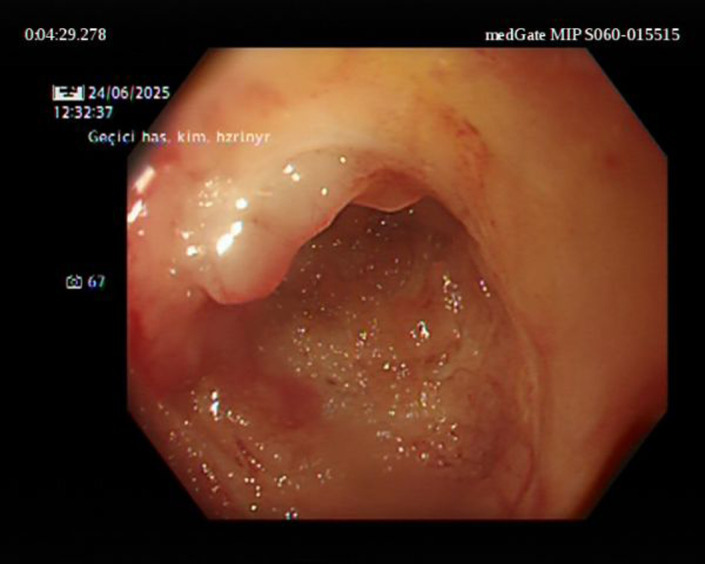


Figure 2. Identifiable biliary epithelium in the diverticulum.


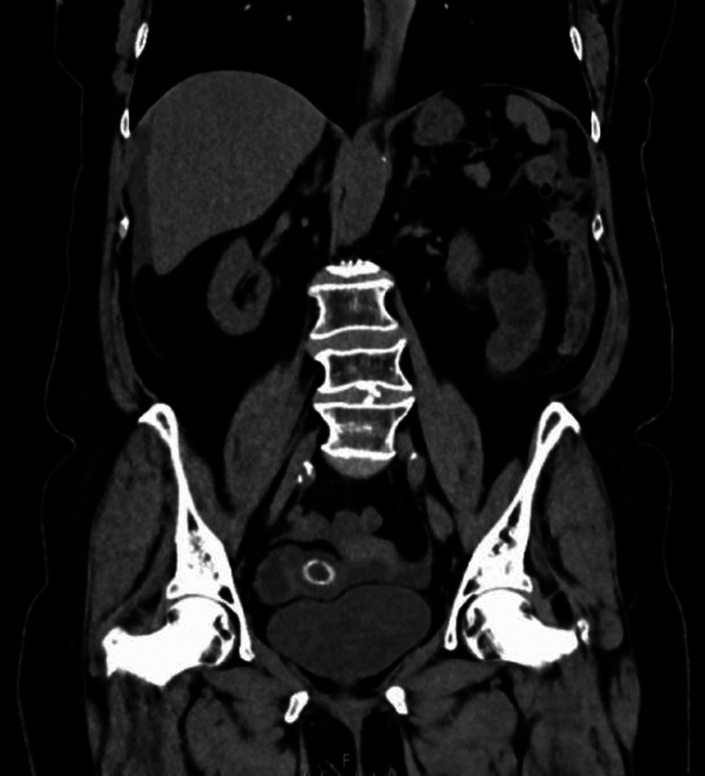


Figure 3. Abdominopelvic CT section: Calcified structure in the distal ileum compatible with a gallstone, accompanied by findings of ileus.

## PS-131 The Impact of Familial Predisposition on Disease Severity and Treatment Requirements in Inflammatory Bowel Disease

**Eren Düzgün**

Gastroenterology Clinic, İzmir City Hospital, İzmir, Türkiye

Inflammatory bowel disease (IBD) is a complex autoimmune condition characterized by chronic inflammation of the gastrointestinal tract and damage to the mucosal layer. It primarily presents in 2 clinical forms: Crohn’s disease (CD) and ulcerative colitis (UC). Numerous studies have demonstrated that IBD has a strong genetic component, and individuals with a family history of IBD are at significantly increased risk of developing the disease. A positive family history of IBD has been associated with variations in age of onset, clinical course, and response to treatment. Genetic predisposition is thought to play a crucial role in determining the phenotypic features and prognosis of the disease. Several studies suggest that patients with a familial background of IBD may experience a more severe disease course and require more intensive treatment protocols. In this study, data were retrospectively collected from patients diagnosed with Crohn’s disease and ulcerative colitis who presented to the IBD outpatient clinic at İzmir City Hospital’s Department of Gastroenterology. The study focused on patients with first-, second-, and third-degree relatives affected by IBD. According to the findings, the rate of biological drug usage among patients with a family history of IBD was 71.8% in those diagnosed with CD and 20.8% in those diagnosed with UC. These results suggest that a family history of IBD—particularly in Crohn’s disease—is associated with a more aggressive disease course and a greater need for advanced therapeutic interventions.

## PS-132 How Should ERCP Be Performed in Situs Inversus Totalis?

**Mustafa Zanyar Akkuzu ^1^ , Ahmet Yavuz ^1^ , Ümit Karabulut ^1^ , Çiğdem Budak Ece ^1^ , Berat Ebik ^1^**

Department of Gastroenterology, Diyarbakir Gazi Yasargil Training and Research Hospital, Ministry of Health University, Kayapınar, Diyarbakır, Türkiye

Situs inversus totalis (SIT) is a rare autosomal recessive condition characterized by complete inversion of thoracic and abdominal organs, with an estimated incidence ranging from 1 in 6000 to 1 in 80 000. SIT results from a 270° clockwise rotation of the embryonic midgut, contrary to the normal 270° counterclockwise rotation^1^. This reversed anatomy complicates both the diagnosis and treatment of intra-abdominal diseases.^2^^,^^3^ Endoscopic procedures, particularly ERCP, demand specialized techniques in such patients. A literature review revealed fewer than 50 published ERCP cases in patients with SIT^4^, primarily as case reports highlighting variations in endoscope handling and patient positioning^5^. Due to these challenges, ERCP in SIT is technically demanding and associated with increased risk. Here, the present experience with a successful ERCP in a patient with SIT is reported, aiming to provide practical insights for similar rare cases.


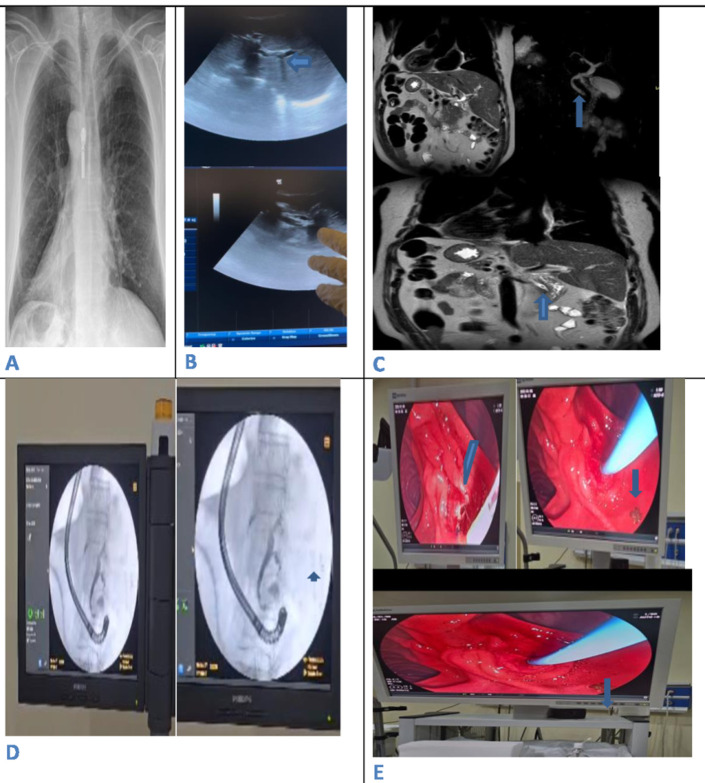


Figure 1. Radiological appearances of situs inversus totalis and stone removal with ERCP. (A) PAAC dextrocardia image. (B) Ultrasonographic image of the liver with situs inversus abdominalis and appearance of the stone in the bile duct. (C) Magnetic resonance cholangiography appearance of situs inversus totalis and bile duct stones. (D) Appearance of the common bile duct on fluoroscopic imaging. (E) Removal of the stone with ERCP.

## PS-133 Effective Treatment of Gastric Obstruction Associated with Walled-off Necrosis with Endoscopic Stent

**Sanlı Arabacı, Bora Aktaş, Haluk Cihad Albayrak, Kerem İzmirlioğlu, Mehmet Raşit Ayte, Seçkin Özgül, Metin Uzman, Evrim Kahramanoğlu Aksoy**

Ankara Atatürk Sanatorium Training and Research Hospital, Ankara, Türkiye

One of the complications that may develop after acute pancreatitis is walled-off necrosis (WON), which can prolong the clinical course and lead to severe morbidity. WON occurs when necrotic tissue in the pancreatic or peripancreatic region is surrounded by a fibrous capsule, and over time, it may create a mass effect and influence adjacent organs. Gastric outlet obstruction secondary to external compression or direct gastric wall involvement is a rare complication that may arise due to WON. It may present with symptoms such as nausea, vomiting, and pain, significantly limiting patients’ oral intake. A 35-year-old male patient presented to the outpatient clinic with complaints of recurrent nausea and vomiting occurring immediately after meals. It was learned that at an external center, cystogastrostomy had been performed due to walled-off necrosis (WON) secondary to acute pancreatitis, and the collection had been drained. However, since the patient’s complaints persisted, he was referred to the clinic. Upon detection of gastric outlet obstruction through imaging and endoscopy, a 24F, 10 cm long covered metallic stent was placed. His clinical condition was observed to improve in a short period. In this case, endoscopic gastric lumen stent treatment demonstrates that it can be an effective and safe option in the management of gastric outlet obstruction secondary to WON.


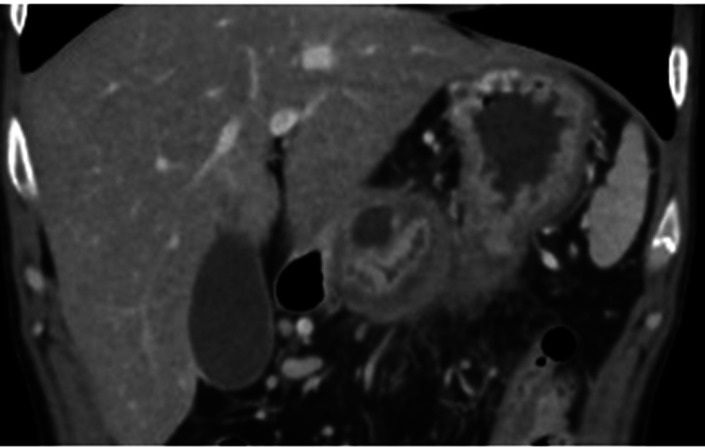


Figure 1. CT images of gastric outlet obstruction.

Axial and coronal contrast-enhanced CT images reveal gastric wall thickening resulting in luminal compression, accompanied by a peripherally enhancing loculated fluid collection.


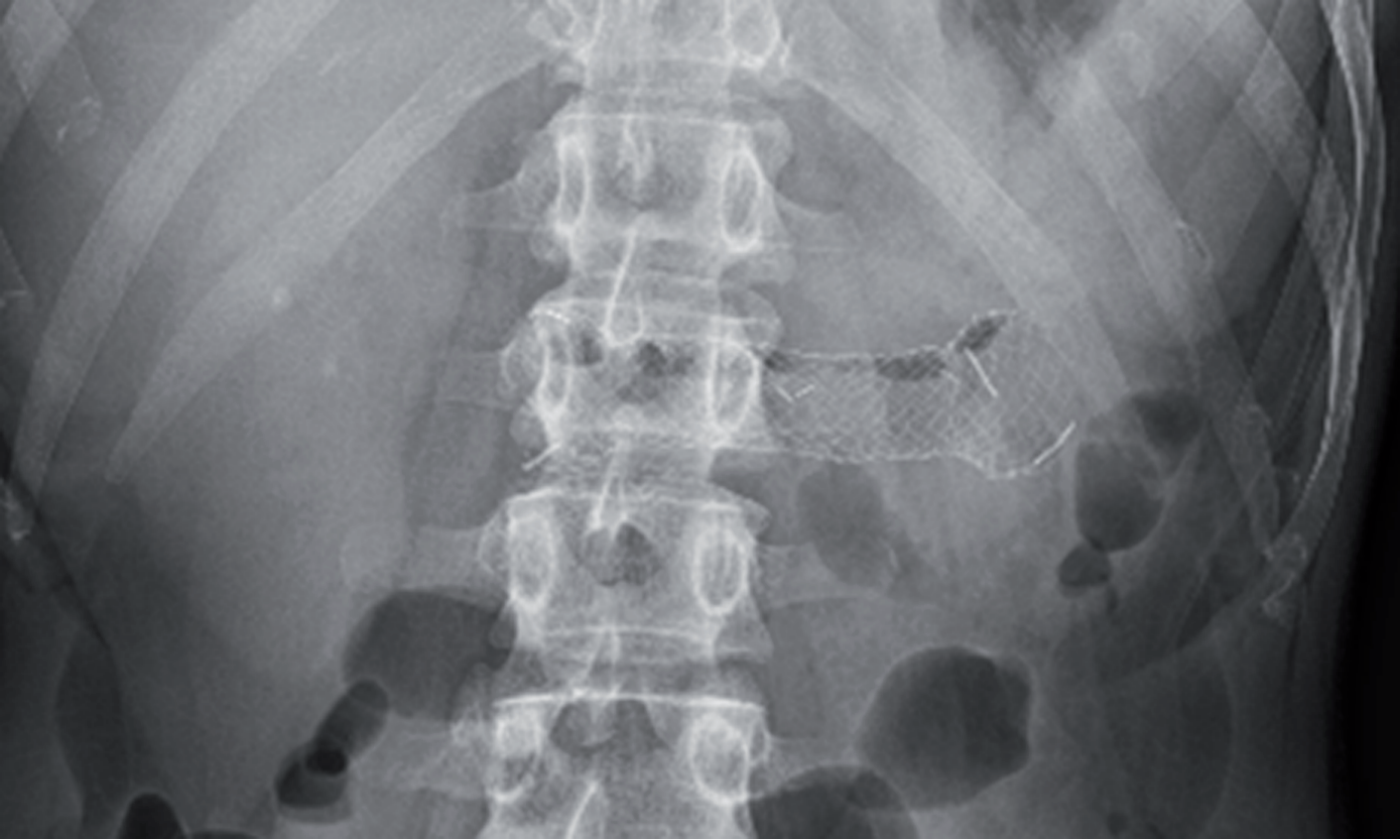
 
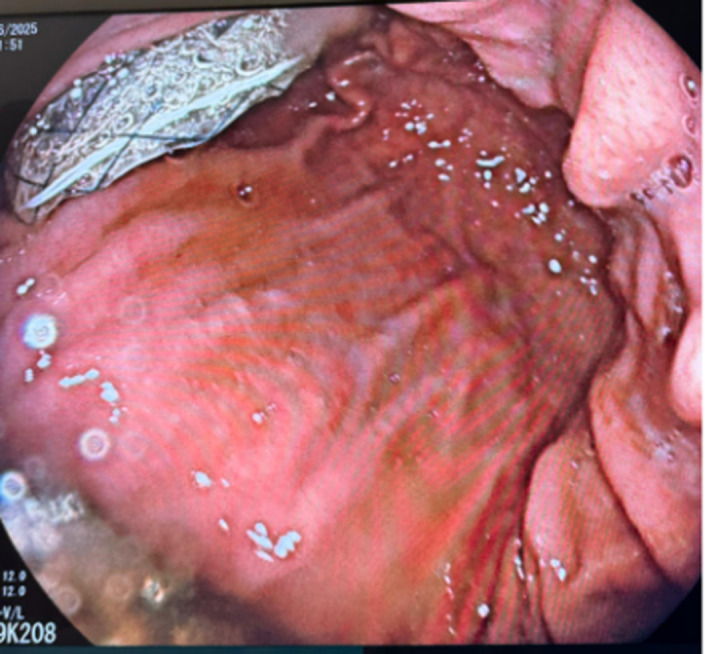


Figure 2. Gastric outlet obstruction (GOO) and endoscopic stent placement.

.

Following gastric outlet obstruction, a metallic stent is visualized within the gastric outlet. Fluid collections were also observed. A metallic stent is also visible.

## PS-135 Single-Center Endoscopic Retrograde Cholangiopancreatography Experiences

**Osman Bedir ^1^ , Aysun Çalışkan ^1^ , Zekiye Nur Harput ^1^ , Derya Ünal ^2^ , Süleyman Coşgun ^1^**

^1^ Gastroenterology Clinic, Kütahya City Hospital, Kütahya, Türkiye

^2^ Internal Medicine Clinic, Kütahya City Hospital, Kütahya, Türkiye

**Background/Aims: **Endoscopic retrograde cholangiopancreatography (ERCP) is an advanced endoscopic procedure that involves inserting an endoscope into the second portion of the duodenum to visualize the bile ducts and pancreatic ducts both fluoroscopically and endoscopically. It requires specialized training, has diagnostic and therapeutic implications, and, although rare, can sometimes lead to serious complications. The goal in this presentation is to share the ERCP experience over the past 1.5 years.

**Materials and Methods:** In this study, 480 ERCP procedures performed at Kütahya City Hospital over the last 1.5 years were evaluated retrospectively. Age, gender, laboratory findings, endoscopic intervention results, and postoperative complications of patients with ERCP were obtained from the hospital automation system. Procedure indications and complications that developed during and after the procedure were evaluated.

**Results**: Of the 480 cases that underwent ERCP, 247 were female (51.5%) and 233 were male (48.5%). The mean age was 64.19 (SD = 15.238) years. Among the cases, 243 (5.6%) were naive, and 236 (49.1%) had undergone the procedure before. Among the naive cases undergoing ERCP, 157 (64.6%) had choledocholithiasis, 23 (9.4%) had malignancy, 28 (11.5%) had bile duct strictures, 13 (5.3%) had cholangitis, 5 (2%) had sphincter of Oddi dysfunction, 4 (1.6%) had bile duct injuries and postoperative bile leakage, 4 (1.6%) had pancreatic duct stones, pancreatitis, and pancreatic leakage, and 4% had other rare conditions. Twenty (8.2%) patients developed PEP, and 1 patient experienced bleeding requiring hospitalization and intervention. Other complications, such as minor bleeding and perforation, were less than 1%.

**Conclusion:** Several factors such as patient selection, the skill of the operator, and the complexity of the procedure can increase the risks of ERCP complications. ERCP indications must be determined accurately. This procedure should be performed in skilled hands or under their supervision due to rare but potentially fatal complications. It is believed that sharing the results will be beneficial for gaining experience.

## PS-136 A Rare Cause of External Compression on the Stomach Fundus: Renal Cell Carcinoma

**Kerem İzmirlioğlu, Bora Aktaş, Sanlı Arabacı, Haluk Cihad Albayrak, Seçkin Özgül, Mehmet Raşit Ayte, Metin Uzman, Evrim Kahramanoğlu Aksoy**

Ankara Atatürk Sanatorium Training and Research Hospital, Ankara, Türkiye

Although external compression is a common cause of findings in upper gastrointestinal endoscopy, it is often overlooked and missed. It can be confused with subepithelial lesions, especially during standard endoscopic examinations. A broad differential diagnosis should be considered in cases with external compression findings that resemble a submucosal lesion on endoscopy. Although gastrointestinal stromal tumors, lymphoma, pancreatic masses, or splenic pathologies are frequently observed, tumors originating from retroperitoneal organs should not be ignored. Importantly, these patients require more detailed evaluation and advanced imaging and diagnostic methods.

Renal cell carcinoma is the most common malignant tumor of the kidney and is known for its clinically silent course and late-stage symptoms. Although the classic triad of hematuria, flank pain, and a palpable mass is common, these findings are only observed in a small portion of patients. In this case, compression was detected in the proximal gastric fundus and corpus during the endoscopic examination performed on a 49-year-old male patient who had been suffering from weight loss, bloating, nausea, and vomiting for 3 months in the outpatient clinic. Advanced imaging methods performed on the patient revealed that the gastric compression was due to renal cell carcinoma. These findings were examined in light of the literature and discussed the symptoms that suggest malignancy, as well as the imaging methods that should be used when external compression of the stomach is observed. Finally, it was emphasized that external pressure on the stomach is a finding that should not be overlooked and warrants investigation, with renal cell carcinoma being a very rare potential cause.


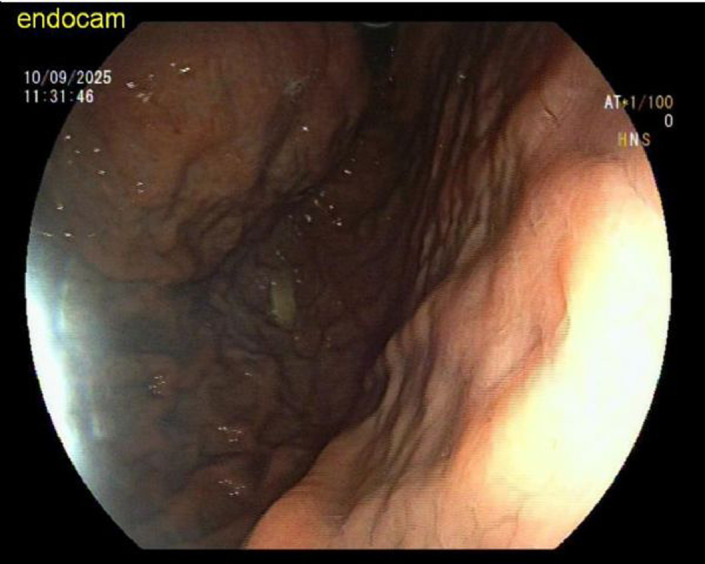


Figure 1. Endoscopy image. External compression on the stomach fundus and corpus


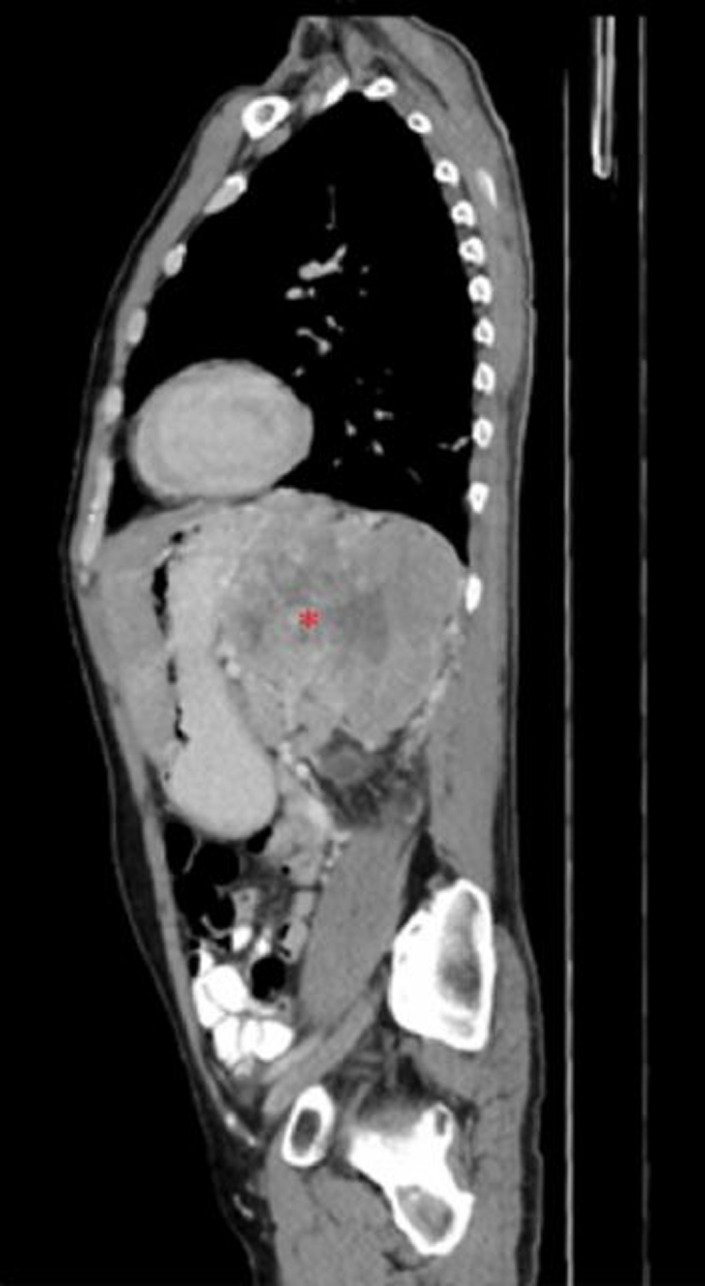


Figure 2. Abdominal CT sagittal plane. The mass in the left kidney area is compressing the fundus of the stomach


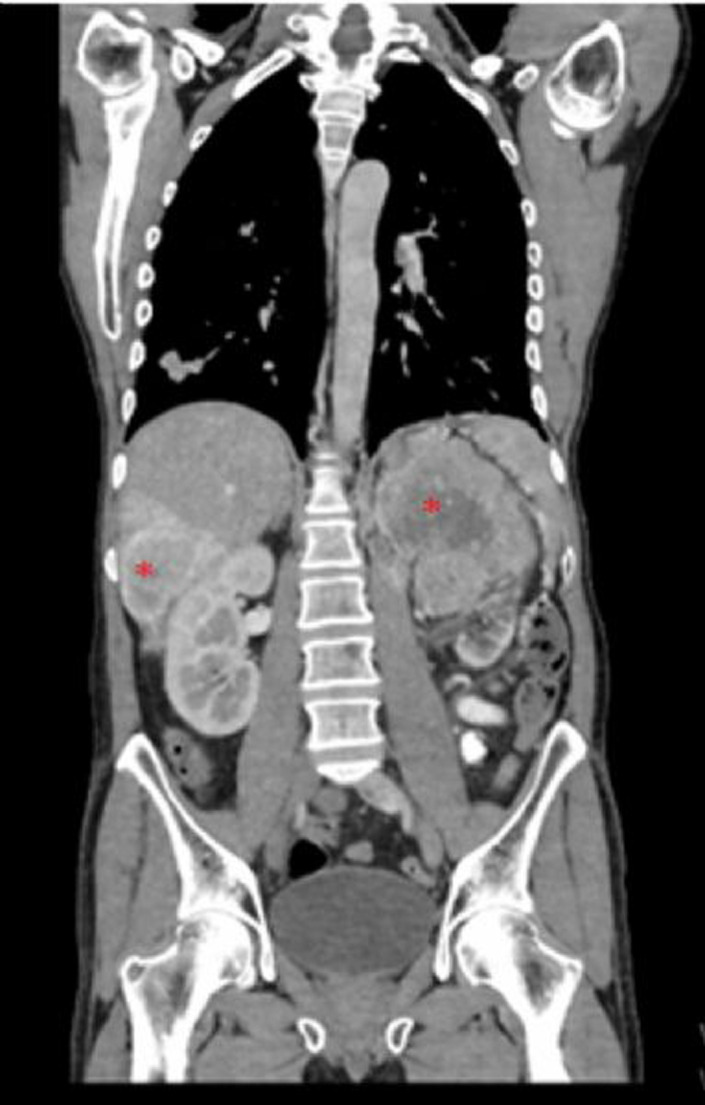


Figure 3. Abdominal CT coronal plane. A mass originating from the left kidney and adjacent organ metastases is observed.

## PS-138 Endoscopic Diagnosis and Management of Acute Esophageal Variceal Bleeding: Tertiary Center Experience

**Ahmet Faruk Kalkışım ^1^ , Muhammed Mustafa İnce ^1^ , Mahmut Yüksel ^1^ , Diler Taş Kılıç ^2^ , Hasan Tankut Köseoğlu ^1^ , Mevlüt Hamamcı ^1^ , Derya Arı ^1^ , Mücahit Ergül ^1^ , Emin Altıparmak ^1^ , Meral Akdoğan Kayhan ^1^**

^1^Department of Gastroenterology, Ankara Bilkent City Hospital, Ankara, Türkiye

^2^Department of Gastroenterology, Ankara Training and Research Hospital, Ankara, Türkiye

**Background/Aims:** The incidence of esophageal variceal bleeding (EVB) in patients with cirrhosis varies between 10% and 15% annually, with the rate increasing with the severity of liver disease and the size of the varices. The objective of this study is to review the clinical outcomes of the treatment and follow-up of patients admitted to the hospital emergency department with EVB.

**Materials and Methods:** This study includes 143 patients with cirrhosis who presented to the emergency department of the hospital between 2019 and 2023 with symptoms of upper gastrointestinal bleeding and were diagnosed with gastrointestinal varices during emergency endoscopy. Demographic information, medical history, and details about the treatment provided to the patients in the hospital were recorded.

**Results:** The length of hospitalization was observed to be longer in women than in men (*P* = .036). Patients who underwent endoscopic intervention after 12 hours had a longer hospitalization than those who underwent endoscopic intervention before 12 hours (*P* = .035). A statistically significant correlation was identified between 6-week mortality and Model for End-Stage Liver Disease-sodium (MELD-Na) scores (*P* = .012). MELD-Na scores were higher in the group of patients who experienced mortality within a 6-week period.

**Conclusion:** When patients with cirrhosis present to the emergency department with signs of gastrointestinal bleeding, variceal bleeding should be suspected first, and the infusion of vasoactive agents should be started as soon as possible. The endoscopic procedure should be performed within 12 hours.

## PS-139 Clarithromycin-Associated Acute Pancreatitis: A Case Report

**Akif Orhan, Serkan Öcal, Osman Çağın Buldukoğlu, Galip Egemen Atar, Mehmet Şerif Aktaş, Merve Eren**

Department of Gastroenterology, Antalya Training and Research Hospital, Antalya, Türkiye

Acute pancreatitis is an inflammatory disease of the pancreas that develops due to the premature activation of digestive enzymes. The most common etiological causes are gallstones and alcohol consumption, whereas drug-induced pancreatitis is rare. Among macrolide antibiotics, cases related to erythromycin and roxithromycin have been reported, but clarithromycin-associated cases remain extremely rare. Possible mechanisms include sphincter of Oddi spasm leading to biliary reflux or hypersensitivity reactions. A case of acute pancreatitis that developed during clarithromycin therapy is presented. A 69-year-old man with a history of type 2 diabetes mellitus and hypertension was being followed in the orthopedic ward due to a hip fracture. During hospitalization, he developed a cough, and chest radiography revealed findings consistent with pneumonia. The patient was started on ceftriaxone 2 g and clarithromycin 500 mg orally. On the fourth day of treatment, he developed severe epigastric pain radiating to the back. Laboratory tests revealed serum amylase of 171 U/L and lipase of 228 U/L. Abdominal CT showed pancreatic edema and peripancreatic fluid collections. Gallstones, hypercalcemia, and hypertriglyceridemia were excluded. Due to clinical deterioration, he was transferred to the intensive care unit, where oral intake was stopped, and intravenous fluids, analgesia, and supportive treatment were initiated. Following the discontinuation of clarithromycin, clinical and laboratory improvement was observed. The patient recovered and was discharged after the completion of treatment. In this case, the onset of acute pancreatitis shortly after the initiation of clarithromycin, in the absence of other etiological factors, strongly suggests a drug-related association. The literature includes reports of both reversible and fatal clarithromycin-associated cases. This indicates that, although rare, clarithromycin may cause pancreatitis as a serious adverse event. In the present patient, early recognition, discontinuation of the drug, and supportive treatment led to recovery. Clinicians should consider drug-induced pancreatitis in patients presenting with unexplained abdominal pain while taking clarithromycin.

## PS-140 A Rare Cause of Abdominal Pain: Intestinal Lymphoma

**Fadime Yılmaz ^1^ , Süleyman Dolu ^2^**

^1^Department of Internal Medicine, Dokuz Eylul University Faculty of Medicine, İzmir, Türkiye

^2^Department of Gastroenterology, Dokuz Eylul University Faculty of Medicine, İzmir, Türkiye

Intestinal lymphomas are rare extranodal non-Hodgkin lymphomas originating from the gastrointestinal tract, most commonly involving the small intestine. Clinical manifestations are often nonspecific and may include abdominal pain, anorexia, weight loss, constipation, or signs of intestinal obstruction. As a result, diagnosis is frequently delayed, and advanced imaging and endoscopic evaluation play a crucial role in detection. This case report presents the diagnostic process of an intestinal lymphoma in a patient admitted with abdominal pain and weight loss. A 64-year-old man with a history of hypertension, type 2 diabetes mellitus, and hyperlipidemia presented with a 2-month history of abdominal pain, dyspepsia, intermittent constipation, and an unintentional weight loss of 5 kilograms. The patient had previously been hospitalized twice for ileus but had no surgical history. Upper and lower endoscopic examinations performed at an external center were unremarkable. Contrast-enhanced abdominal computed tomography revealed a 12-cm concentric wall thickening, mural enhancement, and aneurysmal dilation in the proximal jejunum, accompanied by heterogeneous mesenteric tissue and conglomerated necrotic lymph nodes. In addition, a 23-mm omental metastatic lesion was detected. Diagnostic double-balloon enteroscopy revealed a friable, ulcerative mass in the midjejunum that obstructed the scope passage, and multiple biopsies were obtained. Histopathological examination confirmed the diagnosis of intestinal lymphoma. The radiologic and endoscopic findings were consistent with the characteristic features of intestinal lymphoma. This case emphasizes that intestinal lymphoma should be considered in the differential diagnosis of patients presenting with chronic abdominal pain and unexplained weight loss, particularly when imaging reveals aneurysmal dilation and concentric wall thickening. Early recognition and histopathological confirmation through endoscopic biopsy are essential for timely hematologic evaluation and appropriate treatment planning.


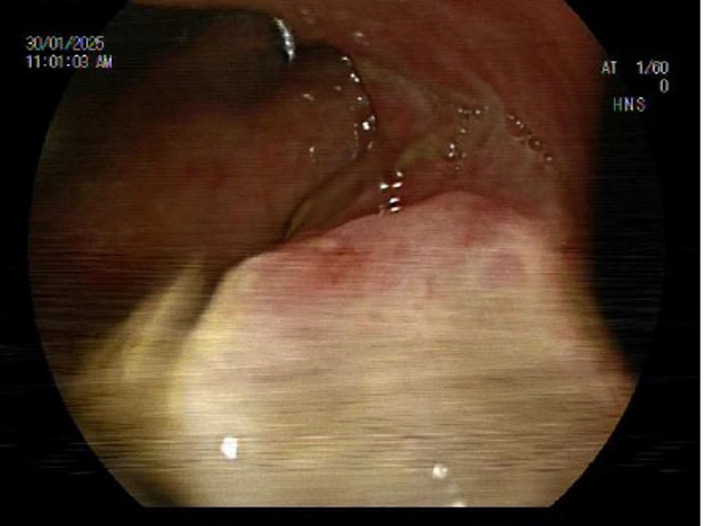


Figure 1. Ulcerated and fragile mass appearance in the jejunum during double-balloon enteroscopy.

## PS-141 IgG4-Related Autoimmune Pancreatitis: A Case Report on Diagnosis, Treatment, and Relapse Management

**Fadime Yılmaz ^1^ , Sema Çakıroğlu ^1^ , Göksel Bengi ^2^ , Müjde Soytürk ^2^ , Ömer Selahattin Topalak ^2^ , Nilay Danış ^2^ , Süleyman Dolu ^2^**

^1^Department of Internal Medicine, Dokuz Eylul University Faculty of Medicine, İzmir, Türkiye

^2^Department of Gastroenterology, Dokuz Eylul University Faculty of Medicine, İzmir, Türkiye

IgG4-related autoimmune pancreatitis (type 1 AIP) is the pancreatic manifestation of IgG4-related disease, representing a rare, steroid-responsive form of chronic pancreatitis. It accounts for approximately 6% of chronic pancreatitis cases, and its pathogenesis is characterized by IgG4-positive plasma cell infiltration, storiform fibrosis, and obliterative phlebitis. Clinically, it often presents with painless obstructive jaundice, cholestatic liver enzyme increase, and biliary strictures. Diagnosis is established through a comprehensive assessment of characteristic imaging findings, increased serum IgG4 levels, histopathologic features, and a prompt response to corticosteroid therapy. A 66-year-old man with a history of diabetes mellitus, coronary artery disease, and ulcerative colitis for 23 years presented with jaundice. Laboratory evaluation revealed AST: 433 U/L, ALT: 399 U/L, GGT: 1784 U/L, ALP: 132 U/L, total bilirubin: 16.6 mg/dL, and direct bilirubin: 8.92 mg/dL. MRCP showed irregularities and focal dilatations in the intrahepatic and common bile ducts, along with biliary sludge. Endoscopic ultrasound (EUS) demonstrated a hypoechoic heterogeneous area in the pancreas and a dilated common bile duct. Fine-needle aspiration biopsy was negative for malignancy, and an increased serum IgG4 level (1280 mg/dL) supported the diagnosis of IgG4-related autoimmune pancreatitis. Methylprednisolone 40 mg/day was initiated, leading to normalization of liver function tests, a decrease in bilirubin to 1.71 mg/dL, and IgG4 reduction to 562 mg/dL. During follow-up, laboratory results showed recurrence with AST: 235 U/L, ALT: 280 U/L, ALP: 1206 U/L, GGT: 993 U/L, total bilirubin: 1.35 mg/dL, direct bilirubin: 0.41 mg/dL, and IgG4: 1250 mg/dL. Consequently, ursodeoxycholic acid (UDCA) and azathioprine (AZA 50 mg/day, gradual escalation) were added to low-dose methylprednisolone (8 mg/day). Following AZA therapy, liver function tests normalized completely, and the patient achieved clinical remission. This case demonstrates the favorable steroid response of IgG4-related autoimmune pancreatitis and the effectiveness of immunomodulator therapy in managing relapses.


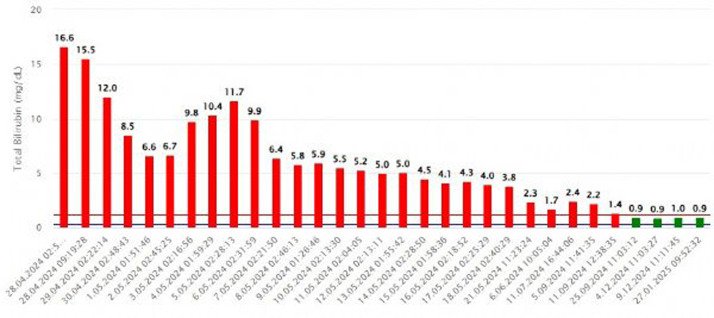


Figure 1. Changes in total bilirubin levels over time after methylprednisolone and azathioprine therapy.

In a patient with an initial total bilirubin level of 16.6 mg/dL, a marked improvement was achieved with methylprednisolone followed by azathioprine therapy, resulting in normalization of the bilirubin curve.

## PS-142 Herpes Simplex Virus Esophagitis Following Solid Organ Transplantation: Clinical Challenges and Therapeutic Approach

**Nur İlayda Genç ^1^ , Devrim Müge Özarı Gülnar ^2^ , Burcu Saka Gürbüz ^3^ , Genco Gençdal ^2^**

^1^Department of Internal Medicine, Koç University Hospital, İstanbul, Türkiye

^2^Department of Gastroenterology and Hepatology, Koç University Hospital, İstanbul, Türkiye

^3^Department of Pathology, Koç University Hospital, İstanbul, Türkiye

Herpes simplex virus (HSV) infections frequently occur as reactivation events in immunosuppressed patients and represent a significant risk in the posttransplantation period [1]. They are particularly common in the early posttransplant phase and may present with severe clinical manifestations [2]. This report aims to present a case of HSV esophagitis that developed following renal transplantation. A 69-year-old female patient with a history of chronic kidney disease secondary to microscopic polyarteritis nodosa (mPAN), hypothyroidism, and hypertension was evaluated for sore throat and a sensation of food sticking while eating. She had undergone renal transplantation 1 week earlier, and her symptoms had started just after the operation, progressively worsening. Dysphagia was present for both liquids and solids, being more pronounced with solids. Medication history revealed long-term use of methylprednisolone 4 mg, amlodipine10 mg, and levothyroxine sodium 25 µg. Before transplantation, she had received 5 sessions of plasmapheresis and intravenous immunoglobulin (IVIG),with 40-60 mg of methylprednisolone as premedication during each session. After transplantation, 3 additional sessions of plasmapheresis and antithymocyte globulin (ATG) therapy were administered. As part of her immunosuppressive and prophylactic therapy, she was receiving tacrolimus 2 × 3.5 mg, methylprednisolone 5 mg, mycophenolate mofetil 2 × 720 mg, valganciclovir 450 mg, trimethoprim-sulfamethoxazole 400/80 mg, and fluconazole 100 mg. On physical examination, a few white aphthous-like lesions were noted in the oral cavity. Epigastric tenderness was present. Laboratory evaluation revealed no electrolyte imbalance. C-reactive protein (CRP) was 8.6 mg/L. Complete blood count showed leukocytes 7.40 K/µL, neutrophils 6.9 K/µL, and lymphocytes 0.10 K/µL. Nystatin suspension, pantoprazole 40 mg/day, an alginate-sodium bicarbonate-calcium carbonate suspension 3×1, and sucralfate 3×1 were started. Endoscopy revealed circumferential erosions and ulcers in the distal esophagus (Los Angeles classification: Grade D esophagitis) and erosive antral gastritis. Follow-up CMV-PCR was negative. Histopathology demonstrated the “3M” triad (multinucleation, chromatin margination, and nuclear molding), consistent with HSV esophagitis. Valganciclovir 2 × 1000 mg (14-21 days) was initiated. However, due to the development of nausea and myalgia, the drug was discontinued. Valganciclovir was replaced with valacyclovir 2 × 1000 mg, under which the patient’s symptoms showed marked improvement.

Conclusion: Although Candida and CMV esophagitis are among the most common causes of symptoms such as dysphagia and odynophagia, HSV esophagitis can also occur in transplant recipients, making the differential diagnosis more challenging [3-4]. This case highlights the importance of early endoscopic intervention for guiding differential diagnosis and appropriate treatment.


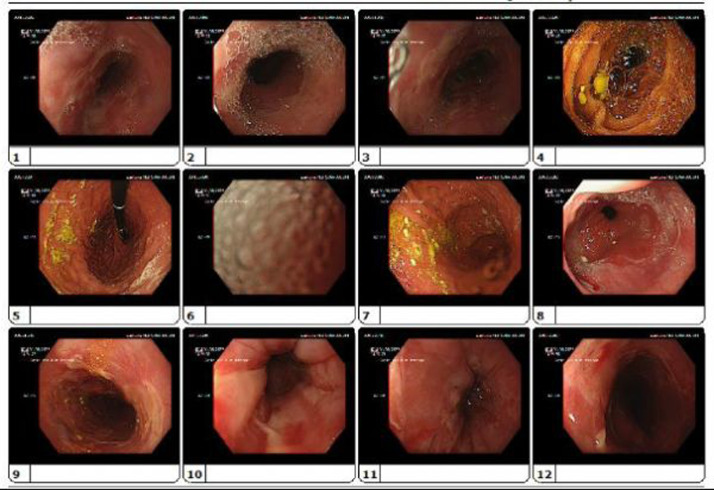


Figure 1. Endoscopic findings.

Upper gastrointestinal endoscopy demonstrating circumferential erosions and deep ulcers in the distal esophagus, consistent with Los Angeles classification Grade D esophagitis


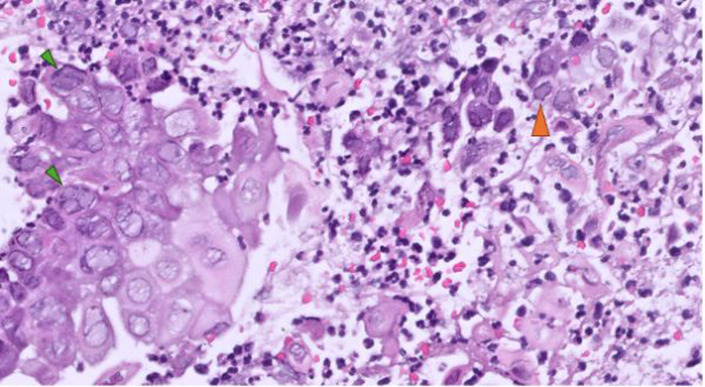


Figure 2. Histopathology.

Squamous epithelial cells demonstrating the cytopathic effects of HSV within the ulcer base (H&E ×400).

## PS-143 A Case Series of IgG4-Related Disease Presenting as Pancreatic Cancer Mimic

**Berrin Yalınbaş Kaya ^1^ , Erdal Bodakçı ^2^ , Ayşenur Sevinç ^1^ , Yusuf Abul ^1^ , Muhammet Kocabaş ^1^ , Ali Türeyen ^1^ , Selcan Cesur ^1^**

^1^Department of Gastroenterology, Eskişehir City Hospital, Health Sciences University, Eskişehir, Türkiye

^2^Department of Rheumatology, Eskişehir City Hospital, Health Sciences University, Eskişehir, Türkiye

Immunoglobulin G4-related disease (IgG4-RD) is a systemic fibroinflammatory disorder. Its most frequent manifestation, type 1 autoimmune pancreatitis (AIP-1), often presents with pancreatic mass lesions that closely mimic pancreatic adenocarcinoma (PA), creating a significant diagnostic challenge. This case series highlights the clinical, laboratory, and radiological spectrum of IgG4-RD associated with cholestasis resembling malignancy. Case 1 involved a 58-year-old female who developed prolonged postoperative cholestasis. Imaging revealed a 5-cm pancreatic mass with a high SUVmax (7.1). Despite repeated negative biopsies for malignancy, markedly increased serum IgG4 (14.5 g/dL) and storiform fibrosis confirmed AIP. She responded dramatically to steroids and azathioprine. Case 2 was a 65-year-old man who presented with cholestasis and cholecystitis. MRCP/CT demonstrated pancreatic thickening. Increased IgG4 (458 mg/dL) and biopsies showing storiform fibrosis confirmed AIP with IgG4-related cholangiopathy (IgG4-RC). Prednisolone and methotrexate were initiated. Case 3 involved a 57-year-old man who developed cholestasis after acute pancreatitis. PET-CT showed pancreatic head involvement, prostate uptake, and widespread lymphadenopathy (SUVmax: 8.28). Multiorgan IgG4-RD was confirmed by biopsies. Steroid and methotrexate treatment led to biochemical and radiological remission. Case 4 was a 41-year-old woman who presented with a focal pancreatic lesion (40×31 mm). Initial biopsies were nondiagnostic, and the patient subsequently developed portal vein thrombosis. A repeat pancreatic biopsy demonstrated IgG4-positive plasma cell infiltration, confirming the diagnosis of IgG4-related disease. Methotrexate and prednisolone were initiated, resulting in a favorable therapeutic response. Case 5 was a 46-year-old man with cholestasis, pancreatitis, and alcoholic hepatitis who had increased IgG4 (4.62 g/L) and liver biopsy findings of IgG4+ plasma cell infiltration, consistent with AIP and IgG4-RC. IgG4-RD presenting with a pancreatic mass and increased FDG uptake can be indistinguishable from PA. It should be considered in patients with repeatedly negative biopsies and increased serum IgG4. A rapid, favorable response to corticosteroids and immunosuppressants is a key diagnostic hallmark.

Table 1.Biochemical Data of Cases

19-9 IgG4, immunoglobulin G4; ALP, alkaline phosphatase; CA 19-9, carbohydrate antigen.

Table 2.Clinical Findings of Cases

F, female; M, male.

Table 3.Radiological and Pathological Data of Cases

CT, computed tomography; FDG, fluorodeoxyglucose; IgG4, immunoglobulin G4; MRCP, magnetic resonance cholangiopancreatography; MRI, magnetic resonance imaging; PET-CT, positron emission tomography-computed tomography; US, ultrasonography.

**Table:** 

Case	ALP	Bilirubin	CA 19-9	IgG4
1	120	10.2/7.82	3.37	14.5
2	206	8.2/5.9	7.12	4.58
3	448	4.3/3.2	129.7	1.1
4	60	0.39/0.19	<2	0.688
5	556	7.18/4.64	12.2	28

**Table:** 

Case	Age/Gender	Symptom/Time to Diagnosis	Differential Diagnosis
1	58/F	Weight loss, abdominal pain/ 54 months	Pancreatic carcinoma
2	65/M	Pruritus/1 month	Pancreatic carcinoma
3	57/M	Postpancreatitis jaundice, weight loss/ 3 months	Metastatic pancreatic carcinoma, prostate carcinoma
4	41/F	Fatigue, jaundice, portal hypertension/6 months	Pancreatic carcinoma
5	46/M	Abdominal pain, increased LFT/5 months	Pancreatic carcinoma

**Table:** 

Case	Involvement and Biopsy	Radiology	Pathology
1	Pancreas head and pancreas tru-cut	MRCP/CT: Hypoechoic mass, distal CBD stricture	Chronic inflammation, storiform fibrosis, IgG4+ plasma cells
2	Pancreas and Pancreas tru-cut	CT/MRI: Increased pancreas thickness	IgG4+ plasma cells, storiform fibrosis, lymphoplasmacytic inflammation
3	Pancreas, prostate and pancreas, prostate tru-cut	PET-CT: Pancreas mass, lymphadenopathy, prostate involvement	Storiform fibrosis, lymphoplasmacytic inflammation, IgG4+ plasma cells
4	Pancreas head, liver, and pancreas tru-cut	US/PET-CT: pancreas hypoechoic lesion, enhanced FDG in pancreatic head	Fibrosis, IgG4+ plasma cells, lymphoid aggregation
5	Pancreas, liver, and liver tru-cut	CT/PET-CT: pancreas body and tail	IgG4+ plasma cells Lymphoplasmacytic infiltration, fibrosis

## PS-144 Grasping Forceps Preference for Endoscopic Removal of Gastrointestinal Foreign Bodies

**Ayşe Bostancı, Satiye Tuğba Köseoğlu, Songül Tarkan Tüysüz, Kübra Köroğlu Gürbüz, Muharrem Bostancı, Kaan Demirören**

Pediatric Gastroenterology Clinic, SBU Yüksek Ihtisas Training and Research Hospital, Bursa, Türkiye

**Background/Aims:** Foreign bodies (FBs) lodged in the airway or gastrointestinal (GI) tract and causing death are the sixth most common cause of accidental death, with 64% of these cases occurring in children aged 5 years and younger. This study aimed to determine the grasping forceps used for the removal of FBs in children.

**Materials and Methods:** Data from 132 (4%) of 3300 upper GI endoscopy procedures performed in the pediatric gastroenterology unit between 2018 and 2025 for FB ingestion were reviewed.

**Results:** Although no FBs were observed in 51 (39%) of the cases, they were removed endoscopically in 81 (61%). Of the removed FBs, 39 (48%) were coins, 13 (16%) were needles, 6 (7%) were discoid batteries, 4 (5%) were cylindrical batteries, 4 (4%) were magnets, and 14 (17%) were objects such as screws, drawer handles, and toothpicks. Grasping forceps with a net were most commonly used for the removal of these objects (58 cases, 72%). Additionally, a polyp snare was used in 15 (19%) of the cases, and rat-tooth grasping forceps were used in 5 (6%). In 3 cases, the FBs were grasped with rat-tooth, alligator-jaw, and tripod grasping forceps, pulled into the stomach, and then removed using grasping forceps with a net.

**Conclusion:** Although the grasping forceps chosen for each type of FB differ depending on the length and width of the object, the most commonly used grasping forceps in this study were those with a net and polyp snare. Therefore, these forceps should be available in every endoscopy unit.

## PS-145 Secretory Diarrhea due to Villous Adenoma Presenting with Increased Fecal Calprotectin

**Sema Çakıroğlu ^1^ , Mesut Şan ^2^ , Süleyman Dolu ^2^ , Hale Akpınar ^2^**

^1^Department of Internal Medicine, Dokuz Eylül University Faculty of Medicine, İzmir, Türkiye

^2^Department of Gastroenterology, Dokuz Eylül University Faculty of Medicine, İzmir, Türkiye

Chronic diarrhea is a challenging condition with a wide differential diagnosis, including infectious, inflammatory, malabsorptive, functional, and less commonly, neoplastic causes. Villous adenomas are usually asymptomatic polyps; however, when large and located in the rectosigmoid region, they may secrete excessive mucus and electrolytes, resulting in secretory diarrhea. This clinical picture, known as McKittrick-Wheelock syndrome, is characterized by hypokalemia, dehydration, and metabolic disturbances. Fecal calprotectin is commonly used as a biomarker for inflammatory bowel disease (IBD). Nevertheless, tumoral processes such as villous adenomas may also cause significant increases, potentially misleading clinicians toward inflammatory disorders. A 58-year-old man with hypertension was admitted with severe nausea and diarrhea for 6-7 months. He reported 30-40 daily bowel movements of variable consistency, mostly yellow and mucus-rich. Etiological investigations were performed. Fecal fat testing excluded steatorrhea, and serology for celiac disease was negative. Despite replacement therapy, he had persistent hypokalemia. Laboratory evaluation revealed increased fecal calprotectin (1700 μg/mg). With a preliminary diagnosis of IBD, endoscopy was performed. Colonoscopy revealed a pedunculated 2.5 cm polyp in the midsigmoid colon, which was resected. Following polypectomy, the patient’s diarrhea ceased, and electrolyte levels normalized. Histopathology confirmed villous adenoma. This case shows that resistant hypokalemia and high-volume, mucoid diarrhea may be caused by villous adenoma, a rare but treatable entity. Increased fecal calprotectin is not specific for IBD and may also increase in neoplastic conditions. Awareness of this overlap is crucial to avoid misdiagnosis and delay. Endoscopy plays a vital role, as complete removal of the lesion can lead to dramatic clinical improvement. In patients with chronic diarrhea, electrolyte disturbances, and high fecal calprotectin, clinicians should maintain a broad differential diagnosis, including both inflammatory and neoplastic causes.


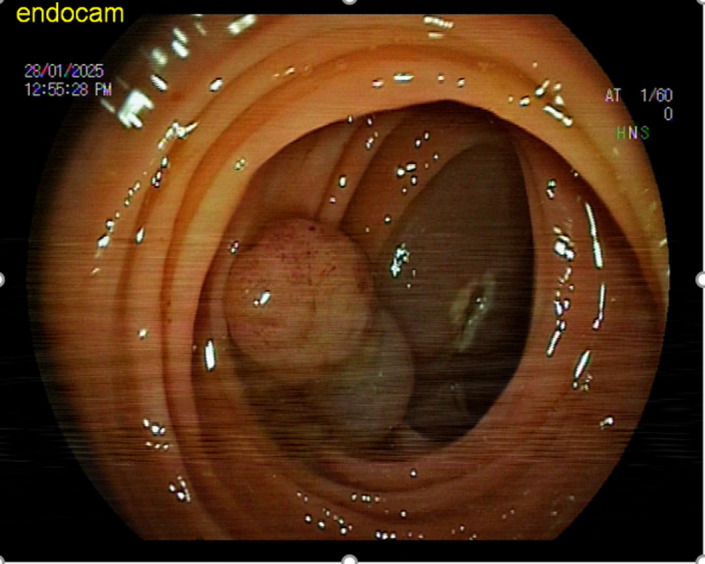


Figure 1. The image of the villous adenoma after resection.

## PS-146 Diagnosis with Double-Balloon Enteroscopy: A Rare Case of Jejunal Diverticulosis

**Sema Çakıroğlu ^1^ , Fadime Yılmaz ^1^ , Süleyman Dolu ^2^**

^1^Department of Internal Medicine, Dokuz Eylül University Faculty of Medicine, İzmir, Türkiye

^2^Department of Gastroenterology, Dokuz Eylül University Faculty of Medicine, İzmir, Türkiye

Gastrointestinal (GI) bleeding is a common cause of emergency admissions and hospitalizations. In elderly patients, underlying chronic diseases and comorbidities may complicate the identification of the bleeding source. Although most upper and lower GI bleeding cases can be diagnosed through standard endoscopy, some patients experience persistent bleeding despite normal findings. In such cases, small bowel bleeding should be considered. Small bowel diverticula are rare and usually asymptomatic but may occasionally cause severe and recurrent bleeding. Jejunal diverticulosis, in particular, is uncommon and can present with significant clinical complications. Early recognition is important to avoid unnecessary investigations and repeated transfusions. Here, a patient with recurrent hematochezia who required multiple blood transfusions and was diagnosed with jejunal diverticulosis using advanced endoscopic evaluation was reported. A 68-year-old woman with hypertension and type 2 diabetes mellitus was admitted for the evaluation of recurrent GI bleeding. She was not taking antiplatelet or anticoagulant medications. The patient had several previous hospitalizations for hematochezia and had received a total of 10 units of packed red blood cell transfusions. Prior gastroscopy and colonoscopy at an outside center revealed no pathology. Due to chronic kidney disease, contrast-enhanced CT angiography cannot be performed. Therefore, double-balloon enteroscopy was conducted. The procedure revealed approximately 25-30 diverticula, the largest measuring about 5 cm, extending from the distal duodenum through the jejunum. These findings were consistent with jejunal diverticulosis. Jejunal diverticulosis is a rare but potentially serious condition that may cause massive or recurrent GI bleeding. In elderly patients with comorbidities and unexplained bleeding despite normal endoscopy, small bowel sources should be suspected. Double-balloon enteroscopy provides both diagnostic and potential therapeutic benefits. Early diagnosis can prevent unnecessary procedures, reduce repeated transfusions, and improve patient management.


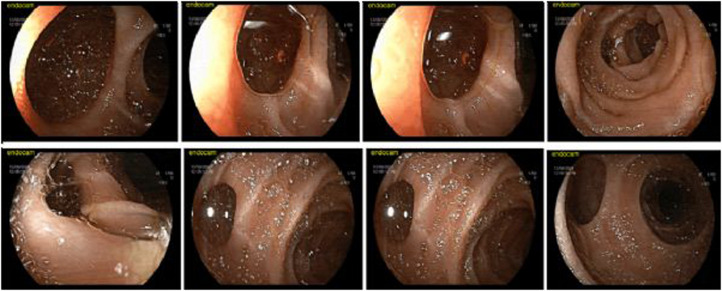


Figure 1. Images of jejunal diverticulosis detected by double-balloon enteroscopy.

## PS-147 Autoimmune Hepatitis-Associated IgA Nephropathy: A Case Report

**Akif Orhan, Serkan Öcal, Osman Çağın Buldukoğlu, Galip Egemen Atar, Mehmet Şerif Aktaş, Merve Eren, Savaş Duman, Ayhan Hilmi Çekin**

Department of Gastroenterology, Antalya Training and Research Hospital, Antalya, Türkiye

Autoimmune hepatitis (AIH) is a chronic liver disease that can rarely involve the kidneys, manifesting as IgA nephropathy (IgAN). The coexistence of AIH and IgAN likely reflects shared immune mechanisms. Recognizing renal involvement is important for management and prognosis, underscoring the need for further research. A 34-year-old man was referred due to increased liver enzymes detected on routine tests. Initial labs showed ALT 89 U/L, AST 118 U/L, GGT 162 U/L, ALP 376 U/L, total bilirubin 1.1 mg/dL, albumin 28.1 g/L, INR 1.38, negative viral markers, ANA (+), ASMA (+), and increased IgG (41 g/L). Before liver biopsy results were available, the patient’s condition worsened, presenting with abdominal pain, ALT 2538 U/L, AST 3259 U/L, total bilirubin 1.8 mg/dL, INR 1.96, and proteinuria of 765 mg/day. Kidney biopsy revealed IgA nephropathy, and liver biopsy was consistent with autoimmune hepatitis. The patient received corticosteroid therapy and an ACE inhibitor, resulting in improved liver function and stabilization of renal parameters. AIH-associated IgAN is exceedingly rare. This case highlights the importance of evaluating renal involvement in patients with AIH presenting with proteinuria or hematuria. Early recognition and appropriate management, including immunosuppressive therapy, can enhance outcomes. Further studies are needed to clarify the pathophysiological link between AIH and IgAN.

## PS-148 Association of Tooth-Brushing Habits and Dental Status with Metabolic Dysfunction–Associated Steatotic Liver Disease Severity Indices: A Report from a Tertiary Care Center in Türkiye

**Sude Türkgüzeli ^1^ , Eda Kaya ^2^ , Çağlayan Keklikkıran ^3^ , Yusuf Yılmaz ^3^**

^1^Recep Tayyip Erdoğan University School of Dentistry, Rize, Türkiye

^2^Department of Medicine, Knappschaft Kliniken Bochum, Ruhr University, Bochum, Germany

^3^ Department of Internal Medicine, Division of Gastroenterology, Recep Tayyip Erdoğan –University School of Medicine, Rize, Türkiye

**Background/Aims:** Accumulating evidence suggests that poor oral hygiene and infrequent tooth brushing are associated with an increased risk of metabolic dysfunction-associated steatotic liver disease (MASLD) and advanced fibrosis. This cohort from a tertiary care center in Türkiye aimed to investigate these associations.

**Materials and Methods:** A total of 483 patients who presented consecutively to the tertiary care hepatology unit. Demographic, laboratory, and FibroScan data were collected prospectively. Hepatic steatosis was defined by a controlled attenuation parameter (CAP) ≥238 dB. Dual etiologies were included in the analysis. Advanced fibrosis was defined as a Fibrosis-4 (FIB-4) index ≥2.67 or liver stiffness measurement (LSM) ≥8 kPa. Cirrhosis was defined as LSM ≥12 kPa, and patients with a FAST score ≥0.67 were considered at risk for MASLD. The associations between these parameters and tooth-brushing habits as well as dental status were analyzed.

**Results:** Tooth brushing frequency was significantly negatively associated with the presence of MASLD (*P* = .012) and with significant fibrosis, defined as LSM ≥ 8 kPa (*P* = .044). In contrast, other dental status parameters showed no significant associations with the noninvasive indices. Age, AST, ALT, platelet count, BMI, waist circumference, systolic and diastolic blood pressure, and heart rate were also significantly associated with LSM ≥ 8 kPa. However, in multivariate analysis, only platelet count, heart rate, age, and AST remained independently associated. Regarding hepatic steatosis, brushing teeth once daily was found to be protective against MASLD (OR 0.534, 95% CI: 0.287-0.993, *P* = .048).

**Conclusion:** In the present analysis, tooth brushing was associated with the presence of MASLD and advanced hepatic fibrosis. However, in multivariate analysis, the association remained significant only for MASLD. Notably, brushing once daily was shown to have a protective effect against MASLD, indicating a potential benefit of tooth brushing at least once daily.

## PS-149 A Rare Diagnosis in a Male Patient Presenting with a Liver Mass: A Case of Breast Carcinoma

**Mesut Şan ^1^ , Süleyman Dolu ^1^ , Rauf Methiyev ^1^ , Civanmert Bayrak ^1^ , Yasemin Çakır ^2^ , Pınar Balcı ^3^**

^1^Department of Gastroenterology, Dokuz Eylul University Faculty of Medicine, İzmir, Türkiye

^2^Department of Pathology, Dokuz Eylul University Faculty of Medicine, İzmir, Türkiye

^3^Department of Radiology, Dokuz Eylül University Faculty of Medicine, İzmir, Türkiye

Male breast carcinoma is a rare malignancy, representing about 1% of all breast cancers. Although histologically similar to the female type, it is often diagnosed at an advanced stage due to low clinical suspicion, leading to a higher incidence of metastatic disease. In male patients presenting with multiple hepatic lesions, breast carcinoma is seldom considered in the absence of a known primary tumor. Here, a rare case of male breast carcinoma initially investigated for possible gastrointestinal malignancy with liver metastases is presented. A 70-year-old man presented with several weeks of abdominal pain. Contrast-enhanced abdominal CT revealed numerous bilobar hypodense hepatic lesions, up to 2 cm in size, suggestive of metastases. The patient was referred to the clinic for evaluation of a possible gastrointestinal primary. Laboratory studies showed markedly increased CEA (1497 ng/mL), CA 125 (>12 000 U/mL), and CA 15-3 (>400 U/mL), whereas CA 19-9, AFP, and PSA were normal. Upper and lower endoscopies were unremarkable. On detailed questioning, the patient reported recent nipple discharge from the left breast. Physical examination revealed a palpable subareolar mass. Core biopsy confirmed invasive breast carcinoma (NST, grade 2).

Immunohistochemistry: ER 95% (+3), PR 90% (+3), HER2 (1+), Ki-67 10%—consistent with a luminal A-like subtype. FDG-PET/CT showed intense uptake in the left breast lesion and additional foci in the liver, bone, and lungs, indicating multiorgan metastatic disease. Male breast carcinoma is often diagnosed at a metastatic stage, frequently during evaluation for malignancy of unknown origin. Combined radiologic, metabolic, and histopathologic assessment is essential for accurate diagnosis and staging. This case highlights the importance of considering male breast carcinoma in the differential diagnosis of male patients presenting with multiorgan metastases, particularly hepatic involvement without a known primary lesion.


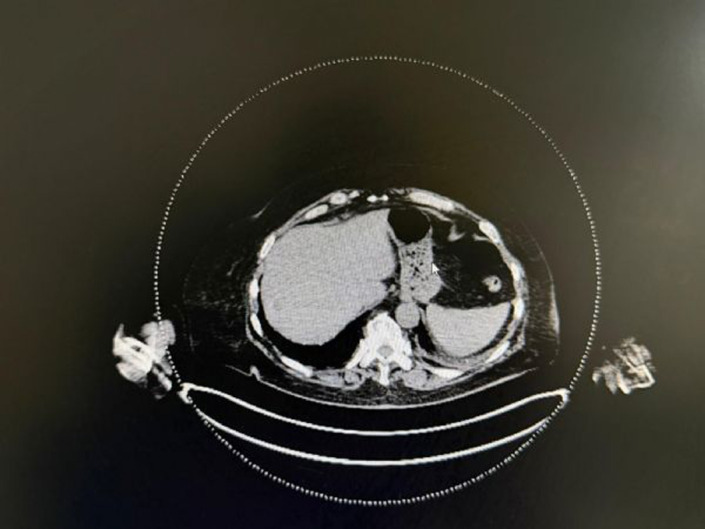



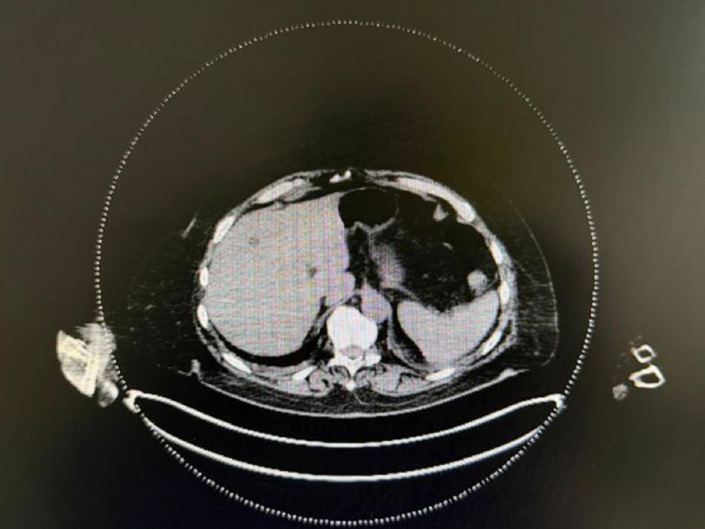


Figure 1. Computed Tomography Image

Scattered millimetric lesions compatible with parenchymal cysts and metastases in the liver parenchyma

Scattered millimetric lesions compatible with parenchymal cysts and metastases in the liver parenchyma


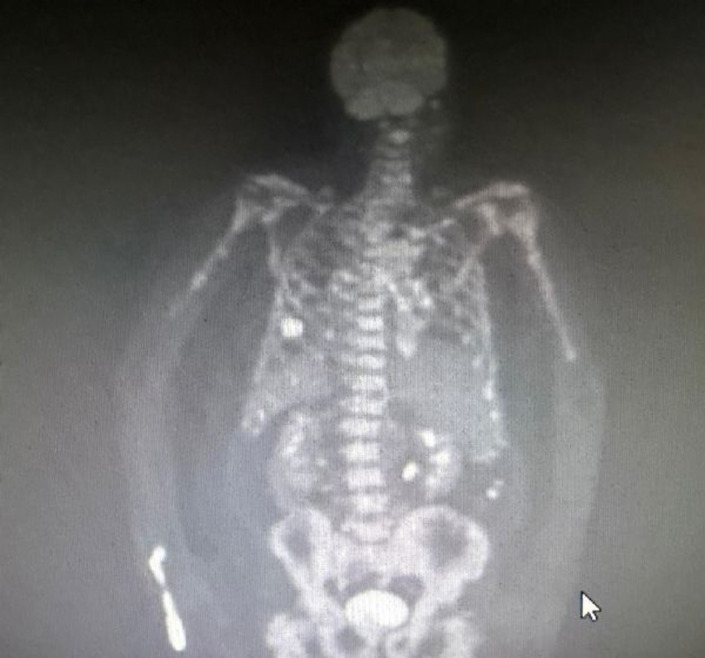


**Figure 2.PET Image**

Malignant soft tissue lesion located retroareolar in the left breast

## PS-150 Hidden Immunodeficiency Presenting with Small Intestinal Findings: From Nodular Lymphoid Hyperplasia to a Diagnosis of Common Variable Immunodeficiency

**Tutku Güleç Eriş ^1^ , Osman Özdoğan ^2^ , Ahmet Emre Ergan ^2^ , Ümit Yeşilova ^2^ , Mammadhasan Mamadov ^2^ , Oktay Bayraktar ^2^ , Mehmet Kasım Aydın ^2^ , Serkan Yaraş ^2^ , Fehmi Ateş ^2^ , Engin Altıntaş ^2^ , Orhan Sezgin ^2^**

^1^Department of Internal Medicine, Mersin University Hospital, Mersin, Türkiye

^2^Department of Gastroenterology, Mersin University Hospital, Mersin, Türkiye

Common variable immunodeficiency is the most frequent cause of primary immunodeficiency in adulthood. In this patient group, B-lymphocyte dysfunction leads to hypogammaglobulinemia, characterized by a marked decrease in immunoglobulin G and at least 1 other isotype, either IgM or IgA. A large proportion of patients present with inflammation and autoimmune manifestations. One of the most commonly affected systems is the gastrointestinal tract, which is the largest immune-related organ. Both infectious and noninfectious enteropathy are more common in CVID. A 40-year-old foreign male patient presented with dyspeptic complaints. His symptoms have persisted for 14 years, since his initial presentation in Russia in 2011, followed by repeated consultations. Endoscopic and colonoscopic evaluations performed in Russia revealed lymphoid hyperplasia, and he was followed up for suspected dysplasia. The patient was re-evaluated due to his prolonged complaints. Endoscopy and colonoscopy were performed. On colonoscopy, advancement to the terminal ileum revealed numerous polypoid lesions, the largest measuring approximately 5-6 mm, consistent with nodular exaggerated lymphoid hyperplasia. In the cecum and proximal ascending colon, micronodular structures were also observed. Multiple biopsies were taken from the nodular lymphoid lesions. Given the persistence of diffuse nodular hyperplastic changes, an immunodeficiency syndrome was suspected. Laboratory investigations showed significantly reduced IgG and IgM levels, with IgA measured at 0 mg/dL. After exclusion of differential diagnoses, the process that began with ileal lymphoid hyperplasia ultimately led to the diagnosis of common variable immunodeficiency. The patient was referred to hematology, and hematologic follow-up continues with a treatment plan of intravenous immunoglobulin infusion every 28 days. This case highlights that, in cases of gastrointestinal complaints and pathologies, noninfectious inflammatory and autoimmune disorders such as common variable immunodeficiency should be considered in the differential diagnosis due to their close relationship with the immune system. Although rare, awareness of such conditions facilitates earlier diagnosis when encountered.

## PS-151 An ERCP Experience in a Patient with Anomaly of Apical Biliary Opening in the Bulbus and Common Bile Duct Stone

**Şafak Meriç Özgenel**

Department of Gastroenterology, Osmangazi University Faculty of Medicine, Eskişehir, Türkiye

Ectopic opening of the common bile duct into the duodenal bulb is a rare condition, occurring in 0.1%-2.7% of cases of abnormal biliary drainage. ERCP is the gold standard for diagnosis. Sphincterotomy should be avoided due to the risk of bleeding and perforation. ERCP with balloon dilatation is a common approach. A 70-year-old male patient was admitted to another center with complaints of epigastric pain, swelling, and fever lasting for 5 days. The patient underwent ERCP for choledocholithiasis at another center, but the procedure failed due to duodenal stenosis. He was then referred to the hospital. On admission, laboratory results indicated WBC: 5870/uL, AST: 71 U/L, ALT: 106 U/L, ALP: 455 U/L, GGT: 411 IU/L, total bilirubin: 4.37 mg/dL, direct bilirubin: 3.69 mg/dL, CRP: 43 mg/L, and procalcitonin: 3.50 ng/mL (normal range: 0-0.046). In the second-look ERCP, a double-channel endoscope was advanced to the bulb, and the second part of the duodenum was passed. The bulb was found to be edematous upon retrospective examination. Scar tissue due to a peptic ulcer was observed. The orifice of the papilla of Vater was noted to open into the bulb (apical opening anomaly). A guidewire was advanced through the wide channel of the double-channel endoscope, and the common bile duct was cannulated with a sphincterotome. In cholangiography, the intrahepatic bile ducts appeared normal, whereas the common bile duct was dilated. Opacity compatible with a stone was observed in the choledoch. Balloon dilatation of the common bile duct was performed using an 8-9-10-mm-diameter dilatation balloon. The common bile duct was recannulated with a stone balloon catheter, and a 0.5-cm-diameter stone was removed. A straight plastic stent was placed in the common bile duct, and the procedure was terminated. Duodenal stenting was not performed. No complications occurred during the postprocedure follow-up, and laboratory parameters improved along with the patient’s clinical symptoms. Cholecystectomy followed by stent removal was planned. Biliary opening anomalies should be considered when the papilla of Vater cannot be visualized in its normal anatomical location. Visualization and cannulation of the common bile duct may be possible with devices such as a forward-viewing dual-channel endoscope. ERCP with balloon dilation is the safest approach.


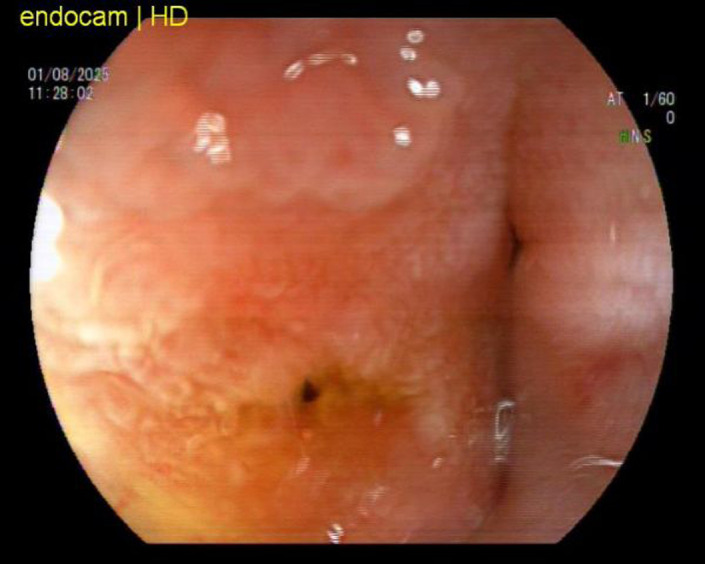


Figure 1. Papilla Vateri seen in the bulb.


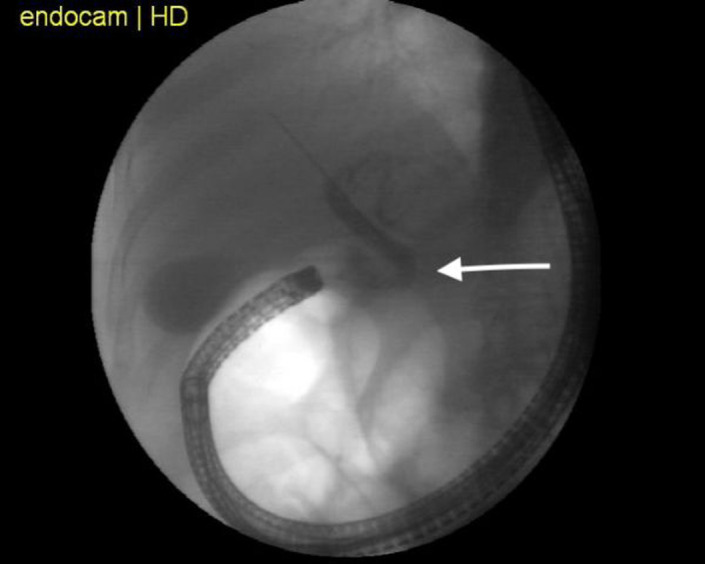


Figure 2. Cholangiogram after radio-opaque material injection; note the hook-shaped appearance of the common bile duct indicated by the white arrow.


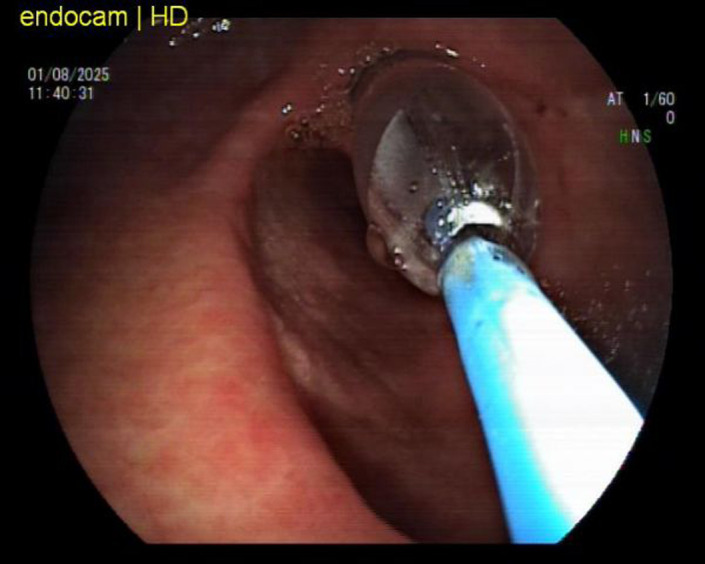


Figure 3. Balloon dilatation of the Papilla Vateri seen in the bulb and the appearance of the balloon extending to the antrum.

## PS-153 Twin Pregnancy in a Patient with Liver Cirrhosis Secondary to Autoimmune Hepatitis: A Rare Case Report

**Şehmus Ölmez, Bünyamin Sarıtaş**

Department of Gastroenterology, Adana City Training and Research Hospital, Health Sciences University, Adana, Türkiye

In patients with liver cirrhosis, pregnancy is rarely seen due to the effects of amenorrhea, anovulation, and advanced age. Pregnancy creates serious risks for both the mother and the fetus. In these patients, twin pregnancies are very rarely observed. The presence of twin pregnancy further increases the risks. Here, a case of twin pregnancy in a patient with liver cirrhosis secondary to autoimmune hepatitis is presented. A 26-year-old patient with a diagnosis of cirrhosis due to autoimmune hepatitis presented to the outpatient clinic at the 12th week of pregnancy for follow-up. She had been diagnosed with cirrhosis for 3.5 years and had grade 2/3 varices in the esophagus on endoscopy. Physical examination revealed hepatosplenomegaly. Laboratory tests showed WBC: 2100/mL, platelet:70 000/mL, AST: 71 U/L, ALT: 76 U/L, ALP: 340 U/L, GGT: 220 U/L, and other laboratory values were normal. Abdominal ultrasonography confirmed liver cirrhosis and detected twin pregnancy. She was closely monitored by gastroenterology and gynecology-obstetrics clinics. No ascites was detected at the last ultrasound at the 27th week of pregnancy. During the 32nd week of pregnancy, an emergency cesarean section was performed due to preterm birth. Breech/Cephalic presentation, 5-7/6-8 APGAR scores, and 1730/1380 g female/female live infants were delivered. No problems developed in the infants during follow-up. On the seventh day postpartum, massive ascites was detected on ultrasonography. The patient’s treatment was adjusted. On the ultrasound performed at the fourth week postpartum, it was observed that the ascites had decreased, and in follow-up, the ascites disappeared. Pregnant patients with cirrhosis have a high risk of ascites, hepatic encephalopathy, variceal bleeding, postpartum exacerbation, and maternal mortality. Variceal bleeding is the most feared complication, especially due to the increase in portal hypertension. The patient was regularly monitored during her pregnancy and developed ascites after childbirth. During the long-term follow-up, the ascites disappeared again.


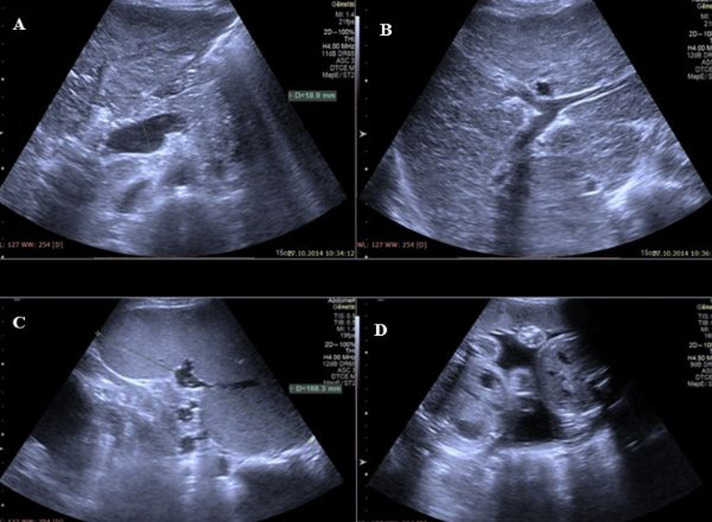


Figure 1. Abdominal ultrasonographic image showing heterogeneity of the liver parenchyma and enlargement of the portal vein (A), hepatomegaly (A, B), splenomegaly (C), and twin pregnancy (D) at 22 weeks of gestation.


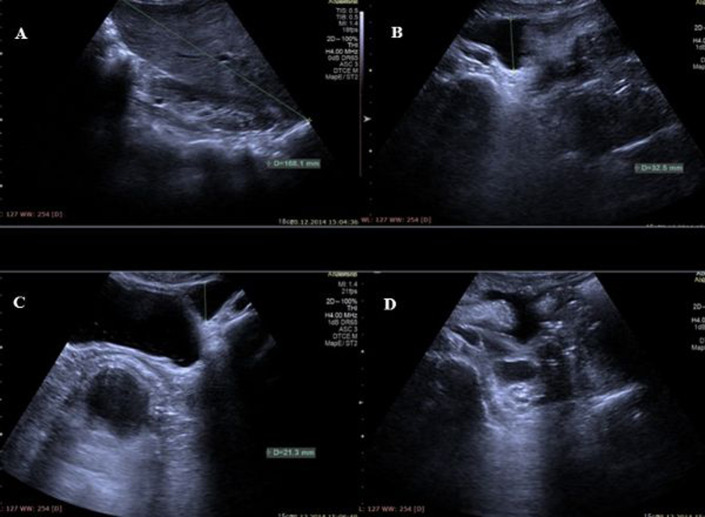


Figure 2. Ultrasonographic image of developing ascites during the first week postpartum.

## PS-154 A Rare Entity: Colonic Leiomyoma Accompanied by a Small, Asymptomatic Polyp

**Ömer Küçükdemirci**

Department of Gastroenterology, Hakkari State Hospital, Hakkari, Türkiye

Leiomyomas are benign neoplasms composed of well-differentiated smooth muscle cells, originating from the muscularis mucosae, muscularis propria, or vascular smooth muscle. Although they can arise anywhere in the gastrointestinal tract, they most frequently occur in the stomach and small intestine. In contrast, involvement of the large intestine is uncommon—approximately 3% of all GI leiomyomas—and, when present, they tend to localize to the left colon. Colonic leiomyomas are typically small, asymptomatic lesions that are often detected incidentally during routine endoscopic examinations. The findings of a 53-year-old patient who underwent colonoscopy for chronic diarrhea, during which 3 small polyps located at least 2 cm apart in the transverse colon were removed by polypectomy; histopathological evaluation revealed a leiomyoma (pic: 1-2-3) is presented. Immunohistochemical evaluation showed positivity for Desmin and SMA, a low Ki-67 index (1%-2%), and negativity for p53. Microscopic examination revealed a regular cell arrangement and low mitotic activity. Consequently, the tumor is assessed as a benign leiomyoma without malignant characteristics. In a subsequent colonoscopy, the sites of the polyps previously removed by polypectomy cannot be located again. Endoscopically similar lesions require distinct treatments, prognoses, and follow-up recommendations, necessitating accurate diagnosis. Endoscopists often rely on colonoscopy and histology for various cases, from routine cancer screenings to complex scenarios like colonic leiomyomas, which are rare.


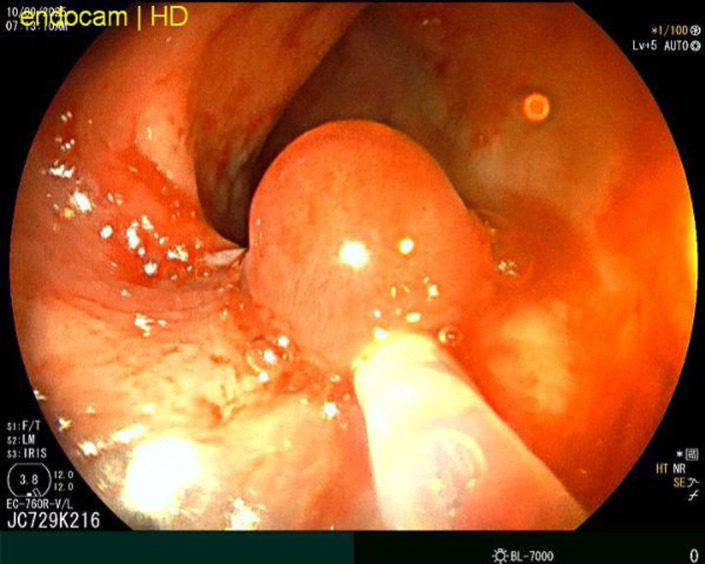


Figure 1. Polyp endoscopic view.


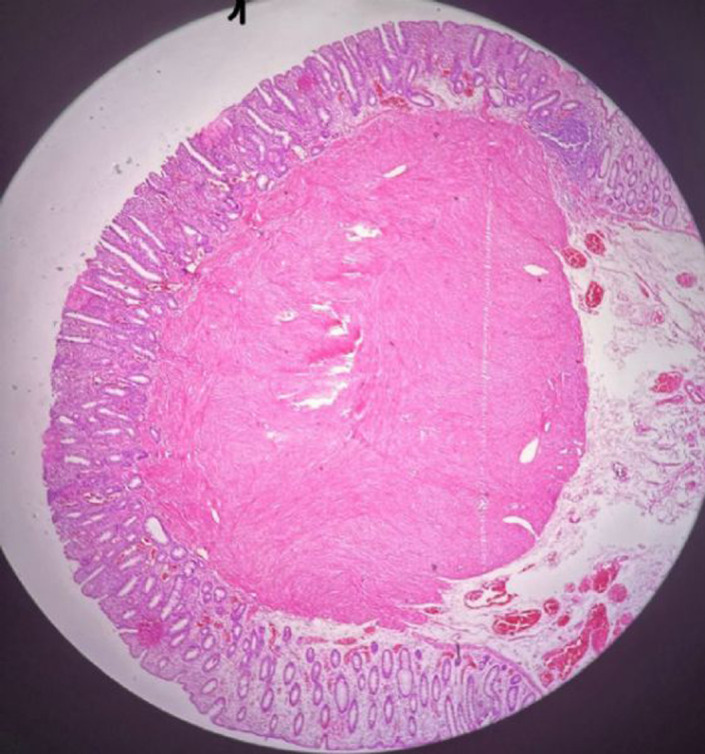


Figure 2. Leiomyoma microscopy.


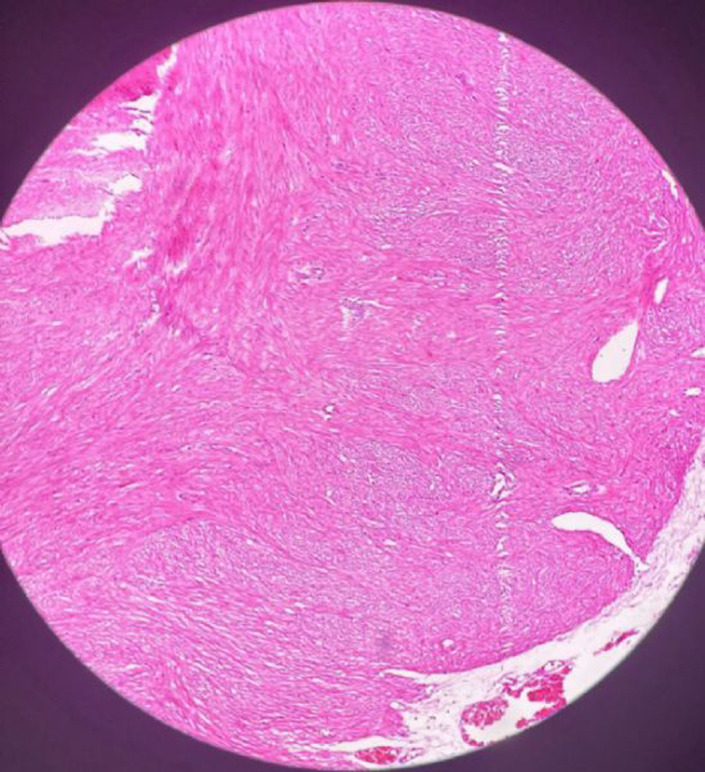


Figure 3. Leiomyoma microscopy.

## PS-155 High-Resolution Esophageal Manometry Findings—Motility Laboratory, Mersin University Faculty of Medicine Hospital

**Fehmi Ateş, Osman Özdoğan, Serkan Yaraş, Mehmet Kasım Aydın, Mammad Hasan Mammadov, Ümit Yeşilova, Ahmet Emre Ergan, Engin Altıntaş, Orhan Sezgin**

Department of Gastroenterology, Mersin University Faculty of Medicine, Mersin, Türkiye

Between January 1, 2022, and October 1, 2025, esophageal high-resolution manometry (HRM) data obtained at the Motility Laboratory of Mersin University Faculty of Medicine Hospital were retrospectively analyzed.

Indications for HRM included:

1. Nonobstructive dysphagia
2. Noncardiac chest pain (NCCP)
3. Preoperative evaluation before anti-reflux surgery
4. Postprocedural evaluation following Peroral Endoscopic Myotomy (POEM)

Dysphagia was defined as difficulty or discomfort during swallowing. Noncardiac chest pain (NCCP) was considered in patients presenting with retrosternal chest pain and a normal cardiovascular evaluation. Patients with a history of stricture, malignancy, or ulcers identified during endoscopy were excluded from the analysis.

A total of 990 patients underwent esophageal HRM, comprising 506 females (51.1%) and 484 males (48.9%), with a mean age of 46.4 ± 14.13 years and a median age of 47 years.

The distribution of HRM indications was as follows:

1.Dysphagia: 703 patients (71.0%)
2.Noncardiac chest pain: 69 patients (7.0%)
3.Preoperative evaluation for anti-reflux surgery: 60 patients (6.0%)
4.Post-POEM evaluation: 158 patients (16.0%)

Achalasia was the most common motility disorder, diagnosed in 406 patients (62.0%). Among those with achalasia, Type 2 was the most prevalent subtype, observed in 340 patients (84%), followed by Type 1 in 33 patients (8%) and Type 3 in 33 patients (8%).

Other motility disorders included:

1.Distal esophageal spasm: 28 patients
2.Absent contractility: 47 patients
3.Esophagogastric junction outflow obstruction: 84 patients

Among minor motility disorders, ineffective esophageal motility (IEM) was identified in 88 patients. Of the 60 patients evaluated before anti-reflux surgery, 9 (15.0%) had IEM and 5 (8.3%) had absent contractility. Among the 158 patients evaluated after POEM, 154 (97.7%) showed normalization of increased esophageal pressure values and resolution of dysphagia symptoms.

## PS-156 Allgrove Syndrome (Triple A)

**Vedat Göral**

Department of Gastroenterology, Medipol Mega Hospital, İstanbul Medipol University, İstanbul, Türkiye

Allgrove syndrome (AS), or 3A syndrome (AAA), is characterized by the triad of adrenocorticotropic hormone (ACTH)-resistant adrenal insufficiency, alacrimia, and achalasia, accompanied by progressive neurological impairment with or without mild intellectual disability. Here, a rare case of Allgrove (3A) Syndrome is presented. The patient was a 30-year-old woman living in Oman. Endoscopy revealed achalasia due to difficulty swallowing, and laboratory tests indicated adrenal insufficiency. Initially, endoscopy performed a bougie dilation for achalasia, followed by Heller myotomy. The difficulty swallowing persisted, albeit mildly. Treatment was initiated for adrenocortical insufficiency. Triple A, or Allgrove syndrome, is a hereditary syndrome primarily characterized by achalasia, alacrima, and adrenocorticotropin (ACTH) resistance addisonianism. In cases of achalasia, Triple A syndrome should not be overlooked, and adrenal insufficiency should also be investigated.

## PS-157 A Rare Case of Postlaparoscopic Cholecystectomy Obstructive Jaundice

**Mustafa Kaplan ^1^ , Nuh Berekatoğlu ^1^ , Nurgül Keskin ^1^ , Zeliha Serindağ ^1^ , Süleyman Dolu ^2^**

^1^Department of Gastroenterology, Sultan Abdulhamid II Training and Research Hospital, University of Health Sciences, İstanbul, Türkiye

^2^Department of Gastroenterology, Dokuz Eylül University, İzmir, Türkiye

In general surgery clinics, although rare, clips and materials applied to the cystic artery, vein, and duct during laparoscopic cholecystectomy may migrate into the bile ducts and the common bile duct (CBD). Case reports on this subject have been published in the literature. In this case, the aim was to evaluate a patient who presented to the emergency department with obstructive jaundice and abdominal pain 1 month after laparoscopic cholecystectomy. A 49-year-old male patient was admitted to the emergency department with complaints of right upper quadrant pain. He had a history of laparoscopic cholecystectomy performed 1 month earlier. Laboratory tests revealed increased transaminases, cholestatic enzymes, and bilirubin. Abdominal CT showed CBD dilatation with suspected sludge material and millimetric stones in the distal CBD. The patient underwent ERCP with a diagnosis of biliary obstruction. A small amount of stone and sludge material was removed from the CBD, and balloon-occluded cholangiography revealed faint linear filling defects within the CBD lumen. Balloon dilatation of the papilla was repeated, and further balloon sweeping of the CBD was performed. During this procedure, 2 surgical clips were retrieved. A repeat balloon-occluded cholangiogram showed no contrast leakage. For control purposes, a plastic stent was placed in the CBD, and the procedure was completed without complications. After ERCP, the patient’s abdominal pain resolved. Although rare, surgical clip and suture material migration into the CBD following laparoscopic cholecystectomy is a recognized complication, often related to biliary injury. It can lead to a wide spectrum of complications, ranging from biliary obstruction to cholangitis. Clip migration should be considered among the potential postoperative complications of laparoscopic cholecystectomy. These may not always be detectable, even with appropriate imaging modalities. Therefore, ERCP remains an effective and successful method for both diagnosis and treatment in such cases.


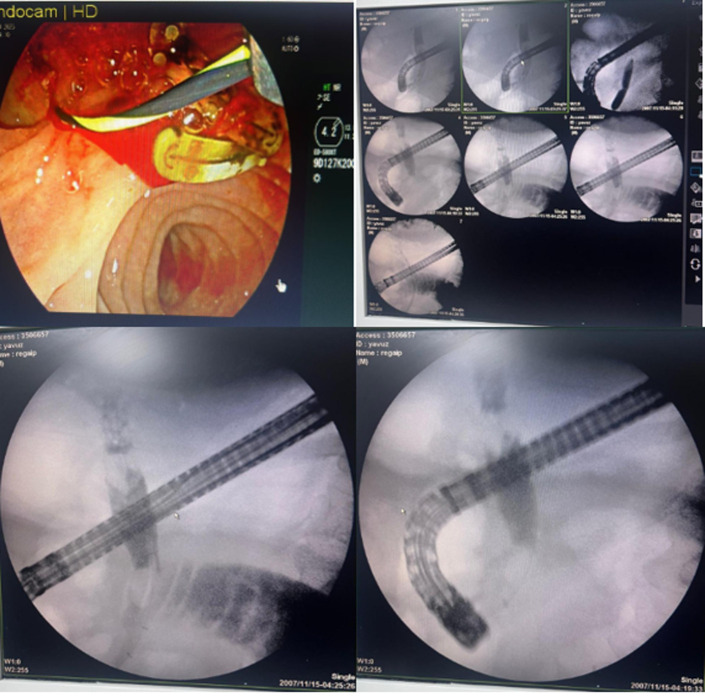


Figure 1. The clip is visualized alongside the guidewire.


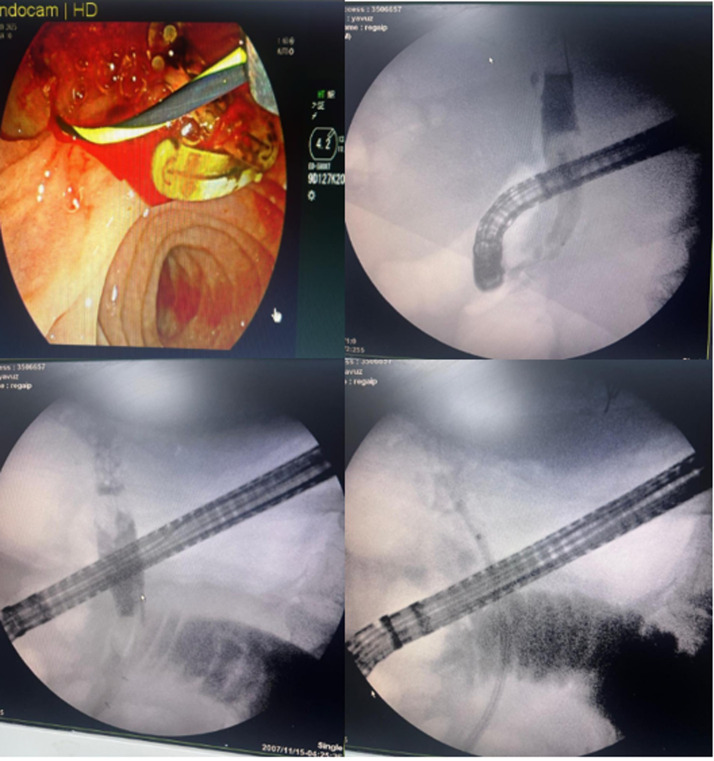


Figure 2. The common bile duct shows no evidence of filling defect or contrast leakage.

## PS-158 Esophagitis and Esophageal Stricture Associated with Edoxaban

**Enes Çelikmakas ^1^ , Özlem Gül ^2^ , Cemile Özsürekci ^3^ , Ufuk Yazar ^1^ , Hatice Azra Begüm Sarıoğlu Salimoğlu ^1^ , Hasan Kurt ^1^ , Bilal Ergül ^2^**

^1^Department of Internal Medicine, Lokman Hekim University Faculty of Medicine, Ankara, Türkiye

^2^Department of Gastroenterology, Lokman Hekim University Faculty of Medicine, Ankara, Türkiye

^3^Department of Geriatrics, Lokman Hekim University Faculty of Medicine, Ankara, Türkiye

Direct oral anticoagulants (DOACs) are widely prescribed for nonvalvular atrial fibrillation and thromboembolic events. Adverse effects, including DOAC-related esophagitis, have been reported. To date, only 1 case of edoxaban-associated esophagitis has been described. Here, a 74-year-old woman with dysphagia, chest pain, a sensation of obstruction, and malnutrition is presented. Edoxaban therapy had been initiated 1 week earlier for nonvalvular atrial fibrillation. Endoscopy revealed a circumferential ulcer with white exudate extending along the esophageal lumen at 17 cm from the incisors. The exudate peeled off as a membrane during advancement, and a stricture at 30 cm prevented scope passage. The distal part of the stricture was reached with a pediatric endoscope. Pathological evaluation excluded viral etiologies and malignancy. Edoxaban was discontinued, and treatment with low molecular weight heparin, a proton pump inhibitor, sucralfate, and an antacid was initiated. Follow-up endoscopies at 1-month intervals showed regression of the lesions.

The patient was lost to follow-up for 1 year and returned with recurrent dysphagia and approximately 40 kg weight loss. Edoxaban had been reintroduced during this period. Endoscopy demonstrated findings similar to the initial examination. A stent was placed at the stricture site to maintain passage and improve oral intake. This case represents one of the first reports of edoxaban-related esophagitis resulting in malnutrition and stricture. Reinitiation of edoxaban without follow-up led to progression of esophageal injury and severe weight loss. In conclusion, edoxaban may cause drug-induced esophagitis and stricture. Patients receiving DOACs who present with dyspeptic symptoms should also be evaluated for esophagitis. Patients diagnosed with esophagitis should be closely monitored until symptoms resolve and hospitalized for treatment if necessary. In patients lost to follow-up, symptoms may progress, leading to inadequate nutrition and malnutrition.


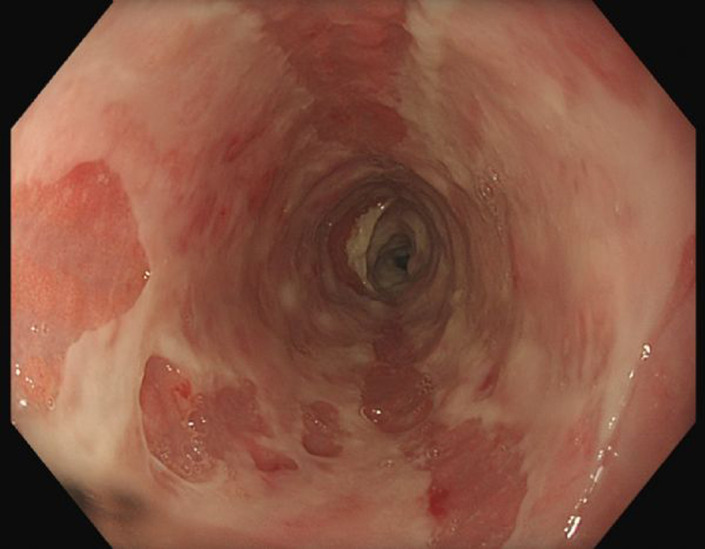


Figure 1. Endoscopy Image

Ulcer with white exudate starting from the proximal esophagus and extending all around the lumen.

## PS-159 Effectiveness of Partial Splenic Artery Embolization in Children with Hypersplenism and Variceal Bleeding

**Günsel Kutluk ^1^ , Fırat Kaya ^1^ , Özgür Kılıçkesmez ^2^**

^1^Department of Pediatric Gastroenterology, Başakşehir Çam ve Sakura City Hospital, University of Health Sciences, İstanbul, Türkiye

^2^Department of Interventional Radiology, Başakşehir Çam ve Sakura City Hospital, University of Health Sciences, İstanbul, Türkiye

Partial splenic artery embolization (PSAE) offers a valuable alternative for managing hypersplenism, preventing esophageal variceal bleeding, and avoiding splenectomy in children who are not surgical candidates. The short- and long-term outcomes of PSAE were evaluated in 17 pediatric patients with portal hypertension (PHT) and hypersplenism. Seventeen patients (10 males, median age 8 years; range, 1-18) underwent PSAE between 1 and 48 months before data collection (median follow-up: 25th-75th percentile, 2-36 months). Demographic features, clinical course, and complications were analyzed. Follow-up assessments included abdominal ultrasound, complete blood count, liver function tests, and endoscopy every 3 months. All patients presented with hypersplenism; 11 also had recurrent variceal bleeding. Underlying causes included extrahepatic PHT in 10 patients (6 with portal vein thrombosis) and intrahepatic PHT in 7 (biliary atresia in 3, Wilson’s disease in 2, Gaucher disease, and bile acid synthesis defect).

Early post-PSAE complications were mild, limited to fever, vomiting, and pain. The mean hospital stay was 9.4 days, prolonged in some cases due to preprocedural band ligation (n = 12). No severe complications such as sepsis, hematoma, or splenic abscess occurred.

Hematological parameters normalized in 15 of 17 patients; 2 showed recurrence during follow-up. The mean annual frequency of variceal bleeding decreased significantly, from 1.8 to 0.25 episodes (*P* < .05). Among 11 patients treated for recurrent variceal bleeding, 8 (72.7%) remained free of bleeding. Endoscopic follow-up revealed regression of esophageal varices in 8 of 11 (72.7%) evaluated patients. Three recent cases had not yet undergone repeat endoscopy. In conclusion, PSAE appears to be a safe and effective treatment for pediatric PHT. It improves hypersplenism, markedly reduces variceal bleeding, and offers a less invasive alternative to splenectomy for appropriately selected patients.

## PS-160 Case of Malignant Melanoma Presenting with a Single Giant Solitary Mass in the Liver

**Yekta Duygu Çimen Beşirli, Halil Yılmaz, Kamil Enli, Mustafa Çelik**

Department of Gastroenterology, Pamukkale University Faculty of Medicine, Denizli, Türkiye

Uveal malignant melanoma is the most common primary ocular malignancy in adults. Metastasis develops in nearly 50% of patients, most frequently involving the liver. Patients often present with hepatic symptoms such as abdominal pain, hepatomegaly, and jaundice. Although metastases typically occur within the first few years after diagnosis, late metastases have been reported. Therefore, lifelong follow-up is essential. A case of uveal malignant melanoma developing hepatic metastasis 20 years after the initial diagnosis is presented. A 62-year-old woman had undergone right eye enucleation 20 years earlier for uveal malignant melanoma and did not attend follow-up visits. No recurrence was reported. She presented to the emergency department with 1 week of abdominal pain and jaundice. Physical examination revealed right upper quadrant tenderness, hepatomegaly, and generalized jaundice, predominantly scleral. Laboratory tests showed increased direct bilirubin and lactate dehydrogenase. CT demonstrated hepatomegaly with a 21×12 cm hypodense liver lesion. MRCP revealed similar findings, suggesting either a metastatic or primary malignancy. Liver biopsy confirmed metastatic malignant melanoma. Large solitary hepatic lesions are usually considered primary liver tumors. In this case, a single giant hepatic mass occurred without extrahepatic disease. The remote melanoma history was initially overlooked due to the long interval. This case highlights that patients with prior uveal melanoma may develop hepatic metastasis even decades later, and large solitary liver masses, though rare, can represent metastases and must be included in the differential diagnosis.


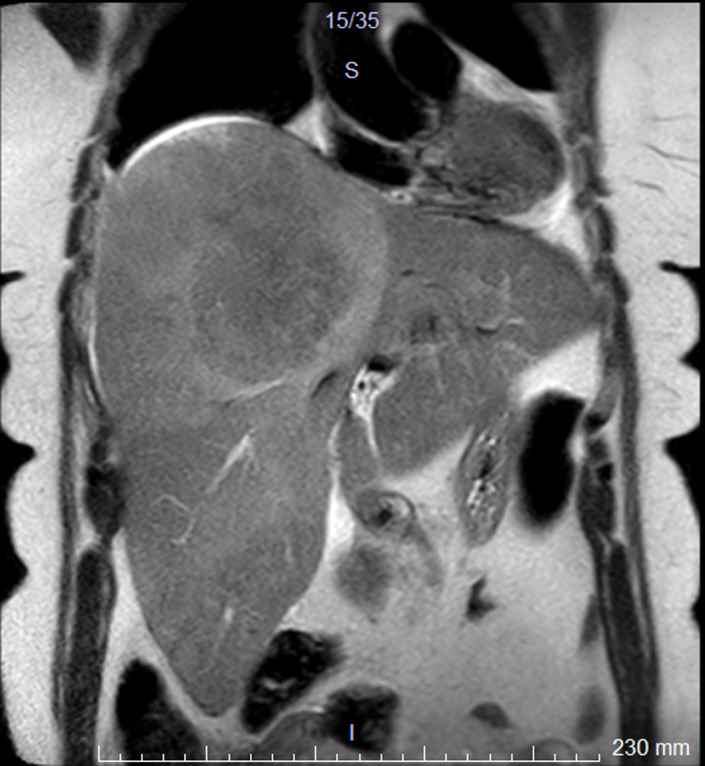


Figure 1. T2 sequence MRI image.

## PS-164 Results of Gastric Histopathological Examination in Patients with Situs Inversus Totalis (SIT): First Case Series in the Literature and Experience of Coastal Black Sea

**Gökhan Aydın ^1^ , Ahmet Cumhur Dülger ^1^ , Eray Beşirli ^2^ , İsmail Aydın ^3^**

^1^Department of Internal Medicine, Division of Gastroenterology, Giresun University Faculty of Medicine, Giresun, Türkiye

^2^Department of Internal Medicine, Giresun University Faculty of Medicine, Giresun, Türkiye

^3^Department of General Surgery, Giresun University Faculty of Medicine, Giresun, Türkiye

**Background/Aims:** There are no current studies comprehensively involving gastric diseases in situs inversus totalis (SIT). The aim of this study was to provide insight into the spectrum and prevalence of gastric histopathologic diseases in patients with SIT, even in a small number of case series.

**Materials and Methods:** A total of 9 patients with dyspepsia (6 females, with a mean age of 47.6 years for women and 60 years for men) over 18 years with SIT were enrolled in this retrospective observational study. Situs status and comorbidities were independently confirmed by 2 physicians, based on a review of radiologic, ultrasonic examination, operative records, and case notes.

**Results:** A total of 9 patients with dyspepsia confirmed to have SIT underwent upper gastrointestinal endoscopy at Giresun University Hospital’s endoscopy ward between October 2020 and October 2025. Two patients had difficulty accessing the second part of the duodenum. Duodenal and gastric biopsies were taken from the duodenum and antrum to detect pathological conditions including celiac disease, *Helicobacter pylori* infection, atrophic gastritis, intestinal metaplasia, and dysplasia. A total of 7 patients had *H. pylori* infection, and none were diagnosed with celiac disease, atrophic gastritis, intestinal metaplasia, or dysplasia.

**Conclusion:** The current study revealed gastric diseases in patients with SIT. A substantial proportion (77%) of dyspeptic patients with SIT had *H. pylori* infection. It was concluded that patients with SIT warrant careful examination for the presence of *H. pylori* infection. Furthermore, precancerous gastric disorders and gluten enteropathy were not detected in this unique patient group.

## PS-166 An Uncommon Cause of Spontaneous Bacterial Peritonitis: Sphingobacterium multivorum

**Pırıl Akıncıoğlu ^1^ , Saliha Yarımoğlu ^2^**

^1^Department of Gastroenterology, Karaman Training and Research Hospital, Karaman, Türkiye

^2^Department of Infectious Diseases, Karaman Training and Research Hospital, Karaman, Türkiye

Spontaneous bacterial peritonitis (SBP) is a common and serious complication in patients with cirrhosis, occurring without an intra-abdominal source of infection. Gram-negative enteric bacteria are the most frequent causative agents; however, rare and opportunistic pathogens can also be implicated. A case of SBP caused by *Sphingobacterium multivorum*, an environmental bacterium infrequently documented in human infections, is presented. A 76-year-old man with cryptogenic liver cirrhosis (Child-Pugh B) was admitted with several days of abdominal pain and distension. Physical examination revealed diffuse abdominal tenderness and massive ascites. Paracentesis showed ascitic fluid with a leukocyte count of 7.38 K/µL; the neutrophil percentage was not determined. Biochemical analysis revealed glucose 48 mg/dL, albumin 9.8 mg/dL, and total protein 17.5 mg/dL. Blood and ascitic fluid cultures were obtained, and empirical intravenous ceftriaxone (1000 mg every 12 hours) was initiated.

Within 20 hours, the patient developed anuria, rising serum creatinine, and metabolic acidosis, suggestive of hepatorenal syndrome. Diuretics were discontinued, and intravenous hydration with albumin replacement was started. A progressive INR increase and altered mental status indicated fulminant hepatic failure, necessitating ICU transfer. Despite intensive care, the patient’s condition worsened, requiring mechanical ventilation and inotropic support, and he ultimately died. Ascitic fluid culture later grew *Sphingobacterium multivorum*. The isolate formed low-convex, smooth, pale yellow colonies on blood agar. It was identified as a Gram-negative, oxidase- and catalase-positive, nonmotile bacillus using the VITEK II system. Antimicrobial susceptibility testing showed resistance to piperacillin-tazobactam, imipenem, amikacin, and aztreonam, but sensitivity to ciprofloxacin, meropenem, cefepime, and trimethoprim-sulfamethoxazole, interpreted according to EUCAST standards. *Sphingobacterium multivorum* is a rare human pathogen, usually affecting immunocompromised patients. Its isolation in SBP is extremely uncommon. This case highlights that atypical pathogens may cause severe infections with rapid deterioration and highlights the importance of considering rare organisms when empirical therapy fails.

## PS-168 Giant Mass Filling the Proximal Colon: Case Report

**Osman Bedir, Süleyman Coşgun**

Kütahya City Hospital Gastroenterology Clinic, Kütahya, Türkiye

The gastrointestinal tract is the most common extranodal site affected by lymphoma, accounting for 5%-20% of all cases. Primary gastrointestinal lymphomas account for only approximately 1%-4% of all gastrointestinal malignancies. Although lymphoma can arise from virtually any region of the gastrointestinal tract, the most frequently affected sites in terms of incidence are the stomach, followed by the small intestine and the ileocecal region. Mantle cell lymphoma is a rare lymphoma of the gastrointestinal tract and can present as multiple lymphomatous polyposis. Here, a case of a giant lymphoma that almost completely obstructed the lumen in the proximal colon is presented. An 83-year-old male patient was scheduled for endoscopy and colonoscopy due to iron deficiency anemia. Colonoscopy revealed a giant mass lesion, almost completely filling the lumen from the hepatic flexure to the base of the cecum, with an irregular surface and occasional exudates. Biopsies taken from the mass were examined and found to be consistent with mantle cell lymphoma. A CT scan of the abdomen and pelvis revealed a 14 cm heterogeneous mass in the ascending colon. A PET-CT scan revealed widespread lymphadenopathy in the mediastinum and abdomen, which was diagnosed as grade-4 mantle cell lymphoma. The patient, who had no signs or symptoms of obstruction, was referred to the hematology department for R-benda treatment. Colorectal lymphoma accounts for a small proportion of both colorectal malignancies and gastrointestinal lymphoma. Most patients present with nonspecific symptoms, often leading to delays in diagnosis and an advanced stage at onset. Treatment generally includes chemotherapy, radiation, surgery, or a combination; whereas surgery is the mainstay of treatment for colon adenocarcinoma. In aggressive lymphomas, resection helps control local disease to prevent obstruction and perforation, but definitive treatment remains limited to CHOP or other polychemotherapy. Unfortunately, despite aggressive treatment, disease recurrence occurs, and most patients with colorectal lymphoma die from the disease.


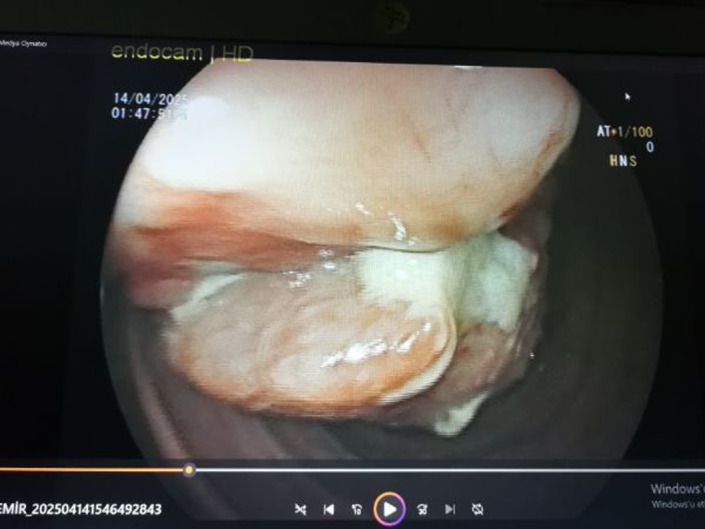
 
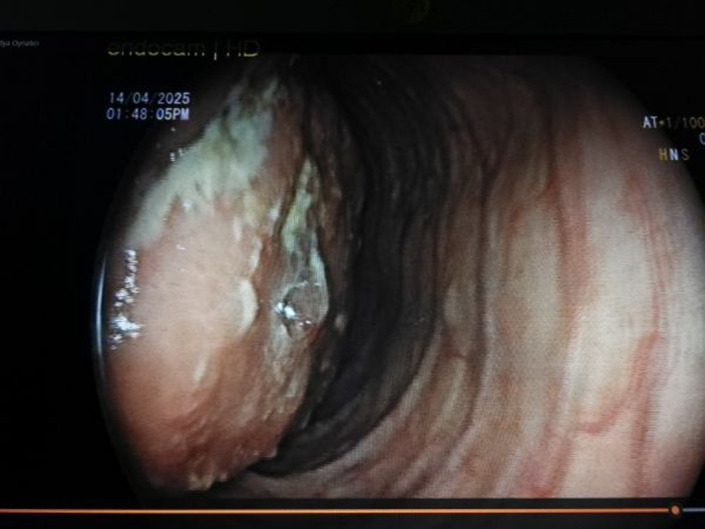
 
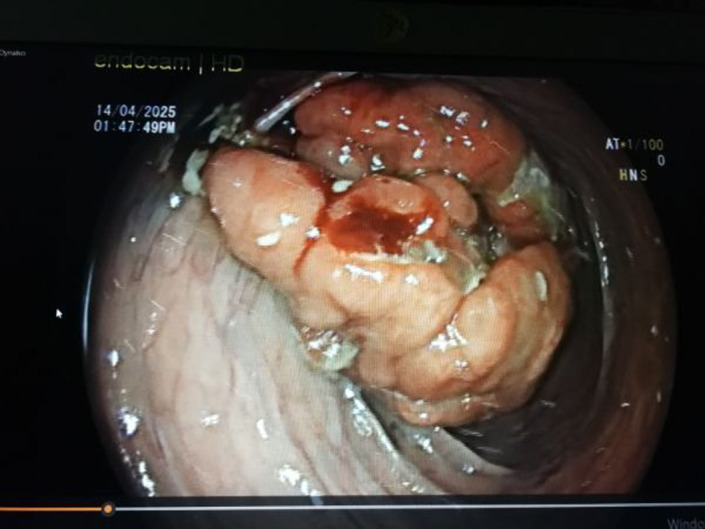


Figure 1. Lymphoma.

## PS-170 Osler-Weber-Rendu Syndrome: A Case Report

**Osman Bedir, Süleyman Coşgun**

Kütahya City Hospital Gastroenterology Clinic, Kütahya, Türkiye

OWRS is an AD disease characterized by vascular telangiectasias in the skin and mucosa. It can present as red papules in the mouth, nose, and oropharynx. Dilated telangiectatic vessels are present on the lips, cheeks, and tongue. Recurrent hemorrhages are observed. Large AVMs can occur in the lungs, liver, and brain. Anemia may develop following gastrointestinal and urinary bleeding. Histopathology reveals superficial, thin-walled vascular structures. Here, a case of OWRS with widespread telangiectasias observed during endoscopy and colonoscopy performed for anemia is reported.

A 28-year-old woman was evaluated for iron deficiency anemia, and endoscopic screening revealed telangiectatic lesions on the lips and cheeks, as well as intraoral telangiectasias. The patient experiences occasional epistaxis and is under gynecological follow-up due to hypermenorrhea. Endoscopy revealed widespread telangiectatic lesions, particularly in the bulb, second portion of the duodenum, and stomach. Colonoscopy also revealed rare but intermittent telangiectatic foci throughout the colon. APC was performed on the larger foci. All clinical findings were evaluated together, leading to the conclusion that this was OWRS. Because this is a new case, further investigation of other possible findings (AVM, cavernous malformation, etc.) and organ scans have not yet been performed. OWRS, characterized by abnormal angiogenesis and thinning of vessels, clinically presents with red to purple telangiectasias in many parts of the body, including the oral mucosa, lips, face, ears, and fingers. However, recurrent nosebleeds and a positive family history play an important role in the differential diagnosis. Sometimes, telangiectasias with or without bleeding are observed in internal organs. Treatment plans are determined by the location of telangiectasias and whether they are symptomatic. APC was administered to large lesions likely to bleed due to anemia, and IV iron was also given. Approximately 10%-15% of patients with HHT have cavernous angiomas, AVMs, and aneurysmal cerebrovascular malformations. The optimal treatment option (neurovascular surgery, embolization, and stereotactic radiosurgery) for these types of cerebrovascular malformations, especially those that are asymptomatic, remains controversial.


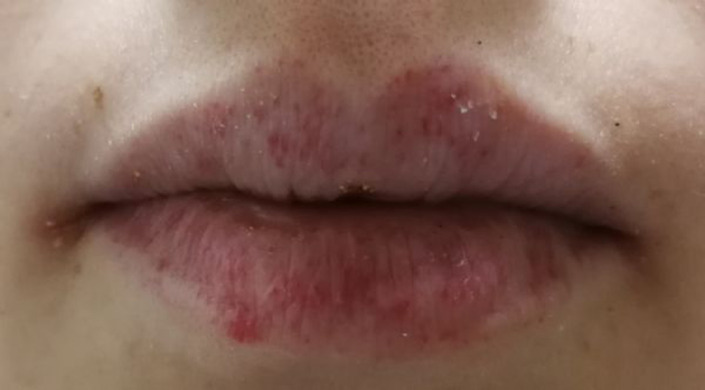
 
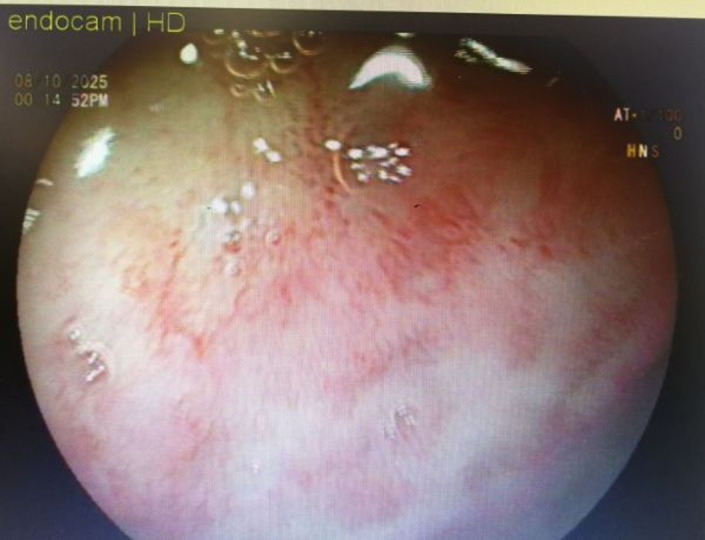
 
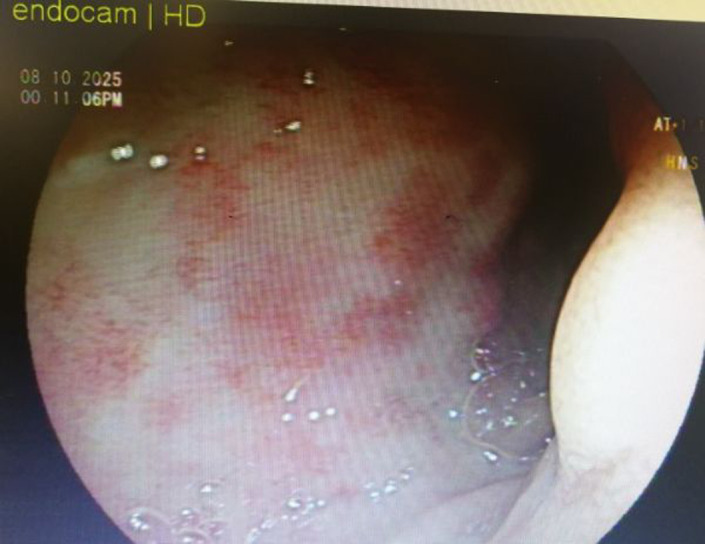


Figure 1. Lips of the patient and the endoscopy.

## PS-171 Celiac Disease Diagnosed at An Advanced Age: A Case Report

**Sakına Gahramanova ^1^ , Direnç Yiğit ^2^**

^1^Department of Internal Medicine, Antalya Private OFM Hospital, Antalya, Türkiye

^2^Department of Gastrointestinal Surgery, Antalya Private OFM Hospital, Antalya, Türkiye

Celiac disease is a proximal small intestine disorder that develops in genetically predisposed individuals after the ingestion of gluten. In recent years, it has been reported that celiac disease is more prevalent in adults than in children. Among adults, the age of diagnosis varies between 40 and 60 years. With this case, the aim was to demonstrate that the disease can occur in all age groups. A 61-year-old male patient presented to the outpatient clinic with complaints of intermittent diarrhea for many years, weight loss, postprandial bloating and gas, and marked fatigue. On physical examination, the skin appeared pale, and there was tenderness in all quadrants of the abdomen upon deep palpation. Laboratory tests revealed: HGB 11 g/dL, MCV 66 fL, Ferritin 6 µg/L, Vitamin B12 115 µg/L. Liver and kidney function tests were within normal limits.

Esophagogastroduodenoscopy (EGD): Findings were consistent with atrophic gastritis. The mucosa of the third part of the duodenum appeared hypertrophic with a cobblestone pattern.

Colonoscopy: A polyp was detected in the sigmoid colon and removed via polypectomy.

Abdominal contrast-enhanced CT scan: Sections at the hypogastric level revealed reactive mesenteric lymph nodes, the largest measuring approximately 14 × 8.5 mm.

Duodenal biopsy: Reported findings of chronic duodenitis and severe villous atrophy were consistent with celiac disease.

The patient was referred to a dietitian for a gluten-free diet, and nutritional deficiencies were addressed through appropriate supplementation. A definitive diagnosis of celiac disease is established through a small intestine biopsy. In adult patients, the classic clinical presentation is rare. Instead, the more frequently encountered form is “atypical celiac disease,” which presents with extraintestinal symptoms.

## PS-172 Mesenchymal Stem Cell Transplantation in Perianal Fistulizing Crohn’s Disease: A Single-Center Experience

**Özge Koç ^1^ , Volkan Yılmaz ^1^ , Amed Trak ^1^ , İbrahim Ethem Geçim ^2^ , Murat Törüner ^1^**

^1^Department of Gastroenterology, Ankara University School of Medicine, Ankara, Türkiye

^2^Department of Surgery, Ankara University School of Medicine, Ankara, Türkiye

**Background/Aims:** Perianal fistulas occur in approximately 4%-10% of patients with Crohn’s disease and represent a challenging complication that significantly impairs quality of life. As the duration of the disease increases, the prevalence of perianal fistulizing disease also rises. Despite the use of advanced medical therapies, the success rate in patients with fistulizing Crohn’s disease remains low. Thus, mesenchymal stem cell therapy was evaluated in patients with fistulizing Crohn’s disease who did not respond to conventional treatment.

**Materials and Methods:** Three male patients followed at the Gastroenterology Department of Ankara University School of Medicine were included. All patients underwent a detailed preoperative evaluation, including rectal examination under general anesthesia and magnetic resonance imaging (MRI) to characterize the anatomical features of the fistula tracts and sinuses. After obtaining informed consent and approval from the Ministry of Health of the Republic of Türkiye, 60 million Wharton’s jelly-derived MSCs were injected into each sinus. Clinical evaluations were conducted at baseline and at weeks 1, 2, 4, 8, and 24 using the Crohn’s Anal Fistula Quality of Life (CAF-QoL) questionnaire, Harvey-Bradshaw Index, Perianal Disease Activity Score, Inflammatory Bowel Disease Questionnaire (IBDQ), and C-reactive protein (CRP) levels. Control MRI was performed at 2 months, and follow-up imaging was planned for 1 year.

**Results:** In the MRI evaluations performed 2 months after treatment, varying degrees of improvement were observed in the patients’ fistula tracts; however, no complete healing of the fistula tracts was detected in any patient by the end of the second month. A decrease in CRP levels was observed in all 3 patients following the procedure. The patients’ questionnaire assessments revealed heterogeneous responses.

**Conclusion:** Wharton’s jelly-derived MSC therapy showed early partial radiological and biochemical improvement in biologically refractory complex perianal fistulizing Crohn’s disease. Larger, long-term, controlled studies are warranted to clarify its efficacy and clinical role.

## PS-173 Prognostic Impact of Endoscopic Mayo Score 0 and 1 in Patients with Ulcerative Colitis in Clinical Remission: A Single-Center Retrospective Study

**Şeyma Şenocak ^1^ , Mukaddes Tozlu ^2^**

^1^Department of Hematology, Sakarya University Training and Research Hospital, Sakarya, Türkiye

^2^Department of Gastroenterology, Sakarya University Faculty of Medicine, Sakarya, Türkiye

**Background/Aims:** Ulcerative colitis (UC) is a chronic inflammatory bowel disease characterized by alternating remission and relapse. Although clinical and endoscopic remission are key goals, a Mayo endoscopic subscore (MSe) of 0 or 1 is generally regarded as mucosal healing. However, the prognostic differences between MSe 0 and MSe 1 remain unclear.

This study aims to assess the impact of MSe 0 and 1 on relapse risk and remission duration in patients with UC who are in clinical remission.

**Materials and Methods:** A total of 157 patients with UC who underwent colonoscopy during clinical remission between January 2009 and December 2023 were retrospectively analyzed. Inclusion criteria included a confirmed diagnosis based on clinical, endoscopic, and histopathological findings, colonoscopy in remission, and at least 12 months of follow-up. Patients were categorized as MSe 0 (n = 83) or MSe 1 (n = 74). Clinical relapse was defined as a partial Mayo score ≥2, treatment modification due to worsening findings, or hospitalization/surgery related to disease activity. Patients were followed until relapse or the end of the study. Relapse rates were compared using Kaplan–Meier analysis and the log-rank test. Independent predictors were determined by Cox regression.

**Results:** During follow-up, 42 patients (26.5%) relapsed. The relapse rate was significantly higher in the MSe 1 group compared to the MSe 0 group (40.5% vs. 14.4%, *P* = .010). Mean remission duration was 29.4 ± 16.3 months in MSe 1 and 43.6 ± 25.1 months in MSe 0 (*P* = .036). Kaplan–Meier analysis showed an earlier decline in relapse-free survival for MSe 1, persisting throughout the follow-up period. Multivariate analysis identified MSe 1 as the only independent predictor (OR = 4.0, 95% CI: 1.8-8.6, *P* = .001).

**Conclusion: **Among patients with UC in clinical remission, MSe 1 was associated with shorter remission duration and a higher risk of relapse. This finding suggests that MSe 1 reflects residual inflammation even during the clinical remission period and serves as an independent predictor of relapse. Achieving complete mucosal healing (MSe 0) should be a primary therapeutic goal.


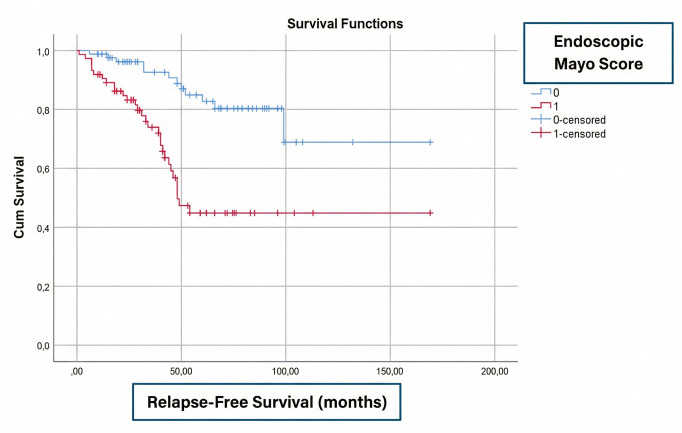


Figure 1. Kaplan–Meier relapse-free survival curves in MSe 0 and MSe 1 groups.

## PS-174 A Rare Diagnosis in Pancreatic Head Masses: Extramedullary Plasmacytoma

**Talha Ercan, Yunus Emre Demiral, İlyas Ethem Şenocak, Yunus Günegül, Mustafa İhsan Uslan**

Department of Gastroenterology, Sakarya University Faculty of Medicine, Sakarya, Türkiye

Pancreatic head masses are most commonly associated with adenocarcinoma and are therefore considered the primary diagnosis by clinicians. Less frequent etiologies include lymphoma, metastasis, and plasmacytoma. Extramedullary plasmacytoma accounts for approximately 3% of plasma cell neoplasms and most often arises in the upper respiratory tract mucosa. Pancreatic involvement is exceedingly rare, with only a limited number of cases reported. A case of extramedullary plasmacytoma manifesting as a pancreatic head mass is presented. A 66-year-old man presented with pruritus and jaundice. Laboratory evaluation revealed cholestatic liver test abnormalities and increased CRP. Abdominal ultrasonography demonstrated a 12 mm dilatation of the common bile duct (CBD) with a distal appearance suggestive of a stone, prompting the patient to undergo ERCP. During ERCP, it was noted that he had a prior gastroenterostomy. The scope was advanced to the papilla, and cholangiography revealed a nearly 2-cm narrowed segment in the middistal CBD. A 10 Fr plastic stent was placed. Contrast-enhanced CT showed marked CBD dilatation and a solid mass of ~3 cm in the pancreatic head. On FDG-PET/CT, the lesion showed no significant uptake, and a DOTA-PET scan revealed no definitive uptake. Given these findings, the patient underwent endoscopic ultrasound (EUS). EUS demonstrated a 3 cm irregular solid lesion in the pancreatic head, from which 2 passes of fine-needle biopsy were obtained. Histopathology revealed CD138 and kappa light-chain positivity, confirming extramedullary plasmacytoma. The patient was discussed in a multidisciplinary tumor board and referred to hematology. Systemic disease was excluded, and local radiotherapy was planned. Although rare, extramedullary plasmacytoma should be considered in the differential diagnosis of pancreatic head masses. It can clinically and radiologically mimic adenocarcinoma, highlighting the importance of tissue diagnosis for accurate management.

## PS-175 Duodenal Invasion Secondary to Metastatic Periportal Lymphadenopathy from Hepatocellular Carcinoma: A Rare Cause of Upper Gastrointestinal Bleeding

**Zeynep Melekoğlu Ellik, Özge Koç, Volkan Yılmaz, Amed Trak, Ramazan Erdem Er, Hale Gökcan, Ramazan İdilman**

Department of Gastroenterology, Ankara University School of Medicine, Ankara, Türkiye

Hepatocellular carcinoma (HCC) frequently metastasizes to the lungs and regional lymph nodes, but direct invasion of the gastrointestinal (GI) tract is exceedingly rare. Duodenal involvement, particularly secondary to metastatic lymphadenopathy, represents an uncommon and diagnostically challenging source of upper GI bleeding in advanced HCC. A 64-year-old man with chronic hepatitis B infection, without cirrhosis, was under surveillance for HCC. He underwent partial hepatectomy for an LI-RADS 5 lesion in hepatic segment VIII with periportal lymphadenectomy in October 2023. Follow-up imaging revealed progressive periportal and para-aortic lymphadenopathy with invasion of the duodenal wall. The patient presented with melena and severe anemia (hemoglobin: 5.3 g/dL). Esophagogastroduodenoscopy demonstrated an ulceroinfiltrative mass involving the duodenal bulb and extending into the second portion of the duodenum. Histopathology confirmed poorly differentiated malignant infiltration consistent with metastatic HCC. Considering the extensive disease and poor performance status, curative treatment was not feasible, and best supportive care was initiated. Duodenal invasion secondary to metastatic periportal lymphadenopathy is an exceptionally rare but clinically significant manifestation of HCC. It should be considered in the differential diagnosis of upper GI bleeding in patients with advanced liver malignancy. Early recognition and multidisciplinary management are essential for accurate diagnosis and optimal palliative care.

## PS-177 A Rare Cause of Lower Gastrointestinal System Bleeding

**Sevinç Tuğçe Güvenir**

Department of Gastroenterology, Batman Education and Research Hospital, Batman, Türkiye

Gastrointestinal stromal tumors (GISTs) are a rare cause of lower gastrointestinal (GI) bleeding. This study aimed to review the management of GISTs and lower GI bleeding based on the case of a patient who was evaluated in the emergency department with melena and diagnosed with jejunal GIST.


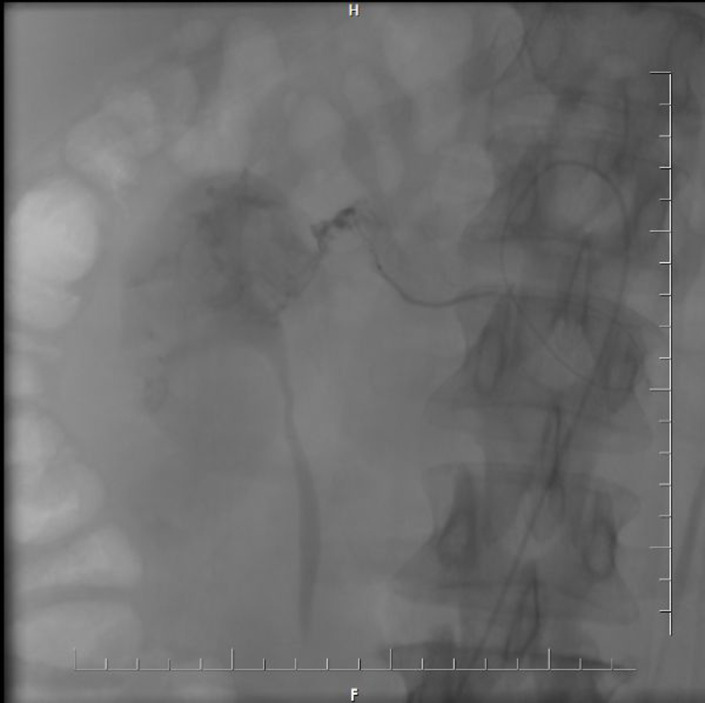


Figure 1. Jejunal GIST image on angiograph.


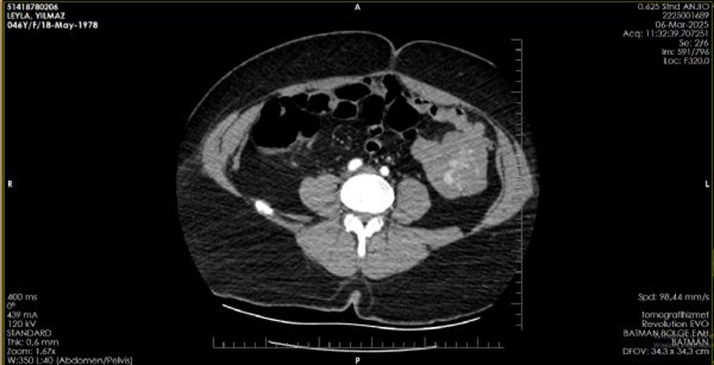


Figure 2. Jejunal GIST image on computed tomography.

## PS-179 Effect of Cholecystectomy on Preventing Recurrence of Acute Pancreatitis

**Gülseren Seven ^1^ , Günel Musayeva ^2^ , Sercan Kiremitçi ^2^ , Ali Tüzün İnce ^2^ , Elmas Biberci Keskin ^3^ , Hakan Şentürk ^1^**

^1^Department of Gastroenterology, Zincirlikuyu Medicana Hospital, Istanbul, Türkiye

^2^Department of Gastroenterology, Bezmialem Foundation University Faculty of Medicine, Istanbul, Türkiye

^3^Department of Internal Medicine, University of Health Sciences Şişli Etfal Hamidiye Training and Research Hospital, Istanbul, Türkiye

**Background/Aims:** Current guidelines recommend cholecystectomy in patients with acute biliary pancreatitis (ABP) to prevent recurrence. Some studies suggest that a significant portion of patients with idiopathic acute pancreatitis (IAP) results from occult biliary disease, and cholecystectomy after an episode of IAP reduces the risk of recurrent pancreatitis. This study aimed to ascertain whether cholecystectomy can prevent pancreatitis recurrence in patients with ABP and IAP.

**Materials and Methods:** Adult patients with their first episode of acute pancreatitis (AP) who were admitted to the inpatient clinic between January 1, 2015,and December 31, 2021, at a tertiary referral center were retrospectively reviewed. AP was diagnosed based on revised Atlanta criteria. Patients with biliary and idiopathic AP were included in the study. Pancreatic, neoplastic, traumatic, hypertriglyceridemia, autoimmune, drug-induced, and other etiologies were excluded. Gallstone pancreatitis was diagnosed if gallstones and/or biliary sludge were confirmed by abdominal US, EUS, and/or ERCP. IAP was diagnosed after extensive workup, including EUS and/or MRCP.

**Results:** A total of 500 patients with their first episode of AP (300 patients with ABP and 200 patients with IAP) were enrolled. The mean age was 58 (range, 20-86) years; 52% were female. The patients were divided into 3 different groups: Group 1, patients who had their first pancreatitis attack after cholecystectomy; Group 2, patients who underwent cholecystectomy after the first episode of pancreatitis; Group 3, patients who had their gallbladder in situ during the follow-up. The number of patients with recurrence after the first episode of AP was 17% in Group 1, 19% in Group 2, and 25% in Group 3 (19% and 35%, respectively). Recurrence in Group 3 of patients with ABP was significantly higher compared to the other groups (*P* < .001).

**Conclusion:** In ABP, the recurrence is high in patients who have their first pancreatitis attack after cholecystectomy. Cholecystectomy alone is not effective in preventing recurrence in patients with the gallbladder in situ during the first episode; however, the risk can be decreased by adding ERCP to cholecystectomy. On the other hand, cholecystectomy is not effective in reducing recurrence in patients with IAP.

## PS-180 A Case of Sarcoidosis Mimicking Peritoneal Carcinomatosis

**Mesut Kuş, Serhat Akpınar, Kadir Gişi, Bülent Kantarçeken**

Department of Internal Medicine, Devision of Gastroenterology, Sütçü İmam University Faculty of Medicine, Kahramanmaraş, Türkiye

Sarcoidosis is a systemic inflammatory disease of unknown cause that can affect nearly any organ and is characterized by the formation of noncaseating granulomas. In this case report, a case of sarcoidosis, a significant mimicker, is discussed, who presented with abdominal distension and was diagnosed with peritoneal carcinomatosis on computed tomography. A 58-year-old woman presented to the outpatient clinic with complaints of abdominal distension. Abdominal ultrasound revealed ascites, and she was admitted to the ward for further investigation of the etiology. Cardiac and liver pathologies were excluded. Initially, a diagnostic paracentesis was performed. The fluid examination revealed a serum-ascites albumin gradient of <1.1, fluid cultures were negative, and histological examination showed no malignant cells. Consequently, the patient underwent upper endoscopy and colonoscopy, both of which yielded normal results. Subsequently, a full-abdominal CT scan was performed to determine the etiology. The CT scan revealed diffuse fluid in the abdomen and increased reticulonodular density in the anterior wall of the mesentery (suggestive of peritoneal carcinomatosis). The patient underwent a laparoscopic biopsy, which revealed nonnecrotizing granulomatous inflammation, with granulomas lacking lymphocytes and containing calcific material (Schaumann bodies) within some giant cells. An angiotensin-converting enzyme (ACE) level was obtained, with a result of 154.7 U/L (normal range: 8-52 U/L). Based on these results, the patient was diagnosed with peritoneal involvement due to sarcoidosis and secondary ascites, and treatment for sarcoidosis was initiated. Clinical findings showed significant improvement, and follow-up treatment is currently ongoing. Peritoneal involvement is an uncommon manifestation of sarcoidosis, especially in the absence of splenic, hepatic, adnexal, or small bowel involvement. Peritoneal sarcoidosis is difficult to distinguish clinically and radiographically from peritoneal carcinomatosis or peritoneal tuberculosis. In these patients, a laparoscopic peritoneal biopsy is recommended, if possible, to document nonnecrotizing granulomas and to exclude malignancy and infection.

## PS-181 A Combined Sphincterotome for ERCP: Initial Experience

**Selman Çelebi, Zeynep Gök Sargın, Leyla Yalçın, Furkan Elciler, Cemile İlkem Güç, Gülçin Koca**

Department of Gastroenterology, University of Health Science, Antalya City Hospital, Antalya, Türkiye

Cannulation in ERCP typically begins with a standard sphincterotome, followed by sphincterotomy using the same device. In difficult cases, repeated use of both needle-knife and standard sphincterotomes, along with catheter and guidewire exchanges, can prolong the procedure and impair visibility due to bleeding during the precut phase. A device that combines both functions may improve procedural efficiency. This study introduces a triple-lumen “combined sphincterotome” capable of performing both standard and needle-knife sphincterotomy. It includes 2 cutting wires, each controlled by independent sliders on the handle. The cautery plug connects to the selected wire, ensuring only 1 is energized at a time. The third lumen accommodates both guidewire insertion and contrast injection. A newly designed single-handed “quick valve” prevents backflow when the guidewire is in place, allowing fluid injection without displacement.

Four patients with choledocholithiasis, who failed biliary cannulation with a standard sphincterotome and had no papillary anomalies, diverticula, or cholangitis, were treated using the combined device. Needle-knife access succeeded on the first attempt in 3 cases and after several attempts in 1. Deep cannulation was performed over the guidewire, followed by cholangiography and completion of sphincterotomy using the standard blade. No complications occurred. This novel device may reduce procedure time in precut cases by minimizing exchanges. Initial results suggest the combined sphincterotome eliminates the need to switch devices after precut. Further controlled studies are needed to confirm its safety and efficacy.


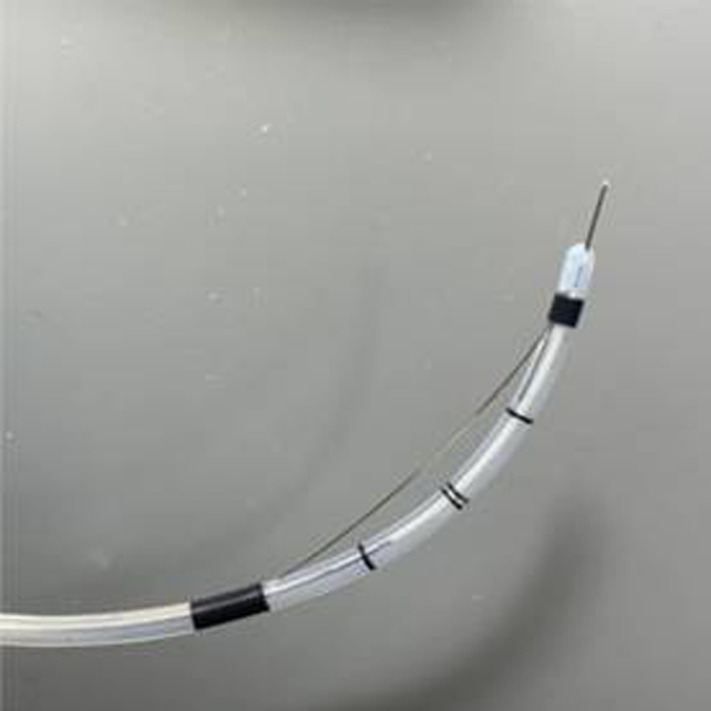


Figure 1. Needle-knife function.


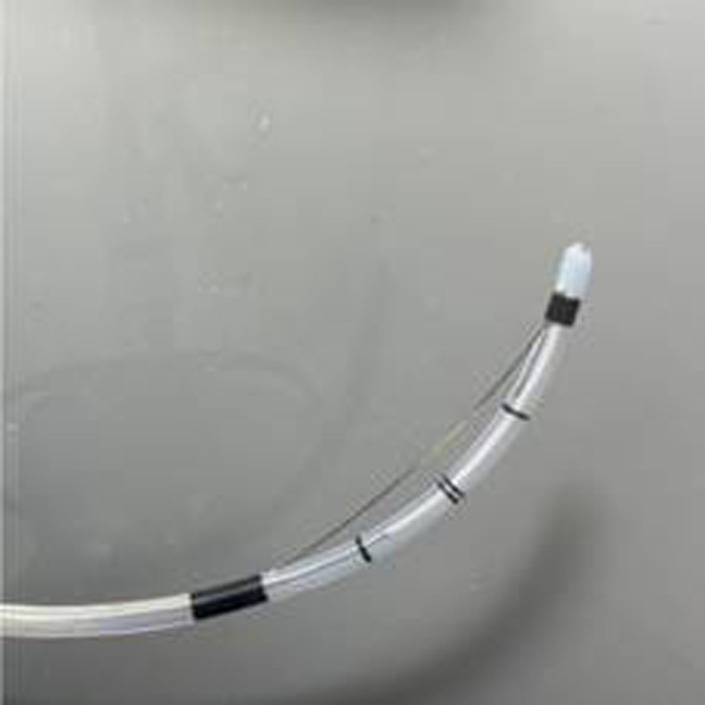


Figure 2. Standard (pull-type) function.


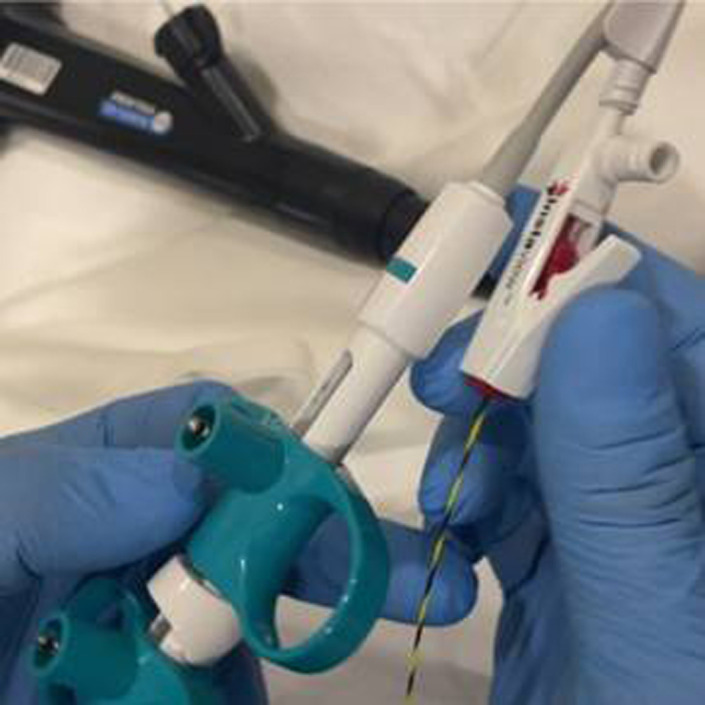


Figure 3. Handle. Each blade has a separate electrical connection. The “quick valve adapter” prevents contrast fluid leakage from the guidewire entry.

## PS-184 A New Design: Fully Closable Retrieval Net

**Selman Çelebi, Zeynep Gök Sargın, Cemile İlkem Güç, Gülçin Koca, Leyla Yalçın, Furkan Elciler**

Department of Gastroenterology, University of Health Science, Antalya City Hospital, Antalya, Türkiye

**Background/Aims:** Conventional retrieval nets lose volume during closure as the net material retracts into the tube. This can crush large polyps or fail to retain elongated foreign bodies. A novel retrieval net design aims to eliminate volume loss during closure, improving specimen integrity and retrieval reliability. This study aims to evaluate the safety and effectiveness of a fully closable retrieval net (MD Craft®, FIL-23-2545, 25×45 mm) in removing colorectal polyps larger than 15 mm.

**Materials and Methods:** Seventeen colonoscopic polypectomy cases involving polyps >15 mm were analyzed. Patients had a mean age of 57 (range, 44-81), with 11 males. Four patients had multiple large polyps (2-4 each); the remainder had 1. Endoscopic mucosal resection (EMR) was performed in 3 cases. No complications related to polypectomy or EMR occurred. All specimens were retrieved using the same fully closable net device.

**Results:** Specimens were successfully captured and removed along with the colonoscope. In multipolyp cases, all samples were retrieved in a single session. No adverse events, device malfunctions, or specimen damage were observed. Macroscopic morphology was preserved in all cases.

**Conclusion:** The device’s unique mechanism—where the net slides toward a fixed distal ring without retracting into the tube—forms a closable pouch that maintains its full volume. A string-linked proximal edge allows for controlled release. This design enables the safe retrieval of tissues up to 30 mm in diameter (~14 cm^3^) without crushing. It may also reduce the risk of foreign body aspiration during upper gastrointestinal procedures.


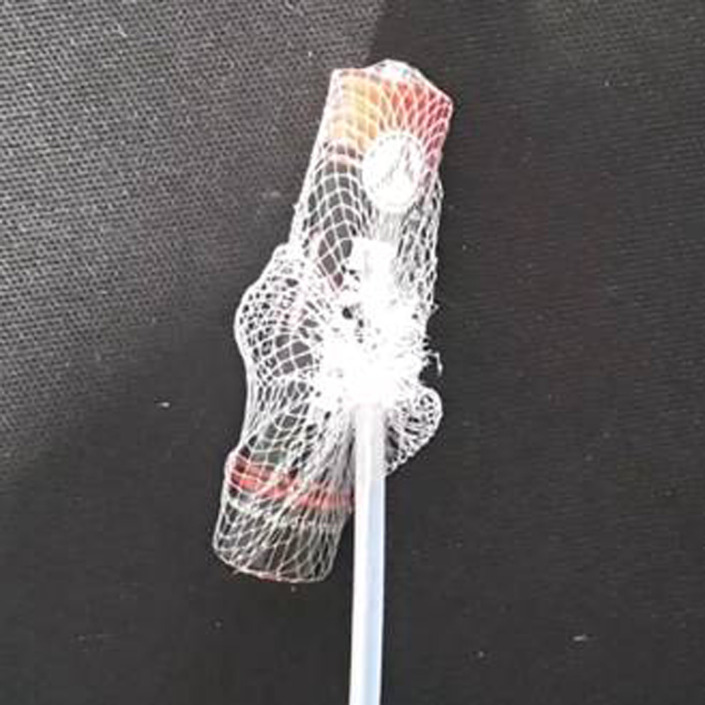


Figure 1. A 45 × 25-mm net enclosing an AA battery.


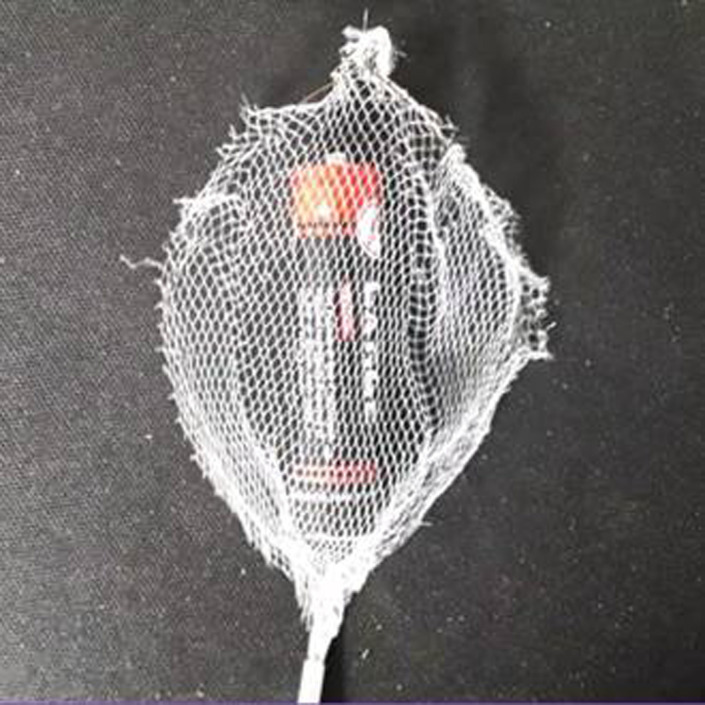


Figure 2. A battery was selected as a model foreign object.

## PS-185 Endoscopic Mucosal Resection and Hybrid Endoscopic Submucosal Dissection in Submucosal Gastric Lesions: A Single-Center Experience

**Ozan Fatih Sarıkaya, Hüseyin Sinan Akay**

İzmir State Hospital, İzmir, Türkiye

**Background/Aims:** To compare the clinical efficacy, feasibility, and safety of endoscopic mucosal resection (EMR) and hybrid endoscopic submucosal dissection (ESD) in submucosal gastric lesions.

**Materials and Methods:** Between 2024 and 2025, 28 patients underwent resection (EMR: n = 16; hybrid ESD: n = 12). Resection was indicated for lesions originating from the second or third gastric wall layers on endoscopic ultrasonography (EUS) or when a definitive prediagnosis cannot be established. EMR or hybrid ESD was selected accordingly, with hybrid ESD preferred for larger or fibrotic lesions. Patient demographics, lesion characteristics (size ≤15 mm, location, histopathology), and procedural outcomes (en-bloc resection, R0 resection, complications) were analyzed.

**Results:** The mean age was 61.5 years, and 57% were male. The mean lesion size was 12.3 mm, most frequently located in the corpus. Histopathology revealed gastric neuroendocrine tumor (n = 15), gastrointestinal stromal tumor (n = 5), leiomyoma (n = 3), ectopic pancreas (n = 3), and lipoma (n = 2). En-bloc resection rates were 87.5% for EMR and 91.6% for hybrid ESD, whereas R0 rates were 81.2% and 83.3%, respectively. Most non-R0 resections originated from third-layer lesions. Complications occurred in 2 patients (7.1%) with transient bleeding; no perforations were observed. The mean procedure time was shorter for EMR (25.4 ± 6.8 minutes) than for hybrid ESD (33.7 ± 7.9 minutes; *P* < .05).

**Conclusion:** Both EMR and hybrid ESD are safe and effective for small submucosal gastric lesions. Hybrid ESD appears advantageous for larger or fibrotic lesions. Limited R0 resection in third-layer lesions emphasizes diagnostic and technical challenges in this group. Performing resection when EUS findings are inconclusive provides both diagnostic yield and therapeutic benefit, supporting clinical decision-making.

## PS-186 Thymoma with Crohn’s Disease: First Case in the Literature: Concomitation of Trabzon

**Gökhan Aydın, Ahmet Cumhur Dülger, Serra Ecren Aykan**

Division of Gastroenterology, Giresun University School of Medicine, Giresun, Türkiye

Thymomas are rare malignant tumors, and their main pathologic characteristics include a wide range of cytologic patterns within thymic epithelial cells and an association with a nontumoral lymphocytic component. They account for approximately half of anterior mediastinal tumors. About one-third of these are associated with myasthenia gravis. Computed tomography with intravenous contrast is the standard diagnostic modality. A concomitation of inflammatory bowel disease with thymomas has never been reported in the literature. A 27-year-old woman who was previously diagnosed with thymoma and treated surgically had isolated colonic Crohn’s disease for at least 11 years and was admitted to the clinic due to constipation. She had previously used adalimumab and infliximab but discontinued them 2 years ago. Her CDAI score was 150, and her laboratory test results were as follows: WBC 4130 h/mm^3^ hemoglobin 10.0 g/dL, platelet 286.000 mcg/L, C-reactive protein 40.07 mg/L, creatinine 0.72 mg/dL, glucose 88 mg/dL, urea 26.5 mg/dL, e-GFR 115.1 mL/dk/m^2^, ALT 52 U/L AST 17.4 U/L total bilirubin 0.42 mg/dL, direct bilirubin 0.17 mg/dL, indirect bilirubin 0.25 mg/dL, total protein 74.5 g/L, albumin 44.7 g/L uric acid 4 mg/dL, ALP 81u/L, GGT 6U/L, LDH 126 U/L, lipase 29.3 u/L, free T3 2.89 ng/L, free T4 1.26 ng/dL, TSH 4.65 mIU/L, ferritin 6.89 mcg/L, folic acid 8.76 mcg/L, vitamin B12 228 ng/L. MRI of the lower abdomen and rectosigmoidoscopy revealed anal stricture with increased lymph nodes and mesenteritis. Upadacitinib induction and maintenance treatment has just started, and she is still being followed up in the hospital. There is currently no report regarding an association between myasthenia gravis and Crohn’s disease. Furthermore, there is also no report using upadacitinib in such a case. Thus, this unique report was also symbolized by the patient’s intern doctor’s birthplace Trabzon and named by the authors as “concomitation of Trabzon.”

## PS-187 Hepatitis B Reactivation due to Rituximab Treatment

**Serhat Akpınar, Kadir Gişi, Mesut Kuş, Bülent Kantarçeken**

Department of Internal Medicine, Division of Gastroenterology, Sütçü İmam University Faculty of Medicine, Kahramanmaraş, Türkiye

Hepatitis B virus (HBV) reactivation is a significant complication in patients receiving long-term chemotherapy or immunosuppressive therapy. In this case, the aim was to present a patient who developed acute liver failure due to HBV reactivation after a rituximab-containing chemotherapy protocol. A 79-year-old male patient was diagnosed with chronic lymphocytic leukemia 1 year ago. Following diagnosis, he was initiated on a rituximab-containing chemotherapy protocol at an external center. During this period, the patient’s liver enzymes, INR, and bilirubin levels were normal. HBsAg was negative, anti-HBs was positive, and anti-HBc IgG was not tested. Approximately 1 year after starting treatment, the patient presented to the gastroenterology clinic with complaints of fatigue, yellowing of the skin, and dark urine. The patient’s blood tests revealed alanine aminotransferase (ALT) of 911 U/L, aspartate aminotransferase (AST) of 740 U/L, total bilirubin of 1.96 mg/dL, direct bilirubin of 1.49 mg/dL, and an INR of 1.28. A hepatitis panel was ordered and found to be positive for HBsAg, positive for anti-HBs, positive for anti-HBc IgG, negative for anti-HBc IgM, and negative for HBV PCR of 586514 IU/mL. The patient was suspected of developing HBV reactivation due to rituximab treatment, and tenofovir disoproxil fumarate 245 mg was initiated as antiviral therapy. Despite treatment, progressive increases in bilirubin and INR levels were observed during follow-up, and the patient was admitted to the intensive care unit. The patient, who had no chance of liver transplantation during follow-up, died due to acute liver failure and complications related to HBV reactivation. The use of drugs with a high risk of reactivation, such as rituximab, along with positive HBV serologic markers, can result in hepatitis B reactivation in these patients. Therefore, prophylactic antiviral therapy against hepatitis B should be initiated before starting chemotherapeutic drugs with a high risk of reactivation.

## PS-188 Endoscopic Botulinum Injection Treatment in a Patient Presenting with Type 2 Achalasia and Associated Rare Esophageal Varices

**Mehmet Şerif Aktaş, Akif Orhan, Muhammet Devran Işık, Serkan Öcal, Ayhan Hilmi Çekin, Galip Egemen Atar, Osman Çağın Buldukoğlu**

Department of Gastroenterology, University of Health and Science, Antalya Training and Research Hospital, Antalya, Türkiye

Achalasia is a rare esophageal motility disorder caused by impaired relaxation of the lower esophageal sphincter, leading to dysphagia, regurgitation, and weight loss. The coexistence of achalasia and esophageal varices is rare, creating significant diagnostic and therapeutic challenges. Standard interventions such as pneumatic dilation, POEM, or surgical myotomy carry a high risk of bleeding in such patients. A 76-year-old man with cryptogenic liver cirrhosis (Child-Pugh A, MELD-Na 11) presented with progressive dysphagia for solids and liquids. Manometry confirmed Type 2 achalasia. Endoscopy revealed extensive food stasis and Grade 3 esophageal varices occupying more than two-thirds of the esophageal lumen. Considering the bleeding risk, endoscopic botulinum toxin injection was performed in 4 quadrants 1-2 cm above the *Z*-line. The procedure was uneventful, and the patient experienced complete resolution of dysphagia during follow-up. No complications were observed. Achalasia can exacerbate malnutrition and complications in patients with cirrhosis. In cases with concomitant esophageal varices, conventional therapies pose a high risk of hemorrhage. Botulinum toxin injection, with or without EUS guidance, is a safe and effective alternative. This case demonstrates successful symptomatic relief of Type 2 achalasia in a patient with high-grade esophageal varices without adverse events, emphasizing individualized, risk-adapted therapy in complex clinical scenarios.

## PS-189 Insecticide-Induced Toxic Hepatitis: A Case Report Highlighting Pyrethroids, Carbamate, and Organophosphate Exposure

**Mehmet Şerif Aktaş, Akif Orhan, Beşir Kaya, Serkan Öcal, Ayhan Hilmi Çekin, Galip Egemen Atar**

Department of Gastroenterology, University of Health and Science, Antalya Training and Research Hospital, Antalya, Türkiye

Toxic hepatitis may result from environmental chemical exposure, including household insecticides. Pyrethroids, carbamates, and organophosphates, commonly used for pest control, are increasingly recognized for their hepatotoxic potential, particularly in individuals with prior liver disease. A 20-year-old woman presented with fatigue, nausea, weight loss, dark urine, and jaundice. Laboratory findings showed severe hepatocellular injury (ALT 2256 U/L, AST 1640 U/L), hyperbilirubinemia (total bilirubin 5.4 mg/dL), and coagulopathy (PT 17.1 seconds, INR 1.57). Viral and autoimmune serologies were negative. A detailed history revealed recent household exposure to an insecticide (Baygon) containing pyrethroids (cyfluthrin, transfluthrin, cypermethrin, prallethrin), a carbamate (propoxur), and an organophosphate (chlorpyrifos). Liver biopsy demonstrated centrilobular hepatocyte loss with subacute necrosis, portal inflammation with lymphocytes, plasma cells, and eosinophils, sinusoidal dilatation, and focal bile duct proliferation—findings consistent with acute toxic hepatitis. Intravenous prednisolone (40 mg) was initiated, resulting in rapid biochemical and clinical improvement by day 7. She was transitioned to oral steroids and achieved full recovery by day 18 (ALT 602 U/L, AST 91 U/L, total bilirubin 1.98 mg/dL, PT 12.7 seconds). Insecticide-induced hepatotoxicity, though rare, should be considered in patients with acute liver injury and a relevant exposure history. Pyrethroids, carbamates, and organophosphates can cause hepatocellular injury through oxidative stress, immune-mediated mechanisms, and metabolic pathways. Early recognition, cessation of exposure, supportive care, and, in selected cases, corticosteroid therapy can lead to favorable outcomes. Routine environmental and occupational history-taking is essential for accurate diagnosis and prevention of recurrence.

## PS-191 A Rare Case: Detection of Primary HCC During Follow-Up After Liver Transplantation

**Serkan Rendeci ^1^ , Rauf Mehtiyev ^1^ , Nilay Danış ^1^ , Aytaç Gülcü ^1^ , Tarkan Ünek ^1^ , Sezai Yılmaz ^2^**

^1^Department of Gastroenterology, Dokuz Eylül University Hospital, İzmir, Türkiye

^2^Department of General Surgery, Malatya Inönü University, Malatya, Türkiye

Hepatocellular carcinoma (HCC) is the most common type of primary liver cancer, the sixth most common cancer worldwide, and the third leading cause of cancer-related death. Although approximately 80% of HCC cases occur in patients with cirrhosis, its incidence is increasing in individuals without cirrhosis. In Türkiye, chronic HBV has been identified as the predominant cause of noncirrhotic HCC. Here, a rare case of liver transplantation due to liver cirrhosis secondary to Wilson’s disease 10 years ago is presented, which revealed no HCC on explant pathology, but developed noncirrhotic HCC during posttransplant follow-up from a marginal donor. A 48-year-old male patient underwent a cadaveric liver transplant in 2015 after liver cirrhosis secondary to Wilson’s disease. Explant pathology revealed Wilson’s cirrhosis, but no HCC was present in the explant. The transplant was a marginal cadaveric transplant. Eight years after the transplant, MRCP performed following LFTs revealed anastomotic stenosis and stones located above the stenosis. Two ERCPs were performed, resulting in the removal of the stones and the insertion of a stent. Ten years after the transplant, MRCP performed due to LFTs revealed a hyperintense nodular lesion measuring 14 mm in segment 3,a 9-mm subcapsular lesion in segment 4B, and 2 additional washouts measuring 8 mm medially. Biopsies from these lesions were reported as hepatocellular carcinoma. During treatment, 1 TARE and 1 RF ablation were performed. Sorafenib treatment was initiated. The patient was being evaluated for transplantation, and therefore, sorafenib was discontinued at the final stage. Following TARE, the patient developed bleeding and discharge from the surgical site, and debridement was performed. Because the wound culture grew meropenem-resistant *Klebsiella* and ESBL(+) *E. coli*, dual antibiotic therapy was initiated. The patient developed DIC following sepsis and subsequently died in the intensive care unit. To the authors’ knowledge, this case is the first in the literature in which HCC developed in the liver of a marginal donor who underwent transplantation due to Wilson’s cirrhosis, and whose explant pathology revealed no HCC but was retained during follow-up.


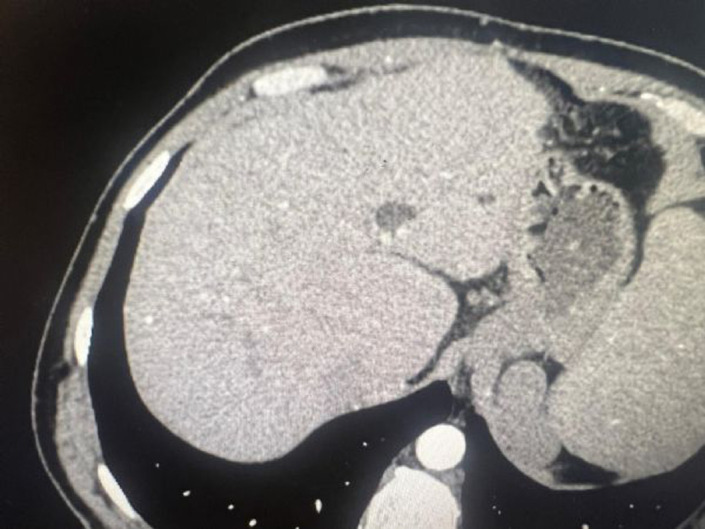


Figure 1. xxx.

## S-192A Case of IgG4-Related Disease Presenting with Ascites and a Typical Jejunal İnvolvement

**Orhan Cem Deniz, Gürhan Şişman, Nurdan Tözün**

Department of Gastroenterology, Acıbadem Mehmet Ali Aydınlar University Faculty of Medicine, Istanbul, Türkiye

The most common causes of ascites include cirrhosis, malignancy, heart failure, and tuberculosis; however, inflammatory processes may also lead to ascites. Immunoglobulin G4 (IgG4)-related disease is a fibroinflammatory condition that can affect multiple organs, most commonly the pancreas, biliary tract, retroperitoneum, kidneys, lungs, and salivary glands. It should be suspected in cases with atypical organ involvement. A rare case of IgG4-related disease with jejunal involvement and ascites is presented. A 52-year-old woman with no prior medical history presented to the gastroenterology outpatient clinic with a 1-month history of abdominal distension. She had unintentionally lost 15 kg over the previous year. There was no history of fever or night sweats, but she reported intermittent abdominal pain and vomiting. Physical examination revealed abdominal distension with shifting dullness. Initial laboratory tests showed ALT 83 IU/L, AST 43 IU/L, LDH 268 IU/L, serum albumin 3 mg/dL, and CRP 3.15 mg/dL; other parameters were within normal limits. Abdominal ultrasonography revealed massive ascites. Diagnostic paracentesis demonstrated exudative fluid. ANA, complement C3-C4, anti-dsDNA, ENA profile, thyroid function tests, celiac markers, and ACE levels were normal. Viral and autoimmune hepatitis panels were negative. ARB, ADA, culture, and Quantiferon tests excluded tuberculosis. Serum IgG4 level was 2.41 g/L (normal, 0.03-2.01). Cytology of the ascitic fluid, gastroscopy, and colonoscopy showed no additional findings. PET-CT showed no FDG uptake. CT revealed jejunal wall thickening with segmental narrowing. An attempt at enteroscopy failed to reach the jejunum. Laparoscopic peritoneal and ovarian biopsies were performed. Histopathology demonstrated numerous IgG4-positive plasma cells (78/high power field) with lymphoid aggregates and fibrosis. Based on the 2019 EULAR classification criteria, IgG4-related disease was diagnosed, and rituximab therapy was initiated. Ascites secondary to IgG4-related disease is rare. Jejunal involvement is particularly atypical and may delay diagnosis. IgG4-related disease should be considered in the differential diagnosis of unexplained ascites.


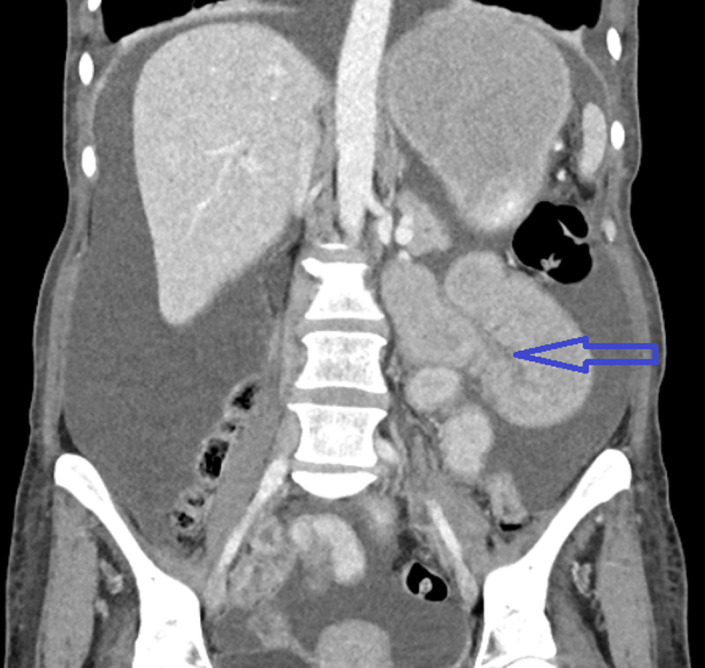


Figure 1. Involvement of the jejunum by the disease. Jejunal wall thickening and segmental narrowing.

## PS-195 Extrahepatic Autoimmune Diseases Associated with Autoimmune Hepatitis

**Abdullah Mübin Özercan ^1^ , Ali Çağrı Oral ^1^ , Sedat Çiçek ^1^ , Furkan Kırsoy ^1^ , Selman Çelik ^1^ , Abdulvahap Hohluoğlu ^1^ , Ahmet Yol ^2^ , Berivan Karataş ^2^ , Mehmet Yalnız ^1^ , İbrahim Halil Bahçecioğlu ^1^**

^1^Department of Gastroenterology, Fırat University School of Medicine, Elazığ, Türkiye

^2^Department of Internal Medicine, Fırat University School of Medicine, Elazıği Türkiye

**Background/Aims:** Autoimmune hepatitis (AIH) is not limited to hepatic involvement but is often associated with various extrahepatic autoimmune diseases. The most common extrahepatic autoimmune conditions accompanying AIH include autoimmune thyroid diseases (Hashimoto’s thyroiditis and Graves’ disease), type 1 diabetes mellitus, celiac disease, rheumatoid arthritis, Sjögren’s syndrome, and systemic lupus erythematosus. This study evaluated the prevalence and classification of extrahepatic autoimmune diseases in patients diagnosed with autoimmune hepatitis.

**Materials and Methods:** Between January 2014 and July 2025, the medical records of patients diagnosed with autoimmune hepatitis based on liver biopsy findings and followed in the clinic were retrospectively reviewed. Patients with overlap syndromes involving primary sclerosing cholangitis or primary biliary cholangitis were excluded. The remaining patients were assessed for extrahepatic autoimmune diseases identified at diagnosis and during follow-up, and these comorbidities were classified accordingly.

**Results:** Among 228 patients diagnosed with autoimmune hepatitis through liver biopsy, 18 patients with overlap syndromes were excluded, leaving 210 eligible patients. The mean age was 45.3 ± 17.2 years (range, 16-73), and 163 (77.6%) were female. The mean follow-up duration was 65.5 months (range, 6-202). Extrahepatic autoimmune diseases were identified in 26 patients (12.3%). The most frequent comorbidities were rheumatologic diseases (n = 12, 42.8%), followed by celiac disease in 5 patients (17.8%), inflammatory bowel diseases in 5 patients (17.8%; 3 with ulcerative colitis, 2 with Crohn’s disease), multiple sclerosis in 3 patients (10.7%), and Hashimoto’s thyroiditis in 3 patients (10.7%). Among the rheumatologic conditions, Sjögren’s syndrome was the most common (n = 5, 17.8%).

**Conclusion: **The presence of extrahepatic autoimmune diseases can influence both prognosis and therapeutic response; therefore, it is recommended that individuals diagnosed with AIH be actively screened for concomitant systemic autoimmune disorders. Particular attention should be given to evaluating rheumatologic diseases in this patient population.

## PS-198 Malignancy-Mimicking Gastric Tuberculosis: A Case with Diagnostic Challenges

**Derya Kirman, Tarkan Karakan, Murat Kekilli, Çağdaş Kalkan, Ali Karataş, Güner Kılıç, Gülden Bilican, Kenan Moral, Halit Kandemir, Beril Demir, Enes Cömert, Fatih Acehan, Yusufcan Yılmaz, Yunus Emre Börü, Mehmet Cindoruk**

Department of Gastroenterology, Gazi University Faculty of Medicine, Ankara, Türkiye

Gastric tuberculosis (TB) is a rare form of extrapulmonary TB caused by the Mycobacterium tuberculosis complex. It may present endoscopically as ulcerative, infiltrative, or mass-like lesions, often mimicking malignancy, lymphoma, or gastrointestinal stromal tumor (GIST). Diagnosis is challenging due to nonspecific symptoms and the limited sensitivity of microbiological tests. Histopathological identification of granulomatous inflammation, supported by immunological assays, is essential. A rare case of gastric TB initially suspected to be GIST, highlighting diagnostic difficulties and the role of systemic granulomatous findings is reported. A 60-year-old woman with diabetes, hypertension, chronic kidney disease, Mobitz type 2 block, and decompensated heart failure initially presented to an external center with 6 months of abdominal pain and dyspnea. Abdominal CT revealed a 63×52 mm mass along the gastric greater curvature with cystic-necrotic areas, suggestive of GIST, along with hepatic and splenic lesions. Upper endoscopy showed a 3×3 cm depressed lesion in the corpus, and EUS revealed a 65×40 mm heterogeneous mass in the muscularis propria. Fine-needle biopsy showed only normal smooth muscle. During follow-up, she developed melena and worsening abdominal pain and was admitted to this hospital. Repeat endoscopy showed a 55- to 60-mm ulcerated mass with active bleeding; angiographic embolization of the left gastric artery controlled the hemorrhage. Liver biopsy demonstrated nonnecrotizing granulomatous hepatitis, and axillary lymph node excision revealed nonnecrotizing granulomatous lymphadenitis. The interferon-gamma release assay was positive. Considering systemic granulomatous findings and nondiagnostic gastric samples, the lesion was interpreted as gastric TB. Anti-TB therapy led to marked regression on follow-up endoscopy. Gastric TB is rare and often misdiagnosed, mimicking malignancy or GIST. Deep tissue sampling and clinical suspicion are crucial. Systemic granulomatous inflammation and a positive IGRA support the diagnosis in unclear cases. Early anti-TB therapy can resolve lesions and avoid unnecessary surgery.

## PS-199 Intrahepatic Cholestasis Case Series with Genetic Mutation

**Doğancan Akyürek ^1^ , Hüseyin Alkım ^1^ , Canan Alkım ^1^ , İlker Şen ^2^ , Emrullah Düzgün Erdem ^2^ , Mehtap Üçer ^2^ , Erman Mercan ^1^ , Semra Dağdelen ^1^ , Şefikcan Biricik ^1^ , Murat Yıldırım ^1^ , Merve Ceren Ceylan Eroğlu ^1^**

^1^Department of Gastroenterology, University of Health Sciences, Şişli Hamidiye Etfal Training and Research Hospital, Istanbul, Türkiye

^2^Şişli Hamidiye Etfal Training and Research Hospital, Gastroenterology Clinic, İstanbul, Türkiye

Benign recurrent intrahepatic cholestasis (BRIK) is a disease characterized by jaundice, severe pruritus, nausea, increased alkaline phosphatase (ALP), direct-dominant hyperbilirubinemia, and near-normal gamma-glutamyl transferase (GGT).

In patients presenting for various reasons, genetic cholestasis was diagnosed in cases with and without identified BRIK gene mutations. Four of the patients were male and 2 were female. All patients were diagnosed in adulthood. The main symptoms at presentation to the hospital in 5 patients were pruritus, jaundice, and nausea. One patient diagnosed upon screening also reported experiencing intermittent itching attacks. Autoimmune hepatitis, viral hepatitis, metabolic diseases, anatomic disorders, and extrahepatic biliary tract diseases were excluded in all patients. Four of the patients had identified BRIK gene mutations. Two male patients, 1 with common bile duct stones and the other with pancreatic adenocarcinoma, had received definitive treatment for their existing conditions. However, because bilirubin levels did not return to normal for months, a BRIK study was conducted, and different gene mutations were found in these patients. An AKR1D1 gene mutation was detected in the patient with prolonged cholestasis triggered by gallstones, and an SLC2A1 gene mutation was detected in the other patient triggered by pancreatic malignancy. The SLC2A1 mutation is associated with transport proteins, whereas AKR1D1 is associated with bile synthesis. This case series was shared to demonstrate that patients with mutations other than the known BRIK genes may also present with intrahepatic cholestasis. Medical treatments such as ursodeoxycholic acid, cholestyramine, rifampin, and steroids were tried in the patients’ treatment. Two patients underwent repeated bilirubin apheresis. Five patients underwent ERCP, and 1 even had a nasobiliary drain inserted. One patient underwent liver transplantation due to intractable pruritus. Based on treatment experience, it is not possible to definitively assert the effectiveness of any method other than liver transplantation.

## PS-200 Pulmonary Metastasis Occurring 11 Years After Liver Transplantation for Hepatocellular Carcinoma on the Background of Wilson’s Disease: A Rare Case of Late Recurrence

**Şerife Özkarakoç Ertürk ^1^ , Amed Trak ^2^ , Zeynep Melek Ellikoğlu ^2^ , Dilara Turan Gökçe ^2^ , Özge Koç ^2^ , Volkan Yılmaz ^2^ , Hale Gökcan ^2^ , Ramazan İdilman ^2^**

^1^Department of Internal Medicine, Ankara University Faculty of Medicine, Ankara, Türkiye

^2^Department of Gastroenterology, Ankara University Faculty of Medicine, Ankara, Türkiye

We present a rare case of a patient who underwent liver transplantation in 2013 due to HCC secondary to Wilson’s disease, with explant histopathology revealing moderately differentiated HCC. The patient remained disease-free for 11 years before developing isolated pulmonary metastasis in 2024. A 63-year-old woman was diagnosed with Wilson’s disease in 2006 following investigations for abdominal pain, which revealed low ceruloplasmin, increased IgG/IgA, granular hepatic parenchyma, right lobe atrophy on ultrasound, and a family history (father, sister, aunt) consistent with Wilson’s disease. In 2010, serum alpha-fetoprotein (AFP) levels increased to 18 ng/mL. Dynamic liver CT identified a 22 × 21-mm lesion in segment 4b, showing arterial phase hyperenhancement and late-phase washout, consistent with HCC. Radiofrequency ablation was performed on December 9, 2011. Subsequently, on May 14, 2013, the patient underwent cadaveric liver transplantation. Posttransplant AFP levels remained stable between 4 and 6 ng/mL over 11 years, with no evidence of recurrence on clinical, biochemical, or radiological surveillance. In April 2024, the patient presented with a cough. Thoracic CT revealed a 47 × 40-mm mass in the apical segment of the right upper lobe, with PET-CT demonstrating a SUVmax of 4.9. On May 9, 2024, an EBUS-TBNA biopsy revealed a cytological diagnosis reported as positive for malignancy, suggestive of metastatic hepatoid epithelial tumor consistent with HCC. Treatment with sorafenib (2 × 400 mg) was initiated, immunosuppression was modified from tacrolimus to everolimus, and entecavir was added. In conclusion, this case highlights that “remission” posttransplantation does not guarantee an absolute cure, and lifelong surveillance is warranted even in patients classified as low risk. This report presents a rare case of isolated pulmonary metastasis occurring 11 years after liver transplantation for HCC secondary to Wilson’s disease.

## PS-201 The Importance of a Second Colonoscopy: A Case of Right Colon-Located McKittrick-Wheelock Syndrome

**Nur İlayda Genç ^1^ , Serhat Özer ^2^ , Fatih Aslan ^2^ , Devrim Müge Özarı Gülnar ^2^ , Alper Yurci ^2^**

^1^Department of Internal Medicine, Koç University Hospital, İstanbul, Türkiye

^2^Department of Gastroenterology and Hepatology, Koç University Hospital, İstanbul, Türkiye

Chronic, recurrent diarrhea in elderly patients includes a wide spectrum of differential diagnoses: infectious, inflammatory, ischemic, neoplastic and drug-induced. In this case, the diagnostic and therapeutic process of a patient whose distal colon cannot be fully visualized in the initial colonoscopy but was diagnosed following a second examination is presented. A 79-year-old woman with a history of hypertension and chronic kidney disease presented with 2 months of diarrhea. She had been treated with ciprofloxacin, rifaximin, loperamide, and probiotics, and had required hospitalization twice. Despite laboratory and radiologic investigations, no specific etiology was identified. Colonoscopy failed to fully visualize the right colon, and the accessible segments showed normal mucosa without biopsy sampling. Her regular medications included olmesartan and mirtazapine. She reported weight loss of 10 kg, from 69 kg to 59 kg. On physical examination, the patient appeared dehydrated, with hyperactive bowel sounds. Laboratory results showed sodium: 137 mmol/L, potassium:3.1 mmol/L, C-reactive protein: 8 mg/L, creatinine: 1.66 mg/dL, hemoglobin: 11.5 g/dL, and MCV: 96 fL. Stool analysis revealed no leukocytes, erythrocytes, protozoa, or fat globules, and culture showed no bacterial growth. Fecal calprotectin: 105.9 µg/g, and elastase>500 µg/g. A repeat colonoscopy performed for full colon evaluation revealed a laterally spreading tumor (LST, IIa+Is type) with a villous and nodular mixed pattern, encircling approximately 300°of the lumen and spanning a 3-4 cm segment in the hepatic flexure. Additionally, a 5-mm satellite polyp was observed. The clinical and endoscopic findings were consistent with McKittrick-Wheelock syndrome, and endoscopic submucosal dissection (ESD) was recommended. During follow-up, worsening diarrhea led to the development of acute kidney injury superimposed on chronic kidney disease. Laboratory findings revealed creatinine:2.92 mg/dL, urea:65 mg/dL, and severe metabolic acidosis (pH 7.19, pCO_2_: 35.9 mm Hg, HCO_3_:12.3 mmol/L). The patient was hospitalized, and olmesartan was discontinued. Intravenous hydration at 2-3 mL/kg/hour and bicarbonate replacement were administered according to deficit. After improvement in urine output and laboratory values, ESD was successfully performed. Histopathological examination revealed tubulovillous adenoma with a polypoid configuration and lateral spread containing extensive high-grade dysplasia. Following the procedure, the frequency of diarrhea decreased, and the patient has remained clinically well. In this case, olmesartan-induced diarrhea was also considered, and further evaluation was performed. Ultimately, the patient was diagnosed with the rare McKittrick-Wheelock syndrome. Although this syndrome is typically reported in the rectosigmoid region (1), this case is notable for the occurrence of a villous adenoma in the right colon, accompanied by renal impairment (2) and severe metabolic acidosis. Furthermore, the inability to fully evaluate the distal colon during the first colonoscopy—and the identification of the lesion only after a second, complete colonoscopy—highlights once again the critical importance of total colon examination in the diagnostic approach to chronic diarrhea.


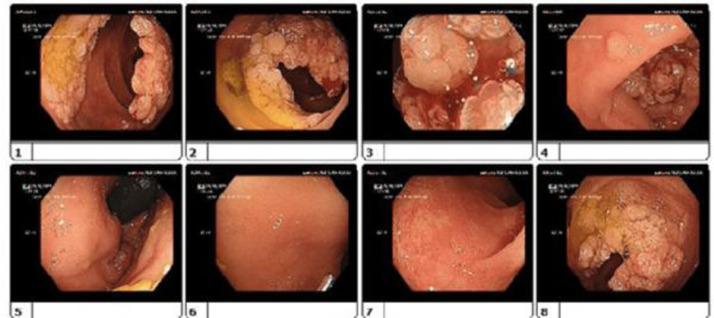


Figure 1. Colonoscopic appearance.

A nodular mixed-type LST (IIa+Is) lesion with a villous pattern, occupying a 3- to 4-cm segment at the hepatic flexure and encircling approximately 300° of the lumen, along with a 5-mm satellite polyp.


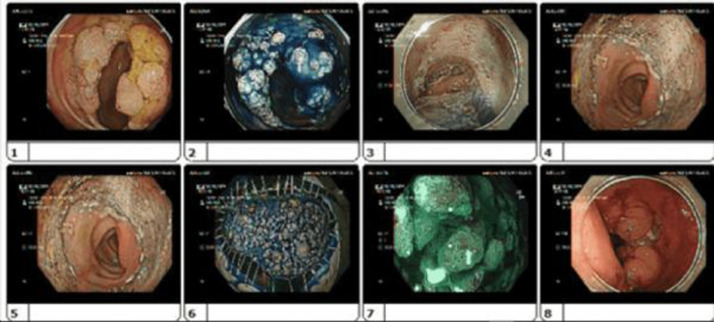


Figure 2. Endoscopic submucosal dissection.

Table 1.Laboratory Results

HCO_3_^-^, bicarbonate; mg/dL, milligrams per deciliter; mmol/L, millimoles per liter.

**Table:** 

Time point	Creatinine (mg/dL)	pH	HCO3- (mmol/L)
Baseline	1.6		
Follow-up 1	2.92		12.3
Follow-up 2	2.47	7.292	12.9
Follow-up 3	2.02	7.247	
Follow-up 4	1.74	7.344	
Follow-up 5	1.51	7.392	
Follow-up 6	1.31		

## PS-203 What Is the Risk of Gastric Cancer According to OLGIM Staging in the Cases with Intestinal Metaplasia?

**Levent Erdem ^1^ , Özlem Özer ^1^ , Alptekin Şen ^2^**

^1^Department of Gastroenterology, Demiroğlu Bilim University, Florence Nightingale Hospital, İstanbul, Türkiye

^2^Department of Pathology, Demiroğlu Bilim University, Florence Nightingale Hospital, İstanbul, Türkiye

**Background/Aims:** Gastric cancer (GC) is one of the most common cancers with a high mortality rate. The most effective method for early diagnosis of GC is upper gastrointestinal endoscopy (UGE) and histopathological examination to detect precancerous lesions (PCL). In recent years, OLGIM histopathological diagnosis and staging have gained importance in the histopathological diagnosis of intestinal metaplasia (IM). The present study aimed to evaluate the risk of GC in patients diagnosed with IM according to the OLGIM classification.

**Materials and Methods:** Consecutive cases with at least 5 biopsies (biopsy sites: 2 biopsies from the antrum, 1 biopsy from the incisura angularis, and 2 biopsies from the corpus) during upper gastrointestinal endoscopy were included in the study. Exclusion criteria included endoscopy for upper GI bleeding and advanced liver or kidney disease.

Age, gender, endoscopy indication, and diagnosis were recorded for all patients.

**Results:** Cases diagnosed with iM as a result of histopathological examination were staged as OLGIM (0-I-II-III) by the pathology department. All patients were evaluated for *H. pylori*. The study identified 144 cases of IM, with 84 females (58%) and 60 males (42%), aged 24-82 years. *H. pylori* was positive in 92 (64%) and negative in 52 (36%).

A total of 100 cases with IM (69%) were staged as OLGIM I, 36 (25%) as OLGIM II, and 8 (5%) as OLGIM III. All cases with OLGIM III were in the 60-80 age group.

**Conclusion:** In the present study, the majority of iM cases were staged as OLGIM I.

One in 4 cases was identified as stage II.

It is noteworthy that even young patients (in their30s and 40s) had stage II cases.

The rate of advanced-stage (OLGIM III) iM, which carries a high risk of gastric cancer, was found to be low. The diagnosis rate and stage of intestinal metaplasia increased with age.

## PS-204 Can the Treatment Paradox in Fundic Gland Polyps Caused by Proton Pump Inhibitors Be Corrected with Polypectomy?

**Azra Hatice Begüm Salimoğlu Sarıoğlu ^1^ , Enes Çelikmakas ^1^ , Ufuk Yazar ^1^ , Hasan Kurt ^1^ , Bilal Ergül ^2^ , Özlem Gül ^2^**

^1^Department of Internal Medicine, Lokman Hekim University Faculty of Medicine, Ankara, Türkiye

^2^Department of Gastroenterology, Lokman Hekim University Faculty of Medicine, Ankara, Türkiye

**Background/Aims:** Proton pump inhibitors (PPIs) play a role in the development of fundic gland polyps during chronic use. Although these polyps are mostly benign, new polyp formation can be observed in cases where PPI use is not discontinued. This situation is called the “treatment paradox” because even if existing polyps are removed by polypectomy, the pathophysiological process associated with PPIs continues. However, data on the effect of polyps on reflux symptoms and the need for PPI use are limited. This study investigated changes in symptom scores and the need for PPI use in patients who underwent polypectomy due to fundic gland polyps.

**Materials and Methods:** Nine patients diagnosed with fundic gland polyps and undergoing polypectomy were included in the study. All patients completed the Gastroesophageal Reflux Disease-Health Related Quality of Life (GERD-HRQL) questionnaire before the procedure and 3 months after polypectomy. Changes in questionnaire scores and PPI usage were compared.

**Results:** In all cases, the polypectomy procedure was completed without complications. The mean GERD-HRQL score before the procedure was 44.1, whereas the mean score after the procedure decreased to 16.7. An average reduction of 61.97% in symptom scores was observed. At the individual level, a significant decrease in questionnaire scores was noted in all patients. Furthermore, all 9 patients who used daily PPIs before the procedure reported a significant decrease in PPI requirement after the procedure, stating that symptom control was achieved with use only when needed. No patient required repeat intervention due to recurrence of new polyps.

**Conclusion:** This study found a significant decrease in both subjective quality of life scores and PPI usage requirements after polypectomy. These findings suggest that the presence of polyps may contribute to reflux symptoms. Therefore, polypectomy may not only offer a protective solution but also contribute to symptom control. It may also help eliminate the pathophysiological mechanism that can lead to the formation of new polyps and break the habit of daily PPI use.

## PS-205 Upadacitinib in Real-World IBD Practice: 1-Year Single-Center Effectiveness and Safety Data

**Gizem Dağcı, Ersel Bilgin, Sabuhi Mammadov, Besim Fazıl Ağargün, Pelin Telli, Asım Gurbanov, Mehmet Akif Yağlı, Sezen Genç Uluçeçen, Bilger Çavuş, Aslı Çiftçibaşı Örmeci, Kadir Demir, Selman Fatih Beşışık, Sabahattin Kaymakoğlu, Filiz Akyüz**

Division of Gastroenterology, Department of Internal Medicine, İstanbul University Faculty of Medicine, İstanbul, Türkiye

**Background/Aims: **Upadacitinib is a selective Janus kinase inhibitor approved in the country for the treatment of moderate-to-severe ulcerative colitis (UC) and Crohn’s disease (CD). This study aimed to evaluate the 1-year clinical, biochemical, and safety outcomes of upadacitinib therapy in patients with inflammatory bowel disease (IBD) in real-world practice.

**Materials and Methods: **Thirty consecutive patients with IBD who initiated upadacitinib treatment between December 2023 and October 2025 were retrospectively analyzed.

**Results:** Of the 30 patients, 17 (56.7%) had CD and 13 (43.3%) had UC; the median age was 34 years (range, 19-61), and 53% were female. Extraintestinal manifestations were observed in 47% of patients with Crohn’s disease (n = 8), most commonly axial spondyloarthropathy.

All patients had prior exposure to at least 1 anti-TNF agent. Twenty-two patients (74%) had received 3 or more biologic agents, and 1 patient had prior exposure to tofacitinib. The median treatment duration was 15.6 months (IQR: 12.7-16.4); 25/30 (83.3%) patients were on treatment at 1 year, whereas 5/30 (16.7%) discontinued therapy due to loss of response (n = 2), intolerance (n = 1), or complications (n = 2; closed perforation and mandibular osteonecrosis with psoas abscess).

Clinical scores improved significantly: CDAI decreased from 195 to 50 (Δ −139; *P* = .0049; n = 11) and PDAI from 8.5 to 5.0 (Δ −4; *P* = .0312; n = 6). Biochemically, CRP levels showed a significant reduction (*P* = .04), and fecal calprotectin tended to decrease (150→100 µg/g). Lipid parameters remained stable (triglycerides 128→130 mg/dL, *P* = .75; HDL, LDL, and total cholesterol *P* > .05). During treatment, herpes zoster (n = 1) and acneiform rash (n = 1) occurred. Serious adverse events included 1 closed perforation and 1 case of mandibular osteonecrosis with psoas abscess. No deaths were reported.

**Conclusion:** In real-world practice, upadacitinib demonstrated rapid and sustained clinical and biochemical improvement, with a high 1-year treatment persistence rate (83%) and an acceptable safety profile in patients with IBD refractory to anti-TNF and multiple biologic therapies.

## PS-206 The Diagnostic and Therapeutic Role of Cholangioscopy in Complex Biliary Strictures and Challenging Choledocholithiasis Cases: A Case Series

**Ramis Çatalbaş, Bayram Yeşil, Dilek Oğuz**

Division of Gastroenterology, Department of Internal Medicine, Kırıkkale University Faculty of Medicine, Kırıkkale, Türkiye

**Background/Aims:** Endoscopic retrograde cholangiopancreatography (ERCP) is a standard medical procedure for diagnosing and treating diseases of the pancreaticobiliary system. However, complicated biliary strictures and challenging cases of choledocholithiasis can pose significant difficulties in diagnosis and treatment. Cholangioscopy allows for direct visualization of biliary tracts, biopsy sampling from the lesion area, and treatment of difficult lithiasis. It aids in differentiating benign from malignant etiologies and in planning treatment. In this case series, 5 cases where cholangioscopy was utilized for diagnosis and treatment are reported on.

**Materials and Methods:** Patients were followed up at the clinic between February 2024 and October 2025. Cholangioscopy was performed to evaluate 5 patients aged 46-71 years, 4 of whom had biliary strictures, and 1 had a history of difficult choledocholithiasis requiring repeated ERCP. Laboratory, radiological, and pathological findings were retrospectively reviewed.

**Conclusion:** Cholangioscopy enables direct evaluation of suspected complicated strictures, targeted biopsy, and treatment during the procedure. In this case series, a definitive diagnosis was achieved in 80% of cases. Its use should be expanded, and larger series are needed.

## PS-207 Late-Onset Immunodeficiency in a Patient Diagnosed with Myelin Oligodendrocyte Glycoprotein Antibody-Associated Disease

**Dr. Muratcan Kasımoğlu ^1^ , Dr. Ayşe Akpınar ^1^ , Uzm. Dr. Ümitcan Ateş ^2^ , Prof. Dr. Ömür Ardeniz ^2^ , Uzm. Dr. Sercan Kamalı ^3^ , Prof. Dr. Nevin Oruç ^3^ , Prof. Dr. Fulya Günşar ^3^**

^1^Department of Internal Medicine, Ege University Faculty of Medicine, İzmir, Türkiye

^2^Division of Allergy and Immunology, Ege University Faculty of Medicine, İzmir, Türkiye

^3^Division of Gastroenterology, Ege University Faculty of Medicine, İzmir, Türkiye

In gastroenterology, chronic diarrhea and weight loss are typically attributed to celiac disease or inflammatory bowel disease. However, these symptoms may sometimes represent the initial signs of immunodeficiency syndromes. Secondary immunodeficiency can result from hematologic malignancies or immunosuppressive therapy. Late-onset hypogammaglobulinemia following anti-CD20 (rituximab) treatment necessitates long-term monitoring and may present with diarrhea and weight loss.

A 35-year-old previously healthy woman was evaluated in 2018 for visual disturbances. She tested positive for myelin oligodendrocyte glycoprotein (MOG) and negative for aquaporin-4 (AQP4) antibodies, confirming MOGAD. Between 2018 and 2020, she received 5 doses of rituximab and corticosteroids. Immunologic data before treatment were unavailable. In 2024, she presented with 9-10 watery stools per day, a 32-kg weight loss, and recurrent infections. Colonoscopy and celiac serology were normal. PET-CT revealed no malignancy but increased bone marrow activity. Treponema pallidum (TP) IgG + IgM was transiently positive. Immunologic evaluation showed markedly reduced immunoglobulins (IgG <3 g/L, IgA 0.5 g/L, IgM 0.25 g/L), consistent with panhypogammaglobulinemia. The kappa/Lambda ratio (1300/685) indicated polyclonal reduction. Total lymphocytes were normal (4.09×10^3^/µL), but subpopulations were abnormal. Treg at 0.8% suggested impaired regulation. CD19 = 0 and CD19/CD20 <1% confirmed B-cell depletion and agammaglobulinemia. The CD4/CD8 ratio (0.3-0.5) showed helper T-cell suppression. NK cells were low-normal, indicating global immune dysfunction. Anti-A and Anti-B negativity (1/8 titer) supported a weak natural antibody response. TP IgG + IgM positivity indicated a past infection or false reactivity.

Findings were compatible with late-onset secondary antibody deficiency syndrome (SAD), although primary immunodeficiency (e.g., CVID) cannot be ruled out due to missing pre-rituximab data. Rituximab may cause persistent immunodeficiency years after treatment, leading to recurrent infections, diarrhea, and weight loss. In patients with MOGAD, TP antibody positivity should be interpreted cautiously for possible cross-reactivity. Immunodeficiency must always be considered in patients with rituximab exposure and unexplained gastrointestinal symptoms.

## PS-208 Extraesophageal Causes of Dysphagia: A Case Series with Clinical, Radiological, and Manometric Findings

**Bengi Öztürk ^1^ , Ersin Gümüş ^2^ , Taylan Kav ^1^**

^1^Gastroenterology Department, Hacettepe University Faculty of Medical, Ankara, Türkiye

^2^Pediatric Gastroenterology Department, Hacettepe University Faculty of Medical, Ankara, Türkiye

In the etiology of dysphagia, besides esophageal causes, extraesophageal pathologies play a significant role. These conditions may mimic esophageal motility disorders, causing diagnostic confusion. This report presents the clinical, endoscopic, radiological, and manometric findings of 8 patients in whom extraesophageal factors were the primary cause of dysphagia.

All 8 patients had intermittent dysphagia to solids. Except for 1, none exhibited alarm symptoms or significant weight loss. Case 1 involved a 51-year-old woman who had normal upper GI endoscopy, barium esophagography, and HRM. Neck US showed cervical cystic lymphangioma. Symptoms resolved after cyst aspiration. Case 2 involved a 43-year-old man who had normal endoscopy and barium study. HRM showed increased upper esophageal sphincter pressure, suggestive of a cricopharyngeal bar. Dysphagia improved after swallowing physiotherapy. Case 3 involved a 66-year-old woman with primary biliary cholangitis and ankylosing spondylitis who had normal endoscopy, barium, CT, and HRM. A swallowing study demonstrated cervical compression from AS-related kyphoscoliosis. Symptoms improved after physiotherapy. Case 4 involved a 78-year-old diabetic woman with severe reflux and dysphagia who had a hiatal hernia, increased DeMeester score, and normal HRM, consistent with GERD-related dysphagia. After LAD stenting, symptoms resolved; subsequent Toupet fundoplication was performed. Case 5 involved a 42-year-old woman who had normal endoscopy; the barium study revealed compression from the aortic knob. Dysphagia resolved after cardiac ablation. Case 6 involved a 59-year-old man who experienced a 40-kg weight loss and was excluded for malignancy. He had normal endoscopy and HRM. The barium study demonstrated cervical osteophytic compression. Case 7 involved a 71-year-old woman who presented with similar cervical osteophyte-related dysphagia on the barium study, with normal endoscopy and HRM. Case 8 involved a 40-year-old woman who had normal endoscopy, barium, and HRM; functional dysphagia was diagnosed, and symptoms resolved after tricyclic antidepressant therapy. Focusing solely on esophageal motility disorders may lead to diagnostic pitfalls. Cervical osteophytes, vascular structures, thoracic deformities, neck masses, or functional dysfunctions can contribute to dysphagia. A multidisciplinary approach—combining swallowing physiotherapy and systemic disease management—yields favorable outcomes. Integrated interpretation of clinical, radiological, and manometric data is crucial for the accurate identification of extraesophageal dysphagia causes.

## PS-209 Crohn’s Disease or Plastron Appendicitis? A Challenging Case in Differential Diagnosis

**Ümit Yeşilova, Osman Özdoğan, Feramuz Demir Apaydın, Mammadhasan Mammadov, Ahmet Emre Ergan, Oktay Bayraktar, Mehmet Kasım Aydın**

Department of Gastroenterology, Mersin University Faculty of Medicine, Mersin, Türkiye

Plastron appendicitis is a complication of acute appendicitis characterized by abscess formation or an inflammatory mass. It may present with clinical and radiological findings similar to Crohn’s disease, particularly in cases involving the terminal ileum. Accurate diagnosis is therefore critical for determining the appropriate therapeutic approach. A 30-year-old female patient presented to an external center with sudden-onset abdominal pain and diarrhea. Laboratory tests revealed CRP: 166 mg/L and WBC: 16.61×10^3^/µL. Abdominal CT demonstrated terminal ileal wall thickening and lymphadenopathy, and the patient was initially treated with antibiotics based on a preliminary diagnosis of inflammatory bowel disease. Due to a poor response to treatment, she was referred to the clinic with a presumptive diagnosis of Crohn’s disease. Given the abrupt onset of symptoms and the absence of previous similar complaints, non-Crohn pathologies were also investigated. Oral and IV contrast-enhanced abdominal CT showed increased density and millimetric abscess foci localized around the cecal base and appendix. Wall thickening was noted in the terminal ileum and sigmoid colon. As the appendix cannot be visualized and the clinical presentation was atypical for Crohn’s disease, the findings were interpreted as indicative of plastron appendicitis. The patient was monitored in the general surgery ward and later discharged with appropriate antibiotic therapy. At the 3-week follow-up, abdominal ultrasonography revealed complete resolution of the right lower quadrant findings. Colonoscopy demonstrated a normal terminal ileum. Soft granulation tissue, thought to be secondary to healing, was observed at the appendiceal orifice, and a biopsy was obtained.

This case highlights that plastron appendicitis should not be overlooked in the differential diagnosis of patients presenting with suspected Crohn’s disease. Careful evaluation of clinical and radiological findings enabled the avoidance of unnecessary steroid therapy and successful management with conservative antibiotic treatment.


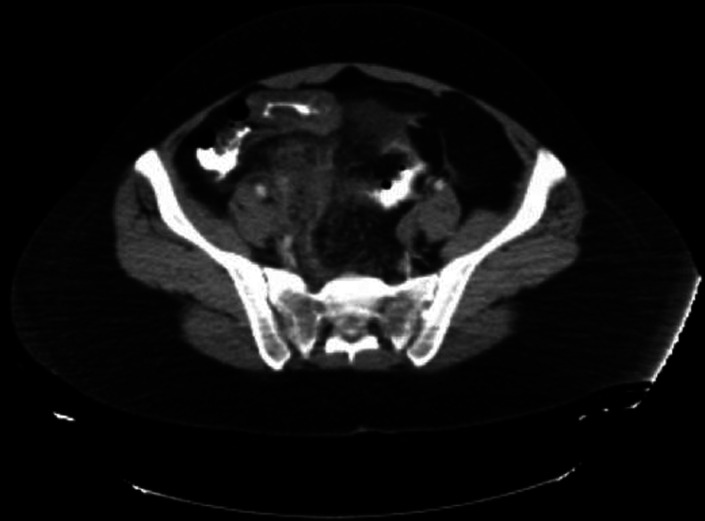


Figure 1. CT image showing terminal ileal wall thickening.


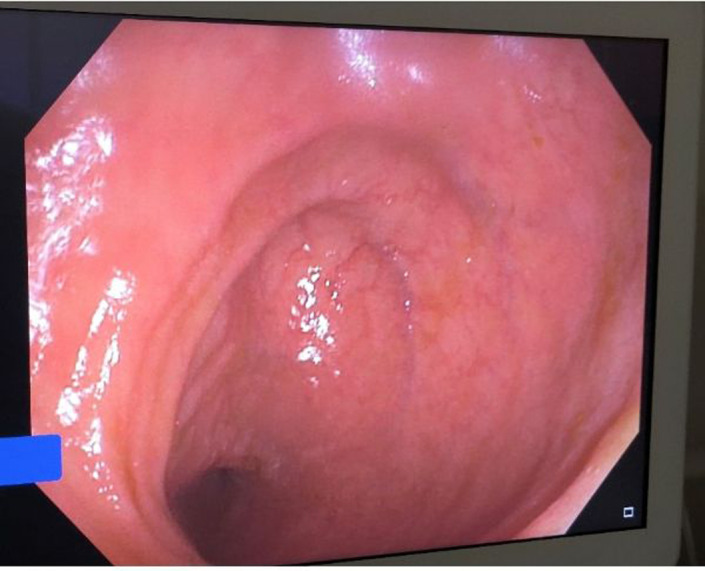


Figure 2. Colonoscopic view of the normal terminal ileum after antibiotic therapy.


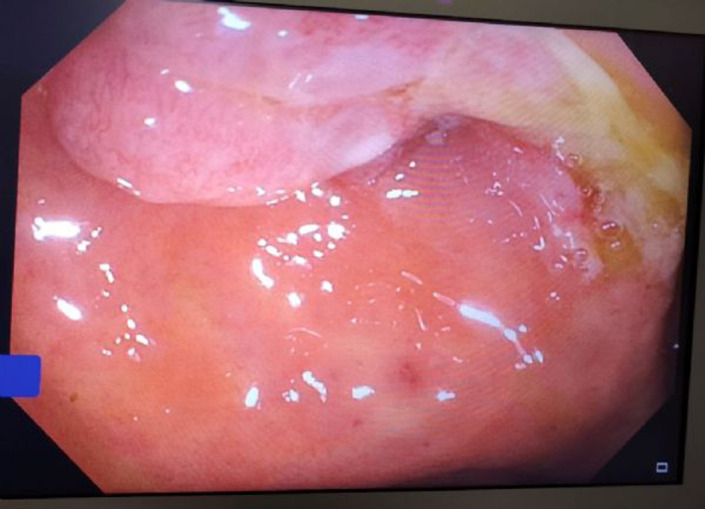


Figure 3. Granulation tissue secondary to inflammation in the appendix after antibiotic therapy.

## PS-210 A Rare Coexistence: A Case of Concomitant Celiac Disease and Esophageal Squamous Cell Carcinoma

**Gülmira Akçay ^1^ , Artuner Varlıbaş ^1^ , Bayram Yeşil ^2^ , Zekiye Nur Öztürk ^1^ , Ramis Çatalbaş ^2^ , Dilek Oğuz ^2^**

^1^Department of Internal Medicine, School of Medicine, Kırıkkale University School of Medicine, Kırıkkale, Türkiye

^2^Department of Internal Medicine, Division of Gastroenterology, Kırıkkale University School of Medicine, Kırıkkale, Türkiye

Celiac disease is an autoimmune enteropathy triggered by gluten ingestion in genetically predisposed individuals. Although it primarily presents with gastrointestinal symptoms, it can also be associated with various extraintestinal and neoplastic conditions. Although the association between celiac disease and small intestinal lymphoma is well established, the coexistence of celiac disease with esophageal squamous cell carcinoma (SCC) is extremely rare. Chronic inflammation, immune dysregulation, and nutritional deficiencies may play a role in this relationship. A 36-year-old woman was admitted with progressive dysphagia, retrosternal burning, and regurgitation. Her initial endoscopic evaluation and duodenal biopsy findings were consistent with celiac disease, and she was advised to adopt a gluten-free diet. During follow-up, her dysphagia worsened, prompting a repeat endoscopy that revealed an ulcerated mass in the midesophagus. Histopathological examination confirmed squamous cell carcinoma. The patient subsequently underwent surgical resection. Her medical history also included rheumatoid arthritis, which required long-term immunomodulatory therapy. This case highlights that celiac disease, beyond being a chronic enteropathy, may predispose individuals to malignancies in the upper gastrointestinal tract. Chronic mucosal inflammation, altered epithelial regeneration, and persistent immune activation can contribute to carcinogenesis. Moreover, coexisting autoimmune conditions and prolonged immunosuppressive therapy may further increase the risk of malignant transformation. The simultaneous presence of celiac disease and esophageal SCC highlights the need for vigilance in monitoring patients with persistent or progressive upper gastrointestinal symptoms.

Celiac disease should be considered in patients presenting with upper gastrointestinal complaints, particularly when associated with autoimmune comorbidities. Early diagnosis and appropriate dietary management may not only improve gastrointestinal health but may also reduce the risk of malignant complications such as esophageal squamous cell carcinoma.

## PS-211 Celiac Disease Presenting with Infertility and Recurrent Pregnancy Loss: A Case Report

**Gülmira Akçay ^1^ , Artuner Varlıbaş ^1^ , Bayram Yeşil ^2^ , Ramis Çatalbaş ^2^ , Dilek Oğuz ^2^**

^1^Department of Internal Medicine, Kırıkkale University School of Medicine, Kırıkkale, Türkiye

^2^Department of Internal Medicine, Division of Gastroenterology, Kırıkkale University School of Medicine, Kırıkkale, Türkiye

Celiac disease is an autoimmune enteropathy triggered by gluten intake in individuals with a genetic predisposition. Although it generally presents with gastrointestinal symptoms, the systemic effects of the disease are often overlooked. In recent years, the relationship between celiac disease and infertility and pregnancy loss has gained increasing attention. Malabsorption, autoimmune mechanisms, and nutritional deficiencies can adversely affect reproductive health. A 54-year-old female patient had been followed up with a diagnosis of idiopathic thrombocytopenic purpura (ITP) for 30 years. She had been receiving corticosteroid and eltrombopag treatment and had undergone splenectomy 20 years earlier. She was admitted with complaints of nausea, dyspepsia, diarrhea, and weight loss. Endoscopic examination revealed duodenal mucosal atrophy; biopsy showed villous shortening and increased intraepithelial lymphocytes (Marsh type 3b), leading to the diagnosis of celiac disease. From the patient’s history, it was noted that she had secondary infertility and recurrent pregnancy losses. Gynecological and hormonal evaluations showed no pathology. After initiating a gluten-free diet, improvement was observed in her gastrointestinal symptoms. This case demonstrates that celiac disease is not limited to the digestive system but can also affect reproductive health. In undiagnosed celiac disease, chronic inflammation, mucosal damage, and micronutrient deficiencies such as folate, iron, and zinc can impair endometrial receptivity and lead to implantation failure, whereas autoimmune oophoritis or endometrial damage may contribute to infertility. Autoimmune processes may negatively affect placental development and fetoplacental circulation, leading to recurrent pregnancy losses. Therefore, in women of reproductive age with unexplained infertility or recurrent pregnancy losses, celiac disease should always be considered.

Early diagnosis and treatment of celiac disease with a gluten-free diet can positively affect not only gastrointestinal recovery but also reproductive health. This case emphasizes the importance of evaluating celiac disease in the differential diagnosis of unexplained pregnancy losses.

## PS-215 Terminal Ileal Relapse of Gastric Diffuse Large B-Cell Lymphoma Detected by Colonoscopy After Remission: A Rare Case

**Şefikcan Biricik ^1^ , Erman Mercan ^1^ , Semra Dağdelen ^1^ , Doğancan Akyürek ^1^ , Murat Yıldırım ^1^ , Merve Ceren Ceylan Eroğlu ^1^ , İlker Şen ^1^ , Emrullah Düzgün Erdem ^1^ , Mehtap Üçer ^1^ , Tuğrul Elverdi ^2^ , Hüseyin Alkım ^1^ , Canan Alkım ^1^**

^1^Department of Gastroenterology, University of Health Sciences, Şişli Hamidiye Etfal Training and Research Hospital, İstanbul, Türkiye

^2^Division of Hematology, Department of Internal Medicine, İstanbul University-Cerrahpaşa Cerrahpaşa Faculty of Medicine, İstanbul, Türkiye

Primary gastric diffuse large B-cell lymphoma (PG-DLBL) is the most common form of extranodal lymphoma. Although R-CHOP-based therapy achieves high remission rates, distant visceral relapses remain extremely rare. A 61-year-old man, diagnosed with gastric DLBL in November 2021, achieved complete remission after R-CHOP and intrathecal methotrexate therapy and had been disease-free for 5 years. He presented with constipation, abdominal pain, loss of appetite, and a weight loss of 4 kg. Gastroscopy revealed only edematous and hyperemic gastritis. Colonoscopy demonstrated a 5- to 6-cm granular, polypoid, and eroded lesion surrounding the ileocecal valve, from which biopsies were taken. PET-CT revealed a hypermetabolic mass in the terminal ileum (69×80 mm, SUVmax 16.7), necrotic mesenteric lymph nodes (SUVmax 12.1), and a hypermetabolic lesion in liver segments 5-6 (SUVmax 18.0). Findings were consistent with systemic relapse involving the terminal ileum and liver. Relapses of PG-DLBL are typically local or regional. Systemic visceral relapses, particularly involving the terminal ileum, mesenteric lymph nodes, or liver, are exceedingly rare and have been reported only in isolated case reports. In this patient, multiple systemic relapses occurred after 5 years of remission. Colonoscopy and PET-CT played a decisive role in identifying both the relapse focus and visceral dissemination. This case emphasizes the importance of evaluating not only the upper but also the lower gastrointestinal tract and visceral organs through metabolic imaging in patients presenting with new gastrointestinal symptoms during follow-up. Systemic relapse after remission in gastric DLBL is rare but possible. In patients with new gastrointestinal complaints, comprehensive evaluation including colonoscopy and PET-CT should be considered.


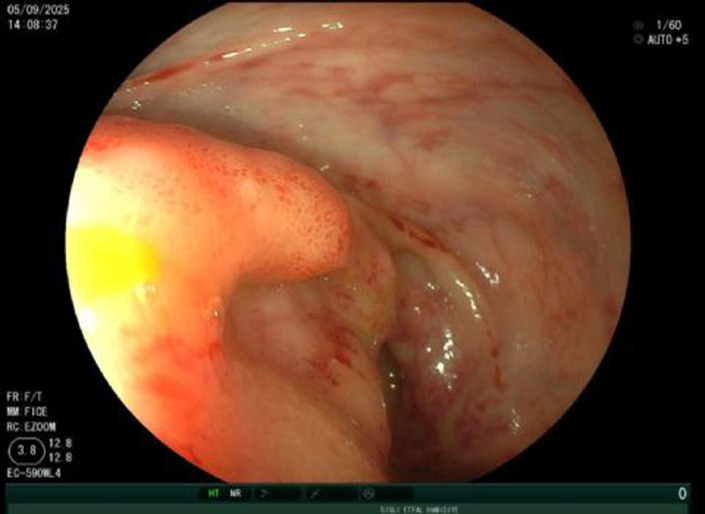


Figure 1. Ileocecal valve.

## PS-216 Endoscopic Ultrasound-Guided Percutaneous Endoscopic Gastrostomy

**Harun Yetimoğlu, Fatih Albayrak, Hakan Dursun, Mehmet Davutoğlu**

Department of Gastroenterology, Atatürk University Faculty of Medicine, Erzurum, Türkiye

Percutaneous endoscopic gastrostomy (PEG) is the preferred method for nutritional support in patients with normal gastrointestinal function who require long-term enteral feeding. Patients with a history of laparotomy or those without abdominal wall transillumination during endoscopy are at high risk for complications from the PEG procedure. In patients who cannot be treated with the standard technique, PEG can be performed with EUS guidance. A 70-year-old patient diagnosed with Parkinson’s disease underwent percutaneous endoscopic gastrojejunostomy (PEG-J) catheter placement to receive Levodopa/Carbidopa Intestinal Gel therapy. This procedure was not performed because abdominal wall transillumination was not possible with the standard technique. The PEG-J catheter placement under endoscopic ultrasound guidance was presented. Although the studies are very limited, they suggest that PEG catheter insertion can be performed under EUS guidance in patients who have undergone extensive laparotomy without transillumination or who are considered to be at high risk for PEG, without relying on another operator and without resorting to more invasive gastrostomy methods.

## PS-217 Adult-Onset Celiac Crisis Presenting with Severe Metabolic Acidosis and Malnutrition: A Case Report

**Aylin Suran ^1^ , Ceyhun Solakoğlu ^2^ , Erkan Kaya ^2^ , Ünsal Arif ^2^ , Hasan Fıstıkçıoğlu ^2^ , Süha Göksel ^3^ , Suna Yapalı ^4^**

^1^Acıbadem Mehmet Ali Aydınlar University School of Medicine, İstanbul, Türkiye

^2^Acıbadem Dr. Şinasi Can Hospital, İstanbul, Türkiye

^3^Acıbadem Maslak Hospital, İstanbul, Türkiye

^4^Department of Gastroenterology, Acıbadem Mehmet Ali Aydınlar University, İstanbul, Türkiye

Celiac crisis (CC) represents an acute, severe, and potentially life-threatening manifestation of celiac disease (CD), characterized by profuse diarrhea, dehydration, electrolyte imbalance, and metabolic acidosis. It is exceptionally rare in adults and often misdiagnosed as infectious enteritis or other gastrointestinal disorders. Herein, an adult patient diagnosed with CD who presented with severe metabolic acidosis and malnutrition was reported. A 33-year-old woman was admitted with chronic diarrhea, dehydration, and weakness. Arterial blood gas analysis revealed a pH of 7.10 and HCO_3_^-^ of 9 mmol/L, consistent with severe metabolic acidosis. Laboratory findings included leukocytes 6220/µL, hemoglobin 8.1 g/dL, potassium 2.1 mmol/L, CRP 0.62 mg/dL, albumin 2.4 mg/dL, and PT/INR 32.5 sec/3.01. She was transferred to the ICU and treated empirically with metronidazole and ciprofloxacin. Stool cultures and antigen tests for *Salmonella*, *Shigella*, *Campylobacter*, *C. difficile*, *Giardia*, *Cryptosporidium*, *Entamoeba histolytica*, rotavirus, and adenovirus were negative. Given the lack of improvement, a gastroenterology consultation was obtained. Thyroid, parathyroid, and HIV tests were normal. Iron, folate, and vitamin B12 deficiencies were detected, with normal immunoglobulin levels. Celiac serology revealed anti-tTG IgA: 175 U/mL (N < 20) and positive anti-endomysial antibodies. Endoscopy demonstrated scalloping, a mosaic pattern, loss of duodenal folds, fissuring, and nodularity. Duodenal biopsies showed severe villous atrophy, crypt hyperplasia, and intraepithelial lymphocytosis (Marsh 3c), confirming CD. Ileocolonoscopy and biopsies were normal. A gluten-free diet with vitamin and albumin supplementation led to rapid clinical and biochemical improvement. Celiac crisis, though rare in adults, should be considered in cases of unexplained severe and chronic diarrhea unresponsive to antibiotics, even in the ICU setting. Early recognition and prompt initiation of a gluten-free diet are essential for recovery and the prevention of life-threatening complications.

## PS-218 A Challenging Diagnosis in a Patient Presenting with Ascites: Tuberculous Peritonitis

**Nur İlayda Genç ^1^ , Devrim Müge Özarı Gülnar ^2^ , İbrahim Beyhan ^1^ , Nilüfer Alpay Kanıtez ^3^ , Alper Yurci ^2^**

^1^Department of Internal Medicine, Koç University Hospital, İstanbul, Türkiye

^2^Department of Gastroenterology and Hepatology, Koç University Hospital, İstanbul, Türkiye

^3^Department of Rheumatology, Koç University Hospital, İstanbul, Türkiye

Tuberculous peritonitis (TBP) should be considered in the differential diagnosis of ascites with a serum-ascites albumin gradient (SAAG) <1.1 g/dL. In this report, a case of TBP diagnosed through the growth of *Mycobacterium tuberculosis* in tissue culture despite negative microscopic and PCR results in both ascitic fluid and biopsy samples was presented. The aim is to emphasize the continuing gold-standard role of culture.

A 51-year-old male patient was admitted with complaints of increasing abdominal distension, a 15-kg weight loss, and night sweats. He had no fever. His medical history included hypertension, chronic kidney disease, and gout. There was no history of alcohol use or chronic liver disease. On physical examination, dullness consistent with ascites was detected above the umbilical level, and fine crackles were heard at the lung bases. Laboratory tests revealed normocytic anemia, increased acute-phase reactants, increased creatinine levels, and parathyroid hormone-suppressed hypercalcemia. Liver function tests were normal. Ascitic fluid analysis showed total protein:6.03 g/dL, albumin: 3.2 g/dL, glucose: 85 mg/dL, LDH:232 U/L, and cell count: 650/μL (93% lymphocytes, 7% neutrophils). SAAG was<1.1 g/dL, indicating nonportal hypertensive ascites. Cytologic examination revealed no evidence of malignancy. The ADA level was 59.8 U/L. Abdominal MRI demonstrated peritoneal thickening and an “omental cake” appearance, raising suspicion for metastatic disease or granulomatous infection. Peritoneal biopsy revealed nonnecrotizing granulomatous inflammation. However, Ziehl-Neelsen staining was negative for acid-fast bacilli (AFB), and tissue PCR, Quantiferon test, and ascitic fluid PCR were all negative. Direct microscopy also showed no AFB. Rheumatologic diseases such as sarcoidosis, SLE, and idiopathic granulomatous disorders were considered in the differential diagnosis. ANA, ENA profile, and anti-dsDNA (IFA) were negative, and the ACE level was 63U/L. Because of rapid ascitic accumulation and significant symptoms, prednisolone was initiated at 1 mg/kg/day, leading to a dramatic reduction in ascites within days. In the second week, *Mycobacterium tuberculosis* growth was detected in the tissue culture, confirming the diagnosis of TBP. The patient was started on standard quadruple antituberculosis therapy, and a gradual tapering of prednisolone was planned. Follow-up and treatment are ongoing.

In this case, the increased ADA and granulomatous histopathology were suggestive of TBP, yet the definitive diagnosis was made only by the growth of the bacillus in culture, the gold-standard method. The rapid clinical improvement with prednisolone is not surprising, given its anti-inflammatory effects; however, it should be used cautiously and discontinued early to avoid additional immunosuppression.


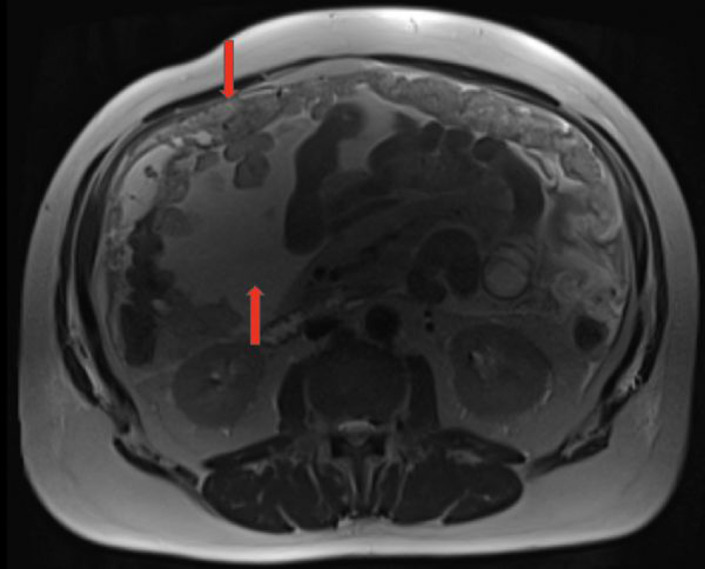


Figure 1. Abdominal MRI revealed diffuse ascites and an omental cake appearance.

Table 1.Laboratory Findings

ADA, adenosine deaminase; CRP, C-reactive protein; HGB, hemoglobin; INR, international normalized ratio; LDH, lactate dehydrogenase; MCV, mean corpuscular volume; PLT, platelet count; PTH, parathyroid hormone; WBC, white blood cell count.

**Table:** 

Hemogram		Biochemistry		Peritoneal Fluid Analysis	
Hemoglobin	10.7 g/dL	Creatinine, serum	2.05 mg/dL	Glucose	85.8 mg/dL
Mean corpuscular volume	82 fL	C-reactive protein	95.3 mg/L	Protein	6.03 g/dL
White blood cell count	4.81 K/uL	Total protein	69 g/L	Lactate dehydrogenase	232 U/L
Platelet count	400 K/uL	Albumin, serum	35.4 g/L	Red blood cells	400 /µL
INR	1.10	Phosphorus (P)	4.2 mg/dL	White blood cells	650 /µL (neutrophils - 7%, lymphocytes - 93%)
Erythrocyte sedimentation rate 1. Saat	55 mm/h	Parathyroid hormone	5.9 pg/mL	Albumin	3.23 g/dL
		25-Hydroxy vitamin D, total	32 ng/mL	Adenosine deaminase (ADA)	59.8 U/L

## PS-219 Anxiety Levels of Patients Undergoing Endoscopic Ultrasound at the Izmir City Hospital Gastroenterology Clinic

**Nuray Yılmaz, Vildan Keten, Emre Büyükçapar, Ayten Parlak, Nuriye Muslu, Filiz Sevin, Leyla Orak**

Department of Gastroenterology, İzmir City Hospital, İzmir, Türkiye

**Background/Aims:** This study was conducted to evaluate the effect of preprocedural information on anxiety levels in patients undergoing EUS.

**Materials and Methods:** A total of 100 patients were enrolled in the Endoscopy Unit of the Gastroenterology Clinic of İzmir City Hospital. Fifty patients were informed about the procedure beforehand, whereas 50 patients underwent the procedure without additional information. All participants completed a 40-item Likert-type anxiety scale, and a total anxiety score was calculated for each individual.

**Results:** Before comparing the differences between the 2 groups in the statistical analyses, some basic assumptions affecting the selection of the test to be used were examined. The total anxiety scores of the informed group ranged from 50 to 129, with a mean anxiety score of 80.84 and a SD of 17.51. In the uninformed group, the mean anxiety score was 74.58, and the SD was 13.43. The Mann–Whitney *U*-test found no statistically significant difference between the groups (U = 1012.0, *P* =.101).

**Conclusion:** These findings highlight several clinically important points. First, it appears that providing patient information may not always reduce anxiety levels. Although detailed information about the procedure may reduce uncertainty for some individuals, it can increase anxiety for others. Second, the lack of significance suggests that the relationship between information and anxiety is multidimensional, influenced by individual differences such as personality traits, previous experiences, and health concerns. In conclusion, it is evident that providing information in clinical practice is an ethical responsibility and a factor that increases patient satisfaction. However, it is important to recognize that many factors affect anxiety levels, and information processes should be arranged in accordance with the psychological needs of individuals.

## PS-221 Diagnostic Efficacy Comparison Between Fine-Needle Aspiration and Fine-Needle Biopsy in Endoscopic Ultrasound-Guided Lesions

**Asena Uygur ^1^ , Özlem Gökçen ^1^ , Müjde Soytürk ^2^ , Göksel Bengi ^2^**

^1^Department of Nursing, Dokuz Eylul University Faculty of Medicine, İzmir, Türkiye

^2^Gastroenterology Department, Dokuz Eylul University Faculty of Medicine, İzmir, Türkiye

**Background/Aims:** Endoscopic ultrasound (EUS) is the gold standard for evaluating deep-seated lesions such as those in the pancreas, liver, and mediastinum, offering high-resolution imaging and targeted tissue acquisition. Fine-needle aspiration (FNA) has long been used for cytological diagnosis, whereas the newer fine-needle biopsy (FNB) technique aims to preserve histological structure and improve diagnostic accuracy.

This study compares the diagnostic yield and histopathological distribution of EUS-guided FNA and FNB samples at the center.

**Materials-Methods:** A total of 107 patients who underwent EUS-guided sampling between January and September 2025 were retrospectively evaluated. FNA was performed in 86 cases, and FNB in 21 cases. Pathology results were reviewed, diagnostic adequacy rates were calculated, and both groups were statistically compared.

**Results:** In the FNA group, the most frequent diagnosis was adenocarcinoma (n = 22), followed by insufficient samples/fibrosis (n = 26), benign epithelial cells (n = 10), malignant epithelial cells (n = 8), and several rare entities including HCC, NET, B-cell lymphoid neoplasia, mucinous cyst, ectopic pancreas, poorly differentiated carcinoma, solid pseudopapillary tumor, mesothelioma, and malignant lymphoma.

In the FNB group, the findings included adenocarcinoma (n = 10), RCC (n = 2), malignant cells (n = 2), well-differentiated NET (n = 1), B-cell lymphoid hyperplasia (n = 1), granulomatous inflammation (n = 1), fibrosis/chronic inflammation (n = 1), and hypocellular/insufficient sample (n = 1).

The diagnostic adequacy rate was 70% (60/86) for FNA and 95% (20/21) for FNB, showing a statistically significant difference (*P* < .05).

**Conclusion:** EUS-guided FNB provides higher diagnostic accuracy and sample adequacy than FNA. By preserving tissue architecture, FNB facilitates histopathological evaluation and reduces the need for repeat sampling. These results indicate that FNB should be considered the preferred method for EUS-guided tissue acquisition in clinical practice.

**Table:** 

Characteristics	Intervention Group (n = 32)	Control Group (n = 32)	*P*
Physical and demographic			
Age, years	37.81 ± 10.71	37.06 ± 9.80	.76^a^
Body mass index, kg/m^2^	25.94 ± 1.79	25.68 ± 1.67	.54^a^
Sex, n (%)			
Women	20 (62.5)	20 (62.5)	.99^b^
Men	12 (37.5)	12 (37.5)	
Education level, years	13.03 ± 5.12	14.25 ± 3.94	.87^a^
Marital status, n (%)			
Married	20 (62.5)	19 (59.4)	.79^b^
Single	12 (37.5)	13 (40.6)	
Working status, n (%)			
Employed	20 (62.5)	21 (65.6)	.79^b^
Unemployed	12 (37.5)	11 (34.4)	
Lifestyle			
Number of meals/day, n (%)			
≤2	15 (46.9)	17 (53.1)	.80^b^
>2	17 (53.1)	15 (46.9)	
Breakfast meal, yes, n (%)	15 (46.9)	14 (43.8)	.80^b^
Water consumption, n (%)			
≤1	16 (50)	15 (46.9)	.96^b^
1-2	13 (40.6)	14 (43.8)	
≥2	3 (9.4)	3 (9.4)	
Tea, yes, n (%)	28 (87.5)	29 (90.6)	.99^b^
Coffee, yes, n (%)	26 (81.3)	27 (84.4)	.99^b^
Alcohol, yes, n (%)	10 (31.2)	13 (40.6)	.60^b^
Smoking, yes, n (%)	11 (34.4)	13 (40.6)	.79^b^
Physical activity level, MET × minute/week	626.09 ± 319.64	638.65 ± 333.77	.85^a^
Medical			
Systemic/metabolic disease, yes, n (%)	5 (15.6)	3 (9.4)	.70^b^
Abdominal surgery, yes, n (%)	8 (25)	9 (28.1)	.99^b^
Laxative use, yes, n (%)	18 (56.3)	17 (53.1)	.99^b^
Family history of FC, yes, n (%)	22 (68.8)	24 (75)	.78^b^

**Table:** 

Primary Outcome Measure	Time Point	Intervention Group (n = 32)	Control Group (n = 32)	*P* ^b^
Defecation frequency/week	Baseline	2.28 ± 1.25	2.34 ± 1.31	**<.001**
	After intervention	6.09 ± 1.02	5.09 ± 1.32	
	***P* ^a^**	**<.001**	**<.001**	

**Table:** 

Secondary Outcome Measures	Time Point	Intervention Group (n = 32)	Control Group (n = 32)	*P* ^b^
Defecation time	Baseline	21.72 ± 5.90	22.97 ± 6.07	**<.001**
	After intervention	7.66 ± 3.10	11.09 ± 3.96	
	***P* ^a^**	**<.001**	**<.001**	
Laxative use/week	Baseline	1.91 ± 1.87	2.13 ± 2.09	**<.001**
	After intervention	0.09 ± 0.39	0.88 ± 1.15	
	***P* ^a^**	**<.001**	**<.001**	
Stool consistency	Baseline	1.44 ± 0.66	1.63 ± 0.90	**<.001**
	After intervention	2.91 ± 0.39	2.06 ± 0.75	
	***P* ^a^**	**<.001**	**<.001**	
Constipation severity	Baseline	34.81 ± 12.85	35.75 ± 10.87	**<.001**
	After intervention	8.72 ± 6.99	16.47 ± 8.19	
	***P* ^a^**	**<.001**	**<.001**	
Gastrointestinal symptom severity	Baseline	23.66 ± 5.51	23.13 ± 5.10	**<.001**
	After intervention	7.03 ± 5.55	13.38 ± 5.29	
	***P* ^a^**	**<.001**	**<.001**	
Quality of life	Baseline	85.19 ± 11.24	84.63 ± 10.04	**<.001**
	After intervention	59.16 ± 11.66	68.47 ± 9.01	
	***P* ^a^**	**<.001**	**<.001**	
Abdominal muscle strength, n (%)	Baseline	<3 15 (46.9) >3 17 (53.1)	<3 13 (40.6) >3 19 (59.4)	**.04 ^c^**
	After intervention	<3 2 (6.3) >3 30 (93.7)	<3 9 (28.1) >3 23 (71.9)	
	***P* ^a^**	**<.001**	**.04**	
Functional exercise capacity	Baseline	566.88 ± 142.48	607.34 ± 157.78	**<.001**
	After intervention	829.38 ± 130.78	703.59 ± 141.86	
	***P* ^a^**	**<.001**	**<.001**	

